# Carbonyl mediated fluorescence in aceno[*n*]helicenones and fluoreno[*n*]helicenes†

**DOI:** 10.1039/d4sc00892h

**Published:** 2024-05-01

**Authors:** Michal Šámal, Ludmilla Sturm, Marzena Banasiewicz, Irena Deperasinska, Boleslaw Kozankiewicz, Olaf Morawski, Yuuya Nagata, Pierre Dechambenoit, Harald Bock, Amandine Rossel, Miloš Buděšínský, Anthony Boudier, Andrej Jančařík

**Affiliations:** a Institute of Organic Chemistry and Biochemistry of the Czech Academy of Sciences 166 10 Prague 6 Czech Republic samal@uochb.cas.cz; b Université de Bordeaux, CNRS, Centre de Recherche Paul Pascal, CRPP UMR 5031 33600 Pessac France andrej.jancarik@crpp.cnrs.fr; c Institute of Physics, Polish Academy of Sciences Al. Lotników 32/46 02-668 Warsaw Poland; d Japan Institute for Chemical Reaction Design and Discovery (WPI-ICReDD), Hokkaido University Sapporo Hokkaido 001-0021 Japan; e Institut de Chimie et Biologie des Membranes et des Nanoobjets (CBMN), Université de Bordeaux-INP UMR 5248, Allée St Hilaire 33607 Pessac Cedex France

## Abstract

Helicenes are very attractive chiral non-planar polycyclic aromatic hydrocarbons possessing strong chiroptical properties. However, most of the helicenes absorb light mainly in the ultraviolet region, with only a small segment in the blue part of the visible spectrum. Furthermore, carbo[*n*]helicenes exhibit only weak luminescence that limits their utilization. Herein, we demonstrate that peripheral decoration of the helicene backbone with an aryl-carbonyl group shifts the absorption to the visible region and simultaneously improves their fluorescence quantum yields. We thus show that the carbonyl group, commonly considered as detrimental to emission, has the capability of improving optical and photophysical properties. Two different families, aceno[*n*]helicenones and fluoreno[*n*]helicenes, are presented with comprehensive spectrochemical characterization. TD-DFT calculations were implemented to clarify their electronic profiles. We show that increasing the helical length in aceno[*n*]helicenes increases absorption onset, *g*_abs_ and *g*_lum_. Extension of the peripheral aromatic part in fluoreno[*n*]helicenes leads to a blue shift in both absorption and emission.

## Abstract 2

Carbo[*n*]helicenes are poor emitters with the emission peak in purple or blue region. Attaching an aryl-carbonyl group to the helical skeleton shifts the absorption and emission to the visible region and improves the fluorescence quantum yield.

## Graphical abstract

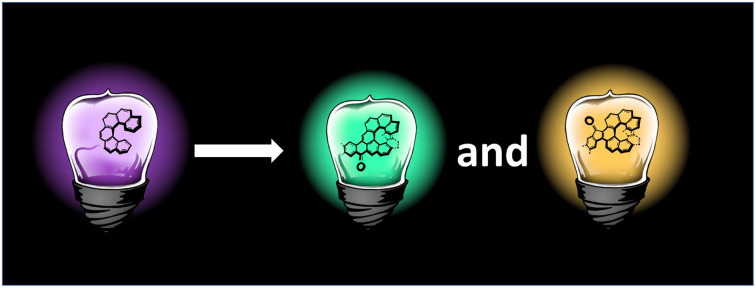

## Introduction

The introduction of a carbonyl group into the aromatic skeleton significantly reduces the HOMO–LUMO gap which is an essential parameter in organic electronics. This effect is often evident by a shift of the absorption and the emission into the visible region. Even relatively short conjugated carbonyl systems are capable of absorption of long-wavelength parts of the visible spectrum. However, carbonyl groups are generally considered as detrimental to the fluorescence quantum yield.^1^ This is due to efficient intersystem crossing (ISC) between singlet and triplet excited manifolds of nπ* and ππ* character. The ISC yield of aromatic ketones is related simultaneously to the very small energetic singlet-triplet gap (<0.2 eV) due to the orthogonal orientation between non-bonding *n* and anti-bonding π* orbitals and to the strong spin–orbit coupling following El-Sayed's rule.^2^ Therefore, most aromatic ketones undergo extremely fast ISC within picoseconds, *e.g.* benzophenone: 5–10 ps,^3^ xanthone: 2 ps,^4^ anthrone: 70 ps.^5^ This results from the proviso that systems which possess low lying singlet ^1^nπ* or ^1^ππ* (S1 and potentially S2) states are coupled with triplet states of similar energy and, intrinsically, different symmetry (^3^ππ* or ^1^nπ*, respectively).^2^ For this reason, the integration of a carbonyl group into the aromatic system would seem counter-intuitive for the purpose of improving the photophysical properties. Thus, the carbonyl group is rarely considered beneficial for the luminescence. Herein we show that the installation of an aryl-carbonyl group at the periphery of the carbo[*n*]helicene helix not only shifts the absorption by more than 100 nm (>4270 cm^−1^) but also significantly increases the fluorescence quantum yield (*Φ*_F_) when compared to pristine carbo[*n*]helicenes. Although helicenes are known to exhibit very remarkable chiroptical properties such as high optical rotations (>±1000), circular dichroism, circularly polarized luminescence and nonlinear optical activity,^6^ they show only weak luminescence: the fluorescence quantum yield (*Φ*_F_) of [6]helicene is *Φ*_F_ = 0.04, [7]helicene *Φ*_F_ = 0.02 and [*n*]helicene (where *n* ≥ 8) *Φ*_F_ ≤ 0.01.^7^ This is mainly due to the small oscillator strength and to significant intersystem crossing (ISC).^8^ Furthermore, the absorption onset wavelength (*λ*_onset_) of helicenes increases initially with increasing length but saturates around 490 nm ([11]helicene). Thus, pristine carbo[*n*]helicenes are colorless (*n* ≤ 6) or yellow (*n* ≥ 7) substances. In order to qualify helicenes for use in various luminescence-based (chiropto)electronic applications such as fluorescent probes, signaling systems, OLEDs, bioimaging *etc.*, it is necessary to improve their luminescence quantum yields and modulate their absorption/emission (HOMO–LUMO gap) on demand. To improve photochemical parameters with emphasis on *Φ*_F_, a number of different approaches have been employed,^9^ such as substitution, hetero-doping, lateral extension, or metal complexation.

**Fig. 1:**
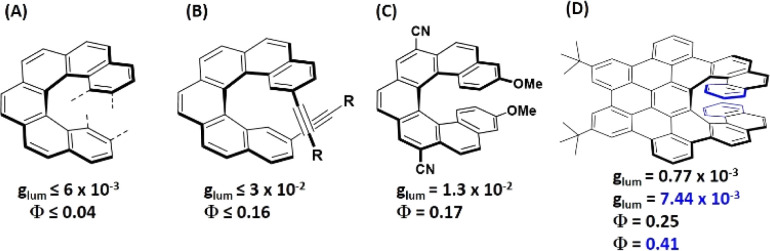
Luminescent properties of helicenes and their derivatives.

**Fig. 2:**
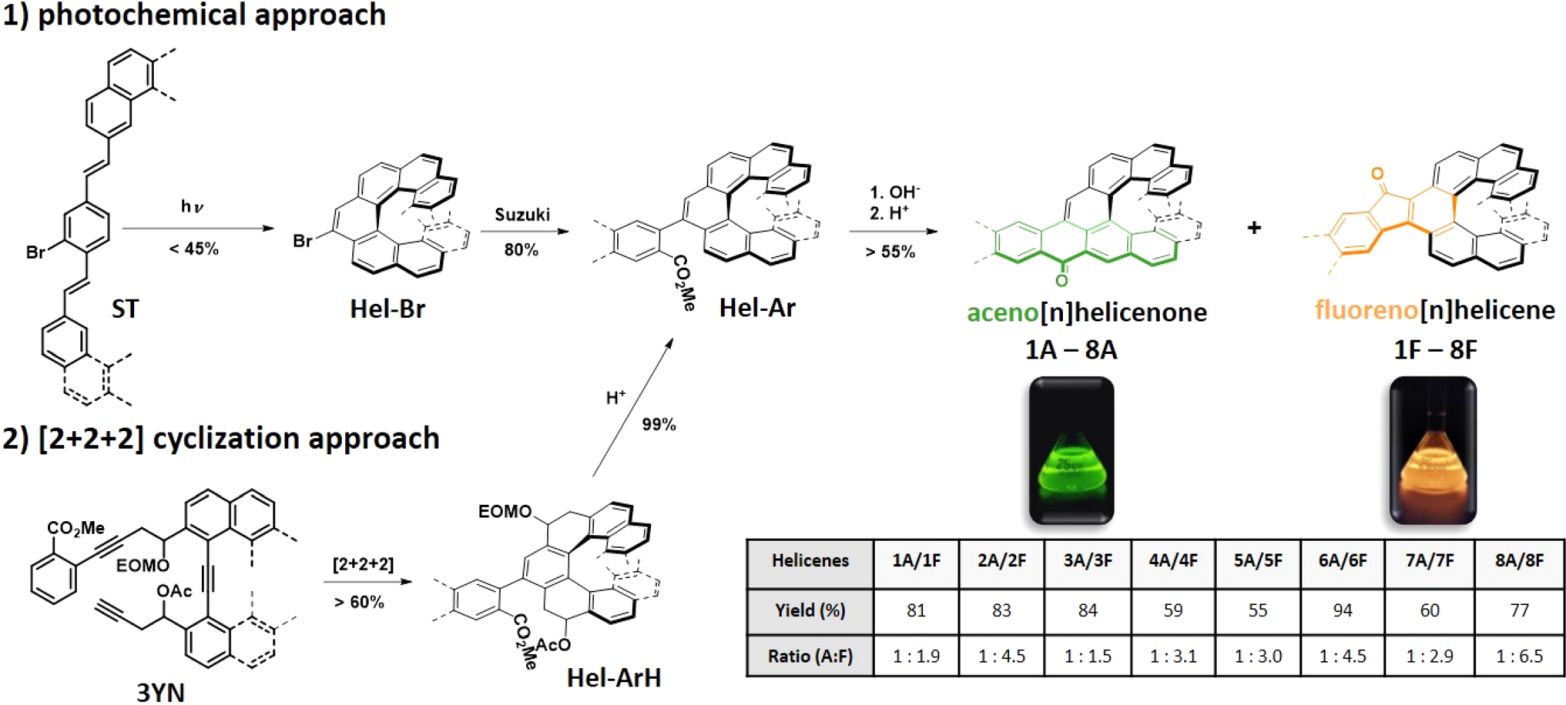
Synthesis of aceno[*n*]helicenones 1A–8A and fluoreno[*n*]helicenes 1F–8F; structures of 1A–8A/1F–8F are shown in Fig. 3, EOM = ethoxymethyl ether.

Crassous, Favereau, Autschbach and coworkers have shown that the functionalization of [6]helicene allows the modulation of the circularly polarized luminescence, the absorption onset and the fluorescence quantum yield (Fig. 1B).^10,11^ Matsuda, Hirose *et al.* have developed [7]helicene derivatives with enhanced circularly polarized luminescence and improved fluorescence quantum yields (Fig. 1C).^12^ In the case of helicenes longer than [7]helicene, only theoretical studies have been conducted to investigate their chiroptical properties. It was shown that the dissymmetry factor of carbo[*n*]helicenes increases with the helix length *n*.^14^ Recently, Narita, Müllen, Pieters and coworkers have demonstrated that a small increase (from *n* = 7 to 9) of the helix length in laterally extended [*n*]helicenes can lead to a 10-fold increase of the dissymmetry factor (Fig. 1D).^13^ Herein, we show that the carbonyl group can do the job of increasing *Φ*_F_ while simultaneously shifting the absorption onset to longer wavelengths. It is striking that although many helicenes bearing a carbonyl moiety have been described, all of them are orange/red solids which exhibit no or very weak fluorescence.^15–19^ The non-emissivity was always attributed to the carbonyl group. Thus fluorenone-fused helicenes have been extensively used only as precursors to synthesize a variety of highly luminescent helicene-like materials such as fluorene-fused helicenes,^15,16^ azahelicenes^20^ or indenofluorenes.^21,22^ In this study we show that installation of fluorenone or acenone units at the periphery of the helicene (Fig. 2) has a very positive effect on their photophysical properties. The absorptions and emissions are shifted into the visible region and the fluorescence quantum yields are greatly enhanced. In addition, our study demonstrates that the established rule that the carbonyl group is detrimental to such properties has important limitations. We show that the carbonyl group can indeed improve the optical and photophysical properties. Furthermore, we show that the absorption and luminescence dissymmetry factors (*g*_abs_ and *g*_lum_) in the obtained systems strongly depend on the helix length *n*, in contrast to pristine helicenes.

## Results and discussion

**Fig. 3:**
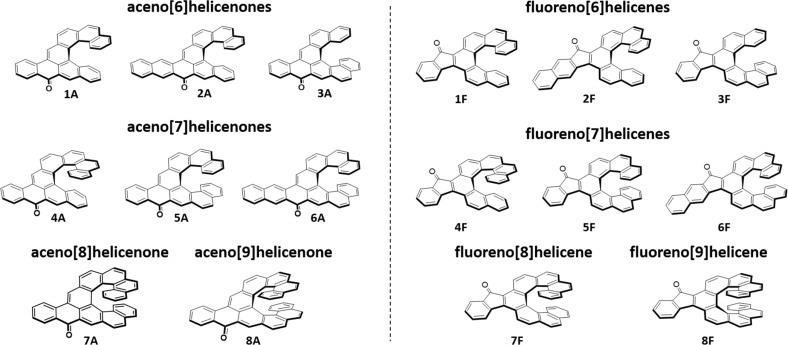
List of aceno[*n*]helicenones 1A–8A and fluoreno[*n*]helicenes 1F–8F.

For this study we have designed and synthesized two families of carbo[*n*]helicenes. The first family, aceno[*n*]helicenones 1A–8A (Fig. 2 and 3), are helicenes that are peripherally annulated *via* an aryl-carbonyl group that formally creates an acene unit. The second family, fluoreno[*n*]helicenes 1F–8F, is annulated such that a fluorenone unit is inserted into the helicene backbone. Installing an aromatic carbonyl group in this way at the periphery of the helicene scaffold has a significant effect on the optical properties of the studied helicenes. For example, the absorption is shifted to the visible region by more than 50 nm (2160–3370 cm^−1^) in the aceno[*n*]helicenone family and more than 100 nm (4270–6000 cm^−1^) in the fluoreno-derivatives, and the fluorescence quantum yields are increased up to six times when compared to the pristine helicenes. We have deployed two versatile approaches providing aceno-/fluoreno[*n*]helicenone series 1A–8A/1F–8F (Fig. 2).

The first approach is based on the Mallory photo-cyclization of suitable bromo-stilbene precursors ST (prepared by Wittig reaction) providing directly bromo-helicenes Hel-Br. The bromo atom fulfils two crucial functions that are (1) deactivation of the *ortho*-position against photocyclization and thus directing the cyclization toward the helicene and (2) being a coupling partner in a subsequent Suzuki reaction. The second approach is based on [2 + 2 + 2] cycloisomerization of suitable tri-ynes 3YN, developed by I. Stará and I. Starý *et al.*^23^ Intramolecular cyclization (catalyzed by a Co^I^ catalyst) followed by elimination/aromatization provides directly arylated helicenes Hel-Ar bearing carboxylic ester group. Basic hydrolysis of the ester group leads to the acids. The helicenic esters Hel-Ar and their corresponding acids consist of two unseparable atropodiastereomers (for details, see ESI† page S158). The final acid-catalyzed cyclization afforded aceno[*n*]helicenones and fluoreno[*n*]helicenes 1A–8A/1F–8F simultaneously in ratios varying between 1 : 1 and 1 : 7. The fluoreno[*n*]helicenes were always formed preferentially. The density functional theory (DFT) calculations at the level of the ωB97X-D/Def2SVP on the formation process of 1A and 1F suggested that one atropodiastereomer preferentially produces 1F, while the other atropodiastereomer affords a mixture of 1A and 1F (see ESI† page S189). This result of the calculation showed a good agreement with the experimental outcomes and corroborates that fluoreno[*n*]helicenones are formed preferentially. The significant difference in polarity of aceno[*n*]helicenones and fluoreno[*n*]helicenes allows us to very effectively separate the two helicenes by traditional column chromatography. All the final structures are well soluble in common organic solvents, and thus they were characterized by proton and carbon nuclear magnetic resonance spectroscopy (^1^H and ^13^C NMR) and high-resolution mass spectrometry (HR-MS). In addition, the structures of the fluoreno[*n*]helicenes 5F, 6F and 8F were unambiguously confirmed by single crystal X-ray diffraction (for details, see ESI† page S165).

### Photophysical and electrochemical properties of aceno[*n*]helicenones 1A–8A

**Fig. 4:**
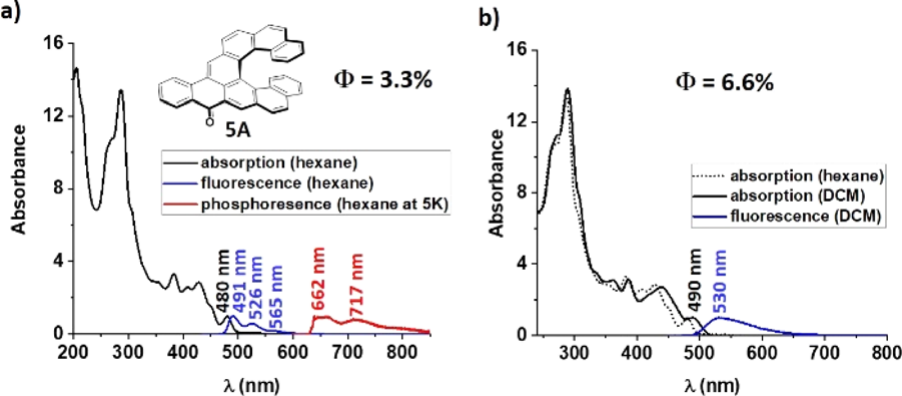
Absorption and emission spectra of 5A in (a) hexane (b) dichloromethane, (*c* ≈ 1 × 10^−5^ M).

**Fig. 5:**
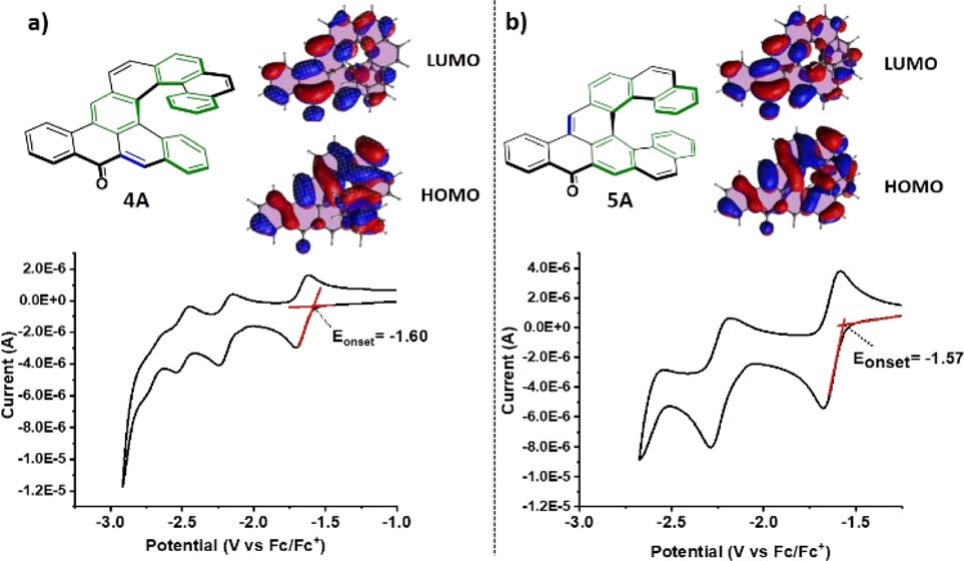
(a) Electronic configuration of 4A and its cyclic voltammogram (b) electronic configuration of 5A and its cyclic voltammogram.

The structures of the series have been designed in order to systematically study how the length of the helicene and acene parts will affect their absorption/emission and their chiroptical properties such as electronic circular dichroism (ECD), circularly polarized luminescence (CPL) and optical rotatory power. Isoelectronic aceno[*n*]helicenones 4A and 5A are unique systems which differ in the position of Clar sextets and in the position of a double bond relative to the carbonyl group (in blue color, Fig. 5a and b). This reveals the relation of the geometry with the physicochemical properties. First, aceno[*n*]helicenones 3A, 5A, 7A and 8A have been investigated to probe the effect of the helicene length on absorption and emission (Table 1). The ultraviolet-visible (UV-Vis) absorption and photoluminescence (PL) spectra were recorded first in nonpolar hexane (10^−5^ M) (as a representative example of 5A, Fig. 4). The electronic absorption spectra of 3A, 5A, 7A and 8A cover the entire ultraviolet region and a major part of the blue region with a maximum located at 460 nm, 480 nm, 489 nm and 504 nm, respectively.

**Table 1:** The evolution of the photophysical properties upon elongation of the helix

Helicene	*λ*_(onset)_ [nm] hexane	*λ*_(em)_ [nm] DCM	*B*_CPL_ c	*g*_lum_ d (10^−3^) DCM (hexane)	[*α*]_D_^20^ DCM	*Φ*_PL_ e (%) hexane/DCM/	*E*_HOMO_ f [eV]	*E*_LUMO_ g [eV]
lA	469	513^a^	6.6	+1.7	+2147	3.6/12.5/11.4	−5.89	−3.21
				−1.5	−2167			
2A	475	534^a^	1.0	+0.58	+770	5.0/8.3/7.6	−5.88	−3.23
				−0.52	−748			
3A	467	494^a^	2.9	+1.2	+1094	1.3/8.5/7.8	−5.95	−3.27
				−1.0	−1031			
4A	481	520^b^	6.0	+3.0	+3493	3.4/8.3/7.6	−5.82	−3.2
				−2.9	−3526			
5A	493	530^a^	4.3	+2.0	+3620	3.3/6.6/5.8	−5.78	−3.23
				−2.0	−3672			
6A	497	546^a^	2.5	+0.82	+2014	7.9/11.2/10.8	−5.78	−3.23
				−0.80	−1945			
7A	501	538^a^	3.9	+3.0	+4789	4.0/4.9/6.8	−5.74	−3.23
				−3.1	−4633			
8A	517	555^a^	12.3	+6.2 (−7.8)	+6015	5.1/6.4/4.9	−5.68	−3.25
				−6.1 (8.0)	−6297			

a Wavelength of excitation 330 nm.

b Wavelength of excitation 336 nm.

c Brightness calculated as *B*_CPL_ = *ε*_max_ × *Φ*_PL_ × ǀ*g*_lum_ǀ/2.

d Concentration *c* ≈ 1 × 10^−5^ M.

e Measured at room temperature (*c* ≈ 1 × 10^−6^ M).

f Calculated as *E*_HOMO_ = *E*_LUMO_ − *E*_(0,0)_.

g Calculated using the equation *E*_LUMO_ = −[
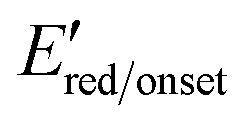
 + 4.8] referenced against Fc/Fc^+^.^24^

These absorption maxima located in the visible light region render these compounds orange. The absorption maximum is red-shifting with each further benzene annulation. A major increase is observed when going from aceno[6]helicenone 3A to aceno[7]helicenone 5A, which is most likely related to the fact that the two terminal rings of the [7]helicene backbone of 5A overlap. This overlap brings additional through-space conjugation and a perceptibly red-shifted absorption. This aceno[*n*]helicenone series follows a regular trend of decrease of the gap (*E*_0,0_) with increase of the length of the π-system (for details, see ESI† Table S1, page S147 and S158). The lowest absorption bands arise from the allowed π → π* transitions with high oscillator strengths (*f* = 0.43 for 3A; *f* = 0.19 for 5A; *f* = 0.16 for 7A and *f* = 0.16 for 8A) and correspond mainly to S_0_ → S_1_ transitions. In nonpolar hexane, the fluorescence spectra of the 1A–8A series show a well-structured vibrational emission band which can be ascribed to a locally excited (LE) state character. Very small Stokes shifts (≤530 cm^−1^, ≤11 nm) are pointing to small changes of the electron distributions in the ground and excited states. In all cases of aceno-derivatives 1A–8A, solvatofluorochromism was observed. The polarity of the environment has only a limited effect on the absorption, causing a small red-shift of the long-wavelength absorption bands. However, passing from low-polarity hexane to polar dichloromethane (DCM) or acetonitrile (ACN) has a pronounced effect on the emission spectra. There is a notable disappearance of the vibrational features of the emission band, accompanied by a strong red-shift. Such large Stokes shifts (1220–2420 cm^−1^, 30–60 nm) indicate that the dipole moment of low-lying excited states (S_1_) is larger than in the ground state (S_0_). According to the calculation results, the dipole moments of aceno-helicenes 1A–8A in the ground state are of the order of 4–5 D, and in the excited state 8–12 D, which corroborate to the experimentally observed Stokes shifts with increasing of solvent polarity (for details, see ESI† page S163). This difference of dipole moments characterizes an electron–donor–acceptor (EDA) system. In our case, the carbonyl group behaves as an acceptor unit owing to its strong electron-withdrawing effect, and the electron rich helicene wing functions as a donor. This electron flow is visible in the electron density distribution of HOMO and LUMO, obtained by density functional theory (DFT) and time-dependent DFT (TD-DFT) calculations based on B3LYP functional and 6-31g(d) basis set. Although the HOMO and LUMO are extended over the whole molecule, the main density of the HOMO is located on the helicene wing and the main density of the LUMO is distributed over the carbonyl-acene part (Fig. 5a and b). Moreover, the symmetry of these orbitals, compared to the orbitals of the parent helicene, is disturbed and causes a clear increase in oscillator strengths for the transitions between the S_0_ and S_1_ states (for details see ESI† page S162). This is accompanied by an increase in the fluorescence yield of aceno-helicenes 1A–8A. However, the fluorescence yield is limited by non-radiative intersystem crossing processes taking place by different channels, *i.e.* to the numerous triplet states with energies close to that of the S_1_ state. However, the effect is less prominent as the length of the helix increases. For instance, there is a six-fold *Φ*_F_ increase in a case of aceno[6]helicenone 3A in polar DCM (*Φ*_F_ = 0.085) or ACN (*Φ*_F_ = 0.078), when compared to the value in nonpolar hexane (*Φ*_F_ = 0.013), while for aceno[9]helicenone the *Φ*_F_ are comparable in nonpolar or polar environment (for details see the Table 1 and S147†). The emission is characterized by short fluorescence lifetimes (2–4 ns) of all the aceno-derivatives 1A–8A, much shorter than the lifetimes of the corresponding [*n*]helicenes (10–15 ns).^25^ In the next step, we have investigated effect of the acene length on the photophysical properties. For this purpose, the aceno-derivatives 1A, 2A, 5A and 6A were synthesized. In the acene series, the absorption maximum systematically increases by *ca.* 100 nm with each additional benzene ring.^26,27^ In contrast, increasing the length of the acene part in the aceno[*n*]helicenones by one benzene ring leads to a smaller bathochromic shift than the one caused by an increase in the length of the helicene part. A linear one-benzene ring annulation of the acene part red-shifts the absorption maximum by only 4 nm (1A → 2A, 459 nm → 463 nm or 5A → 6A, 480 nm → 484 nm), whereas elongation of the helicene part by one benzene ring red-shifts the absorption maximum by 9 nm (1A → 4A, 459 nm → 468 nm or 5A → 7A, 480 nm → 489 nm). We assume that this is due to the extra through-space conjugation caused by the overlap of the terminal helicene rings. Linear benzannullation of the acene part increases the *Φ*_F_ of 2A and 6A in nonpolar hexane (*Φ*_F_ of 1A → 2A: 0.036 → 0.050 and 5A → 6A: 0.033 → 0.079). The fluorescence quantum yield of 2A decreases but that of 6A increases in polar DCM (*Φ*_F_ of 1A → 2A, 0.125 → 0.083 and 5A → 6A, 0.066 → 0.112). Last but not least, the isoelectronic derivatives 4A and 5A allow us to study the relation between the geometry and the physicochemical properties. Both structures are composed of tetracene and [7]helicene units. They differ in the position of the ring with double bond character (in blue color, Fig. 5a and b) relative to the carbonyl group. This position can be controlled by the arrangement of the tetracene and [7]helicene units and thus by localization of the Clar π-sextets in the helicene part (green color, Fig. 5a and b). The position and thus the conjugation of the double bond to the carbonyl group may affect the physicochemical properties of the whole aromatic system. It can also have consequences for the chemical reactivity, for instance 4A and 5A may behave differently upon nucleophilic attack (1,2- *vs.* 1,4-addition).^28,29^ We found that most of the photophysical and chiroptical properties of 4A and 5A are comparable and only the absorbance maxima of 5A (480 nm, double bond in *para*-position to the carbonyl) is red-shifted by 12 nm when compared to 4A (468 nm, double bond in *ortho*-position to the carbonyl). However, the geometry strongly affects the redox behavior, as evidenced by cyclic voltammetry (CV).

All the aceno-derivatives 1A–8A exhibit weak phosphorescence in hexane at 5 K, but this emission is absent in DCM. The phosphorescence emission band has two maxima in the 600–800 nm range. The singlet–triplet gap (Δ*E*_S0–T1_) was determined from the fluorescence and phosphorescence onset wavelengths. Plotting the singlet-triplet gaps (Δ*E*_T1–S0_) of the aceno[*n*]helicenones 3A, 5A, 7A and 8A we observed a systematic decrease with increasing length of the helicene part, which confirms that the energy of the triplet state is associated with conjugation length (see ESI† page S158). Likewise, the energy gap Δ*E*_S1–T1_ between the lowest excited singlet (S_1_) and triplet (T_1_) states decreases with increasing length of the helicene part. Surprisingly, elongation of the acene part (1A → 2A or 5A → 6A) leads to an increase of the gap Δ*E*_S0–T1_, in stark contrast to the pristine acenes.^30^ The energy-state level diagrams of 1A–8A were obtained by TD-DFT calculations. All energy diagrams exhibit that higher triplet states (T_3_, T_4_ and potentially T_5_) are energetically close (Δ*E*_S1–T4/3_ ≤ 0.1 eV) to the S_1_ state and may constitute a main channel for non-radiative intersystem crossing (ISC, S_1_ → T_*n*_). The resulting population of the T_1_ state (by nonradiative T_*n*_ → T_1_) is manifested by the emission of phosphorescence. The efficient ISC (together with the nonradiative internal conversion, S_1_ → S_0_) compete with the radiative depopulation of the S_1_ state, and the result is reduced quantum yield of the fluorescence emission.

Cyclic voltammetry (CV) measurements were performed in acetonitrile to determine the redox properties of 1A–8A. All exhibit two reduction processes, except for 4A and 8A, which exhibit three reversible reductions. The first reductions show sharp reversible waves with half-wave potentials around *E*^red^_1/2_ ≈ −1.5 V (for details, see ESI†). The second reduction is irreversible, except for 4A and 8A. 1A–8A all do not show any oxidation wave below +1.0 V (*vs.* Fc/Fc^+^), suggesting a poor electron donating ability of the helicene wing. As already mentioned, isoelectronic 4A and 5A differ only in the position of the double bond (blue color, Fig. 5a and b) relative to the carbonyl group (*para*-/*ortho*-, determined by Clar's aromatic sextets). Interestingly, the systems with a double bond adjacent to the carbonyl group (4A and 8A) exhibit reversible three electron reductions with *E*^red^_1/2_ = −1.66 V, −2.20 V and −2.50 V for 4A and −1.62 V, −2.16 V and −2.53 V for 8A (*vs.* Fc/Fc^+^). This particular geometry renders the doubly reduced species stable at the time scale of the CV. We suppose that the stability of the species after two single electron reductions of 4A and 8A implies that they contain same number of Clar π-sextets (for details, see ESI† page S164). From the onset of the reduction wave, the LUMO energy levels of 1A–8A were calculated. The HOMO energy levels were calculated from the wavelength of the absorption onset.

### Chiroptical properties of aceno[*n*]helicenones 1A–8A

**Fig. 6:**
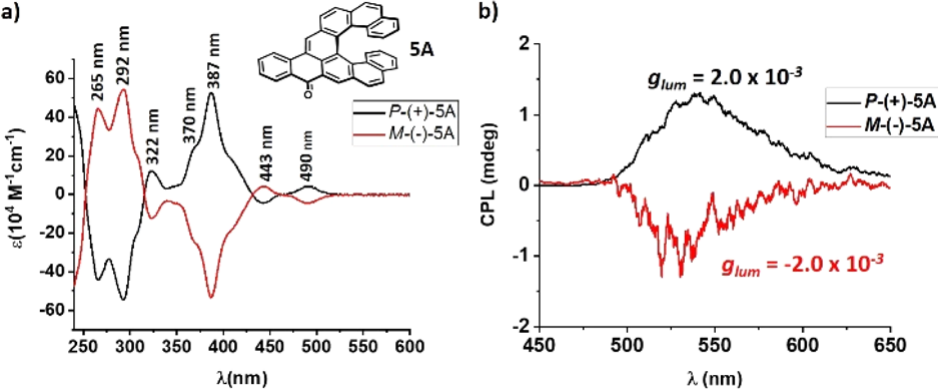
(a) Circular dichroism spectra of 5A in DCM. (b) CPL spectra of (*P*) and (*M*) enantiomers of 5A in DCM, (*c* ≈ 1 × 10^−5^ M).

To investigate chiroptical properties of the aceno[*n*]helicenones 1A–8A, the racemic mixtures were resolved into their corresponding enantiomers by HPLC on a chiral stationary phase column. The absolute configurations were assigned by circular dichroism (CD) spectroscopy with the aid of TD-DFT calculations at the level of CAM-B3LYP/Def2TZVP//CAM-B3LYP/Def2SVP with solvent effect (for details, see ESI† page S159–S161). In all cases the first eluting fraction was assigned as the *P*-(+) and the second one as *M*-(−) enantiomer. 1A–8A all show perfect mirror image CD spectra with strong opposite Cotton effects (see 5A as a representative example in Fig. 6a). The *P*-(+) enantiomers of 3A, 5A, 7A and 8A show a dominant positive Cotton effect in their CD spectra with maximum at 360 nm, 387 nm, 402 and 414 nm, respectively. These positive absorption bands were assigned to S_0_ → S_4–6_ transitions. All the aceno-derivatives exhibit large absorption dissymmetry factors (*g*_abs_) in the range of 3.2 × 10^−3^–13.6 × 10^−3^ (for details see ESI,† page S131–S153). The *g*_abs_ increases as the length of the helicene increases. There is a two-fold increase of the *g*_abs_ when going from tetraceno[6]helicenone 3A (*g*_abs_ = 6.0 × 10^−3^) to tetraceno[9]helicenone 8A (*g*_abs_ = 13.6 × 10^−3^). In contrast, the *g*_abs_ decreases as the length of the acene part increases (1A → 2A, *g*_abs_ = 4.5 × 10^−3^ → 1.9 × 10^−3^; 5A → 6A, *g*_abs_ = 10.0 × 10^−3^ → 6.0 × 10^−3^). All the aceno-derivatives 1A–8A show mirror-image CPL spectra and the sign of the CPL matches that of the CD of the corresponding transition. Based on theory,^31^ the luminescence dissymmetry factor (*g*_lum_) of pristine carbo[*n*]helicenes should increase as the extension of the helix increases. Our calculations and experimental data confirm the same upward trend in aceno[*n*]helicenones. The *g*_lum_ values of 3A, 5A, 7A and 8A derivatives were as follows: *P*-3A, *g*_lum_ = 1.2 × 10^−3^; *P*-5A, *g*_lum_ = 2.0 × 10^−3^; *P*-7A, *g*_lum_ = 3.0 × 10^−3^; *P*-8A, *g*_lum_ = 6.2 × 10^−3^. The polar environment induces an excited CT state in the aceno[*n*]helicenones 1A–8A. Since the CPL reflects the structure of the emissive excited state, we have probed and compared the CPL of the 8A in nonpolar cyclohexane (favoring a LE state) and polar DCM (favoring a CT or a HLCT state). The *g*_lum_ value of *P*-8A in nonpolar cyclohexane (8.0 × 10^−3^) was significantly increased when compared to the polar DCM (6.2 × 10^−3^). Lengthening of the acene part substantially decreases the *g*_lum_ value (*P*-1A → *P*-2A, *g*_lum_ = 1.7 × 10^−3^ → 0.58 × 10^−3^; *P*-5A → *P*-6A, *g*_lum_ = 2.0 × 10^−3^ → 0.82 × 10^−3^). The rotatory power of 1A–8A is lower when compared to the parent carbo[*n*]helicenes, but with a similar trend, *i.e.* the specific rotation is increasing with extension of the helix. For instance, the specific rotation is increased by a factor of six when going from [6]helicene *P*-3A (+1094 deg cm^2^ g^−1^) to [9]helicene *P*-8A (+6015 deg cm^2^ g^−1^). The lengthening of the acene part has the opposite effect, *i.e.* the specific rotation is decreasing with the length of the acene part (*P*-1A → *P*-2A, +2147 → +770 deg cm^2^ g^−1^; *P*-5A → *P*-6A, +3620 → +2014 deg cm^2^ g^−1^).

### Photophysical and electrochemical properties of fluoreno[*n*]helicenes 1F–8F

**Fig. 7:**
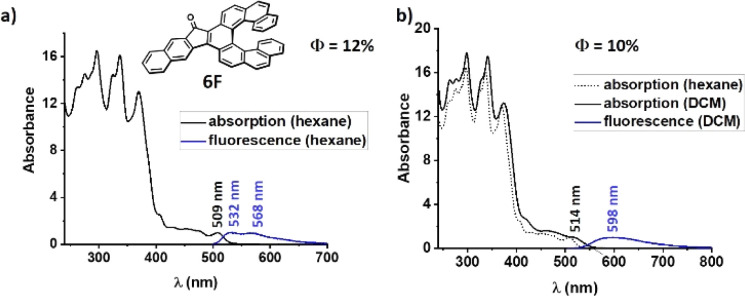
(a) Absorption and emission spectra of 6F in nonpolar hexane. (b) Absorption and emission spectra of 6F in polar DCM, (*c* ≈ 1 × 10^−5^ M).

The absorption onset of the fluoreno[*n*]helicenes 1F–8F is located at longer wavelengths than 500 nm, which makes them red in the solid state (Fig. 7). The absorption onset is systematically red-shifted with each benzannulation of the helix. There is increase an of 54 nm when going form [6]helicene 3F to [9]helicene 8F (3F*λ*_onset_ = 534 nm, 5F*λ*_onset_ = 549 nm, 7F*λ*_onset_ = 560 nm, 8F*λ*_onset_ = 588 nm). Interestingly, the biggest increment (28 nm) is observed when going from [8]helicene 7F to [9]helicene 8F and not when going from [6]helicene 3F to [7]helicene 5F, where the terminal rings start to overlap and extra through space-conjugation appears. Unfortunately, we do not have a satisfying explanation for that observation at this moment. The longest absorption band (assigned to π → π*) of the fluoreno-derivatives is broad and featureless, except for 2F, 3F and 6F, whose spectra are more structured.

While the absorption spectra of 2F, 3F and 6F red-shift and broaden with increasing polarity, those of the other fluoreno homologs are not modified by changing the polarity of the environment, suggesting that there are no significant changes of the electron distributions in the ground state. The emission spectra of 1F–8F are broad (spanning over 200 nm) with two weakly visible maxima in nonpolar hexane. The emission spectra in polar DCM are broad and featureless with very large Stokes shifts (2730–5350 cm^−1^). This suggests that the excited state S_1_ has partial LE character in a low-polar environment and CT character in a polar media. In this system the fluorenone substructure behaves as an acceptor group owing to the strong electron-withdrawing effect of the carbonyl, and the electron rich helicene wings behave as a donor. 1F–8F all exhibit moderate fluorescence in nonpolar hexane and very weak emission (except 2F and 6F, see below) in a polar environment. This difference is governed mainly by the higher non-radiative decay rate constant *k*_nr_ in polar media, whereas the radiative rate constant *k*_r_ is independent of the polarity. The *k*_nr_ increases as the length of the helicene increases, whereas *k*_r_ remains almost unchanged, and thus the *Φ*_F_ decreases from [6]helicene 3F (*Φ*_F_ = 0.115) to [9]helicene 8F (*Φ*_F_ = 0.029). The fluorescence lifetimes (5–10 ns) are higher than in the aceno[*n*]helicenones 1 A-8A (2–4 ns) but lower than for the parent helicenes (10–15 ns).^25^ Annulation of one benzene ring in the linear direction of the fluorenone affects considerably the photophysical properties. Primarily, extension of 1F and 5F by one benzene ring leads to a blue-shift of the absorption by 20 nm (1F → 2F, 535 nm → 515 nm; 5F → 6F, 549 nm → 528 nm, Table 2). This hypsochromic shift is due to both electronic and geometric effects. The electronic effect includes an increase of the LUMO energy caused by a supplementary electron donation from the new annulated ring to the carbonyl group. This extra electron density lowers the carbonyl (C=O) bond strength, as confirmed by infrared spectroscopy. The stretching frequencies of 1F (*ν*_CO_ = 1700 cm^−1^) and 5F (*ν*_CO_ = 1700 cm^−1^) are higher than those of 2F (*ν*_CO_ = 1692 cm^−1^) and 6F (*ν*_CO_ = 1692 cm^−1^), respectively. The length and thus the bond strength of the carbonyl group of 5F and 6F (5F = 1.220 Å *vs.*6F = 1.226 Å) was probed by single crystal XRD which confirmed the donating effect of the extra benzene ring. Interestingly, the one benzene ring annulation (5F → 6F) significantly increases the distance of the two terminal rings in solid state (distance between the centroids of the overlapping benzene rings A–C, 5F_A–C_ = 3.814 Å, 6F_A–C_ = 4.047 Å, for details, see ESI† page S165) of the helicene backbone, with a disruptive effect on the through-space conjugation. However theoretical calculations do not show such a large distance between the terminal rings (5F_A–C_ = 3.71 Å; 6F_A–C_ = 3.70 Å, calculated at the level wB97X-D/Def2SVP using Gaussian 16 Rev C.02). The extra benzene ring has a strong effect on the *Φ*_F_ both in nonpolar (*Φ*_F_ in hexane of 1F → 2F, 0.055 → 0.22 and 5F → 6F, 0.042 → 0.12) and in polar environment (*Φ*_F_ in DCM of 1F → 2F, 0.006 → 0.139 and 5F → 6F, 0.006 → 0.10). The oscillator strength was only slightly improved, but the non-radiative decay rate constant *k*_nr_ was drastically reduced in both nonpolar and polar media, leading to an increased *Φ*_F_. These fluoreno[*n*]helicenes show a regular trend of decreasing gap (*E*_0,0_) with increasing length of the helicene. The decrease of the gap, when going from fluoreno[6]helicene 3F to fluoreno[6]helicene 8F is less prominent (*E*_0,0_3F–8F = 0.19 eV) than for the homologous aceno[*n*]helicenones 3A and 8A (*E*_0,0_3A–8A = 0.25 eV). 1F–8F do not exhibit phosphorescence even at 5K. This can be explained by their energy-level diagram profiles. All the diagrams of 1F–8F have similar profiles and show that there is only one triplet state below the lowest excited state S_1_. All the higher triplet states are well above the S_1_. Thus, the ISC from the S_1_ to T_2_ and to higher triplet states is endothermic and suppressed at low temperatures. T_1_ is the only triplet state which could participate in ISC from S_1_. However, the relatively high energy difference (*E*_S1–T1_ > 0.4 eV) and similar nature of S_1_ and T_1_ states (spin–orbit-coupling, SOC, in case of 6F = 0.143 and 7F = 0.124) renders ISC inefficient. Therefore, the internal conversion from S_1_ to S_0_ becomes the only nonradiative deactivation channel. Based on our experiments and theoretical calculations, the Δ*E*_S0–T1_ gap systematically decreases as the length of the helicene part increases, as the energy of the triplet state is associated with conjugation length (see ESI† page S158). The energy of T_1_ is decreasing to the same extent as the energy of S_1_, thus the energy gap Δ*E*_S1–T1_ remains constant as the length of the helix increases. Benzannulation of the fluorene part lifts the S_1_ but has only a minuscule effect on the T_1_, thus there is an increase of the Δ*E*_S1–T1_ gap. CV measurements were performed to determine the electrochemical properties of 1F–8F. All exhibit two reversible reduction waves. From the onset of the reduction wave, the LUMO energy levels of 1F–8F were calculated. The HOMO energy levels were calculated from the absorption onset wavelength, *i.e.* from the optical gap (for details, see ESI† Table S2 page S157).

**Table 2:** The evolution of the photophysical properties upon elongation of the helix

Helicene	*λ*_(onset)_ [nm] hexane	*λ*_(em)_ [nm] DCM	*B*_CPL_ c (*g*_lum_ × 10^−3^)^d^ DCM	*Φ*_F_ e (%) hexane/DCM/ACN	*E*_HOMO_ f [eV]	*E*_LUMO_ g [eV]
1F	535	545^a^		5.5/0.6/0.6	−5.8	−3.43
2F	515	521^b^		22.0/13.9/13.5	−5.87	−3.43
3F	534	545^a^		11.5/1.9/1.85	−5.79	−3.42
4F	549	557^a^		6.9/1.07/0.76	−5.68	−3.37
5F	549	560^b^		4.22/0.55/0.61	−5.71	−3.4
6F	528	532^b^	8.8 (+3.9/−3.6)	12.0/10.0/8.4	−5.76	−3.37
7F	560	575^a^		6.2/0.86/0.72	−5.69	−3.43
8F	588	600		2.9/0.38/0.70	−5.62	−3.44

a Wavelength of excitation 330 nm.

b Wavelength of excitation 336 nm.

c Brightness calculated as *B*_CPL_ = *Є*_max_ × *Φ*_PL_ × ǀ*g*_lum_ǀ/2.

d Concentration *c* ≈ 1 × 10^−5^ M.

e Measured at room temperature (*c* ≈ 1 × 10^−6^ M).

f Calculated as *E*_HOMO_ = *E*_LUMO_ − *E*_(0,0)_.

g Calculated using the equation *E*_LUMO_ = −[
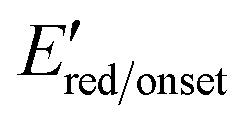
 + 4.8] referenced against Fc/Fc^+^.^24^

### Chiroptical properties of fluoreno[7]helicene 6F

**Fig. 8:**
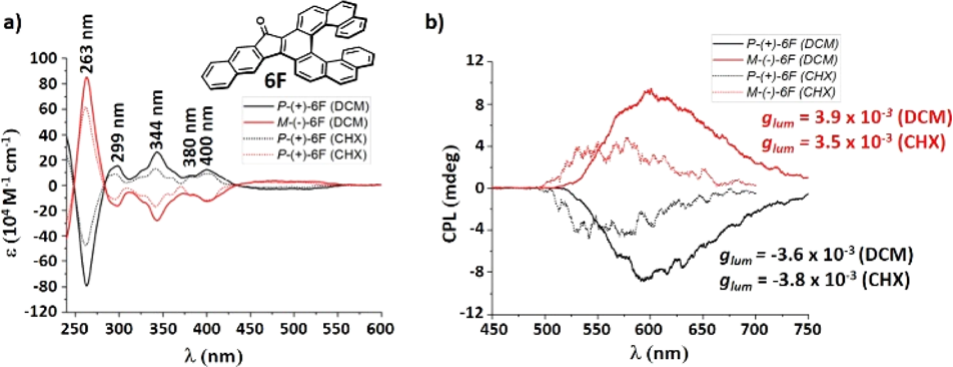
(a) Circular dichroism spectra of 6F. (b) CPL spectra of (*P*) and (*M*) enantiomers of 6F, (*c* ≈ 1 × 10^−5^ M).

We could separate the enantiomers of fluoreno[7]helicene 6F by HPLC on a chiral stationary phase column. The absolute configurations were assigned by CD spectroscopy with the aid of TD-DFT calculations. The first eluting fraction was assigned as the *P*-(+) and the second one as the *M*-(−) enantiomer. The enantiomers give perfectly mirror-symmetric CD spectra (Fig. 8) with opposite Cotton effects, with the maximum at 263 nm and a large dissymmetry factor (*P*-6F, *g*_abs_ = −5.7 × 10^−3^). The longest absorption band at 434–562 nm, with *g*_abs_ = −2.5 × 10^−3^ (*P*-6F), is assigned to the S_0_ → S_1_ transition. The CPL spectra are mirror-symmetric as well, with relatively high *g*_lum_ = 3.9/-3.8 × 10^−3^, and the sign of the CPL matches that of the CD. The absorption and luminescence dissymmetry factors *g*_abs_ and *g*_lum_ are not affected by the polarity of the environment. On the basis of our calculations, the *g*_lum_ of the fluoreno-family is decreasing as the helix increases (3F → 5F → 7F → 8F, *g*_lum_ = 6.3 × 10^−3^ → 6.9 × 10^−3^ → 5.7 × 10^−3^ → 2.5 × 10^−3^) which is the opposite of the aceno[*n*]helicenones 1A–8A. Benzannulation negatively influences the luminescence dissymmetry factor (1F → 2F*g*_lum_ = 7.7 × 10^−3^ → 4.9 × 10^−3^; 5F → 6F*g*_lum_ = 6.9 × 10^−3^ → 4.2 × 10^−3^). The specific rotation of *P*-6F (+434 deg cm^2^ g^−1^) is significantly lower when compared to the parent carbo[7]helicene (±6200 deg cm^2^ g^−1^)^32^ or aceno[7]helicene *P*-6A (+2014 deg cm^2^ g^−1^). Three of the structures 5F, 6F and 8F were unambiguously confirmed by single-crystal XRD analysis. In all cases, suitable crystals were grown from a racemic solution by slow evaporation of cyclohexane : DCM (1 : 1). The interplanar angles of the two terminal rings A and C are 25.2°, 30.5° and 15.7°, respectively (for details, see ESI† page S168).

## Conclusions

In summary, we have made a comprehensive investigation about the structure–property relationship of carbonyl-aceno[*n*]helicenes 1A–8A and carbonyl-fluoreno[*n*]helicenes 1F–8F. We analyzed their structural, photophysical and chiroptical properties using NMR, CV, ECD, CPL and single crystal XRD, and the experimental results were confirmed by TD-DFT calculations. We have shown that the installation of a carbonyl group at the periphery of the carbo[*n*]helicene backbone improves greatly the photophysical properties. The aceno[*n*]helicenones 1A–8A and fluoreno[*n*]helicenes 1F–8F are much more emissive than the parent helicenes. This highlights the limitations of the generally accepted rule of nonemissivity of aromatic carbonyl compounds. Elongation of the helix leads to convergent evolution of the HOMO–LUMO energy gap for both families. Elongation of the acene part leads to an expected red-shift, but benzene ring annulation on the fluorenone part causes a counterintuitive blue shift. This divergent evolution of the energy gaps was corroborated by TD-DFT calculations. CD and CPL have shown large absorption and luminescence dissymmetry factors *g*_abs_ and *g*_lum_, which are dependent on the length of the acene/fluorenone and helicene part. *g*_abs_ and *g*_lum_ substantially increase with the helical length (*n*) in the family of aceno[*n*]helicenones 1A–8A. In contrast, our theoretical calculations show that the *g*_lum_ of fluoreno[*n*]helicenes 1F–8F is decreasing as the length of the helix increases.

## Data availability

The data are provided in the ESI.†

## Author contributions

M. S., A. J., L. S. and A. R. performed all the synthetic work. L. S. and A. B. performed CD, CPL and CV characterization. M. B., B. K. and O. M. performed all the photophysical studies. I. D. did all theoretical calculations. Y. N. calculated dissymmetry factors (*g*_abs_ and *g*_lum_). A. J. wrote the article with assistance of H. B. and I. D.

## Conflicts of interest

There are no conflicts to declare.

## Supplementary Material

SC-015-D4SC00892H-s001

SC-015-D4SC00892H-s002
