# Supplementary material for: Carbonyl mediated fluorescence in aceno[n]helicenones and fluoreno[n]helicenes
Source: Chem Sci. 2024 May 1;15(25):9842–50. doi: 10.1039/d4sc00892h (PMC11206200; doi:10.1039/d4sc00892h)
Supplement: SC-015-D4SC00892H-s001 [file SC-015-D4SC00892H-s001.pdf]

Electronic Supplementary Material (ESI) for Chemical Science.
This journal is © The Royal Society of Chemistry 2024

Carbonyl Mediated Fluorescence in Aceno[n]helicenones and Fluoreno[n]helicenes

Supplementary Information

Michal Šámal,[1]* Ludmilla Sturm,[2] Marzena Banasiewicz,[3] Irena Deperasinska,[3] Boleslaw Kozankiewicz,[3] Olaf Morawski,[3] Yuuya Nagata[4], Pierre Dechambenoit,[2] Harald Bock,[2] Amandine Rossel,[2] Miloš Buděšínský,[1] Anthony Boudier[5] and Andrej Jančařík[2]*

[1] Institute of Organic Chemistry and Biochemistry of the Czech Academy of Sciences, 166 10 Prague 6, Czech Republic

[2] Université de Bordeaux, CNRS, Centre de Recherche Paul Pascal, CRPP, UMR 5031, 33600 Pessac, France

[3] Institute of Physics, Polish Academy of Sciences, Al. Lotników 32/46, 02-668 Warsaw, Poland

[4] Japan Institute for Chemical Reaction Design and Discovery (WPI-ICReDD), Hokkaido University, Sapporo, Hokkaido 001-0021, Japan

[5] Institut Européen de Chimie et Biologie (IECB), Université de Bordeaux-CNRS, UMR 5248, 2 rue Robert Escarpit, F-33607 Pessac Cedex, France

*correspondence to: samal@uochb.cas.cz, andrej.jancarik@crpp.cnrs.fr

Table of Contents

List of abbreviations.. S3

Experimental Section: General Information S4

Synthesis of Aceno- and Fluoreno[n]Helicenones ***rac*-1A(F)–8A(F)**................................ S10

Aceno[6]Helicenone ***rac*-1A** and Fluoreno[6]Helicenone ***rac*-1F** S11

Aceno[6]Helicenone ***rac*-2A** and Fluoreno[6]Helicenone ***rac*-2F** S23

Aceno[6]Helicenone ***rac*-3A** and Fluoreno[6]Helicenone ***rac*-3F** S30

Aceno[7]Helicenone ***rac*-4A** and Fluoreno[7]Helicenone ***rac*-4F**............................. S38

Aceno[7]Helicenone ***rac*-5A** and Fluoreno[7]Helicenone ***rac*-5F**.............................. S44

Aceno[7]Helicenone ***rac*-6A** and Fluoreno[7]Helicenone ***rac*-6F** S49

Aceno[8]Helicenone ***rac*-7A** and Fluoreno[8]Helicenone ***rac*-7F** S53

Aceno[9]Helicenone ***rac*-8A** and Fluoreno[9]Helicenone ***rac*-8F** S58

HPLC Resolution of Helicenones ***rac*-1A-8A** and **6F** into Enantiomers S64

^{1}H and ^{13}C NMR spectra of **1** - **54** S82

Photophysical and chiroptical properties of **1A(F)–8A(F)** S131

Experimental and calculated ECD Spectra of Helicenones **1A-8A** and **6F** S159

Crystalllographic data of **5F**, **6F** and **8F**.. S165

XYZ coordinates and energies of ground and excited states of **1A(F)–8A(F)** S170

Computional Investigation of the Electrophilic Cyclization S189

List of Abbreviations:

S_n (n = 0, 1, 2….)	Singlet States
T_n (n = 0, 1, 2….)	Triplet States
ISC	InterSystem Crossing
fc	vertical transition by Franck-Condon principle
Fc	Ferrocene
Fc^+	Ferrocenium
k_r	radiative rate constant
k_{nr}	nonradiative decay rate constant
Φ_{PL}	photoluminescent quantum yield
t_{fl}	fluorescence lifetime
f	oscillator strength
SOC	spin orbit coupling
g_{abs}	absorption dissymmetry factor
g_{lum}	emission dissymmetry factor
DCM	dichloromethane
CHX	cyclohexane
ACN	acetonitrile
HOMO	highest occupied molecular orbital
LUMO	lowest unoccupied molecular orbital
TDDFT	Time-dependent density-functional theory
CV	cyclic voltammetry

Experimental Section

General: Melting point were determined on Mikro-Heiztisch Polytherm A (Hund, Wetzlar) apparatus and are uncorrected. The NMR spectra were measured on JEOL-400 instrument or Bruker Advance III HD, 400 and 600 instruments, respectively. The ^{1}H NMR spectra were measured at 400.13 MHz and 600.13 MHz, the ^{13}C NMR spectra at 100.61 MHz and 150.90 MHz in $CDCl_3$, CD_2Cl_2 or tetrachloroethane-d_2 as indicated in 5 mm PFG probe with indirect detection. For standardisation of ^{1}H NMR spectra the residual signals of solvent (δ 7.26 for $CHCl_3$, δ 5.32 for CH_2Cl_2, δ 6.00 for tetrachloroethane) was used. In the case of ^{13}C spectra the signal of solvent (δ 77.16 for $CDCl_3$, δ 54.00 for CD_2Cl_2, δ 73.78 for tetrachloroethane-d_2) was used. The chemical shifts are given in δ-scale, the coupling constants *J* are given in Hz. For the correct assignment of both the ^{1}H and ^{13}C NMR spectra of key compounds, homonuclear 2D-H,H-COSY, 2D-H,H-ROESY, and heteronuclear 2D-H,C-HSQC, and 2D-H,C-HMBC experiments were performed. The IR spectra were measured in $CHCl_3$ or KBr. The EI mass spectra were determined at an ionising voltage of 70 eV, the *m/z* values are given along with their relative intensities (%). The standard 70 eV spectra were recorded in the positive ion mode. The sample was dissolved in chloroform, loaded into a quartz cup of the direct probe and inserted into the ion source. The source temperature was 220 °C. For exact mass measurement, the spectrum was internally calibrated using perfluorotri-*n*-butylamine (Heptacosa). The ESI mass spectra were recorded using a quadrupole orthogonal acceleration time-of-flight tandem mass spectrometer (Q-Tof micro, Waters) and high resolution ESI mass spectra using a hybrid FT mass spectrometer combining a linear ion trap MS and the Orbitrap mass analyzer (LTQ Orbitrap XL, Thermo Fisher Scientific). The conditions were optimised for suitable ionisation in the ESI Orbitrap source (sheat gas flow rate 35 a.u., aux gas flow rate 10 a.u. of nitrogen, source voltage 4.3 kV, capillary voltage 40 V, capillary temperature 275 °C, tube lens voltage 155 V). The samples were dissolved in methanol and applied by direct injection. As a mobile phase was used 80% methanol (flow rate 100 µl/min). The APCI mass spectra were recorded using an LTQ Orbitrap XL (Thermo Fisher Scientific) hybrid mass spectrometer equipped with an APCI ion source. The APCI vaporizer and heated capillary temperatures were set to 400 °C and 200 °C, respectively; the corona discharge current was 3.5 µA. Nitrogen served both as the sheath and auxiliary gas at flow rate 55 and 5 arbitary units, respectively. The ionization conditions were the same for low-resolution as well as high-resolution experiment. The HR spectra were aquired at a resolution of 100 000. Optical rotations were measured in CH_2Cl_2 using an Autopol IV instrument (Rudolph Research Analytical). For analytical separations (CSP screening, optimization, and determination of enantiomeric excess), isocratic HPLC systems (Waters Acquity or Knauer Smartline) were used (5 µL injection volume). Waters Acquity PDA (or Knauer Smartline 2500) and IBZ Messtechnik Chiralyser were used as detectors. For semi-preparative resolutions, Puriflash PF5.250 (Interchim) chromatograph equipped with a diode array detector was used.

TLC was performed on Silica gel 60 F_{254}-coated aluminium sheets (Merck) and spots were detected by the solution of $Ce(SO_4)_2 \cdot 4\ H_2O$ (1%) and $H_3P(Mo_3O_{10})_4$ (2%) in sulfuric acid (10%). The flash chromatography was performed on Silica gel 60 (0.040-0.063 mm, Merck). Biotage

Initiator EXP EU (300 W power) was used for reactions carried out in microwave oven. Triethylamine, *N*,*N*-diisopropylamine and dichloromethane were distilled from calcium hydride under nitrogen; tetrahydrofuran was freshly distilled from sodium/benzophenone under nitrogen; toluene was freshly distilled from sodium under nitrogen. Otherwise, all commercially available solvents, catalysts and reagent grade materials were used as received. The starting materials 3-bromo-4-(bromomethyl)benzonitrile, (2-Naphthylmethyl)-(triphenyl)phosphonium bromide, benzaldehyde, 1-bromo-2-naphthaldehyde, 2-bromobenzaldehyde, 2-bromo-1,4-dimethylbenzene, 2-naphthaldehyde, 2-methoxycarbonylphenylboronic acid and 3-bromophenanthrene were purchased, CpCo(CO)(fum) (fum = dimethyl fumarate)[1], aldehyde **16**[2], methyl 2-iodobenzoate[3], aldehyde **27**[2], 4-Bromophenanthrene-3-carbaldehyde[4], methyl 3-iodonaphthalene-2-carboxylate[5] were synthesized according to literature procedure.

Spectroscopic Measurements:

Room temperature measurements were performed with dilute solutions of the compounds poured into a standard (10×10 mm) quartz cuvette. Absorption spectra at room temperature were recorded by using a PerkinElmer Lambda 35 spectrophotometer whereas emission spectra were obtained by using an Edinburgh Instruments FLS 1000 spectrofluorometer. Fluorescence quantum yields (FQY) of the compounds 1A, 2A, 3A, 4A and 5A were measured using as a reference standard Coumarin 153 in EtOH (FQY=0.38). A reference standard for 6A, 7A and 8A was Rh6G in EtOH (FQY=0.94). The absorption and emission spectra of aceno[n]helicenones and fluoreno[n]helicenes recorded in acetonitrile are identical with spectra recorded in dichloromethane and thus they are not presented here.

Fluorescence decay traces were recorded using as an excitation source pulsed DeltaDiode lasers (λexc = 303, 336 or 391 nm) which were installed in a Horiba Fluorolog-3 modular spectrofluorometer. Repetition rate of the diodes was either 8 or 16 MHz. Fluorescence decay curves observed at the maxima of the spectra were recorded with the aid of "time correlated" single photon counting technique (TCSPC). Decay times were determined by using a HORIBA Scientific decay analysis software – DAS6 program, which iteratively fits a theoretical curve to an experimental decay. Estimated accuracy of the obtained decay times was 50 ps.

Emission experiments at 5 K were done using a homemade liquid helium cryostat. Liquid solutions of the investigated compounds (concentrations ~10^{-5} M) were poured into a homemade, cylinder-shaped fused silica glass cuvette (inner diameter 4 mm) and quickly

---

[1] A. Geny, N. Agenet, L. Iannazzo, M. Malacria, C. Aubert, V. Gandon, *Angew. Chem. Int. Ed.* **2009**, *48*, 1810.
[2] M. Šámal, S. Chercheja, J. Rybáček, J. Vacek Chocholoušová, J. Vacek, L. Bednárová, D. Šaman, I. G. Stará, I. Starý, *J. Am. Chem. Soc.* **2015**, *137*, 8469.
[3] C. M. G. Azevedo, C. M. M. Afonso, J. X. Soares, S. Reis, D. Sousa, R. T. Lime, M. H. Vasconcelos, M. Pedro, J. Barbosa, L. Gales, M. M. M. Pinto, *Eur. J. Med. Chem.* **2013**, *69*, 798.
[4] E. Kaneko, Y. Matsumoto, K. Kamikawa, *Chem. Eur. J.* **2013**, *19*, 11837.
[5] C. Bosset, R. Coffinier, P. A. Peixoto, M. El Assal, L. Pouysegu, S. Quideau, K. Miqueu, J.-M. Sotiropoulos, *Angew. Chem. Int. Ed.* **2014**, *53*, 9860.

immersed into liquid nitrogen bath (77 K) in order to prevent aggregation. Cuvette with a frozen sample was quickly transferred into the cold cryostat which was in the center of a homemade synchronous-choppers spectrophoshorometer (frequency of the light choppering 800 Hz)[6]. Excitation source during the low-temperature experiments was either Lambdawave 395 nm (300mW) or 375 nm (200mW) diode lasers. Emission light was dispersed with the aid of a McPherson 207 monochromator and detected with a Hamamatsu H10721-20 photomultiplier (operating in photons counting mode). Phosphorescence decay curves were measured by using a "third chopper" method and accumulated with the aid of a Stanford Research SR-430 Multichannel Scaler.

Crystallographic data were collected with a Bruker APEX II Quasar diffractometer, equipped with a graphite monochromator centred on the path of MoK_{α} radiation. Single crystals, made by slow evaporation of cyclohexane:DCM (1:1)., were coated with Cargille™ NHV immersion oil and mounted on a fiber loop, followed by data collection at 120 K. The program SAINT was used to integrate the data, which was thereafter corrected using SADABS.[7] The structure was solved using SHELXT[8] and refined by a full-matrix least-squares method on F^2 using SHELXL-2018.[9] All non-hydrogen atoms were refined with anisotropic displacement parameters, whereas hydrogen atoms were assigned to ideal positions and refined isotropically using a suitable riding model. 8F crystallizes in the chiral $P2_12_12_1$ space group. However, due to the source and the absence of heavy atom, the absolute structure cannot be determined reliably (meaningless calculated Flack parameter).

The crystallographic data are listed in Table S3 (page S164) and the ORTEP-type views depicted page S164. The CIF files have been deposited at the Cambridge Crystallographic Data Centre as supplementary publication no. CCDC 2295945-2295947.

Electrochemistry: Cyclic voltammetry (CV) measurements were performed with a Metrohm Autolab PGSTAT101 equipped with a platinum working electrode. Ferrocene was used as aninternal reference. Measurements were done in CH_3CN or DMF with 0.1 M (n-$Bu_4N)PF_6$ as supporting electrolyte and at scan rates ranging from of 0.01 to 0.2 V/s.

Circular dichroism spectra were collected with JASCO_J-815. Samples were measeured in dichloromethane in specific cases in cyclohexane. Concentration of the samples were cca $1x10^{-5}$.

Circularly polarized luminescence was collected with JASCO_CPL-300. Emissions of all the anantiomers were measured in dichloromethane (M ≈ $1x10^{-5}$) and in specific cases in cyclohexane. Each presented CPL spectra is avarage of twenty emission spectra.

Theoretical calculations: Quantum chemistry calculations at the density functional theory (DFT and TDDFT) level were performed using the Gaussian 16 package [Gaussian]. The PCM procedure was used for description of solvent effects. The structure of each molecule was optimized in the ground electronic state (S0) and the

[6] B. Kozankiewicz, J. Prochorow, *Mol. Cryst. Liq. Cryst.* **1987**, *148*, 93.
[7] G. M. Sheldrick, SADABS Version 2.03, Bruker Analytical X-Ray Systems, Madison, WI, USA, 2000
[8] G. M. Sheldrick, *Acta Cryst.* **2015**, *A71*, 3-8
[9] G. M. Sheldrick, *Acta Cryst.* **2015**, *C71*, 3-8.

lowest electronic excited state (S1). The coordinates can be found in the attached table S4 (page S170). After exploratory calculations using various functionals, the M06/6-31G(d,p) method was selected as the one that relatively best reproduces the electronic spectra determined experimentally.

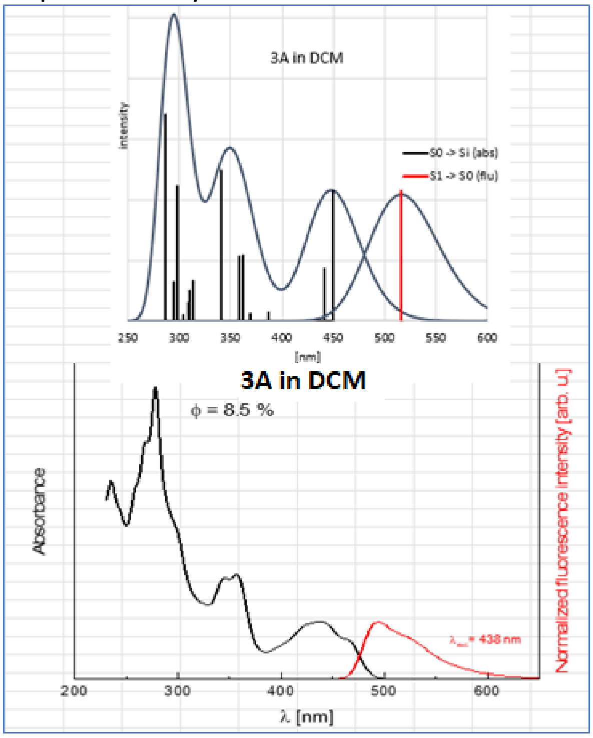

Figure S1. An example - experimental absorption and fluorescence spectra of 3A in DCM and corresponding simulated spectra.)

In the rest of this supplement, the calculation results are presented in the form of energy diagrams, containing information about the energies and the oscillator strengths for S0->S1fc absorption and S1->S0fc fluorescence. These are the so-called vertical electronic transitions in which the molecule does not change geometry, where in absorption it is the geometry of the energy minimum in the ground state, and in fluorescence it is the geometry of the energy minimum in the excited state. The energy diagrams also show systems of triplet states calculated for the S0 geometry.

The Gaussian 16 program was used: Gaussian 16, Revision A.03, M. J. Frisch, G. W. Trucks, H. B. Schlegel, G. E. Scuseria,M. A. Robb, J. R. Cheeseman, G. Scalmani, V. Barone, G. A. Petersson, H. Nakatsuji, X. Li, M. Caricato, A. V. Marenich, J. Bloino, B. G. Janesko, R. Gomperts, B. Mennucci, H. P. Hratchian, J. V. Ortiz, A. F. Izmaylov, J. L. Sonnenberg, D. Williams-Young, F. Ding, F. Lipparini, F. Egidi, J. Goings, B. Peng, A. Petrone, T. Henderson, D. Ranasinghe, V. G. Zakrzewski, J. Gao, N. Rega, G. Zheng, W. Liang, M. Hada, M. Ehara, K. Toyota, R. Fukuda, J. Hasegawa, M. Ishida, T. Nakajima, Y. Honda, O. Kitao, H. Nakai, T. Vreven, K. Throssell, J. A. Montgomery, Jr., J. E. Peralta, F. Ogliaro, M. J. Bearpark, J. J. Heyd, E. N. Brothers, K. N. Kudin, V. N. Staroverov, T. A. Keith, R. Kobayashi, J. Normand, K. Raghavachari, A. P. Rendell, J. C. Burant, S. S. Iyengar, J. Tomasi, M. Cossi, J. M. Millam, M. Klene, C. Adamo, R. Cammi, J. W. Ochterski, R. L. Martin, K. Morokuma, O. Farkas, J. B. Foresman, and D. J. Fox, Gaussian, Inc., Wallingford CT, 2016.

1) Calculations performed without D3 correction

Not applying the D3 correction in the calculations is an oversight, which we will remember in the future. However, we checked that in the case of the molecules we tested, it had practically no effect on the values of the determined energies.

molecule	Electronic state	E[eV] without D3	E[eV] with D3	δE [eV]
1A	S_1	2.771 f=0.2906	2.763 f=0.2847	-0.008
	T_1	1.913	1.909	-0.005
	T_2	2.345	2.311	-0.034
	T_3	2.578	2.558	-0.020
	T_4	2.755	2.747	-0.008
	T_5	3.017	3.010	-0.007
	S_1 (opt.S_1)	2.394 f=0.4150	2.388 f=0.4096	-0.006
	E(0,0)	2.587	2.581	-0.006
7A	S_1	2.513 f=0.1077	2.508 f=0.1061	-0.005
	T_1	1.693	1.689	-0.004
	T_2	2.130	2.126	-0.004
	T_3	2.288	2.287	-0.002
	T_4	2.394	2.390	-0.004
	T_5	2.588	2.587	-0.001
	S_1 (opt.S_1)	2.104 f=0.1796	2.095 f=0.1763	-0.009
	E(0,0)	2.326	2.320	-0.006
1F	S_1	2.368 f=0.0824	2.366 f=0.0812	-0.002
	T_1	1.630	1.628	-0.003
	T_2	2.271	2.270	-0.002
	T_3	2.541	2.539	-0.002
	T_4	2.633	2.631	-0.002
	T_5	2.837	2.836	-0.001
	S_1 (opt.S_1)	1.723 f=0.0760	1.722 f=0.0753	-0.002
	E(0,0)	2.054	2.052	-0.002
8F	S_1	2.196 f=0.0484	2.192 f=0.0480	-0.004
	T_1	1.472	1.468	-0.004
	T_2	2.099	2.095	-0.004
	T_3	2.252	2.249	-0.003
	T_4	2.312	2.311	-0.002
	T_5	2.464	2.462	-0.002
	S_1 (opt.S_1)	1.590 f=0.0439	1.582 f=0.0432	-0.008
	E(0,0)	1.897	1.891	-0.006

Table S0. Comparison of the energies of the electronic states of four molecules optimized in Sthe ground and excited states without and with the D3 correction.

^{1}H NMR spectra of compounds with condensed aromatic rings contain fine line splittings due to long range *J*(H,H) couplings over four and five bonds. In addition to ^4J(H,H-*meta*) and ^5J(H,H-*para)* between protons on the aromatic ring (blue arrows in Fig.2) two types of characteristic small coupling between protons from neighboring rings can be observed (red arrows in Fig.2). While the ^4J(H,H-*meta*) are typically 1 - 2 Hz, all other shown couplings are smaller and about the same size (< 1Hz). This can lead to different types of multiplets with fine splittings about 0.5 Hz. Long range couplings providing a quartet of proton at 8.22 ppm in compound 2F are shown in Figure 3B. The observation of fine splitting of multiplets requires a good magnetic field homogenity and usually also use of wighting function for a line narrowing of signals (see Figure 3).

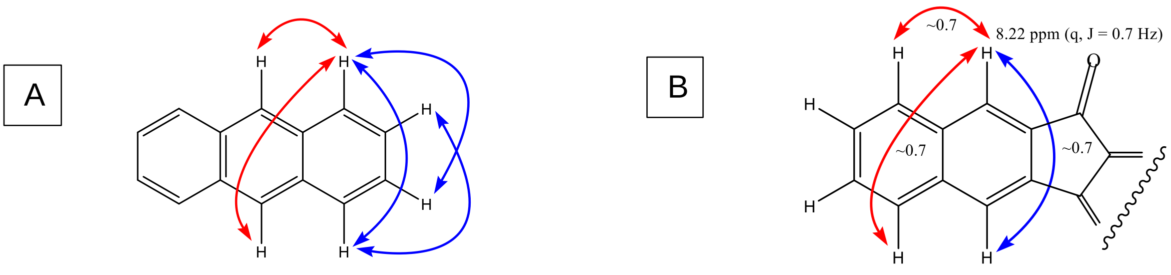

Figure S2 Characteristic types of the long range couplings *J*(H,H) in condensed aromatic ring systems (A); structural fragment of comppund **2F** and long range couplings responsible for the observation of proton at 8.22 ppm as quartet (B).

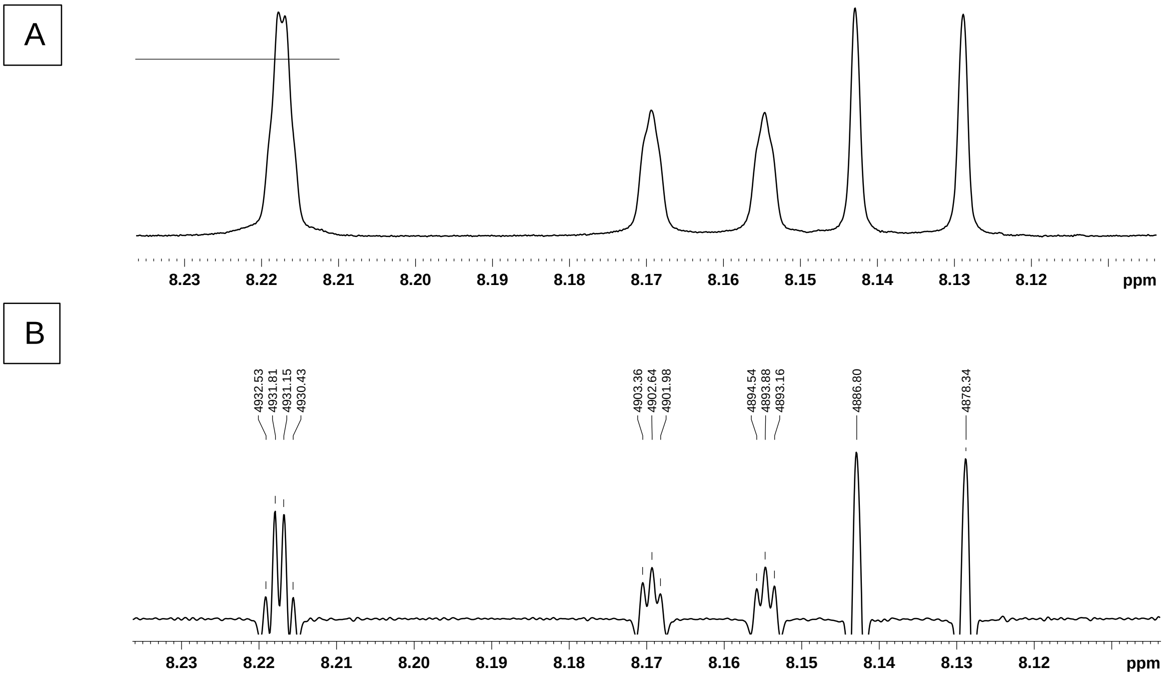

Figure S3 Part of ^{1}H NMR spectrum of compound **2F** after simple Fourier transformation of FID (A) and the same part of spectrum after using Gaussian apodization of FID (lb -1.5; gb 0.3) resulting in line narrowing and observation of fine splitting iof signals (B).

Series of synthesized helicenes: aceno[n]helicenes 1A-8A and fluoreno[n]helicenones 1F-8F

aceno[6]helicenes

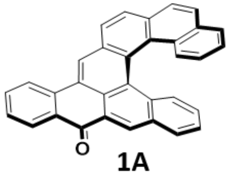
1A

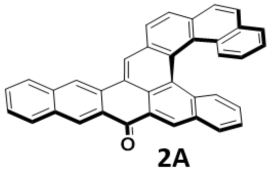
2A

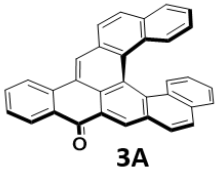
3A

aceno[7]helicenes

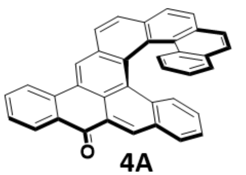
4A

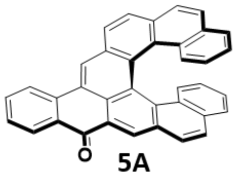
5A

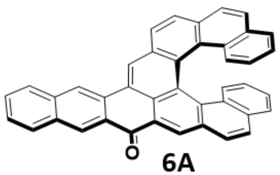
6A

aceno[8]helicene

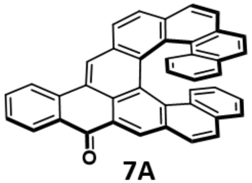
7A

aceno[9]helicene

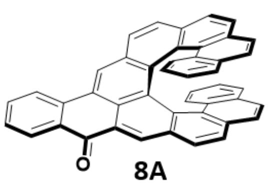
8A

fluoreno[6]helicenes

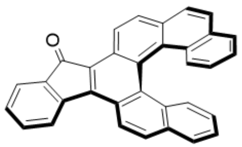
1F

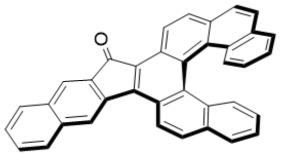
2F

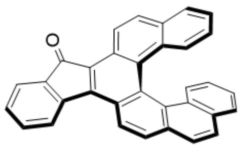
3F

fluoreno[7]helicenes

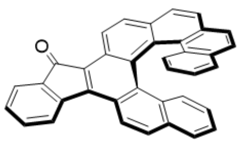
4F

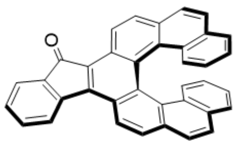
5F

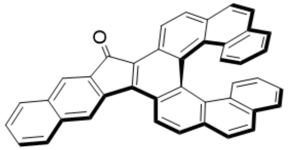
6F

fluoreno[8]helicene

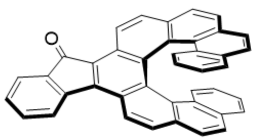
7F

fluoreno[9]helicene

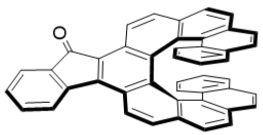
8F

Synthesis of compounds 1A and 1F

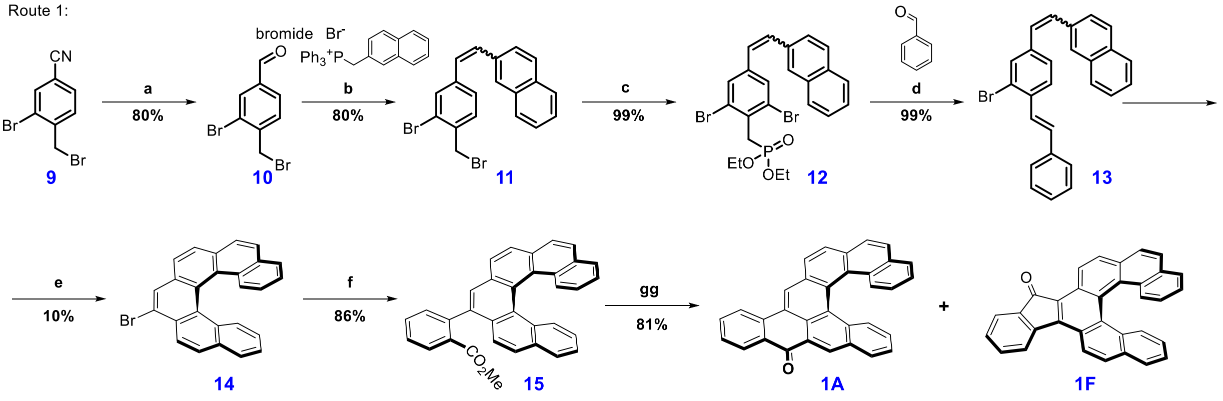

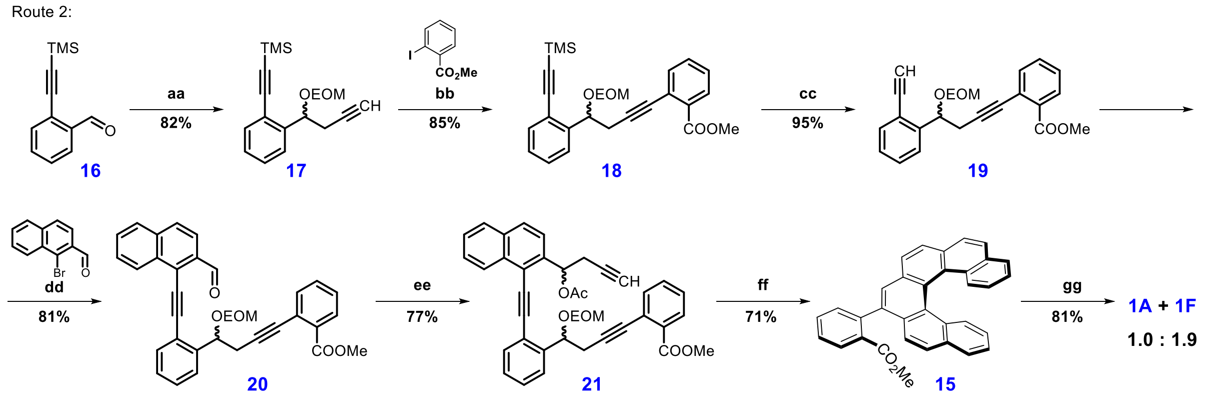

(a) Compound 9 (1.0 equiv.), DIBAL (2.5 equiv.), PhMe, -10-0°C, 6 h;

(b) Compound 10 (1.0 equiv.), (naphthalen-2-ylmethyl)triphenylphosphonium bromide (1.05 equiv.), NaH (1.1 equiv.), THF, rt, 4 h;

(c) compound 11 (1.0 equiv.), $P(OEt)_3$ (1.05 equiv.), 120°C, 16h;

(d) compound 12 (1.0 equiv.), benzaldehyde (1.05 equiv.), NaH (1.1equiv.), PhMe, 120°C, 10 min;

(e) stilbene 13 (1.0 equiv.), I_2 (250mg/1L of the solvent.) cyclohexane, hν, rt, 40 h.;

(f) helicene 14 (1.0 equiv.), 2-methoxycarbonylphenylboronic acid (1.5 equiv.), Pd_2dba_3 (2 mol%), Xphos (4mol%) and $K_3PO_4.H_2O$ (6 equiv.), PhMe, 90 °C, 12h;

(aa) Compound 16 (1.0 equiv.), Zn (2.0 equiv.), propargyl bromide (2.0 equiv.), THF, rt, 25 min, then DMAP (cat.), *N*,*N*-diisopropylethylamine (3.0 equiv.), chloromethyl ethyl ether (2.0 equiv.), DCM, rt, 16h.;

(bb) methyl 2-iodobenzoate (1.0 equiv.), diyne 17 (1.1 equiv.), $Pd(PPh_3)Cl_2$ (2 mol%), CuI (4 mol%), *i*-Pr_2NH:PhMe (3:1), 45 °C, 2 h;

(cc) K_2CO_3 (1.5 equiv.), MeOH, rt, 10 min;

(dd) 1-bromo-2-naphthaldehyde (1.0 equiv.), diyne 19 (1.1 equiv.) $Pd(PPh_3)Cl_2$ (2 mol%), CuI (4 mol%), THF:Et_3N (2:1), 50 °C, 3 h;

(ee) Zn (2.0 equiv.), propargyl bromide (2.0 equiv.), compound 20 (1.0 equiv.), THF, rt, 15 min, then Et_3N (3.0 equiv.), Ac_2O (3.0 equiv.), THF, rt, 2 h;

(ff) triyne 21 (1.0 equiv.), CpCo(CO)(fum) (0.5 equiv.), PhCl, 170 °C, 20 min, then *p*-TsOH.H_2O (5.0 equiv.), 95 °C, 1h;

(gg) ester 15 (1.0 equiv.), KOH (10.0 equiv.), THF:MeOH (1:1), 65 °C, 4 h, then $MeSO_3H$, 80°C, 1h.

<u>3-Bromo-4-(bromomethyl)benzaldehyde</u> 10

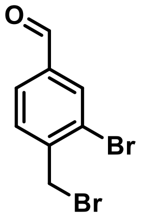

In a dry flask under inert atmosphere, 3-Bromo-4-(bromomethyl)benzonitrile **9** (12g, 43.8mmol, 1 eq.) was dissolved in dry Toluene (160 mL) and cooled down to 0°C. DIBAL-H (60 mL, 25%wt. in Toluene, 2.5 equiv.) was added dropwise and the temperature was kept at 0°C for 6h while stirring. The reaction mixture was quenched with carful addition of water (100mL). The product was extracted with DCM (3x100mL) and the combined organic phases were dried and concentrated under vacuum. The product was then purified by silica gel chromatography (petroleum ether-dichloromethane 98:2). The product **10** was isolated as a white solid (7.9g, 65%). ^{1}H NMR spectrum is in agreement with the published data.[10]

<u>(*E*)-2-(3-Bromo-4-(bromomethyl)styryl)naphthalene</u> 11

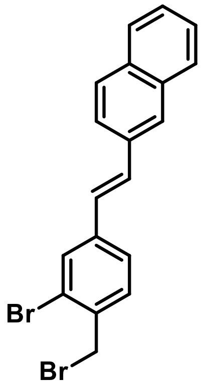

3-Bromo-4(bromomethyl)benzaldehyde **10** (4.5 g; 16.2 mmol) and (naphthalen-2-ylmethyl)triphenylphosphonium bromide (8.2 g; 17.0 mmol, 1.05 equiv.) were charged in dry round bottomed flask under inert atmosphere and dissolved in dry THF (150 mL). The reaction mixture was cooled down to 0°C and then sodium hydride (4.5 g; 17.8 mmol 60% in oil) was added portionwise. The reaction is complete when the color of the reaction turned orange (cca 4h). The mixture was kept at 0°C and quenched with water (150mL). After extraction with DCM (3x100mL), the organic portions were combined and dried over $MgSO_4$. Solvents were removed *in vacuo*, and the residue was chromatographed on silica gel (cyclohexane) to afford the desired product **11** as a mixture of two isomers that can be converted to E-isomer by exposure to the light, white solid (5.2g, 80 %).

^{1}H NMR (400 MHz CD_2Cl_2): 4.66 (s, 2H), 7.18 (d, *J*= 16.3, 1H), 7.34 (d, *J*= 16.3, 1H), 7.46-7.53 (m, 4H), 7.75 (dd, *J*= 8.6, 1.5, 1H), 7.81-7.90 (m, 5H).

^{13}C NMR (101 MHz, CD_2Cl_2): 33.7, 123.3, 124.9, 126.0, 126.3, 126.5, 126.6, 127.3, 127.7, 128.1, 128.5, 130.96, 130.99, 131.5, 133.3, 133.7, 134.2, 136.0, 139.8.

MALDI MS: 400 ([M]$^{+}$).

HR MS-MALDI: calc for $C_{19}H_{14}{}^{79}Br_2$ 399.9457; found 399.9460.

[10] G. Hao, H. Li, F. Yang, D. Dong, Z. Li, Y. Ding, W. Pan, E. Wang, R. Liu, H. Zhou *Bioorganic & Medicinal Chemistry* 29 (2021) 115871.

<u>**Diethyl (*E*)-(2-bromo-4-(2-(naphthalen-2-yl)vinyl)benzyl)phosphonate**</u> **12**

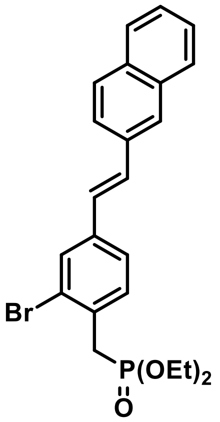

Previous compound **11** (5.2g, 13 mmol) and triethylphosphite (2.24 mL, 13.1 mmol, 1.1 equiv.) were added in well dry round bottom flask and stirred overnight at 120°C under inert atmosphere. The traces of triethylphosphite left were removed *in vacuo* by heating the reaction mixture at 70°C for 4 hours under vacuum. The product **12** as a mixture of two isomers was obtained as a yellowish solid (5.9 g, 99 %).

^{1}H NMR (400 MHz CD_2Cl_2): 1.27 (t, *J* = 6H), 3.36 (s, 1H), 3.41 (s, 1H), 4.01 – 4.08 (m, 4H) 7.17 (d, *J*= 16.3, 1H), 7.30 (d, *J*= 16.3, 1H), 7.44 – 7.51 (m, 4H), 7.75 (dd, *J* = 8.7, 1.8, 1H), 7.80 – 7.88 (m, 5H).

^{13}C NMR (101 MHz, CD_2Cl_2): 16.8 (d, *J* = 6.0, 2C), 33.8 (d, *J* = 138.3), 62.8 (d, *J* = 6.7, 2C), 123.9, 125.7 (d, *J* = 9.3), 126.0 (d, *J* = 3.6), 126.7, 127.0, 127.4 (d, *J* = 2.0), 127.5, 128.2, 128.6, 128.9, 130.6 (d, *J* = 1.5), 131.2 (d, *J* = 3.1), 131.8 (d, *J* = 9.3), 132.3 (d, *J* = 5.2), 133.8, 134.2, 134.9, 138.6 (d, *J* = 4.0).

ESI MS: 459 ($[M+H]^+$).

HR ESI MS: calc for $C_{23}H_{25}{}^{79}BrO_3P$ 459.0719; found 459.0714.

<u>**2-((*E*)-3-Bromo-4-((*E*)-styryl)styryl)naphthalene**</u> **13**

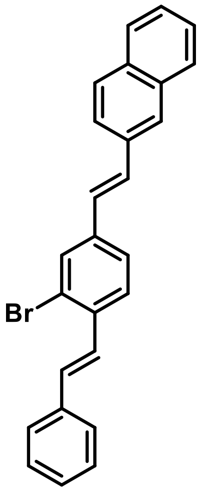

In a well dry round bottom flask were solved previous phosphonate **12** (5.9 g, 13mmol) and benzaldehyde (1.6 g, 1.05 equiv.) in dry toluene (100 mL). To this mixture was added NaH (60%, dispersion in mineral oil 3.9 g, 1.1 equiv.) and the mixture was heated at 120°C for 20 min. under inert atmosphere. The reaction mixture was cooled down to room temperature and then slowly poured into a mixture of ice-water- 37 % HCl (100 g -100 ml- 100 ml). The organic phase was separated and the water phase was extracted with DCM (3 x 100 mL). The organic portions were combined and all the volatiles were removed *in vacuo* and the residue was chromatographed on silica gel (cyclohexane) to give the pure product **13** as a yellow solid (5.2g 99%).

^{1}H NMR (400 MHz, CD_2Cl_2): 7.13 (d, *J* = 16.3, 1H), 7.19 (d, *J* = 16.3, 1H), 7.29 – 7.35 (m, 2H), 7.38-7.42 (m, 2H) 7.45 - 7.60 (m, 6H), 7.73 (d, *J* = 8.2, 1H), 7.76 (dd, *J* = 8.6, 1.8, 1H), 7.82-7.89 (m, 5H).

^{13}C NMR (101 MHz, CD_2Cl_2): 123.9, 125.0, 126.2, 126.7, 127.0, 127.1, 127.4 (3C), 127.5, 127.6, 128.2, 128.6, 128.7, 128.9, 129.30 (2C), 130.5, 131.4, 131.7, 133.8, 134.2, 134.9, 136.5, 137.6, 138.9.

MALDI MS: 410 ([M]$^+$).

HR MS MALDI: calc for $C_{26}H_{19}{}^{79}Br$ 410.0665; found 410.0670.

__7-Bromo[6]helicene__ **14**

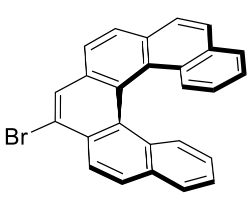

The previous stilbene **13** (2.2 g, 4.8 mmol) was dissolved in cyclohexane (1900 mL). Iodine (0.55 g, 2.16 mmol) was added to the mixture and the reaction mixture was irradiated with medium-pressure mercury (150 W) lamp for 40 h open to air. The whole reaction mixture was evaporated, and the residue was chromatographed on silica gel (cyclohexane-dichloromethane 40:1) to afford the desired product **14** (220 mg, 10%) as a yellowish solid.

^{1}H NMR (400 MHz, CD_2Cl_2): 6.64 - 6.70 (m, 2H), 7.20 – 7.28 (m, 2H), 7.43 (d, *J* = 8.4, 1H), 7.55 (d, *J* = 8.5, 1H), 7.83 - 7.89 (m, 2H), 7.93 - 7.99 (m, 3H), 8.03 – 8.05 (m, 2H), 8.36 (s, 1H), 8.42 (d, *J* = 8.9, 1 H).

^{13}C NMR (101 MHz, CD_2Cl_2): 121.4, 123.5, 124.6, 124.8, 125.1, 125.7, 125.8, 126.1, 126.2, 127.4, 127.6, 127.7, 127.9, 128.0, 128.2, 128.3, 128.9, 129.3, 129.56, 129.62, 129.8, 130.2, 131.4, 131.8, 132.1, 133.2.

MALDI MS: 406 ([M]$^+$).

HR MS MALDI: calc for $C_{26}H_{15}{}^{79}Br$ 406.0352; found 406.0354.

__Methyl 2-hexahelicen-7-ylbenzoate__ **15**

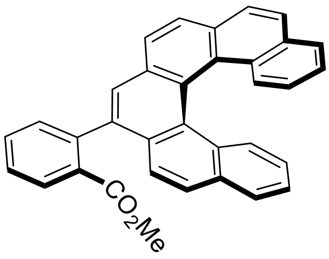

In a dry Schlenk flask was dissolved 7-bromo[6]helicene **14** (20 mg, 0.05 mmol), 2-methoxycarbonylphenylboronic acid (13 mg, 0.074 mmol, 1.5 equiv.), Pd_2dba_3 (1 mg, 1.1 µmol, 2 mol%), Xphos (1.1 mg, 2.3 µmol, 4 mol%) and $K_3PO_4.H_2O$ (70 mg, 0.3 mmol, 6 equiv.) in dry toluene (6 mL). The reaction mixture was thoroughly degassed and then heated at 90 °C overnight. The solvent was evaporated *in vacuo*

and the residue was purified by column chromatography on silca gel (cyclohexane-dichloromethane 10:1). The product **15** was obtained as a yellow solid (20 mg, 86%). The ^{1}H NMR spectrum shows a mixture of two atropodiastereomers (1:2).

^{1}H NMR (400 MHz, CD_2Cl_2, major atropodiastereomer): 3.36 (s, 3H), 6.65 – 6.74 (m, 2H), 7.20 – 7.27 (m, 2H), 7.55 – 7.65 (m, 6H), 7.78 – 7.88 (m, 3H), 7.86 (s, 1H), 7.94 – 7.98 (m, 2H), 8.00 – 8.04 (m, 2H), 8.17 (m, 1H).

^{1}H NMR (400 MHz, CD_2Cl_2, minor atropodiastereomer): 3.34 (s, 3H), 6.65 – 6.74 (m, 2H), 7.20 – 7.27 (m, 2H), 7.55 – 7.65 (m, 6H), 7.78 – 7.88 (m, 3H), 7.86 (s, 1H), 7.94 – 7.98 (m, 2H), 8.00 – 8.04 (m, 2H), 8.12 (m, 1H).

Other characterization data are attached for identical compound prepared by Route 2, shown below.

({2-[1-(Ethoxymethoxy)but-3-yn-1-yl]phenyl}ethynyl)(trimethyl)silane 17

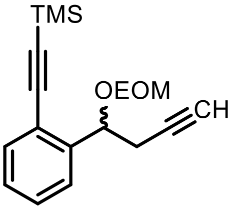

A Schlenk flask was charged with zinc powder (1.29 g, 19.8 mmol, 2.0 equiv.) and flushed with nitrogen. The freshly distilled tetrahydrofuran (8 mL) was added, the suspension was put to a water bath at room temperature and vigorously stirred. Then propargyl bromide (80 wt. % in toluene, 2.20 mL, 19.8 mmol, 2.0 equiv.) was slowly added within 10 min and the mixture was then stirred at room temperature for 10 min before it was transferred by a syringe to the second Schlenk flask with a suspension of aldehyde **16** (2.00 g, 9.88 mmol) in tetrahydrofuran (20 mL). The reaction mixture was stirred at room temperature for 5 min, then quenched with a saturated solution of ammonium chloride (20 mL) and extracted with ethyl acetate (2 x 20 mL). The combined organic layers were dried over anhydrous $MgSO_4$ and evaporated *in vacuo*. The residue was dissolved in dichloromethane (20 mL), DMAP (5 mg, cat.), *N*,*N*-diisopropylethylamine (5.16 mL, 29.6 mmol, 3.0 equiv.), and chloromethyl ethyl ether (1.84 mL, 19.8 mmol, 2.0 equiv.) were added successively. The solution was stirred at room temperature overnight and then quenched with brine (20 mL). The layers were separated and water layer was extracted with dichloromethane (10 mL). The organic layers were dried over anhydrous $MgSO_4$, solvents were removed *in vacuo*, and the residue was chromatographed on silica gel (hexane-ethyl acetate 40:1) to afford the desired product **17** (2.43 g, 82%) as a colorless oil.

^{1}H NMR (400 MHz, $CDCl_3$): 0.27 (s, 9H), 1.18 (t, *J* = 7.1, 3H), 1.98 (t, *J* = 2.6, 1H), 2.61 (ddd, *J* = 16.9, 7.7, 2.6, 1H), 2.75 (ddd, *J* = 16.9, 4.2, 2.7, 1H), 3.53 (dq, *J* = 9.4, 7.1, 1H), 3.85 (dq, *J* = 9.4, 7.1, 1H), 4.62 (d, *J* = 6.9, 1H), 4.76 (d, *J* = 6.9, 1H), 5.34 (dd, *J* = 7.7, 4.1, 1H), 7.22 (td, *J* = 7.5, 1.4, 1H), 7.34 (tdd, *J* = 7.9, 1.4, 0.5, 1H), 7.43 (ddd, *J* = 7.7, 1.4, 0.6, 1H), 7.50 (ddt, *J* = 7.9, 1.2, 0.6, 1H).

^{13}C NMR (101 MHz, $CDCl_3$): 0.0 (3C), 15.2, 26.8, 63.6, 69.7, 73.9, 81.3, 93.5, 100.5, 102.4, 121.6, 125.8, 127.5, 128.8, 132.3, 143.3.

IR ($CHCl_3$): 3309 m, 3070 w, 2977 w, 2961 w, 2898 w, 2887 w, sh, 2156 w, 2122 vw, 1599 vw, 1570 vw, 1478 w, 1448 w, 1410 w, 1262 w, 1251 m, 1231 w, 1160 w, sh, 1148 w, 1112 m, 1100 m, 1044 w, sh, 1018 s, 867 vs, 846 vs, 700 w, 648 m, 632 w, sh cm^{-1}.

EI MS: 300 ($M^{+\bullet}$, 4), 261(31), 232 (35), 217 (40), 209 (17), 187 (23), 143 (97), 115 (83), 73 (100).

HR EI MS: calcd for $C_{18}H_{24}O_2Si$ 300.1540, found 300.1543.

Methyl 2-[4-(ethoxymethoxy)-4-{2-[(trimethylsilyl)ethynyl]phenyl}but-1-yn-1-yl]benzoate 18

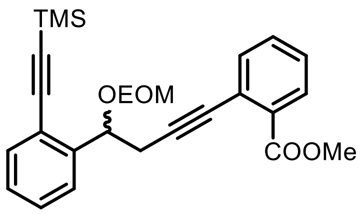

A Schlenk flask was charged with methyl 2-iodobenzoate (1.35 g, 5.15 mmol), $Pd(PPh_3)_2Cl_2$ (72.3 mg, 0.10 mmol, 2 mol%), CuI (39.2 mg, 0.21 mmol, 4 mol%), flushed with nitrogen, and the degassed *N*,*N*-diisopropylamine (45 mL) was added. The mixture was heated to 45 °C before a solution of alkyne **17** (1.70 g, 5.66 mmol, 1.1 equiv.) in degassed toluene (15 mL) was slowly added. The reaction mixture was stirred at the same temperature for 2 h. The solvents were evaporated under the reduced pressure. The residue was chromatographed on silica gel (hexane-ethyl acetate 20:1) to afford the desired product **18** (1.90 g, 85%) as a colorless oil.

^{1}H NMR (400 MHz, $CDCl_3$): 0.25 (s, 9H), 1.13 (t, *J* = 7.1, 3H), 2.95 (dd, *J* = 17.0, 7.2, 1H), 3.05 (dd, *J* = 17.0, 4.8, 1H), 3.53 (dq, *J* = 9.4, 7.0, 1H), 3.81 (dq, *J* = 9.4, 7.1, 1H), 3.87 (s, 3H), 4.67 (d, *J* = 6.8, 1H), 4.81 (d, *J* = 6.9, 1H), 5.43 (dd, *J* = 7.1, 4.8, 1H), 7.23 (td, *J* = 7.5, 1.3, 1H), 7.30 (ddd, *J* = 7.8, 7.3, 1.5, 1H), 7.35 (tdd, *J* = 7.9, 1.5, 0.5, 1H), 7.40 (td, *J* = 7.5, 1.5, 1H), 7.45 (ddd, *J* = 7.6, 1.4, 0.5, 1H), 7.46 (ddd, *J* = 7.8, 1.5, 0.6, 1H), 7.58 (ddt, *J* = 7.8, 1.2, 0.6, 1H), 7.87 (ddd, *J* = 7.9, 1.4, 0.6, 1H).

^{13}C NMR (101 MHz, $CDCl_3$): 0.0 (3C), 15.2, 28.1, 52.2, 63.6, 74.2, 80.6, 92.5, 93.7, 100.2, 102.7, 121.8, 124.4, 126.1, 127.4, 127.4, 128.9, 130.2, 131.5, 132.1, 132.3, 134.5, 143.5, 166.9.

IR ($CHCl_3$): 3071 w, 2977 m, 2955 m, 2898 w, 2887 w, sh, 2845 w, 2235 w, 2156 w, 1729 s, 1719 s, sh, 1597 w, 1568 w, 1486 m, 1448 m, 1435 m, 1415 w, 1298 s, 1279 s, 1252 vs, 1233 m, 1163 w, 1149 w, sh, 1131 m, 1112 m, 1099 m, sh, 1043 s, 1018 s, 965 w, 867 vs, 845 vs, 701 w, 646 w cm^{-1}.

EI MS: 434 ($M^{+\bullet}$, 2), 358(33), 343 (27), 261 (100), 233 (99), 217 (100), 188 (58), 173 (90), 143 (100), 115 (99), 73 (97).

HR EI MS: calcd for $C_{26}H_{30}O_4Si$ 434.1908, found 434.1896.

<u>**Methyl 2-[4-(ethoxymethoxy)-4-(2-ethynylphenyl)but-1-yn-1-yl]benzoate**</u> **19**

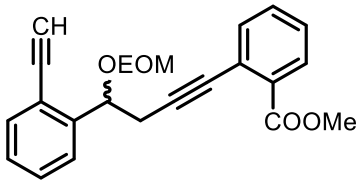

Diyne **18** (1.85 g, 4.26 mmol) was dissolved in methanol (40 mL) and potassium carbonate (883 mg, 6.39 mmol, 1.5 equiv.) was added. The mixture was stirred at room temperature for 10 min. The reaction was quenched with a saturated ammonium chloride solution (100 mL), extracted with dichloromethane (2 x 80 mL), and the combined organic layers were dried over anhydrous magnesium sulfate. The solvents were evaporated under the reduced pressure and the residue was purified by flash chromatography on silica gel (hexane-ethyl acetate 10:1) to afford **19** (1.47 g, 95%) as a colorless oil.

^{1}H NMR (400 MHz, $CDCl_3$): 1.11 (t, *J* = 7.1, 3H), 2.97 (dd, *J* = 17.0, 7.0, 1H), 3.04 (dd, *J* = 17.0, 5.1, 1H), 3.35 (s, 1H), 3.51 (dq, *J* = 9.5, 7.1, 1H), 3.77 (dq, *J* = 9.4, 7.1, 1H), 3.87 (s, 3H), 4.67 (d, *J* = 6.9, 1H), 4.81 (d, *J* = 6.9, 1H), 5.41 (dd, *J* = 7.1, 5.1, 1H), 7.25 (td, *J* = 7.6, 1.4, 1H), 7.30 (td, *J* = 7.6, 1.5, 1H), 7.36 – 7.42 (m, 2H), 7.45 (dd, *J* = 7.7, 1.2, 1H), 7.49 (dd, *J* = 7.7, 1.3, 1H), 7.58 – 7.61 (m, 1H), 7.86 (ddd, *J* = 7.8, 1.4, 0.6, 1H).

^{13}C NMR (101 MHz, $CDCl_3$): 15.1, 28.3, 52.2, 63.6, 74.3, 80.7, 81.3, 82.6, 92.2, 93.8, 120.7, 124.3, 126.4, 127.4, 127.6, 129.1, 130.2, 131.5, 132.1, 132.9, 134.4, 143.6, 167.0.

IR ($CHCl_3$): 3304 m, 3071 w, 2979 m, 2953 w, 2886 m, 2845 w, 2234 w, 2106 w, 1727 vs, 1598 w, 1568 w, 1468 w, 1448 m, 1435 m, 1297 s, 1279 s, 1256 s, 1232 m, 1164 m, 1149 w, 1131 s, 1111 s, 1099 s, 1044 vs, 1018 vs, 965 w, 701 w, 658 m, 620 m cm^{-1}.

EI MS: 362 ($M^{+\bullet}$, 2), 333 (15), 271 (63), 226 (43), 188 (59), 173 (91), 161 (80), 117 (100), 83 (39), 59 (61).

HR EI MS: calcd for $C_{23}H_{22}O_4$ 362.1513, found 362.1496.

Methyl 2-[4-(ethoxymethoxy)-4-{2-[(2-formylnaphthalen-1-yl)ethynyl]phenyl}but-1-yn-1-yl]benzoate **20**

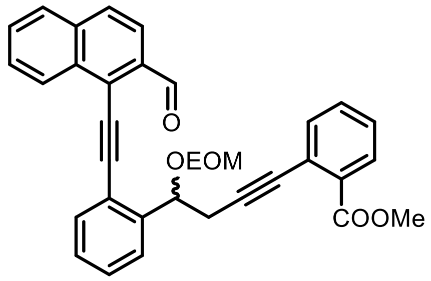

A Schlenk flask was charged with 1-bromo-2-naphthaldehyde (294 mg, 1.25 mmol), bis(triphenylphosphine)palladium chloride (17.5 mg, 0.03 mmol, 2 mol%), copper(I) iodide (9.52 mg, 0.05 mmol, 4 mol%) and flushed with argon. The degassed tetrahydrofuran (10 mL) and degassed triethylamine (10 mL) were added and the mixture was heated to 50 °C. Then diyne **19** (500 mg, 1.38 mmol, 1.1 equiv.) in degassed tetrahydrofuran (10 mL) was slowly added and the reaction was stirred at 50 °C for 3 hours. The solvents were evaporated under the reduced pressure. The residue was chromatographed on silica gel (hexane-ethyl acetate 20:1 to 10:1) to afford the desired product **20** (577 mg, 81%).

^{1}H NMR (400 MHz, $CDCl_3$): 1.11 (t, *J* = 7.1, 3H), 3.13 (d, *J* = 6.5, 2H), 3.55 (dq, *J* = 9.5, 7.0, 1H), 3.74 (s, 3H), 3.83 (dq, *J* = 9.5, 7.1, 1H), 4.73 (d, *J* = 7.0, 1H), 4.86 (d, *J* = 7.0, 1H), 5.63 (t, *J* = 6.4, 1H), 7.24 – 7.29 (m, 1H), 7.32 (td, *J* = 7.5, 1.6, 1H), 7.391 (td, *J* = 7.5, 1.4, 1H), 7.392 (ddd, *J* = 7.7, 1.5, 0.5, 1H), 7.49 (tdd, *J* = 7.8, 1.4, 0.5, 1H), 7.58 – 7.66 (m, 2H), 7.69 (ddt, *J* = 7.8, 1.3, 0.5, 1H), 7.73 (ddd, *J* = 7.6, 1.4, 0.5, 1H), 7.83 (ddd, *J* = 7.7, 1.6, 0.6, 1H), 7.86 – 7.90 (m, 2H), 7.99 (d, *J* = 8.6, 1H), 8.65 – 8.68 (m, 1H), 10.92 (d, *J* = 0.9, 1H).

^{13}C NMR (101 MHz, $CDCl_3$): 15.1, 28.8, 52.1, 63.8, 74.7, 81.2, 88.1, 91.8, 93.6, 100.0, 121.3, 122.3, 124.1, 127.0, 127.3, 127.49, 127.50, 128.0, 128.1, 128.6, 129.2, 129.5, 129.8, 130.2, 131.5, 132.0, 132.9, 133.3, 134.47, 134.53, 135.9, 143.2, 166.7, 192.1.

IR ($CHCl_3$): 3063 w, 2979 w, 2953 w, 2886 w, 2847 w, 2742 w, 2236 w, 2204 w, 1727 s, 1694 vs, 1679 s, 1618 w, 1592 m, 1568 w, 1507 w, 1486 m, 1458 m, 1448 m, 1434 m, 1401 w, sh, 1385 m, 1333 m, 1297 s, 1278 s, 1256 s, 1163 w, 1151 m, sh, 1131 m, 1121 m, 1095 m, 1044 s, 1029 s, sh, 1018 s, 963 w, 871 w, 823 m, 701 w, 657 w, 570 w, 435 w cm^{-1}.

ESI MS: 539 ($[M+Na]^+$).

HR ESI MS: calcd for $C_{34}H_{28}O_5Na$ 539.1829, found 539.1827.

Methyl 2-{4-[2-({2-[1-(acetyloxy)but-3-yn-1-yl]naphthalen-1-yl}ethynyl)phenyl]-4-(ethoxymethoxy)but-1-yn-1-yl}benzoate **21**

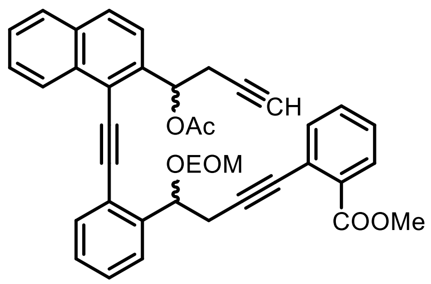

A Schlenk flask was filled with zinc powder (139 mg, 2.12 mmol, 2 equiv.) and flushed with argon. Freshly distilled tetrahydrofuran (4 mL) was added and vigorously stirred. Then propargyl bromide (80 wt. % in toluene, 236 µL, 2.12 mmol, 2 equiv.) was added and stirred for 10 min, before it was transferred by a syringe to the second Schlenk flask, which contained aldehyde **20** (546 mg, 1.06 mmol) and tetrahydrofuran (20 mL). The reaction mixture was stirred for 5 min at room temperature, before triethylamine (443 µL, 3.18 mmol, 3 equiv.) and acetic anhydride (303 µL, 3.18 mmol, 3 equiv.) were added and the solution was stirred for 2 h at room temperature. The solvents were evaporated under the reduced pressure and the residue was purified by flash chromatography on silica gel (hexane-ethyl acetate 10:1) to obtain acetate **21** (488 mg, 77%) as a mixture of diastereomers in a ratio about 1:1.

^{1}H NMR (400 MHz, $CDCl_3$, mixture of diastereomers): 1.09 (t, *J* = 7.1, 3H), 1.10 (t, *J* = 7.1, 3H), 2.01 (t, *J* = 3.0, 1H), 2.02 (t, *J* = 3.0, 1H), 2.14 (s, 3H), 2.15 (s, 3H), 2.931 (ddd, *J* = 17.0, 6.6, 2.7, 1H), 2.933 (ddd, *J* = 16.9, 6.4, 2.6, 1H), 3.006 (dd, *J* = 17.1, 5.6, 1H), 3.012 (dd, *J* = 17.1, 5.6, 1H), 3.12 (dd, *J* = 17.0, 7.1, 2H), 3.21 (dd, *J* = 17.0, 5.2, 2H), 3.554 (dq, *J* = 9.8, 7.1, 1H), 3.561 (dq, *J* = 9.8, 7.1, 1H), 3.75 (s, 3H), 3.76 (s, 3H), 3.75 – 3.86 (m, 2H), 4.775 (d, *J* = 6.9, 1H), 4.781 (d, *J* = 6.9, 1H), 4.89 (d, *J* = 6.9, 1H), 4.91 (d, *J* = 6.9, 1H), 5.62 – 5.67 (m, 2H), 6.73 (t, *J* = 6.1, 2H), 7.24 – 7.57 (m, 14H), 7.636 (d, *J* = 8.6, 1H), 7.638 (d, *J* = 8.6, 1H), 7.69 (dt, *J* = 7.7, 1.4, 2H), 7.72 – 7.77 (m, 2H), 7.81 – 7.89 (m, 6H), 8.50 – 8.55 (m, 2H).

^{13}C NMR (101 MHz, $CDCl_3$, mixture of diastereomers): 15.1, 15.2, 21.2 (2C), 25.8, 25.9, 28.6, 28.7, 52.09, 52.10, 63.8 (2C), 71.11, 71.12, 72.2, 72.3, 74.89, 74.92, 79.50, 79.53, 81.0 (2C), 89.67, 89.72, 92.2, 92.3, 93.9, 94.0, 98.35, 98.36, 118.8, 118.9, 121.6, 121.7, 123.15, 123.21, 124.3 (2C), 126.7 (2C), 126.8 (3C), 126.9, 127.4 (2C), 127.5 (2C), 127.9 (2C), 128.2 (2C), 129.0, 129.1, 129.1 (2C), 130.2 (2C), 131.49, 131.51, 132.03, 132.04, 132.86, 132.87, 132.90, 133.0, 133.2 (2C), 134.50, 134.52, 139.5, 139.6, 142.8, 142.9, 166.9 (2C), 169.86, 169.88.

IR ($CHCl_3$): 3309 m, 3063 w, 2978 w, 2953 w, 2931 w, 2887 w, sh, 2844 w, sh, 2233 w, 2205 vw, 2124 vw, 1733 vs, 1597 w, 1568 w, 1509 w, 1486 m, 1448 m, 1435 m, 1372 m, 1297 m, 1278 m, 1254 s, sh, 1238 vs, 1164 w, 1149 w, 1131 m, 1112 m, 1094 m, 1044 s, 1022 s, 965 w, 867 w, 821 m, 701 w, 639 w, 609 w, 436 w cm^{-1}.

ESI MS: 621 ($[M+Na]^+$).

HR ESI MS: calculated for $C_{39}H_{34}O_6Na$ 621.2248, found 621.2245.

Methyl 2-hexahelicen-7-ylbenzoate 15

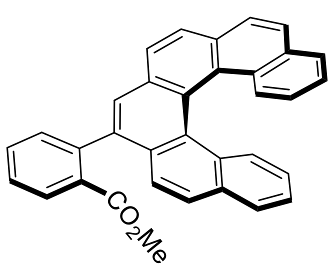

Triyne **21** (440 mg, 0.73 mmol) was loaded to a microwave vial and chlorobenzene (12 mL) was added. The solution was bubbled with nitrogen for 5 minutes before CpCo(CO)(fum) (109 mg, 0.37 mmol, 0.5 equiv, fum = dimethylfumarate) was added. The seal was closed and the reaction mixture was heated to 170 °C in the microwave reactor for 20 minutes. *p*-Toluenesulfonic acid monohydrate (694 mg, 3.65 mmol, 5 equiv.) was added and the reaction mixture was strirred for 1 h at 95 °C. The solvent was evaporated in vacuum. The residue was purified by flash chromatography on silica gel (hexane-ethyl acetate 20:1) to obtain helicene **15** (239 mg, 71%) as a mixture of two atropodiastereomers in a ratio 2:1.

^{1}H NMR (400 MHz, $CDCl_3$, major atropodiastereomer): 3.32 (s, 3H), 6.65 – 6.75 (m, 2H), 7.19 – 7.26 (m, 2H), 7.56 – 7.73 (m, 6H), 7.77 – 7.86 (m, 3H), 7.85 (s, 1H), 7.91 – 7.97 (m, 2H), 7.98 – 8.02 (m, 2H), 8.18 (ddd, *J* = 7.8, 1.5, 0.6, 1H).

^{13}C NMR (101 MHz, $CDCl_3$, major atropodiastereomer): 52.1, 123.6, 123.9, 124.90, 124.92, 125.7, 125.8, 126.3, 126.4, 126.9, 127.5, 127.6, 127.7, 127.7, 127.9, 127.95 (2C), 128.0, 128.11, 128.3, 129.9, 130.1, 130.2, 130.6, 131.3, 131.4, 131.7, 132.0, 132.1, 132.3, 132.4, 139.3, 141.4, 168.1.

^{1}H NMR (400 MHz, $CDCl_3$, minor atropodiastereomer): 3.25 (s, 3H), 6.65 – 6.75 (m, 2H), 7.19 – 7.26 (m, 2H), 7.56 – 7.73 (m, 6H), 7.77 – 7.86 (m, 3H), 7.85 (s, 1H), 7.91 – 7.97 (m, 2H), 7.98 – 8.02 (m, 2H), 8.12 (m, 1H).

^{13}C NMR (101 MHz, $CDCl_3$, minor atropodiastereomer): 51.9, 123.7, 123.8, 124.7, 124.7, 125.6, 125.7, 126.4, 126.9, 127.0, 127.4, 127.6, 127.7, 127.8, 127.90, 127.93 (2C), 127.95, 128.2, 128.4, 130.0, 130.1, 130.3, 130.4, 131.3, 131.5, 131.7 (2C), 132.0, 132.2, 132.5, 138.4, 141.2, 168.2.

IR ($CHCl_3$): 3050 w, 2953 w, 2854 w, 1717 vs, 1617 vw, 1599 w, 1572 vw, 1521 vw, 1503 w, 1460 vw, 1448 w, 1434 w, 1395 vw, 1367 w, 1296 s, 1281 m, sh, 1260 m, 1192 w, 1162 vw, 1129 m, 1117 w, 1084 m, 1049 w, 1042 w, 964 w, sh, 914 vw, 889 w, 883 w, 833 s, 808 w, 662 w, 648 vw, 618 w, 585 w, 529 w cm^{-1}.

ESI MS: 485 ($[M+Na]^+$).

HR ESI MS: calculated for $C_{34}H_{22}O_2Na$ 485.1512, found 485.1513.

<u>12*H*-Naphtho[3,2,1-*gh*]hexahelicen-12-one</u> 1A

<u>17*H*-Indeno[1,2-*i*]hexahelicen-17-one</u> 1F

Ester **15** (200 mg, 0.43 mmol) was dissolved in tetrahydrofuran (5 mL) and methanol (5 mL), and potassium hydroxide (240 mg, 4.30 mmol, 10 equiv.) was added. The mixture was heated to 65 °C and stirred for 4 h. Then the reaction was cooled to rt and diluted hydrochloric acid was added to reach pH 1. The mixture was extracted with diethyl ether (2x 20 mL), and the combined organic layers were dried over anhydrous magnesium sulfate. The solvents were evaporated under the reduced pressure to obtain crude carboxylic acid, which was directly used further. This acid was dissolved in methanesulfonic acid (10 mL) and the flask was put to the oil bath pre-heated to 80 °C. The stirring was continued for 1 h at 80 °C and then the reaction mixture was poured over crushed ice. The mixture was extracted with dichloromethane (2x 20 mL), the combined organic layers were washed with saturated sodium bicarbonate solution (10 mL), dried over anhydrous magnesium sulfate. The solvent was evaporated *in vacuo* and the residue was purified by flash chromatography on silica gel (hexane-dichloromethane 2:1 to 1:2) to afford **1A** (51.2 mg, 28%) as an orange solid and **1F** (97.3 mg, 53%) as a red solid.

<u>12*H*-Naphtho[3,2,1-*gh*]hexahelicen-12-one</u> 1A

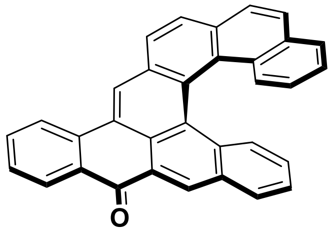

M.p.: 273 - 274 °C (CH_3CN).

Optical rotation: $[\alpha]^{20}_D$ +2147° (c 0.022, CH_2Cl_2); $[\alpha]^{20}_D$ -2165° (c 0.030, CH_2Cl_2).

^{1}H NMR (400 MHz, $CDCl_3$): 6.67 (ddd, *J* = 8.4, 6.9, 1.4, 1H), 6.82 (ddd, *J* = 8.5, 6.9, 1.4, 1H), 7.23 (ddd, *J* = 8.0, 6.8, 1.1, 1H), 7.32 (ddd, *J* = 8.0, 6.8, 1.1, 1H), 7.51 (d, *J* = 8.5, 1H), 7.60 (td, *J* = 8.1, 1.0, 1H), 7.66 (d, *J* = 8.6, 1H), 7.78 – 7.84 (m, 2H), 7.92 (d, *J* = 8.6, 1H), 7.94 (d, *J* = 8.7, 1H), 8.01 (d, *J* = 8.2, 1H), 8.07 (d, *J* = 8.1, 1H), 8.09 (bd, *J* = 8.8, 1H), 8.49 (d, *J* = 8.1, 1H), 8.58 (dd, *J* = 7.9, 1.5, 1H), 8.80 (s, 1H), 9.23 (s, 1H).

^{13}C NMR (101 MHz, $CDCl_3$): 124.0, 124.2, 125.1, 125.2, 125.4, 125.8, 126.1, 126.3, 126.4, 126.6, 127.4 (2C), 127.8 (2C), 128.06, 128.14, 128.3, 128.4, 128.5, 128.6, 128.8, 129.9, 130.2, 130.85, 130.93, 132.1 (2C), 132.2, 132.6, 132.9, 134.0, 136.8, 184.7.

IR ($CHCl_3$): 3051 w, 2956 w, 2928 w, 2856 w, 1653 vs, 1611 m, 1602 m, 1587 m, 1557 w, 1522 w, 1480 w, 1450 w, 1442 w, 1407 w, 1389 w, 1367 w, 1342 w, 1325 w, 1279 m, 1273 m, 1247 m, 1173 w, 1162 w, 1148 vw, 1082 vw, 1013 w, 934 w, 885 w, 862 w, 833 m, 692 w, 645 w, 623 w, 610 w, 582 w, 532 w, 451 vw cm^{-1}.

APCI MS: 431 ($[M+H]^+$).

HR APCI MS: calcd for $C_{33}H_{19}O$ 431.1430, found 431.1429.

<u>17*H*-Indeno[1,2-*i*]hexahelicen-17-one</u> 1F

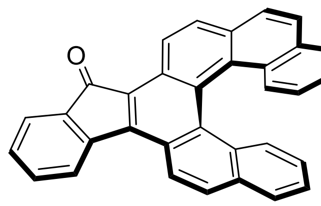

M.p.: 260 - 261 °C (CH_3CN).

^{1}H NMR (400 MHz, $CDCl_3$): 6.62 – 6.71 (m, 2H), 7.18 (t, *J* = 7.4, 1H), 7.24 (t, *J* = 7.3, 1H), 7.32 (t, *J* = 7.4, 1H), 7.38 (d, *J* = 8.6, 1H), 7.49 (d, *J* = 9.0, 1H), 7.53 (t, *J* = 7.7, 1H), 7.71 (d, *J* = 7.2, 1H), 7.77 (d, *J* = 7.1, 1H), 7.78 (d, *J* = 8.3, 1H), 7.84 – 7.91 (m, 2H), 7.97 (bd, *J* = 8.7, 1H), 8.01 (bd, *J* = 8.3, 1H), 8.06 – 8.13 (m, 1H), 8.51 – 8.59 (m, 1H), 9.30 (bd, *J* = 8.3, 1H).

^{13}C NMR (101 MHz, $CDCl_3$): 121.6, 122.4, 123.7, 124.0, 125.0, 125.5, 125.6, 125.8, 125.9, 126.3, 126.4, 127.2, 127.5, 127.8, 127.9, 128.4, 128.5, 128.98, 129.02, 129.1, 129.3, 129.6, 130.0, 130.2, 131.3, 132.2, 132.6, 134.4, 134.6, 135.9, 143.6, 144.1, 195.7.

IR ($CHCl_3$): 3052 w, 2956 m, 2927 w, 2855 w, 1700 vs, 1616 w, 1608 m, 1588 vw, 1570 vw, 1516 m, 1484 w, 1465 m, 1446 w, 1390 w, 1332 w, 1290 w, 1250 w, 1239 w, 1200 w, 1174 w, 1159 w, 1087 w, 1018 w, 983 w, 904 w, 844 m, 830 m, 806 w, 631 w, 622 m, 586 vw, 532 w, 463 vw cm^{-1}.

APCI MS: 431 ($[M+H]^+$).

HR APCI MS: calcd for $C_{33}H_{19}O$ 431.1430, found 431.1427.

Synthesis of compounds 2A and 2F

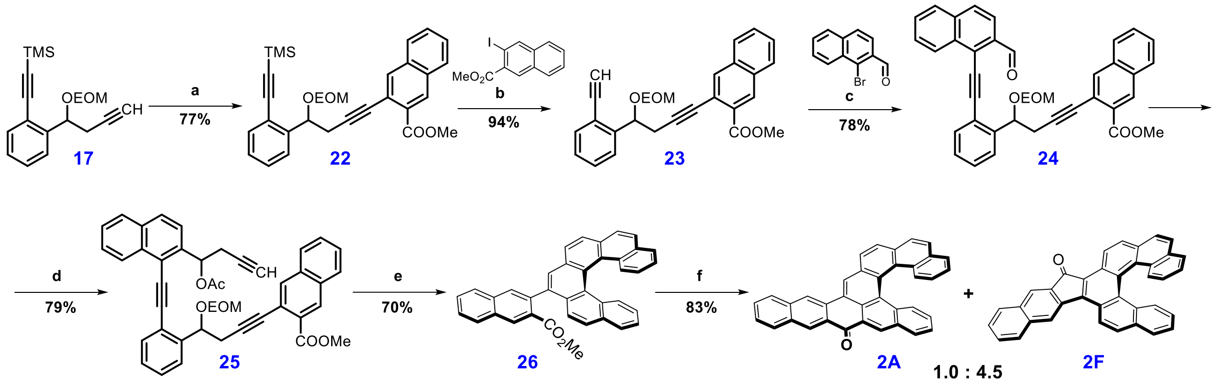

(a) Methyl 3-iodonaphthalene-2-carboxylate (1.0 equiv.), diyne 17 (1.1 equiv.), $Pd(PPh_3)Cl_2$ (2 mol%), CuI (4 mol%), *i*-Pr_2NH:PhMe (3:1), 45 °C, 3 h;

(b) compound 22 (1.0 equiv.), K_2CO_3 (1.5 equiv.), MeOH, rt, 15 min;

(c) 1-bromo-2-naphthaldehyde (1.0 equiv.), diyne 23 (1.1equiv.), $Pd(PPh_3)Cl_2$ (2 mol%), CuI (4 mol%), THF:Et_3N (2:1), 50 °C, 3 h;

(d) Zn (2.0 equiv.), propargyl bromide (2.0 equiv.), compound 24 (1.0 equiv.), THF, rt, 15 min, then Et_3N (3.0 equiv.), Ac_2O (3.0 equiv.), THF, rt, 3 h;

(e) triyne 25 (1.0 equiv.), CpCo(CO)(fum) (0.5 equiv.), PhCl, 170 °C,20 min, then *p*-TsOH.H_2O (5.0 equiv.), 95 °C, 1h;

(f) ester 26 (1.0 equiv.), KOH (10.0 equiv.), THF:MeOH (1:1), 65 °C, 3 h, then $MeSO_3H$, 80°C, 1h.

Methyl 3-[4-(ethoxymethoxy)-4-{2-[(trimethylsilyl)ethynyl]phenyl}but-1-yn-1-yl]naphthalene-2-carboxylate 22

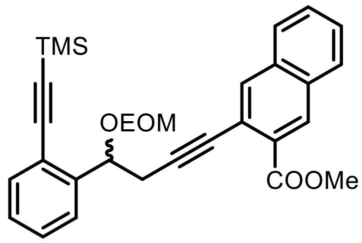

A Schlenk flask was charged with methyl 3-iodonaphthalene-2-carboxylate (568 mg, 1.82 mmol), $Pd(PPh_3)_2Cl_2$ (25.5 mg, 0.04 mmol, 2 mol%), CuI (13.9 mg, 0.07 mmol, 4 mol%), flushed with nitrogen, and the degassed *N*,*N*-diisopropylamine (15 mL) was added. The mixture was heated to 45 °C before a solution of alkyne **17** (600 mg, 2.00 mmol, 1.1 equiv.) in degassed toluene (5 mL) was slowly added. The reaction mixture was stirred at the same temperature for 3 h. The solvents were evaporated under the reduced pressure. The residue was chromatographed on silica gel (hexane-ethyl acetate 20:1) to afford the desired product **22** (679 mg, 77%) as an oil.

^{1}H NMR (400 MHz, $CDCl_3$): 0.27 (s, 9H), 1.15 (t, *J* = 7.1, 3H), 2.98 (dd, *J* = 17.0, 7.3, 1H), 3.09 (dd, *J* = 17.0, 4.6, 1H), 3.55 (dq, *J* = 9.5, 7.1, 1H), 3.86 (dq, *J* = 9.4, 7.0, 1H), 3.93 (s, 3H), 4.70 (d, *J* = 6.8, 1H), 4.84 (d, *J* = 6.9, 1H), 5.47 (dd, *J* = 7.3, 4.6, 1H), 7.24 (td, *J* = 7.6, 1.3, 1H), 7.37 (td, *J* = 7.6, 1.4, 1H), 7.48 (ddd, *J* = 7.6, 1.4, 0.6, 1H), 7.51 (dd, *J* = 8.1, 1.3, 1H), 7.56 (ddd, *J* = 8.2, 6.9,

1.4, 1H), 7.61 (ddd, J = 7.8, 1.3, 0.6, 1H), 7.76 (ddq, J = 8.0, 1.3, 0.7, 1H), 7.86 (ddq, J = 8.0, 1.3, 0.7, 1H), 7.97 (s, 1H), 8.43 (s, 1H).

^{13}C NMR (101 MHz, $CDCl_3$): 0.1 (3C), 15.2, 28.2, 52.3, 63.6, 74.3, 80.8, 91.2, 93.7, 100.2, 102.7, 120.2, 121.8, 126.2, 127.2, 127.3, 127.5, 128.6, 128.9, 128.97, 129.03, 131.5, 131.6, 132.3, 134.4, 134.5, 143.6, 167.0.

IR ($CHCl_3$): 3061 w, 2976 m, 2955 m, 2898 w, 2886 w, sh, 2844 w, 2231 w, 2156 m, 1729 s, 1628 w, 1599 w, 1591 w, 1571 w, 1495 w, 1478 w, 1464 m, 1447 m, 1433 w, 1415 w, 1329 m, 1283 vs, 1272 s, 1251 s, 1232 m, 1160 w, sh, 1149 m, 1112 m, 1100 m, 1092 m, 1043 s, 1018 s, 957 w, 913 w, 897 m, 867 vs, 845 vs, 700 w, 646 w, 477 m cm^{-1}.

EI MS: 484 ($M^{+\bullet}$, 2), 408 (16), 305 (18), 261 (39), 223 (53), 179 (24), 165 (18), 143 (100), 115 (71), 73 (82).

HR EI MS: calcd for $C_{30}H_{32}O_4Si$ 484.2064, found 484.2057.

Methyl 3-[4-(ethoxymethoxy)-4-(2-ethynylphenyl)but-1-yn-1-yl]naphthalene-2-carboxylate 23

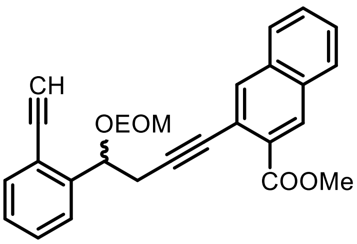

Diyne **22** (649 mg, 1.34 mmol) was dissolved in methanol (20 mL) and potassium carbonate (278 mg, 2.01 mmol, 1.5 equiv.) was added. The mixture was stirred at room temperature for 15 min. The reaction was quenched with a saturated ammonium chloride solution (50 mL), extracted with dichloromethane (2 x 30 mL), and the combined organic layers were dried over anhydrous magnesium sulfate. The solvents were evaporated under the reduced pressure and the residue was purified by flash chromatography on silica gel (hexane-ethyl acetate 10:1) to afford **23** (520 mg, 94%) as a colorless oil.

^{1}H NMR (400 MHz, $CDCl_3$): 1.13 (t, J = 7.1, 3H), 3.01 (dd, J = 17.0, 7.2, 1H), 3.08 (dd, J = 17.0, 5.0, 1H), 3.38 (s, 1H), 3.54 (dq, J = 9.4, 7.1, 1H), 3.81 (dq, J = 9.5, 7.1, 1H), 3.94 (s, 3H), 4.70 (d, J = 6.9, 1H), 4.85 (d, J = 6.9, 1H), 5.46 (dd, J = 7.2, 5.0, 1H), 7.27 (td, J = 7.6, 1.3, 1H), 7.41 (td, J = 7.7, 1.4, 1H), 7.51 (ddd, J = 8.2, 6.9, 1.4, 1H), 7.52 (dd, J = 8.0, 1.4, 1H), 7.56 (ddd, J = 8.2, 6.9, 1.4, 1H), 7.61 – 7.65 (m, 1H), 7.77 (dq, J = 8.1, 0.6, 1H), 7.87 (dq, J = 8.0, 0.7, 1H), 7.97 (s, 1H), 8.43 (s, 1H).

^{13}**C NMR** (101 MHz, $CDCl_3$): 15.1, 28.4, 52.3, 63.7, 74.4, 81.0, 81.3, 82.6, 90.9, 93.8, 120.0, 120.7, 126.5, 127.27, 127.32, 127.6, 128.7, 129.0, 129.1, 129.2, 131.5, 131.6, 133.0, 134.4, 134.5, 143.8, 167.1.

IR ($CHCl_3$): 3304 m, 3061 w, 2979 w, 2953 w, 2887 w, 2844 w, sh, 2230 w, 2106 vw, 1727 s, 1628 w, 1602 w, sh, 1591 w, 1572 w, 1495 w, 1481 w, 1464 m, 1447 m, 1433 w, 1329 w, 1284 vs, 1271 s, sh, 1231 m, 1158 w, 1149 w, 1111 m, 1100 m, 1093 m, sh, 1042 s, 1018 s, 957 w, 914 w, 898 w, 657 w, 620 w, 477 w cm^{-1}.

ESI MS: 435 ($[M+Na]^+$).

HR APCI MS: calcd for $C_{27}H_{24}O_4Na$ 435.1567, found 435.1566.

<u>Methyl 3-[4-(ethoxymethoxy)-4-{2-[(2-formylnaphthalen-1-yl)ethynyl]phenyl}but-1-yn-1-yl]naphthalene-2-carboxylate</u> 24

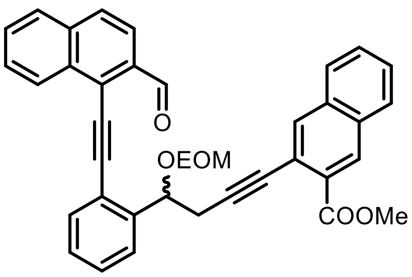

A Schlenk flask was charged with 1-bromo-2-naphthaldehyde (207 mg, 0.88 mmol), bis(triphenylphosphine)palladium chloride (12.4 mg, 0.02 mmol, 2 mol%), copper(I) iodide (6.70 mg, 0.04 mmol, 4 mol%) and flushed with argon. The degassed tetrahydrofuran (5 mL) and degassed triethylamine (5 mL) were added and the mixture was heated to 50 °C. Then diyne **23** (400 mg, 0.97 mmol, 1.1 equiv.) in degassed tetrahydrofuran (5 mL) was slowly added and the reaction was stirred at 50 °C for 3 hours. The solvents were evaporated under the reduced pressure. The residue was chromatographed on silica gel (hexane-ethyl acetate 20:1 to 10:1) to afford the desired product **24** (389 mg, 78%).

^{1}**H NMR** (400 MHz, $CDCl_3$): 1.12 (t, *J* = 7.1, 3H), 3.14 – 3.17 (m, 2H), 3.57 (dq, *J* = 9.5, 7.0, 1H), 3.80 (s, 3H), 3.88 (dq, *J* = 9.5, 7.1, 1H), 4.76 (d, *J* = 7.0, 1H), 4.89 (d, *J* = 7.0, 1H), 5.68 (t, *J* = 6.4, 1H), 7.41 (td, *J* = 7.5, 1.4, 1H), 7.46 – 7.54 (m, 3H), 7.55 – 7.64 (m, 3H), 7.70 – 7.76 (m, 2H), 7.80 – 7.88 (m, 3H), 7.87 (s, 1H), 7.97 (d, *J* = 8.6, 1H), 8.37 (s, 1H), 8.64 – 8.69 (m, 1H), 10.94 (d, *J* = 0.9, 1H).

^{13}**C NMR** (101 MHz, $CDCl_3$): 15.2, 28.9, 52.2, 63.8, 74.8, 81.4, 88.1, 90.6, 93.6, 100.1, 119.9, 121.4, 122.2, 127.0, 127.25, 127.3 (2C), 127.5, 127.9, 128.0, 128.5, 128.6, 128.8, 128.9, 129.1, 129.4, 129.8, 131.5, 131.6, 132.9, 133.3, 134.3, 134.5, 134.6, 135.9, 143.4, 166.8, 192.0.

IR ($CHCl_3$): 3062 w, 2978 w, 2952 m, 2930 m, 2889 w, 2229 w, 2203 w, 1728 s, 1694 vs, 1679 s, 1628 w, 1618 w, 1592 w, 1568 w, 1506 w, 1495 w, 1484 w, 1464 w, 1458 w, 1447 m, 1433 m, 1402 w, sh, 1385 w, 1332 m, 1284 vs, 1272 s, sh, 1257 m, 1159 w, 1149 w, 1111 m, 1095 m, 1043 m, sh, 1028 m, sh, 1018 s, 957 w, 916 w, 898 w, 871 w, 822 m, 657 w, 638 w, 570 w, 477 m, 435 w cm^{-1}.

ESI MS: 589 ($[M+Na]^+$).

HR ESI MS: calcd for $C_{38}H_{30}O_5Na$ 589.1986, found 589.1985.

Methyl 3-{4-[2-({2-[1-(acetyloxy)but-3-yn-1-yl]naphthalen-1-yl}ethynyl)phenyl]-4-(ethoxymethoxy)but-1-yn-1-yl}naphthalene-2-carboxylate 25

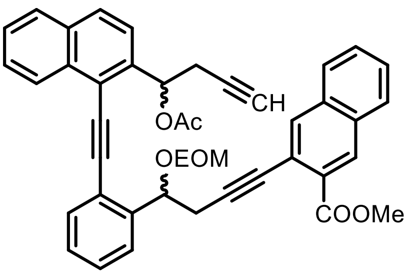

A Schlenk flask was filled with zinc powder (85.0 mg, 1.30 mmol, 2 equiv.) and flushed with argon. Freshly distilled tetrahydrofuran (3 mL) was added and vigorously stirred. Then propargyl bromide (80 wt. % in toluene, 145 µL, 1.30 mmol, 2 equiv.) was added and stirred for 10 min, before it was transferred by a syringe to the second Schlenk flask, which contained aldehyde **24** (368 mg, 0.65 mmol) and tetrahydrofuran (15 mL). The reaction mixture was stirred for 5 min at room temperature, before triethylamine (266 µL, 1.95 mmol, 3 equiv.) and acetic anhydride (183 µL, 1.95 mmol, 3 equiv.) were added and the solution was stirred for 3 h at room temperature. The solvents were evaporated under the reduced pressure and the residue was purified by flash chromatography on silica gel (hexane-ethyl acetate 10:1) to obtain acetate **25** (332 mg, 79%) as a mixture of diastereomers in a ratio about 1:1.

^{1}H NMR (400 MHz, $CDCl_3$, mixture of diastereomers): 1.10 (t, J = 7.1, 3H), 1.11 (t, J = 7.1, 3H), 2.00 (t, J = 2.6, 1H), 2.03 (t, J = 2.6, 1H), 2.13 (s, 3H), 2.14 (s, 3H), 2.90 – 3.07 (m, 4H), 3.17 (dd, J = 17.0, 7.2, 2H), 3.24 (dd, J = 17.0, 5.3, 2H), 3.54 – 3.63 (m, 2H), 3.81 (s, 3H), 3.82 (s, 3H), 3.82 – 3.90 (m, 2H), 4.80 (d, J = 6.9, 1H), 4.81 (d, J = 6.9, 1H), 4.92 (d, J = 6.8, 1H), 4.93 (d, J = 6.9, 1H), 5.69 (dd, J = 7.2, 5.3, 1H), 5.70 (dd, J = 7.2, 5.2, 1H), 6.75 (t, J = 6.1, 2H), 7.379 (td, J = 7.4, 1.4, 1H), 7.380 (td, J = 7.4, 1.4, 1H), 7.43 – 7.56 (m, 10H), 7.58 – 7.63 (m, 2H), 7.64 (d, J = 8.6, 2H), 7.72 (dt, J = 7.8, 1.5, 2H), 7.75 – 7.78 (m, 2H), 7.81 - 7.85 (m, 4H), 7.86 (d, J = 8.5, 2H), 7.89 (s, 2H), 8.39 (s, 2H), 8.52 – 8.58 (m, 2H).

^{13}C NMR (101 MHz, $CDCl_3$, mixture of diastereomers): 15.16, 15.19, 21.2 (2C), 25.8, 25.9, 28.7, 28.9, 52.2 (2C), 63.8 (2C), 71.12, 71.14, 72.2, 72.3, 75.0, 75.1, 79.5 (2C), 81.2 (2C), 89.7, 89.8,

90.99, 91.03, 93.9, 94.0, 98.4 (2C), 118.85, 118.88, 120.1 (2C), 121.71, 121.72, 123.17, 123.23, 126.7 (2C), 126.77, 126.81 (2C), 126.9, 127.2 (2C), 127.3 (2C), 127.5 (2C), 127.9 (2C), 128.2 (2C), 128.5 (2C), 128.9 (2C), 129.0 (2C), 129.05 (2C), 129.1 (2C), 131.5 (2C), 131.6, 131.6, 132.87 (2C), 132.93, 133.0, 133.2 (2C), 134.3 (2C), 134.5 (2C), 139.5 (2C), 142.9, 143.0, 167.0 (2C), 169.86, 169.89.

IR ($CHCl_3$): 3309 w, 3061 w, 2977 w, 2953 w, 2930 w, 2888 w, sh, 2842 w, sh, 2230 w, 2202 vw, 2124 vw, 1732 vs, 1628 w, 1592 w, 1571 w, 1509 w, 1494 w, 1484 w, 1464 w, 1447 m, 1433 w, 1372 m, 1329 m, 1283 vs, 1273 s, sh, 1234 vs, 1149 w, 1112 m, 1095 m, 1044 s, 1024 s, sh, 1019 s, 957 w, 915 w, 898 w, 867 w, 821 m, 641 w, 598 vw, 477 w, 437 w cm^{-1}.

ESI MS: 671 ($[M+Na]^+$).

HR ESI MS: calculated for $C_{43}H_{36}O_6Na$ 671.2404, found 671.2403.

Methyl 3-hexahelicen-7-ylnaphthalene-2-carboxylate 26

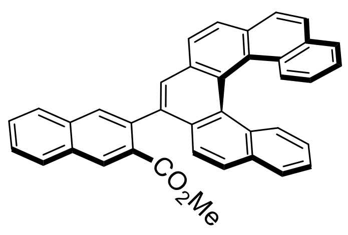

Triyne **25** (310 mg, 0.48 mmol) was loaded to a microwave vial and chlorobenzene (12 mL) was added. The solution was bubbled with nitrogen for 5 minutes before CpCo(CO)(fum) (70.8 mg, 0.24 mmol, 0.5 equiv, fum = dimethylfumarate) was added. The seal was closed and the reaction mixture was heated to 170 °C in the microwave reactor for 20 minutes. *p*-Toluenesulfonic acid monohydrate (694 mg, 3.65 mmol, 5 equiv.) was added and the reaction mixture was strirred for 1 h at 95 °C. The solvent was evaporated in vacuum. The residue was purified by flash chromatography on silica gel (hexane-ethyl acetate 20:1 to 10:1) to obtain helicene **26** (172 mg, 70%) as a mixture of two atropodiastereomers in a ratio 2:1.

^{1}H NMR (400 MHz, $CDCl_3$, major atropodiastereomer): 3.37 (s, 3H), 6.66 – 6.76 (m, 2H), 7.19 – 7.27 (m, 2H), 7.60 (d, *J* = 8.5, 1H), 7.62 – 7.72 (m, 4H), 7.78 (d, *J* = 8.5, 1H), 7.79 (m, 1H), 7.85 (dd, *J* = 8.0, 1.5, 1H), 7.91 – 7.99 (m, 4H), 8.00 – 8.04 (m, 2H), 8.07 (bs, 1H), 8.10 (m, 1H), 8.76 (s, 1H).

^{13}C NMR (101 MHz, $CDCl_3$, major atropodiastereomer): 52.2, 123.7, 123.9, 124.92, 124.93, 125.7, 125.8, 126.5, 126.7, 127.0, 127.2, 127.5, 127.66, 127.68, 127.8, 127.91, 127.94 (2C), 128.0, 128.2, 128.3, 128.8, 129.2, 129.6, 129.9, 130.1, 130.7, 131.1, 131.3, 131.4, 132.0 (2C), 132.2, 132.5, 135.1, 137.5, 139.5, 168.1.

^{1}H NMR (400 MHz, $CDCl_3$, minor atropodiastereomer): 3.28 (s, 3H), 6.66 – 6.76 (m, 2H), 7.19 – 7.27 (m, 2H), 7.60 (d, *J* = 8.5, 1H), 7.62 – 7.72 (m, 4H), 7.78 (d, *J* = 8.5, 1H), 7.79 (m, 1H), 7.83 (m, 1H), 7.91 – 7.99 (m, 4H), 8.00 – 8.04 (m, 2H), 8.08 (bs, 1H), 8.10 (m, 1H), 8.69 (s, 1H).

^{13}C NMR (101 MHz, $CDCl_3$, minor atropodiastereomer): 52.0, 123.9 (2C), 124.60, 124.7, 125.6, 125.7, 126.4, 127.1, 127.2, 127.3, 127.4, 127.6, 127.7, 127.8, 127.87, 127.91, 127.94, 128.0, 128.2, 128.4, 128.7, 129.1, 130.02, 130.03, 130.2, 130.7, 131.3, 131.4, 131.5, 131.6, 132.0, 132.2, 132.6, 134.7, 137.4, 138.5, 168.1.

IR ($CHCl_3$): 3061 w, sh, 3052 w, 2952 w, 2928 w, 2855 w, 2844 w, sh, 1724 s, 1713 s, sh, 1631 w, 1594 w, 1502 w, 1489 w, 1460 w, 1447 m, 1433 w, 1326 w, 1282 vs, 1270 s, sh, 1241 w, 1198 m, 1149 w, 1137 w, 1132 w, 1075 m, 1043 w, 958 w, 915 w, 902 w, 884 w, 867 w, 833 s, 809 m, 627 w, 620 m, 525 m, 479 w cm^{-1}.

ESI MS: 535 ($[M+Na]^+$).

HR ESI MS: calculated for $C_{38}H_{24}O_2Na$ 535.1669, found 535.1670.

12*H*-Anthra[3,2,1-*gh*]hexahelicen-12-one 2A

19*H*-Benzo[5,6]indeno[1,2-*i*]hexahelicen-19-one 2F

Ester **26** (151 mg, 0.29 mmol) was dissolved in tetrahydrofuran (5 mL) and methanol (5 mL), and potassium hydroxide (163 mg, 2.90 mmol, 10 equiv.) was added. The mixture was heated to 65 °C and stirred for 3 h. Then the reaction was cooled to rt and diluted hydrochloric acid was added to reach pH 1. The mixture was extracted with diethyl ether (2x 20 mL), and the combined organic layers were dried over anhydrous magnesium sulfate. The solvents were evaporated under the reduced pressure to obtain crude carboxylic acid, which was directly used further. This acid was dissolved in methanesulfonic acid (10 mL) and the flask was put to the oil bath pre-heated to 80 °C. The stirring was continued for 1 h at 80 °C and then the reaction mixture was poured over crushed ice. The mixture was extracted with dichloromethane (2x 20 mL), the combined organic layers were washed with saturated sodium bicarbonate solution (10 mL), dried over anhydrous magnesium sulfate. The solvent was evaporated *in vacuo* and the residue was purified by flash chromatography on silica gel (hexane-toluene 1:1 to pure toluene) to afford **2A** (21.00 mg, 15%) as an orange solid and **2F** (94.6 mg, 68%) as a red solid.

12*H*-Anthra[3,2,1-*gh*]hexahelicen-12-one 2A

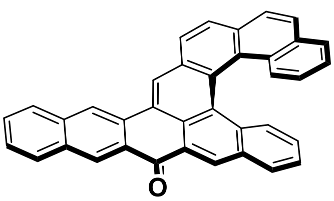

M.p.: 298 - 299 °C (CH_3CN).

Optical rotation: $[\alpha]^{20}_{D}$ +770° (c 0.027, CH_2Cl_2); $[\alpha]^{20}_{D}$ -748° (c 0.022, CH_2Cl_2).

^{1}H NMR (600 MHz, CD_2Cl_2): 6.69 (ddd, *J* = 8.4, 6.8, 1.4, 1H), 6.84 (ddd, *J* = 8.3, 6.7, 1.4, 1H), 7.25 (ddd, *J* = 7.9, 6.8, 1.2, 1H), 7.35 (ddd, *J* = 7.9, 6.7, 1.1, 1H), 7.56 (d, *J* = 8.5, 1H), 7.62 (ddd, *J* = 8.0, 6.6, 1.2, 1H), 7.69 (d, *J* = 8.1, 1H), 7.70 (ddd, *J* = 7.8, 6.6, 1.2, 1H), 7.86 (ddd, *J* = 7.9, 1.4, 0.5, 1H), 7.98 (d, *J* = 8.4, 1H), 7.99 (d, *J* = 8.5, 1H), 8.10 (d, *J* = 8.1, 1H), 8.12 (bd, *J* = 8.2, 1H), 8.14 (m, 2H), 8.21 (dd, *J* = 8.1, 0.4, 1H), 9.02 (s, 1H), 9.07 (s, 1H), 9.11 (s, 1H), 9.23 (s, 1H).

^{13}C NMR (151 MHz, CD_2Cl_2): 123.95, 124.0, 125.3, 125.5, 126.35, 126.37, 126.76, 126.79, 127.0, 127.2, 127.7, 127.8 (2C), 128.2, 128.3, 128.4, 128.5, 128.9, 129.0, 129.07, 129.12, 129.3, 129.5, 130.1, 130.3, 130.4, 130.6, 131.3, 132.3, 132.5, 132.71, 132.73, 133.0, 133.2, 133.7, 136.8, 185.0.

IR ($CHCl_3$): 3057 w, 2957 w, 2928 w, 2855 w, 1660 vs, 1624 s, 1611 w, 1595 m, 1586 w, 1577 w, 1522 w, 1513 w, 1494 w, 1445 w, 1441 w, 1358 w, 1343 m, 1286 w, 1275 m, 1266 w, 1192 vs, 1168 vw, 1036 vw, 1021 w, 959 w, 926 vw, 887 w, 833 m, 805 w, 644 w, 626 w, 608 w, 580 w, 517 w, 509 w, 475 w, sh cm^{-1}.

APCI MS: 481 ($[M+H]^+$).

HR APCI MS: calcd for $C_{37}H_{21}O$ 481.1587, found 481.1583.

<u>19*H*-Benzo[5,6]indeno[1,2-*i*]hexahelicen-19-one</u> 2F

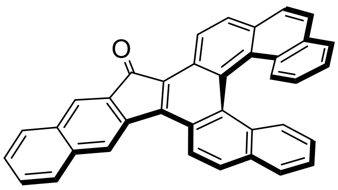

M.p.: 309 - 310 °C (CH_3CN).

^{1}H NMR (600 MHz, CD_2Cl_2): 6.71 (ddd, *J* = 8.5, 6.9, 1.3, 2H), 7.22 (ddd, *J* = 7.9, 6.8, 1.1, 1H), 7.30 (ddd, *J* = 7.9, 6.8, 1.1, 1H), 7.46 (ddt, *J* = 8.6, 1.2, 0.6, 1H), 7.553 (ddt, *J* = 8.6, 1.1, 0.6, 1H), 7.555 (ddd, *J* = 8.0, 6.9, 1.3, 1H), 7.64 (ddd, *J* = 8.1, 6.9, 1.3, 1H), 7.83 (ddt, *J* = 7.9, 1.3, 0.6, 1H), 7.89 (ddt, *J* = 7.8, 1.3, 0.6, 1H), 7.95 (d, *J* = 8.6, 1H), 7.968 (ddq, *J* = 7.9, 1.3, 0.7, 1H), 7.970 (d, *J* = 8.6, 1H), 8.03 (ddq, *J* = 8.0, 1.3, 0.7, 1H), 8.14 (d, *J* = 8.4, 1H), 8.16 (dt, *J* = 8.8, 0.7, 1H), 8.22 (q, *J* = 0.7, 1H), 8.58 (t, *J* = 0.7, 1H), 8.90 (d, *J* = 8.9, 1H), 9.47 (d, *J* = 8.4, 1H).

^{13}C NMR (151 MHz, CD_2Cl_2): 122.3, 122.7, 123.6, 125.0, 125.3, 125.8, 125.9, 126.1, 126.5, 127.0, 127.5, 127.9, 128.0, 128.1, 128.2, 128.8, 128.9, 129.15, 129.17, 129.4, 129.5, 129.6, 129.8, 130.1, 130.2, 130.5, 130.8, 131.8, 132.7, 133.2, 133.8, 134.89, 134.94, 137.4, 138.5, 144.4, 194.4.

IR ($CHCl_3$): 3066 w, 3045 w, 2953 w, 2921 m, 2851 m, 1692 s, 1629 vs, 1605 w, 1594 w, 1566 vw, 1511 w, sh, 1470 w, 1454 w, 1377 vw, 1333 w, 1298 w, 1243 w, 1183 w, 1171 w, 1149 w, sh, 1119 w, 1097 w, 1051 w, 919 w, 904 w, 883 m, 866 w, 841 vs, 828 w, 811 w, 667 w, 638 w, 626 w, 604 w, 580 w, 533 w, 522 w, 497 w, 473 m cm^{-1}.

APCI MS: 481 ($[M+H]^+$).

HR APCI MS: calcd for $C_{37}H_{21}O$ 481.1587, found 481.1581.

Synthesis of compounds 3A and 3F

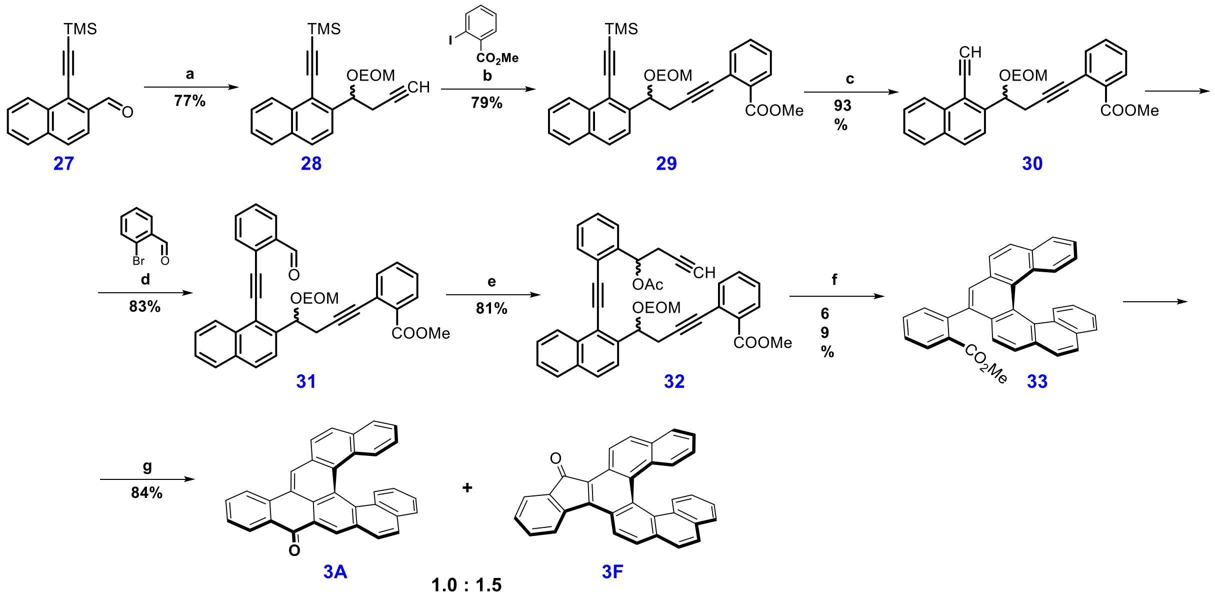

(a) Compound 27 (1.0 equiv.), Zn (2.0 equiv.), propargyl bromide (2.0 equiv.), THF, rt, 25 min, then DMAP (cat.), *N*,*N*-diisopropylethylamine (3.0 equiv.), chloromethyl ethyl ether (2.0 equiv.), DCM, rt, 16 h;

(b) methyl 2-iodobenzoate (1.0 equiv.), diyne 28 (1.1 equiv.), $Pd(PPh_3)Cl_2$ (2 mol%), CuI (4 mol%), *i*-Pr_2NH:PhMe (3:1), 45 °C, 2 h;

(c) compound 29 (1.0 equiv.), K_2CO_3 (1.5 equiv.), MeOH, rt, 10 min;

(d) 2-bromobenzaldehyde (1.0 equiv.), diyne 30 (1.1equiv.), $Pd(PPh_3)Cl_2$ (2 mol%), CuI (4 mol%), THF:Et_3N (2:1), 50 °C, 4 h;

(e) Zn (2.0 equiv.), propargyl bromide (2.0 equiv.), compound 31 (1.0 equiv.), THF, rt, 15 min, then Et_3N (3.0 equiv.), Ac_2O (3.0 equiv.), THF, rt, 2 h;

(f) triyne 32 (1.0 equiv.), CpCo(CO)(fum) (0.5 equiv.), PhCl, 170 °C, 20 min, then *p*-TsOH.H_2O (5.0 equiv.), 95 °C, 1h.

(g) ester 33 (1.0 equiv.), KOH (10.0 equiv.), THF:MeOH (1:1), 65 °C, 4 h, then $MeSO_3H$, 80°C, 1h.

<u>**({2-[1-(Ethoxymethoxy)but-3-yn-1-yl]naphthalen-1-yl}ethynyl)(trimethyl)silane**</u> **28**

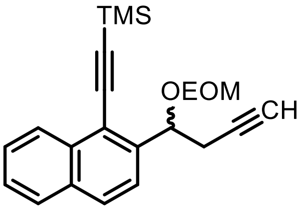

A Schlenk flask was charged with zinc powder (883 mg, 13.5 mmol, 2.0 equiv.) and flushed with nitrogen. The freshly distilled tetrahydrofuran (6 mL) was added, the suspension was put to a water bath at room temperature and vigorously stirred. Then propargyl bromide (80 wt. % in toluene, 1.50 mL, 13.5 mmol, 2.0 equiv.) was slowly added within 10 min and the mixture was then stirred at room temperature for 10 min before it was transferred by a syringe to the second Schlenk flask with a suspension of aldehyde **27** (1.70 g, 6.74 mmol) in tetrahydrofuran (20 mL). The reaction mixture was stirred at room temperature for 5 min, then quenched with a saturated solution of ammonium chloride (20 mL) and extracted with ethyl acetate (2 x 20 mL). The combined organic layers were dried over anhydrous $MgSO_4$ and evaporated *in vacuo*. The residue was dissolved in dichloromethane (25 mL), DMAP (5 mg, cat.), *N*,*N*-diisopropylethylamine (3.52 mL, 20.2 mmol, 3.0 equiv.), and chloromethyl ethyl ether (1.25 mL, 13.5 mmol, 2.0 equiv.) were added successively. The solution was stirred at room temperature overnight and then quenched with brine (20 mL). The layers were separated and water layer was extracted with dichloromethane (10 mL). The organic layers were dried over anhydrous $MgSO_4$, solvents were removed *in vacuo*, and the residue was chromatographed on silica gel (hexane-ethyl acetate 40:1) to afford the desired product **28** (1.82 g, 77%) as a slightly yellow oil.

^{1}H NMR (400 MHz, $CDCl_3$): 0.36 (s, 9H), 1.19 (t, *J* = 7.1, 3H), 2.00 (t, *J* = 2.6, 1H), 2.73 (ddd, *J* = 16.9, 7.5, 2.6, 1H), 2.80 (ddd, *J* = 16.9, 4.7, 2.7, 1H), 3.55 (dq, *J* = 9.4, 7.1, 1H), 3.87 (dq, *J* = 9.4, 7.1, 1H), 4.64 (d, *J* = 6.8, 1H), 4.79 (d, *J* = 6.8, 1H), 5.63 (dd, *J* = 7.5, 4.6, 1H), 7.51 (ddd, *J* = 8.1, 6.9, 1.3, 1H), 7.58 (ddd, *J* = 8.3, 6.8, 1.4, 1H), 7.66 (d, *J* = 8.6, 1H), 7.80 – 7.86 (m, 2H), 8.36 (ddt, *J* = 8.3, 1.4, 0.8, 1H).

^{13}C NMR (101 MHz, $CDCl_3$): 0.2 (3C), 15.2, 26.8, 63.7, 69.9, 74.3, 81.2, 93.5, 100.2, 106.4, 119.1, 123.3, 126.5, 126.6, 127.2, 128.2, 129.0, 132.7, 133.3, 142.3.

IR ($CHCl_3$): 3309 m, 3061 w, 2977 w, 2961 w, 2897 w, 2887 w, sh, 2151 w, 2125 w, sh, 1592 w, 1568 w, 1507 w, 1409 w, 1262 w, 1251 m, 1148 w, 1099 w, 1049 m, 1028 m, 874 s, 846 vs, 825 m, 701 w, 646 m, 450 w cm^{-1}.

EI MS: 350 ($M^{+•}$, 3), 311 (44), 282 (50), 267 (33), 193 (72), 165 (78), 73 (100).

HR EI MS: calcd for $C_{22}H_{26}O_2Si$ 350.1697, found 350.1696.

<u>Methyl 2-[4-(ethoxymethoxy)-4-{1-[(trimethylsilyl)ethynyl]naphthalen-2-yl}but-1-yn-1-yl]benzoate</u> 29

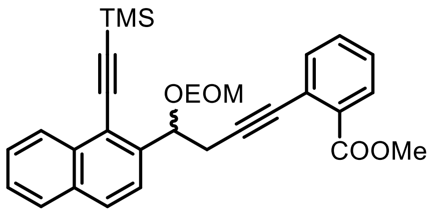

A Schlenk flask was charged with methyl 2-iodobenzoate (781 mg, 2.98 mmol), $Pd(PPh_3)_2Cl_2$ (42.1 mg, 0.06 mmol, 2 mol%), CuI (22.9 mg, 0.12 mmol, 4 mol%), flushed with nitrogen, and the degassed *N*,*N*-diisopropylamine (30 mL) was added. The mixture was heated to 45 °C before a solution of alkyne **28** (1.15 g, 3.28 mmol, 1.1 equiv.) in degassed toluene (10 mL) was slowly added. The reaction mixture was stirred at the same temperature for 2 h. The solvents were evaporated under the reduced pressure. The residue was chromatographed on silica gel (hexane-ethyl acetate 20:1) to afford the desired product **29** (1.14 g, 79%) as a yellow oil.

^{1}H NMR (400 MHz, $CDCl_3$): 0.34 (s, 9H), 1.15 (t, *J* = 7.1, 3H), 3.06 (dd, *J* = 16.9, 6.7, 1H), 3.11 (dd, *J* = 16.9, 5.7, 1H), 3.55 (dq, *J* = 9.5, 7.1, 1H), 3.82 (s, 3H), 3.83 (dq, *J* = 9.4, 7.1, 1H), 4.69 (d, *J* = 6.8, 1H), 4.84 (d, *J* = 6.8, 1H), 5.72 (t, *J* = 6.1, 1H), 7.30 (td, *J* = 7.3, 1.5, 1H), 7.38 (td, *J* = 7.7, 1.5, 1H), 7.45 (ddd, *J* = 7.8, 1.4, 0.7, 1H), 7.51 (ddd, *J* = 8.1, 6.8, 1.3, 1H), 7.59 (ddd, *J* = 8.4, 6.9, 1.4, 1H), 7.73 (d, *J* = 8.6, 1H), 7.81 – 7.87 (m, 2H), 7.87 (ddd, *J* = 7.8, 1.4, 0.6, 1H), 8.38 (bd, *J* = 8.4, 1H).

^{13}C NMR (101 MHz, $CDCl_3$): 0.2 (3C), 15.2, 28.1, 52.1, 63.7, 74.6, 80.8, 92.3, 93.7, 100.4, 106.1, 119.1, 123.6, 124.4, 126.5 (2C), 127.1, 127.4, 128.2, 128.9, 130.2, 131.5, 132.0, 132.7, 133.3, 134.6, 142.5, 166.9.

IR ($CHCl_3$): 3062 w, 2977 w, 2955 m, 2898 w, 2887 w, sh, 2845 w, 2236 w, 2151 w, 1729 s, 1720 m, sh, 1596 w, 1568 w, 1507 w, 1486 m, 1449 w, 1435 w, 1411 w, sh, 1299 m, 1277 m, 1252 s, 1163 w, 1146 w, sh, 1131 m, 1098 m, 1043 m, 1028 s, 965 w, 879 m, 874 m, 846 s, 825 m, 701 w, 644 w, 450 w cm^{-1}.

EI MS: 484 ($M^{+\bullet}$, 2), 311 (70), 282 (67), 267 (52), 239 (22), 193 (93), 178 (20), 165 (74), 73 (100).

HR EI MS: calcd for $C_{30}H_{32}O_4Si$ 484.2064, found 484.2062.

<u>Methyl 2-[4-(ethoxymethoxy)-4-(1-ethynylnaphthalen-2-yl)but-1-yn-1-yl]benzoate</u> 30

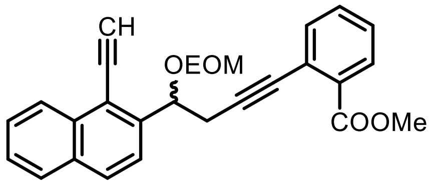

Diyne **29** (1.11 g, 2.29 mmol) was dissolved in methanol (30 mL) and potassium carbonate (475 mg, 3.44 mmol, 1.5 equiv.) was added. The mixture was stirred at room temperature for 10 min. The reaction was quenched with

a saturated ammonium chloride solution (100 mL), extracted with dichloromethane (2 x 50 mL), and the combined organic layers were dried over anhydrous magnesium sulfate. The solvents were evaporated under the reduced pressure and the residue was purified by flash chromatography on silica gel (hexane-ethyl acetate 10:1) to afford **30** (878 mg, 93%) as a colorless oil.

^{1}H NMR (400 MHz, $CDCl_3$): 1.12 (t, *J* = 7.1, 3H), 3.08 (d, *J* = 6.1, 2H), 3.53 (dq, *J* = 9.5, 7.0, 1H), 3.788 (dq, *J* = 9.5, 7.1, 1H), 3.795 (s, 1H), 3.83 (s, 3H), 4.69 (d, *J* = 6.9, 1H), 4.84 (d, *J* = 6.9, 1H), 5.71 (t, *J* = 6.2, 1H), 7.30 (td, *J* = 7.6, 1.5, 1H), 7.38 (td, *J* = 7.5, 1.5, 1H), 7.44 (ddd, *J* = 7.8, 1.5, 0.5, 1H), 7.52 (ddd, *J* = 8.1, 6.9, 1.3, 1H), 7.59 (ddd, *J* = 8.4, 6.9, 1.4, 1H), 7.75 (d, *J* = 8.6, 1H), 7.82 – 7.91 (m, 3H), 8.40 (bd, *J* = 8.4, 1H).

^{13}C NMR (101 MHz, $CDCl_3$): 15.1, 28.3, 52.1, 63.7, 74.7, 79.1, 80.9, 88.1, 92.1, 93.8, 118.0, 123.7, 124.3, 126.4, 126.6, 127.3, 127.4, 128.3, 129.3, 130.2, 131.6, 132.1, 132.7, 133.5, 134.5, 142.8, 167.0.

IR ($CHCl_3$): 3304 m, 3063 w, 2979 m, 2953 m, 2932 w, sh, 2886 w, 2845 w, 2235 w, 2098 w, 1727 vs, 1596 w, 1568 w, 1507 w, 1486 m, 1469 w, sh, 1449 w, 1435 m, 1298 s, 1279 s, 1256 s, 1164 w, 1145 m, 1131 s, 1098 s, 1044 s, 1027 s, 965 w, 869 m, 824 m, 657 m, 617 w cm^{-1}.

EI MS: 412 ($M^{+\bullet}$, 3), 321 (19), 276 (33), 239 (100), 211 (43), 193 (86), 165 (62), 152 (99).

HR EI MS: calcd for $C_{27}H_{24}O_4$ 412.1669, found 412.1660.

__Methyl 2-[4-(ethoxymethoxy)-4-{1-[(2-formylphenyl)ethynyl]naphthalen-2-yl}but-1-yn-1-yl]benzoate__ **31**

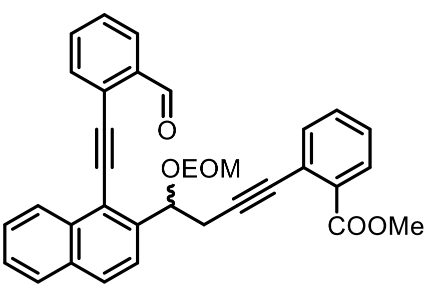

A Schlenk flask was charged with 2-bromobenzaldehyde (224 mg, 1.21 mmol), bis(triphenylphosphine)palladium chloride (17.0 mg, 0.02 mmol, 2 mol%), copper(I) iodide (9.22 mg, 0.05 mmol, 4 mol%) and flushed with argon. The degassed tetrahydrofuran (10 mL) and degassed triethylamine (10 mL) were added and the mixture was heated to 50 °C. Then diyne **30** (550 mg, 1.33 mmol, 1.1 equiv.) in degassed tetrahydrofuran (10 mL) was slowly added and the reaction was stirred at 50 °C for 4 hours. The solvents were evaporated under the reduced pressure. The residue was chromatographed on silica gel (hexane-ethyl acetate 20:1 to 10:1) to afford the desired product **31** (518 mg, 83%).

^{1}**H NMR** (400 MHz, $CDCl_3$): 1.12 (t, *J* = 7.1, 3H), 3.11 (dd, *J* = 16.9, 6.0, 1H), 3.16 (dd, *J* = 16.9, 6.9, 1H), 3.55 (dq, *J* = 9.5, 7.1, 1H), 3.75 (s, 3H), 3.84 (dq, *J* = 9.5, 7.1, 1H), 4.71 (d, *J* = 6.9, 1H), 4.85 (d, *J* = 6.9, 1H), 5.82 (t, *J* = 6.4, 1H), 7.26 – 7.31 (m, 1H), 7.35 (td, *J* = 7.6, 1.5, 1H), 7.42 (ddd, *J* = 7.8, 1.6, 0.6, 1H), 7.49 (dddd, *J* = 8.1, 7.4, 1.3, 0.8, 1H), 7.56 (ddd, *J* = 8.1, 6.8, 1.2, 1H), 7.58 – 7.62 (m, 1H), 7.65 (ddd, *J* = 8.3, 6.9, 1.3, 1H), 7.78 (d, *J* = 8.6, 1H), 7.84 (ddd, *J* = 7.7, 1.3, 0.6, 1H), 7.85 (ddd, *J* = 7.8, 1.5, 0.6, 1H), 7.89 (ddt, *J* = 8.0, 1.2, 0.6, 1H), 7.94 (bd, *J* = 8.7, 1H), 7.98 (ddd, *J* = 7.8, 1.5, 0.6, 1H), 8.45 (ddt, *J* = 8.4, 1.4, 0.8, 1H), 10.78 (d, *J* = 0.8, 1H).
^{13}**C NMR** (101 MHz, $CDCl_3$): 15.1, 28.6, 52.1, 63.8, 74.8, 81.1, 91.96, 91.98, 93.6, 95.9, 118.5, 123.8, 124.2, 126.3, 126.7, 126.8, 127.5, 127.6, 127.9, 128.5, 129.0, 129.8, 130.2, 131.6, 132.0, 132.9, 133.3, 133.9, 134.0, 134.5, 135.9, 142.6, 166.7, 191.4.
IR ($CHCl_3$): 3063 w, 2978 w, 2953 m, 2931 w, 2886 w, 2855 w, 2846 w, sh, 2743 w, 2232 w, 2201 w, 1728 s, 1697 vs, 1654 w, 1594 m, 1568 w, 1508 w, 1486 m, 1449 m, 1435 m, 1298 s, 1277 m, 1255 s, 1163 wm 1147 w, sh, 1131 m, 1097 m, 1045 s, 1027 s, 964 w, 868 w, 825 m, 638 w, 437 w cm^{-1}.
ESI MS: 539 ($[M+Na]^+$).
HR ESI MS: calcd for $C_{34}H_{28}O_5Na$ 539.1829, found 539.1830.

Methyl 2-{4-[1-({2-[1-(acetyloxy)but-3-yn-1-yl]phenyl}ethynyl)naphthalen-2-yl]-4-(ethoxymethoxy)but-1-yn-1-yl}benzoate 32

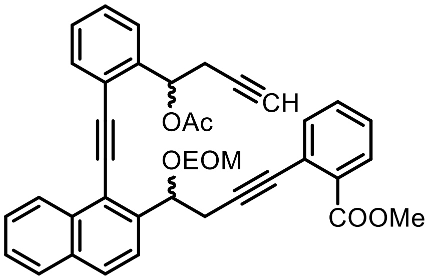

A Schlenk flask was filled with zinc powder (122 mg, 1.86 mmol, 2 equiv.) and flushed with argon. Freshly distilled tetrahydrofuran (4 mL) was added and vigorously stirred. Then propargyl bromide (80 wt. % in toluene, 207 µL, 1.86 mmol, 2 equiv.) was added and stirred for 10 min, before it was transferred by a syringe to the second Schlenk flask, which contained aldehyde **31** (480 mg, 0.93 mmol) and tetrahydrofuran (20 mL). The reaction mixture was stirred for 5 min at room temperature, before triethylamine (380 µL, 2.79 mmol, 3 equiv.) and acetic anhydride (264 µL, 2.79 mmol, 3 equiv.) were added and the solution was stirred for 2 h at room temperature. The solvents were evaporated under the reduced pressure and the residue was purified by flash chromatography on silica gel (hexane-ethyl acetate 10:1) to obtain acetate **32** (451 mg, 81%) as a mixture of diastereomers in a ratio about 1:1.

^{1}H NMR (400 MHz, $CDCl_3$, mixture of diastereomers): 1.09 (t, *J* = 7.1, 3H), 1.10 (t, *J* = 7.1, 3H), 2.03 (t, *J* = 2.8, 1H), 2.04 (t, *J* = 2.7, 1H), 2.14 (s, 6H), 2.91 (dt, *J* = 17.0, 2.5, 2H), 2.88 – 2.96 (m, 2H), 3.00 (dd, *J* = 17.0, 5.4, 1H), 3.01 (dd, *J* = 17.0, 5.5, 1H), 3.10 – 3.23 (m, 4H), 3.546 (dq, *J* = 9.5, 7.1, 1H), 3.553 (dq, *J* = 9.5, 7.1, 1H), 3.767 (s, 3H), 3.773 (s, 3H), 3.77 – 3.86 (m, 2H), 4.75 (d, *J* = 6.8, 1H), 4.76 (d, *J* = 6.8, 1H), 4.88 (d, *J* = 6.8, 1H), 4.89 (d, *J* = 6.8, 1H), 5.80 (t, *J* = 6.1, 2H), 6.56 (t, *J* = 6.1, 2H), 7.26 – 7.44 (m, 10H), 7.51 – 7.57 (m, 4H), 7.64 (ddd, *J* = 8.4, 6.7, 1.2, 2H), 7.72 – 7.76 (m, 2H), 7.80 (d, *J* = 8.6, 2H), 7.83 – 7.92 (m, 6H), 8.55 (bd, *J* = 8.5, 2H).

^{13}C NMR (101 MHz, $CDCl_3$, mixture of diastereomers): 15.1 (2C), 21.1, 21.2, 26.0, 26.1, 28.5, 28.6, 52.10, 52.13, 63.8 (2C), 71.00, 71.04, 72.0, 72.1, 75.1 (2C), 79.5, 79.6, 81.00, 81.04, 90.58, 90.60, 92.3 (2C), 94.0 (2C), 97.37, 97.40, 118.76, 118.79, 121.50, 121.54, 123.85, 123.89, 124.30, 124.33, 126.2, 126.3, 126.6 (2C), 126.6 (2C), 127.4 (4C), 128.2 (2C), 128.3 (2C), 128.91, 128.94, 129.2 (2C), 130.2, 130.3, 131.6 (2C), 132.01, 132.04, 132.8, 132.9, 133.08, 133.11, 133.29, 133.33, 134.5 (2C), 140.5, 140.6, 142.11, 142.14, 166.87, 166.92, 169.8, 169.9.

IR ($CHCl_3$): 3309 m, 3062 w, 2978 w, 2953 w, 2930 w, 2887 w, 2235 w, 2204 vw, 2125 vw, 1732 vs, 1597 w, 1568 w, 1508 w, 1486 m, 1468 w, sh, 1449 m, 1435 m, 1374 m, 1297 m, 1277 m, 1253 s, sh, 1238 vs, 1163 w, 1148 w, sh, 1131 m, 1109 m, 1098 m, 1043 s, 1027 s, 1019 s, sh, 965 w, 868 w, 824 w, 701 w, 641 w, 607 w, 436 w cm^{-1}.

ESI MS: 621 ($[M+Na]^+$).

HR ESI MS: calculated for $C_{39}H_{34}O_6Na$ 621.2248, found 621.2245.

<u>**Methyl 2-hexahelicen-8-ylbenzoate**</u> **33**

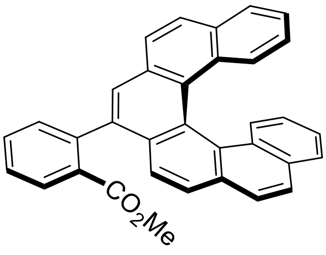

Triyne **32** (420 mg, 0.70 mmol) was loaded to a microwave vial and chlorobenzene (12 mL) was added. The solution was bubbled with nitrogen for 5 minutes before CpCo(CO)(fum) (103 mg, 0.37 mmol, 0.5 equiv, fum = dimethylfumarate) was added. The seal was closed and the reaction mixture was heated to 170 °C in the microwave reactor for 20 minutes. *p*-Toluenesulfonic acid monohydrate (666 mg, 3.50 mmol, 5 equiv.) was added and the reaction mixture was strirred for 1 h at 95 °C. The solvent was evaporated in vacuum. The residue was purified by flash chromatography on silica gel (hexane-ethyl acetate 20:1) to obtain helicene **33** (224 mg, 69%) as a mixture of two atropodiastereomers (AD) in a ratio 1.3:1.

^{1}H NMR (400 MHz, $CDCl_3$, mixture of atropodiastereomers): 3.14 (s, 3H, minor AD), 3.30 (s, 3H, major AD), 6.67 – 6.75 (m, 4H), 7.20 – 7.26 (m, 4H), 7.54 – 7.73 (m, 12H), 7.79 – 7.88 (m, 8H), 7.89 – 7.96 (m, 8H), 8.11 (dd, *J* = 7.8, 1.5, 1H, major AD), 8.14 (dd, *J* = 7.8, 1.5, 1H, minor AD).

^{13}C NMR (101 MHz, $CDCl_3$, mixture of atropodiastereomers): 51.7, 52.0, 124.2, 124.3, 124.4, 124.7, 124.80, 124.82, 124.89, 124.92, 125.5, 125.6 (2C), 125.7, 126.1, 126.2, 126.3, 126.4, 126.9, 127.1 (2C), 127.2, 127.5, 127.6, 127.66 (3C), 127.72, 127.8, 127.90, 127.94, 128.0 (3C), 128.07, 128.12, 128.2, 128.3, 129.9, 130.1, 130.2, 130.36, 130.38, 130.41, 130.43, 130.6, 130.8, 130.9, 131.77, 131.82, 131.87, 131.92, 131.95, 131.98 (3C), 132.2 (2C), 132.3, 132.48, 132.50, 132.51, 138.4, 138.7, 141.1, 141.4, 167.7, 168.2.

IR ($CHCl_3$): 3051 w, 2953 w, 2843 w, sh, 1726 vs, 1717 vs, 1617 vw, 1604 w, 1598 w, 1580 w, 1572 w, 1502 vw, 1490 vw, 1457 vw, 1449 w, 1434 w, 1419 w, 1391 w, 1364 vw, 1297 vs, 1278 s, 1261 s, 1192 m, 1169 w, 1129 m, 1096 w, 1075 w, 1040 w, 965 w, sh, 891 w, 837 m, 825 w, 817 w, 808 m, 687 w, 615 w, 575 w, 517 m cm^{-1}.

ESI MS: 485 ($[M+Na]^+$).

HR ESI MS: calculated for $C_{34}H_{22}O_2Na$ 485.1512, found 485.1511.

<u>12*H*-Naphtho[1,2,3-*jk*]hexahelicen-12-one</u> 3A

<u>13*H*-Indeno[2,1-*i*]hexahelicen-13-one</u> 3F

Ester **33** (195 mg, 0.42 mmol) was dissolved in tetrahydrofuran (5 mL) and methanol (5 mL), and potassium hydroxide (235 mg, 4.20 mmol, 10 equiv.) was added. The mixture was heated to 65 °C and stirred for 4 h. Then the reaction was cooled to rt and diluted hydrochloric acid was added to reach pH 1. The mixture was extracted with diethyl ether (2x 20 mL), and the combined organic layers were dried over anhydrous magnesium sulfate. The solvents were evaporated under the reduced pressure to obtain crude carboxylic acid, which was directly used further. This acid was dissolved in methanesulfonic acid (10 mL) and the flask was put to the oil bath pre-heated to 80 °C. The stirring was continued for 1 h at 80 °C and then the reaction mixture was poured over crushed ice. The mixture was extracted with dichloromethane (2x 20 mL), the combined organic layers were washed with saturated sodium bicarbonate solution (10 mL), dried over anhydrous magnesium sulfate. The solvent was evaporated *in vacuo* and the residue was purified by flash chromatography on silica gel

(hexane-dichloromethane 2:1 to 1:2) to afford **3A** (59.6 mg, 33%) as an orange solid and **3F** (92.3 mg, 51%) as a red solid.

<u>12*H*-Naphtho[1,2,3-*jk*]hexahelicen-12-one</u> 3A

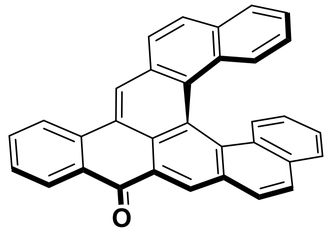

M.p.: 310 – 311 °C (CH_3CN).

Optical rotation: $[\alpha]^{20}_D$ +1094° (c 0.030, CH_2Cl_2); $[\alpha]^{20}_D$ -1031° (c 0.022, CH_2Cl_2).

^{1}H NMR (600 MHz, CD_2Cl_2): 6.72 (ddd, *J* = 8.5, 7.7, 1.5, 1H), 6.74 (ddd, *J* = 8.4, 6.9, 1.4, 1H), 7.25 (ddd, *J* = 8.0, 6.8, 1.2, 1H), 7.29 (ddd, *J* = 7.9, 6.8, 1.2, 1H), 7.45 (dq, *J* = 8.5, 0.9, 1H), 7.58 (dq, *J* = 8.5, 0.8, 1H), 7.63 (ddd, *J* = 8.0, 7.1, 1.1, 1H), 7.84 – 7.89 (m, 3H), 8.00 (bd, *J* = 8.3, 1H), 8.01 (d, *J* = 8.1, 1H), 8.07 (d, *J* = 8.6, 1H), 8.15 (d, *J* = 8.6, 1H), 8.52 (dd, *J* = 7.8, 1.2, 1H), 8.60 (dd, *J* = 7.9, 1.0, 1H), 8.92 (s, 1H), 9.19 (s, 1H).

^{13}C NMR (151 MHz, CD_2Cl_2): 124.1, 125.0, 125.2, 125.4, 125.6, 125.7, 126.5, 126.79, 126.82, 127.21, 127.24, 128.0, 128.2 (3C), 128.8, 129.0, 129.2 (2C), 129.3, 129.7, 129.9, 130.0, 130.9, 131.0, 131.2, 131.4, 132.3, 133.1, 133.7, 134.1, 136.9, 184.0.

IR ($CHCl_3$): 3076 w, 3053 w, 2958 w, 2927 w, 2855 w, 1651 vs, 1602 m, 1594 m, 1576 m, 1557 vw, 1505 w, 1477 w, 1457 vw, 1420 w, 1382 w, 1369 m, 1332 w, sh, 1295 m, 1271 m, 1237 w, 1162 w, 1128 w, 1024 w, 994 w, 922 w, 910 w, 887 w, 814 m, 679 w, 628 w, 610 vw, 517 w, 510 w, 475 w cm^{-1}.

APCI MS: 431 ($[M+H]^+$).

HR APCI MS: calcd for $C_{33}H_{19}O$ 431.1430, found 431.1425.

<u>13*H*-Indeno[2,1-*i*]hexahelicen-13-one</u> 3F

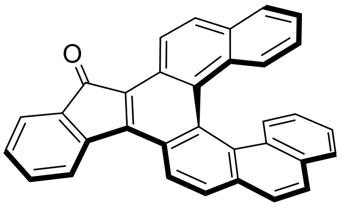

M.p.: 189 – 190 °C (CH_3CN).

^{1}H NMR (400 MHz, $CDCl_3$): 6.63 (ddd, *J* = 8.4, 6.9, 1.4, 1H), 6.69 (ddd, *J* = 8.5, 6.9, 1.4, 1H), 7.18 (ddd, *J* = 8.0, 7.0, 1.1, 1H), 7.20 (ddd, *J* = 7.9, 6.9, 1.0, 1H), 7.32 (t, *J* = 7.3, 1H), 7.33 (d, *J* = 7.8, 1H), 7.45 (bd, *J* = 8.6, 1H), 7.50 (td, *J* = 7.5, 1.3, 1H), 7.70 (dd, *J* = 7.1, 1.3, 1H), 7.74 – 7.79 (m, 2H), 7.85 (d, *J* = 8.6, 1H), 7.94 (d, *J* = 8.3, 2H), 7.96 (d, *J* = 8.2, 1H), 8.04 (d, *J* = 7.6, 1H), 8.57 (d, *J* = 8.6, 1H), 9.24 (d, *J* = 8.9, 1H).

^{13}C NMR (101 MHz, $CDCl_3$): 121.8, 122.6, 123.5, 123.9, 125.3, 125.4, 125.8, 126.1, 126.15, 126.16, 127.5, 127.7, 127.74, 127.76, 127.9, 128.3, 128.5, 128.6, 129.0, 129.73, 129.74, 129.8, 130.17, 130.20, 130.4, 131.9, 132.1, 132.3, 134.3, 135.7, 143.2, 144.1, 195.6.

IR ($CHCl_3$): 3083 w, sh, 3051 w, 2955 w, 2928 w, 2855 w, 1700 vs, 1620 w, 1606 w, 1589 w, 1567 w, 1555 w, 1514 w, 1498 w, 1487 w, 1465 m, 1426 w, 1421 m, 1381 m, 1340 w, 1287 w, 1252 w, 1197 w, 1174 w, 1159 w, 1090 m, 1035 vw, 1015 w, 958 m, 927 w, 868 w, 837 w, 831 m, 816 m, 651 w, 618 w, 520 m, 477 vw cm^{-1}.

APCI MS: 431 ($[M+H]^+$).

HR APCI MS: calcd for $C_{33}H_{19}O$ 431.1430, found 431.1425.

Synthesis of compounds 4A and 4F

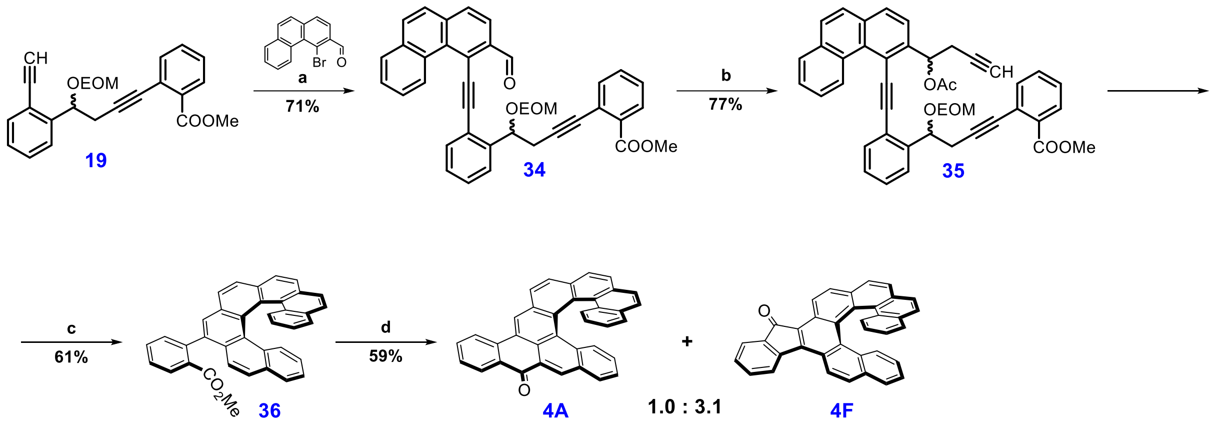

(a) 4-Bromophenanthrene-3-carbaldehyde (1.0 equiv.), diyne 19 (1.1 equiv.), $Pd(PPh_3)Cl_2$ (2 mol%), CuI (4 mol%), THF:Et_3N (2:1), 50 °C, 3 h;

(b) Zn (2.0 equiv.), propargyl bromide (2.0 equiv.), compound 34 (1.0 equiv.), THF, rt, 15 min, then Et_3N (3.0 equiv.), Ac_2O (3.0 equiv.), THF, rt, 4 h;

(c) triyne 35 (1.0 equiv.), CpCo(CO)(fum) (0.7 equiv.), PhCl, 180 °C, 20 min, then *p*-TsOH.H_2O (5.0 equiv.), 95 °C, 1h.

(d) ester 36 (1.0 equiv.), KOH (10.0 equiv.), THF:MeOH (1:1), 65 °C, 5 h, then $MeSO_3H$, 80°C, 2h.

Methyl 2-[4-(ethoxymethoxy)-4-{2-[(3-formylphenanthren-4-yl)ethynyl]phenyl}but-1-yn-1-yl]benzoate 34

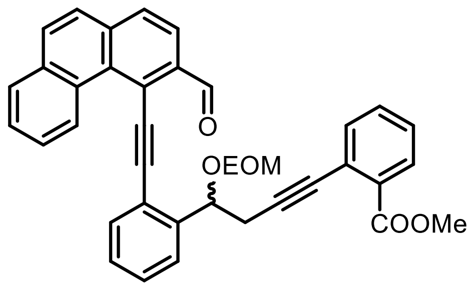

A Schlenk flask was charged with 4-bromophenanthrene-3-carbaldehyde (356 mg, 1.25 mmol), bis(triphenylphosphine)palladium chloride (17.5 mg, 0.03 mmol, 2 mol%), copper(I) iodide (9.52 mg, 0.05 mmol, 4 mol%) and flushed with argon. The

degassed tetrahydrofuran (10 mL) and degassed triethylamine (10 mL) were added and the mixture was heated to 50 °C. Then diyne **19** (500 mg, 1.38 mmol, 1.1 equiv.) in degassed tetrahydrofuran (10 mL) was slowly added and the reaction was stirred at 50 °C for 3 hours. The solvents were evaporated under the reduced pressure. The residue was chromatographed on silica gel (hexane-ethyl acetate 20:1 to 10:1) to afford the desired product **34** (502 mg, 71%).

^{1}H NMR (400 MHz, $CDCl_3$): 1.05 (t, *J* = 7.1, 3H), 3.09 (dd, *J* = 17.0, 6.2, 1H), 3.15 (dd, *J* = 17.0, 6.7, 1H), 3.51 (dq, *J* = 9.5, 7.1, 1H), 3.73 (s, 3H), 3.72 – 3.80 (m, 1H), 4.71 (d, *J* = 7.0, 1H), 4.83 (d, *J* = 7.0, 1H), 5.56 (t, *J* = 6.4, 1H), 7.18 – 7.27 (m, 2H), 7.31 – 7.35 (m, 1H), 7.41 (td, *J* = 7.5, 1.4, 1H), 7.50 (td, *J* = 7.6, 1.4, 1H), 7.66 (ddd, *J* = 8.0, 7.0, 1.2, 1H), 7.69 – 7.80 (m, 5H), 7.88 (d, *J* = 8.6, 1H), 7.90 – 7.95 (m, 2H), 8.14 (d, *J* = 8.2, 1H), 10.29 – 10.34 (m, 1H), 11.13 (d, *J* = 0.9, 1H).

^{13}C NMR (101 MHz, $CDCl_3$): 15.0, 28.8, 52.0, 63.8, 74.8, 81.4, 91.5, 92.2, 93.6, 101.3, 121.6, 124.0, 124.06, 124.07, 126.7, 126.9, 127.0, 127.3, 127.4, 127.7, 128.2, 128.9, 129.7, 129.8, 130.1, 130.4, 130.99, 131.01, 131.5, 131.9, 132.7, 133.5, 134.5, 136.5, 136.7, 143.2, 166.7, 192.9.

IR ($CHCl_3$): 3066 w, 2979 w, 2953 w, 2933 w, 2885 w, 2884 w, sh, 2230 vw, 2193 w, 1727 s, 1686 vs, 1588 m, 1568 w, 1486 m, 1467 w, 1448 m, 1435 m, 1395 w, sh, 1297 m, 1278 m, 1256 vs, 1232 m, 1164 w, 1148 w, 1131 m, 1111 m, 1099 m, 1044 m, 1018 m, 964 w, 869 w, 849 m, 701 w, 637 w, 521 w cm^{-1}.

ESI MS: 589 ($[M+Na]^+$).

HR APCI MS: calcd for $C_{38}H_{30}O_5Na$ 589.1986, found 589.1984.

Methyl 2-{4-[2-({3-[1-(acetyloxy)but-3-yn-1-yl]phenanthren-4-yl}ethynyl)phenyl]-4-(ethoxymethoxy)but-1-yn-1-yl}benzoate 35

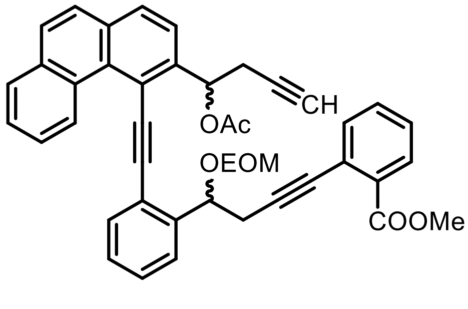

A Schlenk flask was filled with zinc powder (106 mg, 1.62 mmol, 2 equiv.) and flushed with argon. Freshly distilled tetrahydrofuran (4 mL) was added and vigorously stirred. Then propargyl bromide (80 wt. % in toluene, 180 µL, 1.62 mmol, 2 equiv.) was added and stirred for 10 min, before it was transferred by a syringe to the second Schlenk flask, which contained aldehyde **34** (461 mg, 0.81 mmol) and

tetrahydrofuran (20 mL). The reaction mixture was stirred for 5 min at room temperature, before triethylamine (331 μL, 2.43 mmol, 3 equiv.) and acetic anhydride (230 μL, 2.43 mmol, 3 equiv.) were added and the solution was stirred for 4 h at room temperature. The solvents were evaporated under the reduced pressure and the residue was purified by flash chromatography on silica gel (hexane-ethyl acetate 10:1) to obtain acetate **35** (405 mg, 77%) as a mixture of diastereomers in a ratio about 1:1.

^{1}H NMR (400 MHz, $CDCl_3$, mixture of diastereomers): 0.98 (t, *J* = 7.1, 3H), 1.02 (t, *J* = 7.1, 3H), 2.02 (t, *J* = 2.6, 1H), 2.03 (t, *J* = 2.6, 1H), 2.17 (s, 3H), 2.18 (s, 3H), 2.95 (dt, *J* = 17.1, 6.5, 2H), 3.08 (dt, *J* = 17.1, 5.1, 2H), 3.16 – 3.21 (m, 4H), 3.48 (dq, *J* = 9.4, 7.0, 1H), 3.51 (dq, *J* = 9.4, 7.0, 1H), 3.66 – 3.75 (m, 2H), 3.73 (s, 3H), 3.75 (s, 3H), 4.76 (d, *J* = 6.8, 1H), 4.77 (d, *J* = 6.9, 1H), 4.85 (d, *J* = 6.8, 1H), 4.89 (d, *J* = 6.8, 1H), 5.63 (t, *J* = 6.1, 1H), 5.64 (t, *J* = 6.1, 1H), 6.96 (t, *J* = 6.1, 1H), 6.97 (t, *J* = 6.1, 1H), 7.19 – 7.29 (m, 4H), 7.30 – 7.36 (m, 2H), 7.397 (td, *J* = 7.5, 1.5, 1H), 7.400 (td, *J* = 7.5, 1.5, 1H), 7.44 – 7.49 (m, 2H), 7.59 (ddd, *J* = 8.0, 7.0, 1.1, 1H), 7.60 (ddd, *J* = 8.0, 7.0, 1.1, 1H), 7.69 (d, *J* = 8.8, 2H), 7.71 – 7.85 (m, 12H), 7.88 (dt, *J* = 7.9, 1.6, 2H), 7.91 (bd, *J* = 8.3, 2H), 10.34 – 10.38 (m, 2H).

^{13}C NMR (101 MHz, $CDCl_3$, mixture of diastereomers): 14.98, 15.03, 21.2 (2C), 25.96, 26.04, 28.8, 28.9, 52.0, 52.1, 63.77, 63.82, 71.17, 71.20, 71.9, 72.0, 74.9, 75.0, 79.7 (2C), 81.2, 81.3, 92.0, 92.1, 93.6, 93.8, 94.0, 94.1, 99.7, 99.9, 117.0 (2C), 121.8 (2C), 121.9 (2C), 123.7, 123.8, 124.22, 124.23, 126.32, 126.34, 126.67, 126.71, 127.08 (2C), 127.13 (2C), 127.2 (2C), 127.30, 127.32, 128.03, 128.04, 128.4 (2C), 128.6 (2C), 129.1, 129.2, 129.7, 129.8, 130.12, 130.14, 130.8 (2C), 131.4, 131.5, 131.9, 132.0, 132.7 (2C), 132.8, 132.9, 133.44 (2C), 134.46, 134.50, 142.3, 142.4, 142.9 (2C), 166.87 (2C), 169.88, 169.91.

IR ($CHCl_3$): 3309 m, 3054 w, 2979 w, 2952 w, 2933 w, 2886 w, 2845 w, 2232 w, 2192 vw, 2124 vw, 1732 vs, 1625 vw, 1597 w, 1568 w, 1486 m, 1448 m, 1435 m, 1373 m, 1297 m, 1278 m, 1253 s, 1238 vs, 1163 w, 1147 w, 1131 m, 1111 m, 1099 m, 1044 vs, 1022 s, 965 w, 868 w, 845 m, 701 w, 640 w, 602 w cm^{-1}.

ESI MS: 671 ($[M+Na]^+$).

HR ESI MS: calculated for $C_{43}H_{36}O_6Na$ 671.2404, found 671.2402.

Methyl 2-heptahelicen-7-ylbenzoate 36

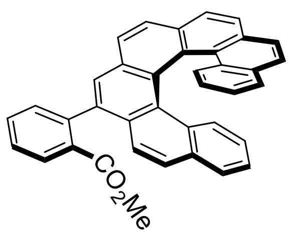

Triyne **35** (364 mg, 0.56 mmol) was loaded to a microwave vial and chlorobenzene (12 mL) was added. The solution was bubbled with nitrogen for 5 minutes before CpCo(CO)(fum) (109 mg, 0.39 mmol, 0.7 equiv, fum = dimethylfumarate) was added. The seal was closed and the reaction mixture was heated to 180 °C in the microwave reactor for 20 minutes. *p*-Toluenesulfonic acid monohydrate (532 mg, 2.80 mmol, 5 equiv.) was added and the reaction mixture was strirred for 1 h at 95 °C. The solvent was evaporated in vacuum. The residue was purified by flash chromatography on silica gel (hexane-ethyl acetate 20:1) to obtain helicene **36** (175 mg, 61%) as a mixture of two atropodiastereomers in a ratio 3.7:1.

^{1}H NMR (400 MHz, $CDCl_3$, major atropodiastereomer): 3.71 (s, 3H), 6.42 (ddd, *J* = 8.4, 6.9, 1.4, 1H), 6.54 (ddd, *J* = 8.4, 6.9, 1.5, 1H), 6.90 (ddd, *J* = 8.0, 6.9, 1.2, 1H), 6.92 (ddd, *J* = 8.2, 6.9, 1.2, 1H), 7.21 (dd, *J* = 8.5, 1.1, 1H), 7.25 – 7.29 (m, 1H), 7.32 (dd, *J* = 8.0, 1.4, 1H), 7.36 (d, *J* = 8.8, 1H), 7.38 (d, *J* = 8.7, 1H), 7.48 – 7.53 (m, 2H), 7.53 (d, *J* = 8.7, 1H), 7.61 (td, *J* = 7.6, 1.5, 1H), 7.68 (td, *J* = 7.5, 1.5, 1H), 7.83 (s, 1H), 7.94 (d, *J* = 8.2, 1H), 7.99 – 8.04 (m, 3H), 8.05 (d, *J* = 8.2, 1H), 8.23 (dd, *J* = 7.8, 1.5, 1H).

^{13}C NMR (101 MHz, $CDCl_3$, major atropodiastereomer): 52.1, 123.2, 123.8, 123.9, 124.8, 124.9, 125.01, 125.04, 125.1, 125.6, 125.8, 126.0, 126.5, 126.7, 126.8, 127.0, 127.2 (2C), 127.4, 127.5, 128.0, 128.3, 128.4, 129.5, 129.6, 130.0, 130.5, 131.0, 131.30, 131.34, 131.5, 131.8, 132.0, 132.2 (2C), 139.6, 141.9, 167.6.

IR ($CHCl_3$): 3053 w, 2953 w, 2928 w, 2855 w, 2844 w, sh, 1723 vs, 1617 vw, 1599 w, 1571 w, 1541 w, 1497 w, 1488 w, 1470 w, sh, 1447 w, 1434 m, 1417 vw, 1359 w, sh, 1294 s, 1275 m, 1260 s, 1238 w, 1163 w, 1133 m, 1091 m, 1073 w, 1044 w, 1035 w, 964 w, 889 w, 881 w, 838 vs, 825 m, 688 w, 648 w, 613 m, 604 w, 588 w, 566 w, 534 w, 525 w, 472 vw cm^{-1}.

ESI MS: 535 ($[M+Na]^+$).

HR ESI MS: calcd for $C_{38}H_{24}O_2Na$ 535.1669, found 535.1670.

14*H*-Naphtho[3,2,1-*gh*]heptahelicen-14-one 4A

15*H*-Indeno[1,2-*i*]heptahelicen-15-one 4F

Ester **36** (152 mg, 0.30 mmol) was dissolved in tetrahydrofuran (4 mL) and methanol (4 mL), and potassium hydroxide (168 mg, 3.00 mmol, 10 equiv.) was added. The mixture was heated

to 65 °C and stirred for 5 h. Then the reaction was cooled to rt and diluted hydrochloric acid was added to reach pH 1. The mixture was extracted with diethyl ether (2x 20 mL), and the combined organic layers were dried over anhydrous magnesium sulfate. The solvents were evaporated under the reduced pressure to obtain crude carboxylic acid, which was directly used further. This acid was dissolved in methanesulfonic acid (10 mL) and the flask was put to the oil bath pre-heated to 80 °C. The stirring was continued for 2 h at 80 °C and then the reaction mixture was poured over crushed ice. The mixture was extracted with dichloromethane (2x 20 mL), the combined organic layers were washed with saturated sodium bicarbonate solution (10 mL), dried over anhydrous magnesium sulfate. The solvent was evaporated *in vacuo* and the residue was purified by flash chromatography on silica gel (hexane-dichloromethane 2:1 to 1:3) to afford **4A** (20.8 mg, 14%) as an orange solid and **4F** (64.4 mg, 45%) as a red solid.

14*H*-Naphtho[3,2,1-*gh*]heptahelicen-14-one 4A

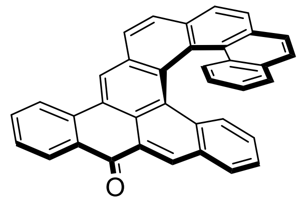

M.p.: 125 - 126 °C (CH_3CN).

Optical rotation: $[\alpha]^{20}_D$ +3493° (c 0.024, CH_2Cl_2); $[\alpha]^{20}_D$ -3526° (c 0.021, CH_2Cl_2).

^{1}H NMR (400 MHz, $CDCl_3$): 6.34 (ddd, *J* = 8.4, 6.8, 1.4, 1H), 6.55 (ddd, *J* = 8.4, 6.9, 1.4, 1H), 6.89 (ddd, *J* = 8.0, 6.8, 1.2, 1H), 7.00 (ddd, *J* = 8.0, 6.8, 1.2, 1H), 7.14 (bd, *J* = 8.4, 1H), 7.23 (bd, *J* = 8.6, 1H), 7.30 (dd, *J* = 8.0, 1.3, 1H), 7.50 (d, *J* = 8.5, 1H), 7.57 (dd, *J* = 8.1, 1.3, 1H), 7.63 (td, *J* = 7.5, 1.1, 1H), 7.72 (d, *J* = 8.5, 1H), 7.85 (ddd, *J* = 8.4, 7.1, 1.5, 1H), 7.95 (d, *J* = 8.2, 1H), 8.00 (d, *J* = 8.3, 1H), 8.07 (d, *J* = 8.2, 1H), 8.12 (d, *J* = 8.2, 1H), 8.59 (t, *J* = 7.0, 1H), 8.60 (d, *J* = 7.8, 1H), 8.82 (s, 1H), 8.86 (s, 1H).

^{13}C NMR (101 MHz, $CDCl_3$): 123.9, 124.2, 124.3, 124.4, 124.7, 125.4 (3C), 125.6, 125.7, 126.0 (2C), 126.2, 126.6, 126.9, 127.0, 127.3, 127.8, 128.0, 128.39, 128.42, 128.5 (2C), 128.8, 129.1, 129.2, 130.8, 131.1, 131.3, 131.8, 131.9, 132.0, 132.1, 132.7, 133.9, 136.8, 184.5.

IR ($CHCl_3$): 3053 w, 3027 w, 2957 w, 2928 vs, 2855 m, 1653 vs, 1610 m, 1601 m, 1583 m, 1554 w, 1522 w, 1498 w, 1486 w, 1465 vw, 1441 w, 1395 w, 1379 w, 1365 vw, 1330 w, 1280 m, 1265 m, 1261 m, 1249 w, 1172 w, 1148 w, 1117 w, 1083 w, sh, 1031 w, 982 w, 963 w, 883 w, 839 m, 829 w, 812 w, 700 w, 621 w, 609 m, 565 w, 541 w, 516 w, 472 w cm^{-1}.

APCI MS: 481 ($[M+H]^+$).

HR APCI MS: calcd for $C_{37}H_{21}O$ 481.1587, found 481.1585.

15*H*-Indeno[1,2-*i*]heptahelicen-15-one **4F**

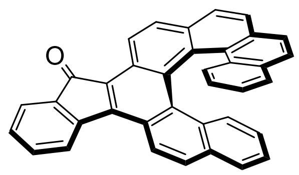

M.p.: 304 – 305 °C (CH_3CN).

^{1}H NMR (600 MHz, CD_2Cl_2): 6.43 (ddd, *J* = 8.3, 6.8, 1.4, 1H), 6.51 (ddd, *J* = 8.3, 6.8, 1.4, 1H), 6.959 (ddd, *J* = 8.0, 6.9, 1.2, 1H), 6.963 (ddd, *J* = 8.0, 6.9, 1.1, 1H), 7.09 (dq, *J* = 8.4, 0.8, 1H), 7.31 (ddt, *J* = 8.1, 1.2, 0.6, 1H), 7.32 (ddt, *J* = 8.1, 1.3, 0.6, 1H), 7.34 (dq, *J* = 8.5, 0.9, 1H), 7.41 (ddd, *J* = 7.7, 7.1, 0.8, 1H), 7.47 (dt, *J* = 8.4, 0.7, 1H), 7.60 (dt, *J* = 8.8, 0.7, 1H), 7.62 (td, *J* = 7.5, 1.3, 1H), 7.71 (d, *J* = 8.5, 1H), 7.75 (ddd, *J* = 7.1, 1.3, 0.6, 1H), 7.94 (dd, *J* = 8.1, 0.5, 1H), 8.00 (dd, *J* = 8.1, 0.5, 1H), 8.13 (dd, *J* = 8.4, 0.5, 1H), 8.21 (dq, *J* = 7.4, 0.7, 1H), 8.47 (d, *J* = 8.9, 1H), 9.35 (d, *J* = 8.4, 1H).

^{13}C NMR (151 MHz, CD_2Cl_2): 121.6, 122.5, 124.0, 124.1, 124.48, 124.50, 124.7, 125.4, 126.0, 126.1, 126.2, 126.3, 126.6, 126.8, 126.9, 127.0, 127.2, 127.3, 127.8, 128.37, 128.42, 128.43, 128.8, 129.3, 129.73, 129.75, 129.9, 131.7, 132.2, 132.4, 132.8, 134.7, 135.2, 136.2, 144.2, 144.4, 195.8.

IR ($CHCl_3$): 3051 w, 2956 w, 2928 w, 2856 w, 1702 vs, 1614 w, 1608 w, 1583 w, 1568 w, 1551 w, 1516 w, 1502 w, 1475 w, 1464 m, 1396 w, 1337 w, 1278 w, 1234 m, 1195 w, 1173 w, 1158 w, 1088 w, 1009 w, 994 w, 959 vw, 900 w, 884 vw, 846 s, 828 m, 806 w, 662 w, 648 w, 617 m, 565 w, 524 w, 471 vw cm^{-1}.

APCI MS: 481 ($[M+H]^+$).

HR APCI MS: calcd for $C_{37}H_{21}O$ 481.1587, found 481.1582.

Synthesis of compounds 5A and 5F

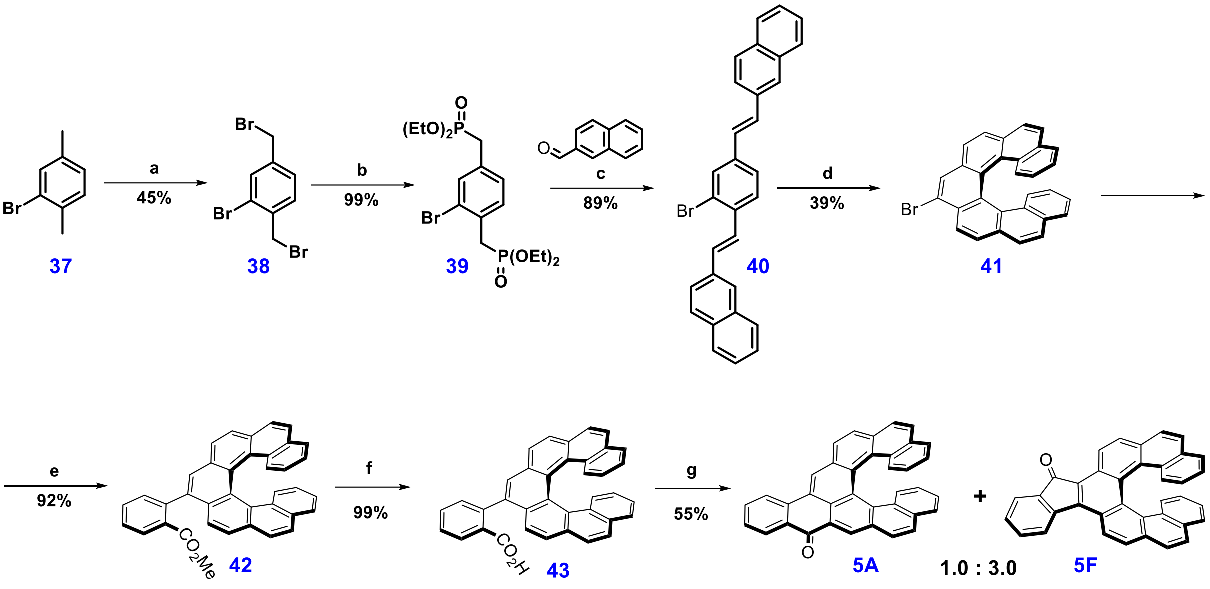

(a) 2-Bromo-1,4-dimethylbenzene 37 (1.0 equiv.), NBS (2.02 equiv.), AIBN (cat.), $CHCl_3$, 62 °C, 4 h;
(b) compound 38 (1.0 equiv.), $P(OEt)_3$ (2.01 equiv.), 120°C, 14 h;
(c) compound 39 (1.0 equiv.), 2-naphthaldehyde (2.77 equiv.), NaH (4.19 equiv.), PhMe, 120 °C, 30 min;
(d) stilbene 40 (1.0 equiv.), I_2 (0.17 equiv.), PhMe:EtOAc (19:1), rt, 40 h;
(e) helicene 41 (1.0 equiv.), 2-methoxycarbonylphenylboronic acid (1.5 equiv.), Pd_2dba_3 (2 mol%), Xphos (4 mol%), $K_3PO_4.H_2O$ (6.0 equiv.), PhMe, 90°C, 14 h;
(f) ester 42 (1.0 equiv.), NaOH (5.0 equiv.), THF:MeOH:H_2O (8:2:1), 70 °C, 16 h;
(g) $MeSO_3H$, 80°C, 30 min.

<u>2-Bromo-1,4-bis(bromoethyl)benzene</u> 38

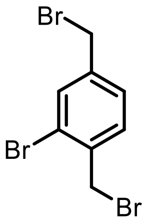

2-Bromo-1,4-dimethylbenzene **37** (6.0 mL, 44 mmol), NBS (15.82 g, 88.9 mmol, 2.02 eq.) and AIBN (60 mg, 0.365 mmol, 0.08 equiv.) were charged in a round bottom flask. Chloroform was added (200 mL) and the reaction was refluxed under inert for 4 h. The whole reaction mixture was filtered through a short silica gel pad (eluent chloroform) to remove polar succinimide. Organic solvent was removed *in vacuo* and the residue was adsorbed on silica-gel and extracted by continuous extraction by hot pentane through Thiele-Pappe attachment. After 5 hours of extraction the product **38** as a white precipitate (6.8 g, 45%) was collected by filtration over a glass frit S3. ^{1}H NMR spectrum is in agreement with the published data.[11]

^{1}H NMR (400 MHz, $CDCl_3$): 4.41 (s, 2H), 4.58 (s, 2H), 7.32 (dd, *J* = 7.9, 1.8, 1H), 7.43 (d, *J* = 7.9, 1H), 7.61 (d, *J* = 1.8, 1H).

---

[11] L. Pan, K-M. Lee, Y-Y. Chan, Z. Ke, Y-Y. Yeung *Org. Lett.* **2023**, *25*, 53.

Tetraethyl [(2-bromobenzene-1,4-diyl)dimethanediyl]bis(phosphonate) 39

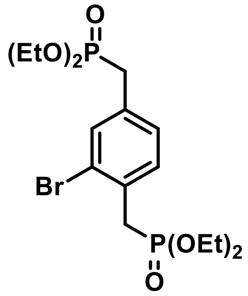

2-Bromo-1,4-bis(bromoethyl)benzene **38** (6.86 g, 20 mmol) and the triethylphosphite (6.9 mL, 40.2 mmol, 2.01 equiv.) were added in well dry round bottom flask and stirred overnight at 120°C under inert. The excess of triethylphosphite was removed *in vacuo* by heating the reaction mixture at 70°C for 4 h. The product **39** as a colorless oil (8.74 g, 99%) was used directly in the next step without any further purification. ^{1}H NMR spectrum is in agreement with the published data.[12]

^{1}H NMR (400 MHz, $CDCl_3$): 1.22 – 1.27 (m, 12H), 3.04 – 3.11 (m, 2H), 3.33 – 3.40 (m, 2H), 3.98 – 4.08 (m, 8H), 7.19 - 7.22 (m, 1H), 7.37 – 7.41 (m, 1H), 7.49 (bs, 1H).

2,2'-[(2-Bromobenzene-1,4-diyl)di(*E*)ethene-2,1-diyl]dinaphthalene 40

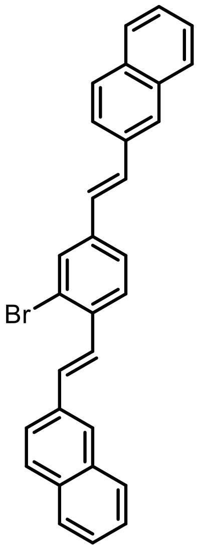

In a well dry round bottom flask were dissolved previous bisphosphate **39** (8.74 g, 19.1 mmol) and 2-naphthaldehyde (8.27 g, 53 mmol, 2.77 equiv.) in dry toluene (350 mL). To this mixture was added NaH (60%, dispersion in mineral oil, 3.2 g, 80 mmol, 4.19 equiv.) and the mixture was heated at 120 °C for 30 min under inert. The reaction mixture was cooled to room temperature and then slowly poured into a mixture of ice-water-37 % HCl (100 g -100 ml-100 ml). The organic phase with partially precipitated yellow product was separated and the water phase was extracted with toluene (3 x 100 mL). The organic portions were combined and the volume was reduced to its 20 % on rotavap. The product **40** as a yellow precipitate (7.8 g, 89%) was collected by filtration over a glass frit S3. ^{1}H NMR spectrum is in agreement with the published data.[13]

^{1}H NMR (400 MHz, CD_2Cl_2): 7.21 (d, J = 16.3, 1H), 7.29 – 7.37 (m, 2H), 7.46 – 7.53 (m, 4H), 7.58 (dd, J = 8.0, 1.8, 1H), 7.64 (d, J = 16.3, 1H) 7.76 - 7.90 (m, 12H).

^{13}C NMR (101 MHz, CD_2Cl_2): 123.9, 124.1, 125.1, 126.3, 126.76, 126.77, 127.0 (2C), 127.2, 127.5, 127.6, 127.7 (2C), 128.2 (2C), 128.60, 128.61, 129.0 (2C), 130.6, 131.5, 131.8, 133.8, 133.9, 134.2 (2C), 135.0, 135.1, 136.5, 139.0.

---

[12] Z.-P. Zhuang, M.-P. Kung, C. Hou, D. M. Skovronsky, T. L. Gur, K. Plössl, J. Q. Trojanowski, V. M.-Y. Lee, H. F. Kung, *J. Med. Chem.* **2001**, *44*, 1905.
[13] L Liu, B. Yang, T. J. Katz, M. K. Poindexter *J. Org. Chem.* **1991**, *56*, 3769.

__9-Bromoheptahelicene__ **41**

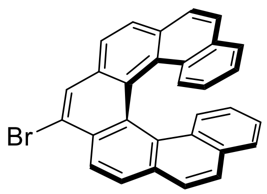

The previous stilbene **40** (3.2 g, 6.94 mmol) was suspended in toluene (400 mL). This mixture was heated to fully dissolve the starting material. When all the solid was dissolved, the mixture was diluted with toluene (1500 mL) and ethyl acetate (100 mL). Iodine (0.3 g, 1.18 mmol) was added to the mixture and the reaction mixture was irradiated with medium-pressure mercury (150 W) lamp for 40 h open to air. The whole reaction mixture was evaporated, and the residue was adsorbed on silica-gel. The product was isolated by continuous extraction with refluxing cyclohexane using Thiele-Pappe attachment. After evaporation of cyclohexane, the residue was triturated in diethyl ether and filtered over glass frit S4 to get slightly yellow product **41** (1.25 g, 39%). ^{1}H NMR spectrum is in agreement with the published data.[14]

^{1}H NMR (400 MHz, $CDCl_3$): 6.40 – 6.44 (m, 2H), 6.89 – 6.94 (m, 2H), 7.04 (d, *J* = 2.7, 1H), 7.06 (d, *J* = 2.9, 1H), 7.30 (d, *J* = 7.9, 2H), 7.48 – 7.52 (m, 2H), 7.69 – 7.52 (m, 2H), 7.90 (d, *J* = 8.2, 2H), 8.01 (d, *J* = 8.5, 1H), 8.34 (s, 1H), 8.45 (d, *J* = 8.5, 1H).

__Methyl 2-heptahelicen-9-ylbenzoate__ **42**

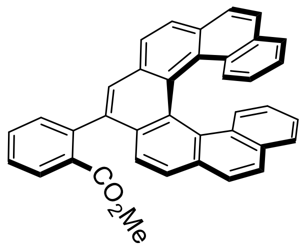

In a dry Schlenk flask was dissolved 9-bromo[7]helicene **41** (100 mg, 0.22 mmol), 2-methoxycarbonylphenylboronic acid (60 mg, 0.33 mmol, 1.5 equiv.), Pd_2dba_3 (4 mg, 4.4 µmol, 2 mol%), Xphos (4.2 mg, 8.8 µmol, 4 mol%) and $K_3PO_4.H_2O$ (304 mg, 1.32 mmol, 6 equiv.) in dry toluene (6 mL). The reaction mixture was thoroughly degassed and then heated at 90 °C overnight. The solvent was evaporated *in vacuo* and the residue was purified by column chromatography on silca gel (cyclohexane- ethyl acetate 20:1). The product **42** was obtained as a yellow solid (104 mg, 92%). The ^{1}H NMR spectrum shows a mixture of two atropodiastereomers (1:1.7).

^{1}H NMR (400 MHz, CD_2Cl_2): 3.24 and 3.35 (s, in ratio 1:1.7, 3H), 6.36 – 6.44 (m, 2H), 6.87 – 6.93 (m, 2H), 7.08 – 7.11 (m, 1H), 7.14 – 7.20 (m, 1H), 7.28 – 7.32 (m, 2H), 7.47 – 7.52 (m, 2H), 7.59 – 7.64 (m, 3H), 7.69 – 7.77 (m, 3H), 7.79 – 7.82 (m, 1H), 7.85 – 7.87 (m, 1H), 7.94 – 8.01 (m, 2H), 8.11 and 8.17 (m, in ratio 1:1.7, 1H).

[14] L Liu, B. Yang, T. J. Katz, M. K. Poindexter *J. Org. Chem.* **1991**, *56*, 3769.

^{13}C NMR: Not recorded due to mixture of atropoisomers.

ESI MS: 535 ($[M+Na]^+$).

HR ESI MS: calcd for $C_{38}H_{24}O_2Na$ 535.1669; found 535.1665.

2-Heptahelicen-9-ylbenzoic acid 43

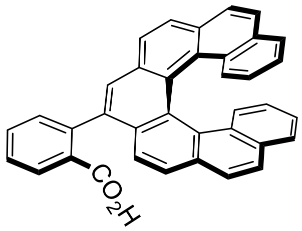

The previous compound **42** (104 mg, 0.203 mmol) and sodium hydroxide (41 mg, 1.01 mmol, 5 equiv.) was dissolved in a mixture of solvents of THF-methanol-water (4:1:0.5 mL). The mixture was stirred overnight at 70 °C under inert. Then the reaction was acidified by diluted hydrochloric acid and the mixture was extracted with dichloromethane (4 x 10 mL). The combined organic portions were dried over $MgSO_4$. After filtration and evaporation of all the volatiles was obtained product **43** as an amorphous yellow solid (101 mg, yield 99%).

^{1}H NMR (400 MHz, tetrachloroethane-*d*$_2$, 110°C): 6.39 – 6.46 (m, 2H), 6.88 – 6.95 (m, 2H), 7.12 – 7.20 (m, 2H), 7.29 – 7.32 (m, 2H), 7.47 – 7.52 (m, 2H), 7.57 – 7.80 (m, 7H), 7.85 – 7.87 (m, 1H), 7.94 (s, 2H), 8.12 and 8.16 (d, in ratio 1:1.3, 1H).

^{13}C NMR: Not recorded due to mixture of atropoisomers.

ESI MS: 497 ($[M-H]^-$).

HR ESI MS: calcd for $C_{37}H_{21}O_2$ 497.1547; found 497.1544.

14*H*-Naphtho[3,2,1-*jk*]heptahelicen-14-one 5A

19*H*-Indeno[1,2-*l*]heptahelicen-19-one 5F

The previous acid **43** (320 mg, 0.64 mmol) was suspended in methanesulfonic acid (12.4 mL) and the reaction was stirred at 80 °C for 30 min. Then, the reaction was poured in water and neutralized by potassium carbonate solution. The mixture was extracted with dichloromethane (4 x 20 mL). The organic phase was dried over $MgSO_4$, filtered over the glass frit (S3) and evaporated *in vacuo*. The residue was chromatographed on silica gel (toluene). The first isolated product corresponds to the fluoreno-derivative **5F** and is red crystalline solid (126.8 mg, 41%) the second corresponds to the aceno-derivative **5A** and is an orange solid (42.3 mg, 14%). The combined yield (169 mg) is 55%.

<u>14*H*-Naphtho[3,2,1-*jk*]heptahelicen-14-one</u> 5A

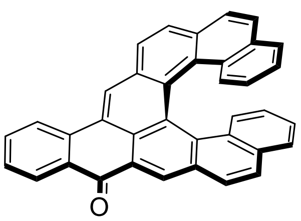

M.p.: 299 - 300 °C (CH_3CN).

Optical rotation: $[\alpha]^{20}_D$ +3620° (c 0.026, CH_2Cl_2); $[\alpha]^{20}_D$ -3672° (c 0.025, CH_2Cl_2).

^{1}H NMR (400 MHz, $CDCl_3$): 6.44 – 6.51 (m, 2H), 6.93 – 6.99 (m, 2H), 7.21 (d, *J* = 8.5, 1H), 7.23 (d, *J* = 8.5, 1H), 7.31 (d, *J* = 3.8, 1H), 7.33 (d, *J* = 4.2, 1H), 7.51 (d, *J* = 8.5, 1H), 7.56 (d, *J* = 8.5, 1H), 7.61 – 7.64 (m, 1H), 7.72 (d, *J* = 8.5, 1H), 7.85 (ddd, *J* = 8.4, 7.1, 1.5, 1H), 7.92 (d, *J* = 8.5, 1H), 7.98 (d, *J* = 8.2, 1H), 8.11 (d, *J* = 8.3, 1H), 8.58 – 8.62 (m, 2H), 8.91 (s, 1H), 9.19 (s, 1H).

^{13}C NMR (101 MHz, $CDCl_3$): 123.8, 124.1, 124.2, 124.3, 124.5, 124.9, 125.5, 125.77, 125.79, 125.9, 126.3, 126.46, 126.52, 126.6, 126.7, 126.9, 127.0, 127.3, 128.2 (2C), 128.3, 128.4, 128.47, 128.49, 129.2, 129.7, 130.3, 130.98, 131.03, 131.8, 131.9, 132.0, 132.3, 133.2, 133.8, 136.7, 184.2.

IR ($CHCl_3$): 3054 w, 2928 w, 2853 w, 1651 vs, 1602 s, 1585 vs, 1574 s, 1554 w, 1499 w, 1474 m, 1451 w, 1425 w, 1372 s, 1333 w, 1300 m, 1286 m, 1277 m, 1176 w, 1139 w, 1109 w, 1016 m, 952 w, 922 w, 885 w, 867 w, 833 s, 813 m, 698 w, 650 m, 642 m, 607 w, 522 w cm^{-1}.

ESI MS: 481 ($[M+H]^+$).

HR ESI MS: calcd for $C_{37}H_{21}O$ 481.1587; found 481.1582.

<u>19*H*-Indeno[1,2-*l*]heptahelicen-19-one</u> 5F

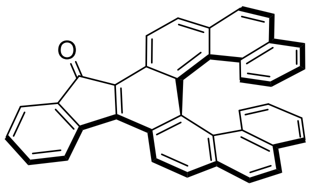

M.p.: 307 - 308 °C (CH_3CN).

^{1}H NMR (400 MHz, $CDCl_3$): 6.44 – 6.49 (m, 2H), 6.90 – 6.95 (m, 2H), 7.02 (d, *J* = 13.6, 1H), 7.05 (d, *J* = 13.6, 1H), 7.27 (td, *J* = 1.5, 0.7, 1H), 7.29 (td, *J* = 1.4, 0.7, 1H), 7.37 (ddd, *J* = 7.8, 7.1, 0.8, 1H), 7.44 (d, *J* = 8.4, 1H), 7.51 (d, *J* = 8.4, 1H), 7.58 (td, *J* = 7.6, 1.3, 1H), 7.67 (d, *J* = 14.0, 1H), 7.69 (d, *J* = 14.0, 1H), 7.76 (ddd, *J* = 7.2, 1.3, 0.6, 1H), 7.99 (d, *J* = 8.4, 1H), 8.01 (d, *J* = 8.4, 1H), 8.18 (d, *J* = 7.5, 1H), 8.69 (d, *J* = 8.6, 1H), 9.33 (d, *J* = 8.5, 1H).

^{13}C NMR (101 MHz, $CDCl_3$): 122.62, 122.63, 123.5, 123.9, 124.2, 124.5, 124.7, 124.9, 125.1, 125.3, 125.4, 125.7, 125.9, 126.8, 126.88, 126.93, 127.4, 127.9, 128.0, 128.5 (2C), 128.9, 129.0, 129.2 (2C), 129.6, 130.0, 131.2, 131.5, 131.9, 132.0, 132.2, 134.3, 135.9, 143.4, 144.2, 195.7.

IR ($CHCl_3$): 3053 w, 2929 w, 2856 w, 1700 vs, 1619 w, 1607 w, 1587 w, 1552 w, 1518 w, 1477 w, 1465 m, 1427 w, 1386 w, 1376 w, 1334 w, 1288 w, 1250 w, 1233 w, 1186 w, 1174 w, 1090 w, 1047 w, 1017 w, 954 w, 905 w, 842 m, 829 s, 656 w, 639 m, 618 m, 527 w, 471 w cm^{-1}.

ESI MS: 481 ([M+H]$^+$).

HR ESI MS: calcd for $C_{37}H_{21}O$ 481.1587; found 481.1589.

Synthesis of compounds 6A and 6F

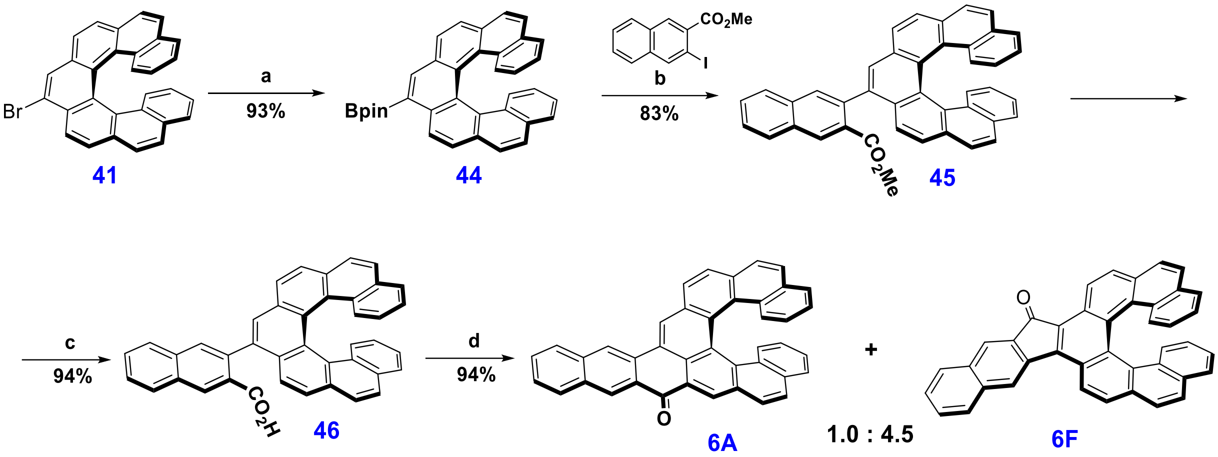

(a) helicene 41 (1.0 equiv.), B_2pin_2 (1.5 equiv.), $PdCl_2$(dppf) (4 mol%), KOAc (3.0 equiv.), dioxane, 70 °C, 24 h;
(b) methyl 3-iodo-2-naphthoate (1.0 equiv.), compound 44 (1.0 equiv.), $Pd(PPh_3)Cl_2$ (4 mol%), K_2CO_3 (1.0 equiv.), PhMe:EtOH:H_2O (4:4:1), 90 °C, 16 h;
(c) ester 45 (1.0 equiv.), NaOH (5.0 equiv.), THF:MeOH:H_2O (19:4.8:2.4), 70 °C, 16 h;
(d) $MeSO_3H$, 80°C, 1h.

<u>2-Heptahelicen-9-yl-4,4,5,5-tetramethyl-1,3,2-dioxaborolane</u> 44

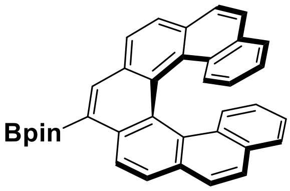

A Schlenk flask was charged with 9-bromo[7]helicene **41** (600 mg, 1.31 mmol), bis(pinacolato)diboron (500 mg, 1.97 mmol, 1.5 equiv.), $PdCl_2$(dppf) (38.3 mg, 52.4 µmol, 4 mol%) and potassium acetate (386 mg, 3.93 mmol, 3 equiv.), flushed with argon and anhydrous dioxane (40 mL) was injected. The reaction mixture was degassed and then it was stirred under inert atmosphere at 70 °C for 1 day. After cooling to rt, the solvent was evaporated under the reduced pressure and the residue was purified by chromatography on silica gel (hexane-ethyl acetate 30:1 to 20:1) to afford product **44** (612 mg, 93%) as a yellow amorphous solid. ^{1}H NMR spectrum is in agreement with the published data.[15]

[15] M. Jakubec, I. Ghosh, J. Storch, B. König *Chem. Eur. J.* **2020**, *26*, 543.

^{1}H NMR (400 MHz, CD_2Cl_2): 1.53 (s, 12H), 6.38 – 6.43 (m, 2H), 6.87 – 6.93 (m, 2H), 7.03 – 7.08 (m, 2H), 7.26 – 7.32 (m, 2H), 7.47 (d, *J* = 6.5, 1H), 7.49 (d, *J* = 6.5, 1H), 7.70 – 7.75 (m, 2H), 7.92 (d, *J* = 8.1, 1H), 7.97 (d, *J* = 8.5, 1H), 8.05 (d, *J* = 8.3, 1H), 8.66 (s, 1H), 8.98 (d, *J* = 8.5, 1H).

Methyl 3-heptahelicen-9-yl)naphthalene-2-carboxylate 45

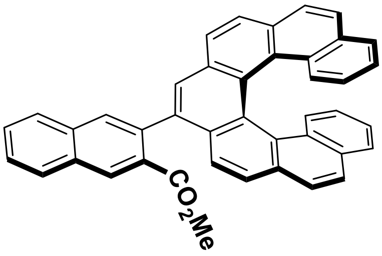

In a dry Schlenk flask was dissolved helicene **44** (474 mg, 0.94 mmol), methyl 3-iodo-2-naphthoate (293 mg, 0.94 mmol, 1.0 equiv.), $PdCl_2(PPh_3)_2$ (26.4 mg, 37.6 µmol, 4 mol%), K_2CO_3 (130 mg, 0.94 mmol, 1 equiv.) and the mixture of solvents of toluene-ethanol-water (25.6 mL, 4:4:1). The reaction mixture was thoroughly degassed and then heated at 90 °C overnight. The solvent was evaporated *in vacuo* and the residue was purified by column chromatography on silca gel (cyclohexane- ethyl acetate 20:1). The product **45** was obtained as a yellow solid (439 mg, 83%). The ^{1}H NMR spectrum shows mixture of two atropodiastereomers (1:1.8).

^{1}H NMR (400 MHz, CD_2Cl_2): 3.17 and 3.34 (s, in ratio 1:1.8, 3H), 6.39 – 6.49 (m, 2H), 6.90 – 6.96 (m, 2H), 7.09 – 7.25 (m, 2H), 7.29 – 7.35 (m, 2H), 7.48 – 7.53 (m, 2H), 7.63 – 7.72 (m, 4H), 7.73 – 7.80 (m, 2H), 7.92 – 8.05 (m, 4H), 8.08 – 8.14 (m, 2H), 8.68 and 8.75 (s, in ratio 1:1.8, 1H).

^{13}C NMR: Not recorded due to mixture of atropoisomers.

FD MS: 562 ($[M]^{+\bullet}$).

HR MS FD: calcd for $C_{42}H_{26}O_2$ 562.1927; found 562.1912.

3-Heptahelicen-9-ylnaphthalene-2-carboxylic acid 46

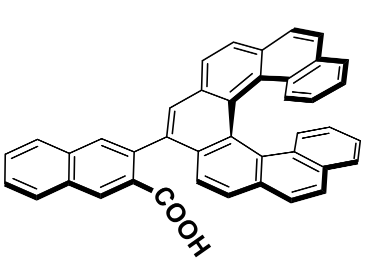

The previous compound **45** (550 mg, 0.977 mmol) and sodium hydroxide (195 mg, 4.88 mmol, 5 equiv.) was dissolved in a mixture of solvents of THF-methanol-water (19:4.8:2.4 mL). The mixture was stirred overnight at 70 °C under inert. Then the reaction was acidified by diluted hydrochloric acid and the mixture was extracted with dichloromethane (4 x 20 mL). The combined organic portions were dried over $MgSO_4$. After filtration and evaporation of all the volatiles was obtained product **46** as an amorphous yellow solid (504 mg, yield 94%).

^{1}H NMR (400 MHz, tetrachloroethane-d_2, 110°C): 6.47 – 6.52 (m, 2H), 6.93 – 7.00 (m, 2H), 7.19 – 7.23 (m, 1H), 7.25 – 7.30 (m, 1H), 7.33 – 7.38 (m, 2H), 7.52 – 7.58 (m, 2H), 7.69 – 7.87 (m, 6H), 7.98 – 8.09 (m, 4H), 8.14 – 8.20 (m, 2H), 8.80 and 8.85 (s, in ratio 1:1.6, 1H).

^{13}C NMR: Not recorded due to mixture of atropoisomers.

FD MS: 548 ([M]$^{+\bullet}$).

HR MS FD: calcd for $C_{41}H_{24}O_2$ 548.1771; found 548.1797.

<u>14*H*-Anthra[3,2,1-*jk*]heptahelicen-14-one</u> 6A

<u>21*H*-Benzo[5,6]indeno[1,2-*l*]heptahelicen-21-one</u> 6F

The previous acid **46** (300 mg, 0.547 mmol) was suspended in methanesulfonic acid (12 mL) and the reaction was stirred at 80 °C for 1 hour. Then, the reaction was poured into water where brown solid was formed. The solid was collected by filtration over glass frit S4 and washed with water and then with methanol and dried. This solid was chromatographed on silica gel (toluene). The first isolated product corresponds to the fluoreno-derivative **6F** and is red crystalline solid (192 mg, 66%) the compound corresponds to the aceno-derivative **6A** and is an orange solid (80 mg, 28%). The combined yield (272 mg) is 94%.

<u>14*H*-Anthra[3,2,1-*jk*]heptahelicen-14-one</u> 6A

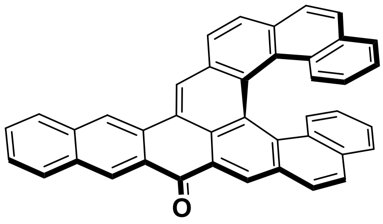

M.p.: 279 - 280 °C (CH_3CN).

Optical rotation: $[\alpha]^{20}_{D}$ +2014° (c 0.029, CH_2Cl_2); $[\alpha]^{20}_{D}$ -1945° (c 0.020, CH_2Cl_2).

^{1}H NMR (600 MHz, tetrachloroethane-d_2): 6.50 (ddd, *J* = 8.3, 6.8, 1.3, 1H), 6.53 (ddd, *J* = 8.3, 6.8, 1.3, 1H), 6.98 (ddd, *J* = 7.9, 6.8, 1.1, 1H), 7.01 (ddd, *J* = 8.0, 6.8, 1.1, 1H), 7.22 (d, *J* = 8.5, 1H), 7.24 (d, *J* = 8.4, 1H), 7.34 – 7.36 (m, 2H), 7.54 (d, *J* = 8.4, 1H), 7.59 (d, *J* = 8.4, 1H), 7.64 (ddd, *J* = 8.1, 6.7, 1.1, 1H), 7.73 (ddd, *J* = 7.9, 6.7, 1.2, 1H), 7.76 (d, *J* = 8.5, 1H), 7.95 (d, *J* = 8.5, 1H), 8.04 (d, *J* = 8.2, 1H), 8.15 – 8.16 (m, 2H), 8.20 (d, *J* = 8.2, 1H), 9.06 (s, 1H), 9.09 (s, 1H), 9.15 (s, 1H), 9.20 (s, 1H).

^{13}C NMR (151 MHz, tetrachloroethane-*d2*): 123.1, 123.6 (2C), 124.1, 124.2, 124.9, 125.2, 125.3, 125.59, 125.61, 125.7, 126.2, 126.7, 126.8, 126.9, 127.1 (2C), 127.8, 128.0, 128.3, 128.4, 128.50, 128.52, 128.8, 129.0, 129.1, 129.7, 129.8, 129.9, 130.4, 131.5, 131.7 (2C), 132.0, 132.2, 132.3, 132.8, 135.9, 184.2.

IR (KBr): 3048 w, 2968 w, 2924 w, 2853 w, 1659 vs, 1623 m, 1587 m, 1572 w, 1553 w, 1502 w, 1489 w, 1478 w, 1464 w, 1445 w, 1424 w, 1373 m, 1364 w, 1355 w, 1337 w, 1285 m, 1274 m,

1208 w, 1190 s, 1166 w, 1111 w, 1027 w, 921 w, 881 w, 830 m, 808 m, 745 s, 681 w, 608 w, 518 w, 475 w cm^{-1}.

FD MS: 530 ($[M]^{+\bullet}$).

HR MS FD: calcd for $C_{41}H_{22}O$ 530.1665; found 530.1673.

<u>21*H*-Benzo[5,6]indeno[1,2-*l*]heptahelicen-21-one</u> 6F

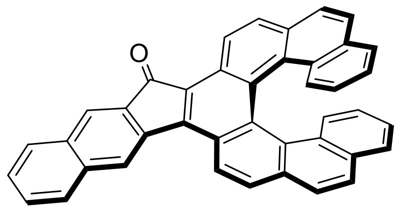

M.p.: > 325 °C (CH_3CN).

Optical rotation: $[\alpha]^{20}_D$ +434° (c 0.025, CH_2Cl_2); $[\alpha]^{20}_D$ -391° (c 0.032, CH_2Cl_2).

^{1}H NMR (600 MHz, tetrachloroethane-*d$_2$*): 6.49 – 6.52 (m, 2H), 6.94 – 6.97 (m, 2H), 7.02 – 7.04 (m, 2H), 7.30 – 7.32 (m, 2H), 7.47 (d, *J* = 8.1, 1H), 7.54 – 7.57 (m, 2H), 7.64 (ddd, *J* = 8.1, 6.9, 1.3, 1H), 7.69 (d, *J* = 8.4, 1H), 7.75 (d, *J* = 8.5, 1H), 7.96 (d, *J* = 7.9, 1H), 8.02 (d, *J* = 8.0, 1H), 8.05 (d, *J* = 8.5, 1H), 8.12 (d, *J* = 8.5, 1H), 8.22 (s, 1H), 8.51 (s, 1H), 8.88 (d, *J* = 8.5, 1H), 9.43 (d, *J* = 8.4, 1H).

^{13}C NMR (151 MHz, tetrachloroethane-*d$_2$*): 122.3, 122.6, 122.9, 124.1, 124.2, 124.3, 124.4, 124.6, 125.1 (2C), 125.36, 125.40, 126.63, 126.65, 126.7, 127.3, 127.46, 127.54, 127.9, 128.2, 128.5 (2C), 128.6, 128.8, 129.0, 129.16, 129.19, 129.4, 129.8, 130.4, 131.1, 131.3, 131.57, 131.59, 132.2, 133.1, 134.4, 136.7, 138.1, 143.6, 194.2.

IR (KBr): 3047 w, 2925 w, 2853 w, 1692 vs, 1629 s, 1604 w, 1593 w, 1551 w, 1512 m, 1495 w, 1474 w, 1439 w, 1421 w, 1353 w, 1337 w, 1291 w, 1267 w, 1250 m, 1239 w, 1203 w, 1180 w, 1151 w, 1142 w, 1119 w, 1106 w, 1059 w, 1024 w, 909 m, 876 w, 841 m, 828 m, 789 w, 744 s, 626 m, 606 w, 526 w, 473 m cm^{-1}.

FD MS: 530 ($[M]^{+\bullet}$).

HR MS FD: calcd for $C_{41}H_{22}O$ 530.1665; found 530.1677.

Synthesis of compounds 7A and 7F

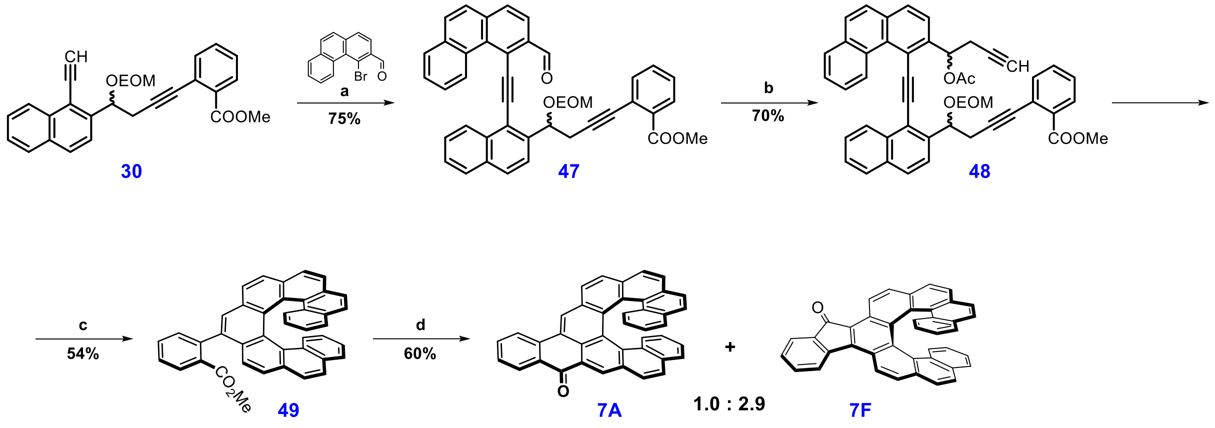

(a) 4-Bromophenanthrene-3-carbaldehyde (1.0 equiv.), diyne 22 (1.1 equiv.), $Pd(PPh_3)Cl_2$ (2 mol%), CuI (4 mol%), THF:Et_3N (3:2), 50 °C, 3 h.;
(b) Zn (2.0 equiv.), propargyl bromide (2.0 equiv.), compound 37 (1.0 equiv.), THF, rt, 15 min. then Et_3N (3.0 equiv.), Ac_2O (3.0 equiv.), THF, rt, 3 h.;
(c) triyne 38 (1.0 equiv.), CpCo(CO)(fum) (0.7 equiv.), PhCl, 180 °C, 20 min. then *p*-TsOH.H_2O (5.0 equiv.), 95 °C, 1h;
(d) ester 39 (1.0 equiv.), KOH (10.0 equiv.), THF:MeOH (1:1), 65 °C, 5 h. then $MeSO_3H$, 80°C, 2h.

Methyl 2-[4-(ethoxymethoxy)-4-{1-[(3-formylphenanthren-4-yl)ethynyl]naphthalen-2-yl}but-1-yn-1-yl]benzoate 47

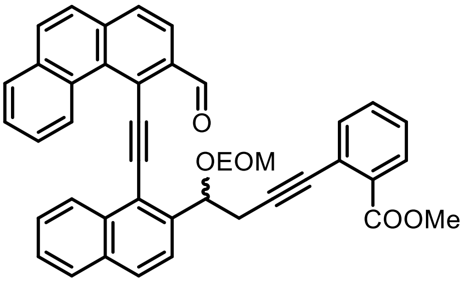

A Schlenk flask was charged with 4-bromophenanthrene-3-carbaldehyde (442 mg, 1.55 mmol), bis(triphenylphosphine)palladium chloride (21.7 mg, 0.03 mmol, 2 mol%), copper(I) iodide (11.8 mg, 0.06 mmol, 4 mol%) and flushed with argon. The degassed tetrahydrofuran (20 mL) and degassed triethylamine (20 mL) were added and the mixture was heated to 50 °C. Then diyne **30** (700 mg, 1.70 mmol, 1.1 equiv.) in degassed tetrahydrofuran (10 mL) was slowly added and the reaction was stirred at 50 °C for 3 hours. The solvents were evaporated under the reduced pressure. The residue was chromatographed on silica gel (hexane-ethyl acetate 20:1 to 10:1) to afford the desired product **47** (716 mg, 75%).

^{1}H NMR (400 MHz, $CDCl_3$): 1.01 (t, *J* = 7.1, 3H), 3.15 (dd, *J* = 17.2, 6.3, 1H), 3.21 (dd, *J* = 17.2, 6.9, 1H), 3.50 (dq, *J* = 9.5, 7.1, 1H), 3.63 (s, 3H), 3.74 (dq, *J* = 9.5, 7.1, 1H), 4.73 (d, *J* = 6.9, 1H), 4.85 (d, *J* = 6.9, 1H), 5.85 (t, *J* = 6.4, 1H), 7.18 – 7.27 (m, 3H), 7.52 – 7.59 (m, 3H), 7.62 (ddd, *J*

= 8.0, 7.0, 1.2, 1H), 7.74 – 7.78 (m, 2H), 7.87 (d, *J* = 8.6, 1H), 7.89 – 7.94 (m, 3H), 7.98 (d, *J* = 8.5, 1H), 8.00 (d, *J* = 8.4, 1H), 8.19 (d, *J* = 8.2, 1H), 8.51 – 8.54 (m, 1H), 10.39 – 10.43 (m, 1H), 11.19 (d, *J* = 0.9, 1H).

^{13}C NMR (101 MHz, $CDCl_3$): 15.0, 28.9, 51.9, 63.8, 75.0, 81.4, 91.5, 93.7, 96.8, 99.4, 118.6, 123.9, 124.0, 124.1, 124.2, 126.5, 126.8, 126.9, 127.0, 127.1, 127.4, 127.6, 127.7, 128.5, 128.9, 130.0, 130.07, 130.14, 130.4, 131.0, 131.1, 131.4, 131.9, 133.0, 133.37, 133.44, 134.4, 136.73, 136.74, 142.5, 166.7, 192.9.

IR ($CHCl_3$): 3061 w, 2978 w, 2953 w, 2931 w, 2884 w, 2233 w, 2187 w, 1728 s, 1699 m, 1682 vs, 1629 w, 1599 w, sh, 1588 m, 1568 w, 1509 w, 1468 w, sh, 1449 w, 1435 m, 1394 m, 1300 m, 1276 m, 1257 vs, 1147 m, 1131 m, 1097 m, 1043 m, sh, 1029 s, 965 w, 868 w, 850 m, 825 w, 636 w, 576 vw, 541 w, 522 w cm^{-1}.

ESI MS: 639 ($[M+Na]^+$).

HR ESI MS: calcd for $C_{42}H_{32}O_5Na$ 639.2142, found 639.2140.

Methyl 2-{4-[1-({3-[1-(acetyloxy)but-3-yn-1-yl]phenanthren-4-yl}ethynyl)naphthalen-2-yl]-4-(ethoxymethoxy)but-1-yn-1-yl}benzoate 48

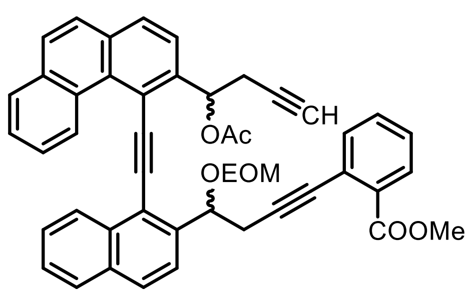

A Schlenk flask was filled with zinc powder (143 mg, 2.18 mmol, 2 equiv.) and flushed with argon. Freshly distilled tetrahydrofuran (6 mL) was added and vigorously stirred. Then propargyl bromide (80 wt. % in toluene, 243 µL, 2.18 mmol, 2 equiv.) was added and stirred for 10 min, before it was transferred by a syringe to the second Schlenk flask, which contained aldehyde **47** (673 mg, 1.09 mmol) and tetrahydrofuran (40 mL). The reaction mixture was stirred for 5 min at room temperature, before triethylamine (449 µL, 3.27 mmol, 3 equiv.) and acetic anhydride (309 µL, 3.27 mmol, 3 equiv.) were added and the solution was stirred for 3 h at room temperature. The solvents were evaporated under the reduced pressure and the residue was purified by flash chromatography on silica gel (hexane-ethyl acetate 10:1) to obtain acetate **48** (532 mg, 70%) as a mixture of diastereomers in a ratio about 1:1.

^{1}H NMR (400 MHz, $CDCl_3$, mixture of diastereomers): 0.93 (t, *J* = 7.1, 3H), 0.96 (t, *J* = 7.1, 3H), 1.99 (t, *J* = 2.6, 1H), 2.00 (t, *J* = 2.6, 1H), 2.14 (s, 3H), 2.15 (s, 3H), 2.93 – 3.11 (m, 4H), 3.17 – 3.24 (m, 4H), 3.42 – 3.54 (m, 2H), 3.63 (s, 3H), 3.63 – 3.72 (m, 2H), 3.66 (s, 3H), 4.76 (d, *J* = 6.8,

1H), 4.79 (d, *J* = 6.8, 1H), 4.84 (d, *J* = 6.8, 1H), 4.89 (d, *J* = 6.8, 1H), 5.88 (t, *J* = 6.1, 1H), 5.89 (t, *J* = 6.1, 1H), 7.027 (t, *J* = 5.9, 1H), 7.031 (t, *J* = 5.9, 1H), 7.19 – 7.32 (m, 6H), 7.43 – 7.51 (m, 2H), 7.52 – 7.59 (m, 6H), 7.73 (d, *J* = 8.8, 2H), 7.77 – 7.81 (m, 4H), 7.836 (d, *J* = 8.3, 1H), 7.840 (d, *J* = 8.3, 1H), 7.86 – 7.93 (m, 6H), 7.94 – 7.99 (m, 4H), 8.63 – 8.70 (m, 2H), 10.39 (bd, *J* = 8.5, 2H).
^{13}C NMR (101 MHz, $CDCl_3$, mixture of diastereomers): 14.96, 15.01, 21.18, 21.20, 26.0, 26.1, 28.8, 29.0, 51.9, 52.0, 63.7, 63.8, 71.4 (2C), 71.9, 72.0, 75.0, 75.5, 79.5 (2C), 81.17, 81.22, 92.14, 92.15, 94.1, 94.2, 97.2, 97.4, 98.3, 98.5, 117.1, 117.2, 118.7, 118.9, 123.8 (2C), 124.19, 124.22, 124.3 (2C), 126.4, 126.5, 126.7 (2C), 126.8, 126.9, 127.02, 127.04, 127.1 (2C), 127.2, 127.28 (2C), 127.31, 127.39, 127.41, 128.3 (2C), 128.48 (2C), 128.53 (2C), 129.4 (2C), 130.0 (2C), 130.1 (2C), 130.3 (2C), 130.8 (2C), 131.42, 131.44, 131.97, 131.99, 132.8 (2C), 133.0 (2C), 133.4 (2C), 133.5 (2C), 134.5 (2C), 142.1, 142.2, 142.4, 142.5, 166.87, 166.88, 169.7, 169.8.
IR ($CHCl_3$): 3309 m, 3058 w, 2977 w, 2953 m, 2930 m, 2886 w, 2233 w, 2192 vw, 2124 vw, 1733 vs, 1712 s, sh, 1624 w, 1597 w, 1568 w, 1503 w, 1486 m, 1469 w, sh, 1449 m, 1435 m, 1372 m, 1300 m, 1277 m, 1253 s, sh, 1239 vs, 1164 w, 1146 m, 1131 w, 1097 m, 1043 vs, 1028 s, 966 w, 868 w, 845 m, 824 w, 640 w, 539 w cm^{-1}.
ESI MS: 721 ($[M+Na]^+$).
HR ESI MS: calculated for $C_{47}H_{38}O_6Na$ 721.2561, found 721.2560.

<u>**Methyl 2-octahelicen-9ylbenzoate**</u> **49**

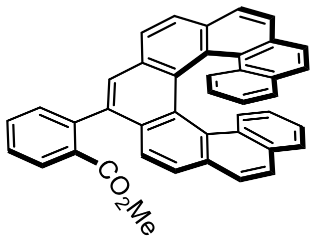

Triyne **48** (506 mg, 0.72 mmol) was loaded to a microwave vial and chlorobenzene (18 mL) was added. The solution was bubbled with nitrogen for 5 minutes before CpCo(CO)(fum) (149 mg, 0.50 mmol, 0.7 equiv, fum = dimethylfumarate) was added. The seal was closed and the reaction mixture was heated to 180 °C in the microwave reactor for 20 minutes. *p*-Toluenesulfonic acid monohydrate (684 mg, 3.60 mmol, 5 equiv.) was added and the reaction mixture was strirred for 1 h at 95 °C. The solvent was evaporated in vacuum. The residue was purified by flash chromatography on silica gel (hexane-ethyl acetate 20:1 to 10:1) to obtain helicene **49** (218 mg, 54%) as a mixture of two atropodiastereomers in a ratio 3.3:1.
^{1}H NMR (400 MHz, $CDCl_3$, major atropodiastereomer): 3.67 (s, 3H), 6.39 – 6.45 (m, 1H), 6.49 (ddd, *J* = 8.3, 6.8, 1.4, 1H), 6.95 – 7.06 (m, 4H), 7.07 – 7.20 (m, 4H), 7.26 – 7.33 (m, 3H), 7.37

(d, *J* = 8.7, 1H), 7.407 (d, *J* = 8.4, 1H), 7.413 (d, *J* = 8.2, 1H), 7.57 (dd, *J* = 7.4, 1.5, 1H), 7.60 – 7.65 (m, 1H), 7.79 (d, *J* = 8.2, 1H), 7.84 (s, 1H), 7.96 – 8.03 (m, 2H), 8.22 (dd, *J* = 7.9, 1.5, 1H).

^{13}C NMR (101 MHz, $CDCl_3$, major atropodiastereomer): 52.1, 123.85, 123.87, 124.0 (2C), 124.5 (2C), 125.1, 125.3, 125.7, 125.8, 125.9, 126.3, 126.4, 126.69 (2C), 126.74 (2C), 126.76, 126.8, 126.9, 127.0, 127.4, 127.8, 127.9, 127.96, 128.01, 128.1, 130.1, 130.51, 130.53, 130.9, 131.4, 131.7, 131.76, 131.82, 132.0, 132.2, 132.3, 139.1, 141.6, 167.9.

IR ($CHCl_3$): 3053 w, 2953 w, 1724 s, 1618 vw, 1597 vw, 1570 vw, 1506 vw, 1485 w, 1465 vw, 1447 w, 1434 w, 1393 w, 1293 s, 1272 m, 1258 s, 1236 m, 1190 m, sh, 1164 w, 1132 w, sh, 1099 w, 1077 w, 1043 w, 963 w, sh, 881 w, 834 vs, 810 vw, 692 w, 644 vw, 608 w, 599 w, 587 w, 552 w, 525 w cm^{-1}.

ESI MS: 585 ([M+Na]$^+$).

HR ESI MS: calculated for $C_{42}H_{26}O_2Na$ 585.1825, found 585.1824.

16*H*-Naphtho[3,2,1-*jk*]octahelicen-16-one 7A

17*H*-Indeno[1,2-*l*]octahelicen-17-one 7F

Ester **49** (182 mg, 0.32 mmol) was dissolved in tetrahydrofuran (10 mL) and methanol (10 mL), and potassium hydroxide (180 mg, 3.20 mmol, 10 equiv.) was added. The mixture was heated to 65 °C and stirred for 5 h. Then the reaction was cooled to rt and diluted hydrochloric acid was added to reach pH 1. The mixture was extracted with diethyl ether (2x 20 mL), and the combined organic layers were dried over anhydrous magnesium sulfate. The solvents were evaporated under the reduced pressure to obtain crude carboxylic acid, which was directly used further. This acid was dissolved in methanesulfonic acid (10 mL) and the flask was put to the oil bath pre-heated to 80 °C. The stirring was continued for 2 h at 80 °C and then the reaction mixture was poured over crushed ice. The mixture was extracted with dichloromethane (2x 20 mL), the combined organic layers were washed with saturated sodium bicarbonate solution (10 mL), dried over anhydrous magnesium sulfate. The solvent was evaporated *in vacuo* and the residue was purified by flash chromatography on silica gel (hexane-dichloromethane 2:1 to 1:3) to afford **7A** (26.3 mg, 15%) as an orange solid and **7F** (76.5 mg, 45%) as a red solid.

16*H*-Naphtho[3,2,1-*jk*]octahelicen-16-one 7A

M.p.: > 315 °C (CH_3CN).

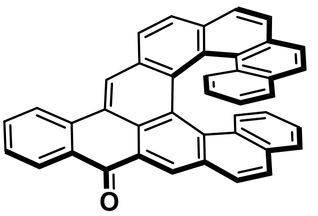

Optical rotation: $[\alpha]^{20}_D$ +4789° (c 0.026, CH_2Cl_2); $[\alpha]^{20}_D$ -4633° (c 0.025, CH_2Cl_2).

^{1}H NMR (400 MHz, $CDCl_3$): 6.37 (ddd, *J* = 8.4, 6.8, 1.4, 1H), 6.49 (ddd, *J* = 8.4, 6.9, 1.4, 1H), 6.99 (ddd, *J* = 8.0, 6.8, 1.2, 1H), 7.04 (ddd, *J* = 8.0, 6.8, 1.1, 1H), 7.05 – 7.10 (m, 3H), 7.19 (d, *J* = 8.5, 1H), 7.21 (d, *J* = 8.5, 1H), 7.23 (d, *J* = 7.9, 1H), 7.33 (dt, *J* = 8.0, 1.7, 2H), 7.42 (d, *J* = 8.2, 1H), 7.64 (ddd, *J* = 8.0, 7.1, 1.1, 1H), 7.77 (d, *J* = 8.2, 1H), 7.86 (ddd, *J* = 8.2, 7.1, 1.5, 1H), 8.02 (d, *J* = 8.2, 1H), 8.11 (d, *J* = 8.3, 1H), 8.61 (dd, *J* = 7.9, 1.5, 1H), 8.62 (dd, *J* = 7.9, 1.0, 1H), 8.67 (s, 1H), 8.89 (s, 1H).

^{13}C NMR (101 MHz, $CDCl_3$): 123.7, 123.9, 124.4, 124.5, 124.6, 125.0, 125.1, 125.23, 125.25, 125.5, 125.9, 126.0, 126.1, 126.3, 126.4, 126.6, 126.99, 127.04, 127.10, 127.13, 127.5, 127.6, 127.7, 127.8, 128.0, 128.1, 128.36, 128.40, 128.5, 130.3, 130.6, 130.7, 131.1, 131.2, 131.8, 132.3, 132.9, 133.1, 133.7, 136.8, 184.1.

IR ($CHCl_3$): 3045 w, 2958 w, 2928 w, 2856 w, 1648 vs, 1601 s, 1581 vs, 1752 m, 1553 w, 1487 m, 1466 w, 1424 w, 1372 s, 1333 w, 1300 m, 1284 s, 1271 m, 1260 w, 1175 w, 1168 w, 1128 w, 1070 vw, 1008 w, 962 vw, 919 w, 883 w, sh, 838 s, 811 m, 699 w, 685 w, 624 m, 603 w, 593 vw, 552 w, 520 w, 476 vw cm^{-1}.

APCI MS: 531 ($[M+H]^+$).

HR APCI MS: calcd for $C_{41}H_{23}O$ 531.1743, found 531.1736.

<u>17*H*-Indeno[1,2-*l*]octahelicen-17-one</u> 7F

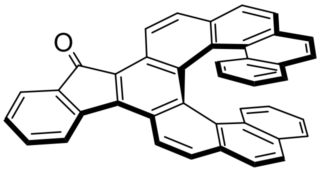

M.p.: > 315 °C (CH_3CN).

^{1}H NMR (600 MHz, CD_2Cl_2): 6.47 (ddd, *J* = 8.3, 6.8, 1.4, 1H), 6.52 (ddd, *J* = 8.3, 6.7, 1.4, 1H), 6.95 (dt, *J* = 8.4, 1.0, 1H), 7.00 (ddd, *J* = 7.9, 6.7, 1.2, 1H), 7.04 (ddd, *J* = 7.9, 6.7, 1.2, 1H), 7.06 (d, *J* = 8.3, 1H), 7.07 (d, *J* = 8.3, 1H), 7.18 (d, *J* = 8.4, 1H), 7.21 (d, *J* = 8.5, 1H), 7.27 (dt, *J* = 8.4, 0.9, 1H), 7.32 (ddt, *J* = 7.9, 1.4, 0.8, 1H), 7.35 (ddt, *J* = 8.0, 1.4, 0.7, 1H), 7.37 (d, *J* = 8.1, 1H), 7.41 (td, *J* = 7.6, 0.8, 1H), 7.52 (d, *J* = 8.5, 1H), 7.62 (td, *J* = 7.5, 1.3, 1H), 7.75 (d, *J* = 8.1, 1H), 7.77 (ddt, *J* = 7.1, 1.3, 0.6, 1H), 8.07 (d, *J* = 8.4, 1H), 8.21 (dq, *J* = 7.5, 0.6, 1H), 8.53 (d, *J* = 8.5, 1H), 9.31 (d, *J* = 8.4, 1H).

^{13}C NMR (151 MHz, CD_2Cl_2): 122.5, 122.6, 123.7, 123.9 (2C), 124.1, 124.8, 124.99, 125.00, 125.03, 125.3, 125.6, 126.20, 126.23, 126.6, 127.14, 127.15, 127.2, 127.3, 127.9, 127.98, 128.01, 128.2, 128.4, 128.5 (2C), 128.6, 128.8, 129.3, 129.9, 131.3, 132.1, 132.2, 132.3, 132.4, 132.9, 134.7, 136.2, 144.0, 144.6, 195.8.

IR ($CHCl_3$): 3044 w, 3014 w, 2953 w, 2920 w, 2851 w, 1695 s, 1606 w, 1590 w, 1582 w, 1562 w, 1515 w, 1463 w, 1431 vw, 1375 vw, 1329 vw, 1275 w, 1240 w, 1230 vw, 1192 vw, 1171 w, 1087 w, 1044 w, 1005 w, 943 w, 892 w, 860 w, 839 m, 823 vs, 689 w, 610 m, 602 w, 590 w, 553 w, 536 w, 524 m, 476 w cm^{-1}.

APCI MS: 531 ($[M+H]^+$).

HR APCI MS: calcd for $C_{41}H_{23}O$ 531.1743, found 531.1735.

Synthesis of compounds 8A and 8F

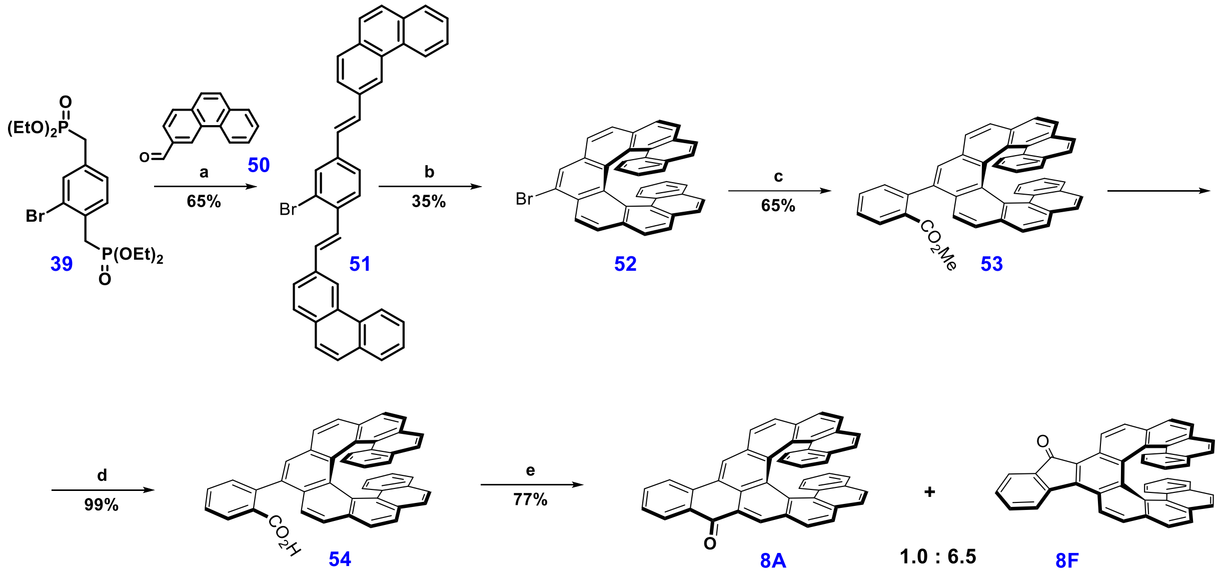

(a) Compound 39 (1.0 equiv.), aldehyde 50 (2.0 equiv.), NaH (4.0 equiv.), PhMe, 120 °C, 30 min;
(b) stilbene 51 (1.0 equiv.), I_2 (0.55 equiv.), PhMe:EtOAc (8:1), hν, rt, 40 h;
(c) helicene 52 (1.0 equiv.), 2-methoxycarbonylphenylboronic acid (1.3 equiv.), Pd_2dba_3 (4 mol%), Xphos (8 mol%), $K_3PO_4.H_2O$ (6.0 equiv.), PhMe, 90°C, 14 h;
(d) ester 42 (1.0 equiv.), NaOH (5.0 equiv.), THF:MeOH:H_2O (11:2.8:1.4), 70 °C, 16 h;
(e) $MeSO_3H$, 80°C, 1 h.

Phenanthrene-3-carbaldehyde 50

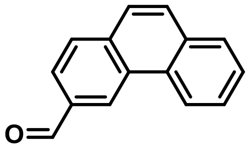

In a well dried Schlenk flask, 3-bromophenanthrene (2.28 g, 8.87 mmol) was dissolved in anhydrous THF (110 mL) under argon. The solution was cooled to -78 °C and then a solution of n-butyllithium (2.5 M in hexanes, 3.6 mL, 9.0 mmol, 1.01 equiv.) was added dropwise. This reaction mixture was stirred at the same temperature for 30 min and then dimethylformamide (1.37 mL, 17.7 mmol, 2.0 equiv.) was added dropwise. The reaction was stirred at -78 °C for 30 min and then warmed to -20 °C.

At this temperature the reaction was quenched with brine (50 mL). The organic phase was separated and the water phase was extracted with diethyl ether (3 x 50 mL). The organic portions were combined and evaporated *in vacuo* and the residue was purified by column chromatography on silca gel (cyclohexane- ethyl acetate 30:1). The product **50** was obtained as a white solid (1.52 g, yield 83%). ^{1}H NMR spectrum is in agreement with the published data.[16]

^{1}H NMR (400 MHz, $CDCl_3$): 7.68 (ddd, *J* = 8.1, 7.0, 1.2, 1H), 7.75 (ddd, *J* = 8.4, 7.0, 1.5, 1H), 7.79 (d, *J* = 8.8, 1H), 7.90 (d, *J* = 8.9, 1H), 7.94 (dd, *J* = 7.8, 1.5, 1H), 8.00 (d, *J* = 8.2, 1H), 8.08 (dd, *J* = 8.2, 1.5, 1H), 8.78 (d, *J* = 8.2, 1H), 9.17 (s, 1H) 10.27 (s, 1H).

<u>**3,3'-[(2-Bromobenzene-1,4-diyl)di(*E*)ethene-2,1-diyl]diphenanthrene**</u> **51**

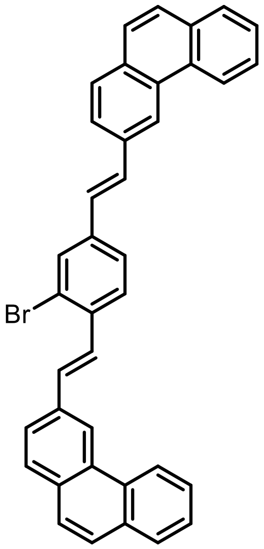

In a well dry round bottom flask were solved previous bisphosphate **39** (1.66 g, 3.63 mmol) and aldehyde **50** (1.5 g, 7.27 mmol, 2.0 equiv.) in dry toluene (90 mL). To this mixture was added NaH (60%, dispersion in mineral oil, 322 mg, 13.45 mmol, 4.0 equiv.) and the mixture was heated at 120 °C for 30 min under inert. The reaction mixture was cooled to room temperature and then slowly poured into a mixture of ice-water- 37 % HCl (100 g -100 ml-100 ml). The organic phase with partially precipitated yellow product was separated and the water phase was extracted with toluene (3 x 50 mL). The organic portions were combined and the volume was reduced to its 20 % on rotavap. The product **51** as a yellow precipitate (1.33 g, 65%) was collected by filtration over a glass frit S3.

^{1}H NMR (400 MHz, CD_2Cl_2): 7.28 – 7.32 (m, 1H), 7.43 – 7.50 (m, 2H), 7.63 – 7.70 (m, 3H), 7.72 – 7.79 (m, 7H), 7.84 – 7.88 (m, 2H), 7.92 - 7.96 (m, 6H), 8.79 – 8.83 (m, 4H).

^{13}C NMR (101 MHz, CD_2Cl_2): 121.9, 122.1, 122.55, 122.56, 124.1, 124.2, 124.6, 125.7, 126.55, 126.58, 126.6, 126.8 (3C), 126.9 (2C), 127.1, 127.19, 127.22, 127.3, 128.7, 129.05, 129.06, 129.9 (2C), 130.2, 130.27, 130.28, 130.9, 131.4, 131.67, 131.73, 132.09, 132.10, 134.9, 135.1, 135.8, 138.2.

FD MS: 560 ($[M]^{+\bullet}$).

HR MS FD: calcd for $C_{38}H_{25}{}^{79}Br$ 560.1134; found 560.1144.

---

[16] S. Fujino, M. Yamaji, H. Okamoto, T. Mutai, I. Yoshikawa, H. Houjou, F. Tani *Photochem. Photobiol. Sci.*, **2017**, *16*, 925.

11-Bromononahelicene 52

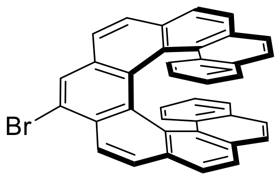

The previous stilbene **51** (1 g, 1.78 mmol) was suspended in toluene (400 mL). This mixture was heated to fully dissolve the starting material. When all the solid was dissolved, the mixture was diluted with toluene (1200 mL) and ethyl acetate (200 mL). Iodine (250 mg, 0.985 mmol) was added to the mixture and the reaction mixture was irradiated with medium-pressure mercury (150 W) lamp for 40 h open to air. The whole reaction mixture was evaporated, and the residue was adsorbed on silica-gel. The product was isolated by continuous extraction with refluxing cyclohexane using Thiele-Pappe attachment. After evaporation of cyclohexane, the residue was triturated in diethyl ether and filtered over glass frit S4 to get slightly yellow product **52** (350 mg, 35%).

^{1}H NMR (400 MHz, CD_2Cl_2): 6.41 (ddt, *J* = 8.3, 6.8, 1.4, 2H), 6.99 – 7.06 (m, 3H), 7.08 – 7.14 (m, 5H), 7.19 (d, *J* = 8.5, 1H), 7.32 (d, *J* = 6.2, 1H), 7.34 (d, *J* = 6.1, 1H), 7.36 – 7.40 (m, 1H), 7.46 (d, *J* = 8.2, 1H), 7.55 (d, *J* = 8.4, 1H), 7.75 (d, *J* = 8.2, 1H), 8.24 (d, *J* = 8.4, 1H), 8.32 (s, 1H).

^{13}C NMR (151 MHz, tetrachloroethane-d_2): 121.1, 122.3, 122.5, 123.56, 123.60, 124.2, 124.6, 124.65, 124.7, 124.8, 125.1, 125.3, 125.4, 125.6, 126.0, 126.1, 126.2, 126.38, 126.43, 126.6, 126.7, 126.8, 127.0, 127.4, 127.5, 128.7, 129.2, 129.7, 129.9, 130.0, 131.0, 131.8, 131.88, 131.92, 131.94.

FD MS: 556 ($[M]^{+\bullet}$).

HR MS FD: calcd for $C_{38}H_{21}{}^{79}Br$ 556.0821; found 556.0830.

Methyl 2-nonahelicen-11-ylbenzoate 53

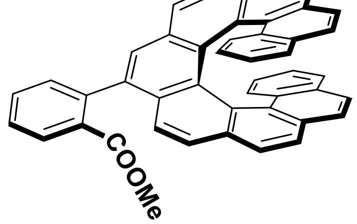

In a dry Schlenk flask was dissolved 11-bromo[9]helicene **52** (270 mg, 0.48 mmol), 2-methoxy-carbonylphenylboronic acid (113 mg, 0.63 mmol, 1.3 equiv.), Pd_2dba_3 (17.6 mg, 19.2 µmol, 4 mol%), Xphos (18.3 mg, 38.4 µmol, 8 mol%) and $K_3PO_4.H_2O$ (663 mg, 2.88 mmol, 6 equiv.) in dry toluene (32 mL). The reaction mixture was thoroughly degassed and then heated at 90 °C overnight. The solvent was evaporated *in vacuo* and the residue was purified by column chromatography on silica gel (cyclohexane - dichloromethane 2:1 to 1:1). The product **53** was obtained as a yellow solid (193 mg, 65%). The ^{1}H NMR shows mixture of two atropodiastereomers (1:2.5).

^{1}H NMR (400 MHz, CD_2Cl_2): 3.19 and 3.68 (s, in ratio 1:2.5, 3H), 6.37 – 6.51 (m, 2H), 6.94 – 7.13 (m, 6H), 7.14 – 7.23 (m, 3H), 7.25 – 7.48 (m, 8H), 7.64 – 7.69 (m, 1H), 7.74 – 7.86 (m, 4H), 8.11 and 8.23 (m, in ratio 1:2.4, 1H).

^{13}C NMR: Not recorded due to mixture of atropoisomers.

FD MS: 612 ($[M]^{+\bullet}$).

HR MS FD: calcd for $C_{46}H_{28}O_2$ 612.2084; found 612.2090.

2-Nonahelicene-11-ylbenzoic acid 54

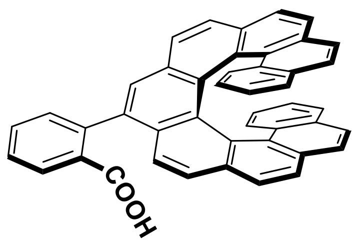

The previous compound **53** (276 mg, 0.45 mmol) and sodium hydroxide (90 mg, 2.25 mmol, 5 equiv.) was dissolved in a mixture of solvents of THF-methanol-water (11 mL:2.8 mL:1.4 mL). The mixture was stirred overnight at 70 °C under inert. Then the reaction was acidified by diluted hydrochloric acid and the mixture was extracted with dichloromethane (4 x 10 mL). The combined organic portions were dried over $MgSO_4$. After filtration and evaporation of all the volatiles was obtained product **54** as an amorphous yellow solid (267 mg, yield 99%).

^{1}H NMR (400 MHz, tetrachloroethane-*d*$_2$, 110°C): 6.44 – 6.53 (m, 2H), 7.03 – 7.13 (m, 6H), 7.21 – 7.24 (m, 3H), 7.29 – 7.50 (m, 8H), 7.68 – 7.71 (m, 1H), 7.79 – 7.91 (m, 4H), 8.24 and 8.32 (d, in ratio 1:2.3, 1H).

^{13}C NMR: Not recorded due to mixture of atropoisomers.

FD MS: 598 ($[M]^{+\bullet}$).

HR MS FD: calcd for $C_{45}H_{26}O_2$ 598.1927; found 598.1934.

18*H*-Naphtho[3,2,1-*mn*]nonahelicen-18-one 8A

23*H*-Indeno[1,2-*o*]nonahelicen-23-one 8F

The previous acid **54** (200 mg, 0.334 mmol) was suspended in methanesulfonic acid (8 mL) and the reaction was stirred at 80 °C for 1 hour. Then, the reaction was poured into water where brown solid was formed. The solid was collected by filtration over glass frit S4 and washed with water and then with methanol and dried. This solid was chromatographed on silica gel (toluene). The first isolated product corresponds to the fluoreno-derivative **8F** and is red solid (130 mg, 66%) the compound corresponds to the aceno-derivative **8A** and is an orange solid (20 mg, 10%). The combined yield (150 mg) is 76 %.

<u>18*H*-Naphtho[3,2,1-*mn*]nonahelicen-18-one</u> 8A

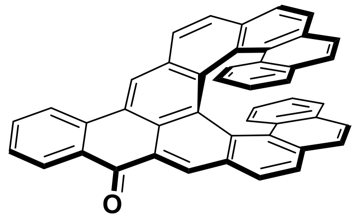

M.p.: > 325 °C (CH_3CN).

Optical rotation: $[\alpha]^{20}_D$ +6015° (c 0.026, CH_2Cl_2); $[\alpha]^{20}_D$ -6297° (c 0.028, CH_2Cl_2).

^{1}H NMR (600 MHz, tetrachloroethane-*d*$_2$): 6.36 (ddd, *J* = 8.3, 6.7, 1.3, 1H), 6.52 (ddd, *J* = 8.3, 6.7, 1.3, 1H), 7.05 (ddd, *J* = 7.9, 6.7, 1.2, 1H), 7.10 (ddd, *J* = 7.9, 6.7, 1.1, 1H), 7.13 (s, 2H), 7.17 (d, *J* = 8.6, 1H), 7.18 (d, *J* = 8.2, 1H), 7.20 (d, *J* = 8.6, 1H), 7.22 (d, *J* = 8.5, 1H), 7.25 (d, *J* = 8.5, 1H), 7.33 (d, *J* = 8.1, 1H), 7.37 – 7.45 (m, 4H), 7.56 (d, *J* = 8.1, 1H), 7.69 – 7.71 (m, 1H), 7.94 (ddd, *J* = 8.2, 7.1, 1.5, 1H), 7.98 (d, *J* = 8.1, 1H), 8.62 (dd, *J* = 7.8, 1.4, 1H), 8.70 (s, 1H), 8.71 (d, *J* = 8.2, 1H), 8.95 (s, 1H).

^{13}C NMR (151 MHz, tetrachloroethane-*d*$_2$): 122.3, 122.8, 123.6, 123.9, 124.1, 124.4, 124.5, 124.86, 124.88, 125.0, 125.2, 125.4, 125.5, 125.8, 125.9, 126.0, 126.1, 126.30, 126.33, 126.59, 126.62, 126.7, 126.9, 127.1, 127.2, 127.58, 127.62, 127.65, 127.8, 128.0, 128.06, 128.10, 128.4, 129.9, 130.3, 130.7, 130.8, 131.1, 131.7, 131.9, 132.3, 132.6, 133.7, 136.5, 183.7.

IR (KBr): 3045 w, 2977 w, 2924 w, 2854 w, 1646 s, 1641 s, 1600 m, 1508 m, 1565 w, 1487 w, 1464 w, 1371 w, 1292 m, 1278 w, 1260 w, 1228 w, 1165 w, 1139 w, 1129 w, 1062 w, 1012 w, 976 w, 876 w, 828 s, 753 vs, 744 s, 721 w, 650 w, 621 m, 599 w, 589 m, 521 w, 489 w cm^{-1}.

FD MS: 580 ($[M]^{+\bullet}$).

HR MS FD: calcd for $C_{45}H_{24}O$ 580.1822; found 580.1832.

<u>23*H*-Indeno[1,2-*o*]nonahelicen-23-one</u> 8F

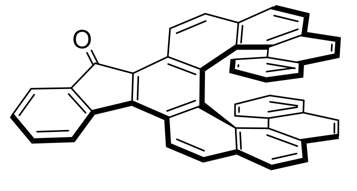

M.p.: > 350°C (CH_3CN).

^{1}H NMR (600 MHz, tetrachloroethane-*d*$_2$): 6.51 – 6.57 (m, 2H), 7.05 – 7.13 (m, 6H), 7.20 (d, *J* = 8.5, 2H), 7.21 (d, *J* = 8.5, 1H), 7.34 (m, *J* = 8.5, 3H), 7.41 – 7.43 (m, 2H), 7.44 – 7.46 (m, 1H), 7.49 (d, *J* = 8.4, 1H), 7.56 (d, *J* = 8.4, 1H), 7.66 (td, *J* = 7.5, 1.3, 1H), 7.82 (d, *J* = 7.2, 1H), 8.24 (d, *J* = 7.4, 1H), 8.53 (d, *J* = 8.4, 1H), 9.07 (d, *J* = 8.3, 1H).

^{13}C NMR (151 MHz, tetrachloroethane-*d*$_2$): 121.4, 121.7, 122.6, 122.9, 123.4, 123.6, 123.9, 124.2, 124.7, 124.84, 124.86, 124.91, 124.94, 125.3, 125.4, 125.7, 125.8, 126.17, 126.23, 126.3, 126.4, 126.58, 126.61, 126.7, 126.79, 126.83, 126.85, 126.9, 127.2, 127.9, 128.4, 128.6, 128.9, 129.2, 129.99, 130.03, 131.9, 132.0, 132.1, 132.9, 133.4, 135.6, 143.8, 144.0, 195.8.

IR (ATR): 3043 w, 1695 ms, 1606 m, 1584 w, 1561 w, 1575 w, 1519 w, 1504 w, 1483 w, 1460 m, 1433 w, 1395 w, 1377 w, 1330 m, 1273 w, 1236 w, 1225 m, 1216 m, 1182 m, 1168 m, 1156 m, 1087 w, 1044 w, 1037 w, 1028 w, 1007 w, 975 w, 947 w, 936 w, 920 w, 885 w, 863 w, 850 m, 831 ms, 813 m, 788 ms, 750 m, 739 ms, 722 s, 701 ms, 680 w, 674 w, 656 w, 646 w, 637 w, 623 m, 608 w, 597 m, 590 m, 536 m, 524 m, 507 w, 472 w, 443 w, 427 w, 416 w cm^{-1}.

FD MS: 580 ($[M]^{+\bullet}$).

HR MS FD: calcd for $C_{45}H_{24}O$ 580.1822; found 580.1815.

HPLC Resolution of 1A, 2A, 3A, 4A, 5A, 6A, 6F, 7A, and 8A into Enantiomers

Chiral resolution of 1A

Chiral HPLC separation: *Anal.*: ChiralArt Amylose-SA (150 x 3 mm, 3 µm, YMC), heptane – toluene 50:50, 1.0% IPA @ 1.0 mL/min., 35 °C, $t_R(+)$ = 6.0 min. (>99% *ee*), $t_R(-)$ = 8.4 min. (>99% *ee*); *Semiprep.*: ChiralArt Amylose-SA (250 x 20 mm, 5 µm, YMC), heptane – toluene 50:50, 0.5% IPA @20 mL/min.

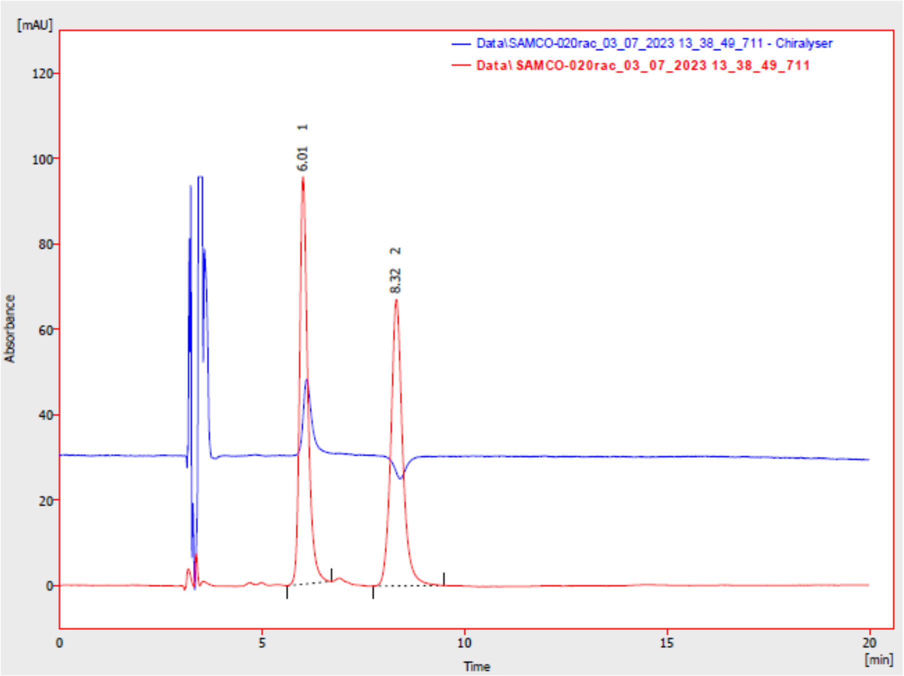

Result Table (Uncal - Data|SAMCO-020rac_03_07_2023 13_38_49_711)

	Reten. Time [min]	Area [mAU.s]	Height [mAU]	Area [%]	Height [%]	W05 [min]	PDA Peak Purity	Compound Name
1	6.012	1335.201	95.428	49.8	58.7	0.20	910	
2	8.317	1347.896	67.064	50.2	41.3	0.29	941	
	Total	2683.098	162.492	100.0	100.0			

Figure S4. HPLC of *rac*-**1A** (red: UV detector (315 nm), blue: downstream polarimetric detector).

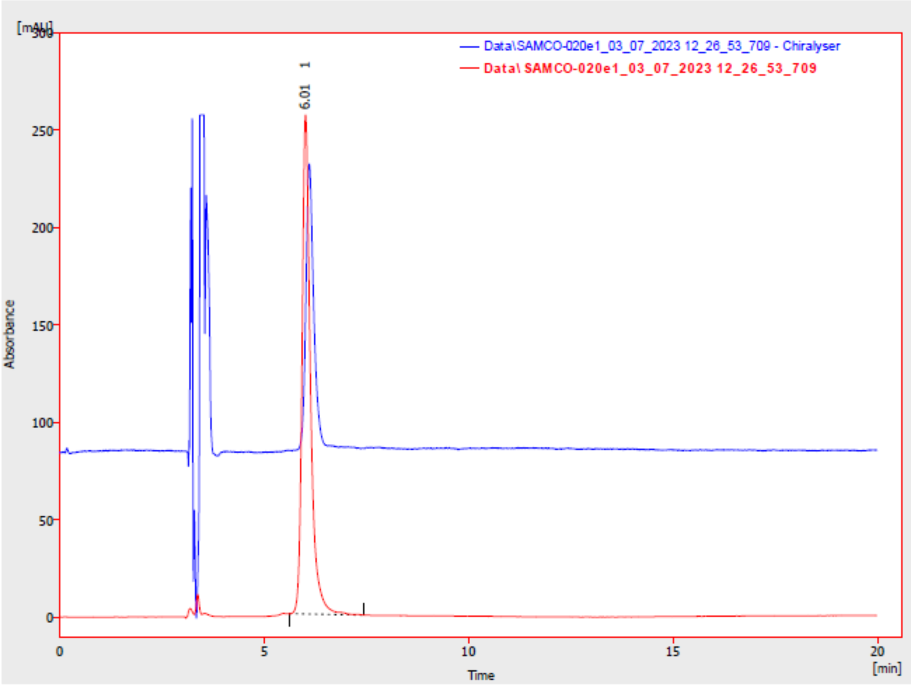

Figure S5. HPLC of (+)-(*P*)-**1A** (red: UV detector (315 nm), blue: downstream polarimetric detector).

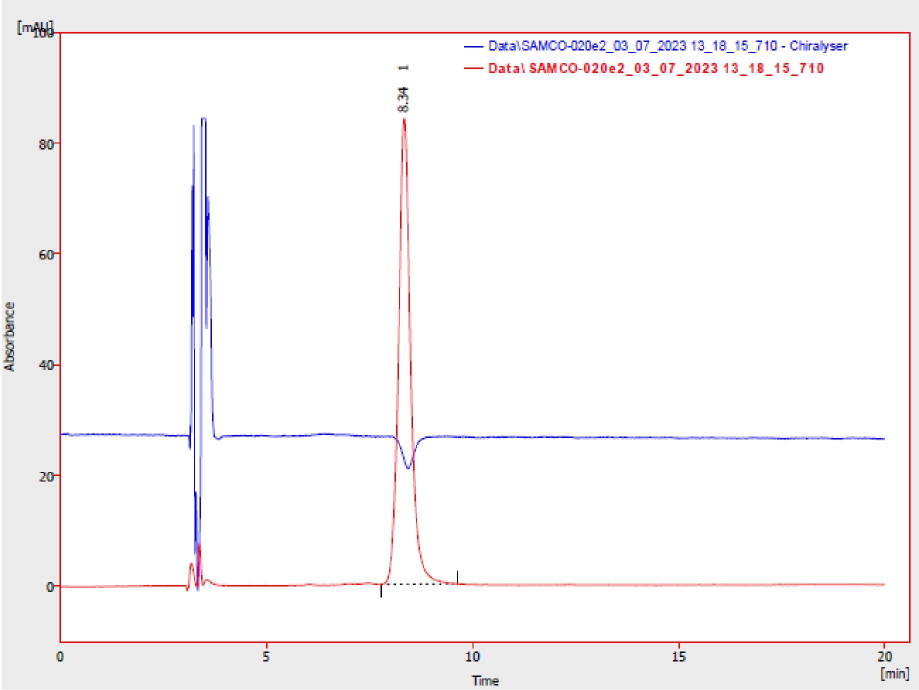

Figure S6. HPLC of (-)-(*M*)-**1A** (red: UV detector (315 nm), blue: downstream polarimetric detector).

Chiral resolution of 2A

Chiral HPLC separation: *Anal*.: Chiralpak IE (250 x 4.6 mm, 5 mm, DAICEL), toluene, 1% IPA @ 1.0 mL/min., 35 °C, $t_R(+)$ = 7.2 min. (>99% *ee*), $t_R(-)$ = 8.8 min. (>99% *ee*); *Semiprep*.: CHIRALPAK® IE (250 x 20 mm, 5 µm, Chiral Technologies), toluene, 0.5% IPA @20 mL/min.

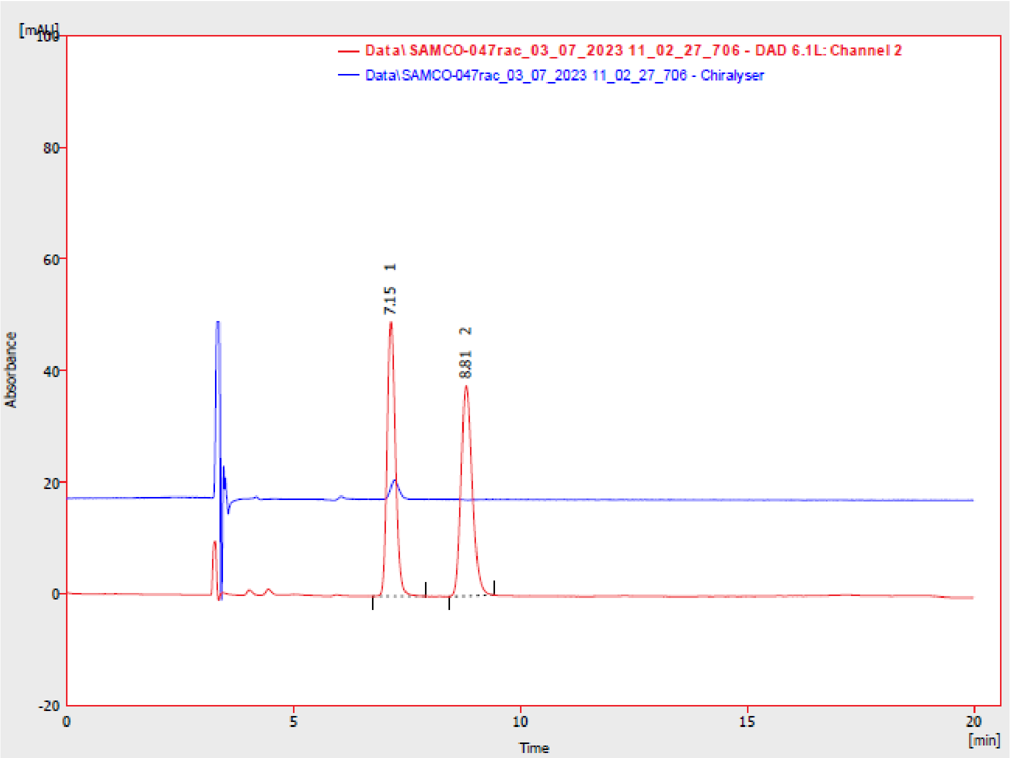

Result Table (Uncal - Data\SAMCO-047rac_03_07_2023 11_02_27_706 - DAD 6.1L: Channel 2)

	Reten. Time [min]	Area [mAU.s]	Height [mAU]	Area [%]	Height [%]	W05 [min]	PDA Peak Purity	Compound Name
1	7.148	622.911	49.181	49.0	56.6	0.19	410	
2	8.808	648.546	37.736	51.0	43.4	0.27	546	
	Total	1271.457	86.918	100.0	100.0			

Figure S7. HPLC of *rac*-**2A** (red: UV detector (350 nm), blue: downstream polarimetric detector).

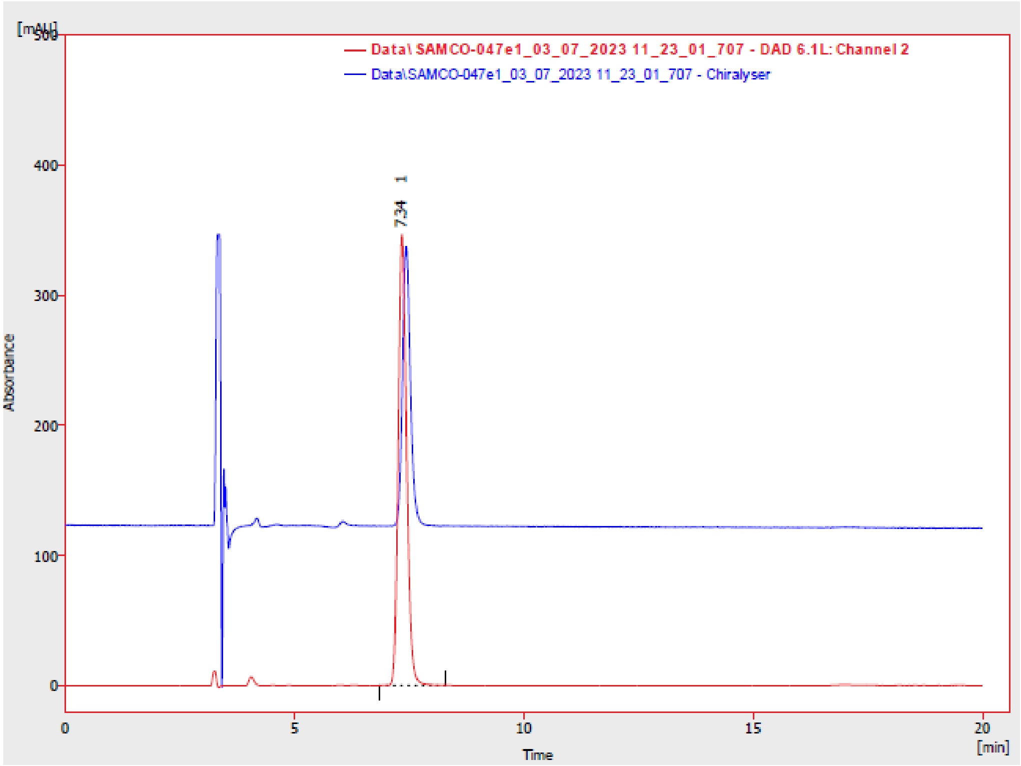

Figure S8. HPLC of (+)-(*P*)-**2A** (red: UV detector (350 nm), blue: downstream polarimetric detector).

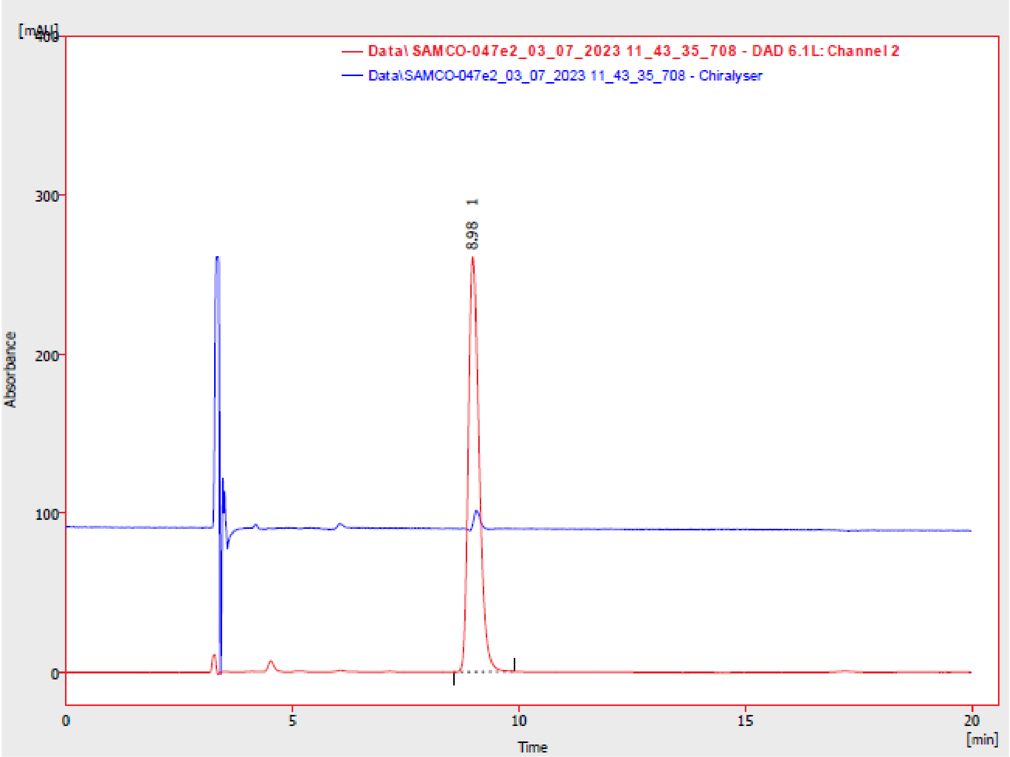

Figure S9. HPLC of (-)-(*M*)-**2A** (red: UV detector (350 nm), blue: downstream polarimetric detector).

Chiral resolution of 3A

Chiral HPLC separation: *Anal*.: Chiralpak IE (250 x 4.6 mm, 5 mm, DAICEL), toluene, 1% IPA @ 1.0 mL/min., 35 °C, t_R(+) = 6.2 min. (>99% *ee*), t_R(-) = 7.0 min. (>99% *ee*); *Semiprep*.: CHIRALPAK® IE (250 x 20 mm, 5 µm, DAICEL), toluene, 0.5% IPA @20 mL/min.

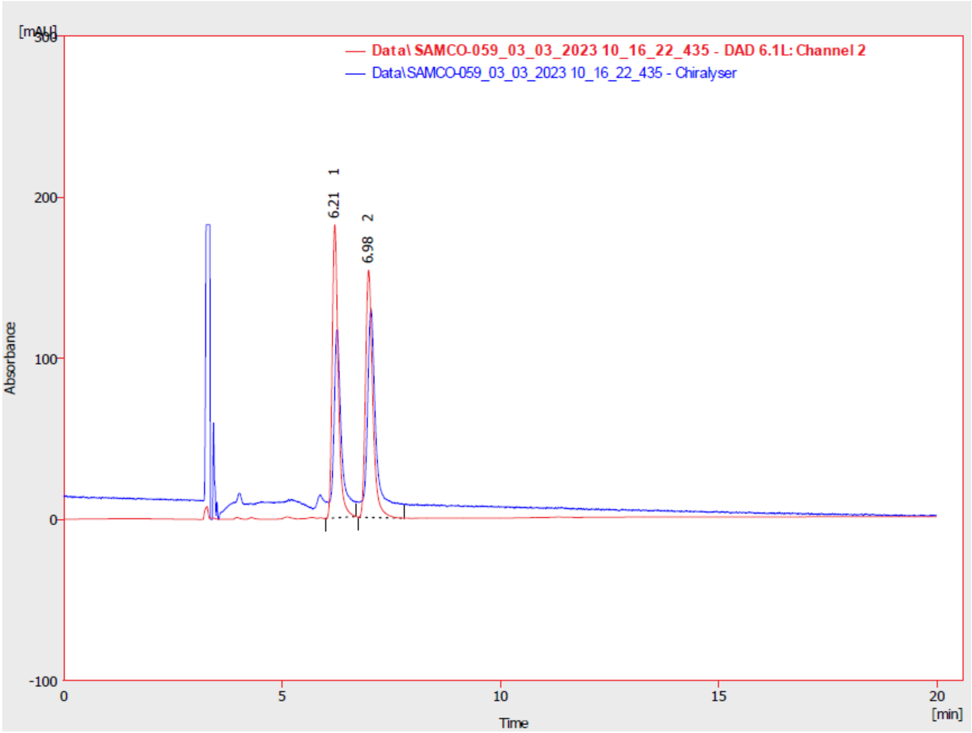

Result Table (Uncal - Data|SAMCO-059_03_03_2023 10_16_22_435 - DAD 6.1L: Channel 2)

	Reten. Time [min]	Area [mAU.s]	Height [mAU]	Area [%]	Height [%]	W05 [min]	PDA Peak Purity	Compound Name
1	6.208	1757.570	181.920	49.8	54.2	0.14	687	
2	6.983	1771.420	153.465	50.2	45.8	0.17	632	
	Total	3528.990	335.384	100.0	100.0			

Figure S10. HPLC of *rac*-**3A** (red: UV detector (350 nm), blue: downstream polarimetric detector).

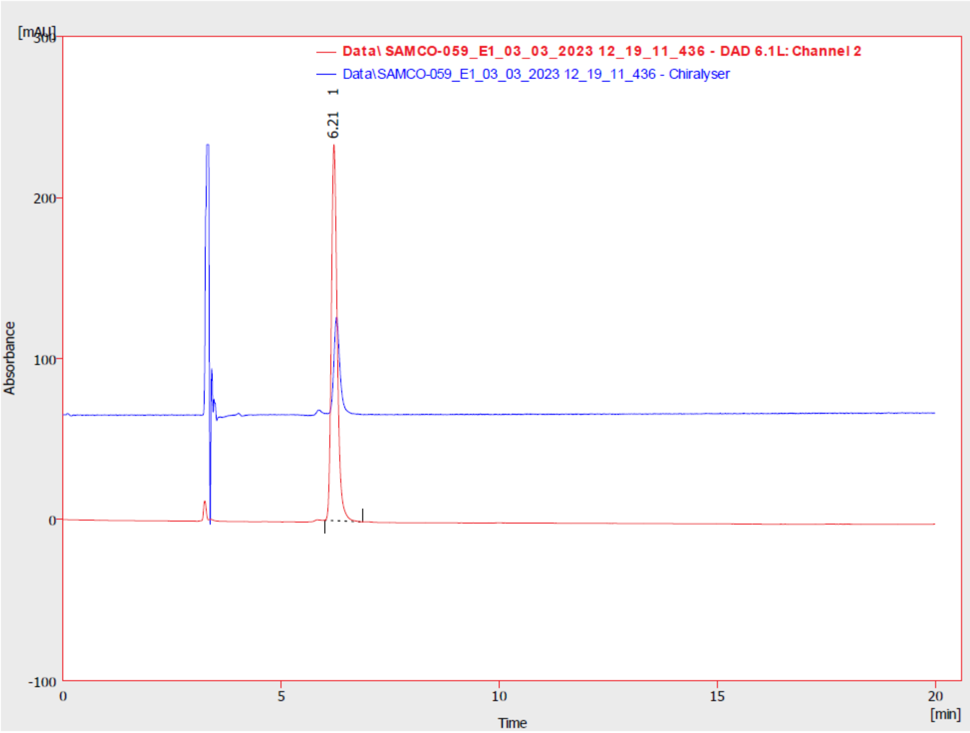

Figure S11. HPLC of (+)-(*P*)-**3A** (red: UV detector (350 nm), blue: downstream polarimetric detector).

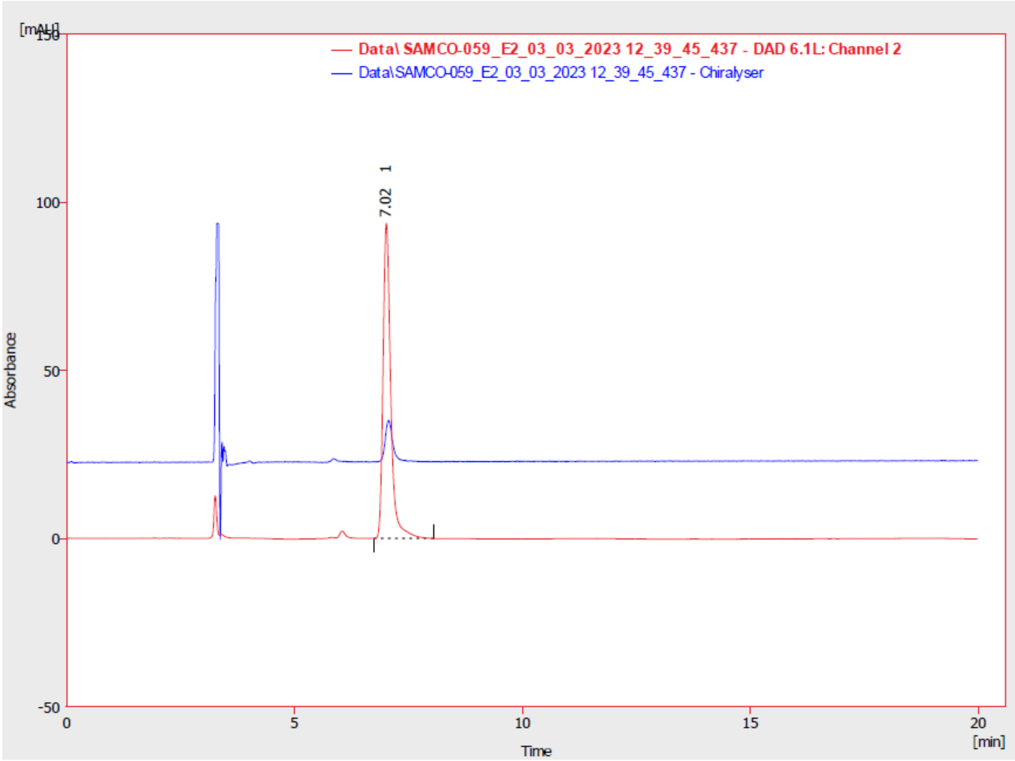

Figure S12. HPLC of (-)-(*M*)-**3A** (red: UV detector (350 nm), blue: downstream polarimetric detector).

Chiral resolution of 4A

Chiral HPLC separation: *Anal.*: Chiralpak IE (250 x 4.6 mm, 5 mm, DAICEL), toluene, 1% IPA @ 1.0 mL/min., 35 °C, t_R(+) = 6.1 min. (>99% *ee*), t_R(-) = 7.0 min. (>99% *ee*); *Semiprep.*: CHIRALPAK® IE (250 x 20 mm, 5 µm, DAICEL), toluene, 0.5% IPA @20 mL/min.

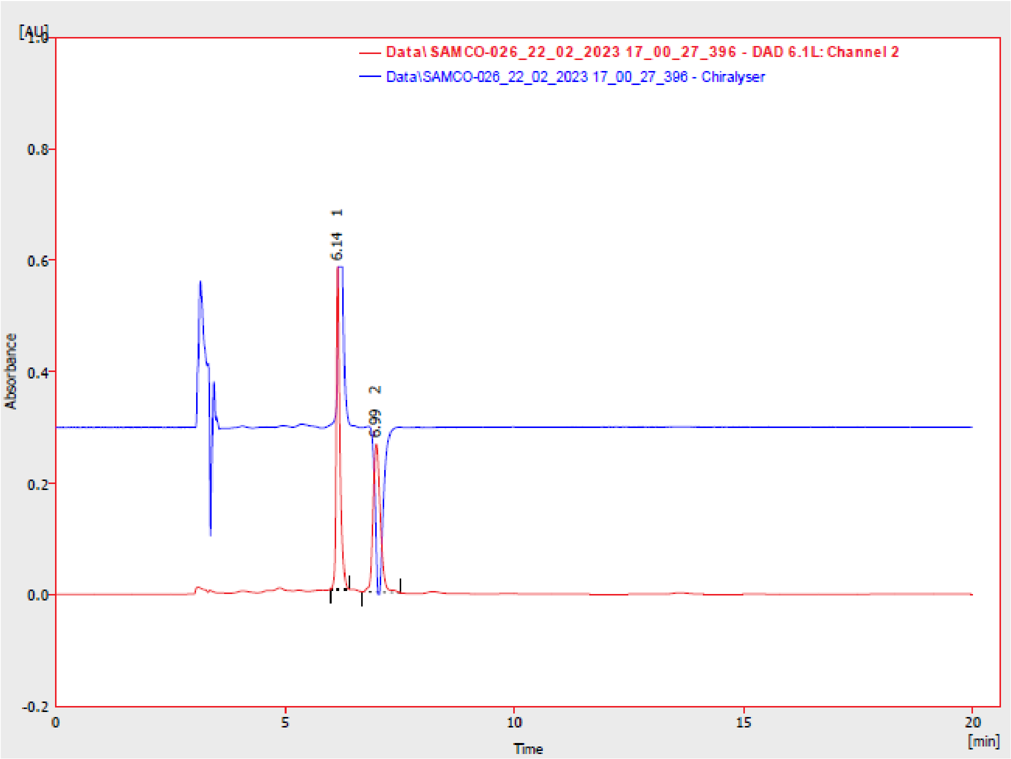

Result Table (Uncal - Data\SAMCO-026_22_02_2023 17_00_27_396 - DAD 6.1L: Channel 2)

	Reten. Time [min]	Area [mAU.s]	Height [mAU]	Area [%]	Height [%]	W05 [min]	PDA Peak Purity	Compound Name
1	6.145	3293.652	579.047	53.3	68.5	0.08	840	
2	6.987	2887.137	265.806	46.7	31.5	0.16	750	
	Total	6180.788	844.853	100.0	100.0			

Figure S13. HPLC of *rac*-**4A** (red: UV detector (350 nm), blue: downstream polarimetric detector).

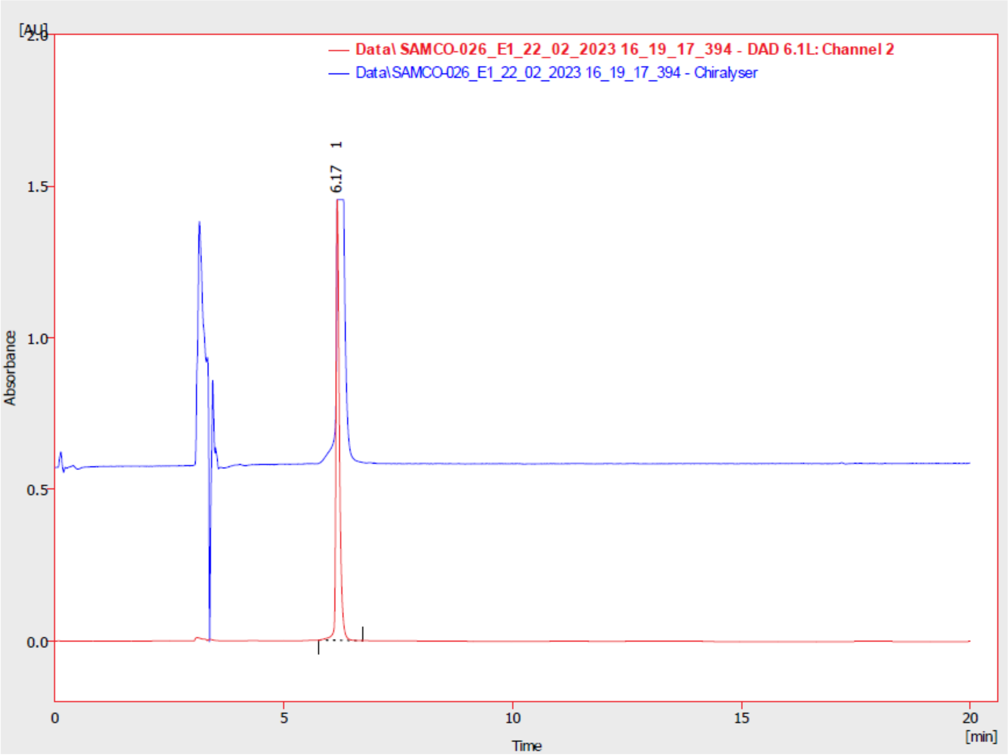

Figure S14. HPLC of (+)-(*P*)-**4A** (red: UV detector (350 nm), blue: downstream polarimetric detector).

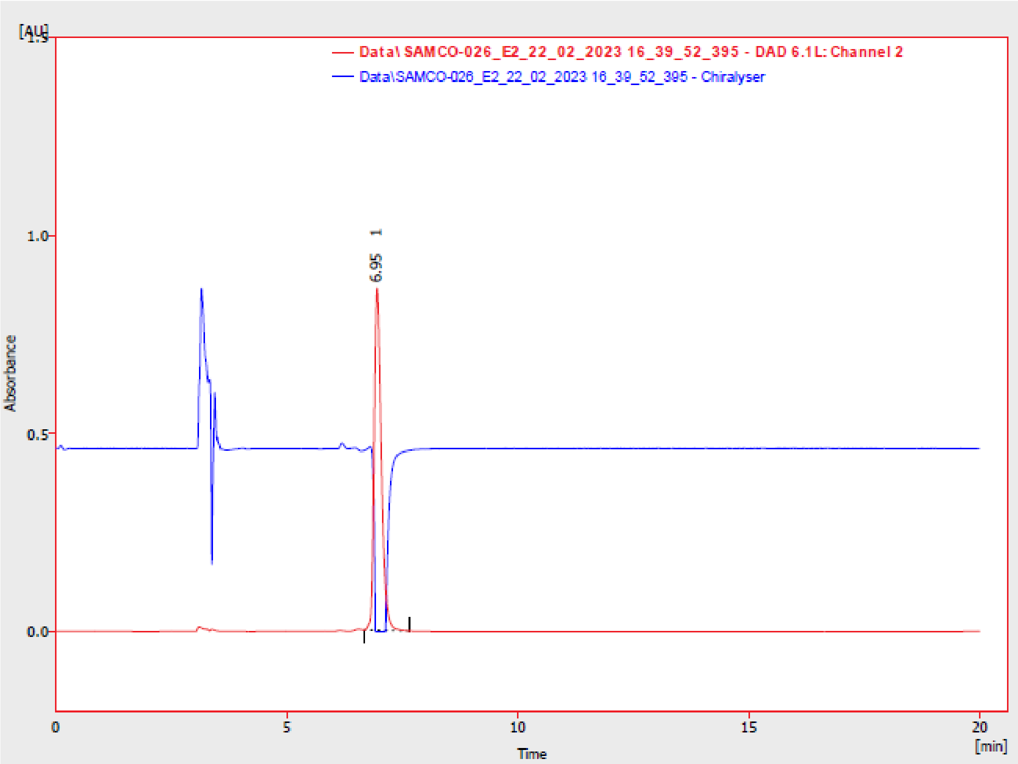

Figure S15. HPLC of (-)-(*M*)-**4A** (red: UV detector (350 nm), blue: downstream polarimetric detector).

Chiral resolution of 5A

Chiral HPLC separation: *Anal*.: Chiralpak IE (250 x 4.6 mm, 5 mm, DAICEL), toluene, 1% IPA @ 1.0 mL/min., 35 °C, t_R(+) = 8.5 min. (>99% *ee*), t_R(-) = 10.1 min. (>99% *ee*); *Semiprep*.: CHIRALPAK® IE (250 x 20 mm, 5 µm, Chiral Technologies), toluene, 0.5% IPA @20 mL/min.

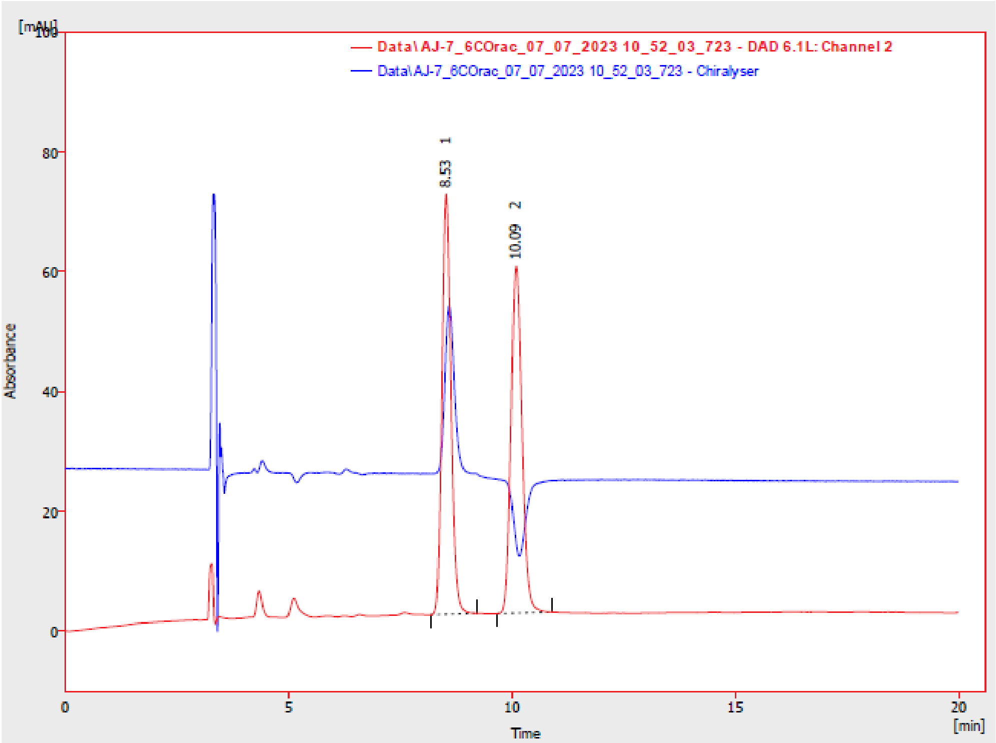

Result Table (Uncal - Data\AJ-7_6COrac_07_07_2023 10_52_03_723 - DAD 6.1L: Channel 2)

	Reten. Time [min]	Area [mAU.s]	Height [mAU]	Area [%]	Height [%]	W05 [min]	PDA Peak Purity	Compound Name
1	8.525	995.700	70.116	50.2	54.8	0.22	463	
2	10.093	986.576	57.903	49.8	45.2	0.26	449	
	Total	1982.276	128.019	100.0	100.0			

Figure S16. HPLC of *rac*-**5A** (red: UV detector (350 nm), blue: downstream polarimetric detector).

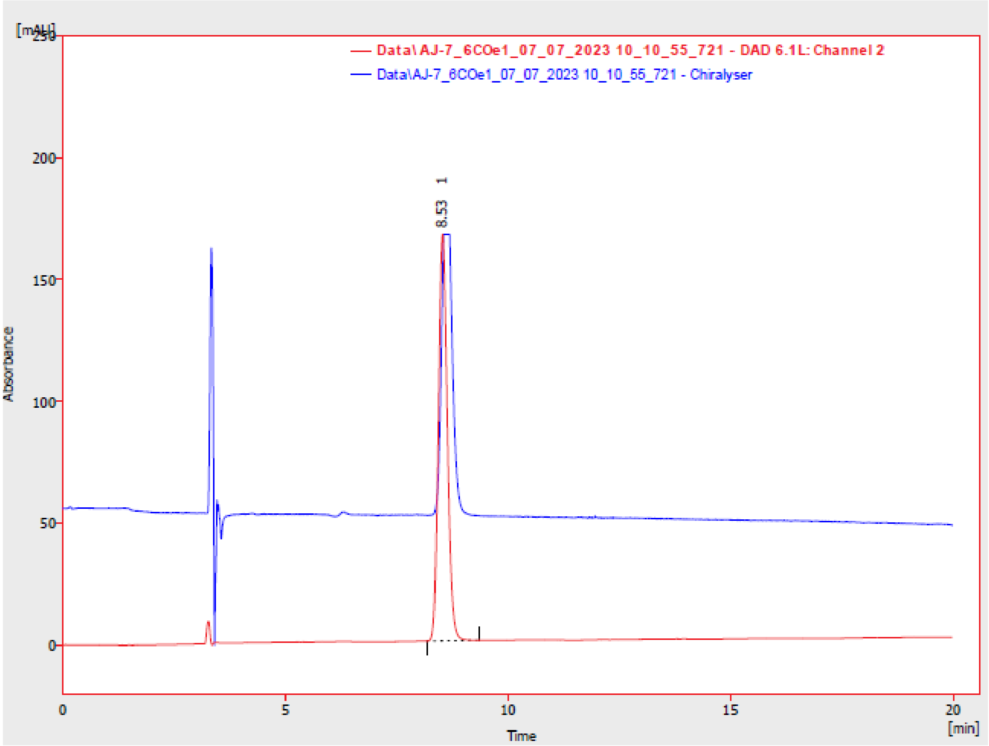

Figure S17. HPLC of (+)-(*P*)-**5A** (red: UV detector (350 nm), blue: downstream polarimetric detector).

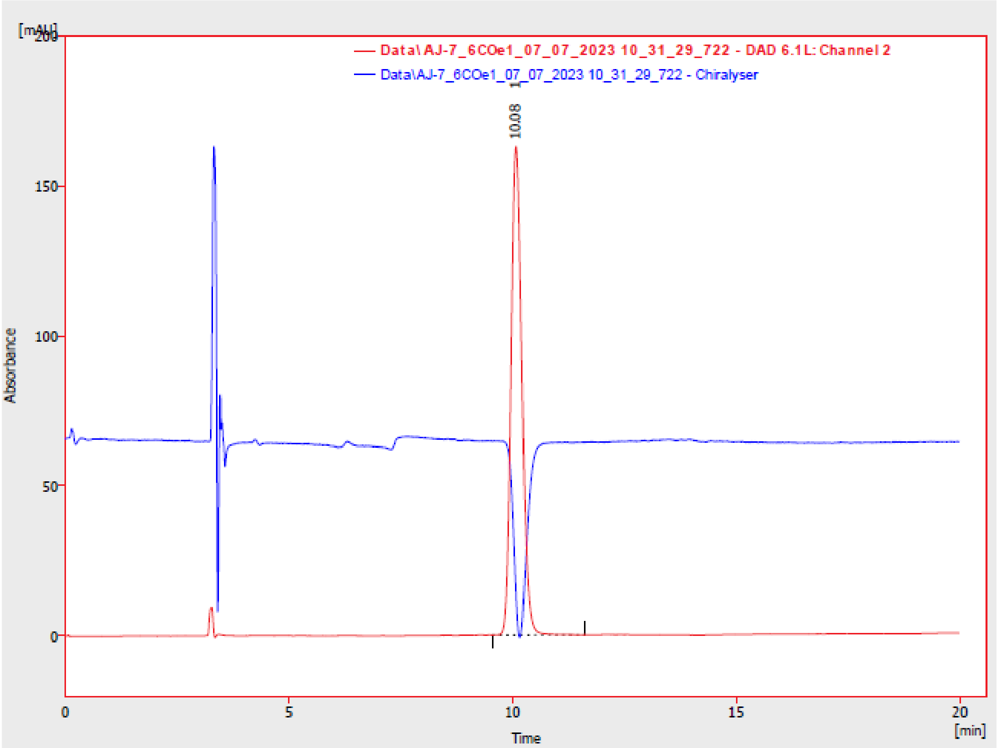

Figure S18. HPLC of (-)-(*M*)-**5A** (red: UV detector (350 nm), blue: downstream polarimetric detector).

Chiral resolution of 6A

Chiral HPLC separation: *Anal.*: Chiralpak IE (250 x 4.6 mm, 5 mm, DAICEL), toluene, 1% IPA @ 1.0 mL/min., 35 °C, $t_R(+)$ = 8.8 min. (>99% *ee*), $t_R(-)$ = 12.5 min. (>99% *ee*); *Semiprep.*: CHIRALPAK® IE (250 x 20 mm, 5 µm, Chiral Technologies), toluene, 0.5% IPA @20 mL/min.

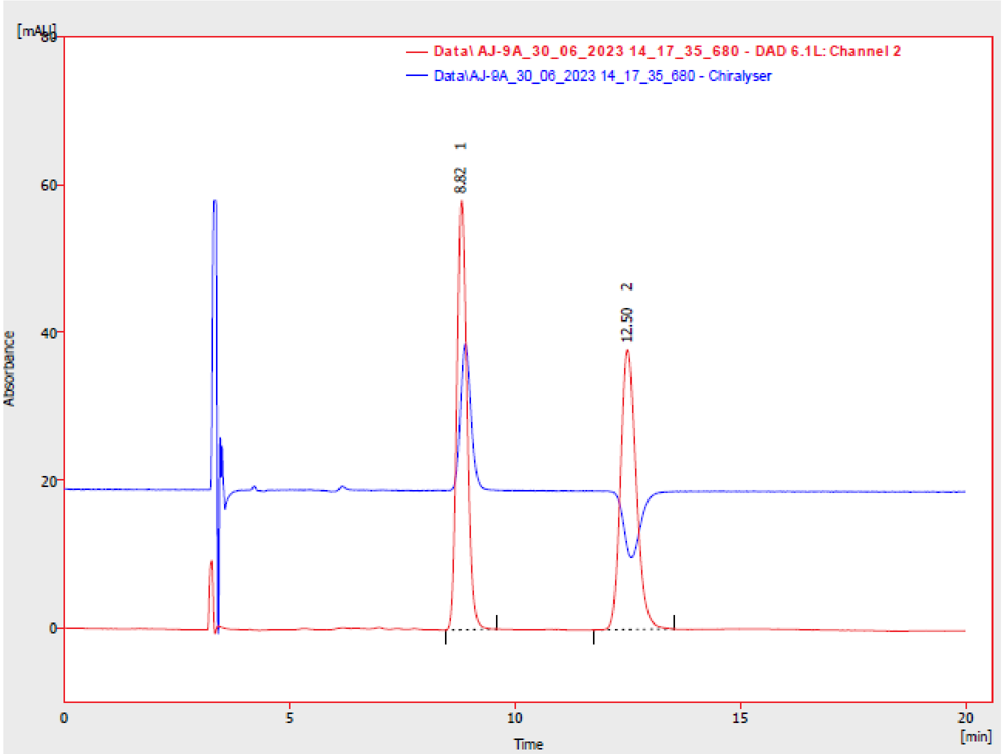

Result Table (Uncal - Data\AJ-9A_30_06_2023 14_17_35_680 - DAD 6.1L: Channel 2)

	Reten. Time [min]	Area [mAU.s]	Height [mAU]	Area [%]	Height [%]	W05 [min]	PDA Peak Purity	Compound Name
1	8.818	909.797	58.068	49.9	60.5	0.24	770	
2	12.498	911.965	37.874	50.1	39.5	0.37	821	
	Total	1821.762	95.943	100.0	100.0			

Figure S19. HPLC of *rac*-**6A** (red: UV detector (350 nm), blue: downstream polarimetric detector).

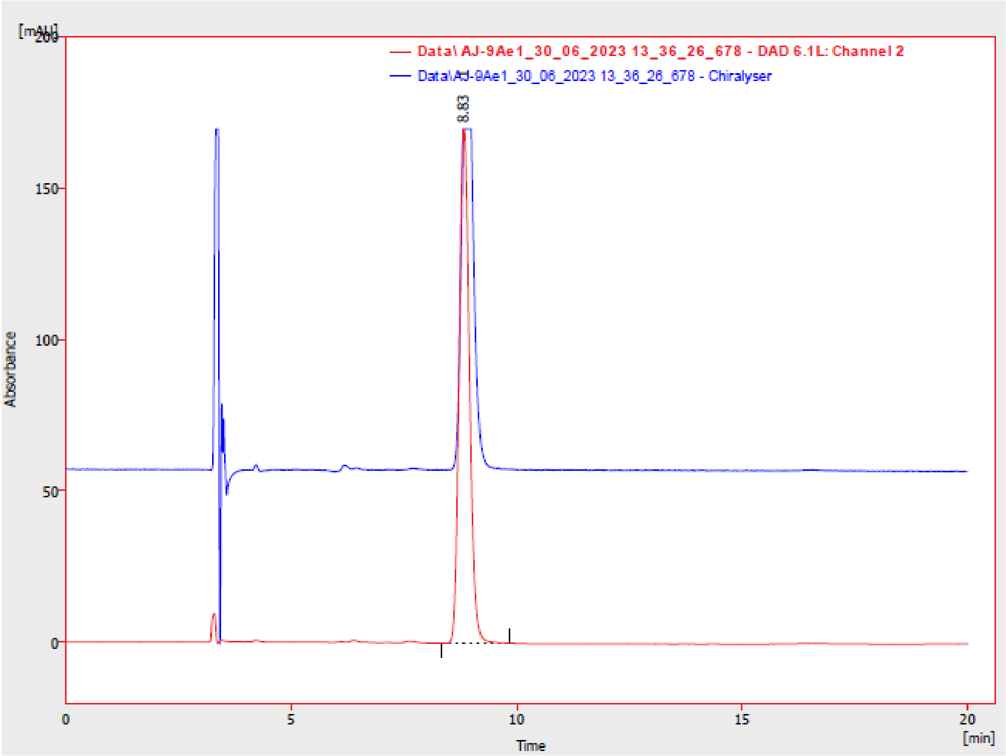

Figure S20. HPLC of (+)-(*P*)-**6A** (red: UV detector (350 nm), blue: downstream polarimetric detector).

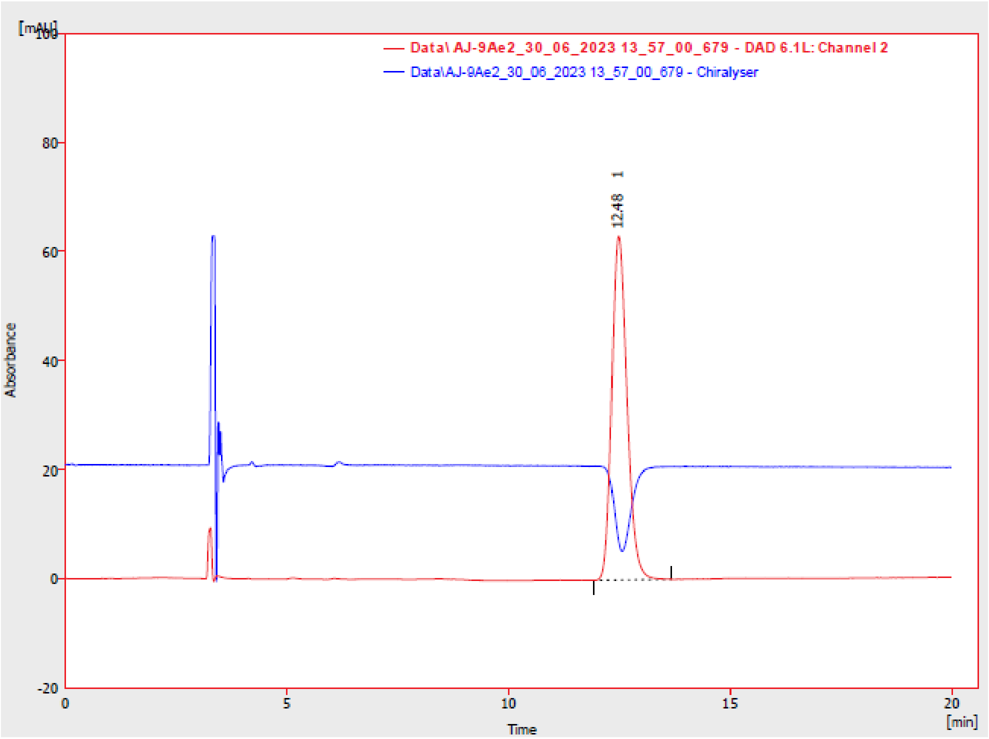

Figure S21. HPLC of (-)-(*M*)-**6A** (red: UV detector (350 nm), blue: downstream polarimetric detector).

Chiral resolution of 6F

Chiral HPLC separation: *Anal*.: Chiralpak IE (250 x 4.6 mm, 5 mm, DAICEL), toluene, 1% IPA @ 1.0 mL/min., 35 °C, $t_R(+)$ = 5.3 min. (>99% *ee*), $t_R(-)$ = 7.4 min. (>99% *ee*); *Semiprep*.: CHIRALPAK® IE (250 x 20 mm, 5 µm, Chiral Technologies), toluene, 0.5% IPA @20 mL/min.

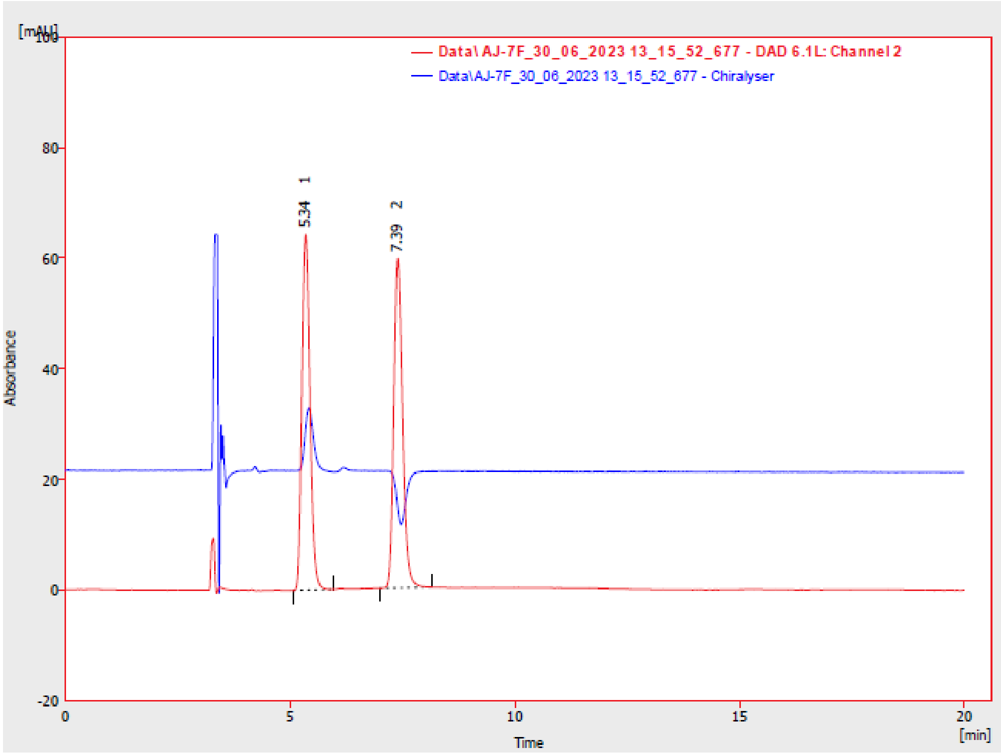

Result Table (Uncal - Data\AJ-7F_30_06_2023 13_15_52_677 - DAD 6.1L: Channel 2)

	Reten. Time [min]	Area [mAU.s]	Height [mAU]	Area [%]	Height [%]	W05 [min]	PDA Peak Purity	Compound Name
1	5.343	810.851	64.310	50.1	51.9	0.20	902	
2	7.393	808.579	59.572	49.9	48.1	0.21	890	
	Total	1619.430	123.882	100.0	100.0			

Figure S22. HPLC of *rac*-**6F** (red: UV detector (350 nm), blue: downstream polarimetric detector).

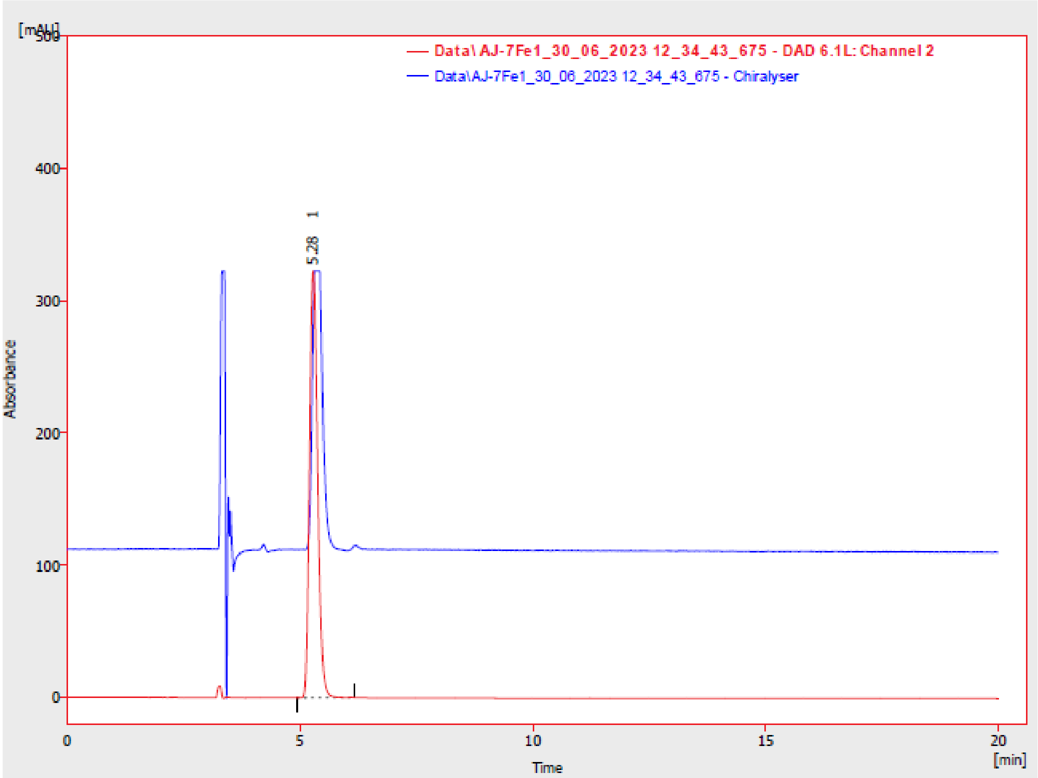

Figure S23. HPLC of (+)-(*P*)-**6F** (red: UV detector (350 nm), blue: downstream polarimetric detector).

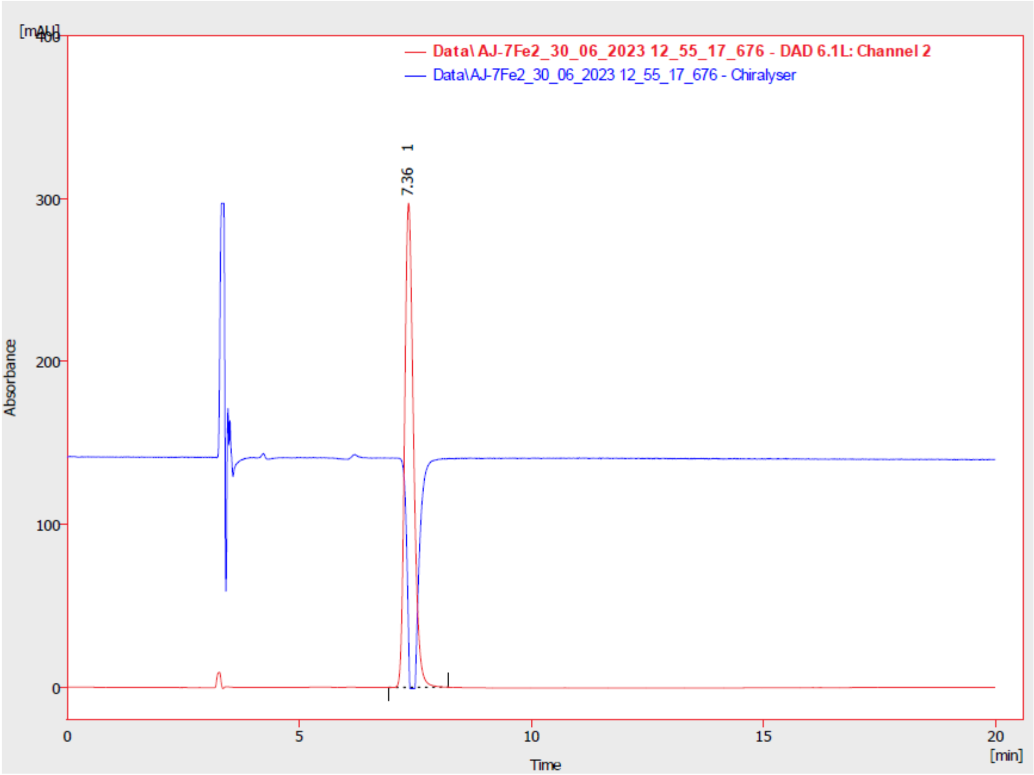

Figure S24. HPLC of (-)-(*M*)-**6F** (red: UV detector (350 nm), blue: downstream polarimetric detector).

Chiral resolution of 7A

Chiral HPLC separation: *Anal*.: Chiralpak IE (250 x 4.6 mm, 5 mm, DAICEL), toluene, 1% IPA @ 1.0 mL/min., 35 °C, $t_R(+)$ = 7.9 min. (>99% *ee*), $t_R(-)$ = 10.0 min. (>99% *ee*); *Semiprep*.: CHIRALPAK® IE (250 x 20 mm, 5 µm, Chiral Technologies), toluene, 0.5% IPA @20 mL/min.

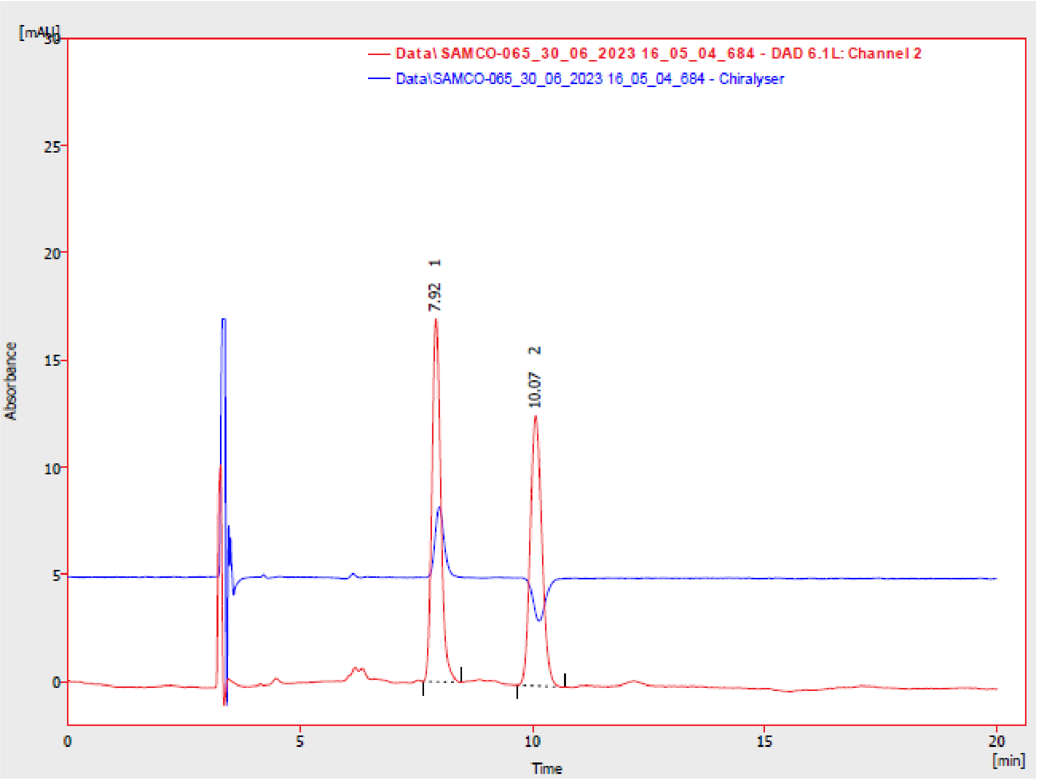

Result Table (Uncal - Data|SAMCO-065_30_06_2023 16_05_04_684 - DAD 6.1L: Channel 2)

	Reten. Time [min]	Area [mAU.s]	Height [mAU]	Area [%]	Height [%]	W05 [min]	PDA Peak Purity	Compound Name
1	7.923	223.916	16.930	50.6	57.3	0.20	915	
2	10.067	218.705	12.601	49.4	42.7	0.27	942	
	Total	442.621	29.531	100.0	100.0			

Figure S25. HPLC of *rac*-**7A** (red: UV detector (350 nm), blue: downstream polarimetric detector).

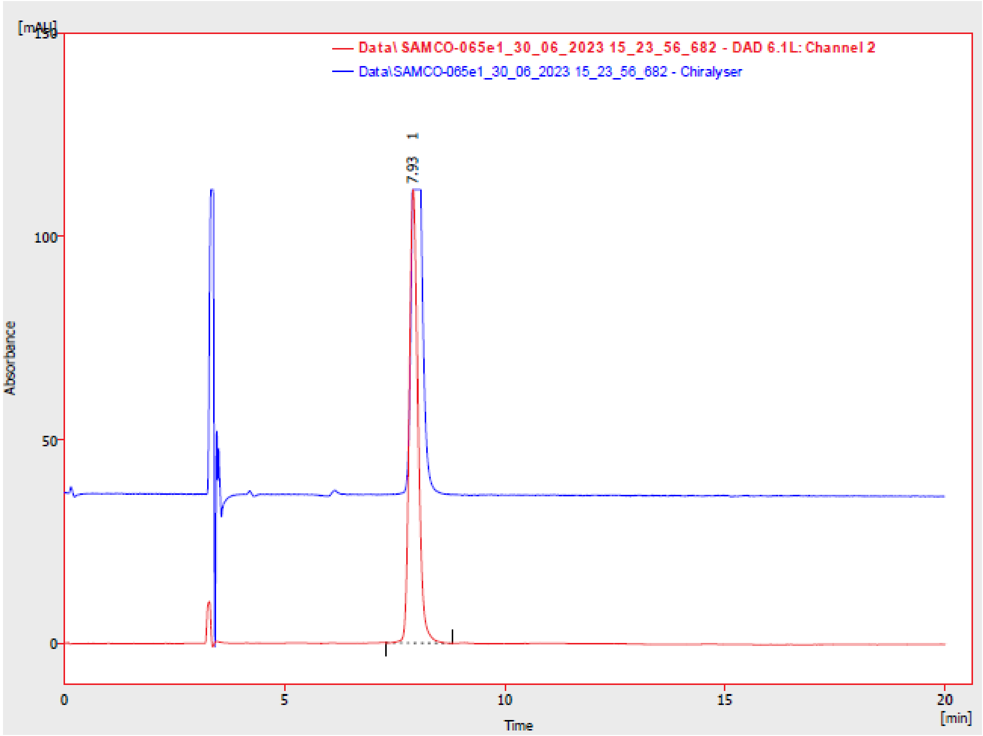

Figure S26. HPLC of (+)-(*P*)-**7A** (red: UV detector (350 nm), blue: downstream polarimetric detector).

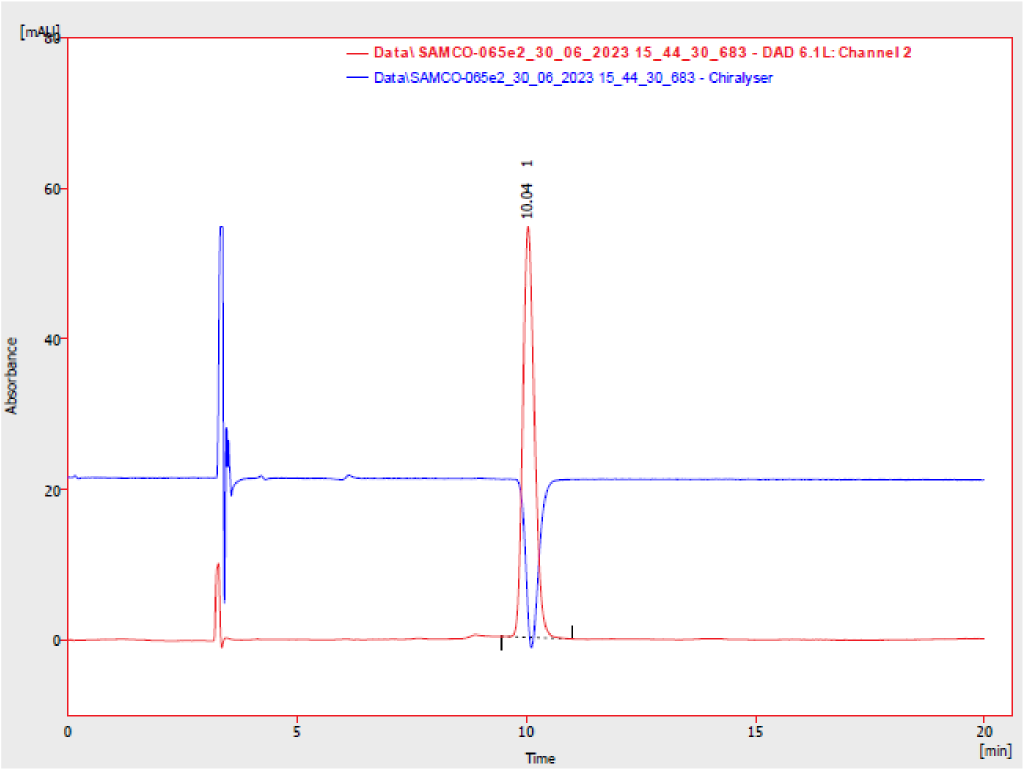

Figure S27. HPLC of (-)-(*M*)-**7A** (red: UV detector (350 nm), blue: downstream polarimetric detector).

Chiral resolution of 8A

Chiral HPLC separation: *Anal*.: Chiralpak IE (250 x 4.6 mm, 5 mm, DAICEL), toluene, 1% IPA @ 1.0 mL/min., 35 °C, t_R(+) = 7.6 min. (>99% *ee*), t_R(-) = 10.4 min. (>99% *ee*); *Semiprep*.: CHIRALPAK® IE (250 x 20 mm, 5 µm, Chiral Technologies), toluene, 0.5% IPA @20 mL/min.

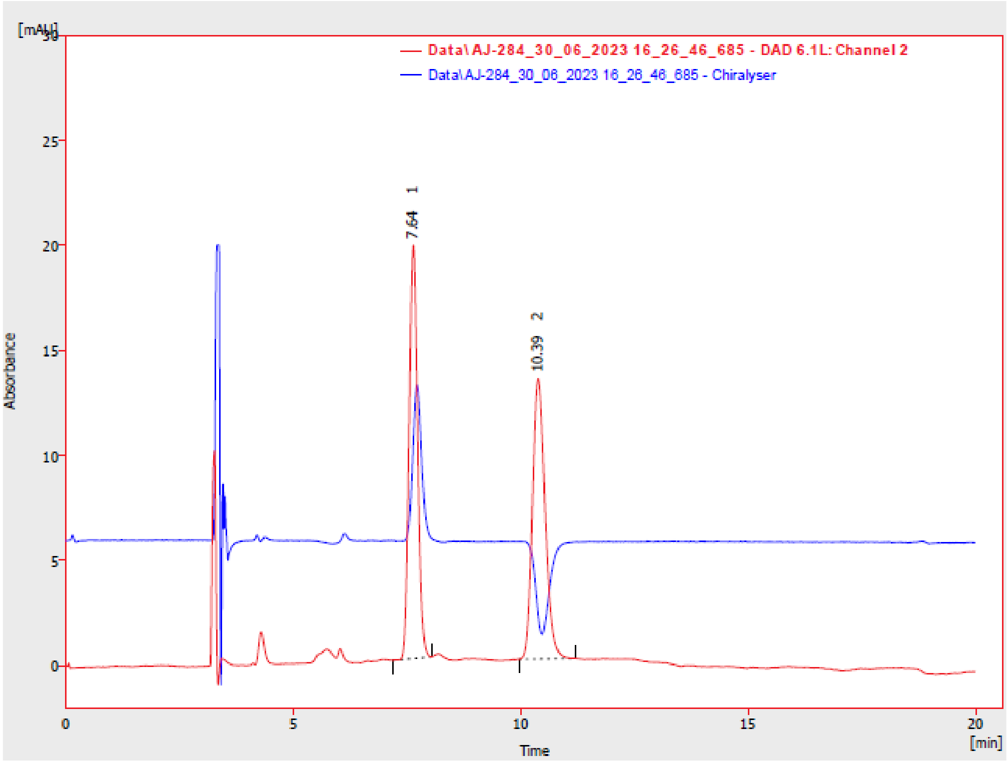

Result Table (Uncal - Data\AJ-284_30_06_2023 16_26_46_685 - DAD 6.1L: Channel 2)

	Reten. Time [min]	Area [mAU.s]	Height [mAU]	Area [%]	Height [%]	W05 [min]	PDA Peak Purity	Compound Name
1	7.640	255.991	19.681	49.8	59.6	0.20	816	
2	10.388	257.787	13.340	50.2	40.4	0.30	870	
	Total	513.778	33.021	100.0	100.0			

Figure S28. HPLC of *rac*-**8A** (red: UV detector (350 nm), blue: downstream polarimetric detector).

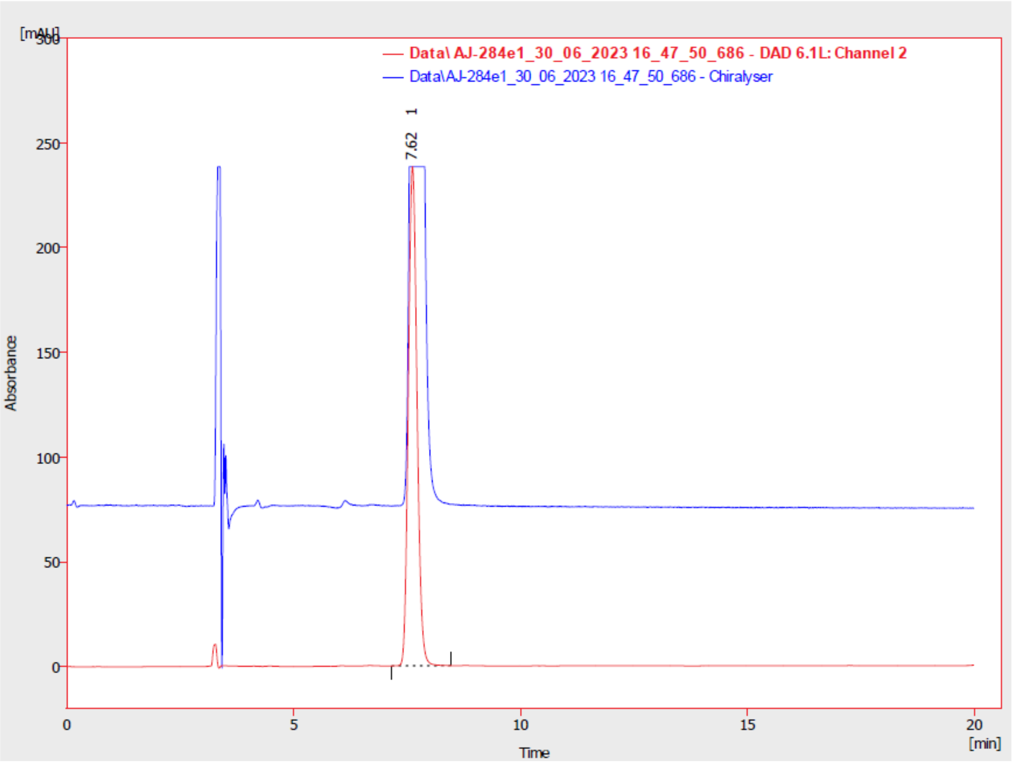

Figure S29. HPLC of (+)-(*P*)-**8A** (red: UV detector (350 nm), blue: downstream polarimetric detector).

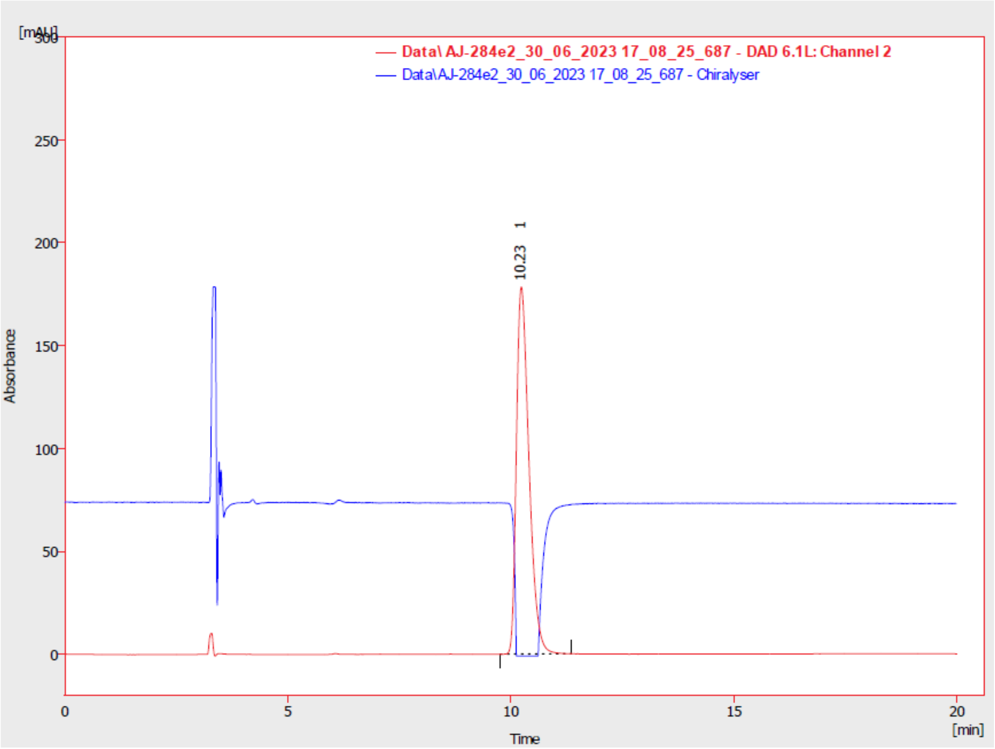

Figure S30. HPLC of (-)-(*M*)-**8A** (red: UV detector (350 nm), blue: downstream polarimetric detector).

^{1}H and ^{13}C NMR spectra of 1–54

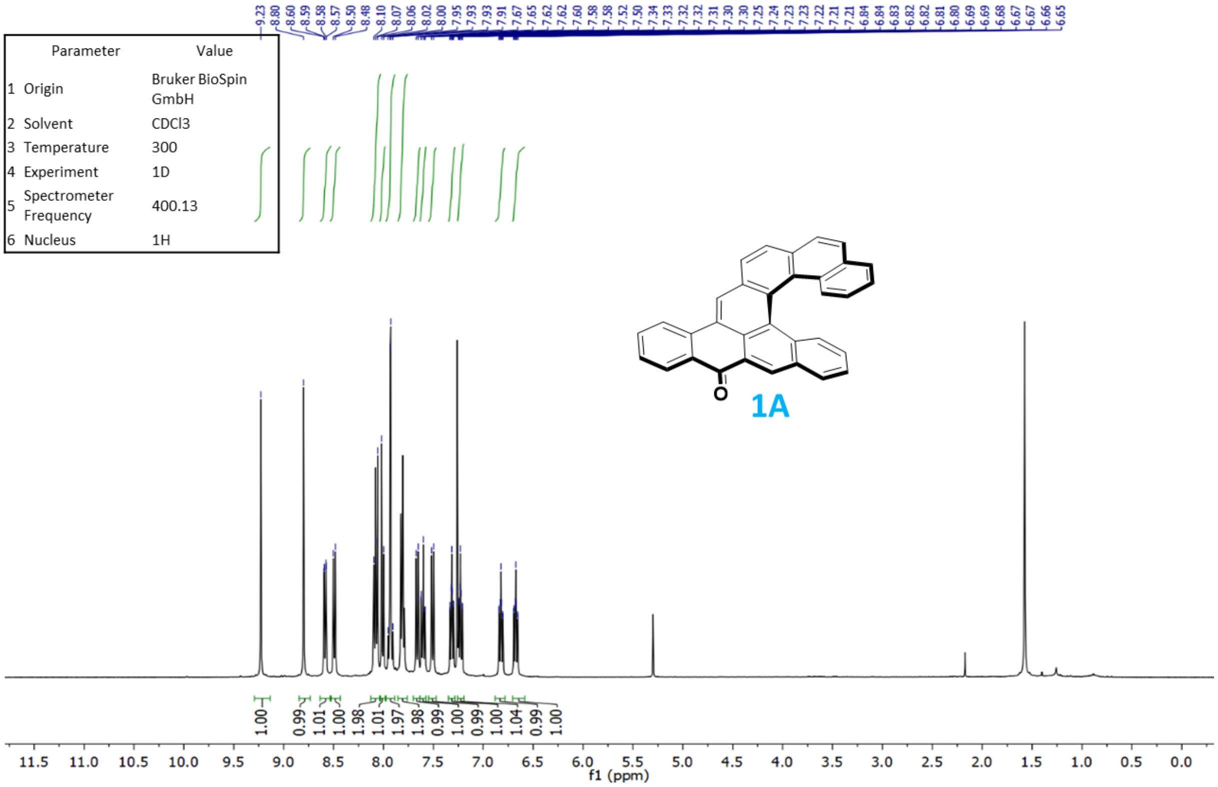

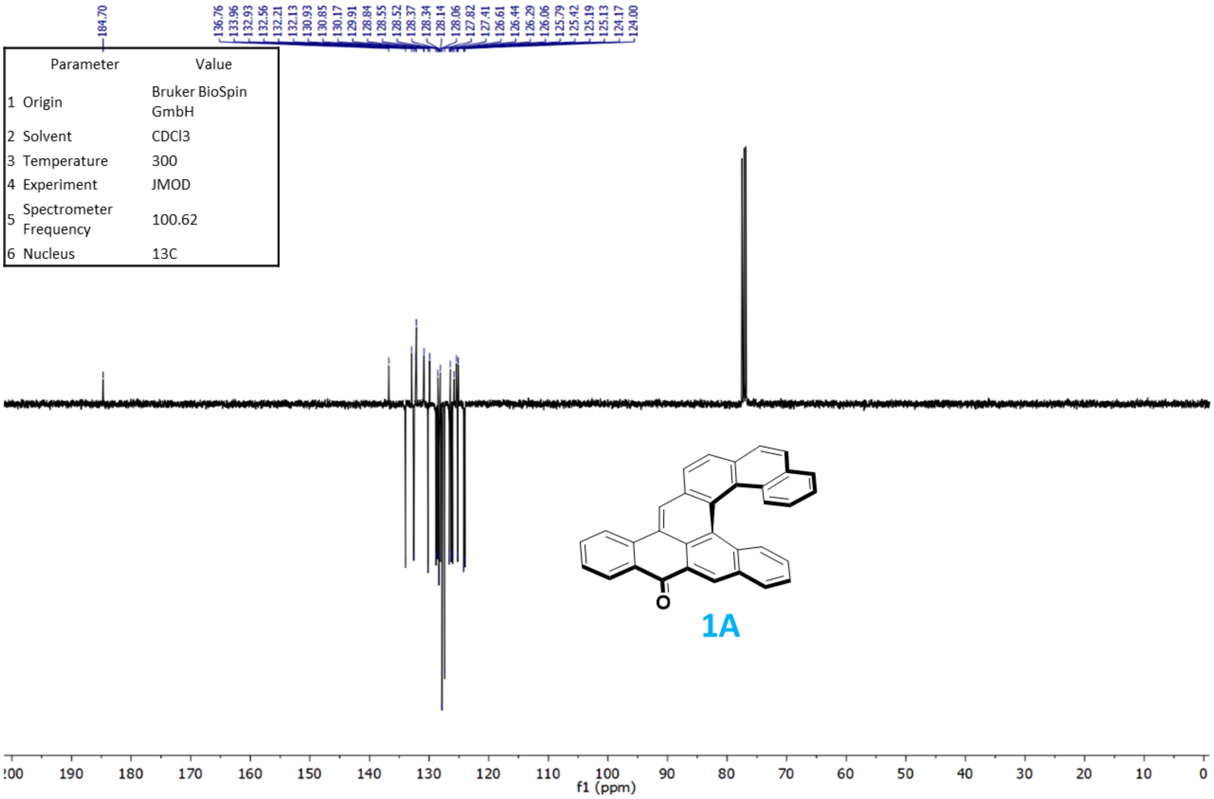

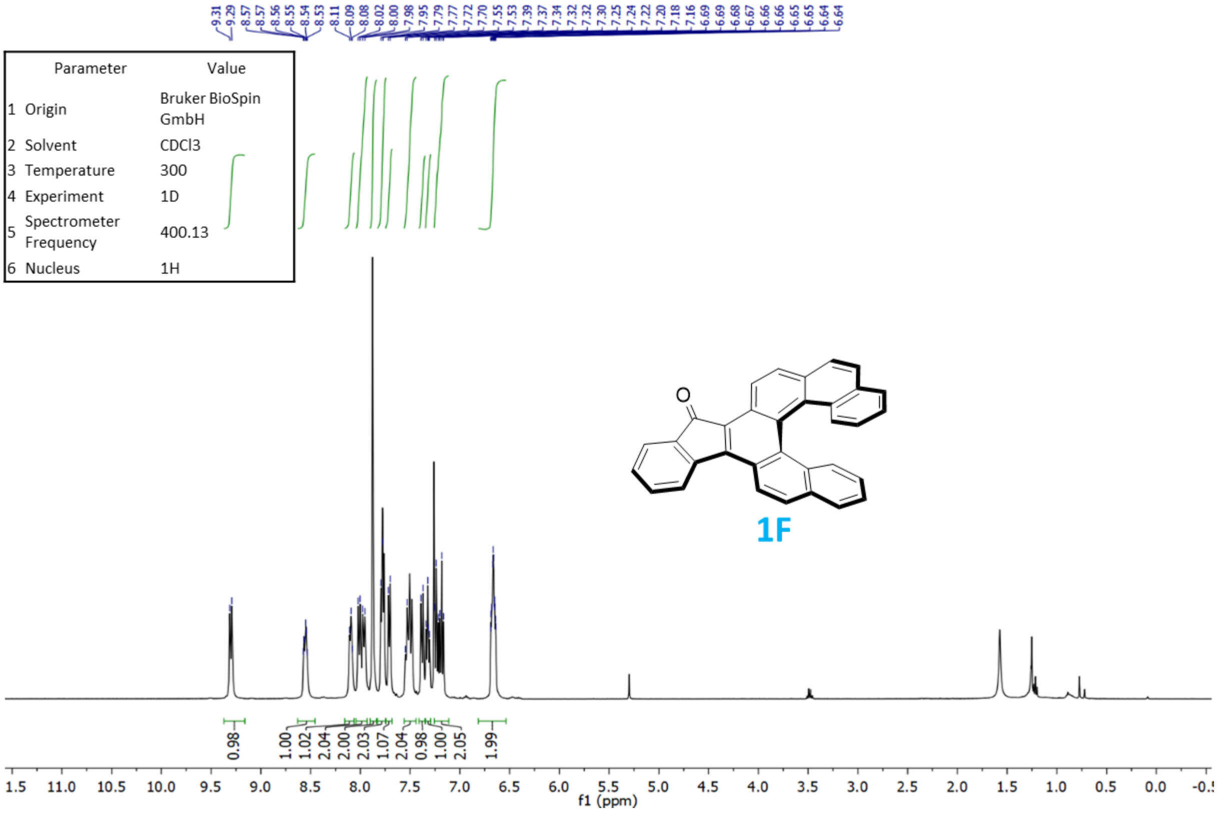

	Parameter	Value
1	Origin	Bruker BioSpin GmbH
2	Solvent	CDCl3
3	Temperature	300
4	Experiment	1D
5	Spectrometer Frequency	400.13
6	Nucleus	1H

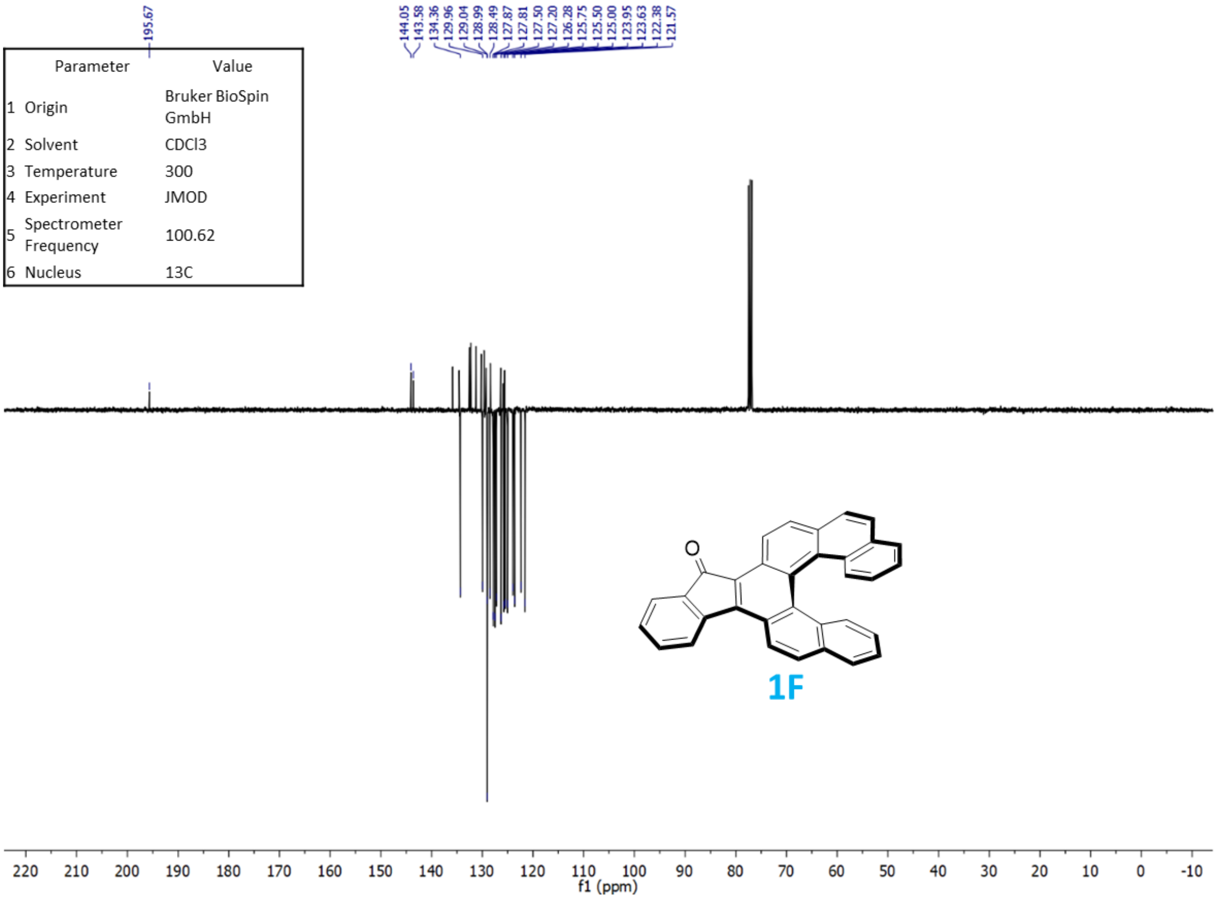

	Parameter	Value
1	Origin	Bruker BioSpin GmbH
2	Solvent	CDCl3
3	Temperature	300
4	Experiment	JMOD
5	Spectrometer Frequency	100.62
6	Nucleus	13C

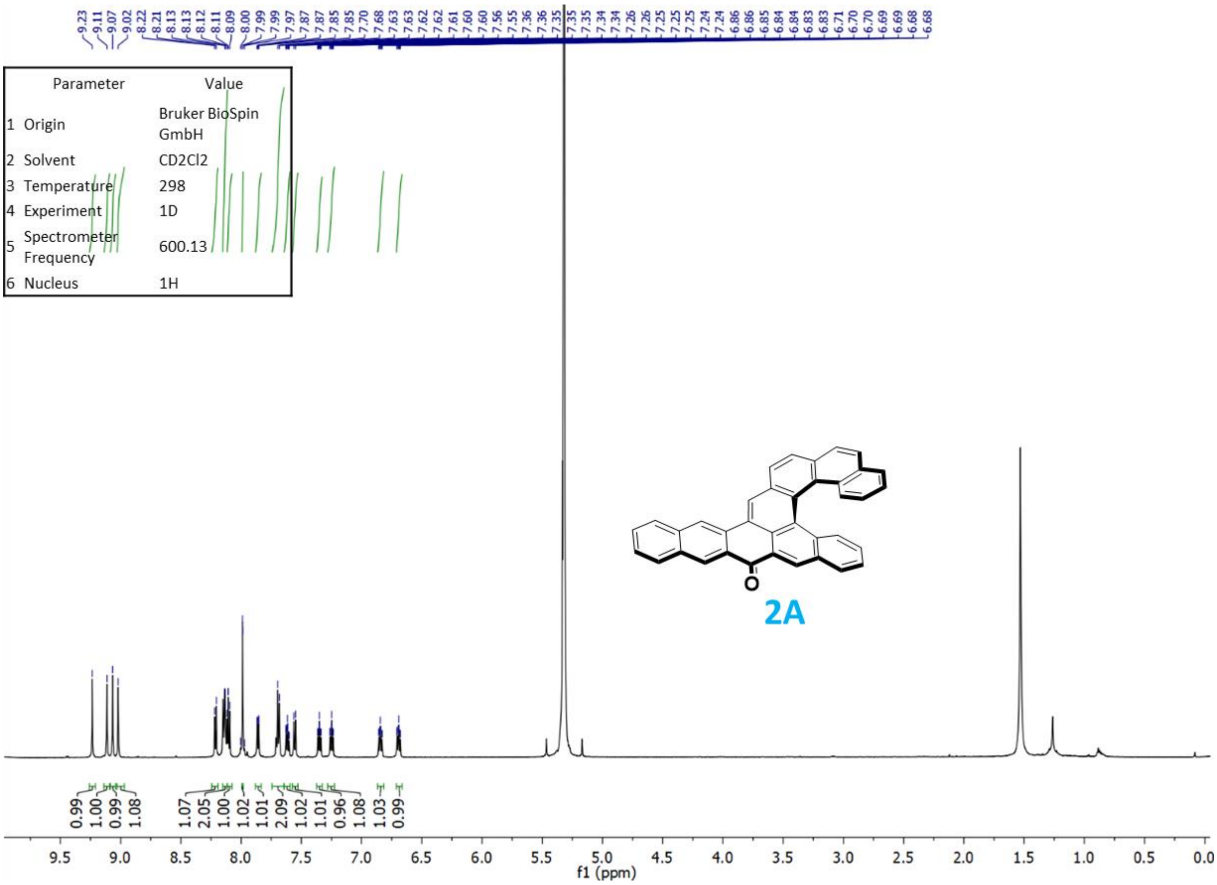

	Parameter	Value
1	Origin	Bruker BioSpin GmbH
2	Solvent	CD2Cl2
3	Temperature	298
4	Experiment	1D
5	Spectrometer Frequency	600.13
6	Nucleus	1H

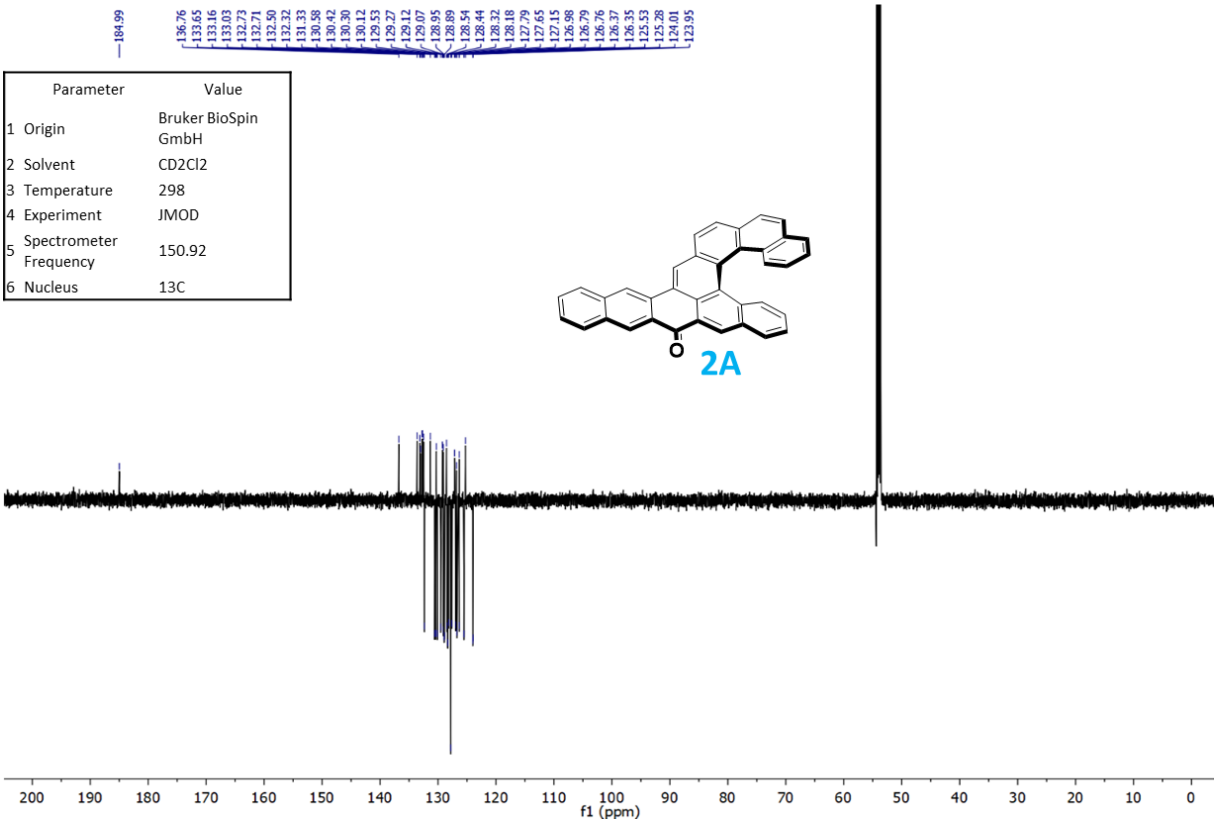

	Parameter	Value
1	Origin	Bruker BioSpin GmbH
2	Solvent	CD2Cl2
3	Temperature	298
4	Experiment	JMOD
5	Spectrometer Frequency	150.92
6	Nucleus	13C

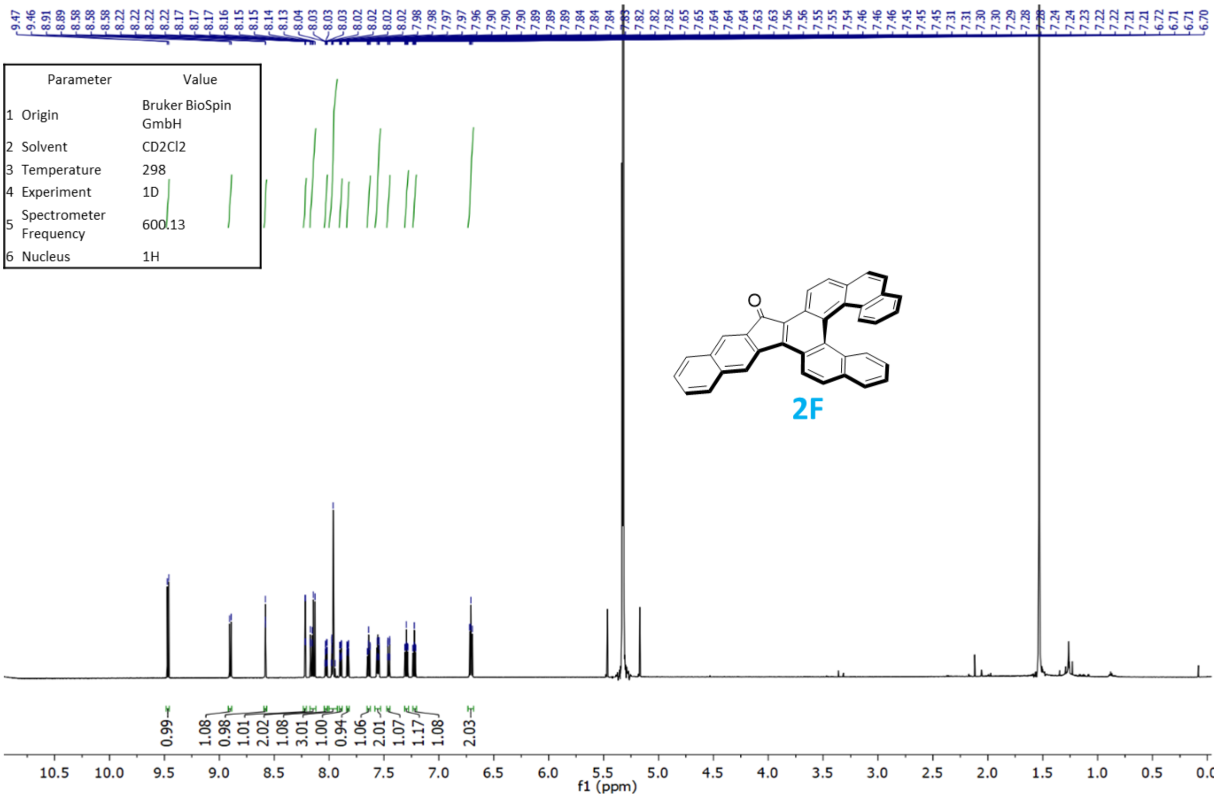
Parameter Value
1 Origin Bruker BioSpin GmbH
2 Solvent CD2Cl2
3 Temperature 298
4 Experiment 1D
5 Spectrometer Frequency 600.13
6 Nucleus 1H
2F
f1 (ppm)

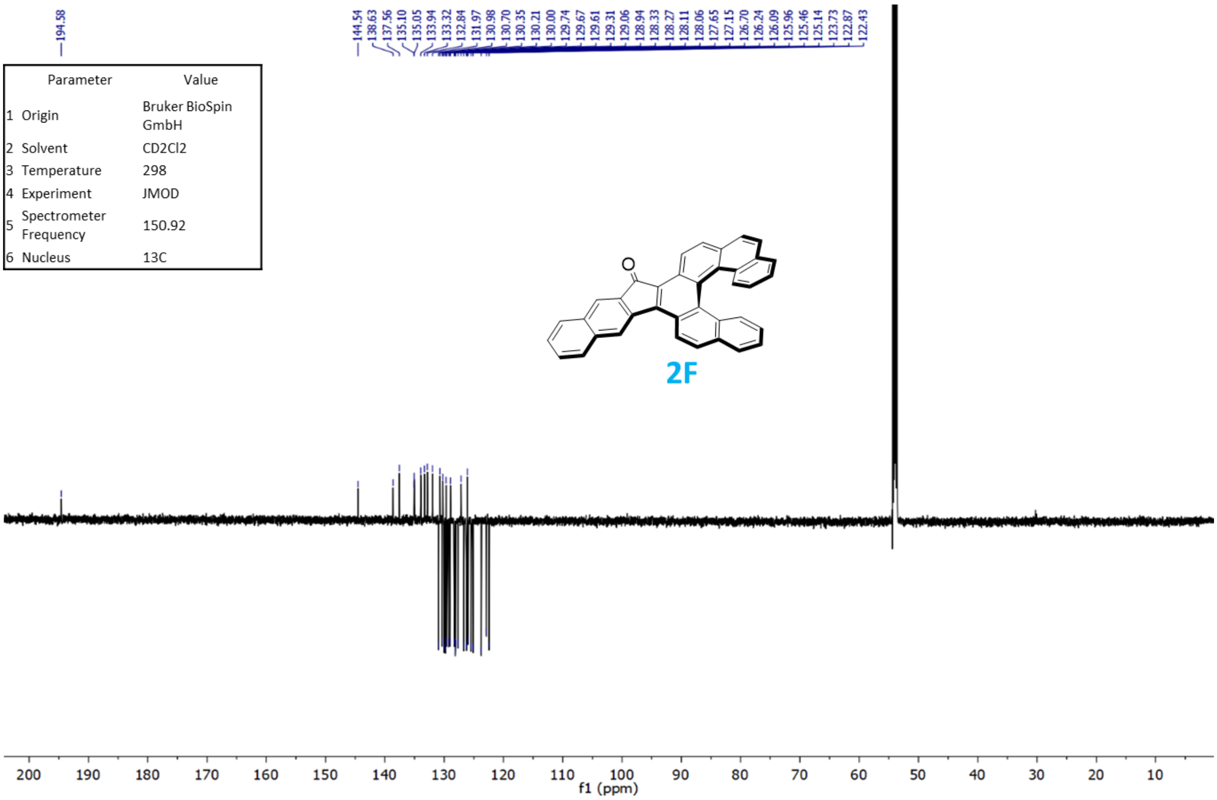
Parameter Value
1 Origin Bruker BioSpin GmbH
2 Solvent CD2Cl2
3 Temperature 298
4 Experiment JMOD
5 Spectrometer Frequency 150.92
6 Nucleus 13C
2F
f1 (ppm)

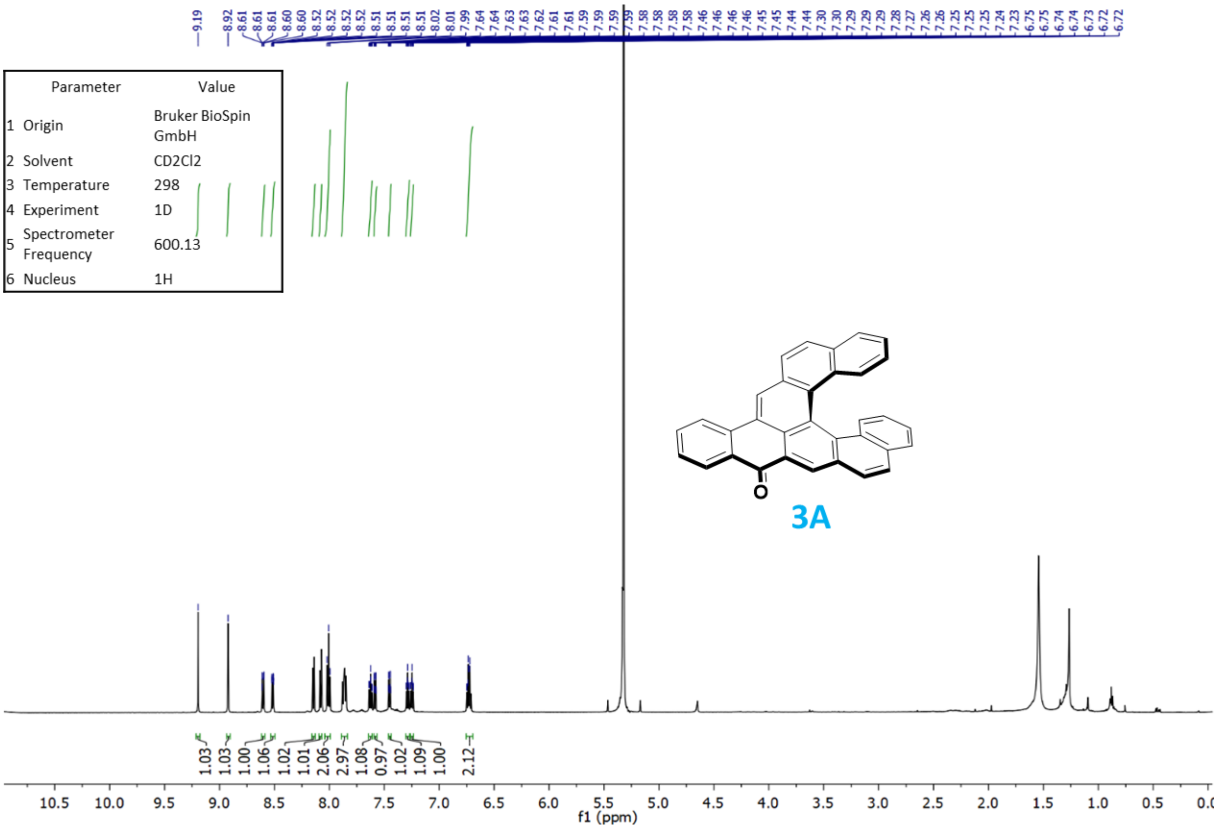
Parameter Value
1 Origin Bruker BioSpin GmbH
2 Solvent CD2Cl2
3 Temperature 298
4 Experiment 1D
5 Spectrometer Frequency 600.13
6 Nucleus 1H
3A
f1 (ppm)

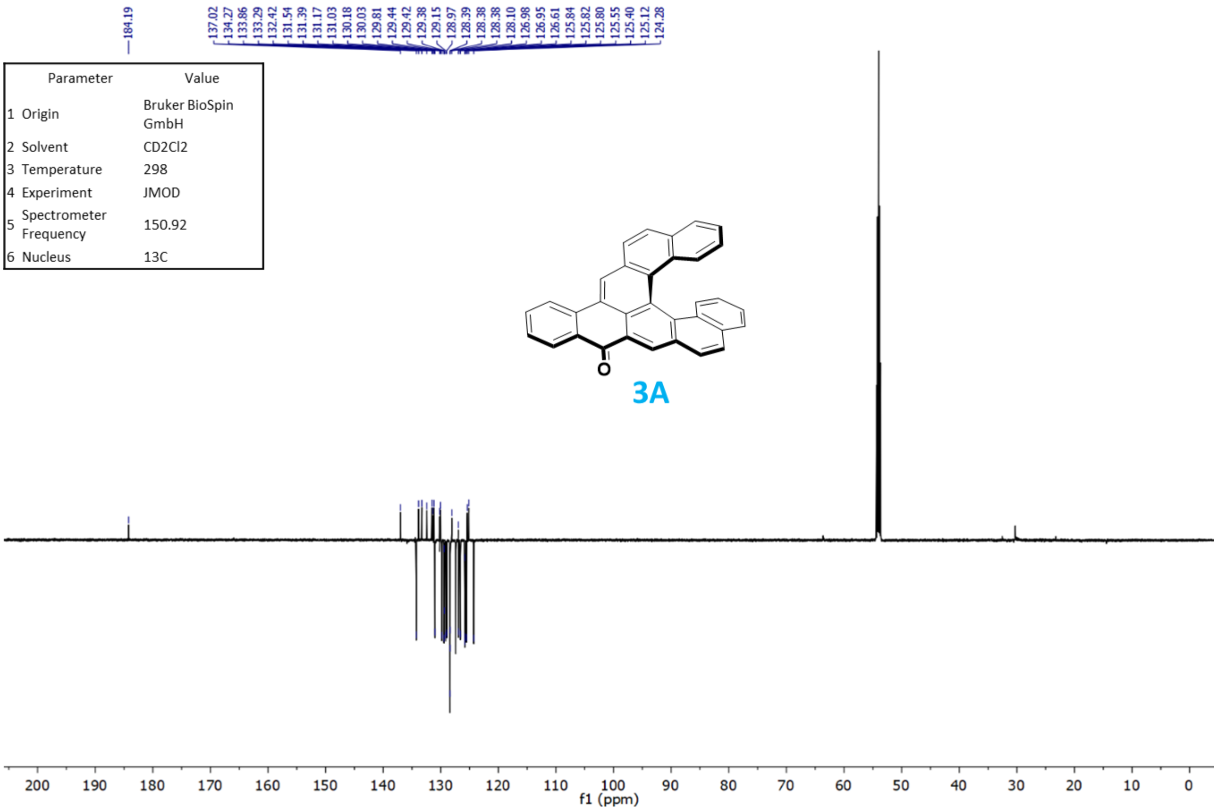
Parameter Value
1 Origin Bruker BioSpin GmbH
2 Solvent CD2Cl2
3 Temperature 298
4 Experiment JMOD
5 Spectrometer Frequency 150.92
6 Nucleus 13C
3A
f1 (ppm)

	Parameter	Value
1	Origin	Bruker BioSpin GmbH
2	Solvent	CDCl3
3	Temperature	300
4	Experiment	1D
5	Spectrometer Frequency	400.13
6	Nucleus	1H

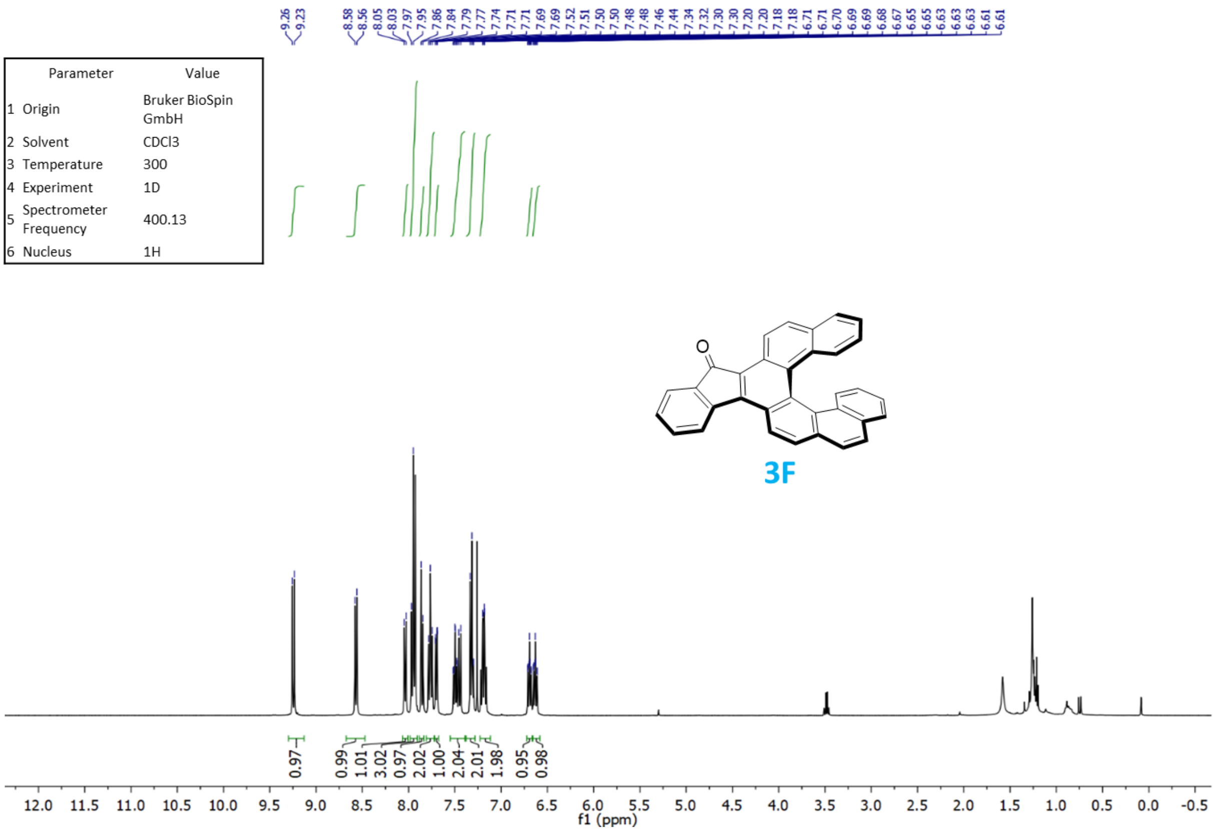

	Parameter	Value
1	Origin	Bruker BioSpin GmbH
2	Solvent	CDCl3
3	Temperature	300
4	Experiment	JMOD
5	Spectrometer Frequency	100.62
6	Nucleus	13C

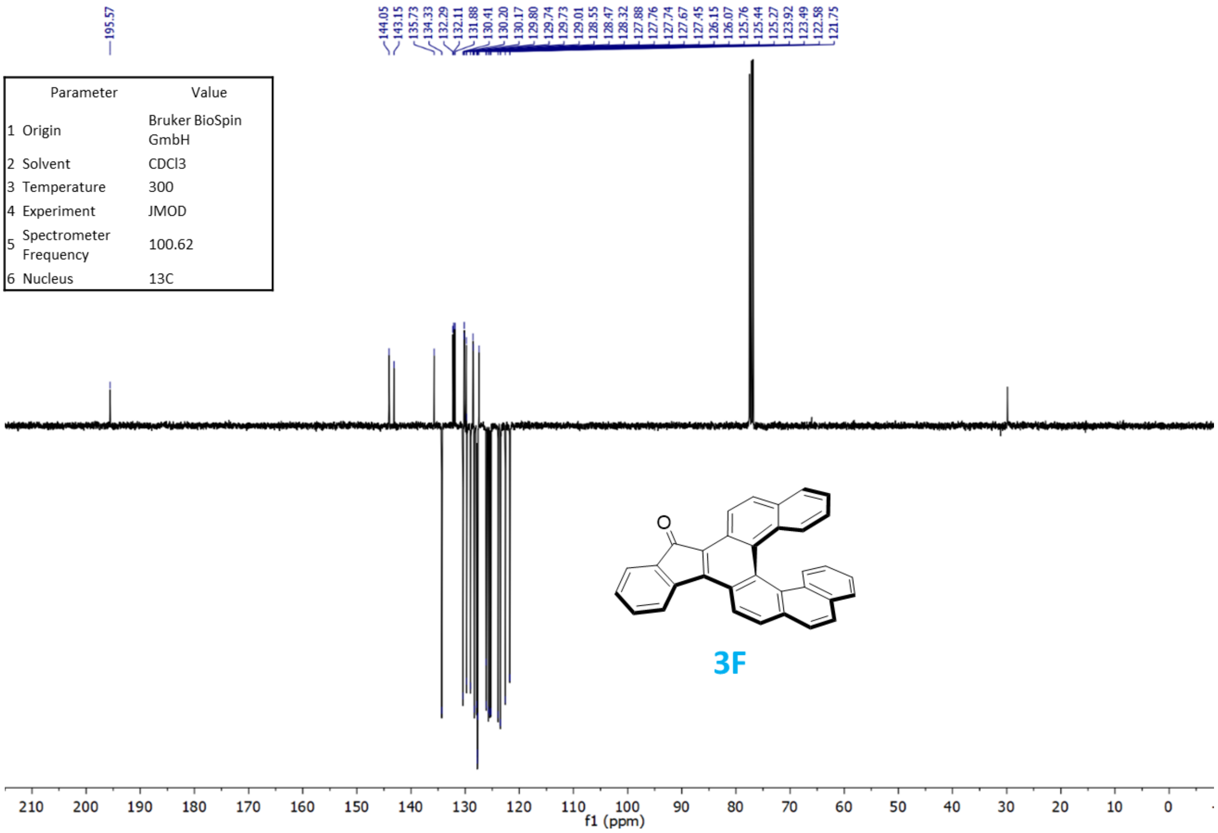

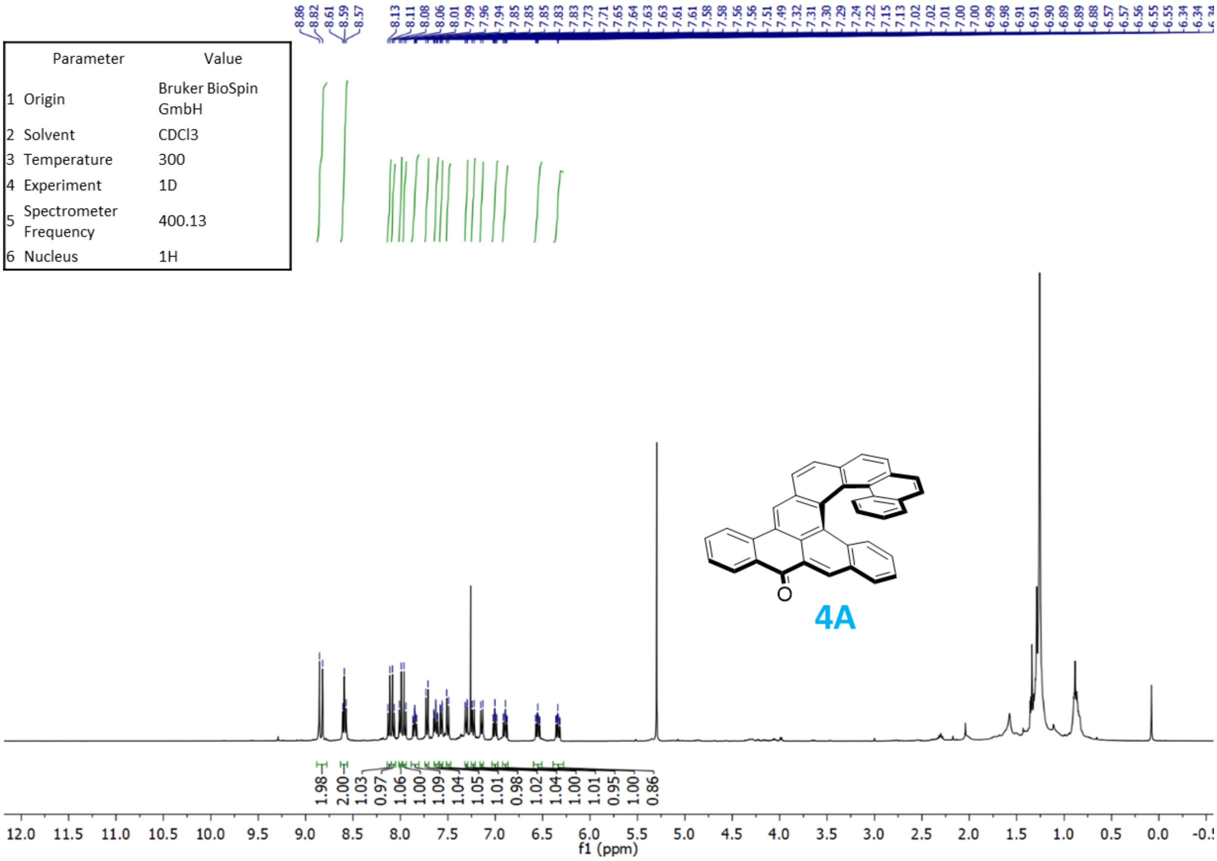

	Parameter	Value
1	Origin	Bruker BioSpin GmbH
2	Solvent	CDCl3
3	Temperature	300
4	Experiment	1D
5	Spectrometer Frequency	400.13
6	Nucleus	1H

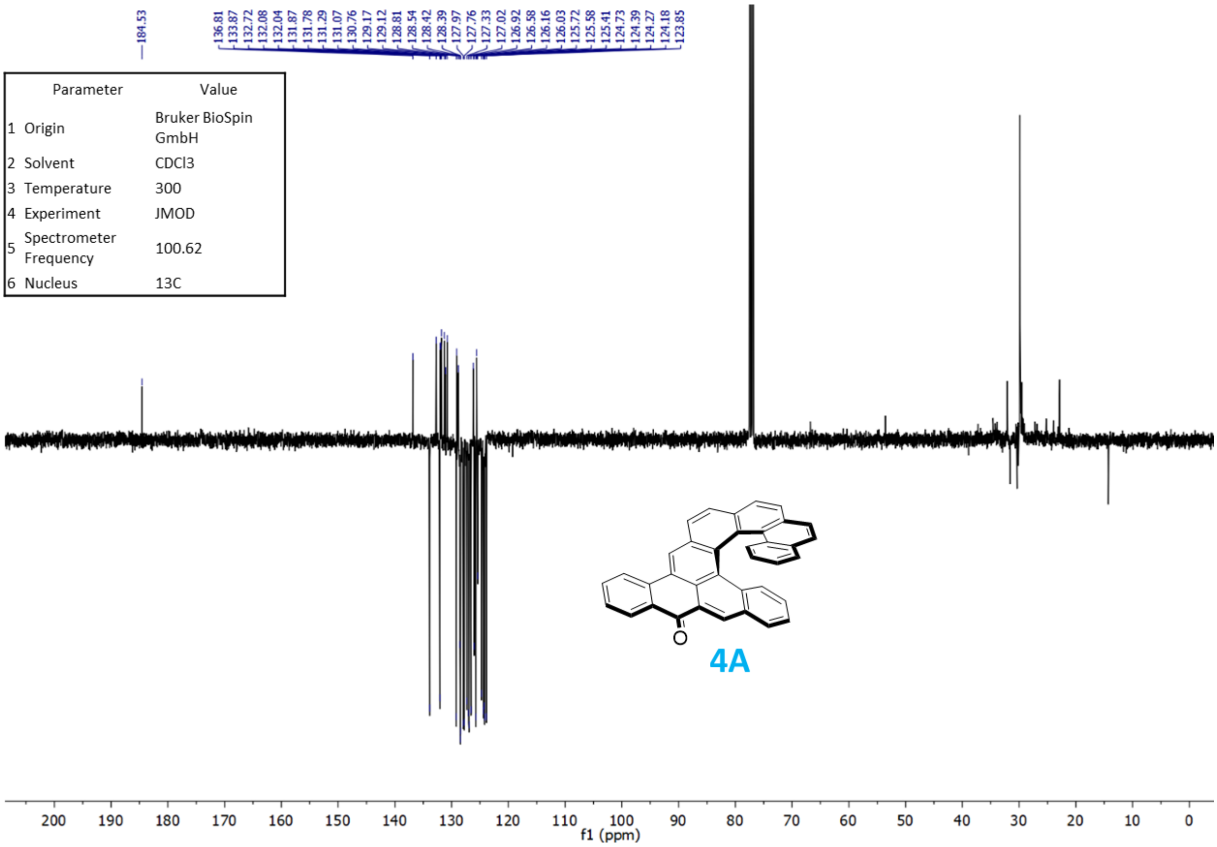

	Parameter	Value
1	Origin	Bruker BioSpin GmbH
2	Solvent	CDCl3
3	Temperature	300
4	Experiment	JMOD
5	Spectrometer Frequency	100.62
6	Nucleus	13C

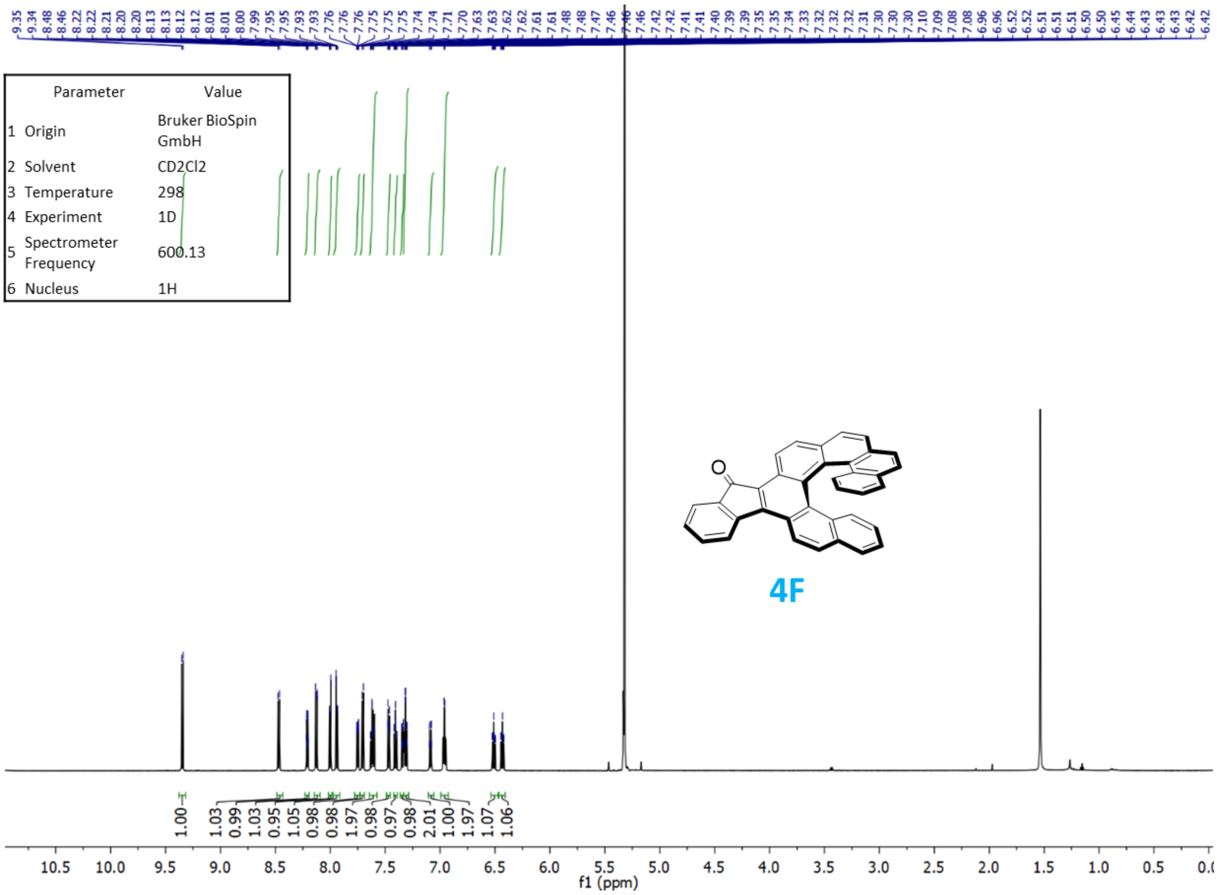

Parameter | Value
1 Origin | Bruker BioSpin GmbH
2 Solvent | CD2Cl2
3 Temperature | 298
4 Experiment | 1D
5 Spectrometer Frequency | 600.13
6 Nucleus | 1H
4F
f1 (ppm)

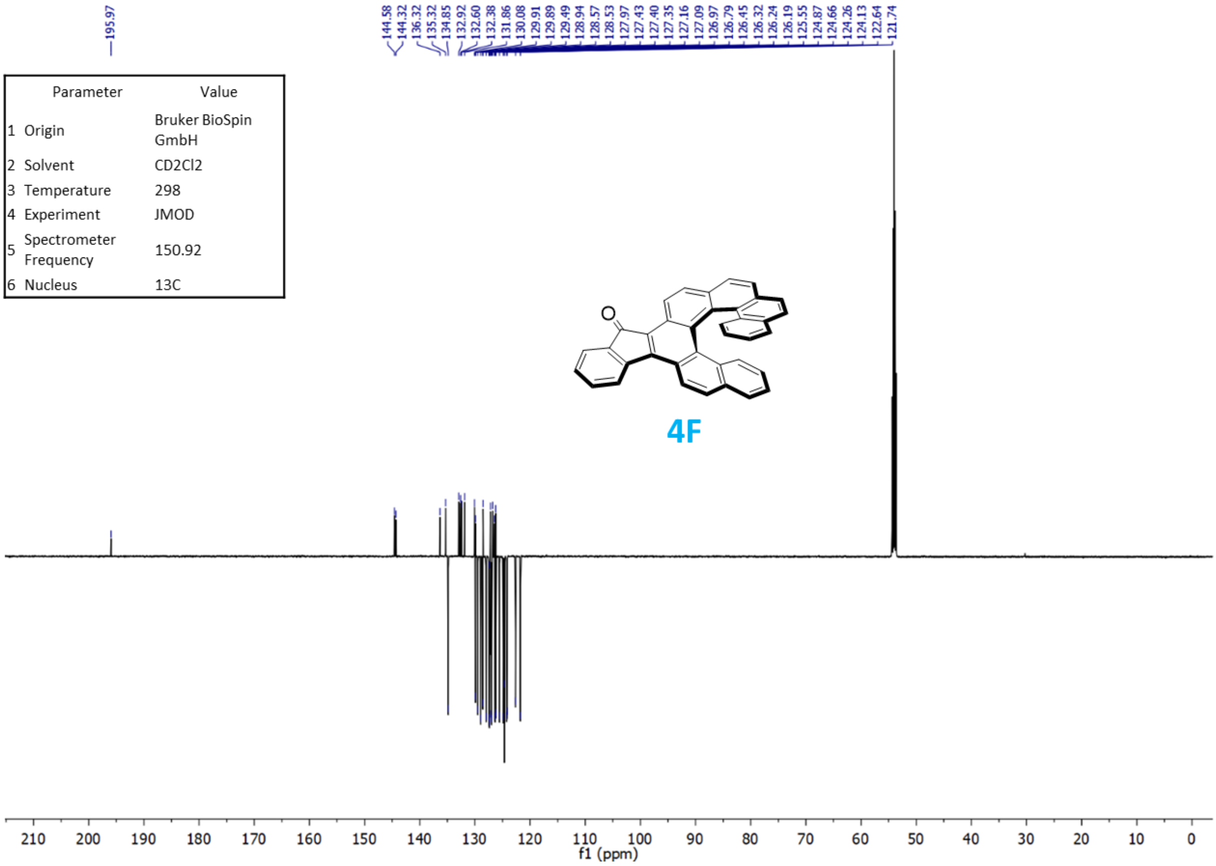

Parameter | Value
1 Origin | Bruker BioSpin GmbH
2 Solvent | CD2Cl2
3 Temperature | 298
4 Experiment | JMOD
5 Spectrometer Frequency | 150.92
6 Nucleus | 13C
4F
f1 (ppm)

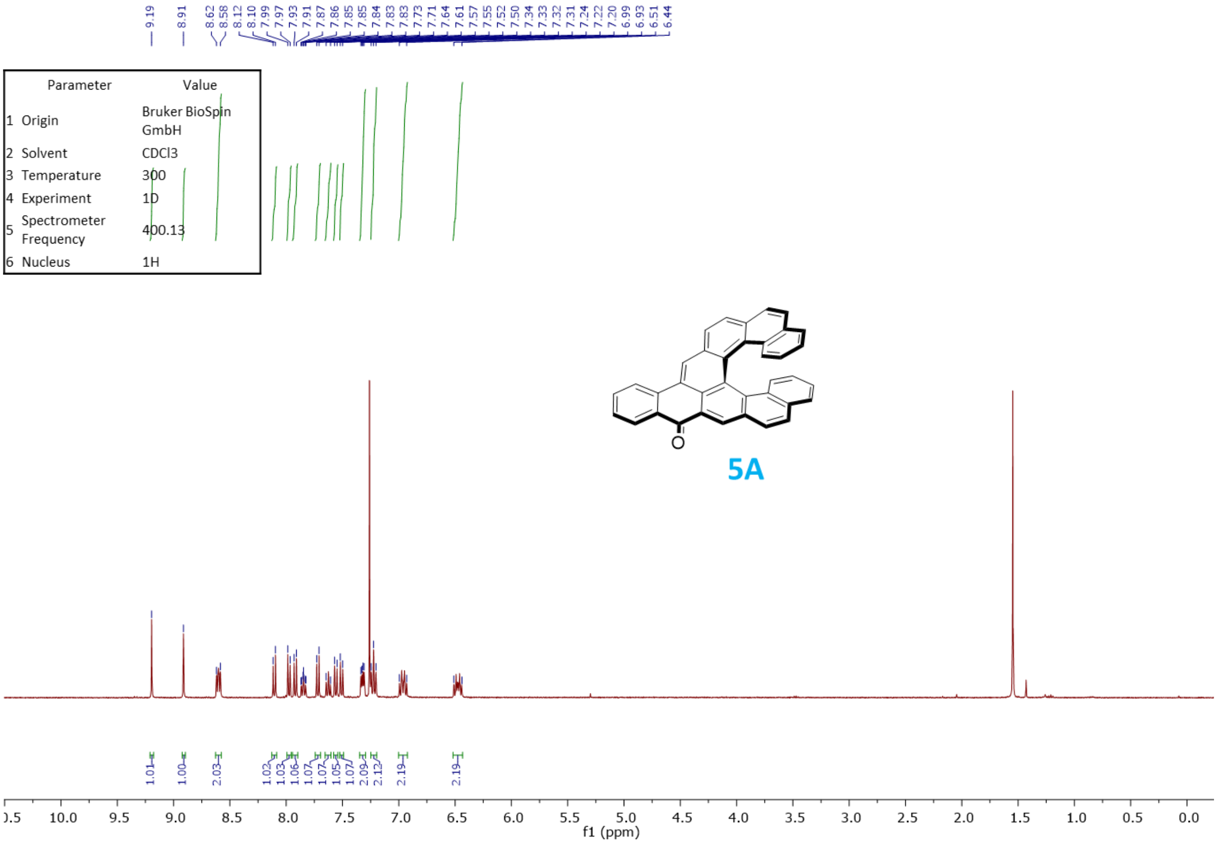

	Parameter	Value
1	Origin	Bruker BioSpin GmbH
2	Solvent	CDCl3
3	Temperature	300
4	Experiment	1D
5	Spectrometer Frequency	400.13
6	Nucleus	1H

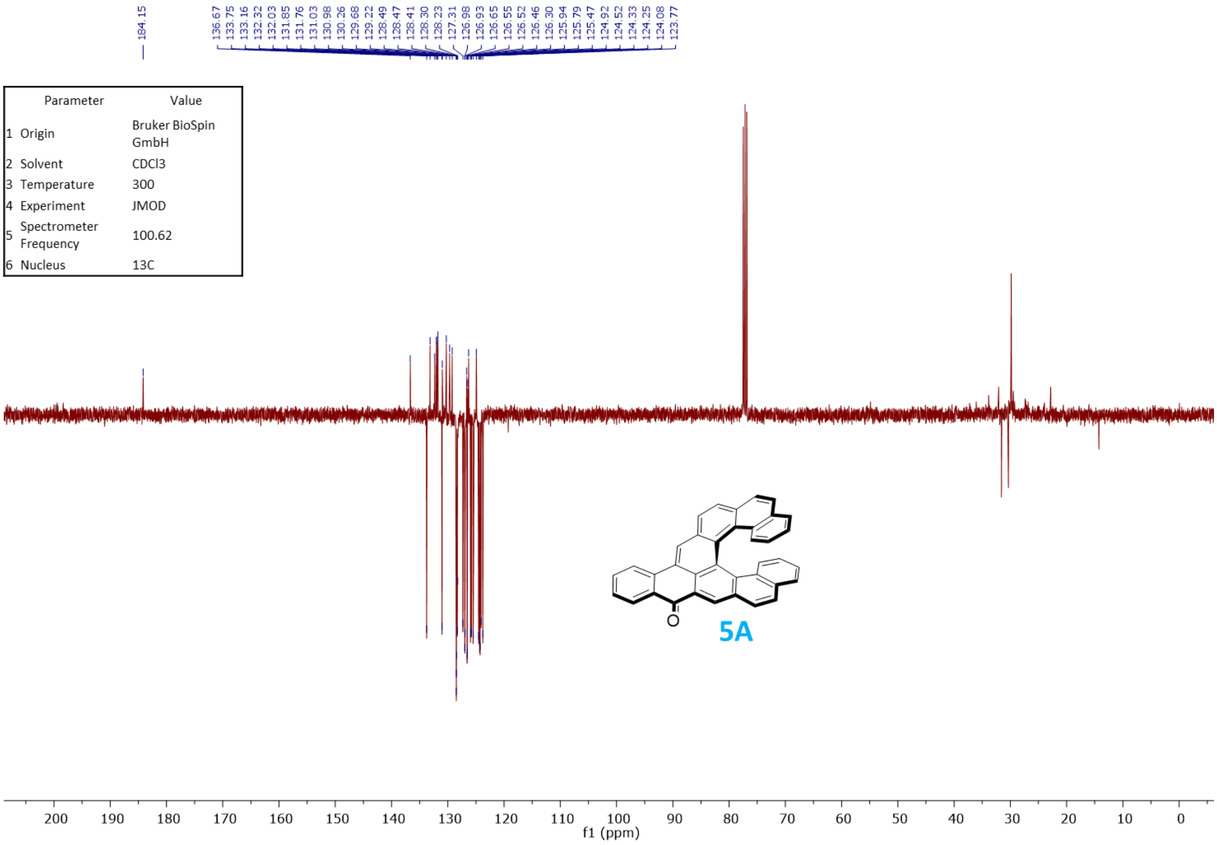

	Parameter	Value
1	Origin	Bruker BioSpin GmbH
2	Solvent	CDCl3
3	Temperature	300
4	Experiment	JMOD
5	Spectrometer Frequency	100.62
6	Nucleus	13C

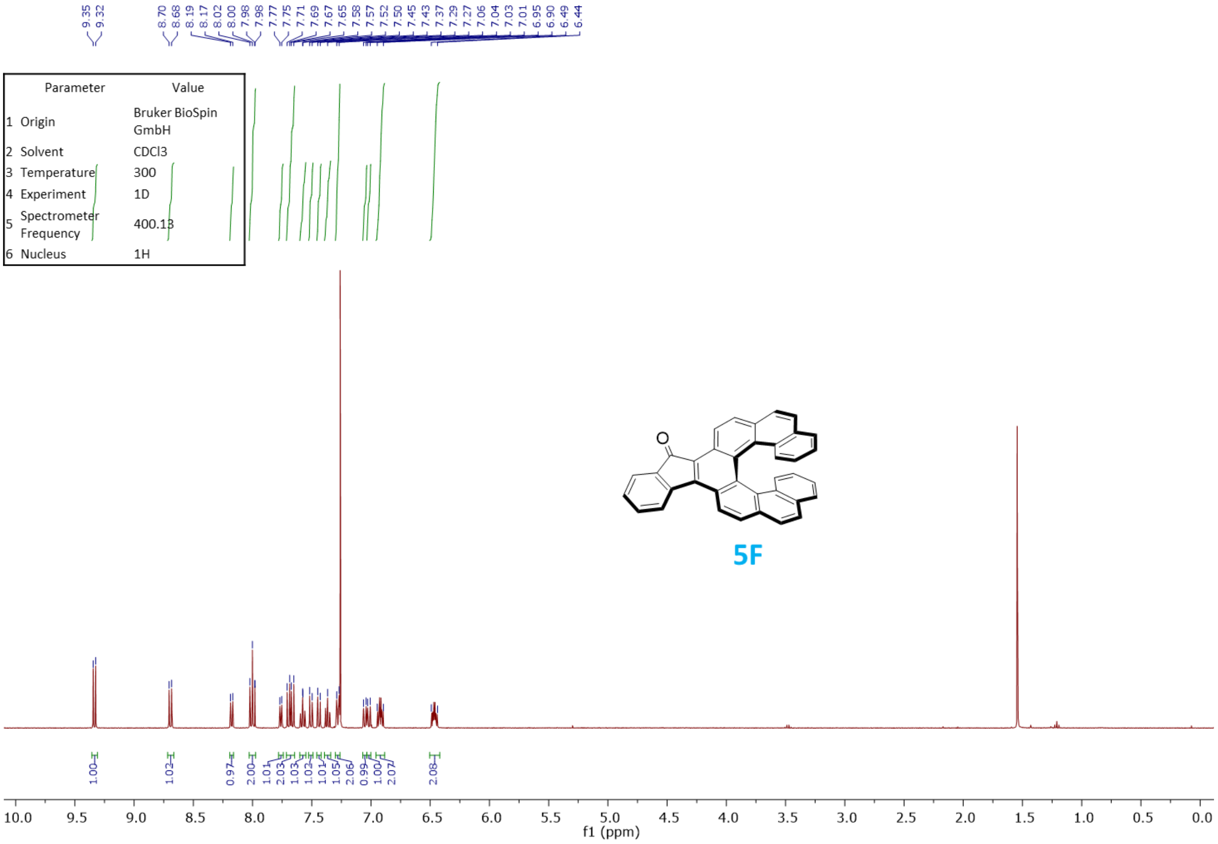
9.35
9.32
8.70
8.68
8.19
8.17
8.02
8.00
7.98
7.98
7.77
7.75
7.71
7.69
7.67
7.65
7.58
7.57
7.52
7.50
7.45
7.43
7.37
7.29
7.27
7.06
7.04
7.03
7.01
6.95
6.90
6.49
6.44
Parameter
Value
1 Origin
Bruker BioSpin GmbH
2 Solvent
CDCl3
3 Temperature
300
4 Experiment
1D
5 Spectrometer Frequency
400.13
6 Nucleus
1H
O
5F
1.00
1.02
0.97
2.00
1.01
2.03
1.03
1.02
1.01
1.05
2.06
0.99
1.00
2.07
2.08
f1 (ppm)

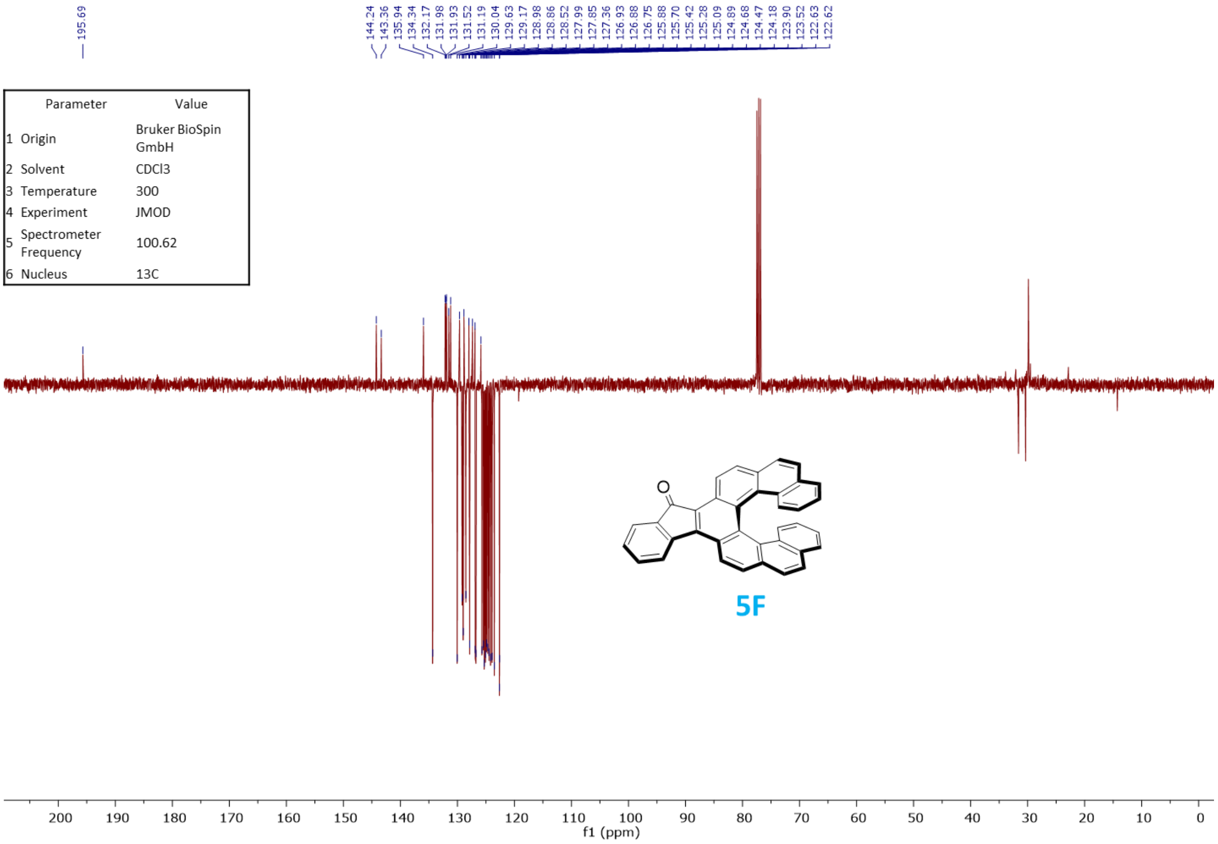
195.69
144.24
143.36
135.94
134.34
132.17
131.98
131.93
131.52
131.19
130.04
129.63
129.17
128.98
128.86
128.52
127.99
127.85
127.36
126.93
126.88
126.75
125.88
125.70
125.42
125.28
125.09
124.89
124.68
124.47
124.18
123.90
123.52
122.63
122.62
Parameter
Value
1 Origin
Bruker BioSpin GmbH
2 Solvent
CDCl3
3 Temperature
300
4 Experiment
JMOD
5 Spectrometer Frequency
100.62
6 Nucleus
13C
O
5F
f1 (ppm)

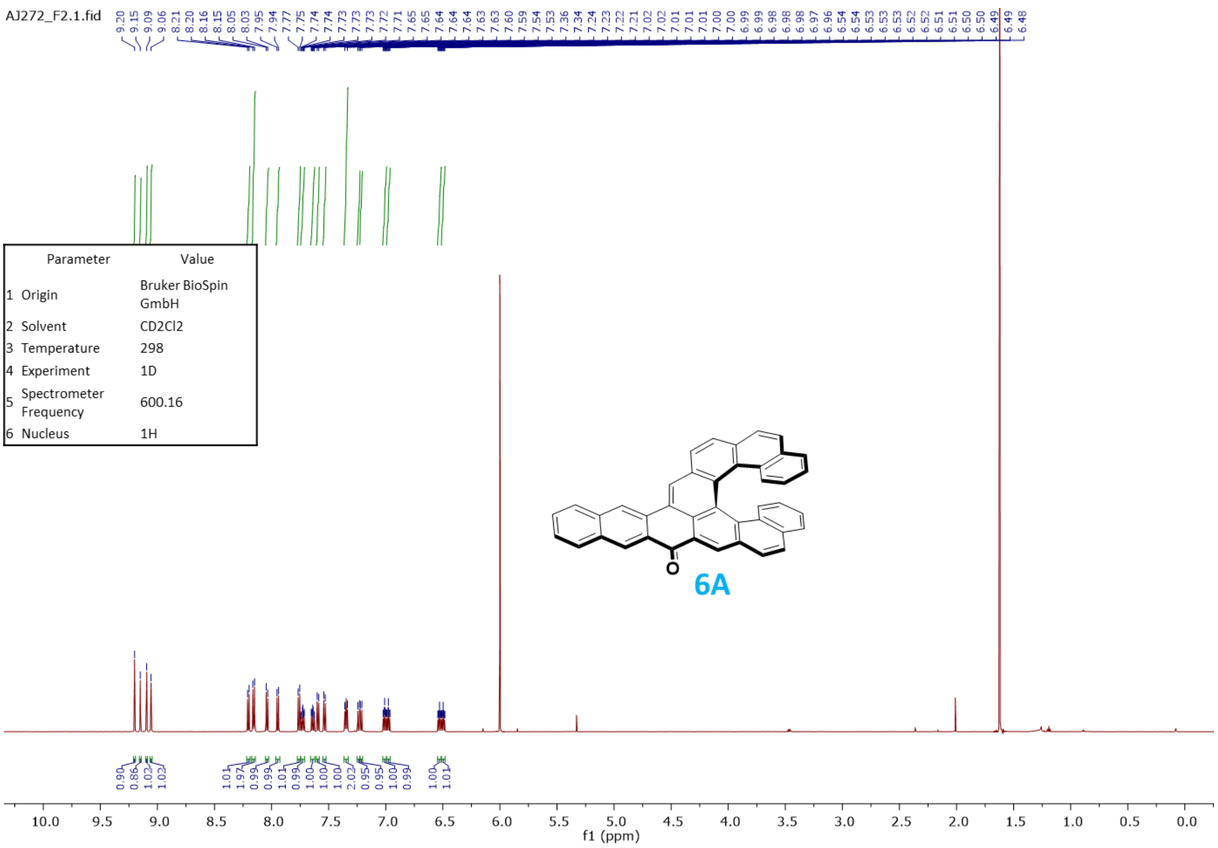

	Parameter	Value
1	Origin	Bruker BioSpin GmbH
2	Solvent	CD2Cl2
3	Temperature	298
4	Experiment	1D
5	Spectrometer Frequency	600.16
6	Nucleus	1H

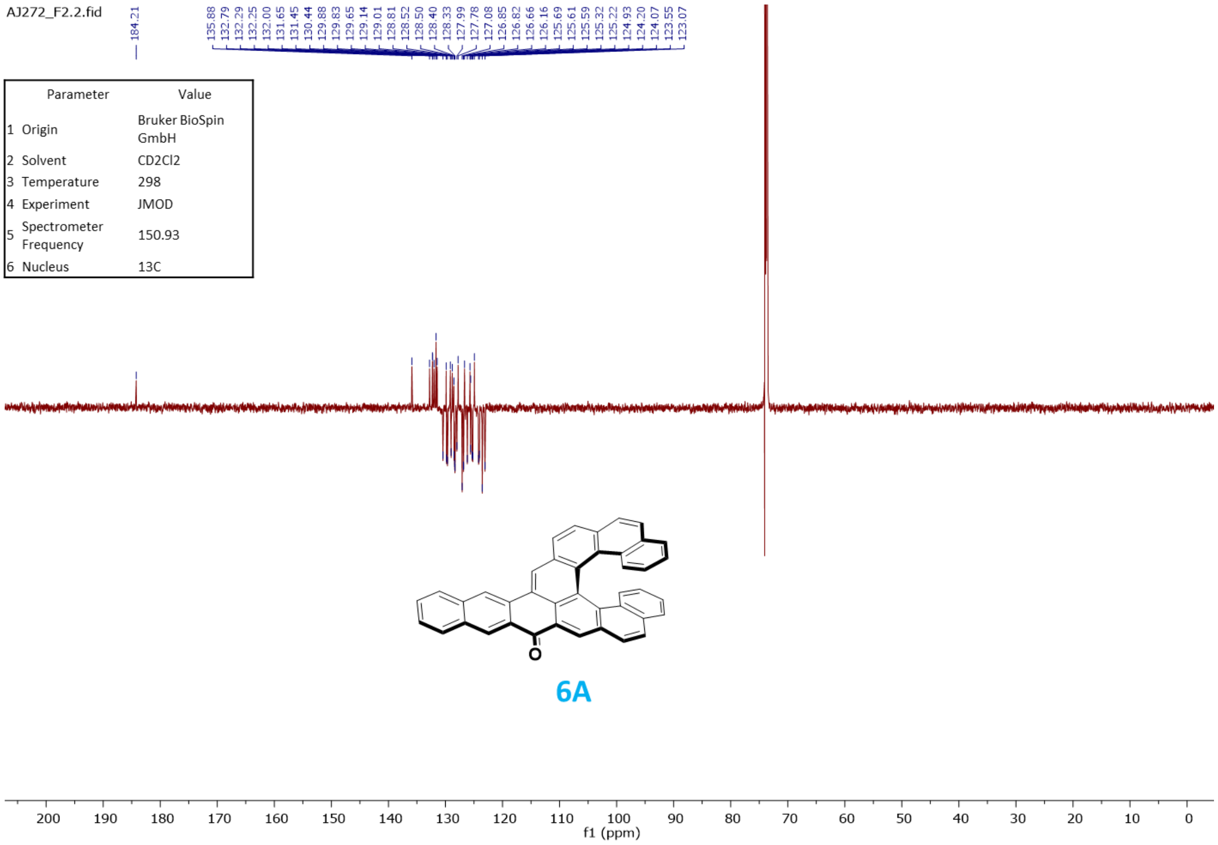

	Parameter	Value
1	Origin	Bruker BioSpin GmbH
2	Solvent	CD2Cl2
3	Temperature	298
4	Experiment	JMOD
5	Spectrometer Frequency	150.93
6	Nucleus	13C

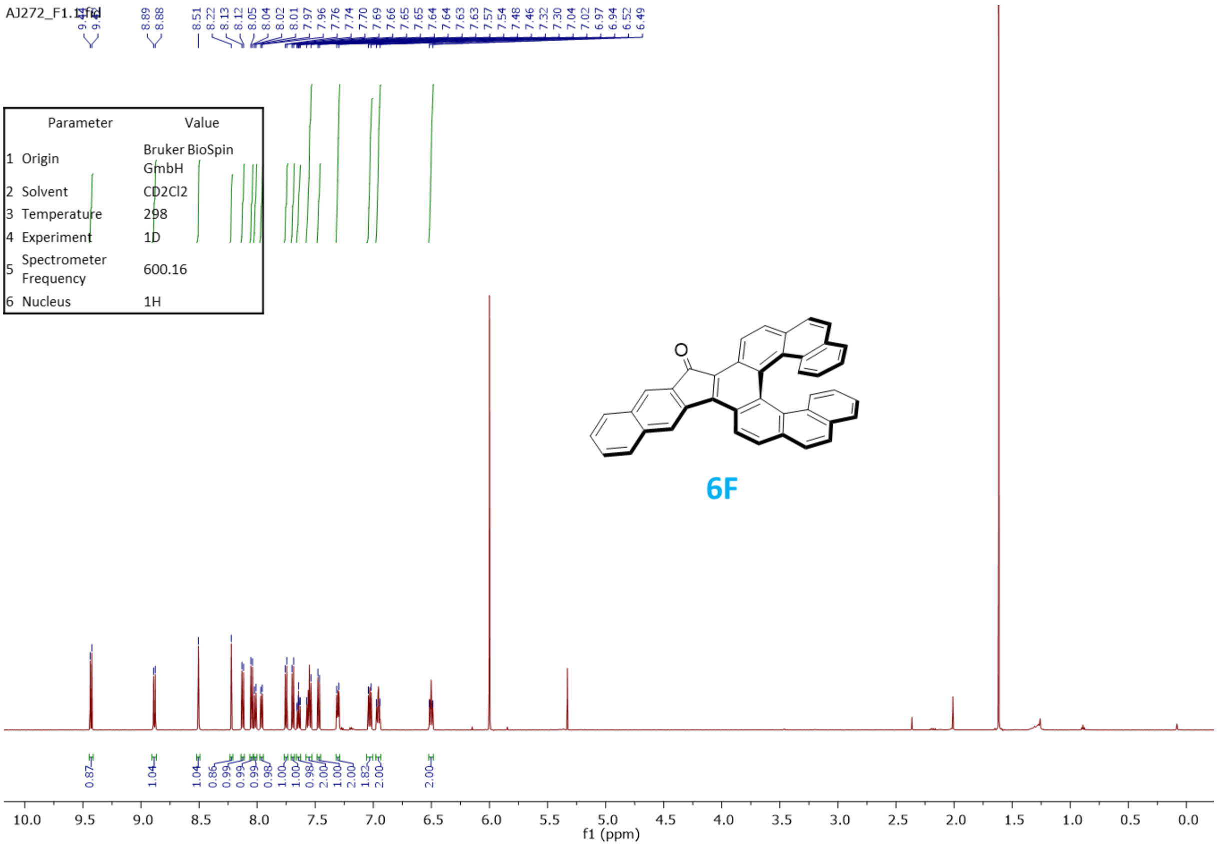

AJ272_F1.1.fid
8.89
8.88
8.51
8.22
8.13
8.12
8.05
8.04
8.02
8.01
7.97
7.96
7.76
7.74
7.70
7.69
7.66
7.65
7.65
7.64
7.64
7.63
7.63
7.57
7.54
7.48
7.46
7.32
7.30
7.04
7.02
6.97
6.94
6.52
6.49
Parameter Value
1 Origin Bruker BioSpin GmbH
2 Solvent CD2Cl2
3 Temperature 298
4 Experiment 1D
5 Spectrometer Frequency 600.16
6 Nucleus 1H
6F
0.87
1.04
1.04
0.86
0.99
0.99
0.99
0.96
1.00
1.00
0.96
2.00
1.00
2.00
1.82
2.00
2.00
10.0 9.5 9.0 8.5 8.0 7.5 7.0 6.5 6.0 5.5 5.0 4.5 4.0 3.5 3.0 2.5 2.0 1.5 1.0 0.5 0.0
f1 (ppm)

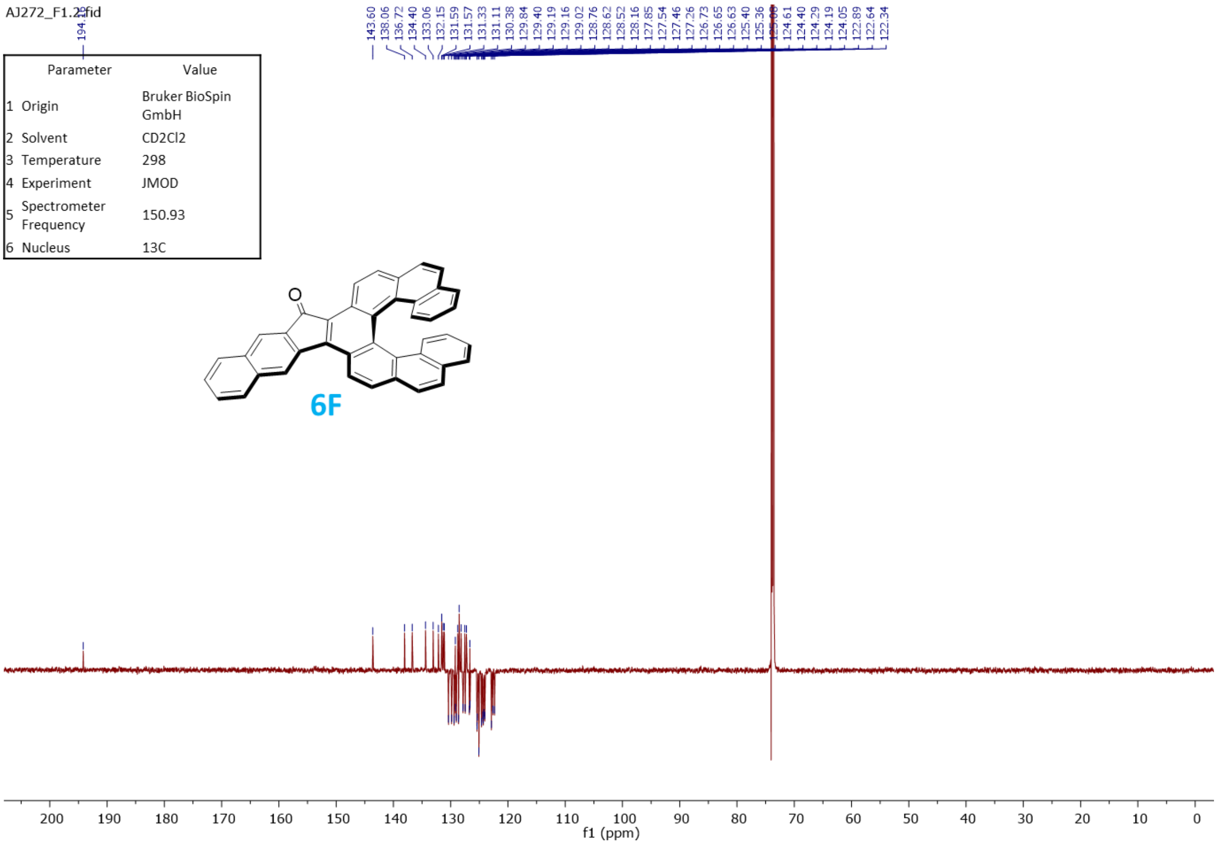

AJ272_F1.2.fid
143.60
138.06
136.72
134.40
133.06
132.15
131.59
131.57
131.33
131.11
130.38
129.84
129.40
129.19
129.16
129.02
128.76
128.62
128.52
128.16
127.85
127.54
127.46
127.26
126.73
126.65
126.63
125.40
125.36
124.61
124.40
124.29
124.19
124.05
122.89
122.64
122.34
Parameter Value
1 Origin Bruker BioSpin GmbH
2 Solvent CD2Cl2
3 Temperature 298
4 Experiment JMOD
5 Spectrometer Frequency 150.93
6 Nucleus 13C
6F
200 190 180 170 160 150 140 130 120 110 100 90 80 70 60 50 40 30 20 10 0
f1 (ppm)

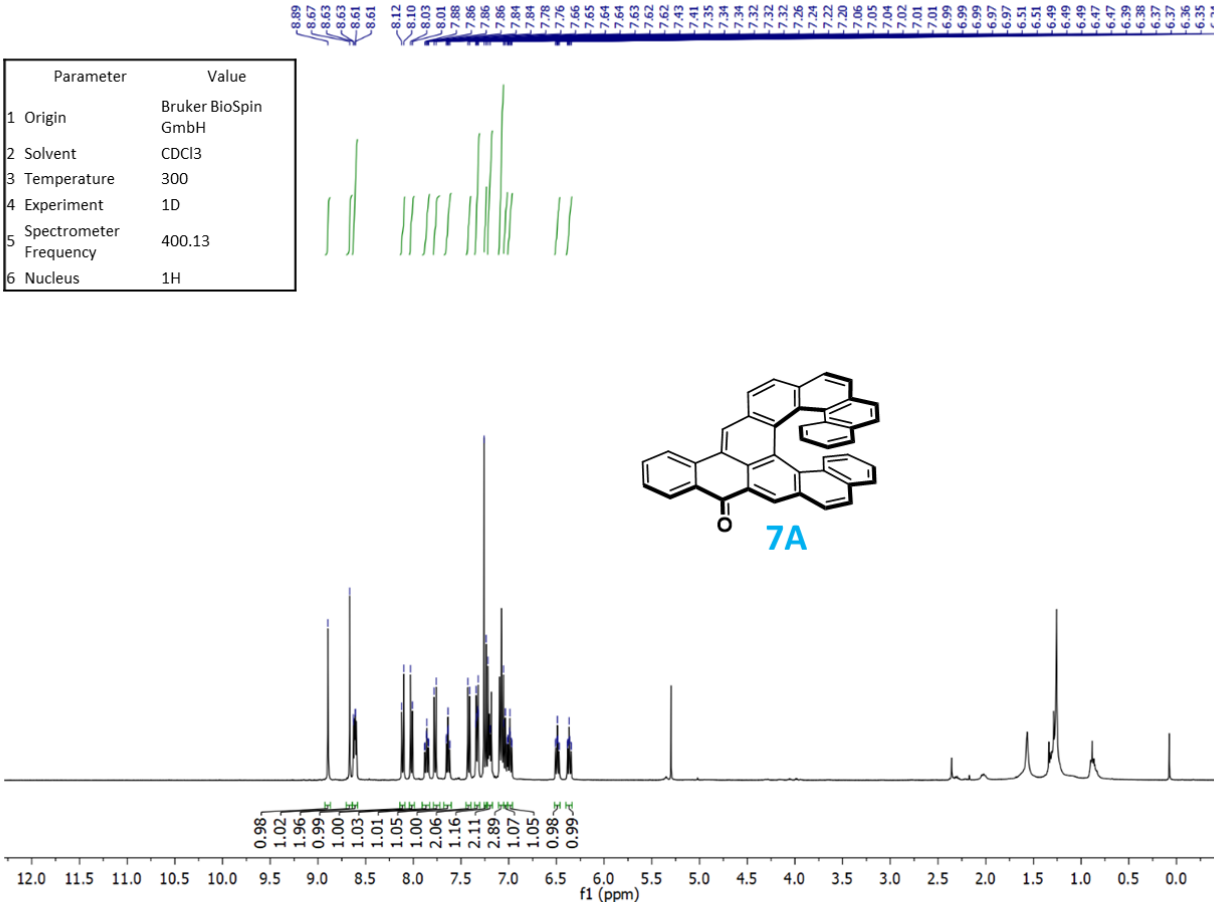

	Parameter	Value
1	Origin	Bruker BioSpin GmbH
2	Solvent	CDCl3
3	Temperature	300
4	Experiment	1D
5	Spectrometer Frequency	400.13
6	Nucleus	1H

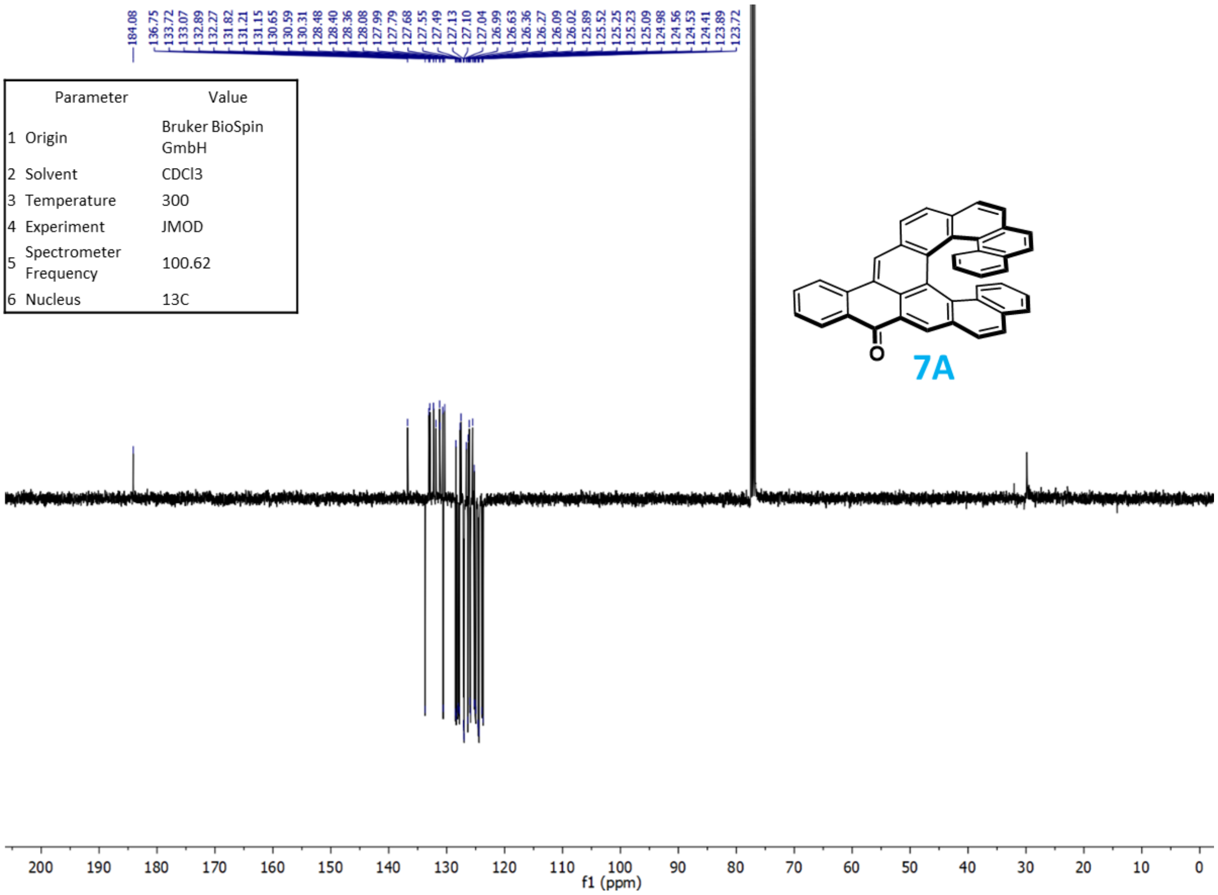

	Parameter	Value
1	Origin	Bruker BioSpin GmbH
2	Solvent	CDCl3
3	Temperature	300
4	Experiment	JMOD
5	Spectrometer Frequency	100.62
6	Nucleus	13C

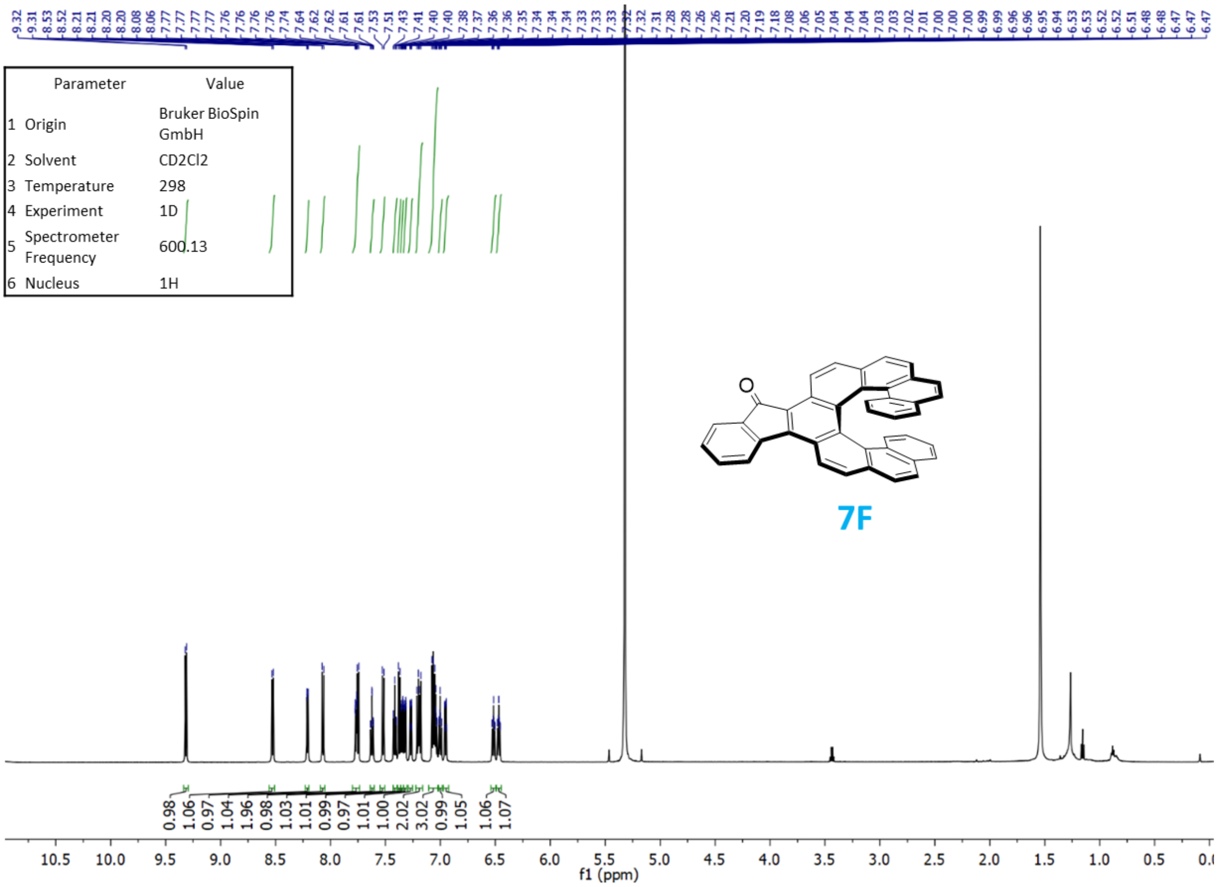
Parameter Value
1 Origin Bruker BioSpin GmbH
2 Solvent CD2Cl2
3 Temperature 298
4 Experiment 1D
5 Spectrometer Frequency 600.13
6 Nucleus 1H
7F
f1 (ppm)

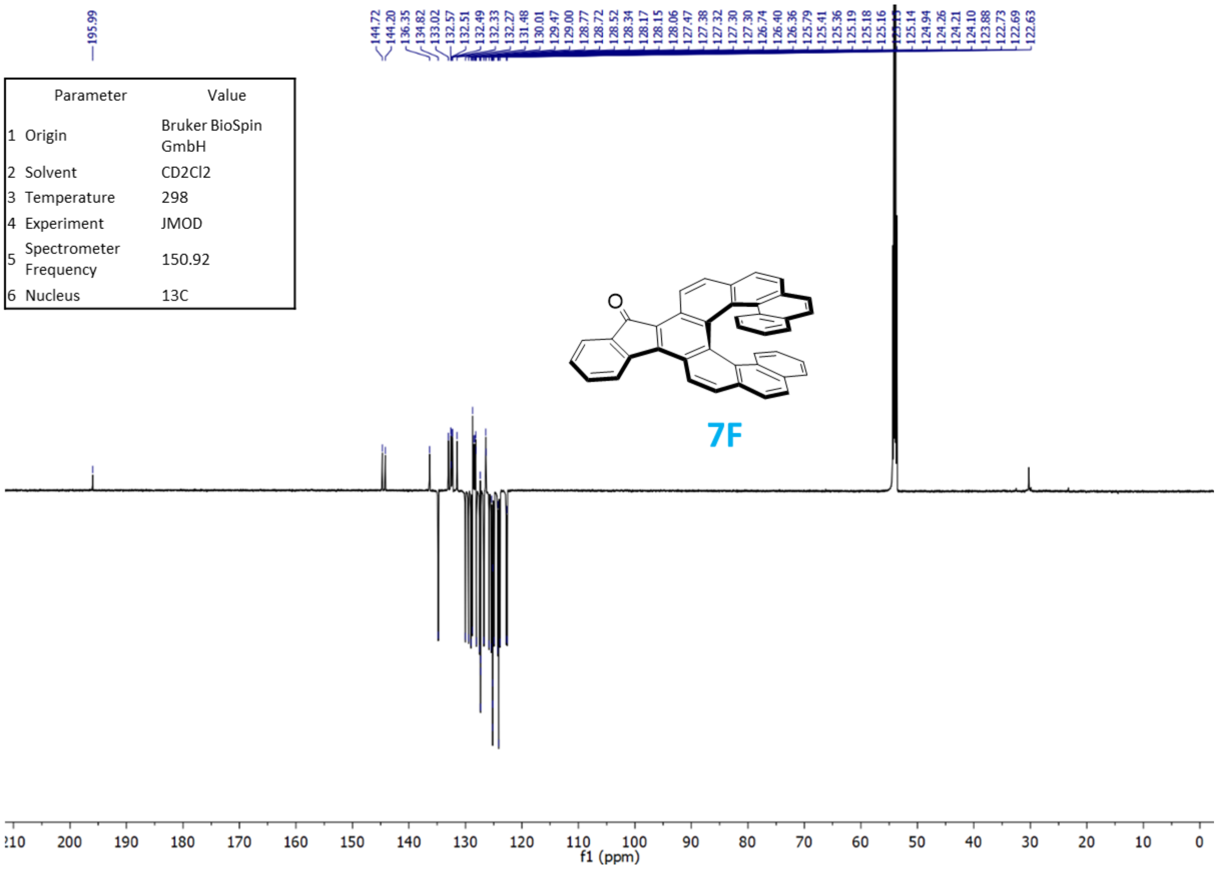
Parameter Value
1 Origin Bruker BioSpin GmbH
2 Solvent CD2Cl2
3 Temperature 298
4 Experiment JMOD
5 Spectrometer Frequency 150.92
6 Nucleus 13C
7F
f1 (ppm)

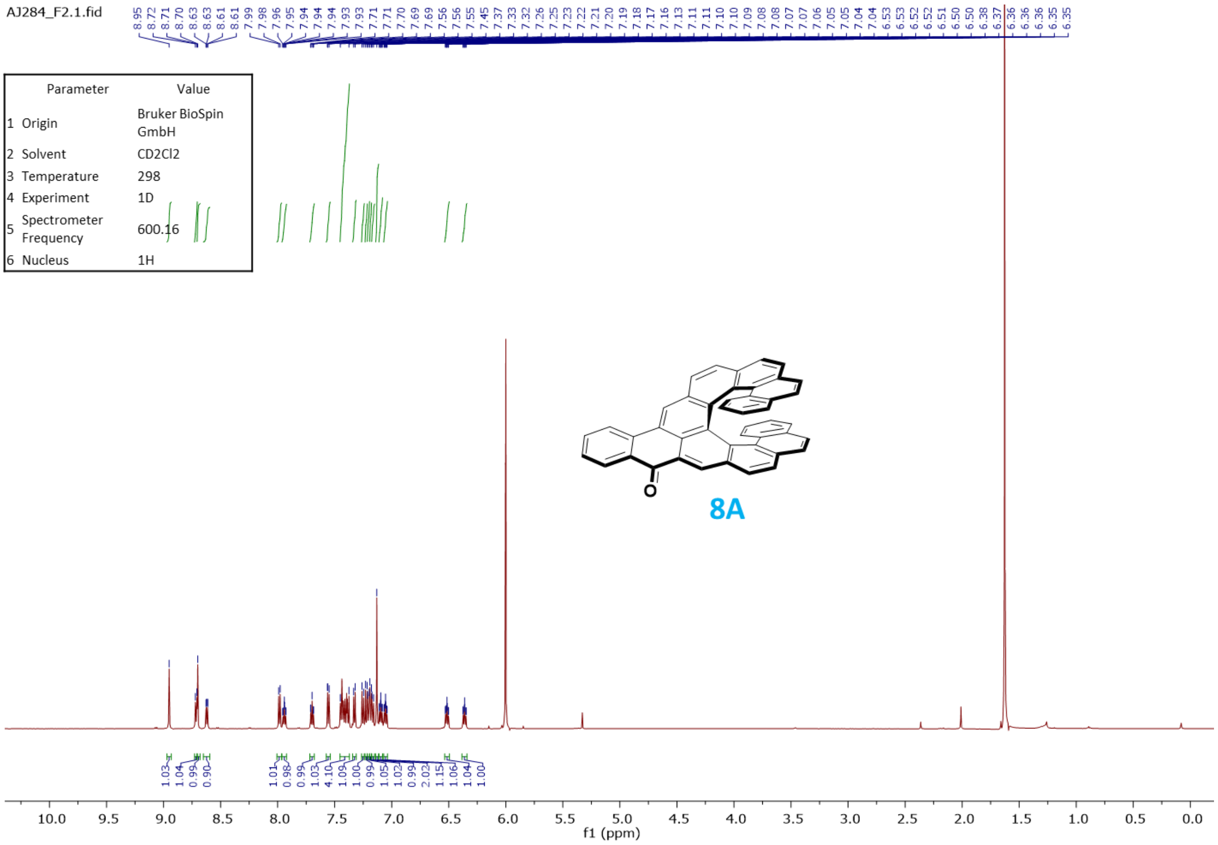
AJ284_F2.1.fid
8.95 8.72 8.71 8.70 8.63 8.63 8.61 8.61 7.99 7.98 7.96 7.95 7.94 7.94 7.94 7.93 7.93 7.71 7.71 7.70 7.69 7.69 7.56 7.56 7.55 7.45 7.37 7.33 7.32 7.26 7.25 7.23 7.22 7.21 7.20 7.19 7.18 7.17 7.16 7.13 7.13 7.11 7.11 7.10 7.10 7.09 7.08 7.08 7.07 7.07 7.06 7.05 7.05 7.04 7.04 6.53 6.53 6.52 6.52 6.51 6.50 6.38 6.37 6.36 6.36 6.35 6.35
Parameter Value
1 Origin Bruker BioSpin GmbH
2 Solvent CD2Cl2
3 Temperature 298
4 Experiment 1D
5 Spectrometer Frequency 600.16
6 Nucleus 1H
O
8A
1.03 1.04 0.99 0.90 1.01 0.98 0.99 1.03 4.10 1.09 1.00 0.99 1.05 1.02 0.99 2.02 1.15 1.06 1.04 1.00
10.0 9.5 9.0 8.5 8.0 7.5 7.0 6.5 6.0 5.5 5.0 4.5 4.0 3.5 3.0 2.5 2.0 1.5 1.0 0.5 0.0
f1 (ppm)

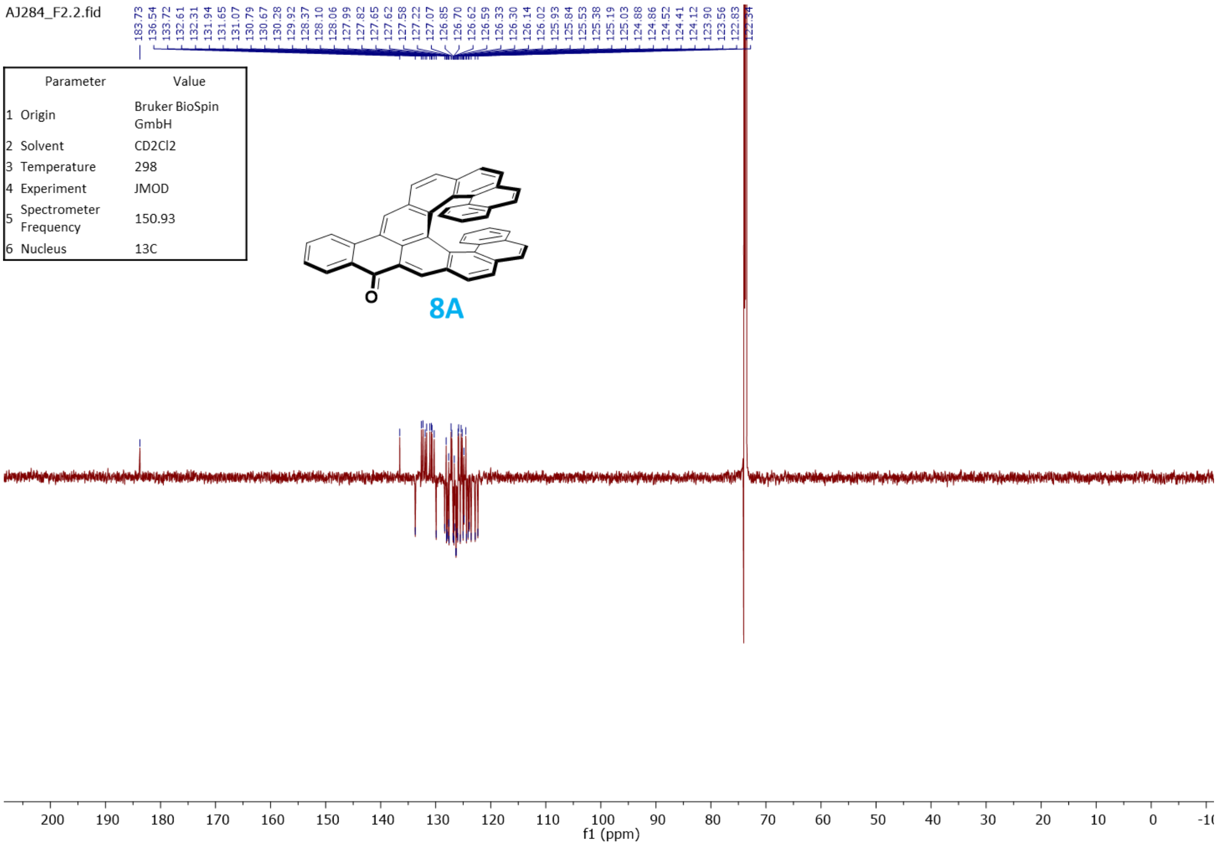
AJ284_F2.2.fid
183.73 136.54 133.72 132.61 132.31 131.94 131.65 131.07 130.79 130.67 130.28 129.92 128.37 128.10 128.06 127.99 127.82 127.65 127.62 127.58 127.22 127.07 126.85 126.70 126.62 126.59 126.33 126.30 126.14 126.02 125.93 125.84 125.53 125.38 125.19 125.03 124.88 124.86 124.52 124.41 124.12 123.90 123.56 122.83 122.34
Parameter Value
1 Origin Bruker BioSpin GmbH
2 Solvent CD2Cl2
3 Temperature 298
4 Experiment JMOD
5 Spectrometer Frequency 150.93
6 Nucleus 13C
O
8A
200 190 180 170 160 150 140 130 120 110 100 90 80 70 60 50 40 30 20 10 0 -10
f1 (ppm)

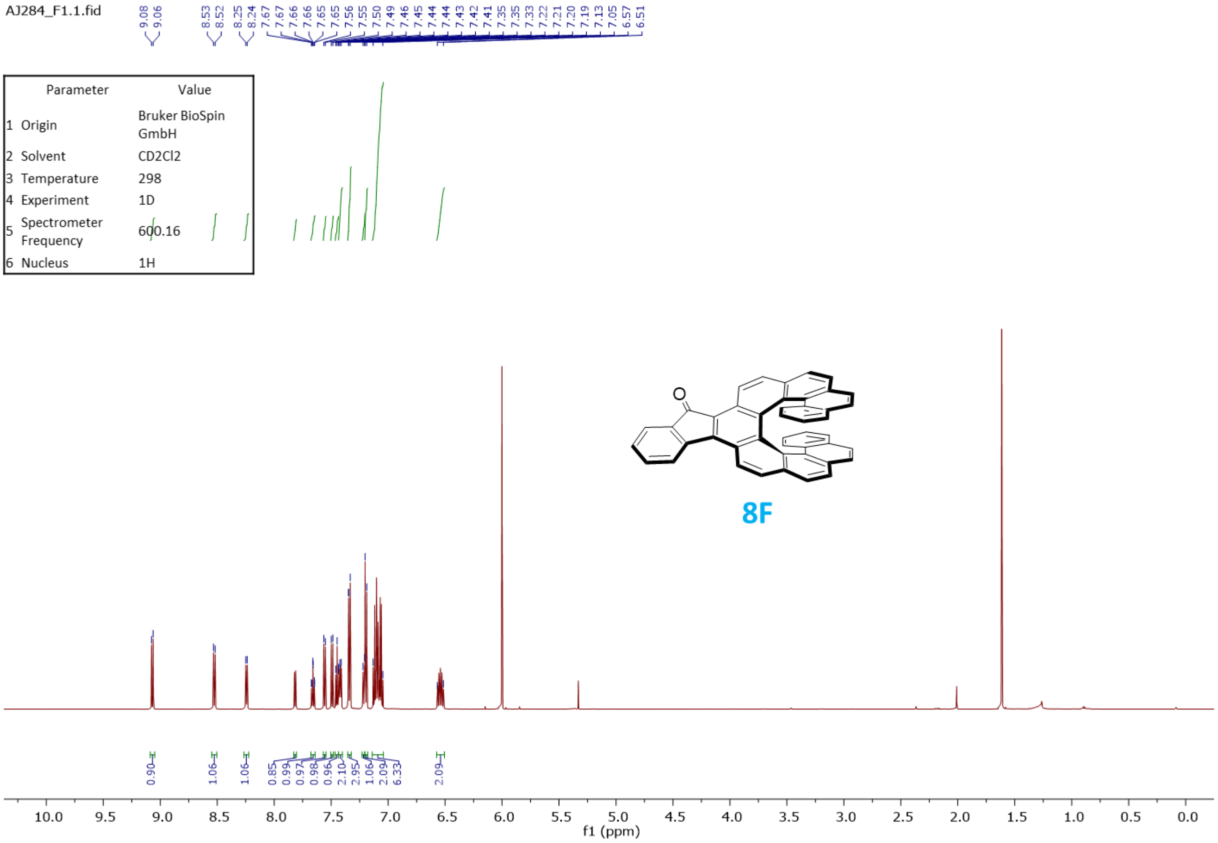
AJ284_F1.1.fid
Parameter | Value
1 Origin | Bruker BioSpin GmbH
2 Solvent | CD2Cl2
3 Temperature | 298
4 Experiment | 1D
5 Spectrometer Frequency | 600.16
6 Nucleus | 1H
8F
f1 (ppm)

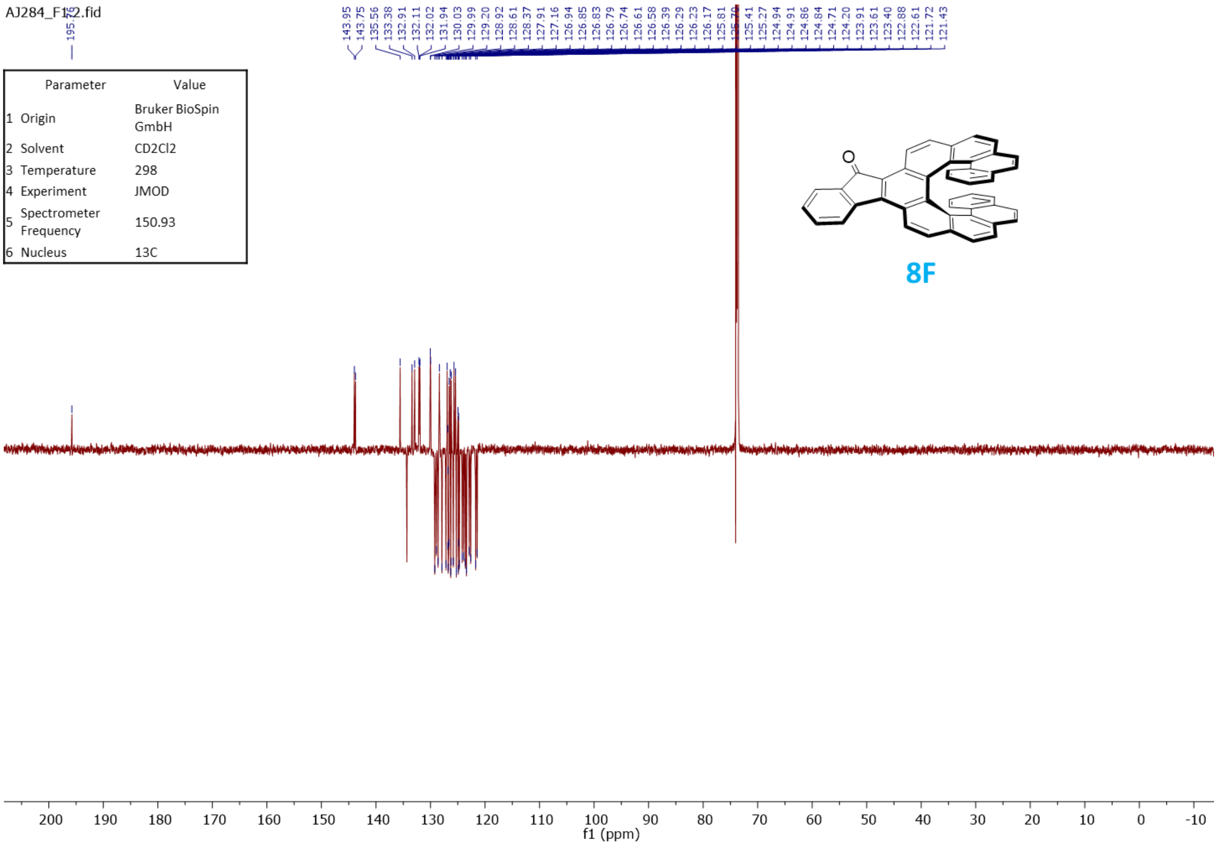
AJ284_F1.2.fid
Parameter | Value
1 Origin | Bruker BioSpin GmbH
2 Solvent | CD2Cl2
3 Temperature | 298
4 Experiment | JMOD
5 Spectrometer Frequency | 150.93
6 Nucleus | 13C
8F
f1 (ppm)

ls wittig1
single_pulse

	Parameter	Value
1	Origin	JEOL
2	Solvent	CD2Cl2
3	Temperature	297
4	Experiment	1D
5	Spectrometer Frequency	399.78
6	Nucleus	1H

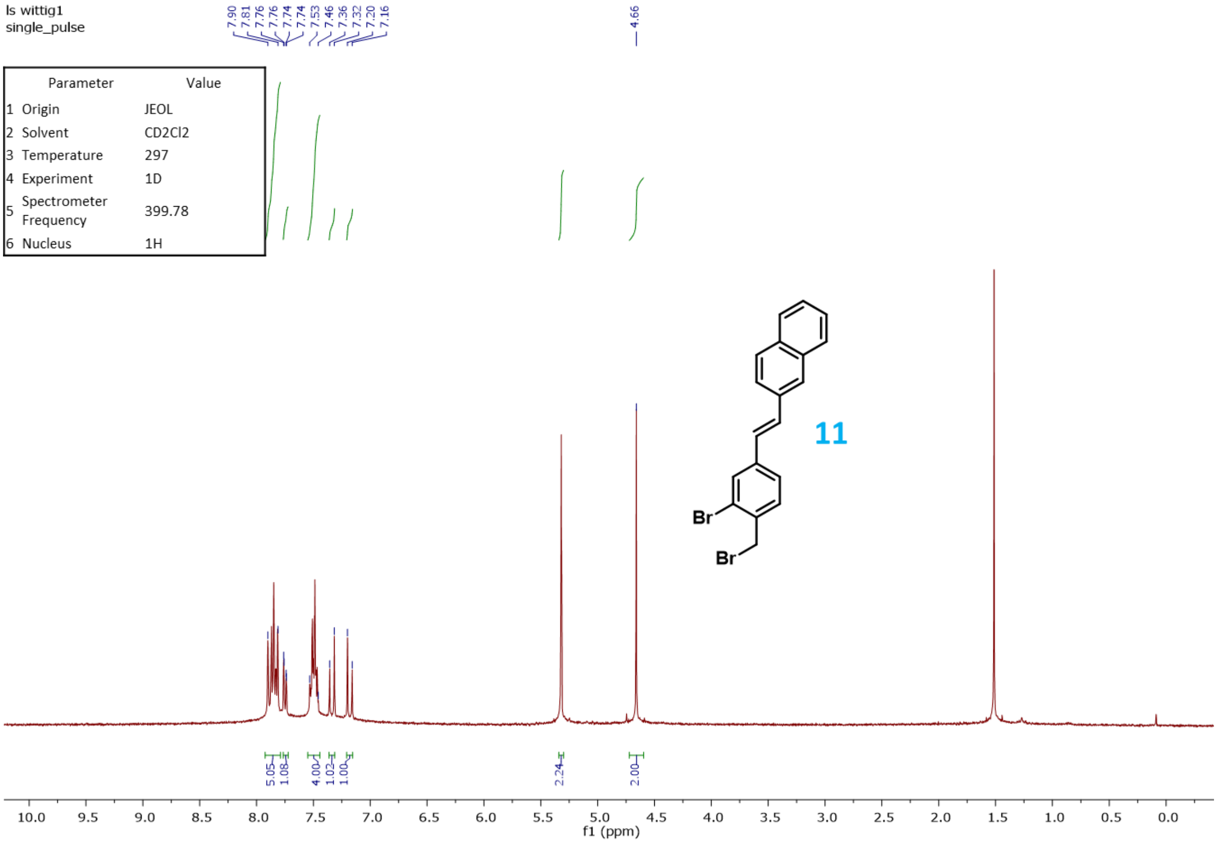

ls-wittig1
APT Experiment

	Parameter	Value
1	Origin	JEOL
2	Solvent	CD2Cl2
3	Temperature	297
4	Experiment	JMOD
5	Spectrometer Frequency	100.53
6	Nucleus	13C

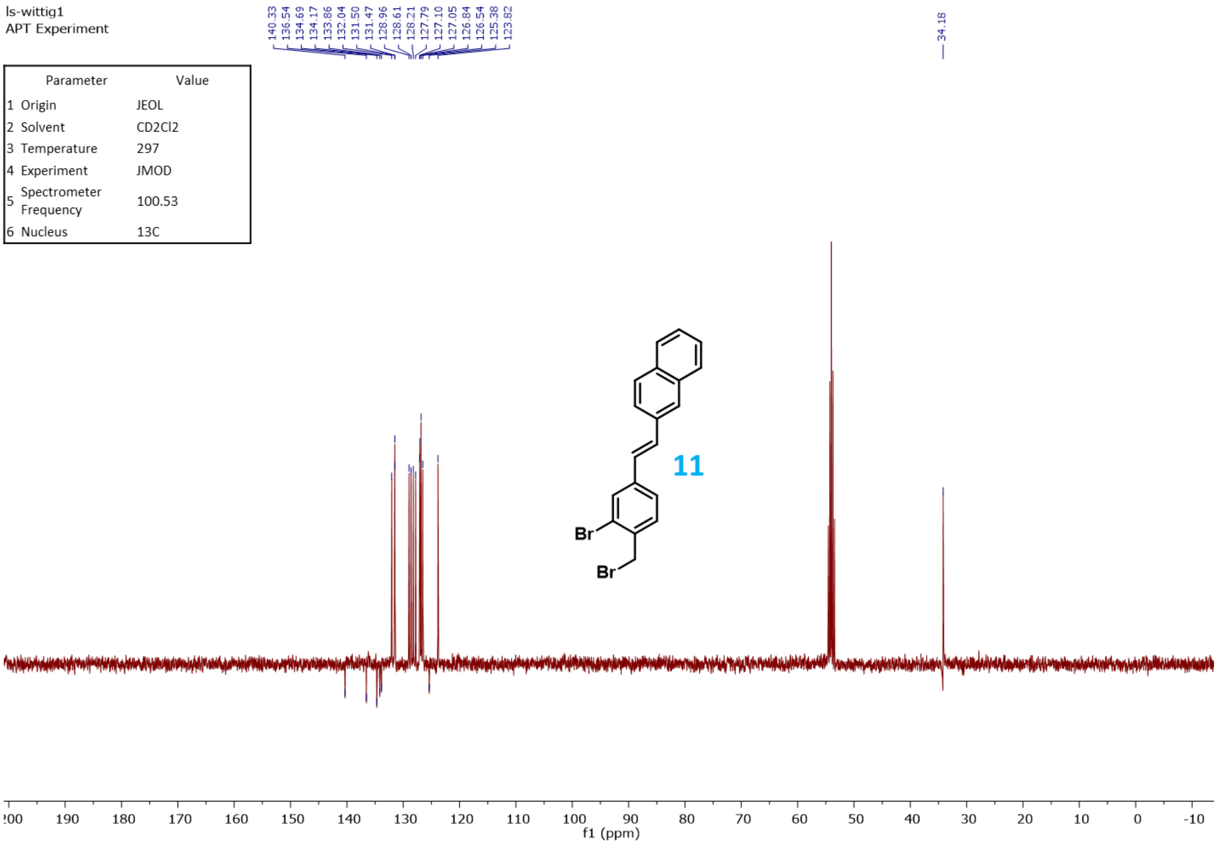

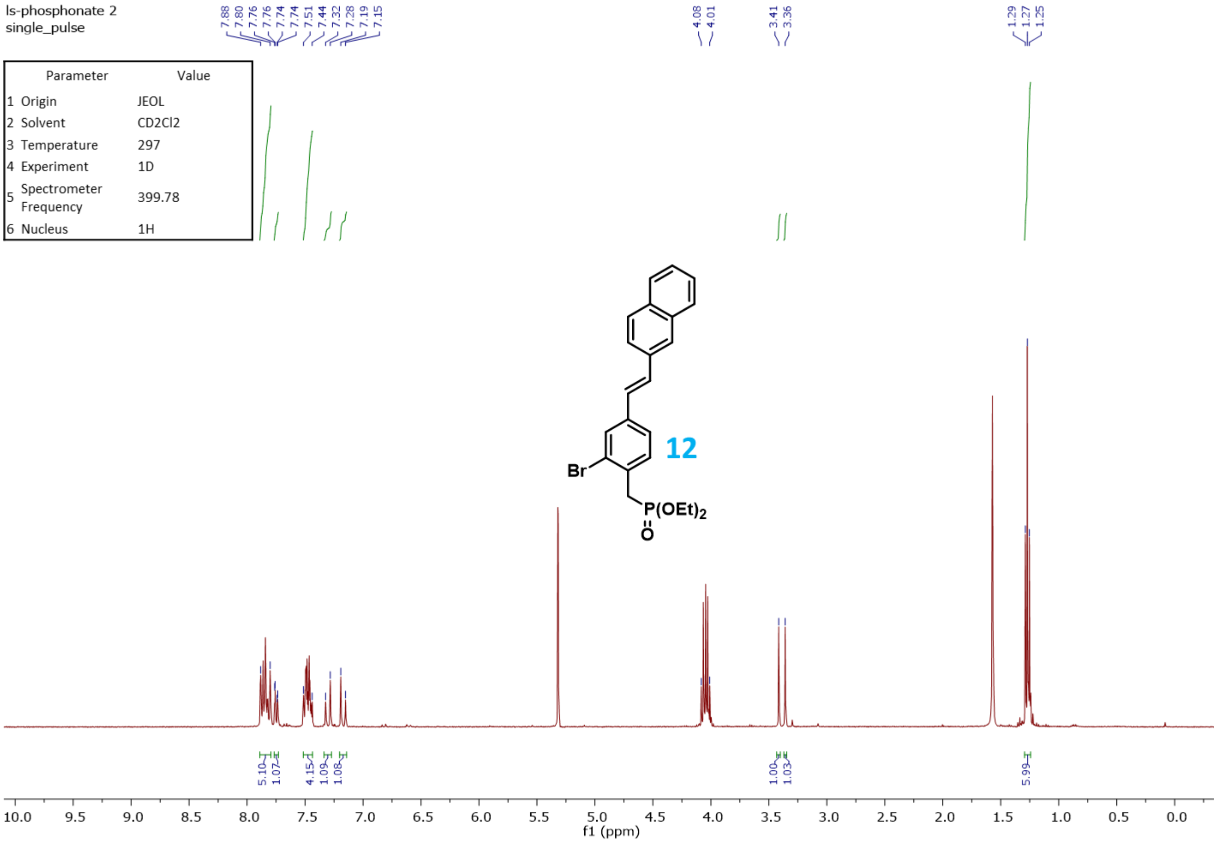

ls-phosphonate 2
single_pulse
7.88 7.80 7.76 7.76 7.74 7.74 7.51 7.44 7.32 7.28 7.19 7.15
4.08 4.01
3.41 3.36
1.29 1.27 1.25
Parameter Value
1 Origin JEOL
2 Solvent CD2Cl2
3 Temperature 297
4 Experiment 1D
5 Spectrometer Frequency 399.78
6 Nucleus 1H
12
Br
P(OEt)2
O
5.10 1.07 4.15 1.09 1.08 1.00 1.03 5.99
10.0 9.5 9.0 8.5 8.0 7.5 7.0 6.5 6.0 5.5 5.0 4.5 4.0 3.5 3.0 2.5 2.0 1.5 1.0 0.5 0.0
f1 (ppm)

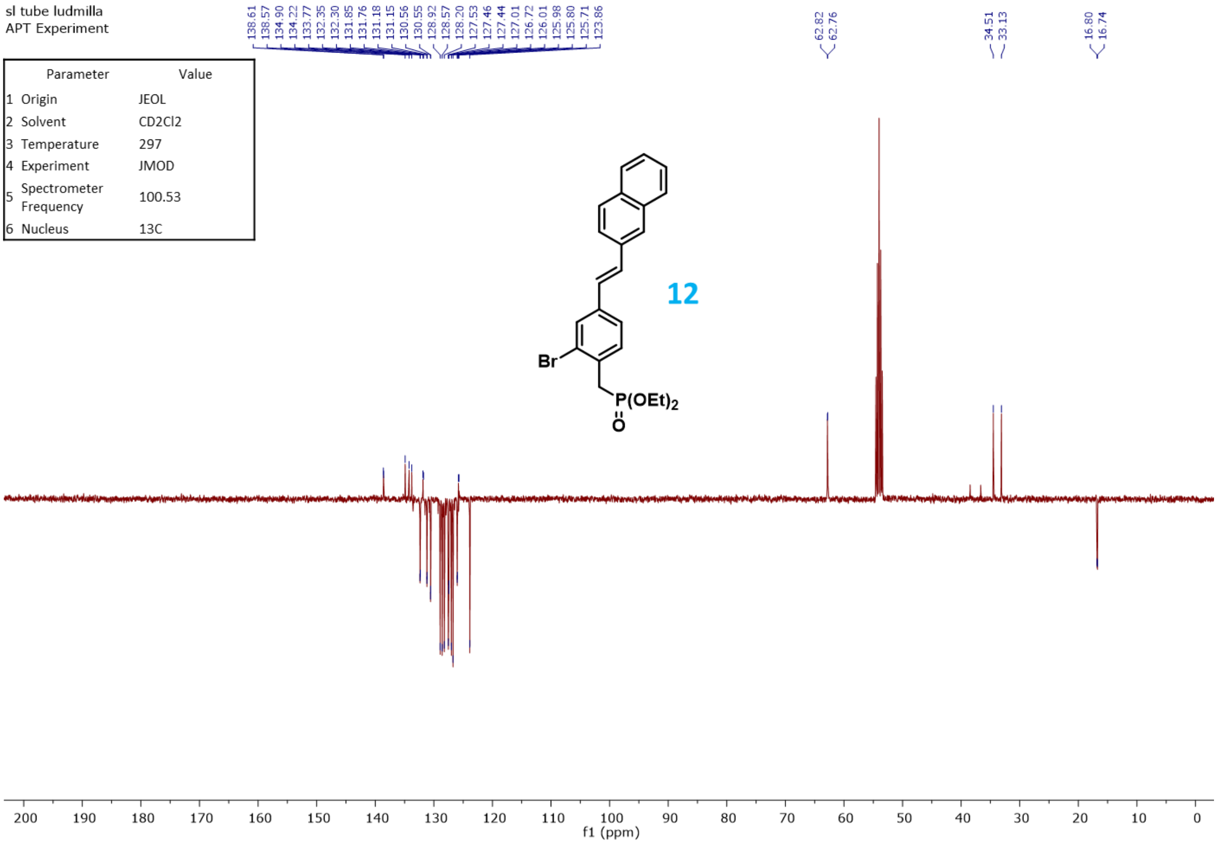

sl tube ludmilla
APT Experiment
138.61 138.57 134.90 134.22 133.77 132.35 132.30 131.85 131.76 131.18 131.15 130.56 130.55 128.92 128.57 128.20 127.53 127.46 127.44 127.01 126.72 126.01 125.98 125.80 125.71 123.86
62.82 62.76
34.51 33.13
16.80 16.74
Parameter Value
1 Origin JEOL
2 Solvent CD2Cl2
3 Temperature 297
4 Experiment JMOD
5 Spectrometer Frequency 100.53
6 Nucleus 13C
12
Br
P(OEt)2
O
200 190 180 170 160 150 140 130 120 110 100 90 80 70 60 50 40 30 20 10 0
f1 (ppm)

ls-bisstilbene
single_pulse

	Parameter	Value
1	Origin	JEOL
2	Solvent	CD2Cl2
3	Temperature	297
4	Experiment	1D
5	Spectrometer Frequency	399.78
6	Nucleus	1H

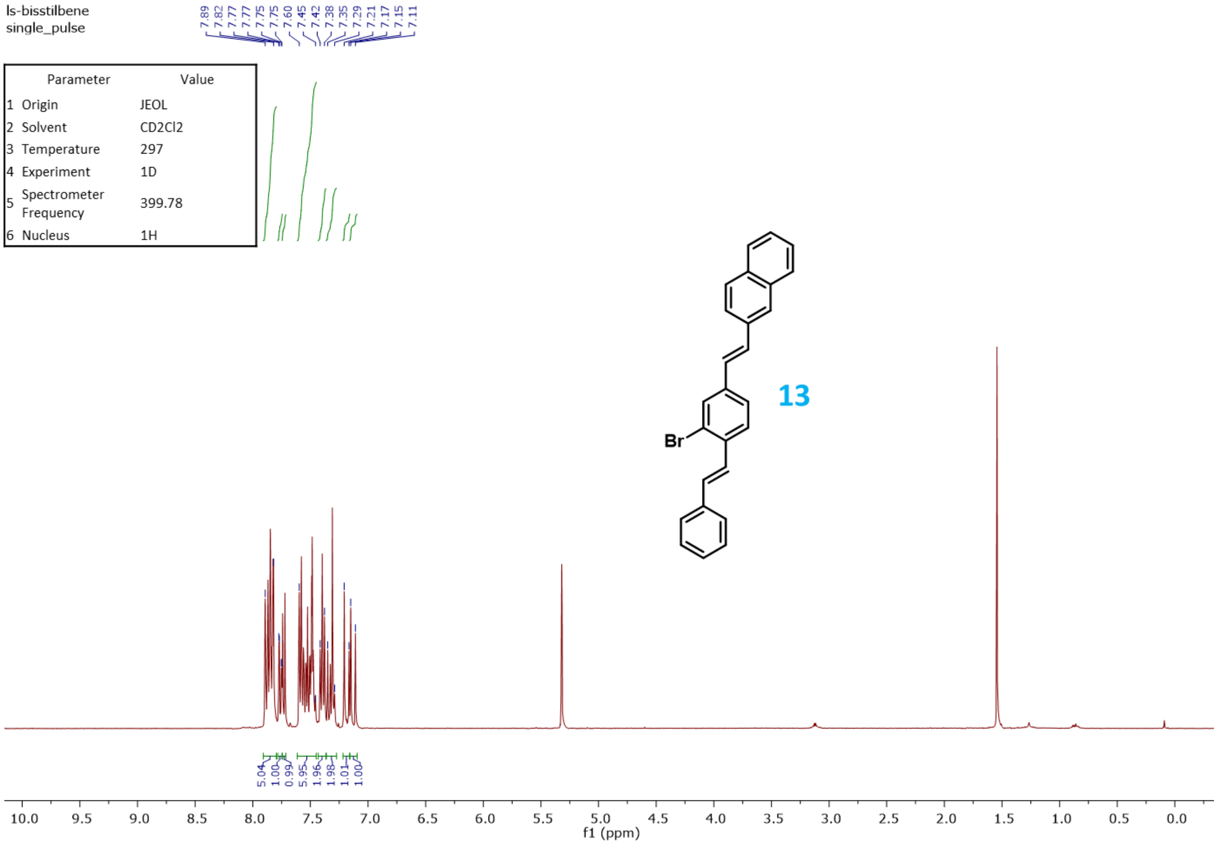

ls-bisstilbene
APT Experiment

	Parameter	Value
1	Origin	JEOL
2	Solvent	CD2Cl2
3	Temperature	297
4	Experiment	JMOD
5	Spectrometer Frequency	100.53
6	Nucleus	13C

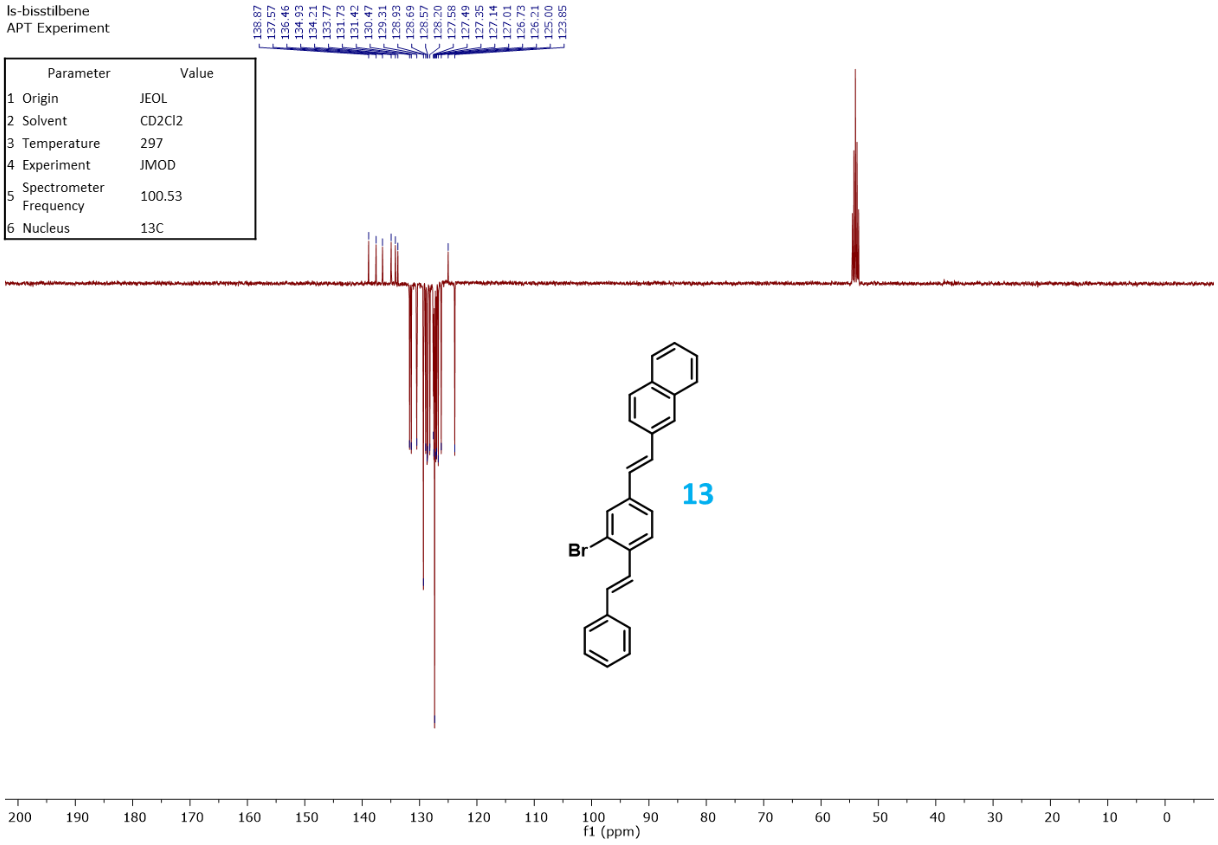

ls-6Helicene pur
single_pulse

Parameter		Value
1	Origin	JEOL
2	Solvent	CD2Cl2
3	Temperature	297
4	Experiment	1D
5	Spectrometer Frequency	399.78
6	Nucleus	1H

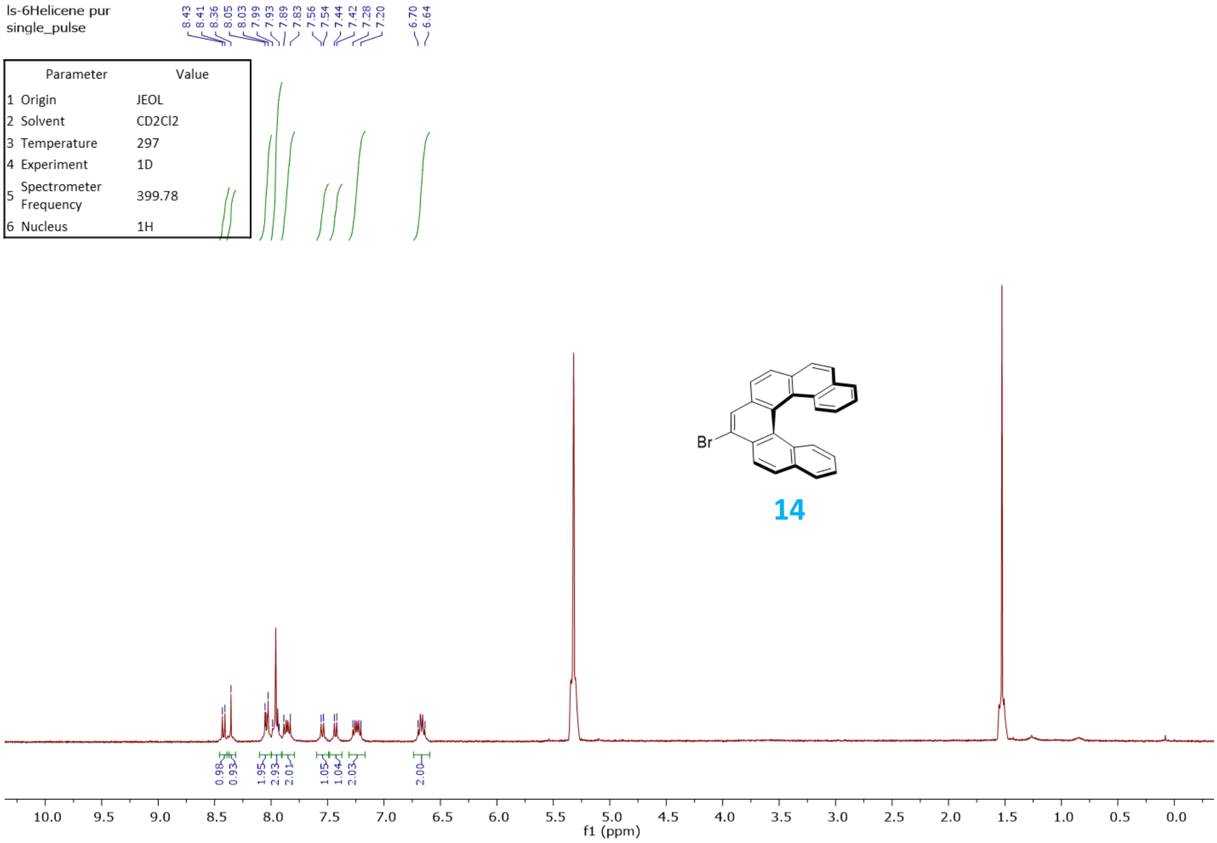

ls-6Helicene again apt
APT Experiment

Parameter		Value
1	Origin	JEOL
2	Solvent	CD2Cl2
3	Temperature	297
4	Experiment	JMOD
5	Spectrometer Frequency	100.53
6	Nucleus	13C

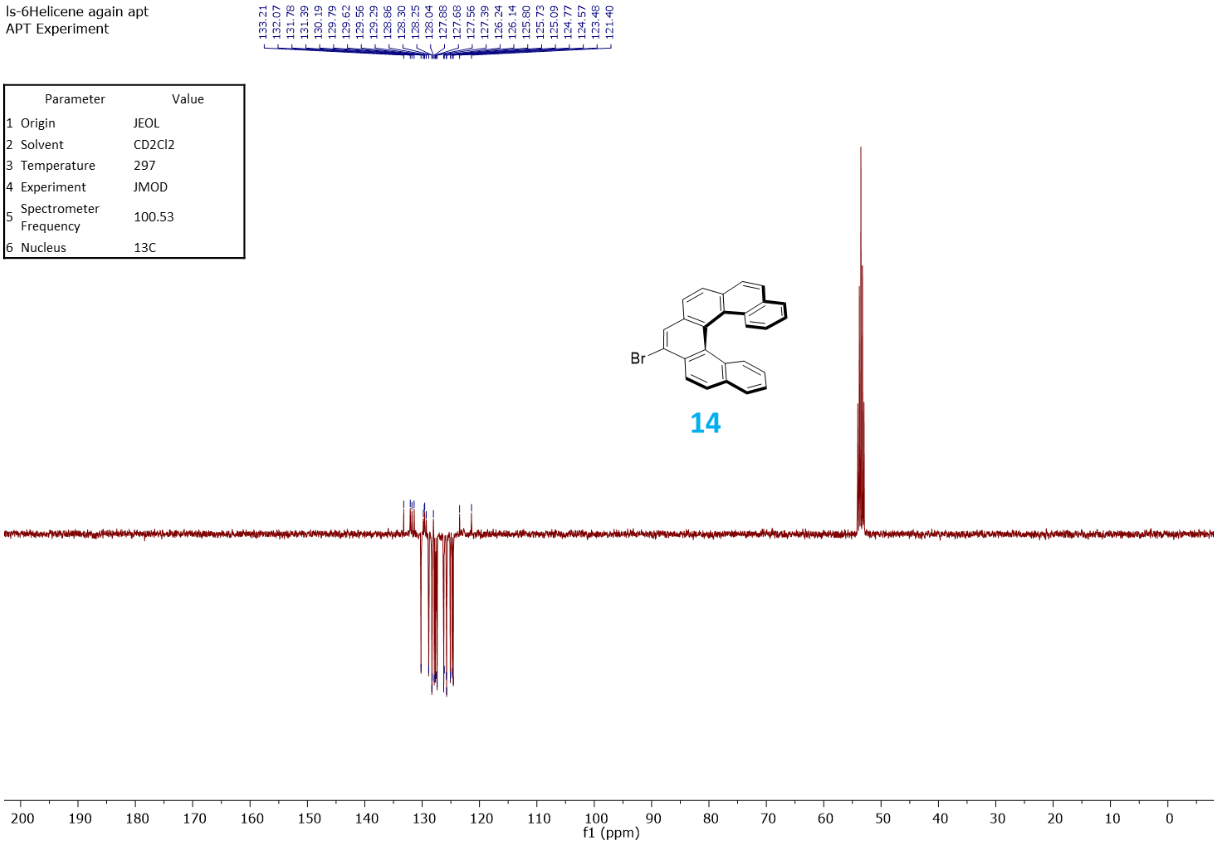

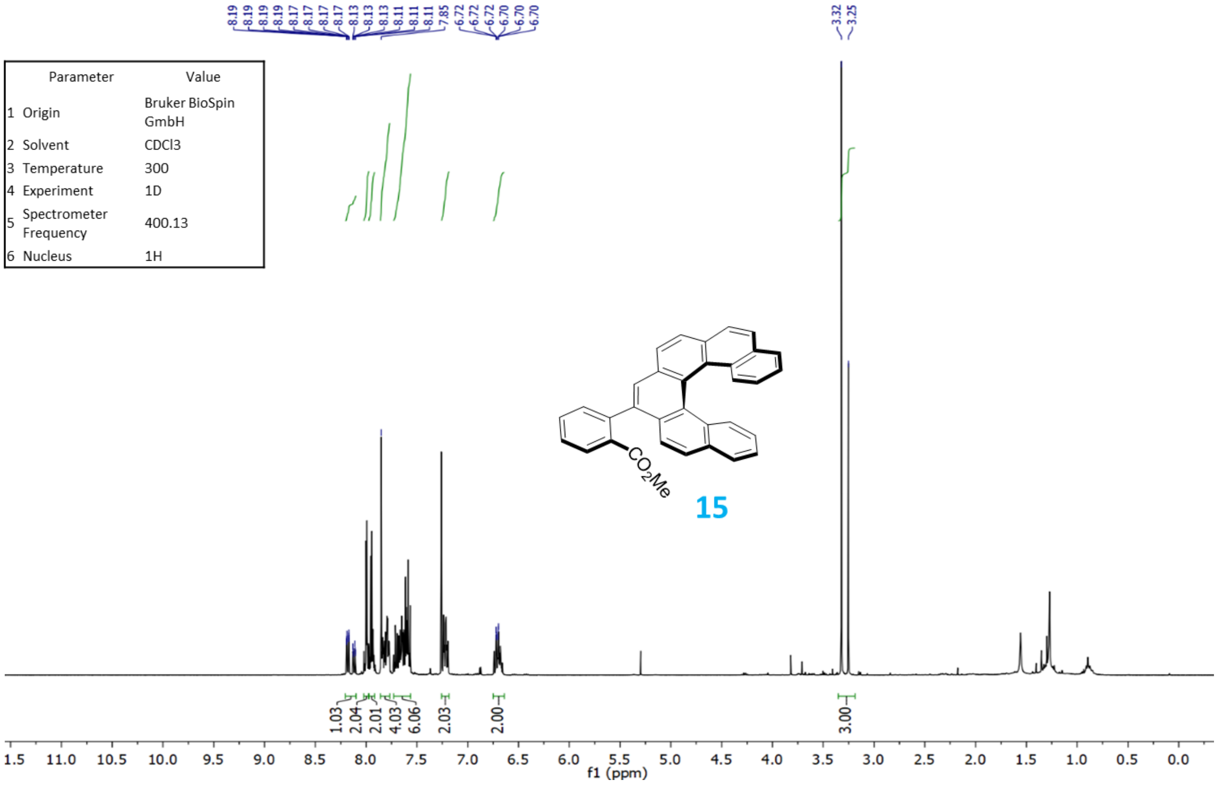

	Parameter	Value
1	Origin	Bruker BioSpin GmbH
2	Solvent	CDCl3
3	Temperature	300
4	Experiment	1D
5	Spectrometer Frequency	400.13
6	Nucleus	1H

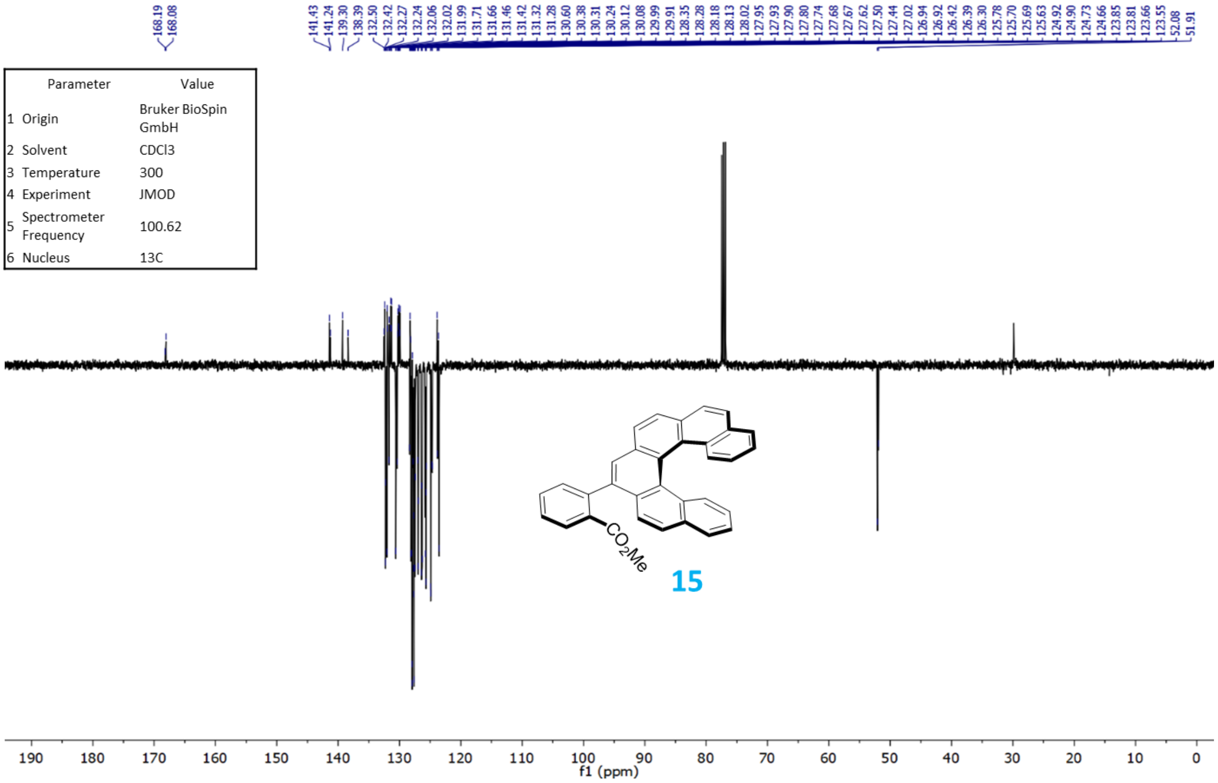

	Parameter	Value
1	Origin	Bruker BioSpin GmbH
2	Solvent	CDCl3
3	Temperature	300
4	Experiment	JMOD
5	Spectrometer Frequency	100.62
6	Nucleus	13C

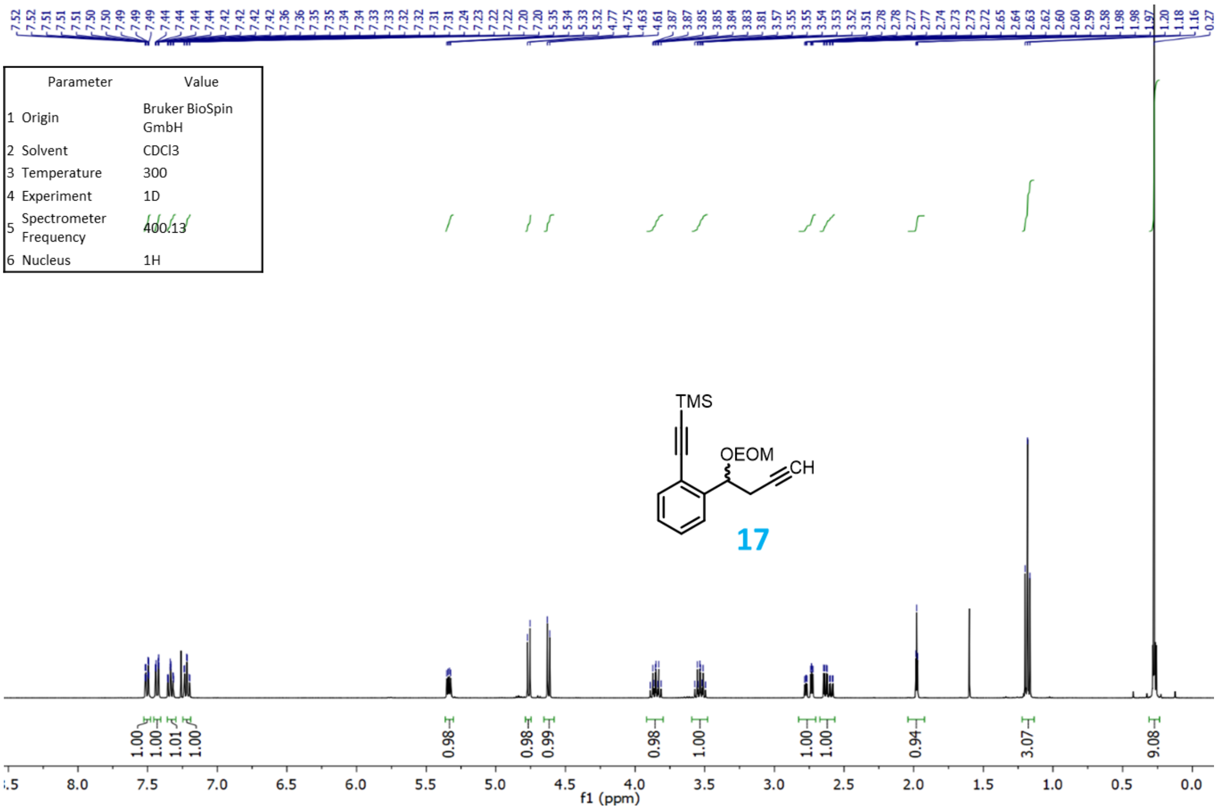
Parameter | Value
1 Origin | Bruker BioSpin GmbH
2 Solvent | CDCl3
3 Temperature | 300
4 Experiment | 1D
5 Spectrometer Frequency | 400.13
6 Nucleus | 1H
TMS
OEOM
CH
17
1.00 1.00 1.01 1.00 0.98 0.98 0.99 0.98 1.00 1.00 1.00 0.94 3.07 9.08
f1 (ppm)

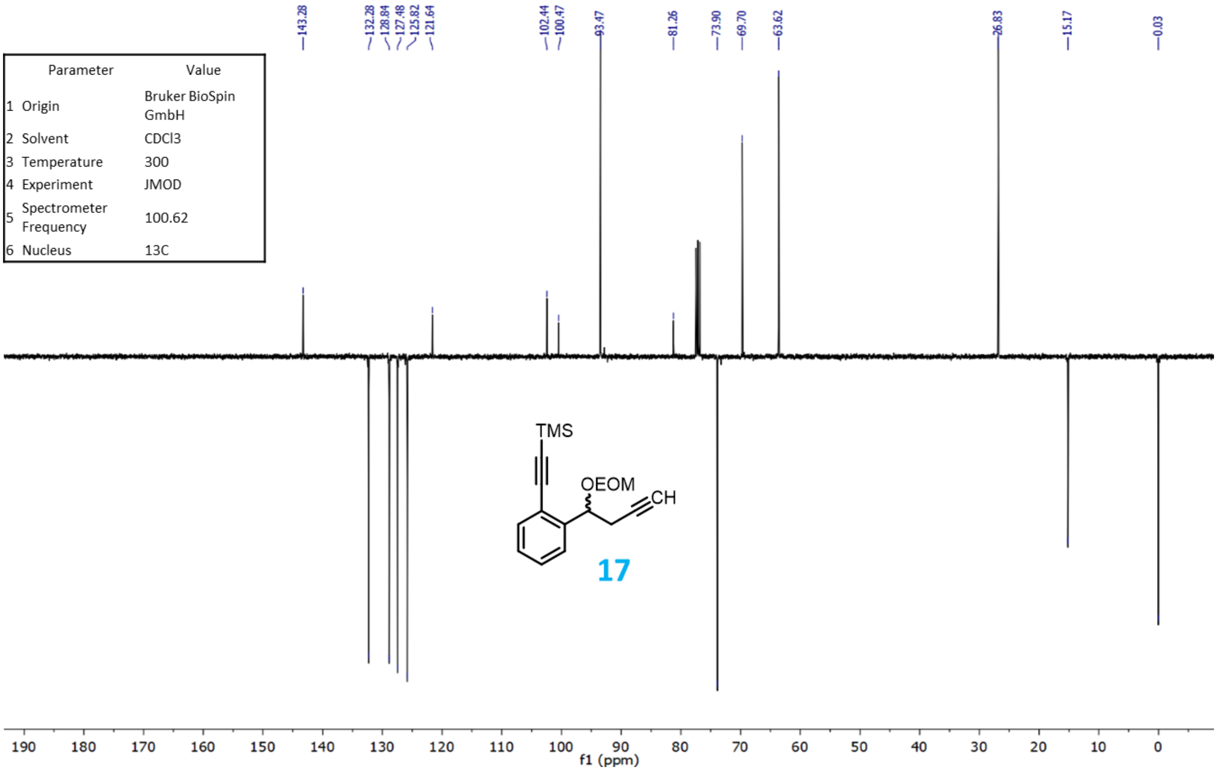
143.28
132.28 128.84 127.48 125.82 121.64
102.44 100.47
93.47
81.26
73.90
69.70
63.62
26.83
15.17
0.03
Parameter | Value
1 Origin | Bruker BioSpin GmbH
2 Solvent | CDCl3
3 Temperature | 300
4 Experiment | JMOD
5 Spectrometer Frequency | 100.62
6 Nucleus | 13C
TMS
OEOM
CH
17
f1 (ppm)

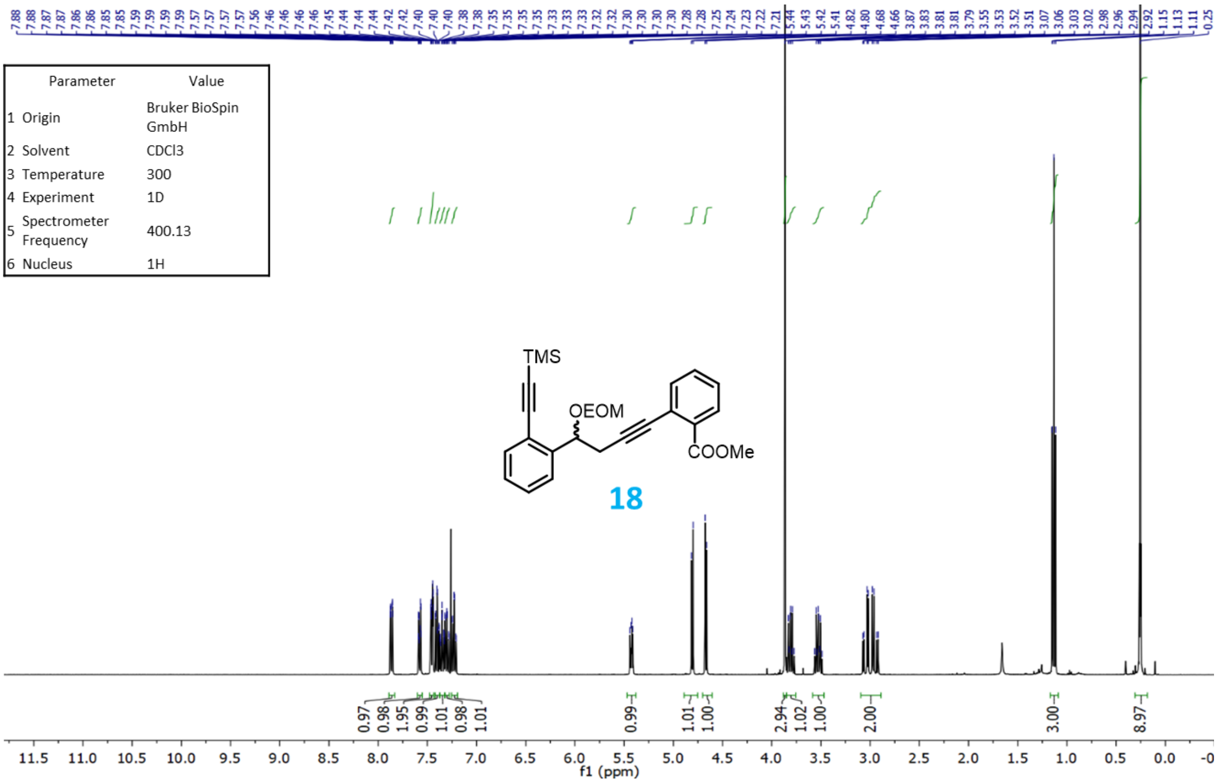

Parameter | Value
1 Origin | Bruker BioSpin GmbH
2 Solvent | CDCl3
3 Temperature | 300
4 Experiment | 1D
5 Spectrometer Frequency | 400.13
6 Nucleus | 1H
TMS
OEOM
COOMe
18
0.97 0.98 1.95 0.99 1.01 0.98 1.01
0.99
1.01 1.00
2.94 1.02 1.00
2.00
3.00
8.97
f1 (ppm)

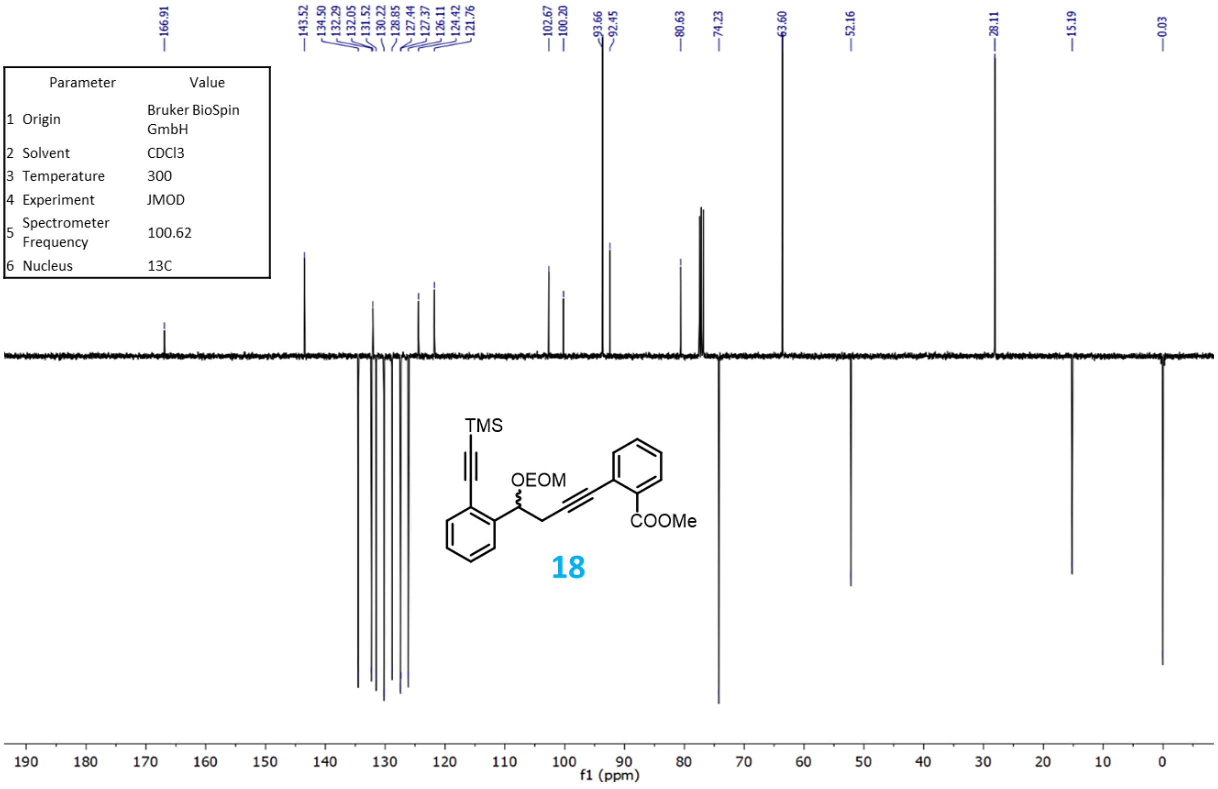

166.91
143.52 134.50 132.29 132.05 131.52 130.22 128.85 127.44 127.37 126.11 124.42 121.76
102.67 100.20 93.66 92.45
80.63 74.23
63.60
52.16
28.11
15.19
-0.03
Parameter | Value
1 Origin | Bruker BioSpin GmbH
2 Solvent | CDCl3
3 Temperature | 300
4 Experiment | JMOD
5 Spectrometer Frequency | 100.62
6 Nucleus | 13C
TMS
OEOM
COOMe
18
f1 (ppm)

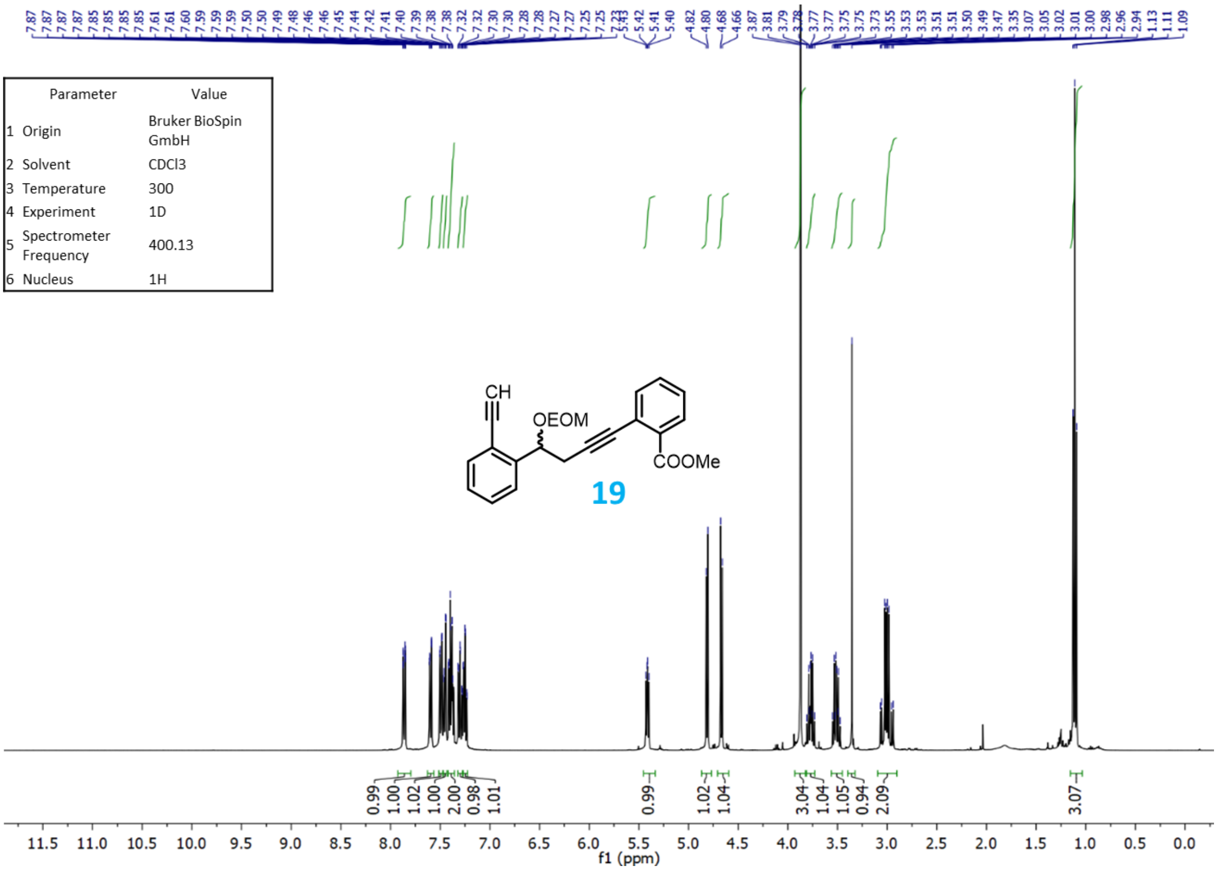

	Parameter	Value
1	Origin	Bruker BioSpin GmbH
2	Solvent	CDCl3
3	Temperature	300
4	Experiment	1D
5	Spectrometer Frequency	400.13
6	Nucleus	1H

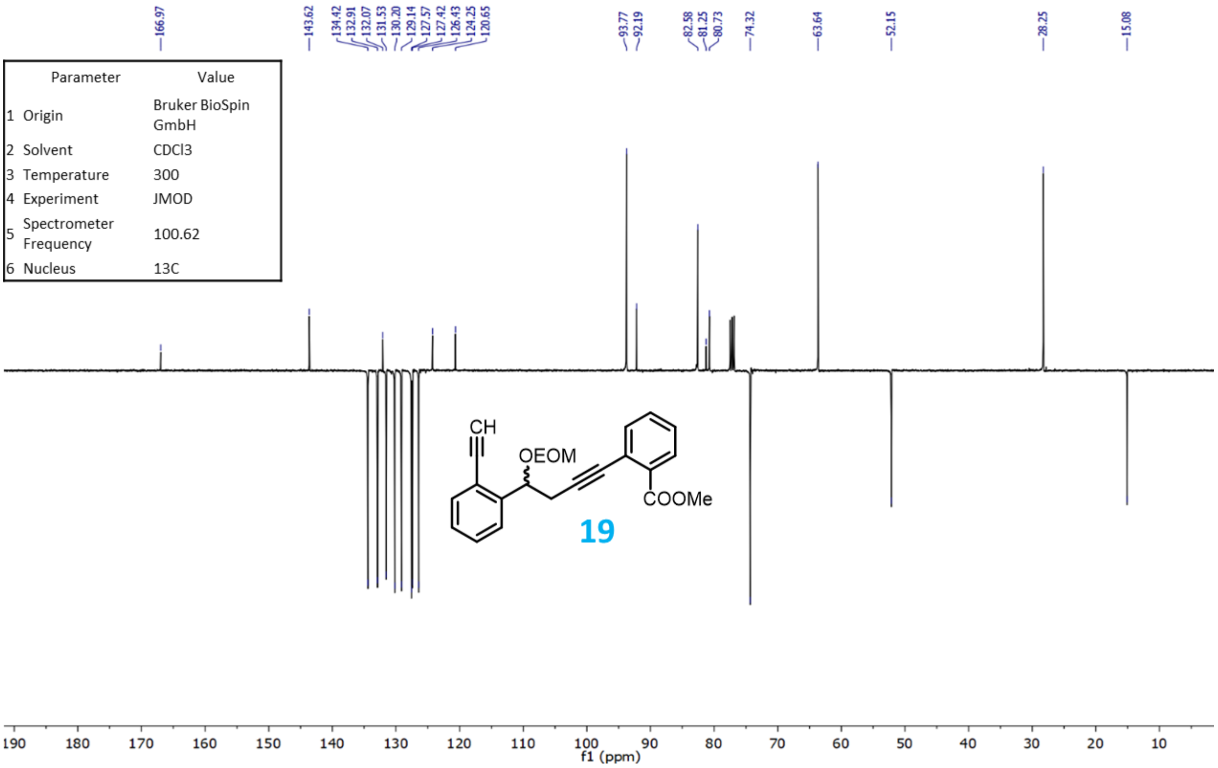

	Parameter	Value
1	Origin	Bruker BioSpin GmbH
2	Solvent	CDCl3
3	Temperature	300
4	Experiment	JMOD
5	Spectrometer Frequency	100.62
6	Nucleus	13C

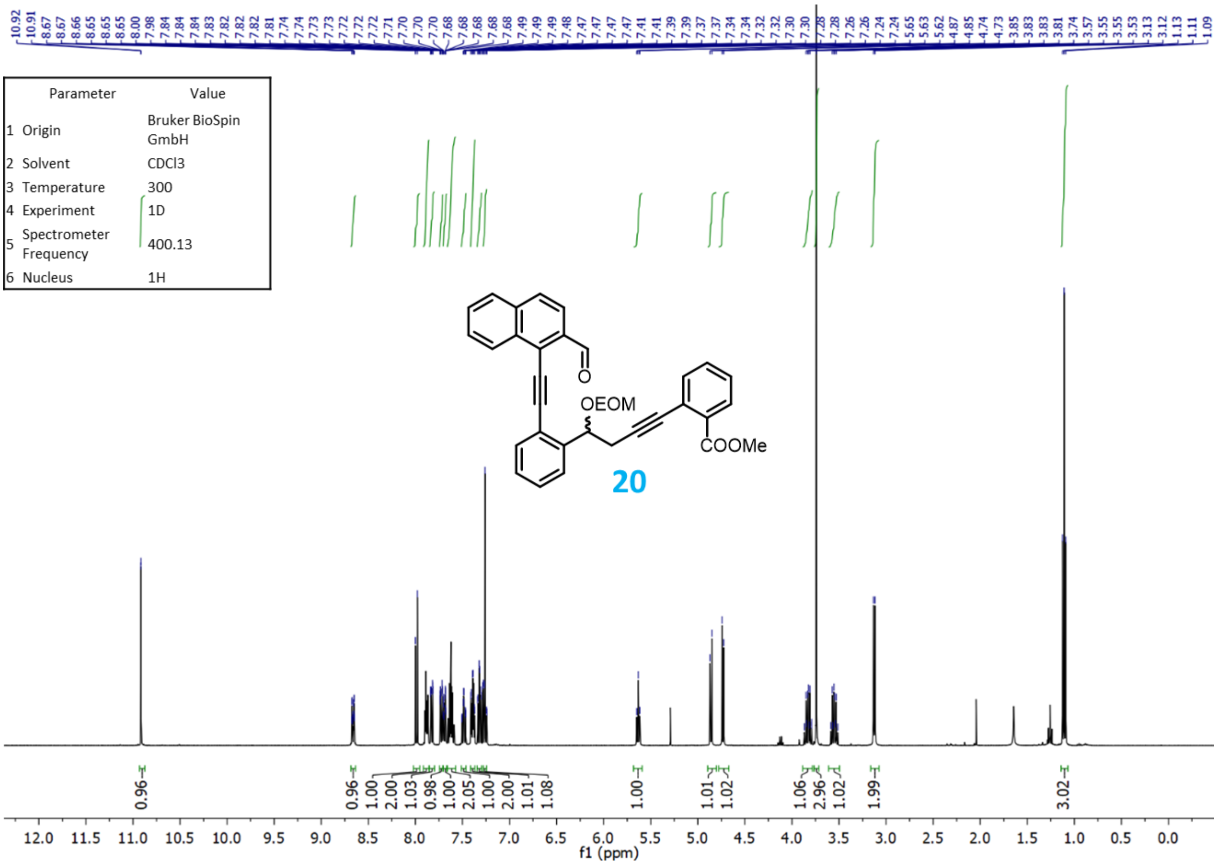
10.92 10.91 8.67 8.67 8.66 8.65 8.65 8.65 8.00 7.98 7.84 7.84 7.84 7.83 7.82 7.82 7.82 7.81 7.74 7.74 7.73 7.73 7.72 7.72 7.72 7.71 7.70 7.70 7.70 7.68 7.68 7.68 7.68 7.68 7.49 7.49 7.49 7.48 7.47 7.47 7.47 7.47 7.41 7.41 7.39 7.39 7.37 7.37 7.34 7.34 7.32 7.32 7.30 7.30 7.28 7.28 7.26 7.26 7.24 7.24 5.65 5.63 5.63 5.62 4.87 4.85 4.74 4.73 3.85 3.83 3.83 3.81 3.74 3.57 3.55 3.55 3.53 3.13 3.12 1.13 1.11 1.09
Parameter | Value
1 Origin | Bruker BioSpin GmbH
2 Solvent | CDCl3
3 Temperature | 300
4 Experiment | 1D
5 Spectrometer Frequency | 400.13
6 Nucleus | 1H
OEOM
COOMe
20
0.96 0.96 1.00 2.00 1.03 0.98 1.00 2.05 1.00 2.00 1.01 1.08 1.00 1.01 1.02 1.06 2.96 1.02 1.99 3.02
12.0 11.5 11.0 10.5 10.0 9.5 9.0 8.5 8.0 7.5 7.0 6.5 6.0 5.5 5.0 4.5 4.0 3.5 3.0 2.5 2.0 1.5 1.0 0.5 0.0
f1 (ppm)

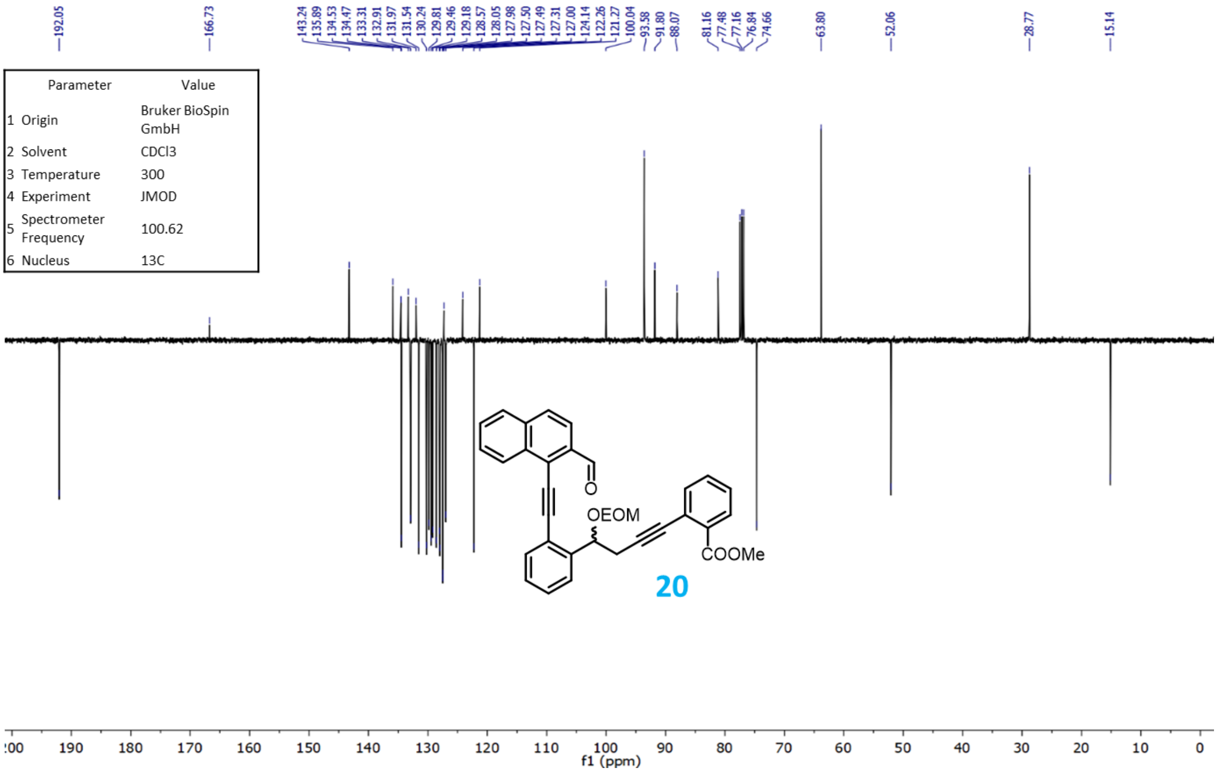
192.05 166.73 143.24 135.89 134.53 134.47 133.31 132.91 131.97 131.54 130.24 129.81 129.46 129.18 128.57 128.05 127.98 127.50 127.49 127.31 127.00 124.14 122.26 121.27 100.04 93.58 91.80 88.07 81.16 77.48 77.16 76.84 74.66 63.80 52.06 28.77 15.14
Parameter | Value
1 Origin | Bruker BioSpin GmbH
2 Solvent | CDCl3
3 Temperature | 300
4 Experiment | JMOD
5 Spectrometer Frequency | 100.62
6 Nucleus | 13C
OEOM
COOMe
20
200 190 180 170 160 150 140 130 120 110 100 90 80 70 60 50 40 30 20 10 0
f1 (ppm)

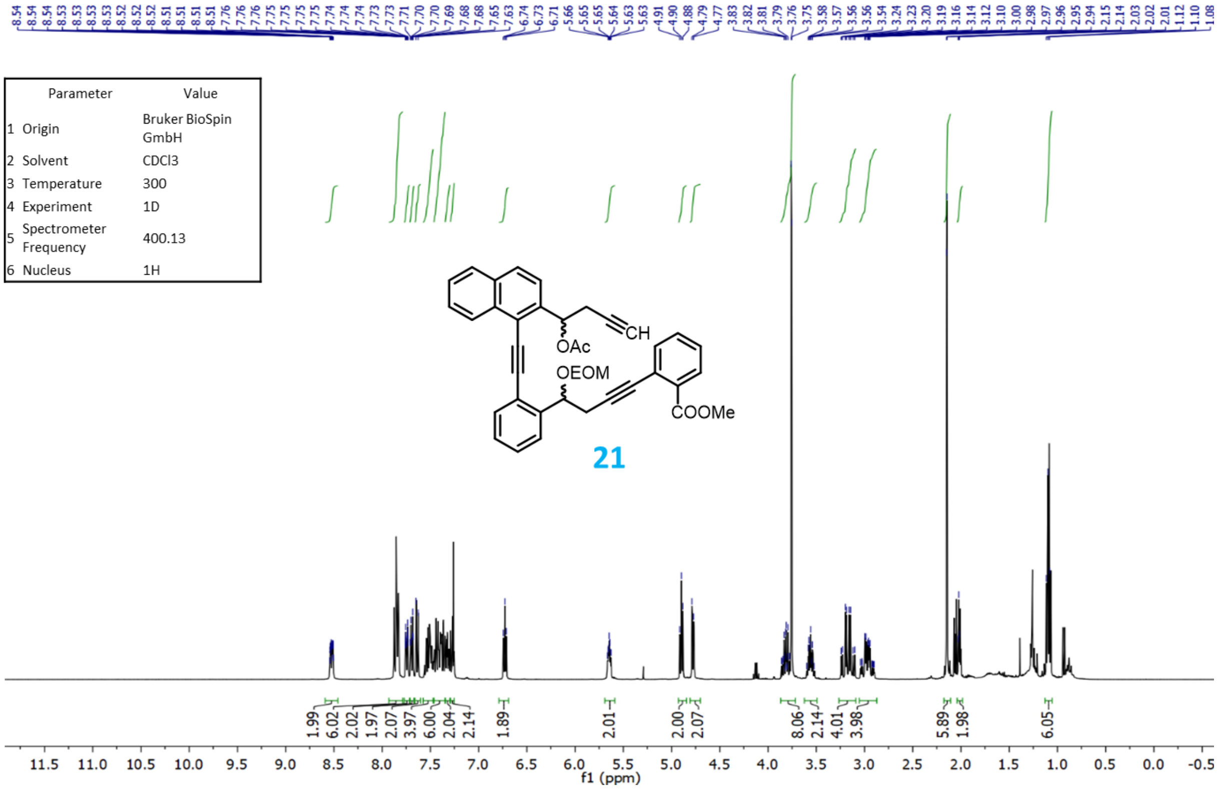

	Parameter	Value
1	Origin	Bruker BioSpin GmbH
2	Solvent	CDCl3
3	Temperature	300
4	Experiment	1D
5	Spectrometer Frequency	400.13
6	Nucleus	1H

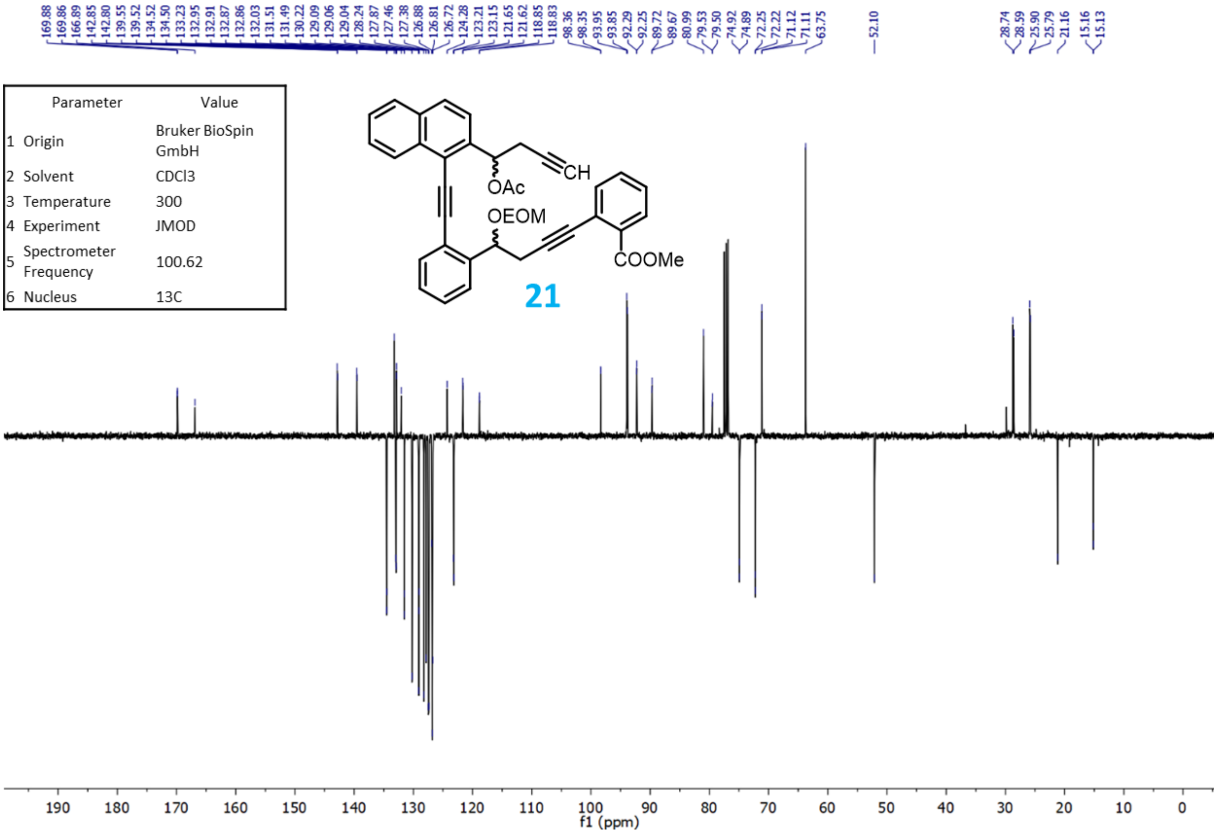

	Parameter	Value
1	Origin	Bruker BioSpin GmbH
2	Solvent	CDCl3
3	Temperature	300
4	Experiment	JMOD
5	Spectrometer Frequency	100.62
6	Nucleus	13C

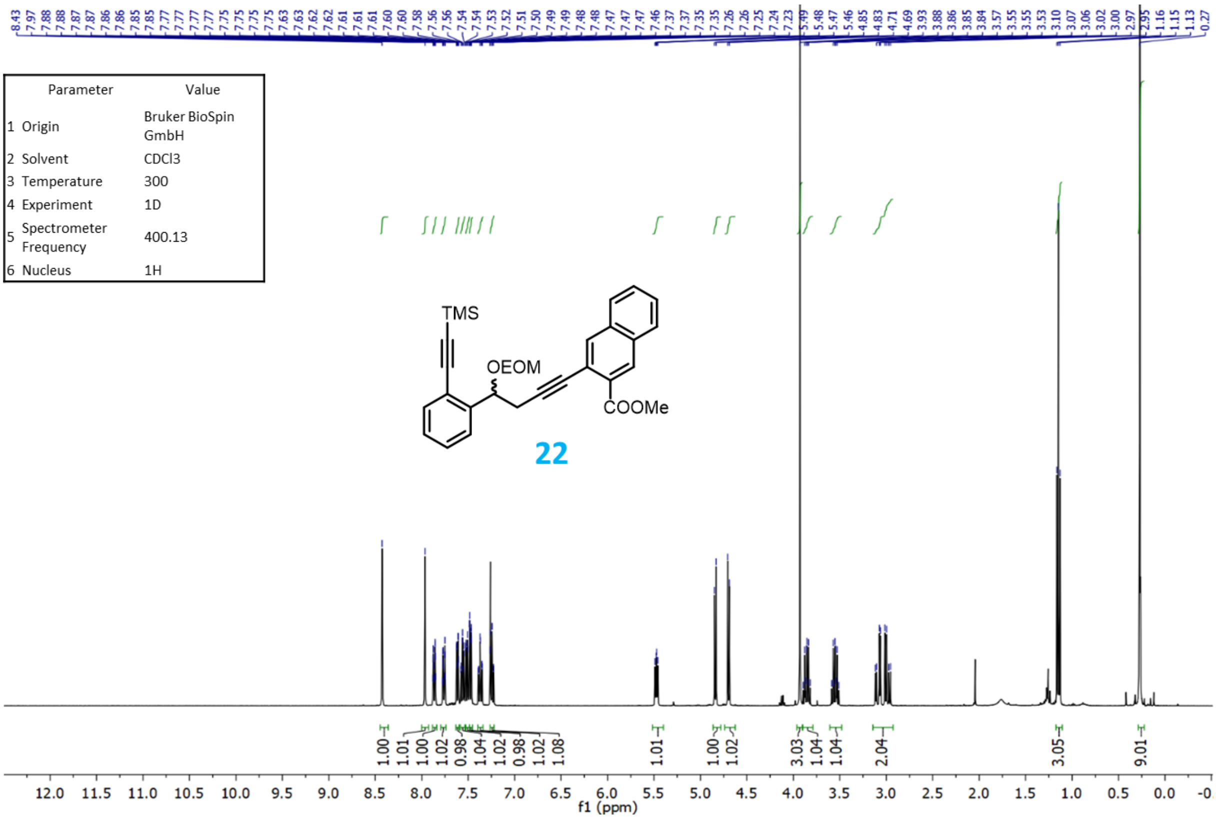

	Parameter	Value
1	Origin	Bruker BioSpin GmbH
2	Solvent	CDCl3
3	Temperature	300
4	Experiment	1D
5	Spectrometer Frequency	400.13
6	Nucleus	1H

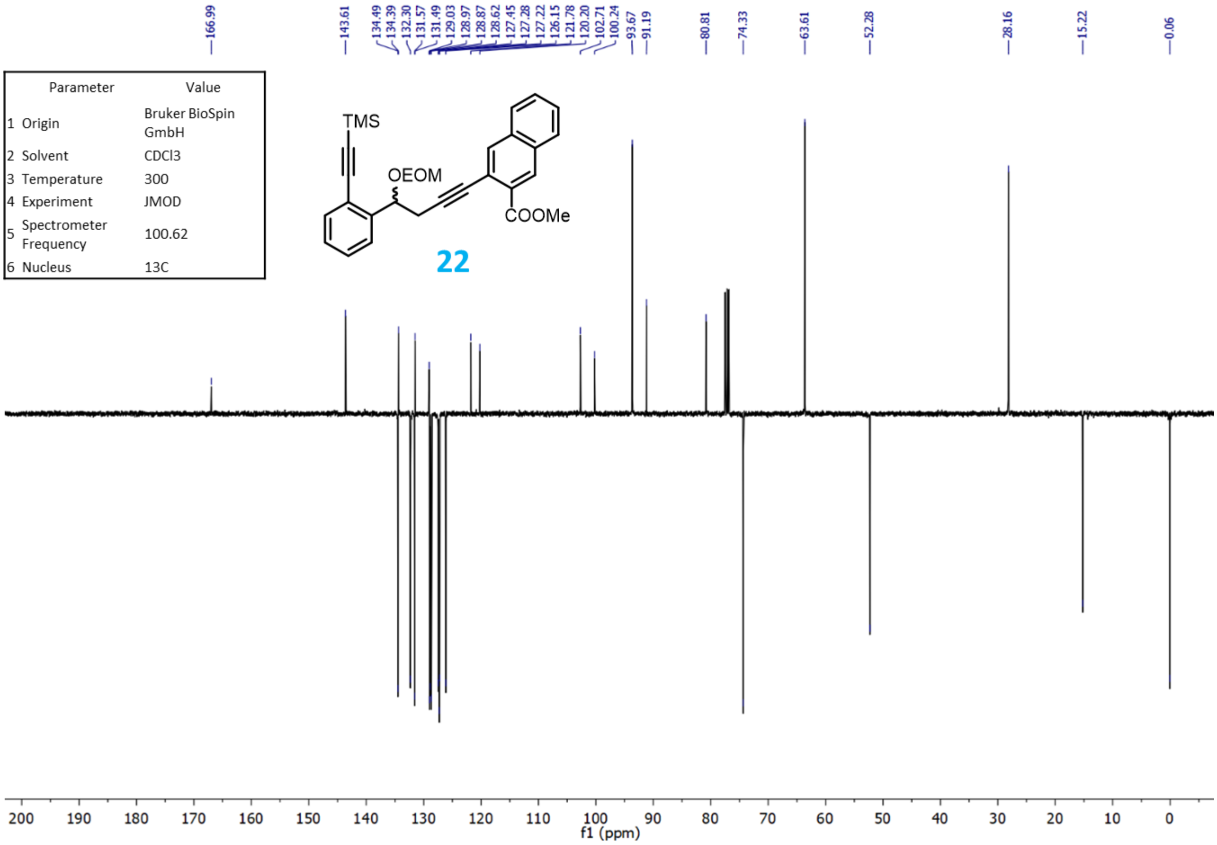

	Parameter	Value
1	Origin	Bruker BioSpin GmbH
2	Solvent	CDCl3
3	Temperature	300
4	Experiment	JMOD
5	Spectrometer Frequency	100.62
6	Nucleus	13C

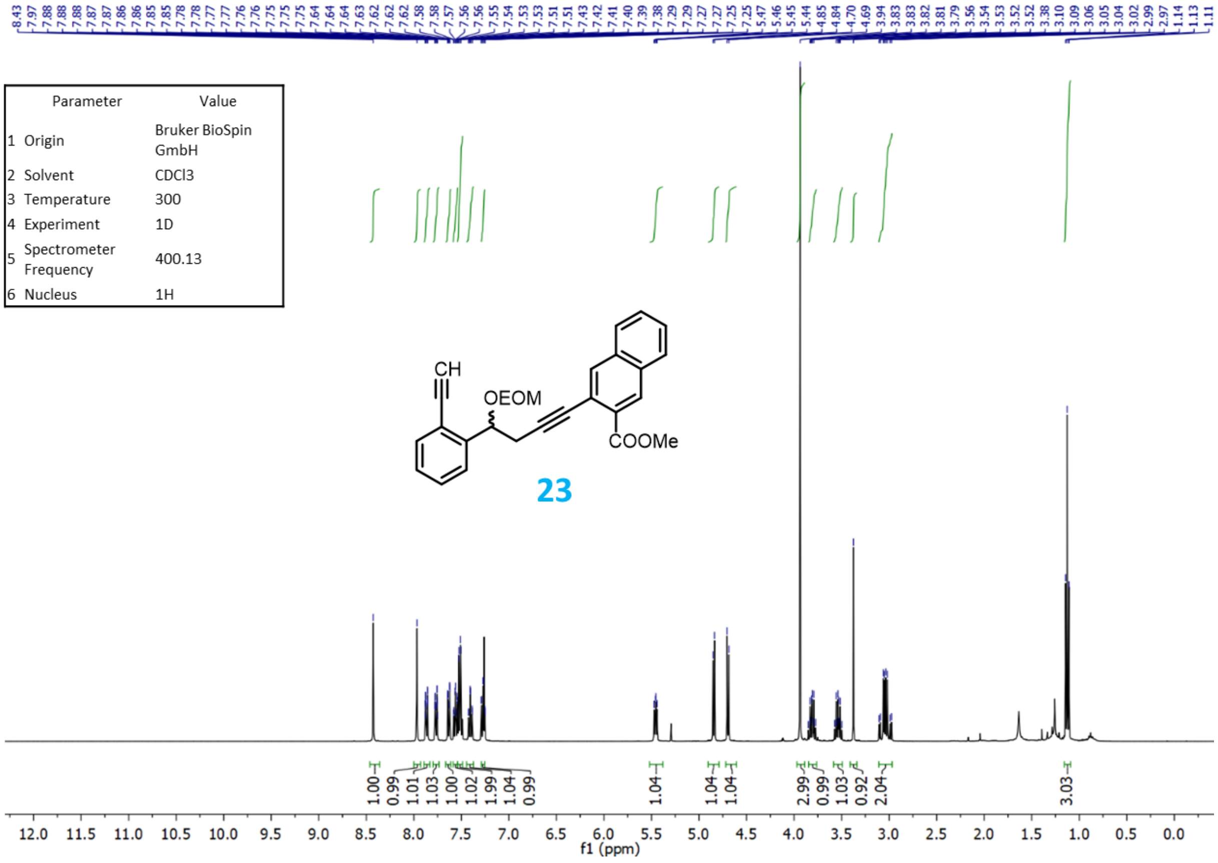
Parameter Value
1 Origin Bruker BioSpin GmbH
2 Solvent CDCl3
3 Temperature 300
4 Experiment 1D
5 Spectrometer Frequency 400.13
6 Nucleus 1H
CH
OEOM
COOMe
23
f1 (ppm)

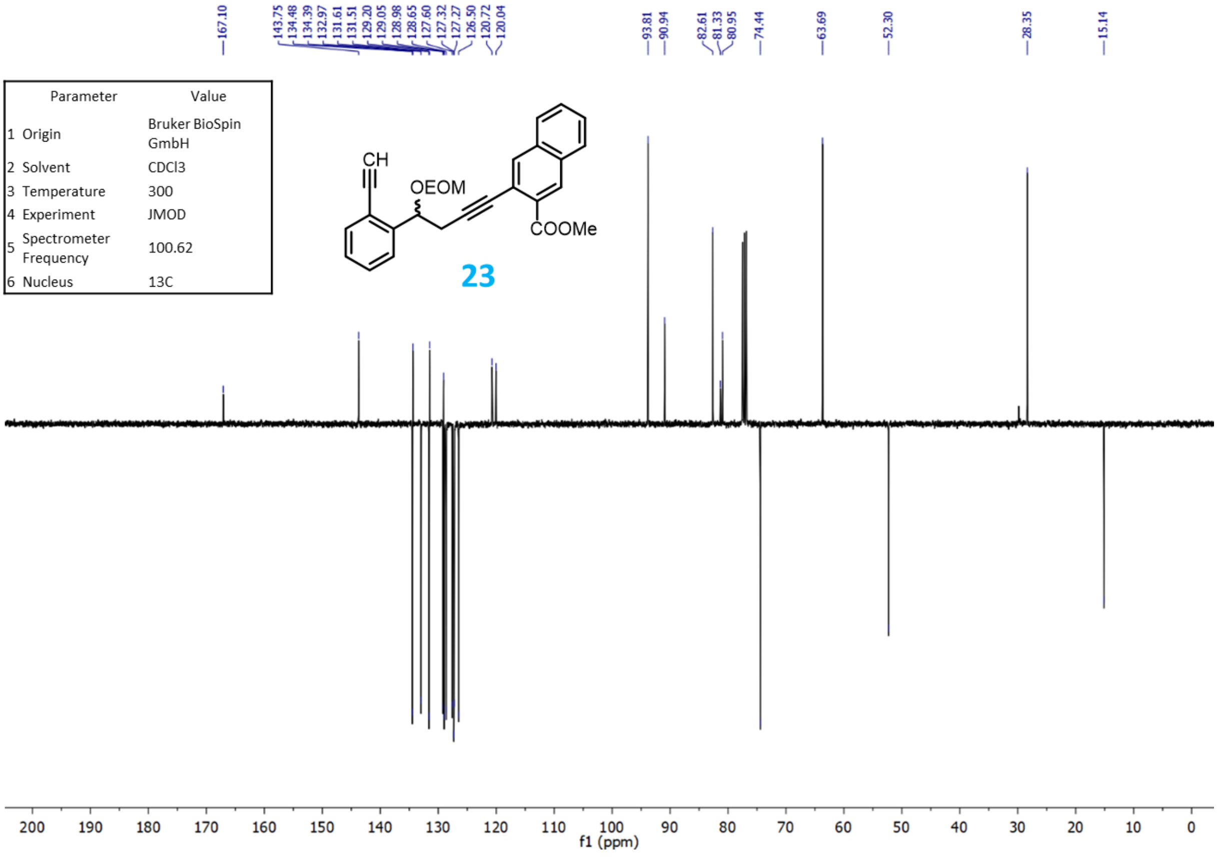
Parameter Value
1 Origin Bruker BioSpin GmbH
2 Solvent CDCl3
3 Temperature 300
4 Experiment JMOD
5 Spectrometer Frequency 100.62
6 Nucleus 13C
CH
OEOM
COOMe
23
f1 (ppm)

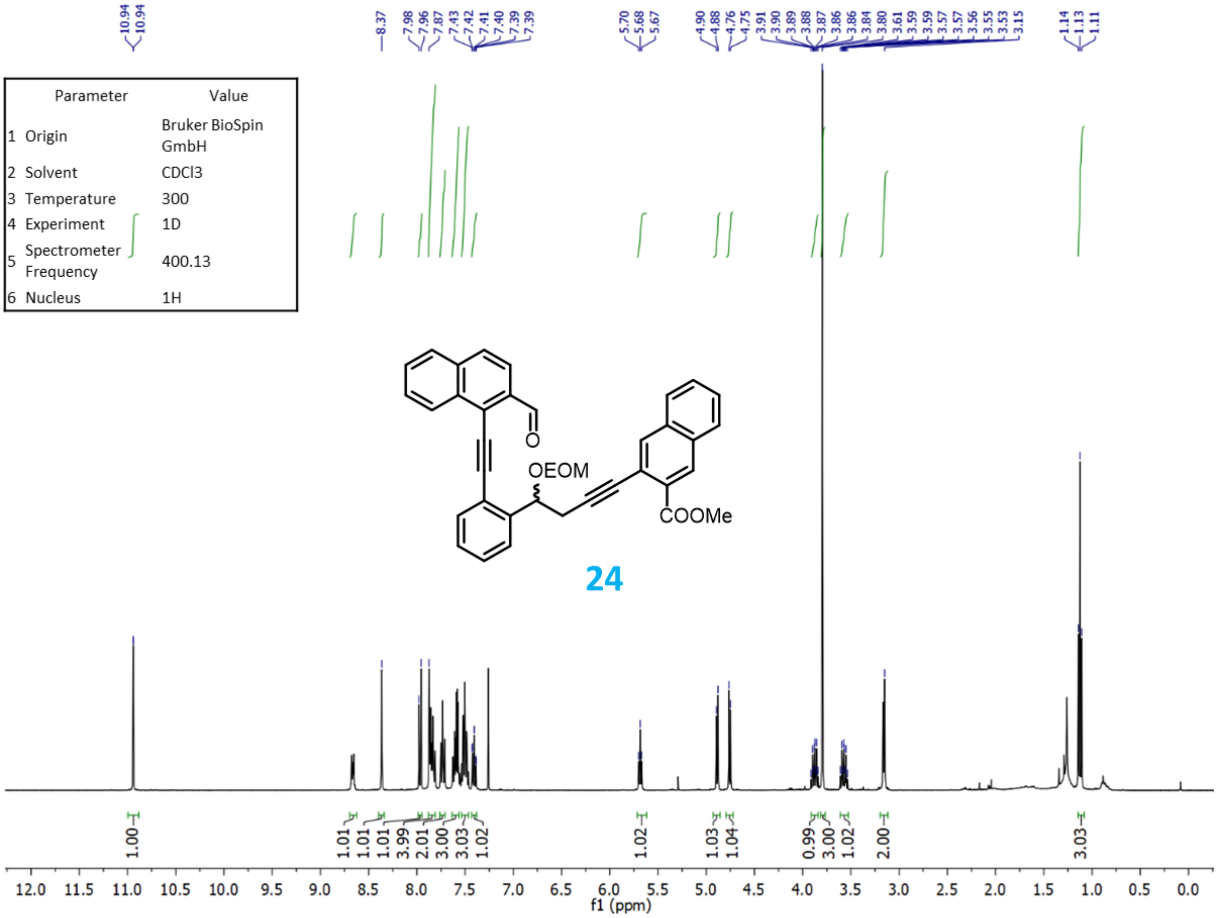
Parameter Value
1 Origin Bruker BioSpin GmbH
2 Solvent CDCl3
3 Temperature 300
4 Experiment 1D
5 Spectrometer Frequency 400.13
6 Nucleus 1H
OEOM
COOMe
O
24
f1 (ppm)

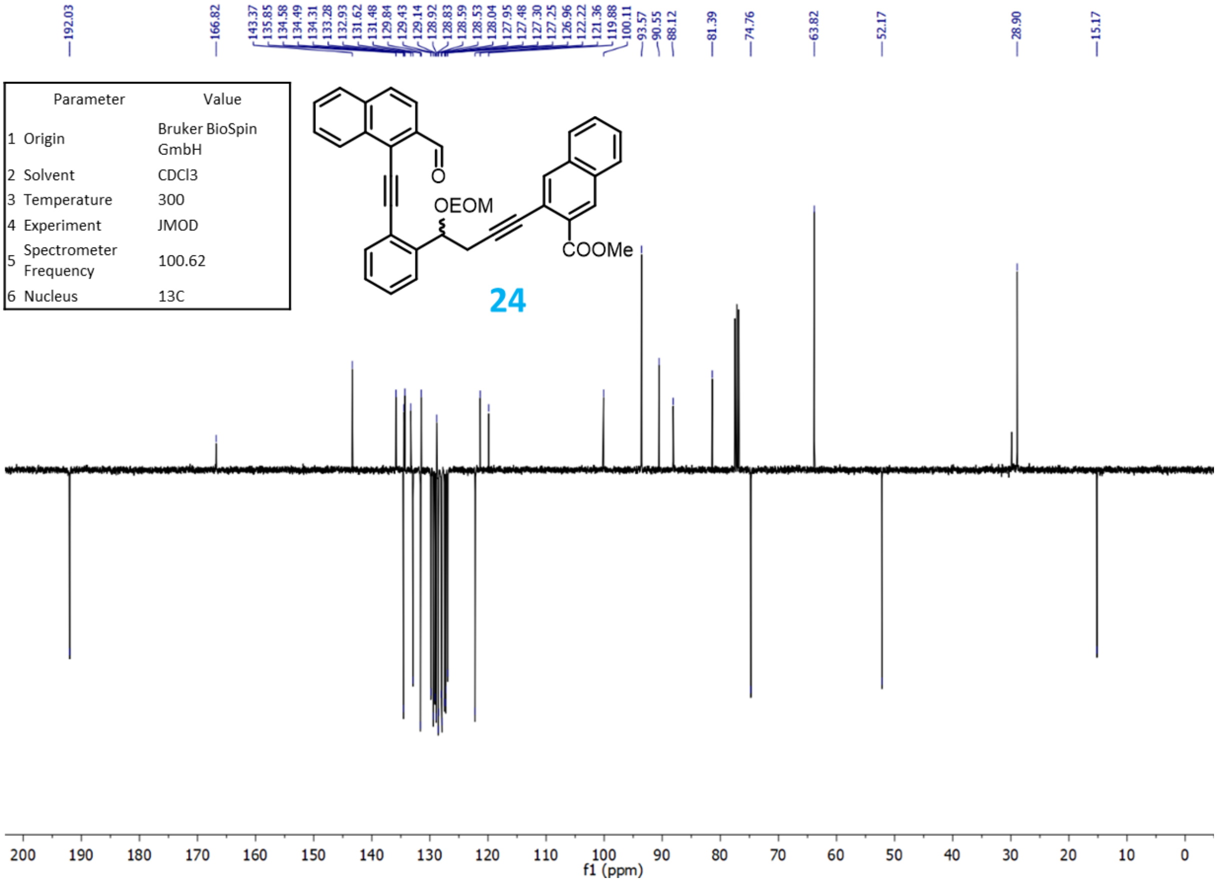
Parameter Value
1 Origin Bruker BioSpin GmbH
2 Solvent CDCl3
3 Temperature 300
4 Experiment JMOD
5 Spectrometer Frequency 100.62
6 Nucleus 13C
OEOM
COOMe
O
24
f1 (ppm)

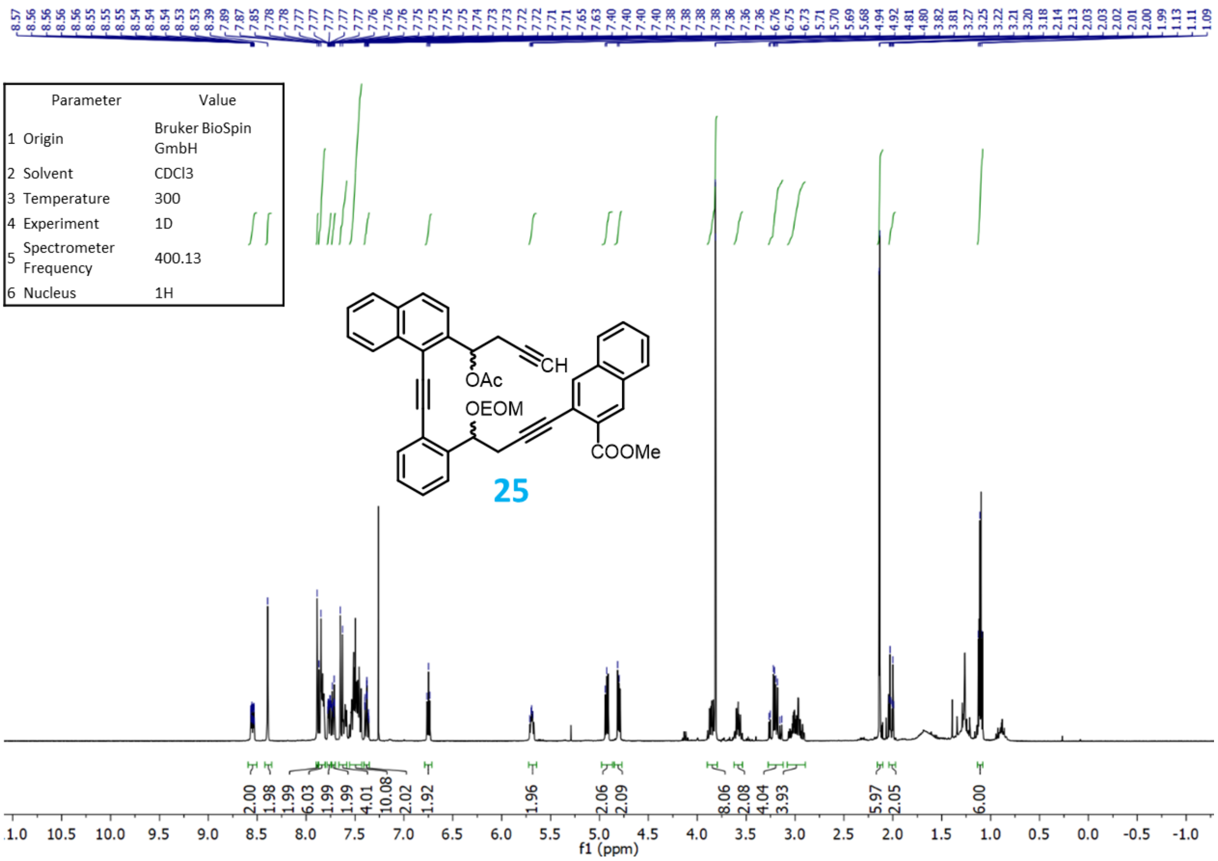
8.57 8.56 8.56 8.56 8.56 8.55 8.55 8.55 8.54 8.54 8.54 8.53 8.53 8.39 7.89 7.87 7.85 7.78 7.78 7.77 7.77 7.77 7.77 7.77 7.76 7.76 7.76 7.75 7.75 7.75 7.75 7.74 7.73 7.73 7.72 7.72 7.71 7.71 7.65 7.63 7.40 7.40 7.40 7.40 7.38 7.38 7.38 7.38 7.36 7.36 7.36 6.76 6.75 6.73 5.71 5.70 5.69 5.68 4.94 4.92 4.81 4.80 3.82 3.81 3.27 3.25 3.22 3.21 3.20 3.18 2.14 2.13 2.03 2.03 2.02 2.01 2.00 1.99 1.13 1.11 1.09
Parameter Value
1 Origin Bruker BioSpin GmbH
2 Solvent CDCl3
3 Temperature 300
4 Experiment 1D
5 Spectrometer Frequency 400.13
6 Nucleus 1H
OAc
CH
OEOM
COOMe
25
2.00 1.98 1.99 6.03 1.99 1.99 4.01 10.08 2.02 1.92 1.96 2.06 2.09 8.06 2.08 4.04 3.93 5.97 2.05 6.00
f1 (ppm)

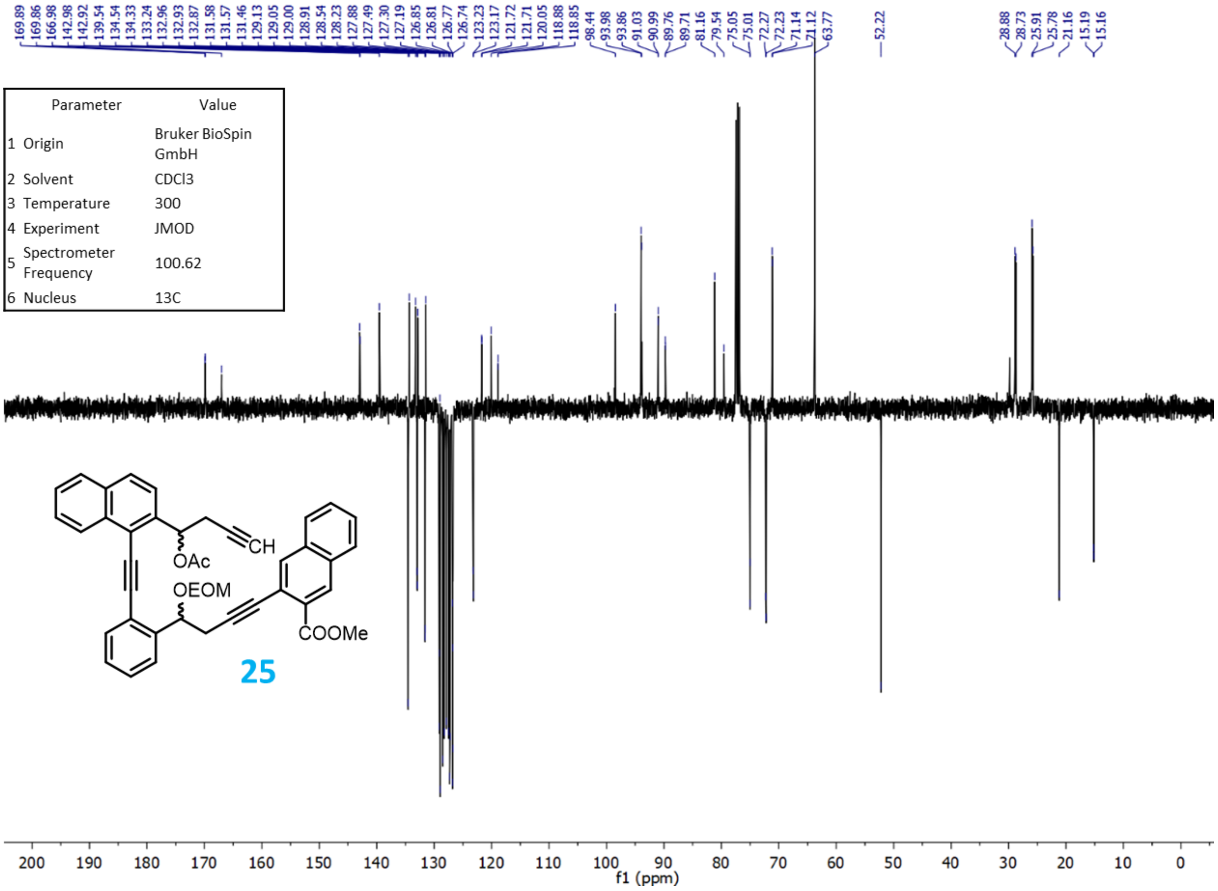
169.89 169.86 166.98 142.98 142.92 139.54 134.54 134.33 133.24 132.96 132.93 132.87 131.58 131.57 131.46 129.13 129.05 129.00 128.91 128.54 128.23 127.88 127.49 127.30 127.19 126.85 126.81 126.77 126.74 123.23 123.17 121.72 121.71 120.05 118.88 118.85 98.44 93.98 93.86 91.03 90.99 89.76 89.71 81.16 79.54 75.05 75.01 72.27 72.23 71.14 71.12 63.77 52.22 28.88 28.73 25.91 25.78 21.16 15.19 15.16
Parameter Value
1 Origin Bruker BioSpin GmbH
2 Solvent CDCl3
3 Temperature 300
4 Experiment JMOD
5 Spectrometer Frequency 100.62
6 Nucleus 13C
OAc
CH
OEOM
COOMe
25
f1 (ppm)

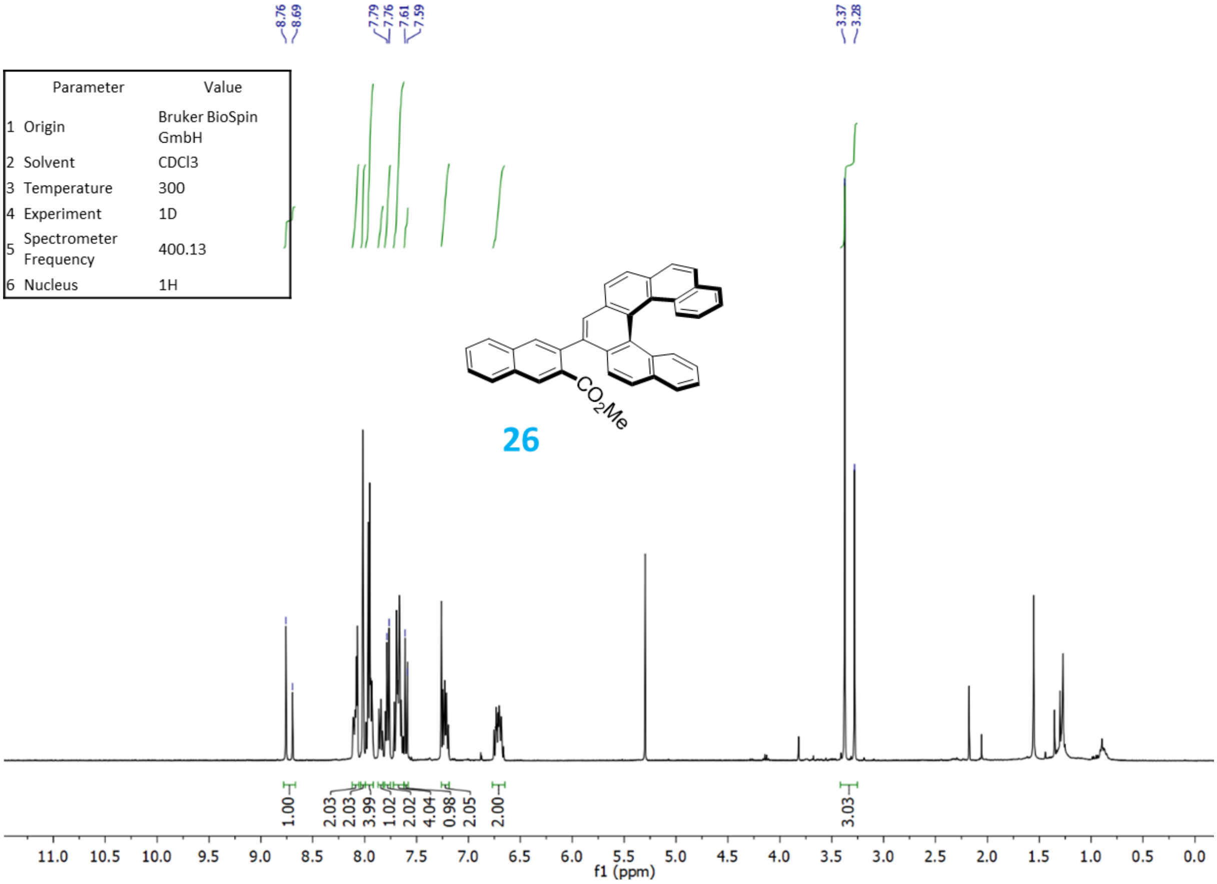

Parameter		Value
1	Origin	Bruker BioSpin GmbH
2	Solvent	CDCl3
3	Temperature	300
4	Experiment	1D
5	Spectrometer Frequency	400.13
6	Nucleus	1H

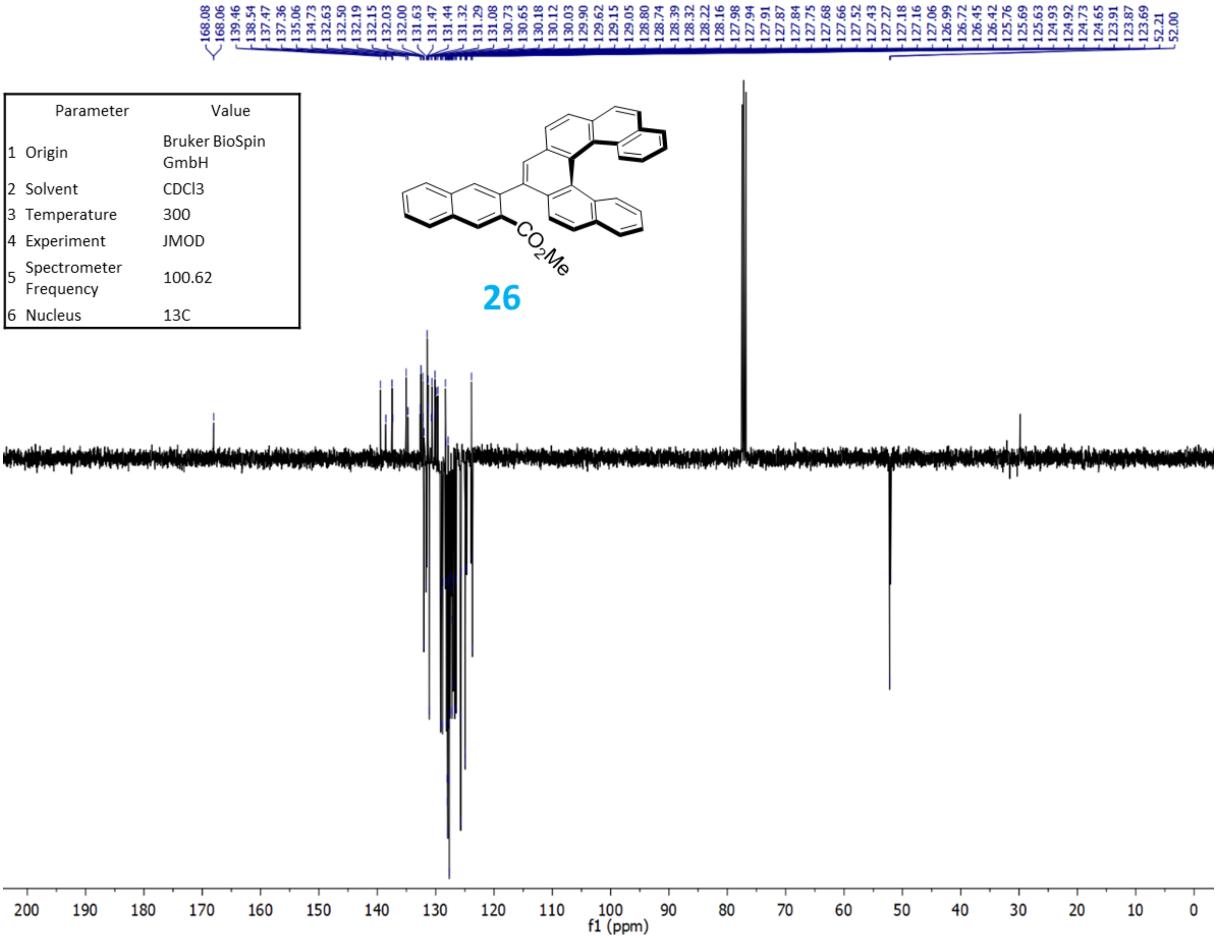

Parameter		Value
1	Origin	Bruker BioSpin GmbH
2	Solvent	CDCl3
3	Temperature	300
4	Experiment	JMOD
5	Spectrometer Frequency	100.62
6	Nucleus	13C

	Parameter	Value
1	Origin	Bruker BioSpin GmbH
2	Solvent	CDCl3
3	Temperature	300
4	Experiment	1D
5	Spectrometer Frequency	400.13
6	Nucleus	1H

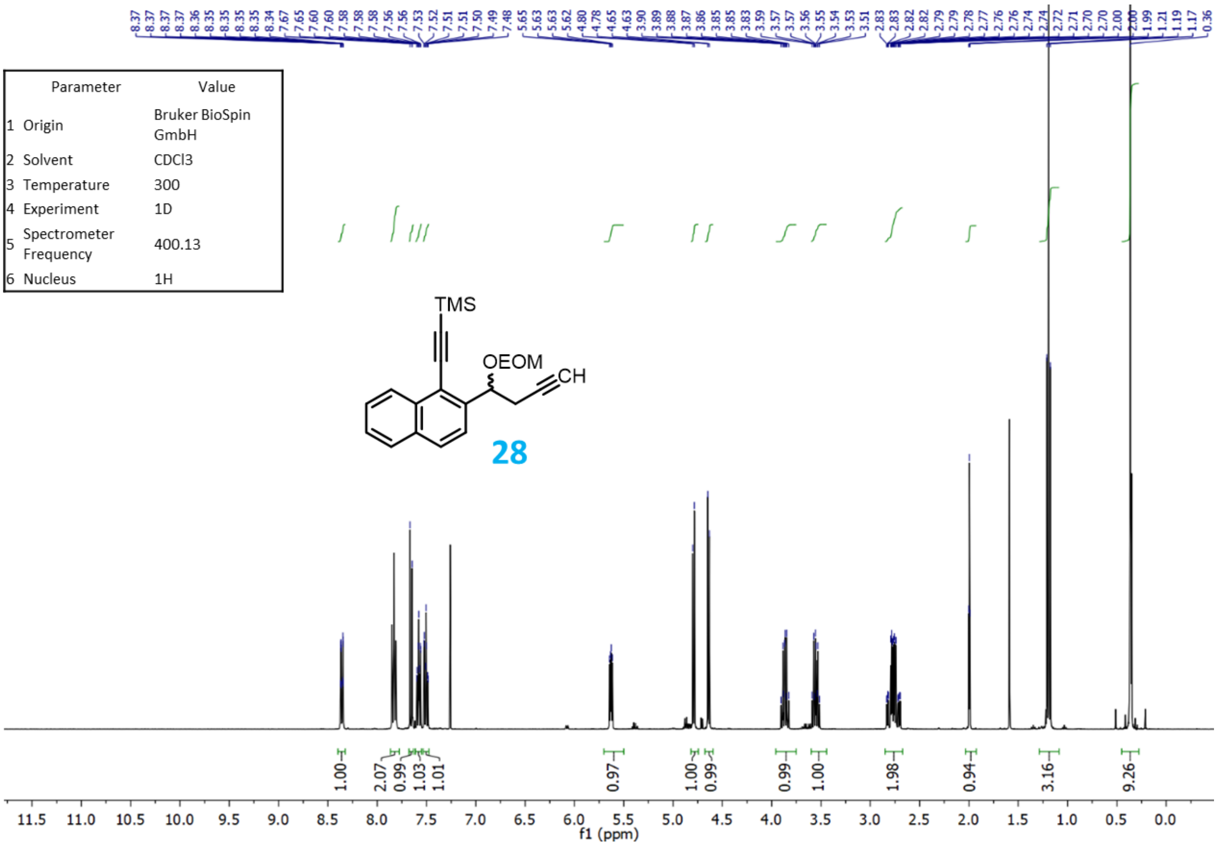

28

	Parameter	Value
1	Origin	Bruker BioSpin GmbH
2	Solvent	CDCl3
3	Temperature	300
4	Experiment	JMOD
5	Spectrometer Frequency	100.62
6	Nucleus	13C

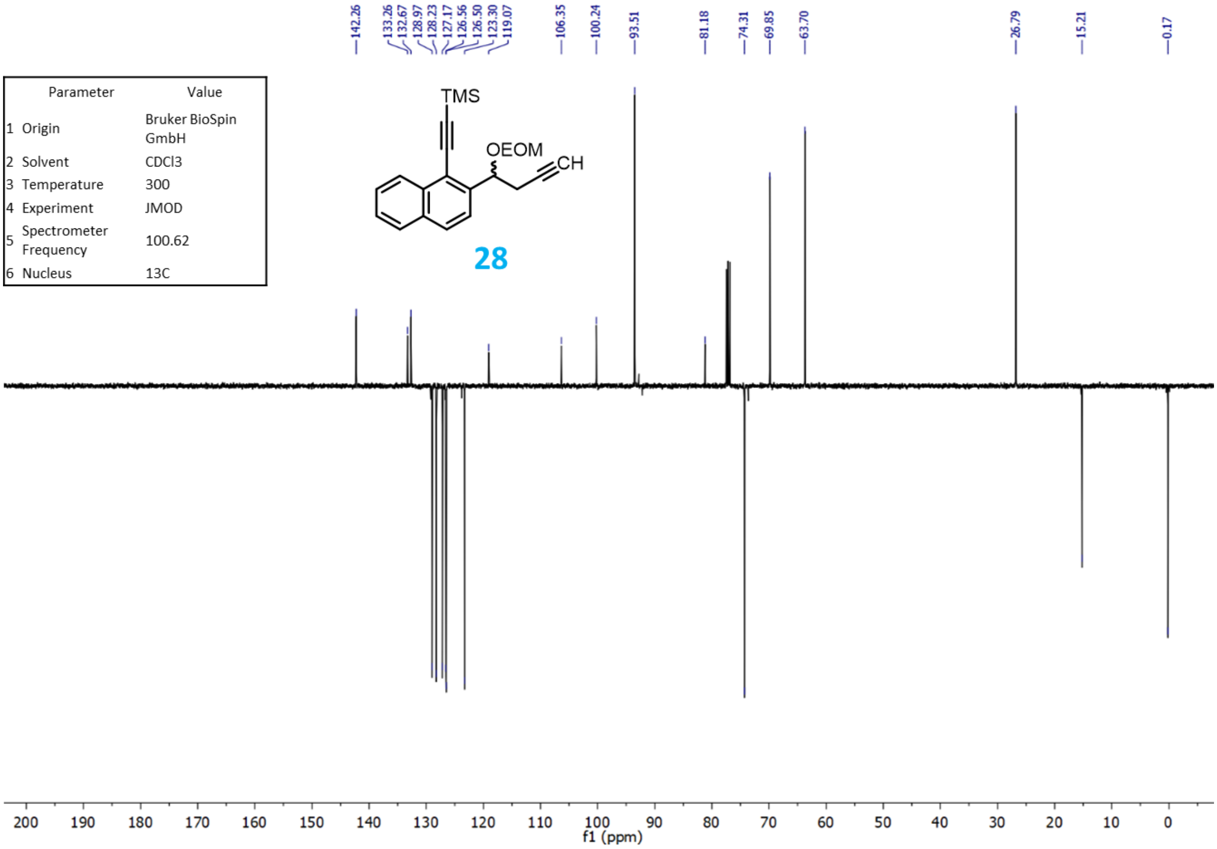

28

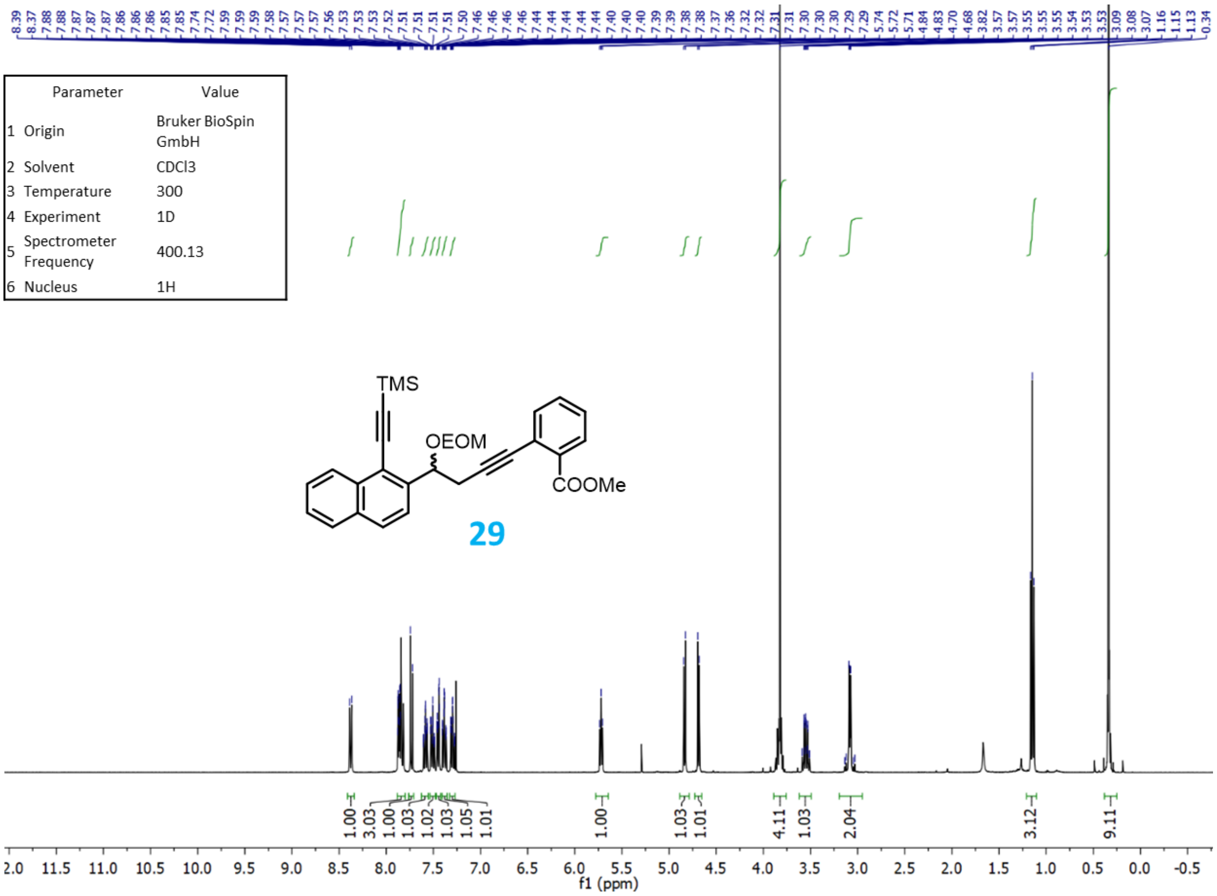
8.39 8.37 7.88 7.88 7.87 7.87 7.86 7.86 7.85 7.85 7.74 7.72 7.59 7.59 7.59 7.58 7.57 7.57 7.56 7.53 7.53 7.52 7.51 7.51 7.51 7.51 7.50 7.46 7.46 7.46 7.44 7.44 7.44 7.44 7.40 7.40 7.40 7.39 7.39 7.38 7.38 7.37 7.36 7.32 7.32 7.31 7.31 7.30 7.30 7.30 7.29 7.29 5.74 5.72 5.71 4.84 4.83 4.70 4.68 3.82 3.57 3.57 3.55 3.55 3.55 3.54 3.53 3.53 3.09 3.08 3.07 1.16 1.15 1.13 0.34
Parameter | Value
1 Origin | Bruker BioSpin GmbH
2 Solvent | CDCl3
3 Temperature | 300
4 Experiment | 1D
5 Spectrometer Frequency | 400.13
6 Nucleus | 1H
TMS
OEOM
COOMe
29
1.00 3.03 1.00 1.03 1.02 1.03 1.05 1.01 1.00 1.03 1.01 4.11 1.03 2.04 3.12 9.11
f1 (ppm)

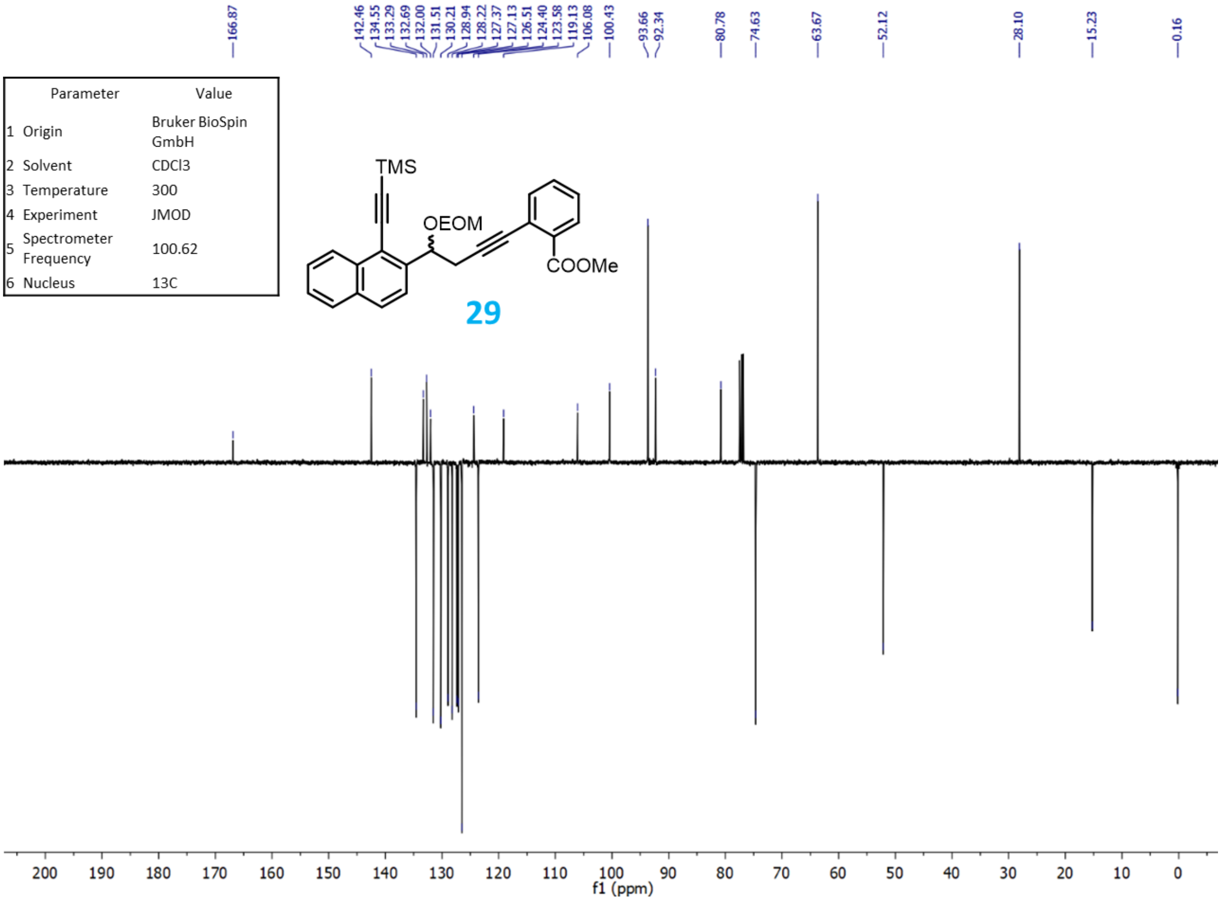
166.87 142.46 134.55 133.29 132.69 132.00 131.51 130.21 128.94 128.22 127.37 127.13 126.51 124.40 123.58 119.13 106.08 100.43 93.66 92.34 80.78 74.63 63.67 52.12 28.10 15.23 0.16
Parameter | Value
1 Origin | Bruker BioSpin GmbH
2 Solvent | CDCl3
3 Temperature | 300
4 Experiment | JMOD
5 Spectrometer Frequency | 100.62
6 Nucleus | 13C
TMS
OEOM
COOMe
29
f1 (ppm)

	Parameter	Value
1	Origin	Bruker BioSpin GmbH
2	Solvent	CDCl3
3	Temperature	300
4	Experiment	1D
5	Spectrometer Frequency	400.13
6	Nucleus	1H

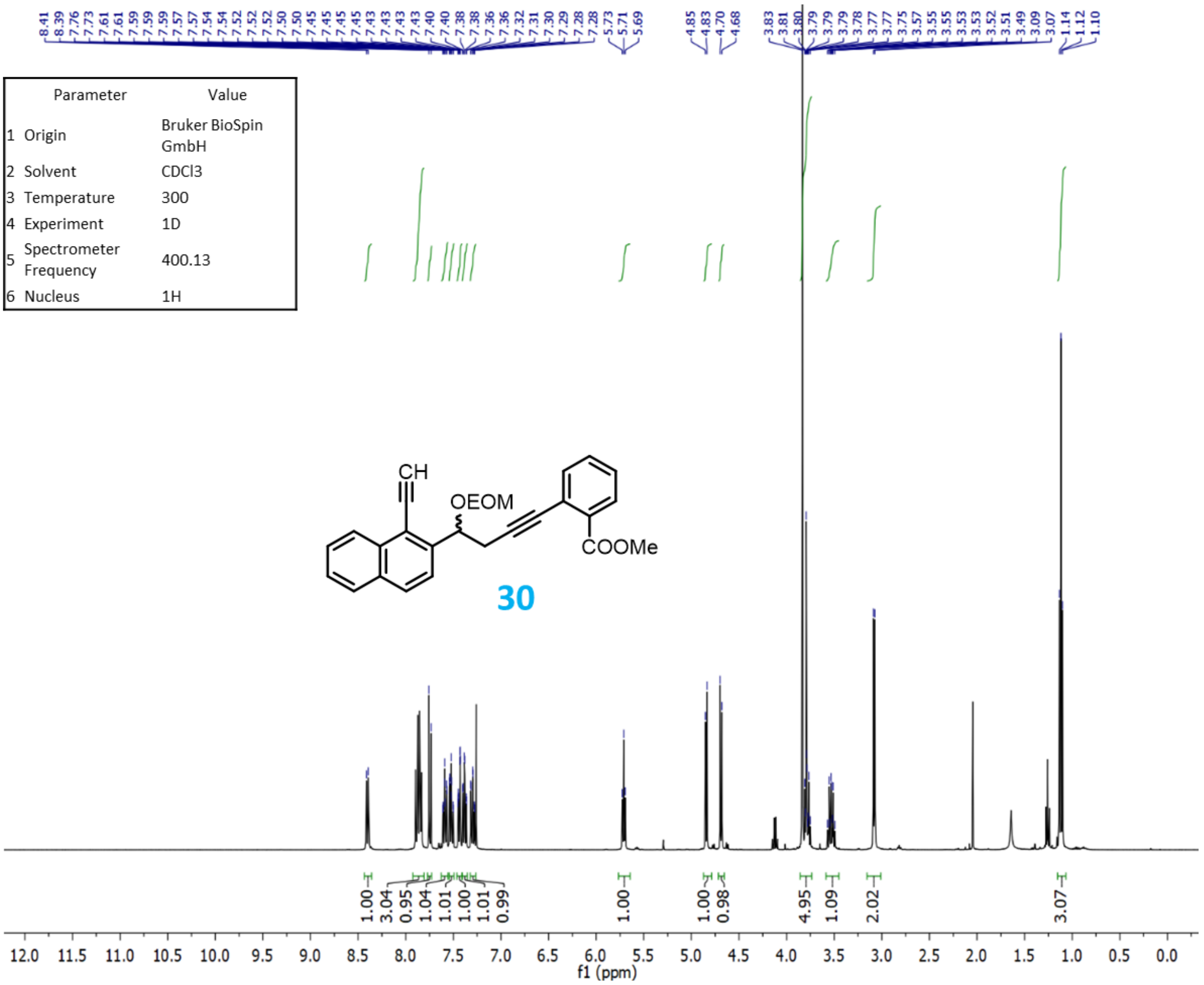

30

	Parameter	Value
1	Origin	Bruker BioSpin GmbH
2	Solvent	CDCl3
3	Temperature	300
4	Experiment	JMOD
5	Spectrometer Frequency	100.62
6	Nucleus	13C

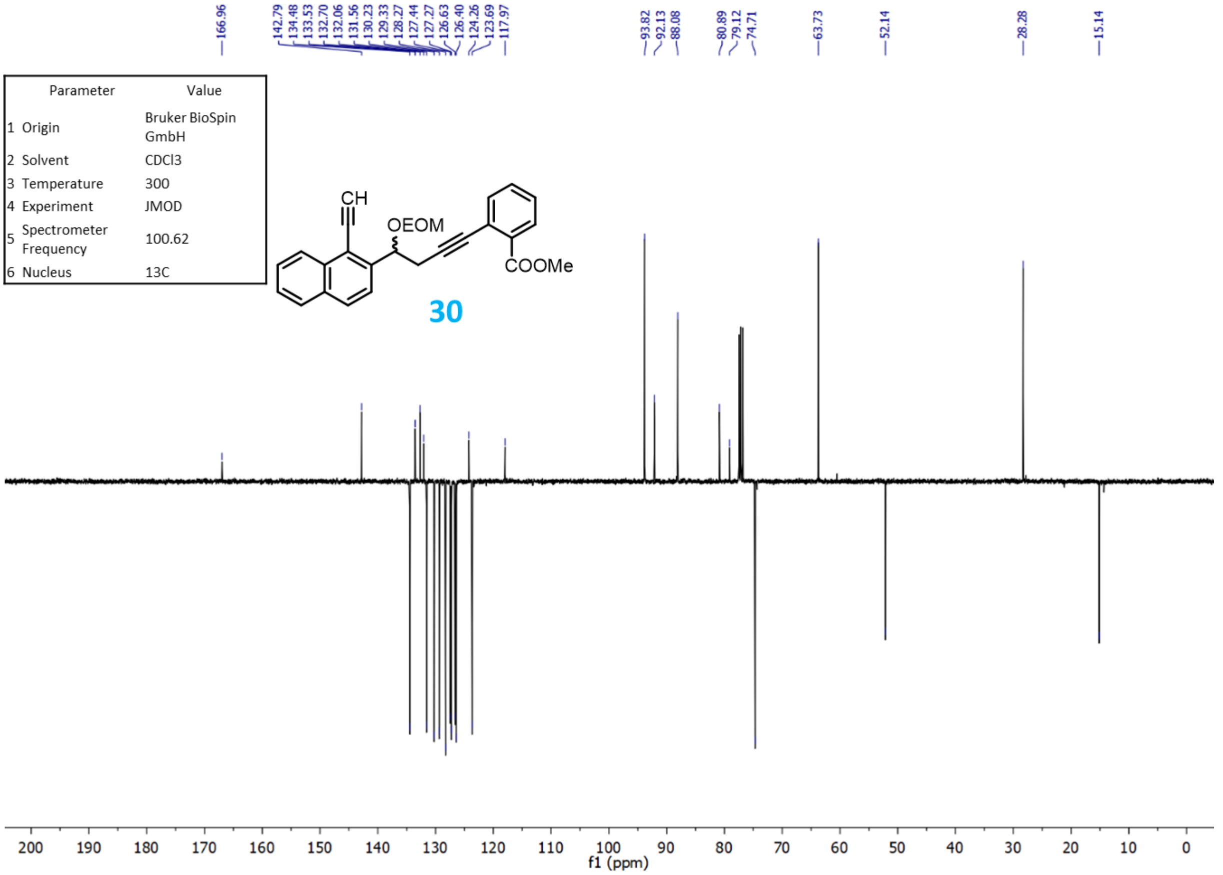

30

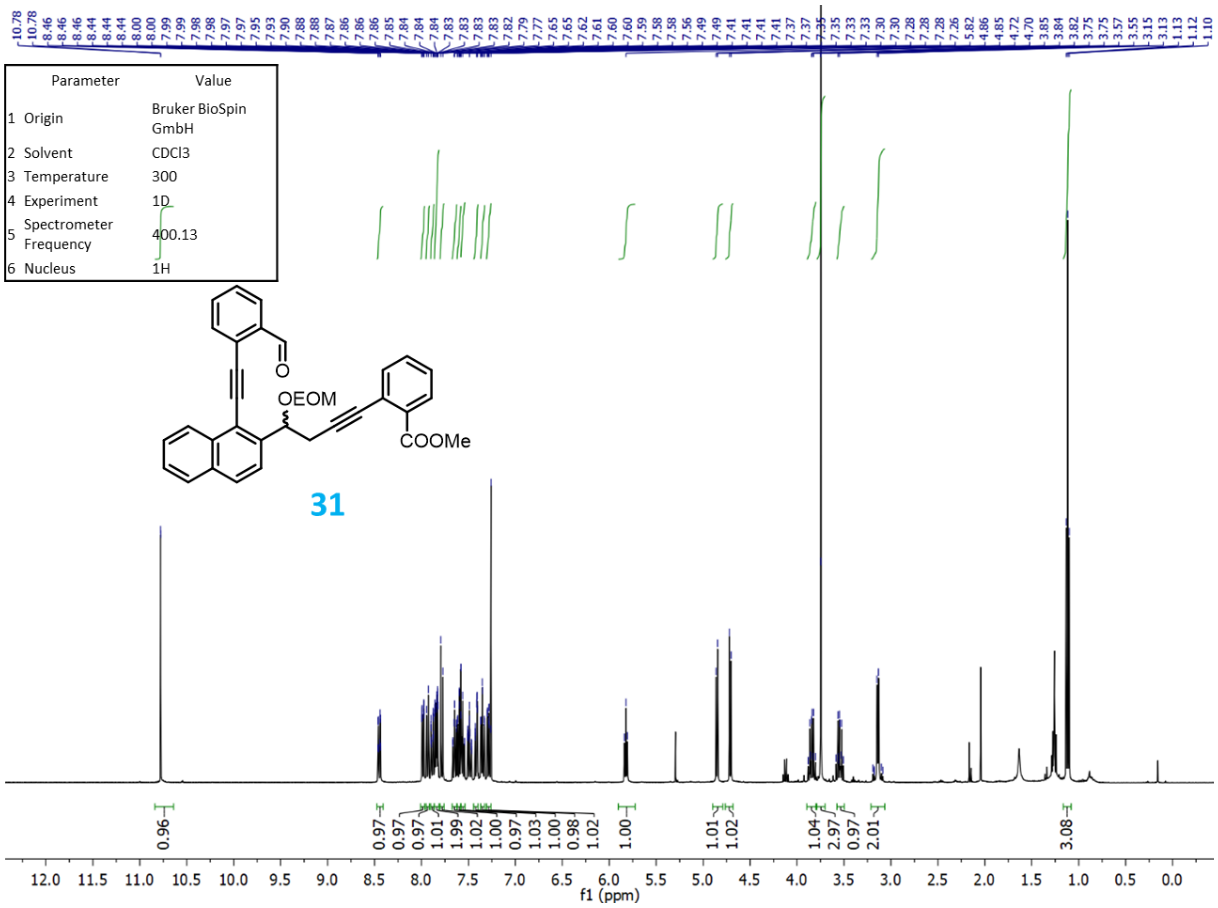
Parameter | Value
1 Origin | Bruker BioSpin GmbH
2 Solvent | CDCl3
3 Temperature | 300
4 Experiment | 1D
5 Spectrometer Frequency | 400.13
6 Nucleus | 1H
OEOM
COOMe
31
f1 (ppm)

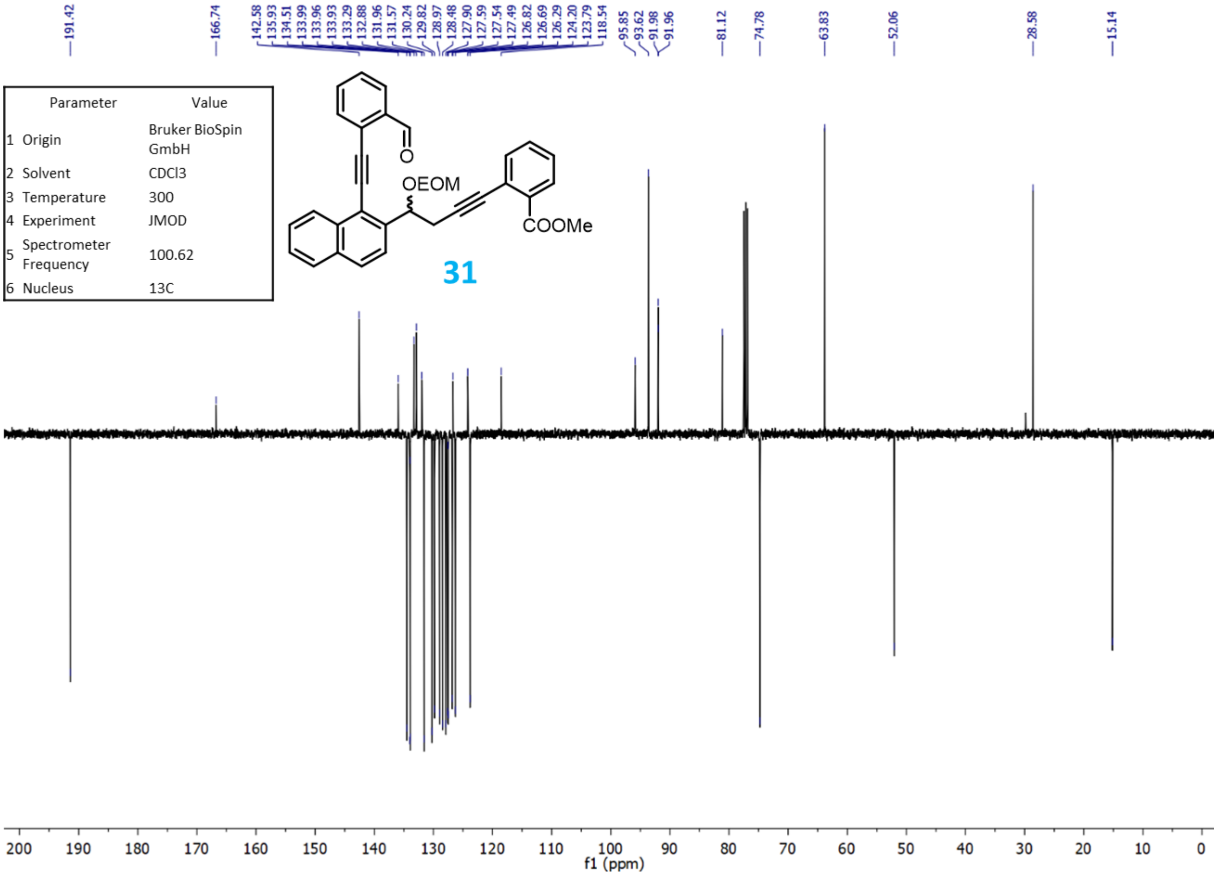
Parameter | Value
1 Origin | Bruker BioSpin GmbH
2 Solvent | CDCl3
3 Temperature | 300
4 Experiment | JMOD
5 Spectrometer Frequency | 100.62
6 Nucleus | 13C
OEOM
COOMe
31
f1 (ppm)

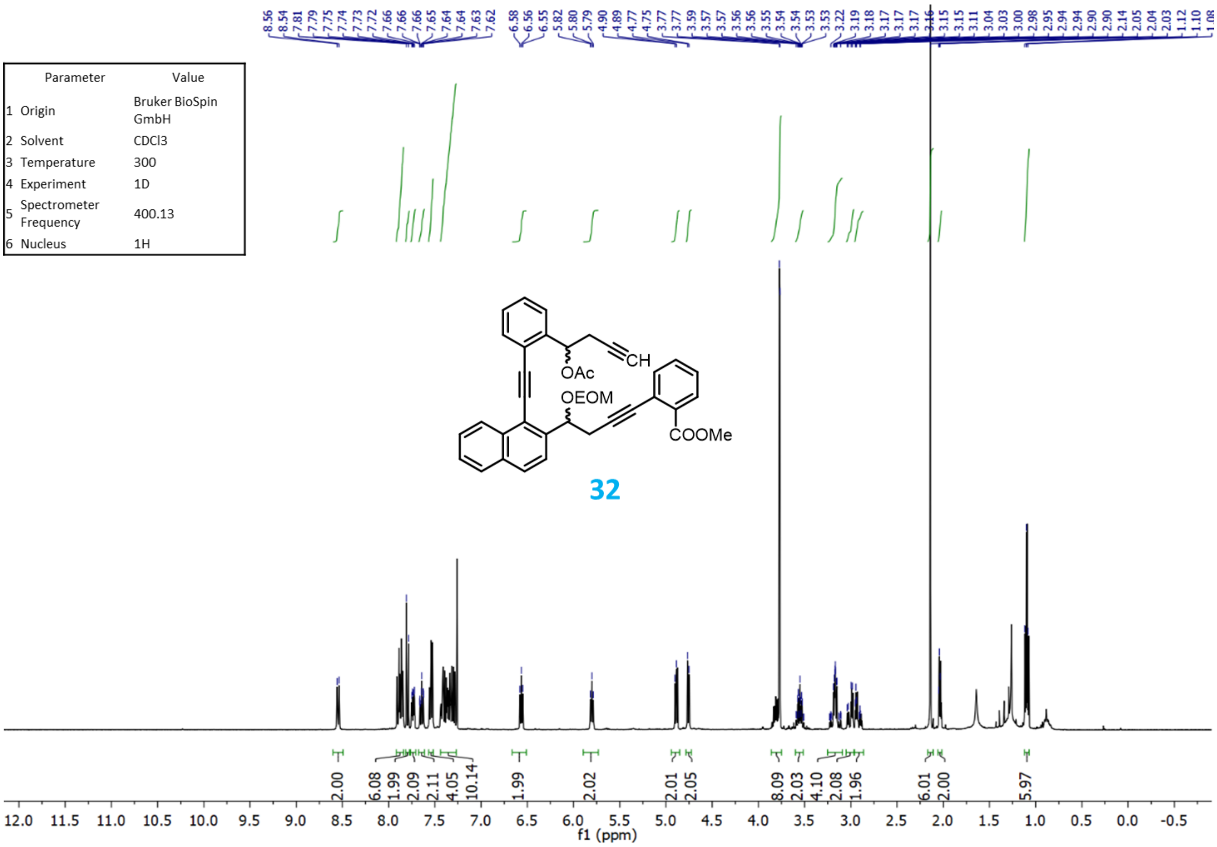

	Parameter	Value
1	Origin	Bruker BioSpin GmbH
2	Solvent	CDCl3
3	Temperature	300
4	Experiment	1D
5	Spectrometer Frequency	400.13
6	Nucleus	1H

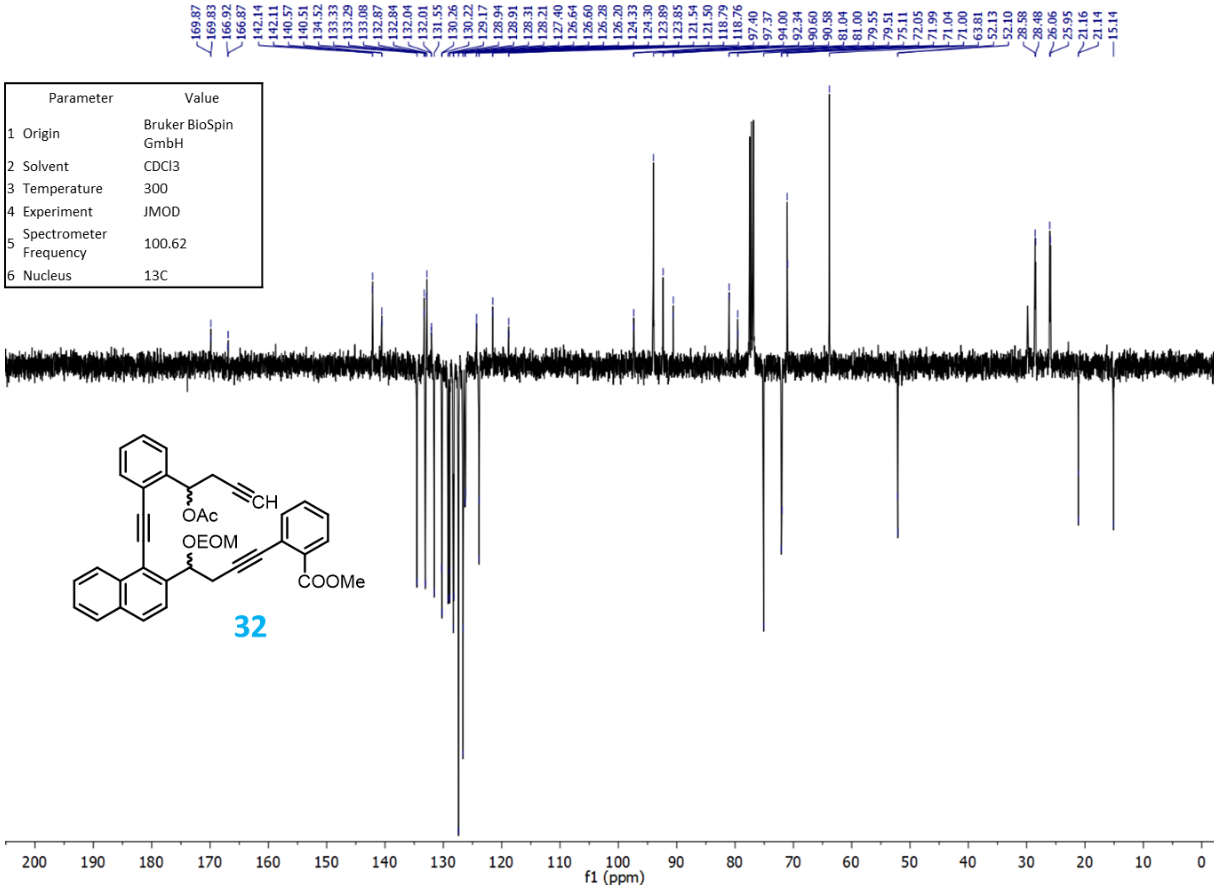

	Parameter	Value
1	Origin	Bruker BioSpin GmbH
2	Solvent	CDCl3
3	Temperature	300
4	Experiment	JMOD
5	Spectrometer Frequency	100.62
6	Nucleus	13C

	Parameter	Value
1	Origin	Bruker BioSpin GmbH
2	Solvent	CDCl3
3	Temperature	300
4	Experiment	1D
5	Spectrometer Frequency	400.13
6	Nucleus	1H

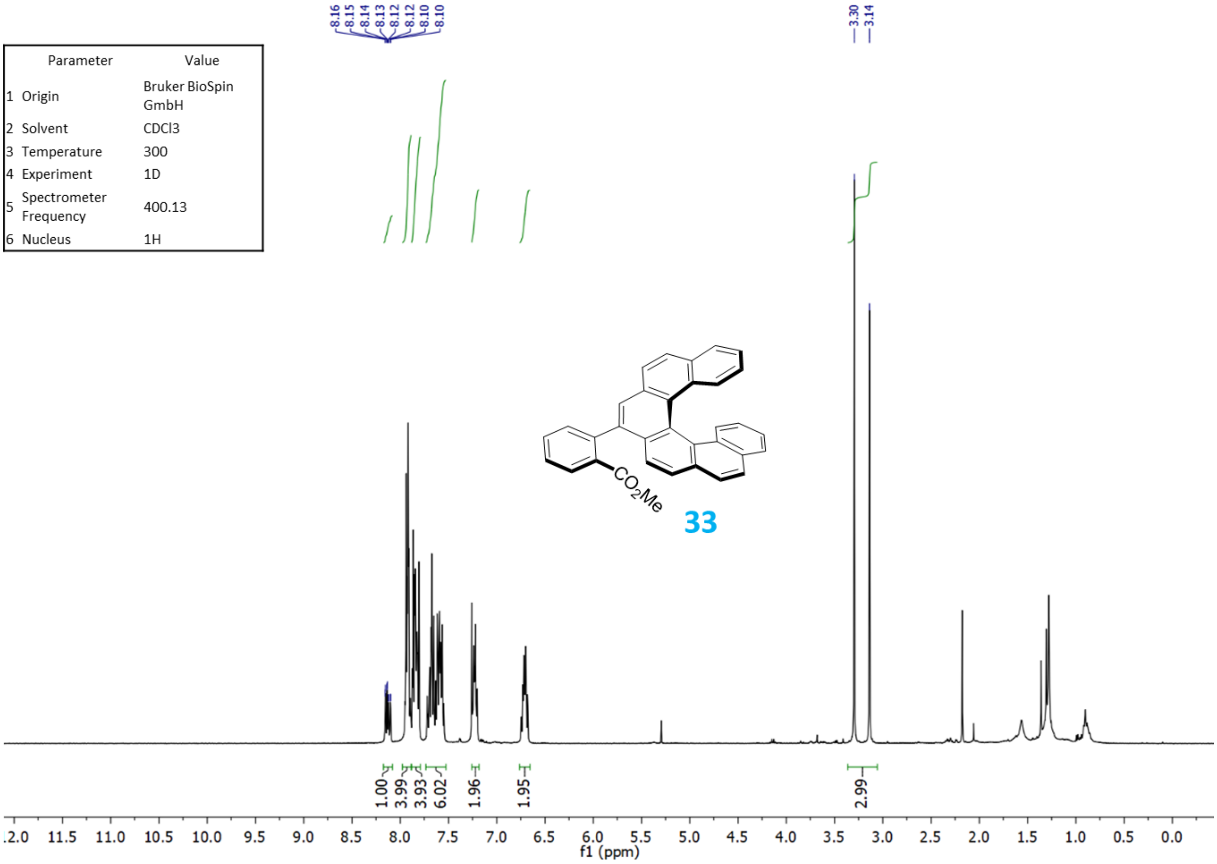

	Parameter	Value
1	Origin	Bruker BioSpin GmbH
2	Solvent	CDCl3
3	Temperature	300
4	Experiment	JMOD
5	Spectrometer Frequency	100.62
6	Nucleus	13C

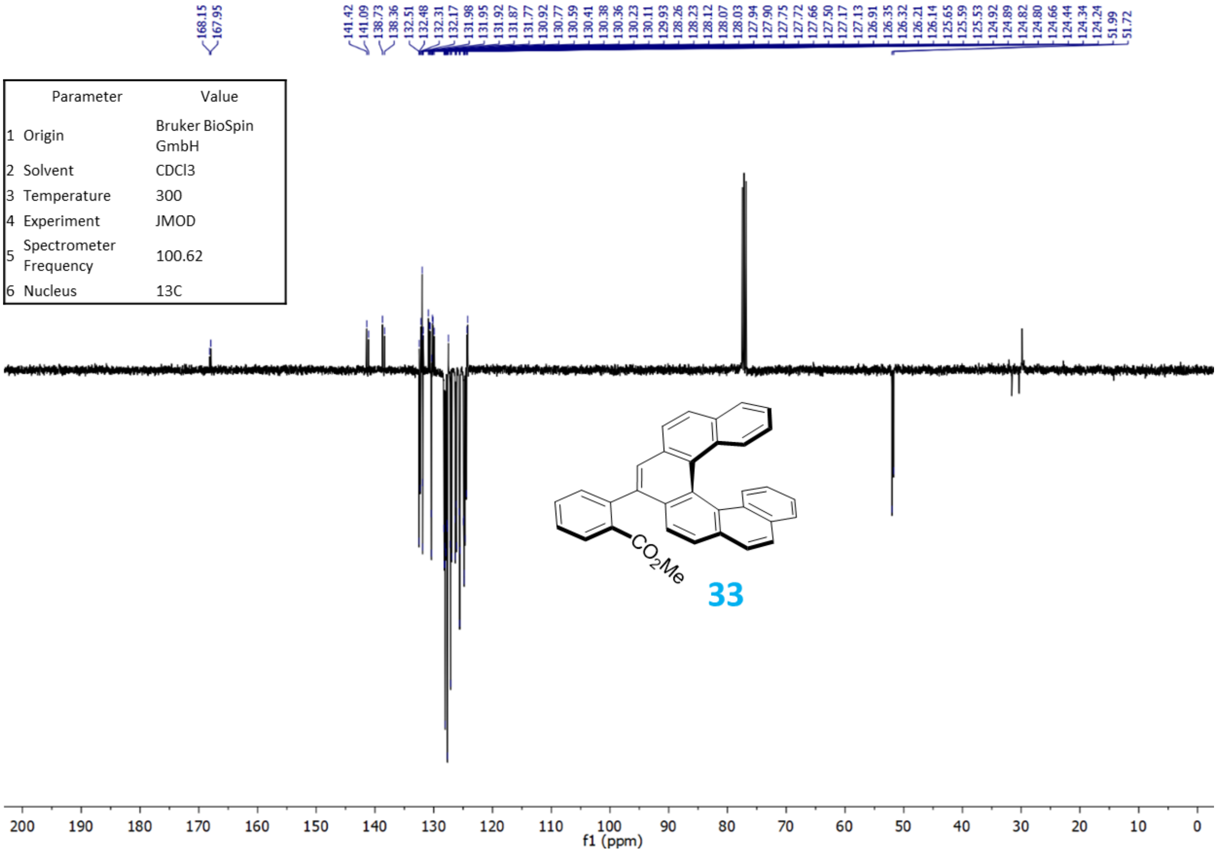

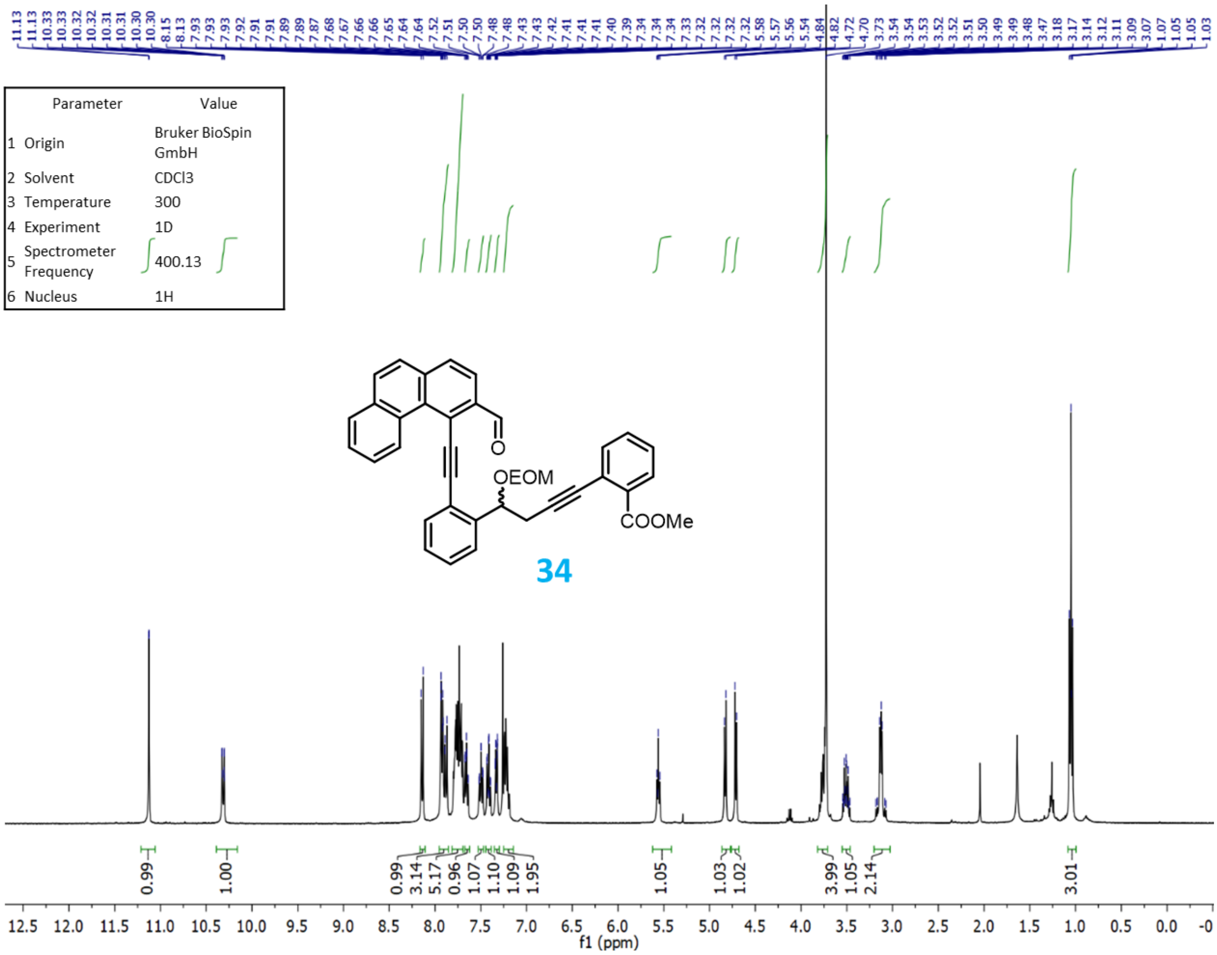

Parameter	Value
1 Origin	Bruker BioSpin GmbH
2 Solvent	CDCl3
3 Temperature	300
4 Experiment	1D
5 Spectrometer Frequency	400.13
6 Nucleus	1H

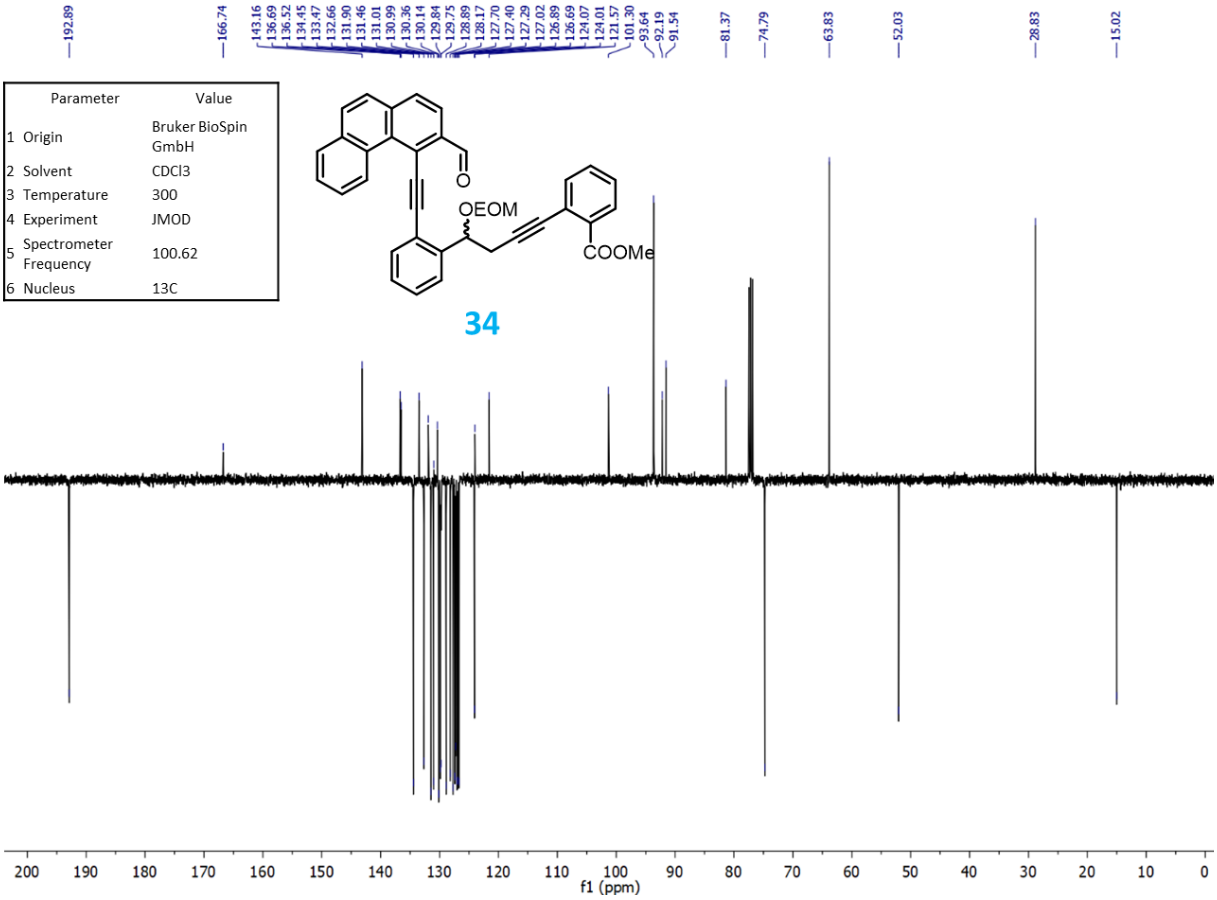

Parameter	Value
1 Origin	Bruker BioSpin GmbH
2 Solvent	CDCl3
3 Temperature	300
4 Experiment	JMOD
5 Spectrometer Frequency	100.62
6 Nucleus	13C

	Parameter	Value
1	Origin	Bruker BioSpin GmbH
2	Solvent	CDCl3
3	Temperature	300
4	Experiment	1D
5	Spectrometer Frequency	400.13
6	Nucleus	1H

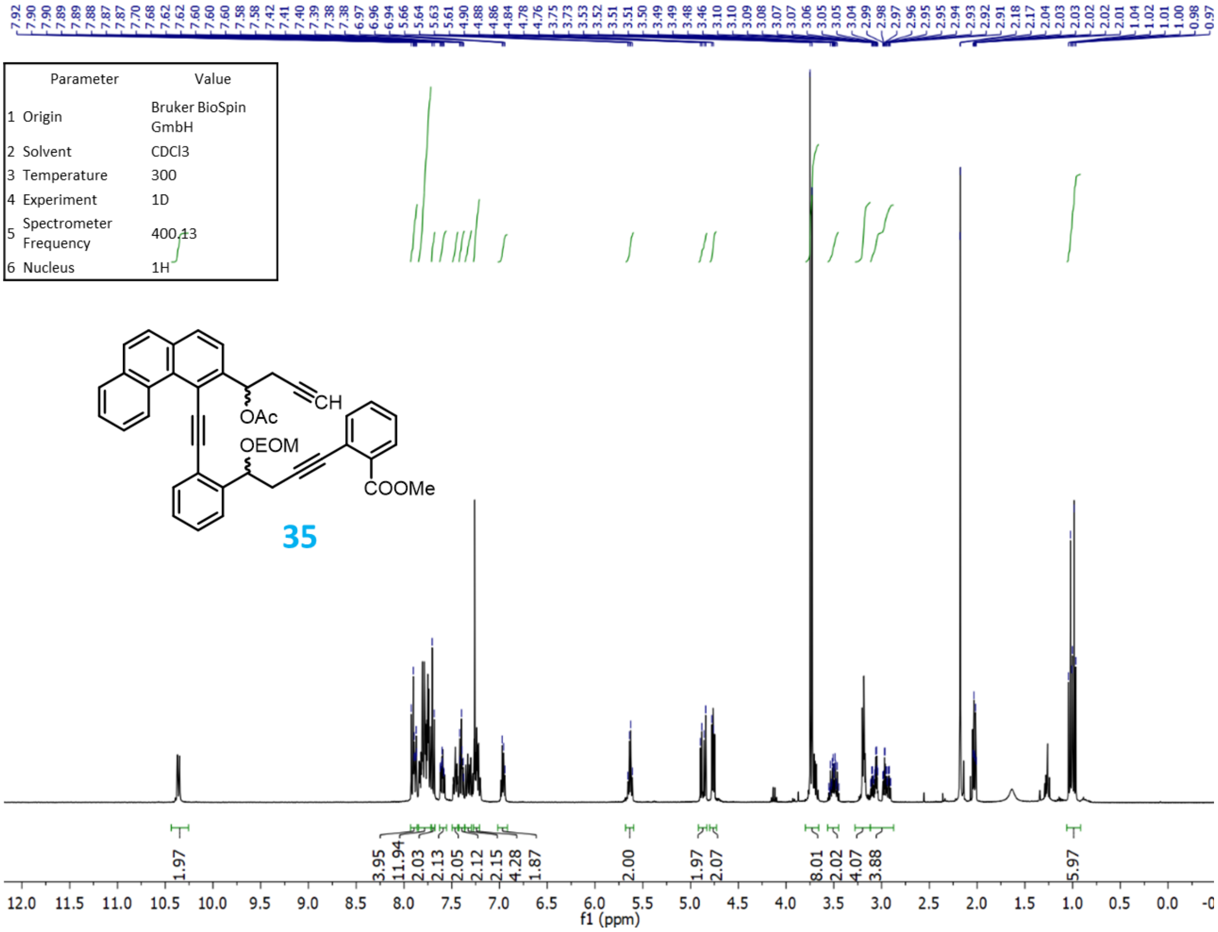

	Parameter	Value
1	Origin	Bruker BioSpin GmbH
2	Solvent	CDCl3
3	Temperature	300
4	Experiment	JMOD
5	Spectrometer Frequency	100.62
6	Nucleus	13C

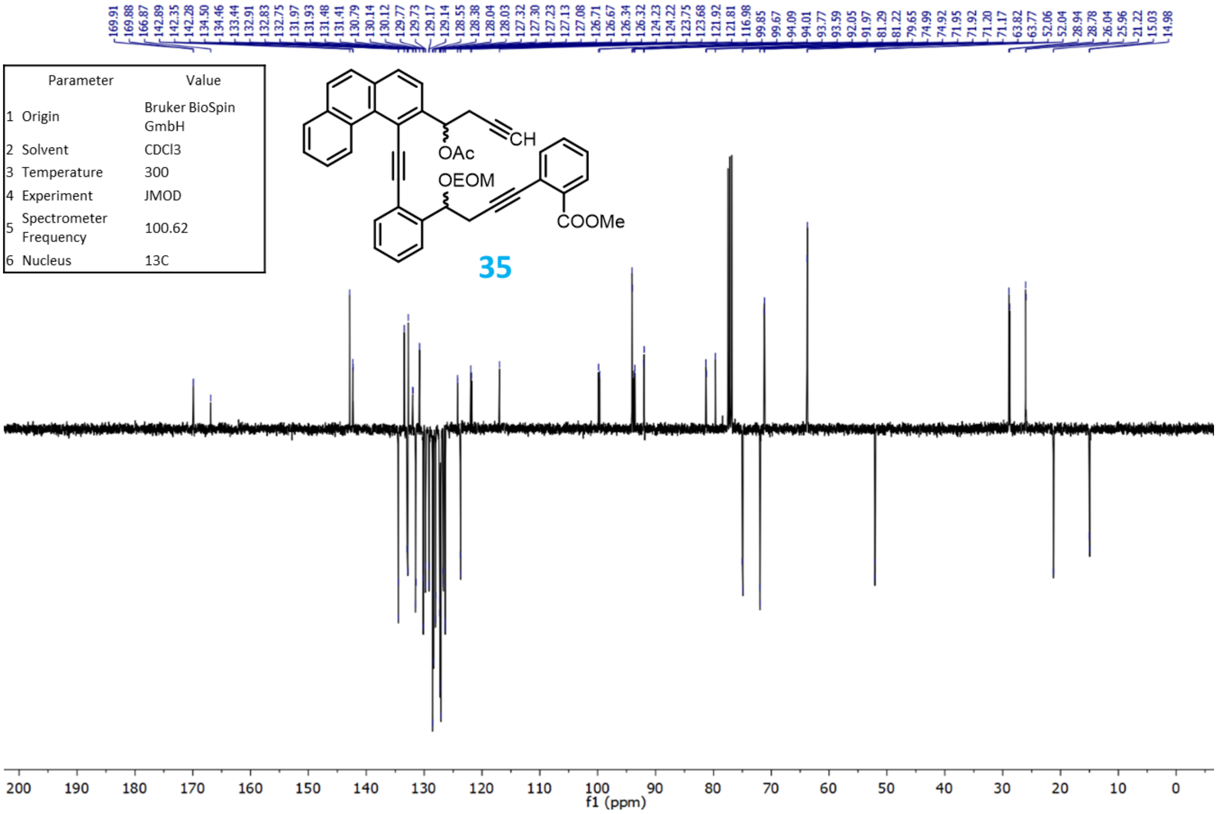

	Parameter	Value
1	Origin	Bruker BioSpin GmbH
2	Solvent	CDCl3
3	Temperature	300
4	Experiment	1D
5	Spectrometer Frequency	400.13
6	Nucleus	1H

36

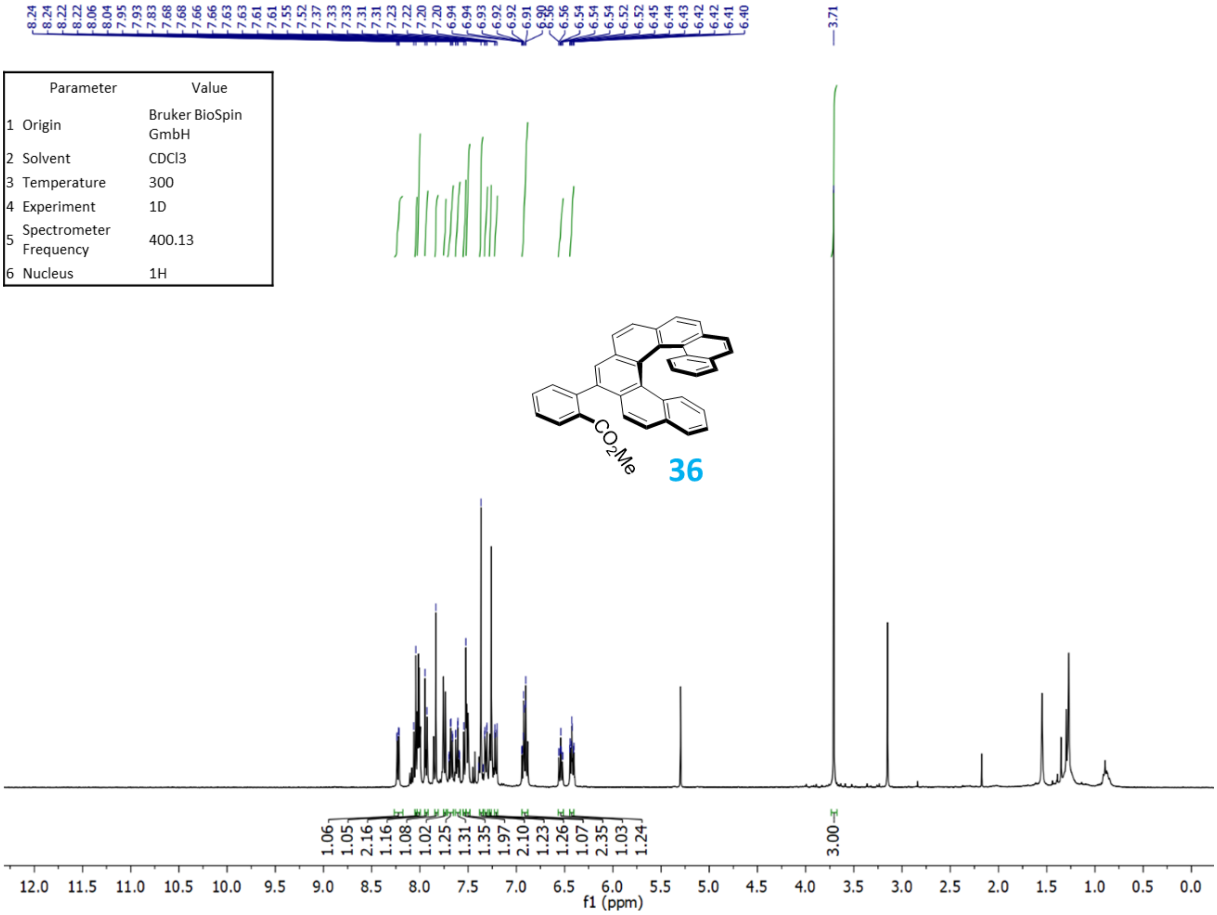

	Parameter	Value
1	Origin	Bruker BioSpin GmbH
2	Solvent	CDCl3
3	Temperature	300
4	Experiment	JMOD
5	Spectrometer Frequency	100.62
6	Nucleus	13C

36

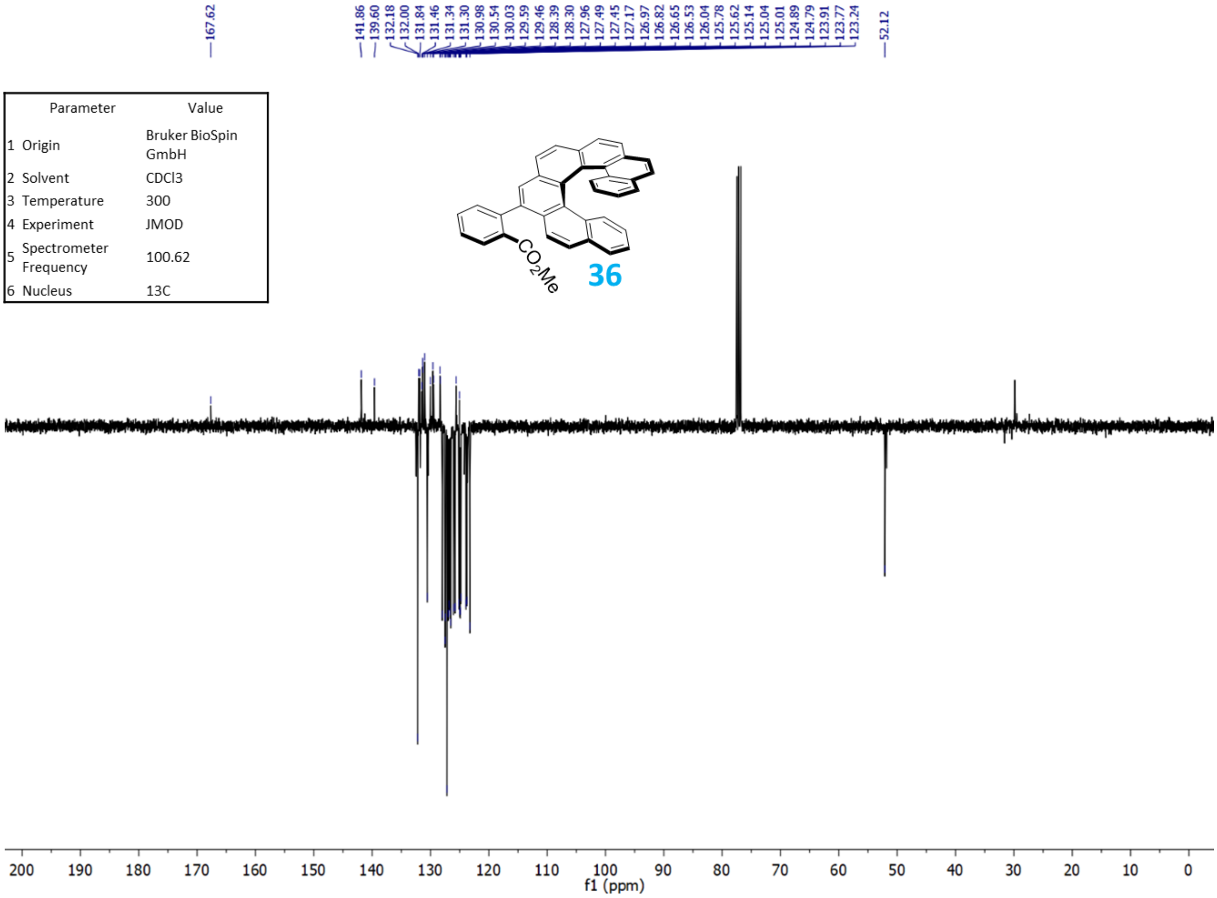

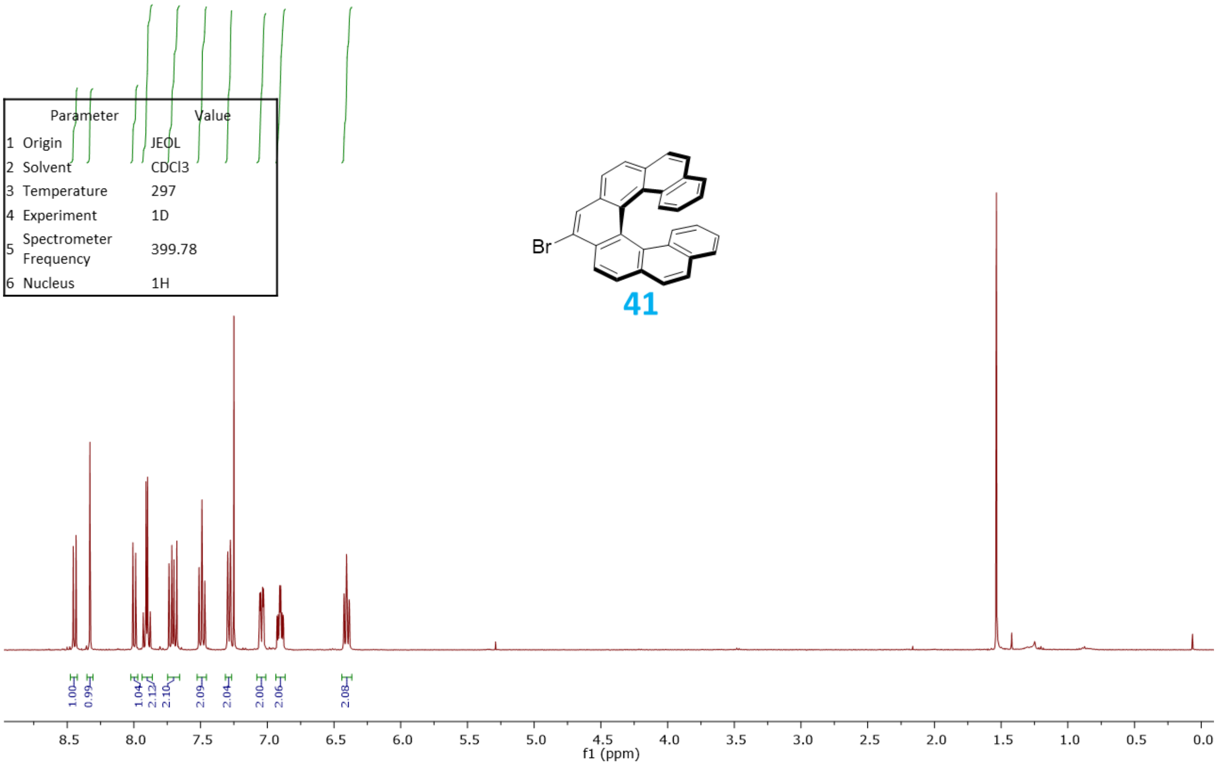

	Parameter	Value
1	Origin	JEOL
2	Solvent	CDCl3
3	Temperature	297
4	Experiment	1D
5	Spectrometer Frequency	399.78
6	Nucleus	1H

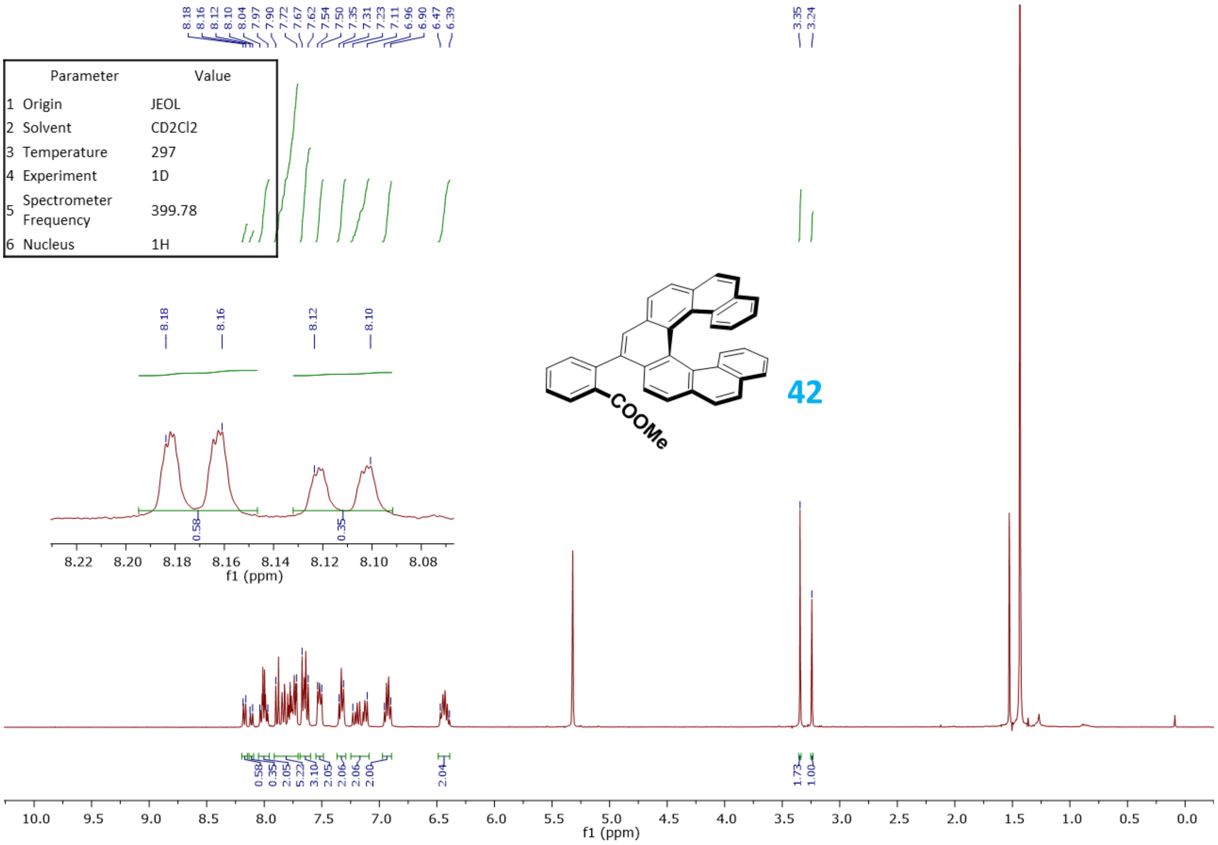

	Parameter	Value
1	Origin	JEOL
2	Solvent	CD2Cl2
3	Temperature	297
4	Experiment	1D
5	Spectrometer Frequency	399.78
6	Nucleus	1H

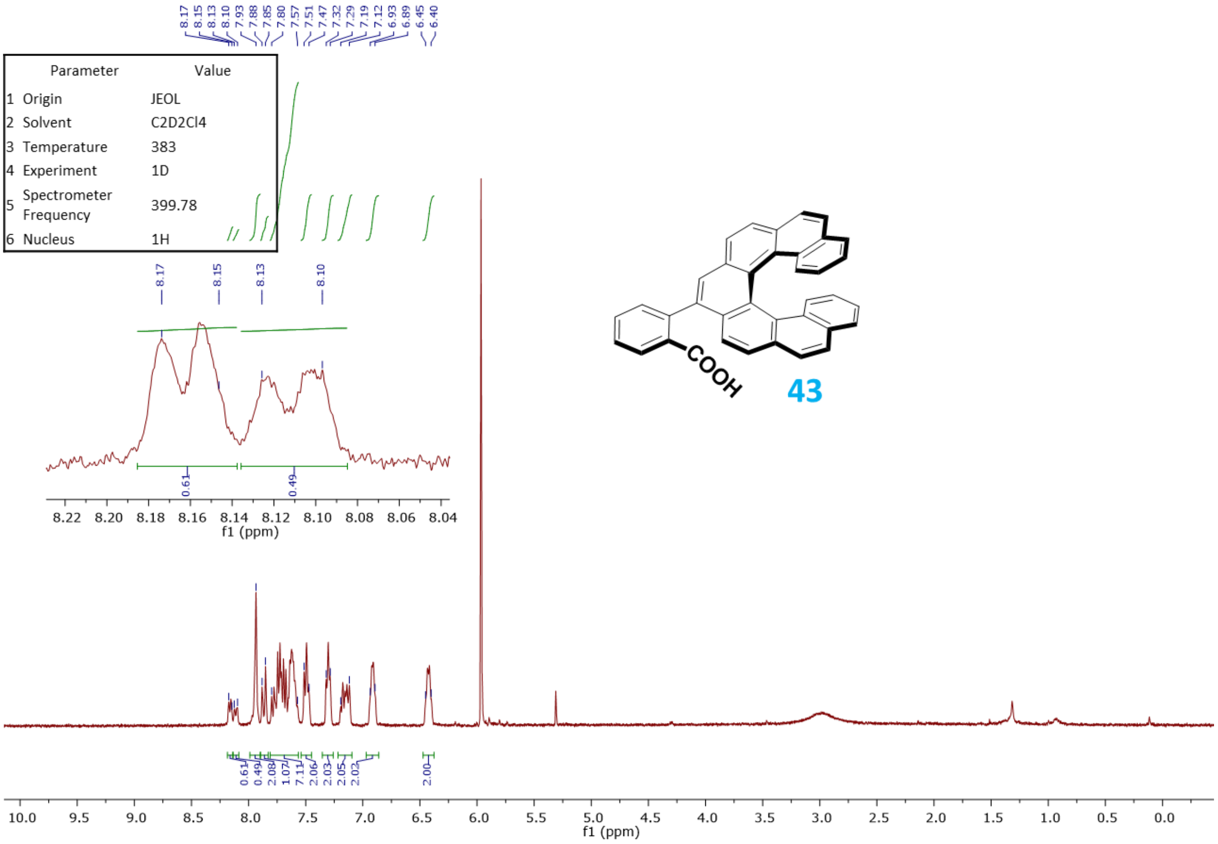

	Parameter	Value
1	Origin	JEOL
2	Solvent	C2D2Cl4
3	Temperature	383
4	Experiment	1D
5	Spectrometer Frequency	399.78
6	Nucleus	1H

43

AJ260
single_pulse

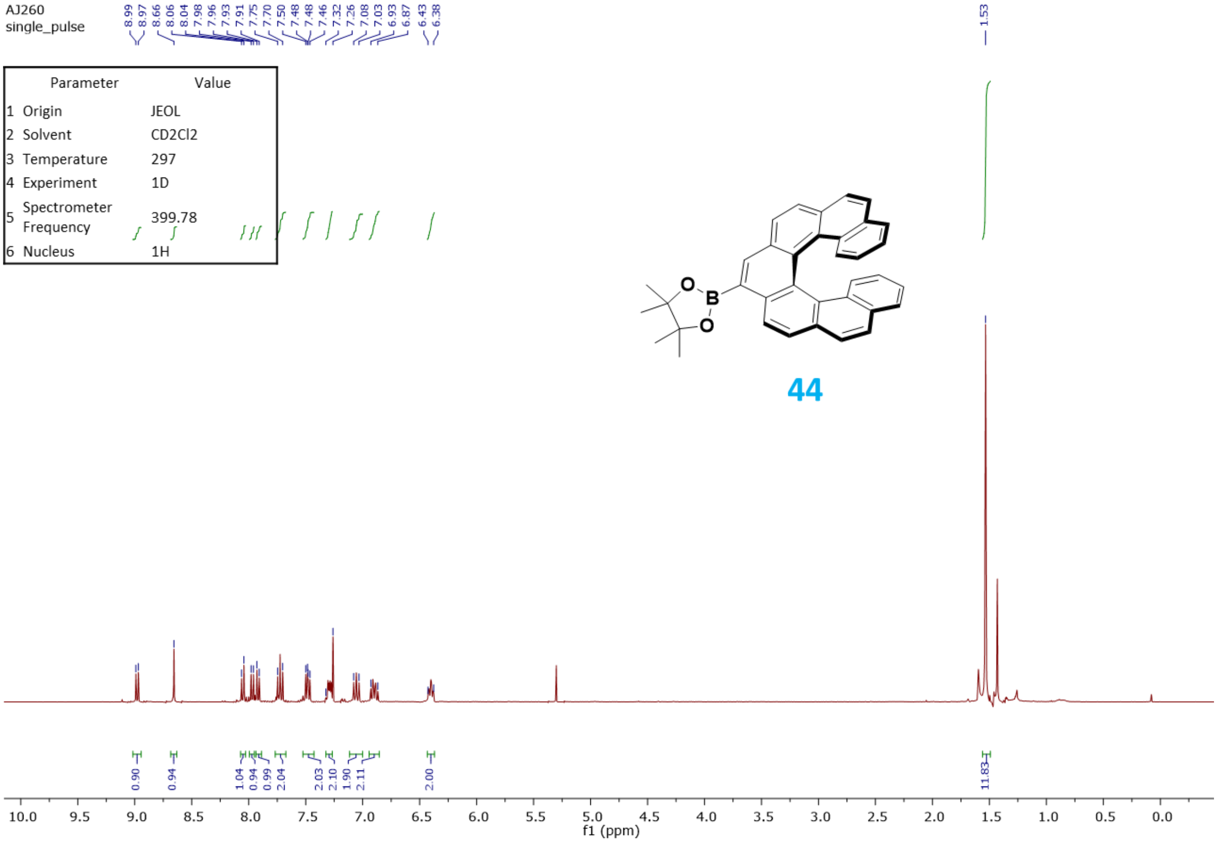

	Parameter	Value
1	Origin	JEOL
2	Solvent	CD2Cl2
3	Temperature	297
4	Experiment	1D
5	Spectrometer Frequency	399.78
6	Nucleus	1H

44

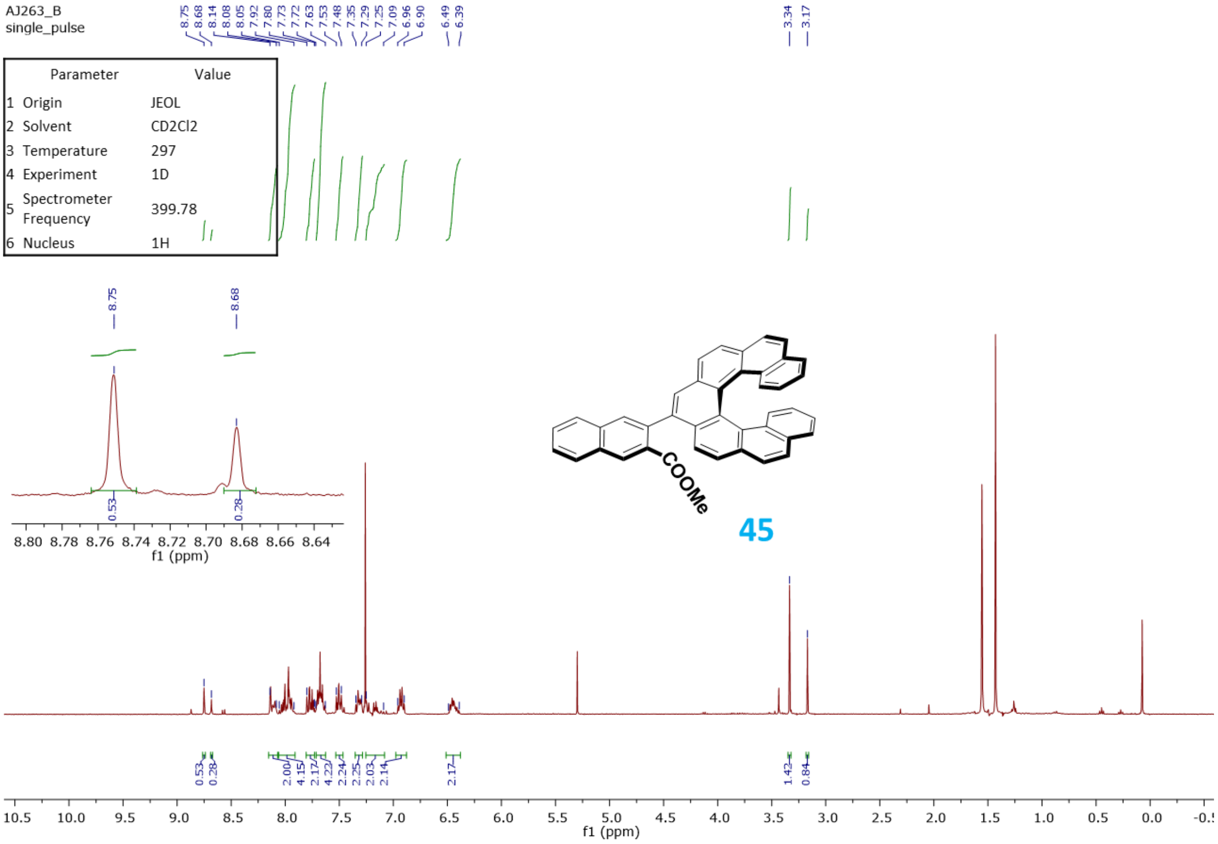
AJ263_B
single_pulse
Parameter Value
1 Origin JEOL
2 Solvent CD2Cl2
3 Temperature 297
4 Experiment 1D
5 Spectrometer Frequency 399.78
6 Nucleus 1H
COOMe
45
f1 (ppm)

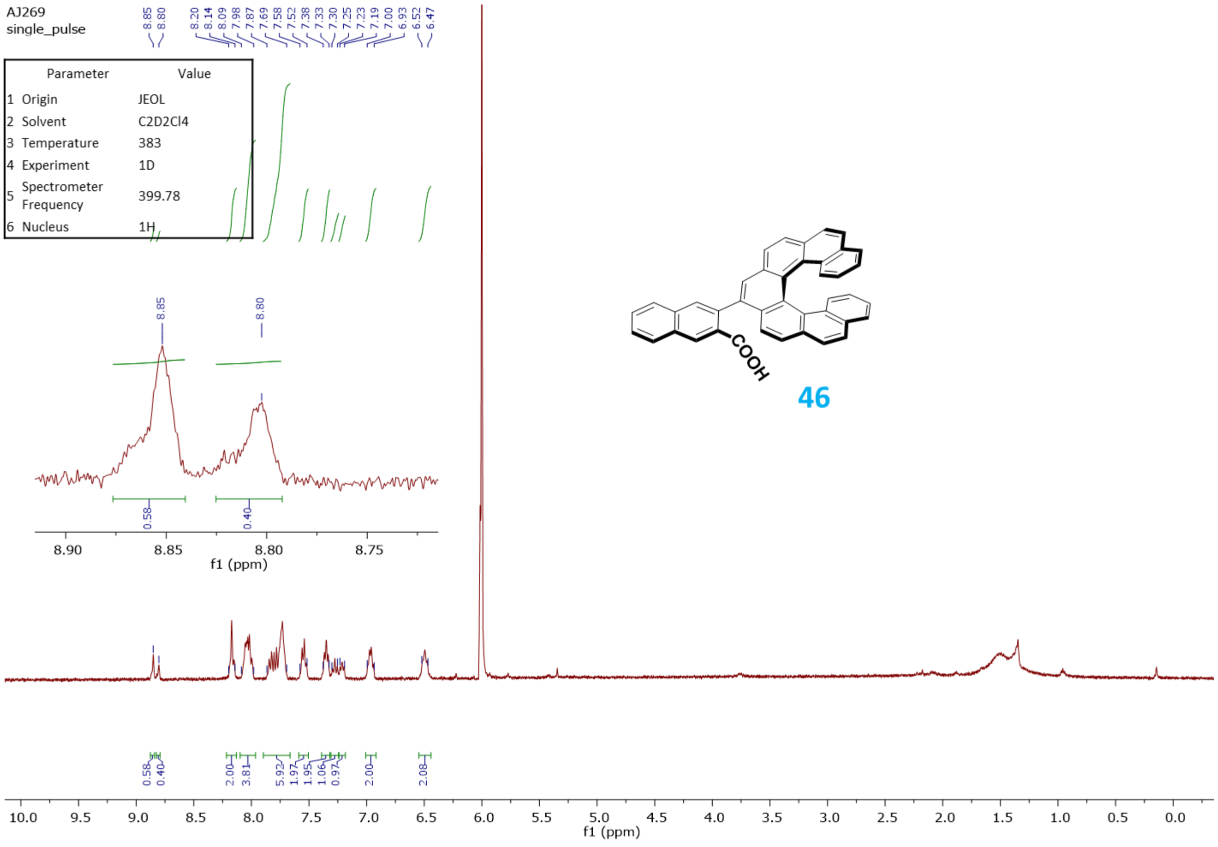
AJ269
single_pulse
Parameter Value
1 Origin JEOL
2 Solvent C2D2Cl4
3 Temperature 383
4 Experiment 1D
5 Spectrometer Frequency 399.78
6 Nucleus 1H
COOH
46
f1 (ppm)

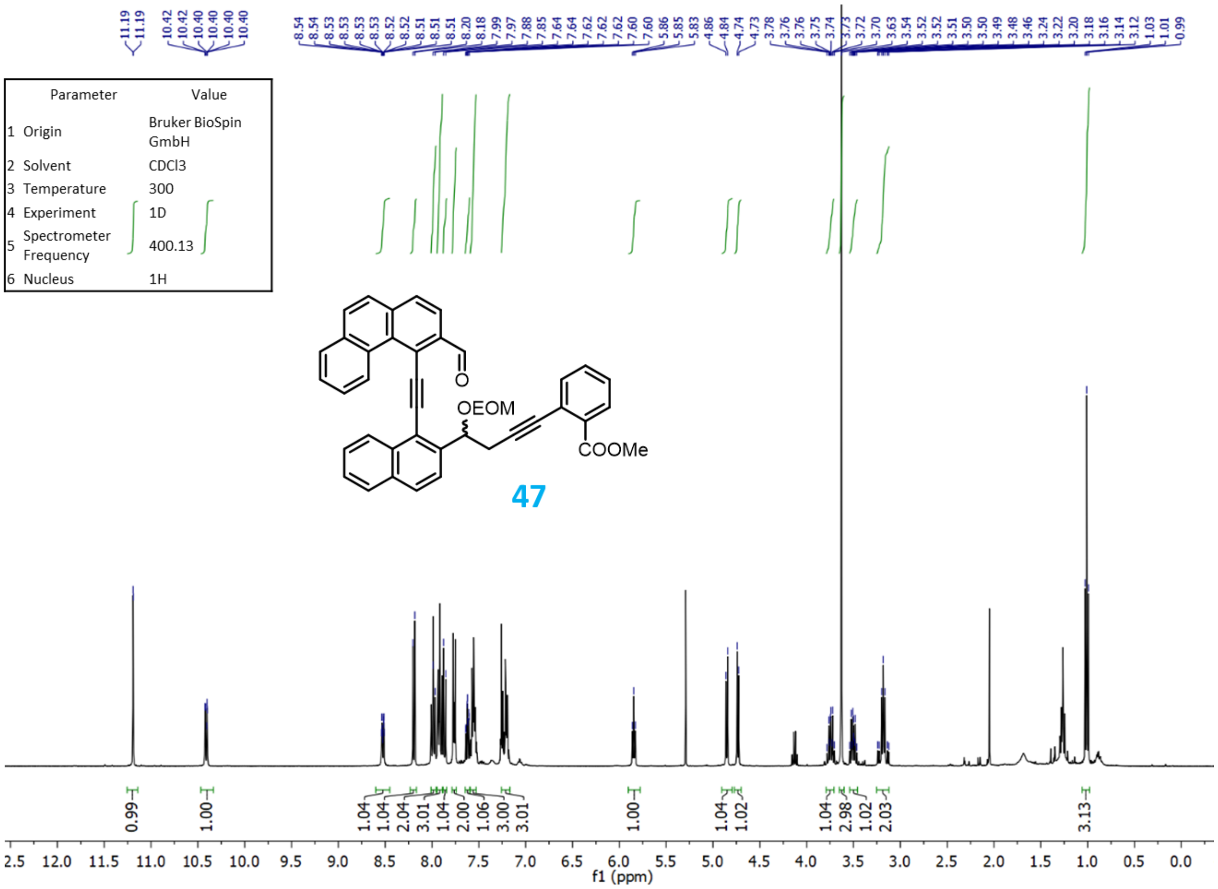
Parameter Value
1 Origin Bruker BioSpin GmbH
2 Solvent CDCl3
3 Temperature 300
4 Experiment 1D
5 Spectrometer Frequency 400.13
6 Nucleus 1H
OEOM
O
COOMe
47
f1 (ppm)

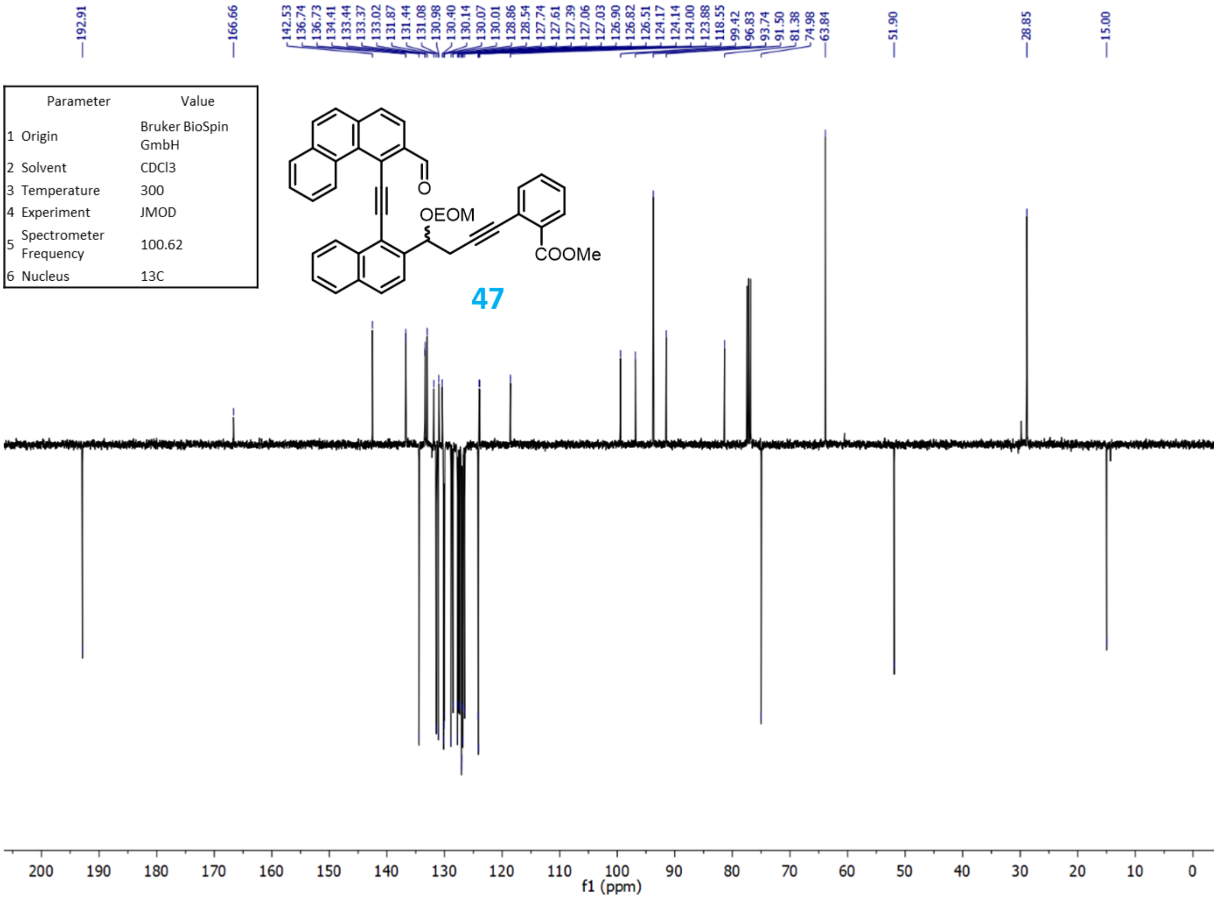
Parameter Value
1 Origin Bruker BioSpin GmbH
2 Solvent CDCl3
3 Temperature 300
4 Experiment JMOD
5 Spectrometer Frequency 100.62
6 Nucleus 13C
OEOM
O
COOMe
47
f1 (ppm)

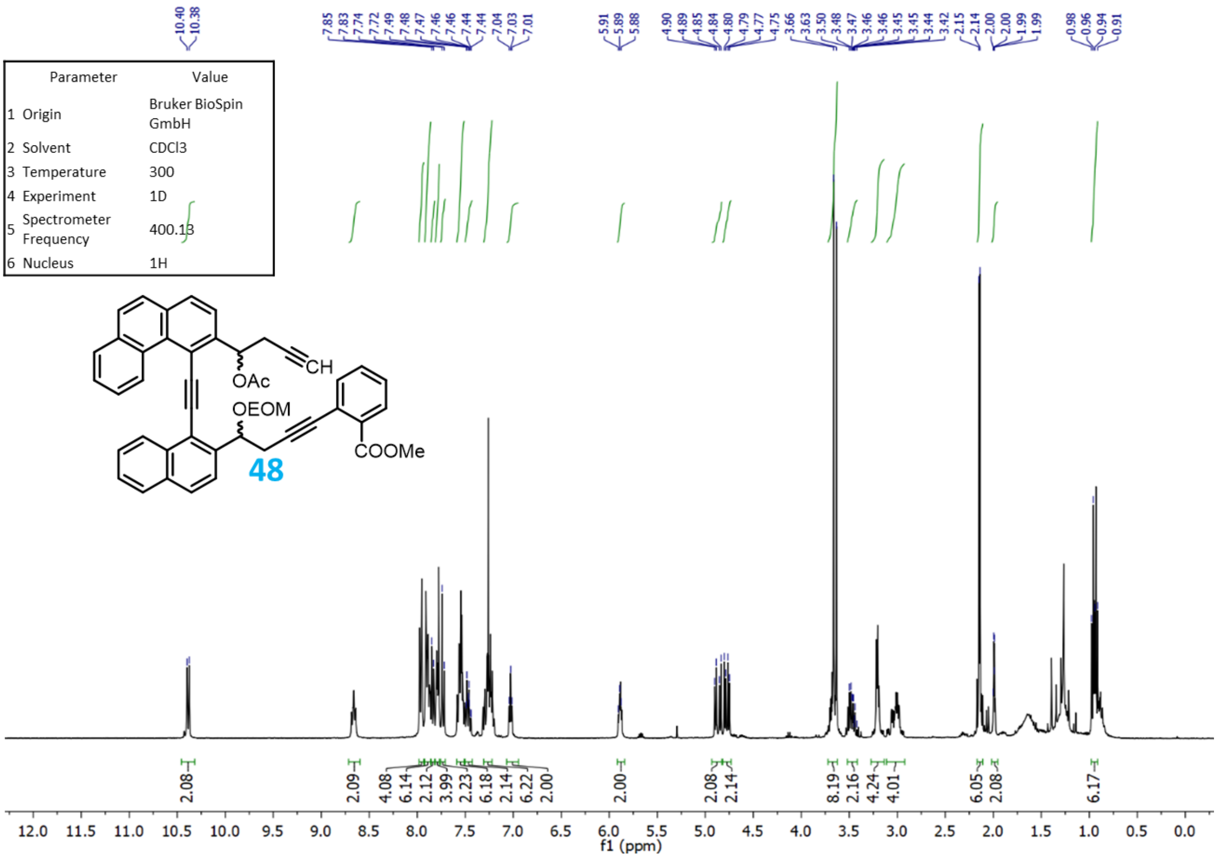
Parameter Value
1 Origin Bruker BioSpin GmbH
2 Solvent CDCl3
3 Temperature 300
4 Experiment 1D
5 Spectrometer Frequency 400.13
6 Nucleus 1H
OAc
CH
OEOM
COOMe
48
f1 (ppm)

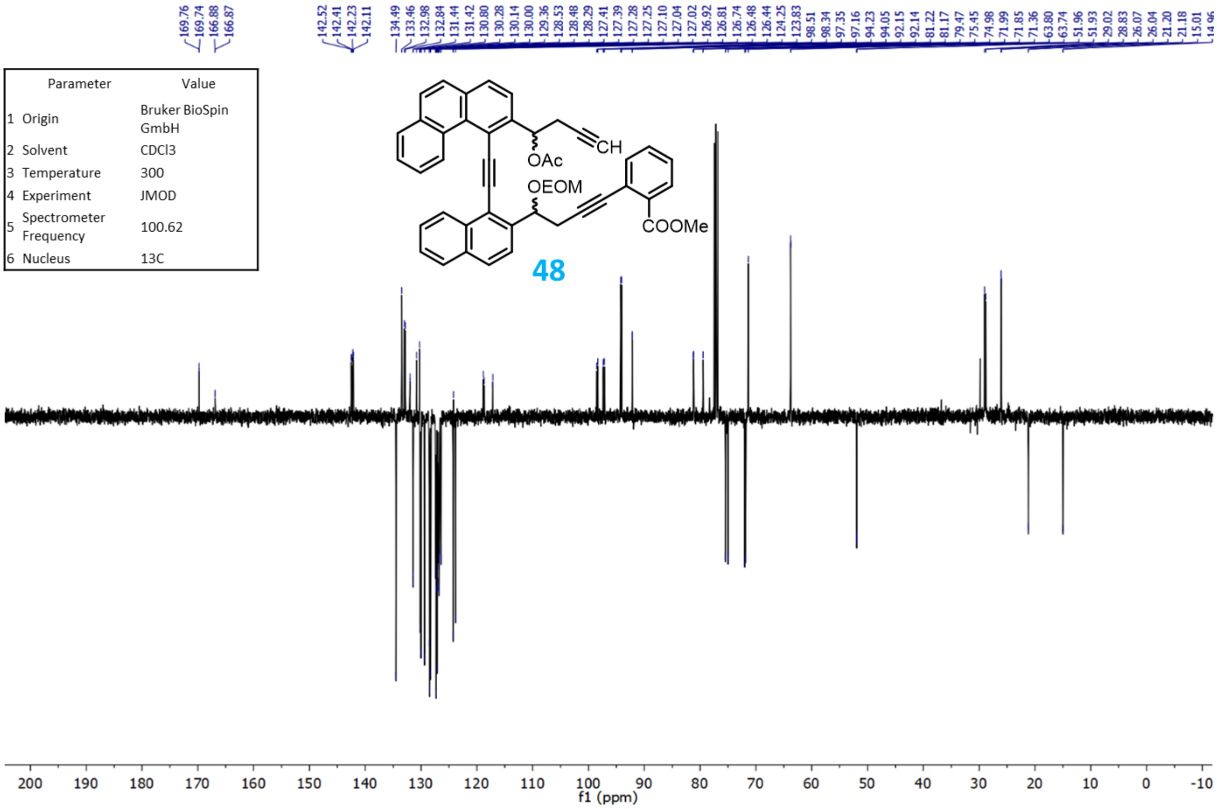
Parameter Value
1 Origin Bruker BioSpin GmbH
2 Solvent CDCl3
3 Temperature 300
4 Experiment JMOD
5 Spectrometer Frequency 100.62
6 Nucleus 13C
OAc
CH
OEOM
COOMe
48
f1 (ppm)

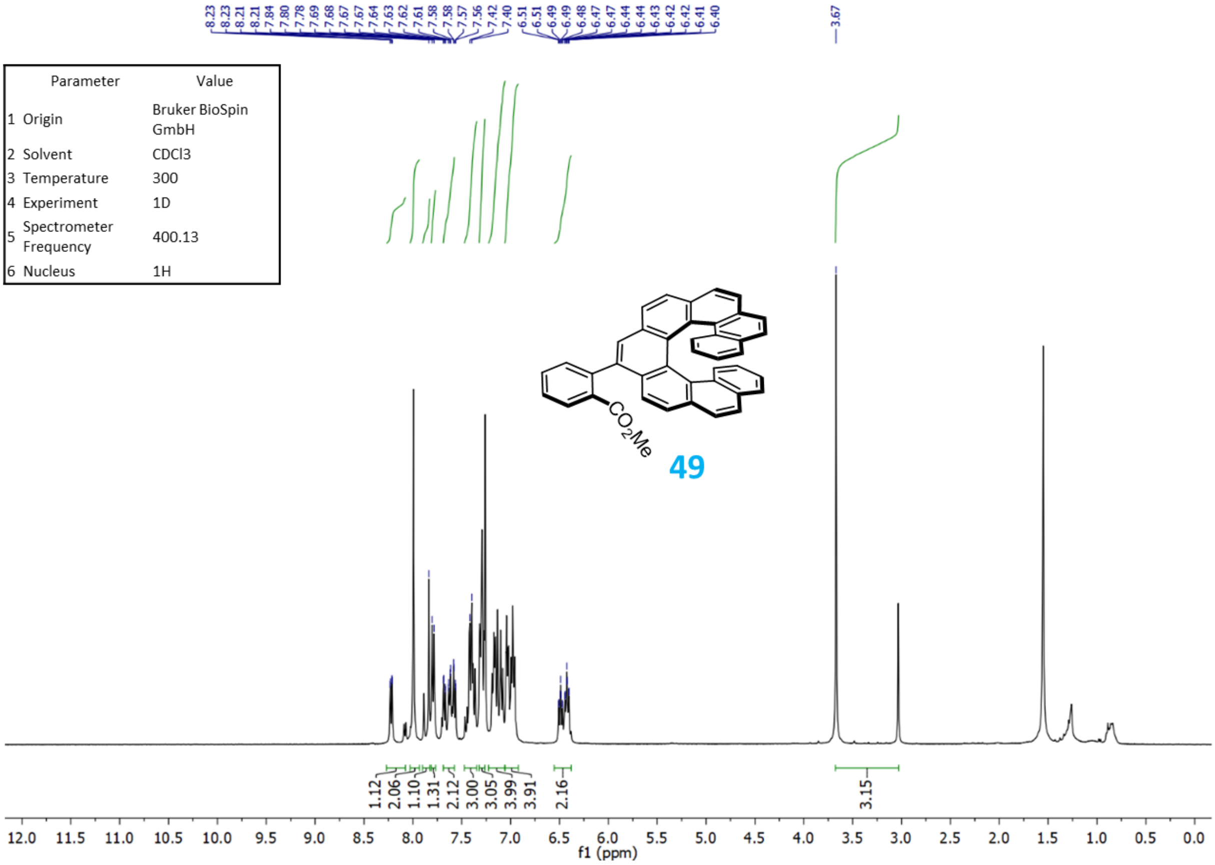

	Parameter	Value
1	Origin	Bruker BioSpin GmbH
2	Solvent	CDCl3
3	Temperature	300
4	Experiment	1D
5	Spectrometer Frequency	400.13
6	Nucleus	1H

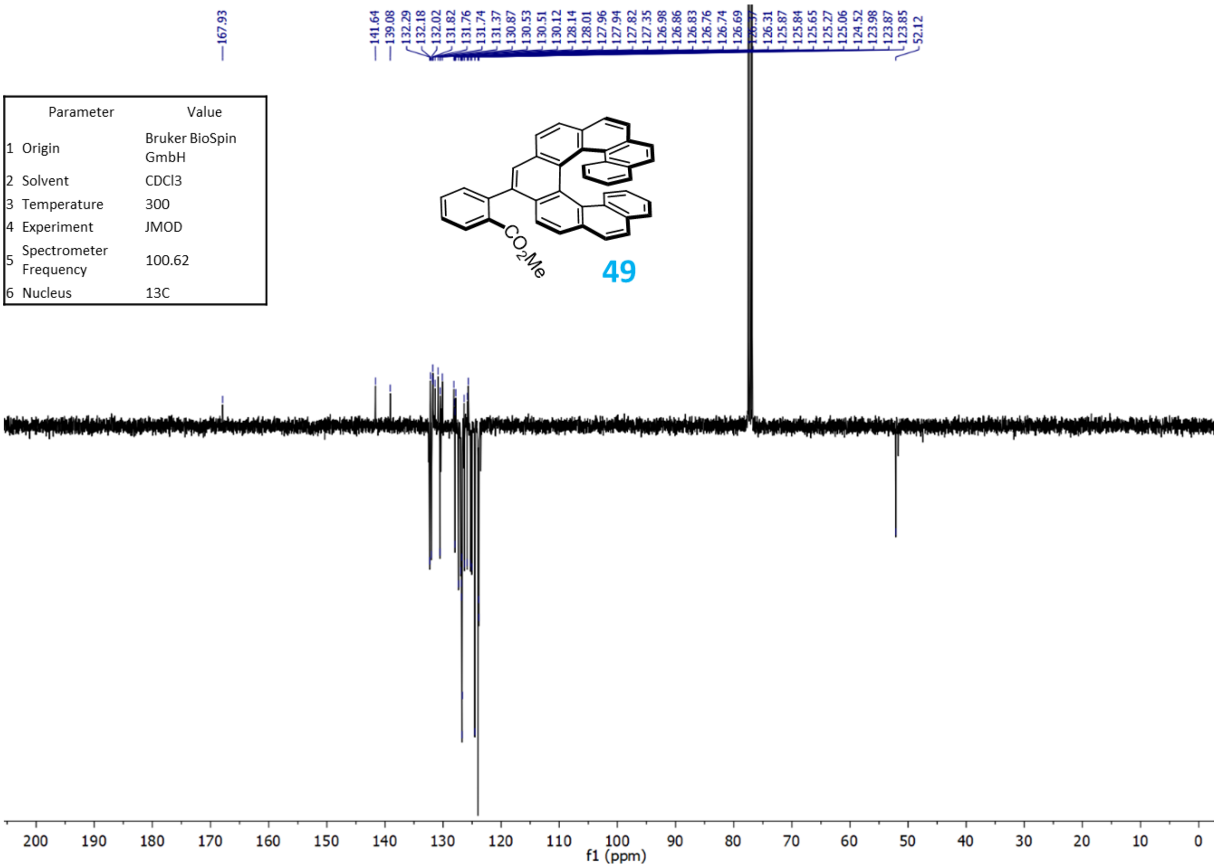

	Parameter	Value
1	Origin	Bruker BioSpin GmbH
2	Solvent	CDCl3
3	Temperature	300
4	Experiment	JMOD
5	Spectrometer Frequency	100.62
6	Nucleus	13C

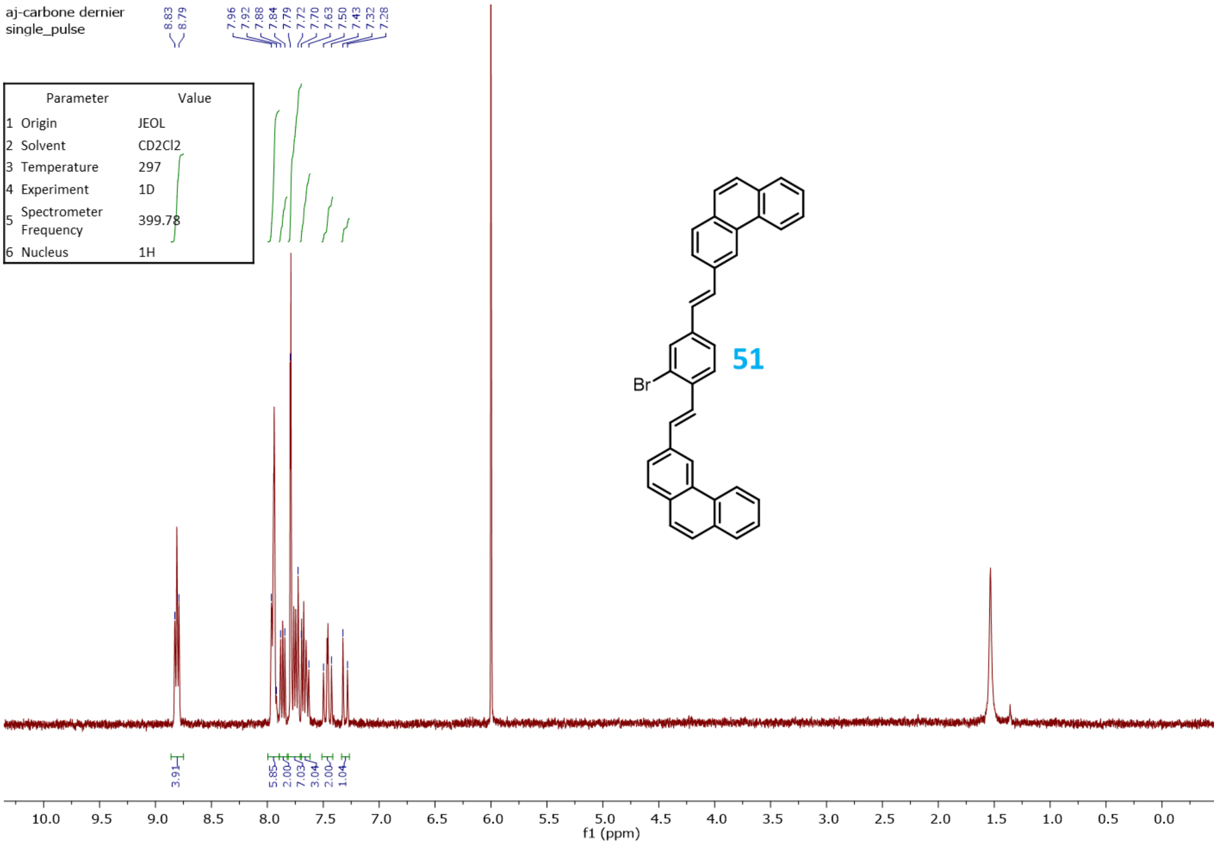
aj-carbone dernier
single_pulse
8.83
8.79
7.96
7.92
7.88
7.84
7.79
7.72
7.70
7.63
7.50
7.43
7.32
7.28
Parameter Value
1 Origin JEOL
2 Solvent CD2Cl2
3 Temperature 297
4 Experiment 1D
5 Spectrometer Frequency 399.78
6 Nucleus 1H
Br
51
3.91
5.85
2.00
7.03
3.04
2.00
1.04
f1 (ppm)

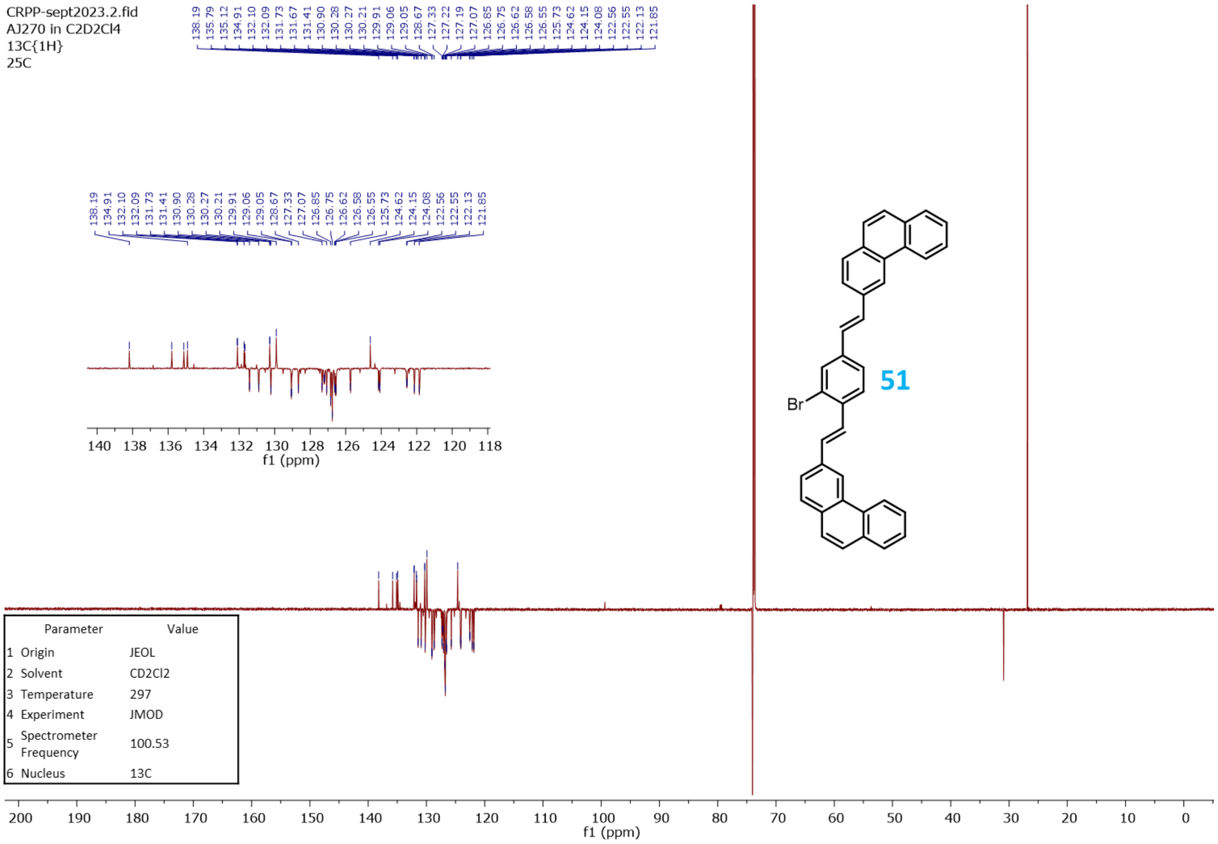
CRPP-sept2023.2.fid
AJ270 in C2D2Cl4
13C{1H}
25C
138.19
135.79
135.12
134.91
132.10
132.09
131.73
131.67
131.41
130.90
130.28
130.27
130.21
129.91
129.06
129.05
128.67
127.33
127.22
127.19
127.07
126.85
126.75
126.62
126.58
126.55
125.73
124.62
124.15
124.08
122.56
122.55
122.13
121.85
f1 (ppm)
Br
51
Parameter Value
1 Origin JEOL
2 Solvent CD2Cl2
3 Temperature 297
4 Experiment JMOD
5 Spectrometer Frequency 100.53
6 Nucleus 13C
f1 (ppm)

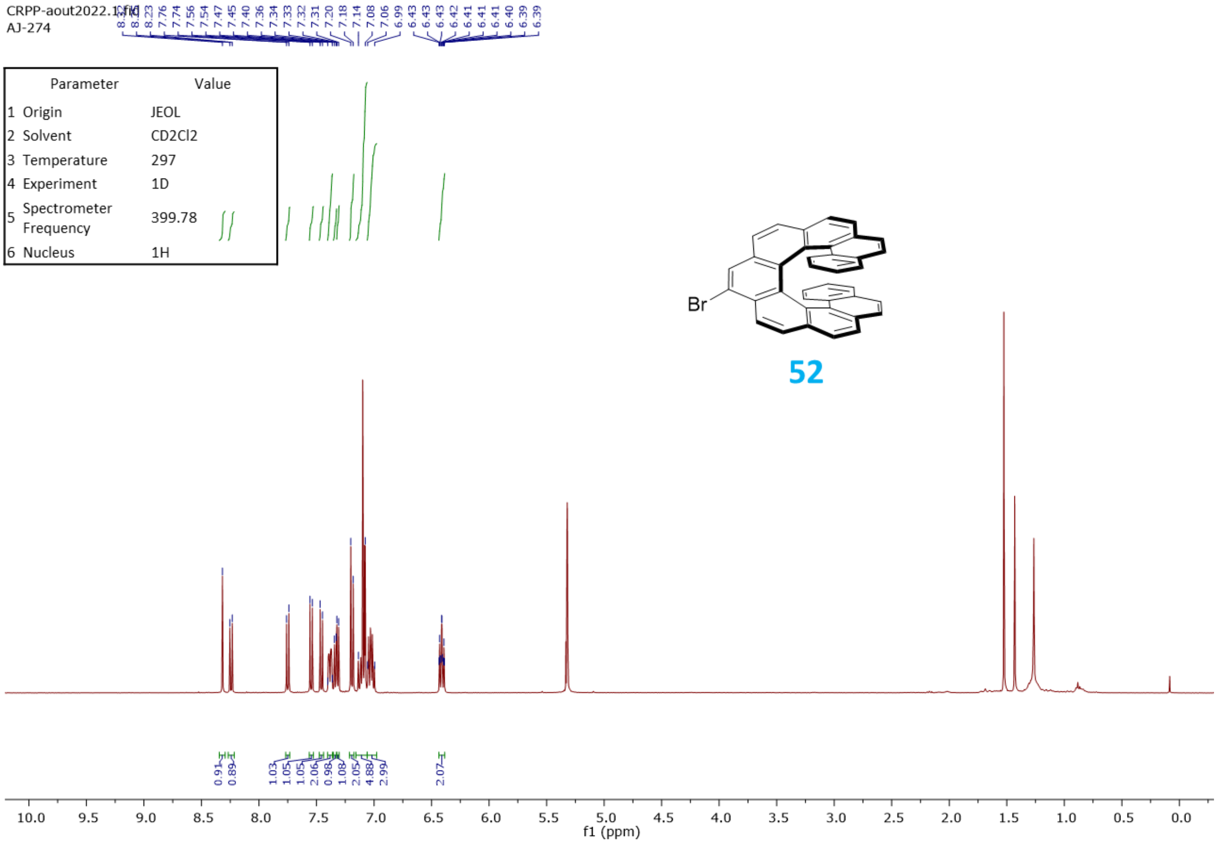
CRPP-aout2022.1.fid
AJ-274
7.76 7.74 7.56 7.54 7.47 7.45 7.40 7.36 7.34 7.33 7.32 7.31 7.20 7.18 7.14 7.08 7.06 6.99 6.43 6.43 6.43 6.42 6.41 6.41 6.40 6.39 6.39
Parameter | Value
1 Origin | JEOL
2 Solvent | CD2Cl2
3 Temperature | 297
4 Experiment | 1D
5 Spectrometer Frequency | 399.78
6 Nucleus | 1H
Br
52
0.91 0.89 1.03 1.05 1.05 2.06 0.98 1.08 2.05 4.88 2.99 2.07
10.0 9.5 9.0 8.5 8.0 7.5 7.0 6.5 6.0 5.5 5.0 4.5 4.0 3.5 3.0 2.5 2.0 1.5 1.0 0.5 0.0
f1 (ppm)

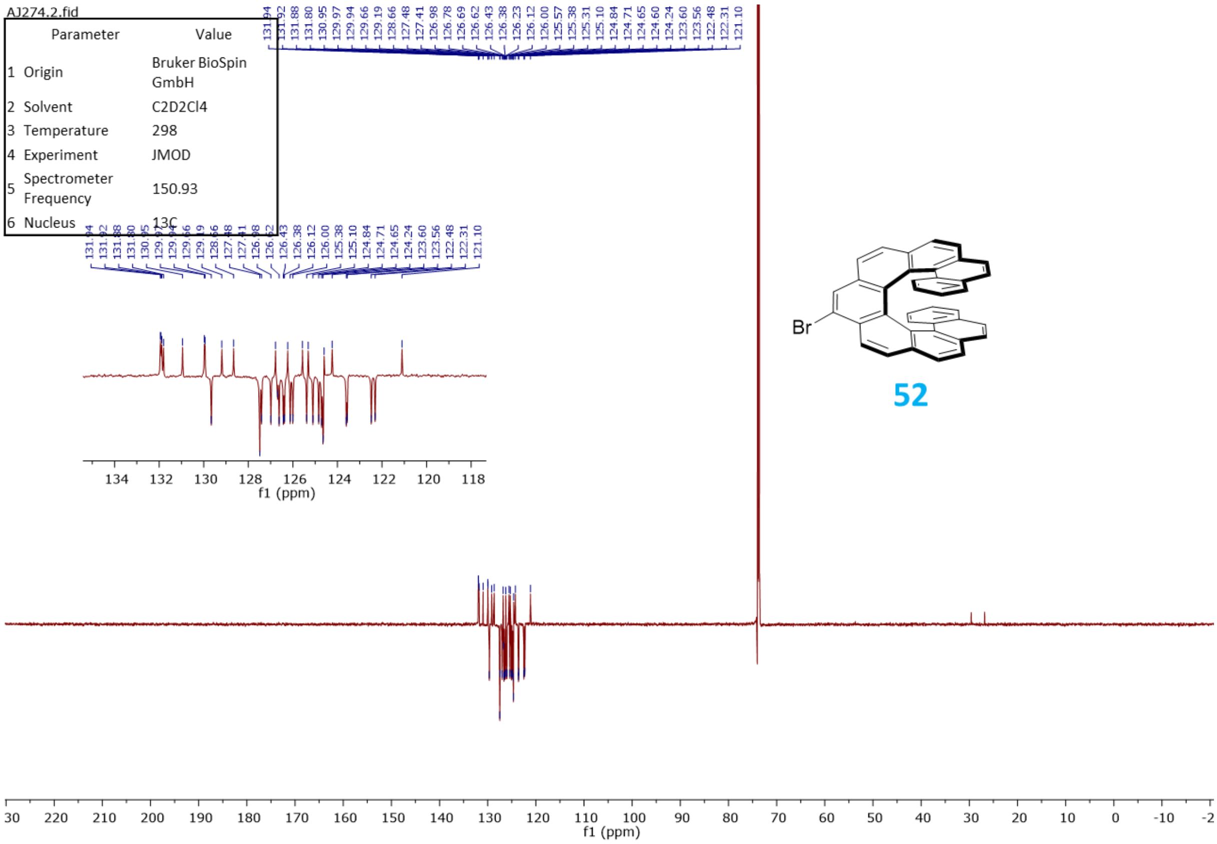
AJ274.2.fid
Parameter | Value
1 Origin | Bruker BioSpin GmbH
2 Solvent | C2D2Cl4
3 Temperature | 298
4 Experiment | JMOD
5 Spectrometer Frequency | 150.93
6 Nucleus | 13C
131.94 131.92 131.88 131.80 130.95 129.97 129.94 129.66 129.19 128.66 127.48 127.41 126.98 126.78 126.69 126.62 126.43 126.38 126.23 126.12 126.00 125.57 125.38 125.31 125.10 124.84 124.71 124.65 124.60 124.24 123.60 123.56 122.48 122.31 121.10
134 132 130 128 126 124 122 120 118
f1 (ppm)
Br
52
220 210 200 190 180 170 160 150 140 130 120 110 100 90 80 70 60 50 40 30 20 10 0 -10
f1 (ppm)

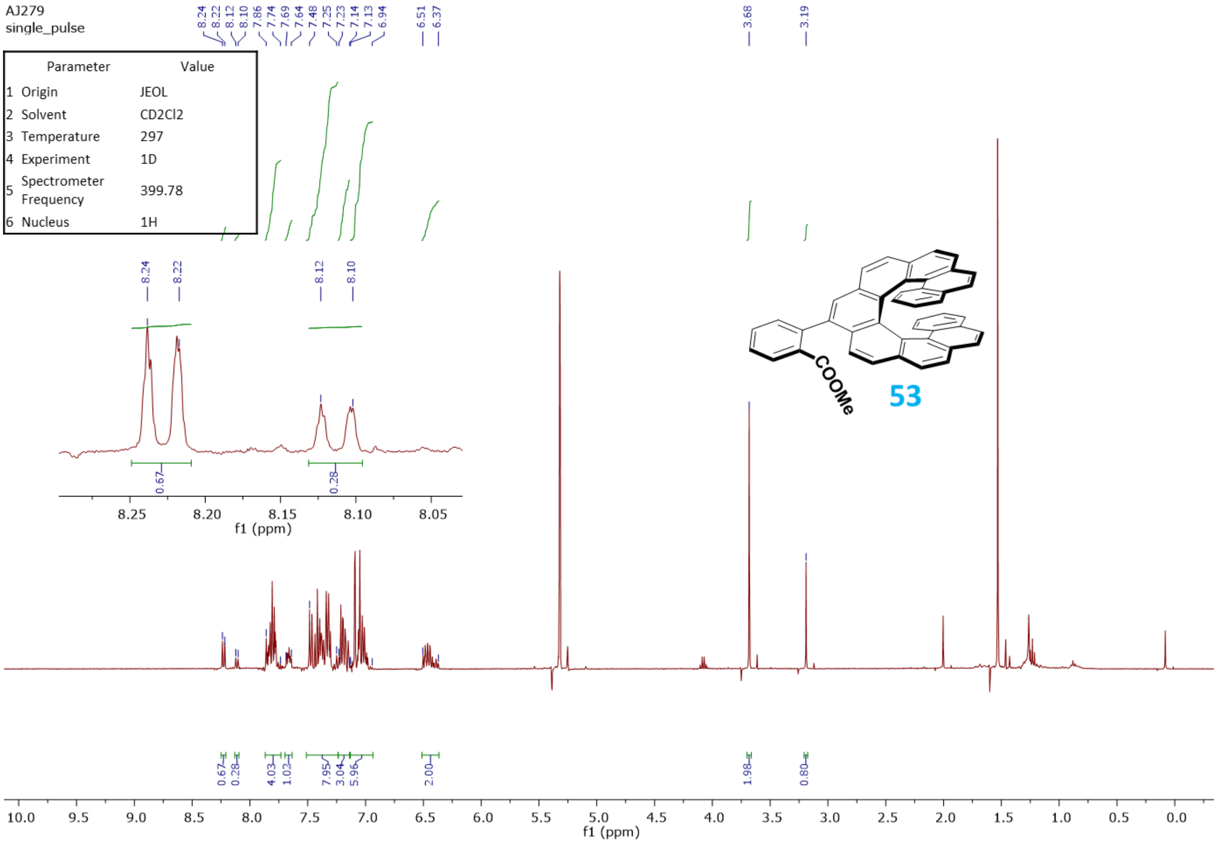
AJ279
single_pulse
Parameter Value
1 Origin JEOL
2 Solvent CD2Cl2
3 Temperature 297
4 Experiment 1D
5 Spectrometer Frequency 399.78
6 Nucleus 1H
8.24 8.22 8.12 8.10 7.86 7.74 7.69 7.64 7.48 7.25 7.23 7.14 7.13 6.94 6.51 6.37 3.68 3.19
0.67 0.28 4.03 1.02 7.95 3.04 5.96 2.00 1.98 0.80
COOMe
53
f1 (ppm)

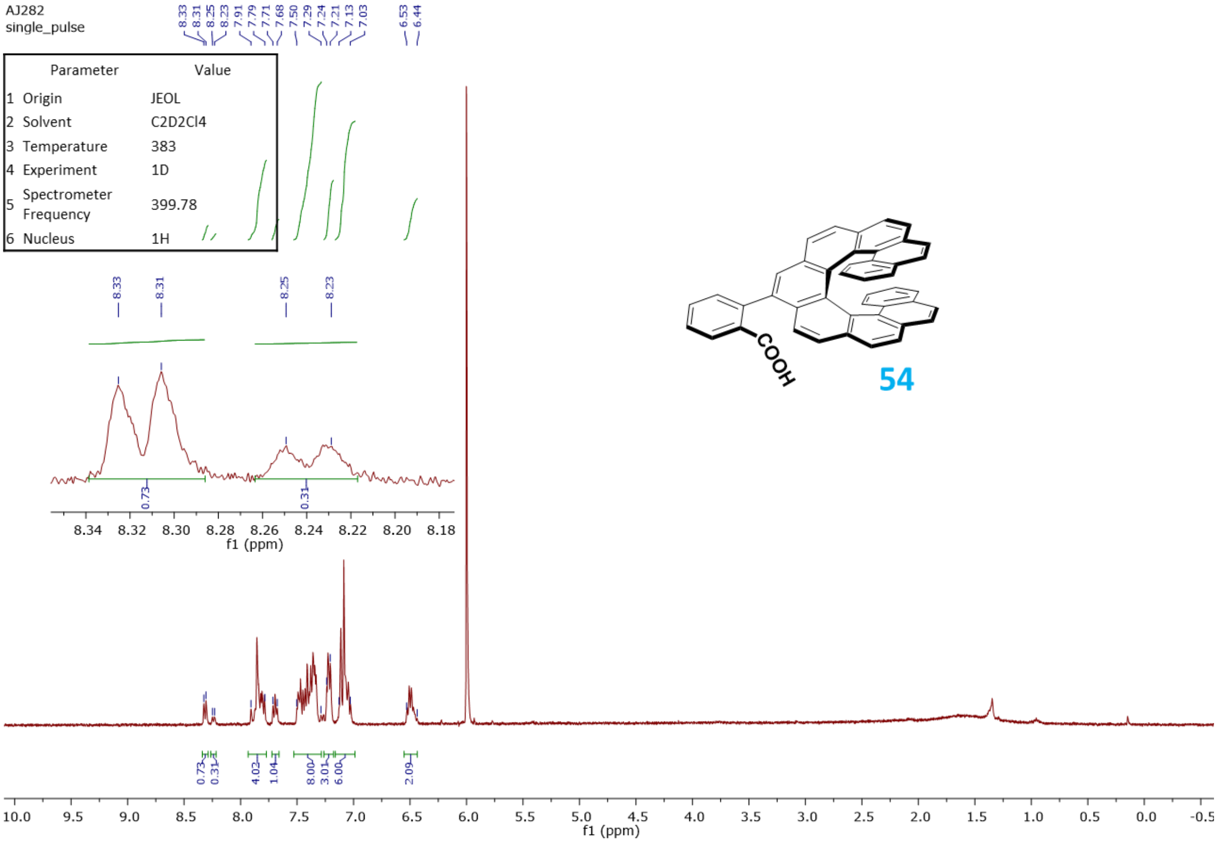
AJ282
single_pulse
Parameter Value
1 Origin JEOL
2 Solvent C2D2Cl4
3 Temperature 383
4 Experiment 1D
5 Spectrometer Frequency 399.78
6 Nucleus 1H
8.33 8.31 8.25 8.23 7.91 7.79 7.71 7.68 7.50 7.29 7.24 7.21 7.13 7.03 6.53 6.44
0.73 0.31 4.02 1.04 8.00 3.01 6.00 2.09
COOH
54
f1 (ppm)

Photophysical and chiroptical properties of 1A(F) – 8A(F)

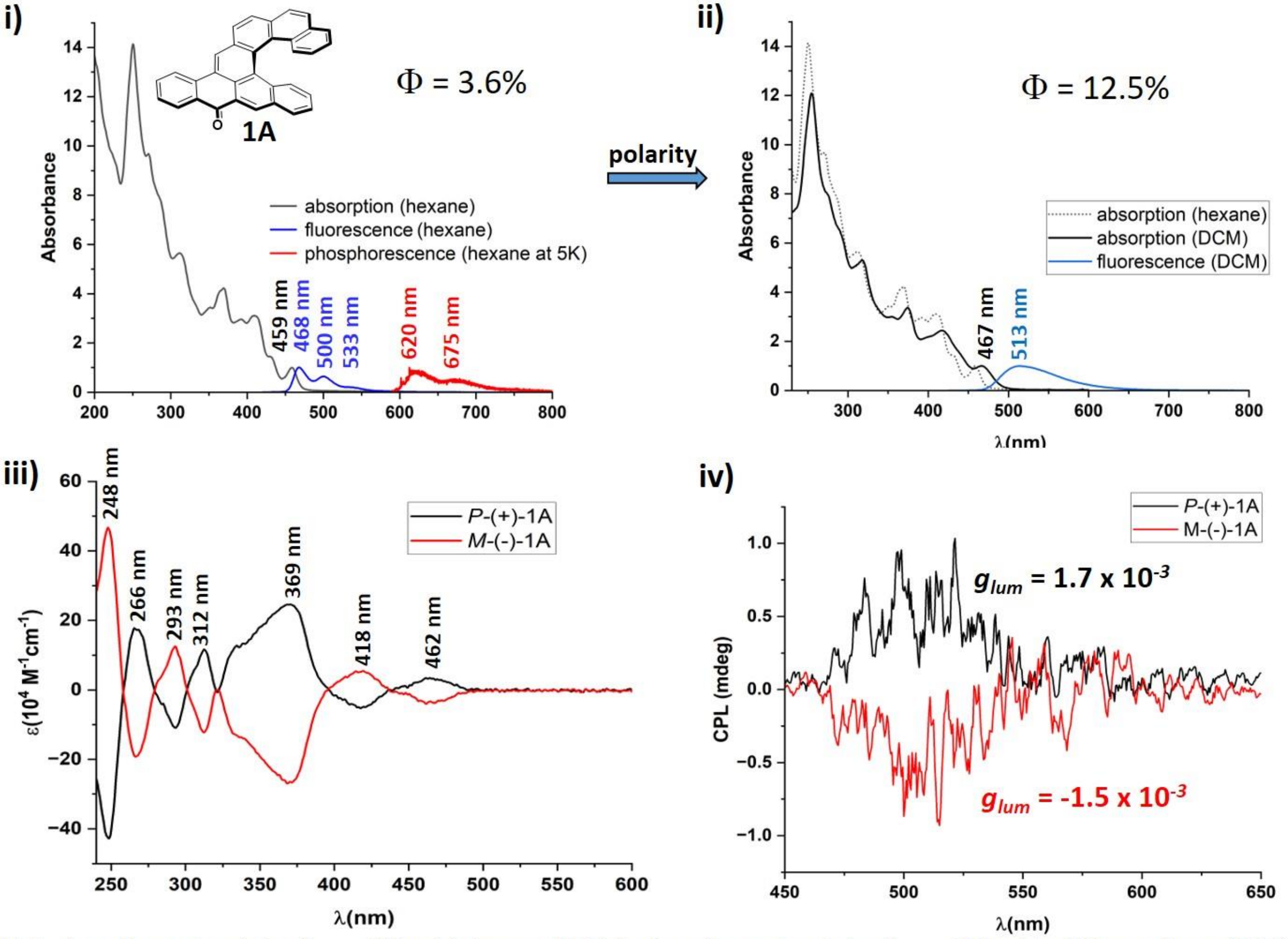

P-(+) Peak at [nm]	g_{abs} DCM
462	$2.2*10^{-3}$
418	$-1.2*10^{-3}$
369	$4.5*10^{-3}$
312	$1.4*10^{-3}$
293	$-1.1*10^{-3}$
266	$1.2*10^{-3}$
248	$-2.6*10^{-3}$

i) UV-vis absorption and emission ($\lambda_{(exc)}$ = 303nm) in hexane **ii)** UV-vis absorption and emission ($\lambda_{(exc)}$ = 303nm) in dichloromethane (DCM) **iii)** CD spectra collected in dichloromethane (DCM) **iv)** CPEL spectra and glum values collected in DCM

a)

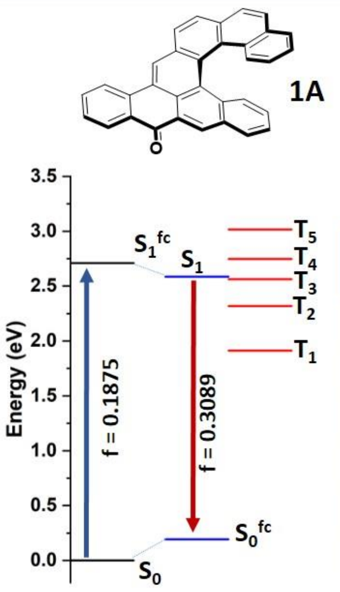

State	E [eV]
S_1	2.587
T_1	1.913
T_2	2.318
T_3	2.563
T_4	2.748
T_5	2.768

b)

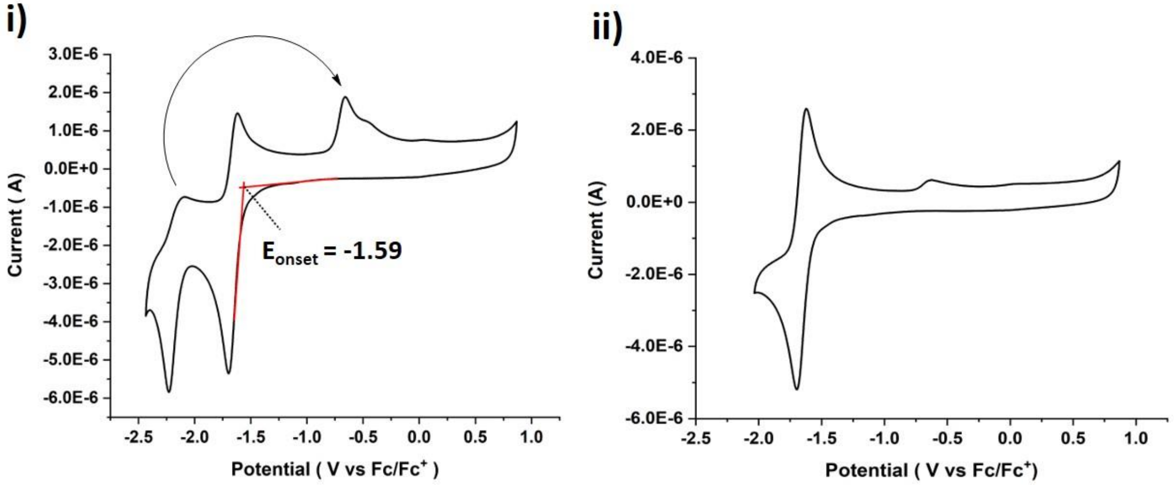

$\lambda_{(max)}$ [nm] DCM	$\lambda_{(em)}$ $\lambda_{(exc)}$ [a] [nm] DCM	g_{lum} [b] (10^{-3}) DCM	$[\alpha]_D^{20}$ DCM	Φ_{PL} [c] (%) hexane/DCM/ACN	t_{fl} [ns] hexane/DCM/ACN	k_r [d] [10^7 s^{-1}] hexane/DCM/ACN	k_{nr} [e] [10^7 s^{-1}] Hexane/DCM/ACN	E_{HOMO} [f] [eV]	E_{LUMO} [g] [eV]	$E_{(0,0)}$ [h] [eV] DCM (hexane)	$E_{(0,0)}$ [i] [eV]	E_{T1-S0} [j] [eV]
467	513(303)	+1.7<br>-1.5	+2147<br>-2167	3.6/12.5/11.4	2.34/4.0/4.25	1.5/3.1/2.7	41.2/21.9/20.8	-5.89	-3.21	2.55<br>(2.68)	2.59	2.08

a) Wavelength of excitation b) Measured in dichloromethane (c ≈ 1 x 10^{-5} M) c) Measured at room temperature (c ≈ 1 x 10^{-6} M) d) radiative rate constant calculated as $k_r = \Phi_{PL}/t_{fl}$ e) nonradiative decay rate constant calculated as $\Phi_{PL} = k_r/(k_r+k_{nr})$ f) Calculated as $E_{HOMO} = E_{LUMO} – E(0,0)$ g) Calculated using the equation $E_{LUMO} = -[E'_{red/onset} + 4.8]$ referenced against Fc/Fc$^+$. h) Determined from the intersection of the absorption and emission curves and using equation $E_g = hc/\lambda$ i) Calculated by TDDFT M06/6-31G(d,p) method j) determined from the phosphorescence λ_{onset}

a) Energy-level diagram calculated by TDDFT M06/6-31G(d,p) method in ACN. **b)** CV voltammograms vs Fc/Fc$^+$ redox couple in acetonitrile (ACN) with a supporting electrolyte $[Bu_4N][PF_6]$ (0.1M), at a scan rate 0.1 V s^{-1} **i)** full CV voltammogram collected in the full range of ACN, the second irreversible reduction process lead to a product(s) with oxidation peak located at -0.6 V **ii)** CV voltammogram of the first reversible one-electron process.

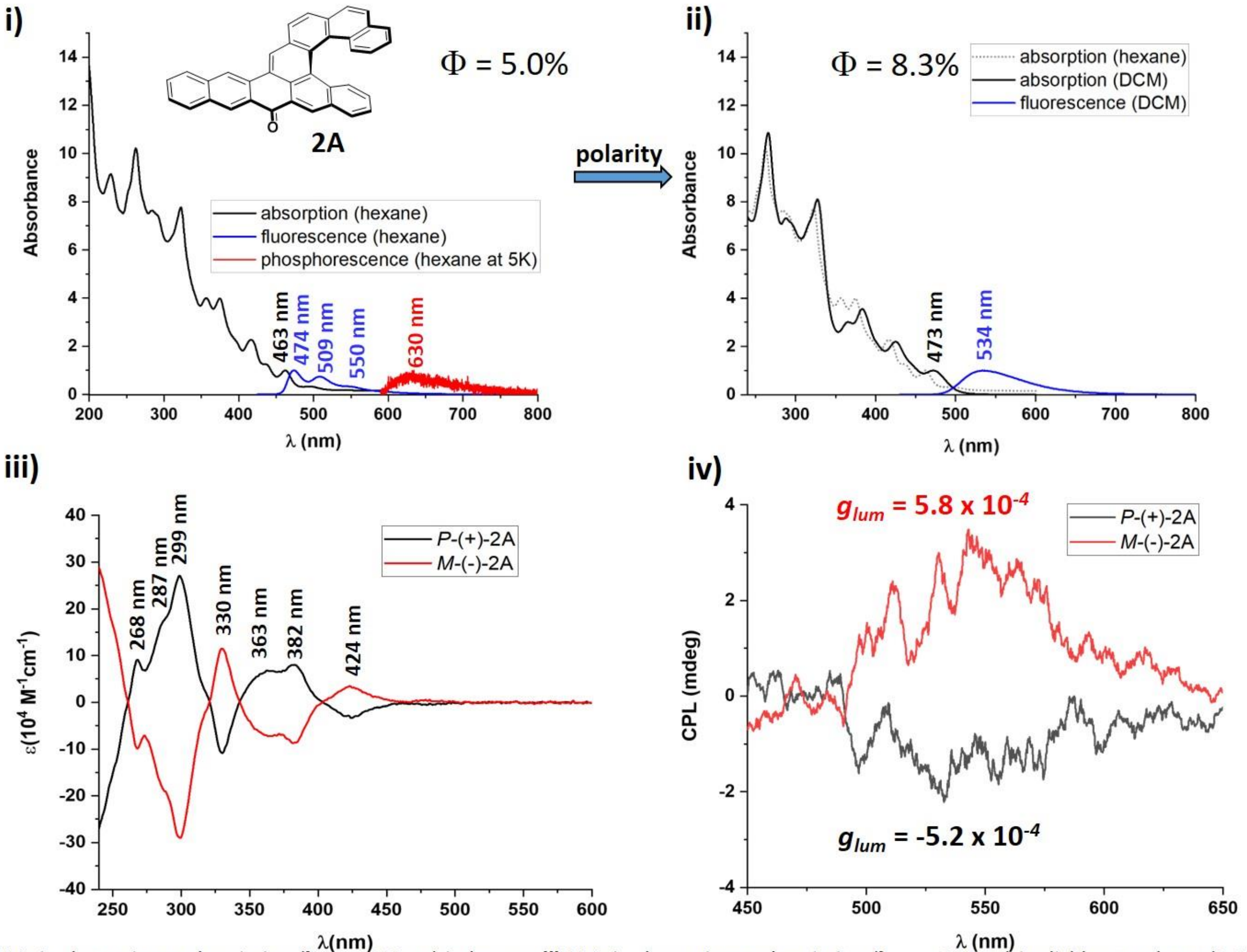

P-(+) Peak at [nm]	g_{abs}
424	$-1.2*10^{-3}$
382	$1.9*10^{-3}$
363	$2.0*10^{-3}$
330	$-1.2*10^{-3}$
299	$3.2*10^{-3}$
287	$2.0*10^{-3}$
268	$0.7*10^{-3}$

i) UV-vis absorption and emission ($\lambda_{(exc)}$ = 303nm) in hexane **ii)** UV-vis absorption and emission ($\lambda_{(exc)}$ = 303nm) in dichloromethane (DCM) **iii)** CD spectra collected in dichloromethane (DCM) **iv)** CPEL spectra and glum values collected in DCM

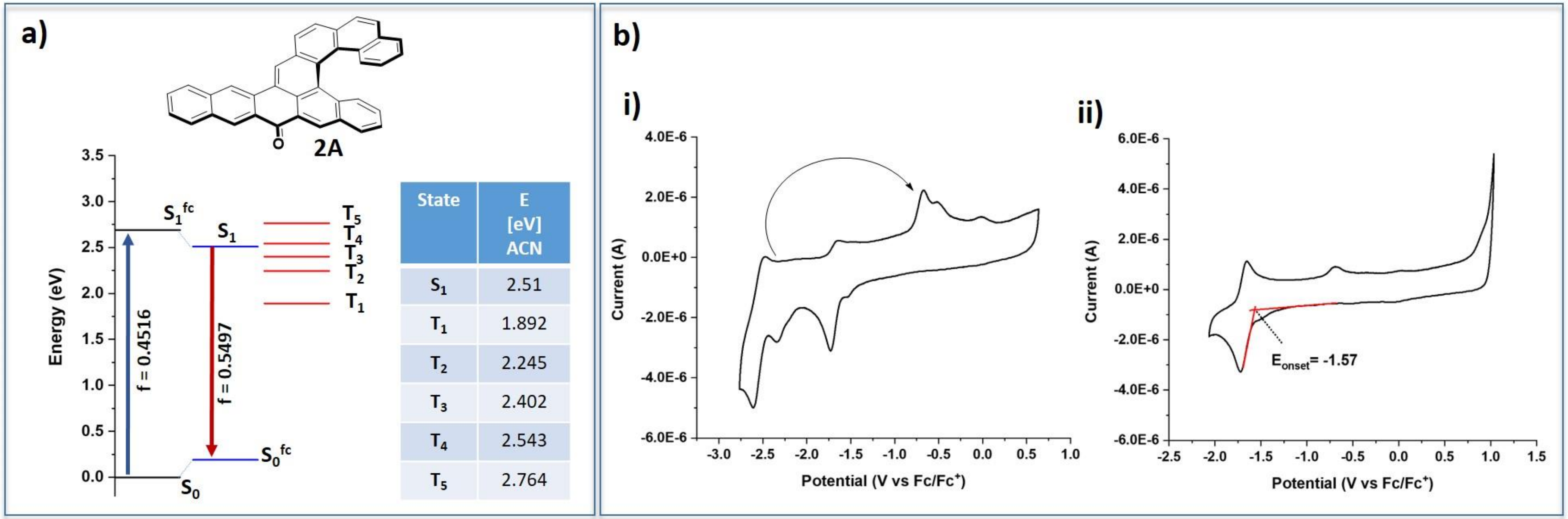

State	E [eV] ACN
S_1	2.51
T_1	1.892
T_2	2.245
T_3	2.402
T_4	2.543
T_5	2.764

$\lambda_{(max)}$ [nm] DCM	$\lambda_{(em)}$ $\lambda_{(exc)}$[a] [nm] DCM	g_{lum}[b] (10^{-3}) DCM	$[\alpha]_D^{20}$ DCM	Φ_{PL}[c] (%) hexane/DCM/ACN	t_{fl} [ns] hexane/DCM/ACN	k_r[d] [10^7 s^{-1}] hexane/DCM/ACN	k_{nr}[e] [10^7 s^{-1}] Hexane/DCM/ACN	E_{HOMO}[f] [eV]	E_{LUMO}[g] [eV]	$E_{(0,0)}$[h] [eV] DCM (hexane)	$E_{(0,0)}$[i] [eV]	E_{T1-S0}[j] [eV]
473	534(303)	+0.58<br>-0.52	+770<br>-748	5.0/8.3/7.6	1.93/2.71/2.5	2.7/3.0/3.0	50.5/33.9/37	-5.88	-3.23	2.49<br>(2.65)	2.51	2.09

a) Wavelength of excitation b) Measured in dichloromethane (c ≈ 1 x 10^{-5} M) c) Measured at room temperature (c ≈ 1 x 10^{-6} M) d) radiative rate constant calculated as $k_r = \Phi_{PL}/t_{fl}$ e) nonradiative decay rate constant calculated as $\Phi_{PL} = k_r/(k_r+k_{nr})$ f) Calculated as $E_{HOMO} = E_{LUMO} - E(0,0)$ g) Calculated using the equation $E_{LUMO} = -[E'_{red/onset} + 4.8]$ referenced against Fc/Fc+. h) Determined from the intersection of the absorption and emission curves and using equation $E_g = hc/\lambda$ i) Calculated by TDDFT M06/6-31G(d,p) method j) determined from the phosphorescence λ_{onset}

a) Energy-level diagram calculated by TDDFT M06/6-31G(d,p) method in ACN. **b)** CV voltammograms vs Fc/Fc+ redox couple in acetonitrile (ACN) with a supporting electrolyte $[Bu_4N][PF_6]$ (0.1M), at a scan rate 0.1 V s^{-1} **i)** full CV voltammogram collected in the full range of ACN, the second irreversible reduction process lead to a product(s) with oxidation peak located at -0.6 V **ii)** CV voltammogram of the first reversible one-electron process.

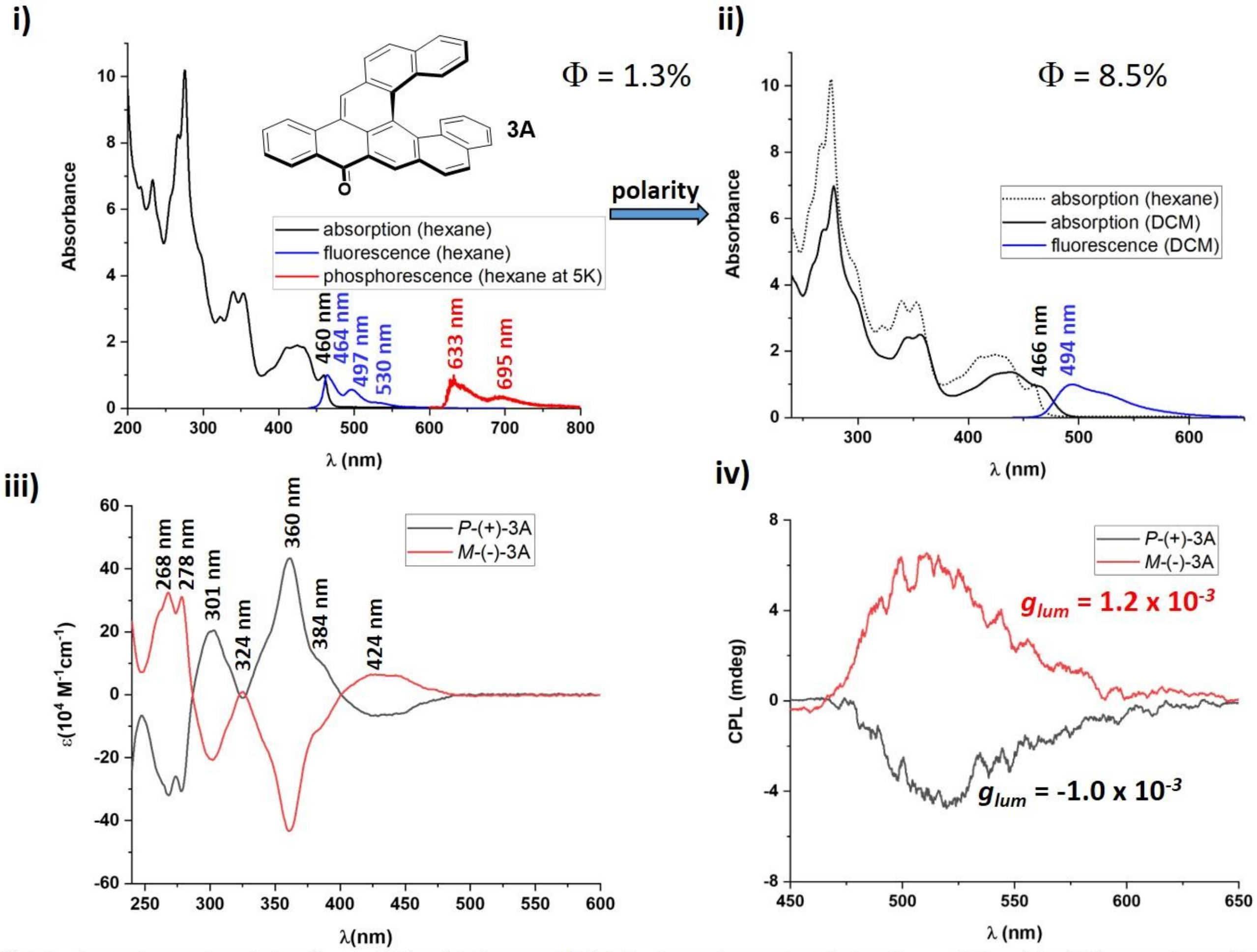

P-(+) Peak at [nm]	g_{abs}
424	$-1.7*10^{-3}$
384	$6.0*10^{-3}$
360	$6.0*10^{-3}$
324	$-3.0*10^{-3}$
301	$2.0*10^{-3}$
278	$-1.5*10^{-3}$
268	$-2.0*10^{-3}$

i) UV-vis absorption and emission ($\lambda_{(exc)}$ = 303nm) in hexane **ii)** UV-vis absorption and emission ($\lambda_{(exc)}$ = 303nm) in dichloromethane (DCM) **iii)** CD spectra collected in dichloromethane (DCM) **iv)** CPEL spectra and glum values collected in DCM

a)

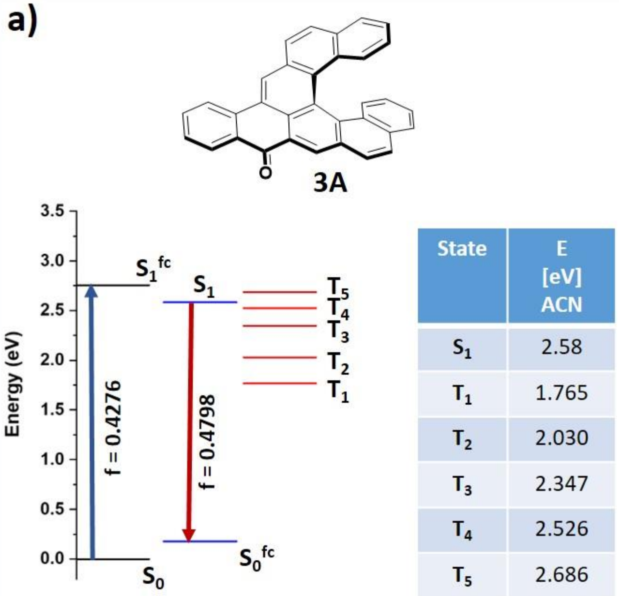

State	E [eV] ACN
S_1	2.58
T_1	1.765
T_2	2.030
T_3	2.347
T_4	2.526
T_5	2.686

b)

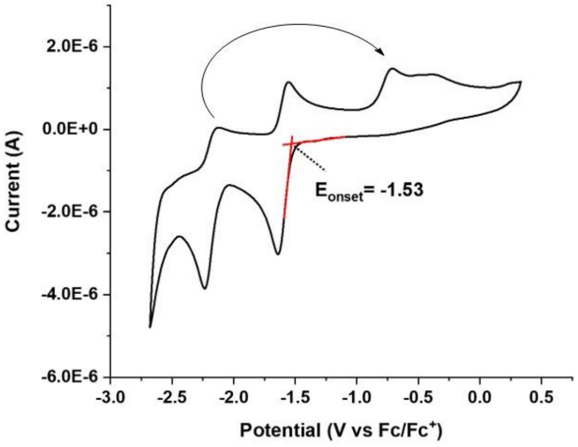

$\lambda_{(max)}$ [nm] DCM	$\lambda_{(em)}$ $\lambda_{(exc)}$ [a] [nm] DCM	g_{lum} [b] (10^{-3}) DCM	$[\alpha]_D^{20}$ DCM	Φ_{PL} [c] (%) hexane/DCM/ACN	t_{fl} [ns] hexane/DCM/ACN	k_r [d] [10^7 s^{-1}] hexane/DCM/ACN	k_{nr} [e] [10^7 s^{-1}] Hexane/DCM/ACN	E_{HOMO} [f] [eV]	E_{LUMO} [g] [eV]	$E_{(0,0)}$ [h] [eV] DCM (hexane)	$E_{(0,0)}$ [i] [eV]	E_{T1-S0} [j] [eV]
466	494(303)	+1.2<br>-1.0	+1094<br>-1031	1.3/8.5/7.8	0.79/2.71/2.89	1.6/3.1/2.7	125.2/33.8/31.9	-5.95	-3.27	2.60<br>(2.68)	2.58	2.00

a) Wavelength of excitation b) Measured in dichloromethane (c ≈ 1 x 10^{-5} M) c) Measured at room temperature (c ≈ 1 x 10^{-6} M) d) radiative rate constant calculated as $k_r = \Phi_{PL}/t_{fl}$ e) nonradiative decay rate constant calculated as $\Phi_{PL} = k_r/(k_r+k_{nr})$ f) Calculated as $E_{HOMO} = E_{LUMO} - E(0,0)$ g) Calculated using the equation $E_{LUMO} = -[E'_{red/onset} + 4.8]$ referenced against Fc/Fc^+. h) Determined from the intersection of the absorption and emission curves and using equation $E_g = hc/\lambda$ i) Calculated by TDDFT M06/6-31G(d,p) method j) determined from the phosphorescence λ_{onset}

a) Energy-level diagram calculated by TDDFT M06/6-31G(d,p) method in ACN. **b)** CV voltammogram vs Fc/Fc^+ redox couple in acetonitrile (ACN) with a supporting electrolyte $[Bu_4N][PF_6]$ (0.1M), at a scan rate 0.1 V s^{-1}, the second irreversible reduction process lead to a product(s) with oxidation peak located at -0.6 V.

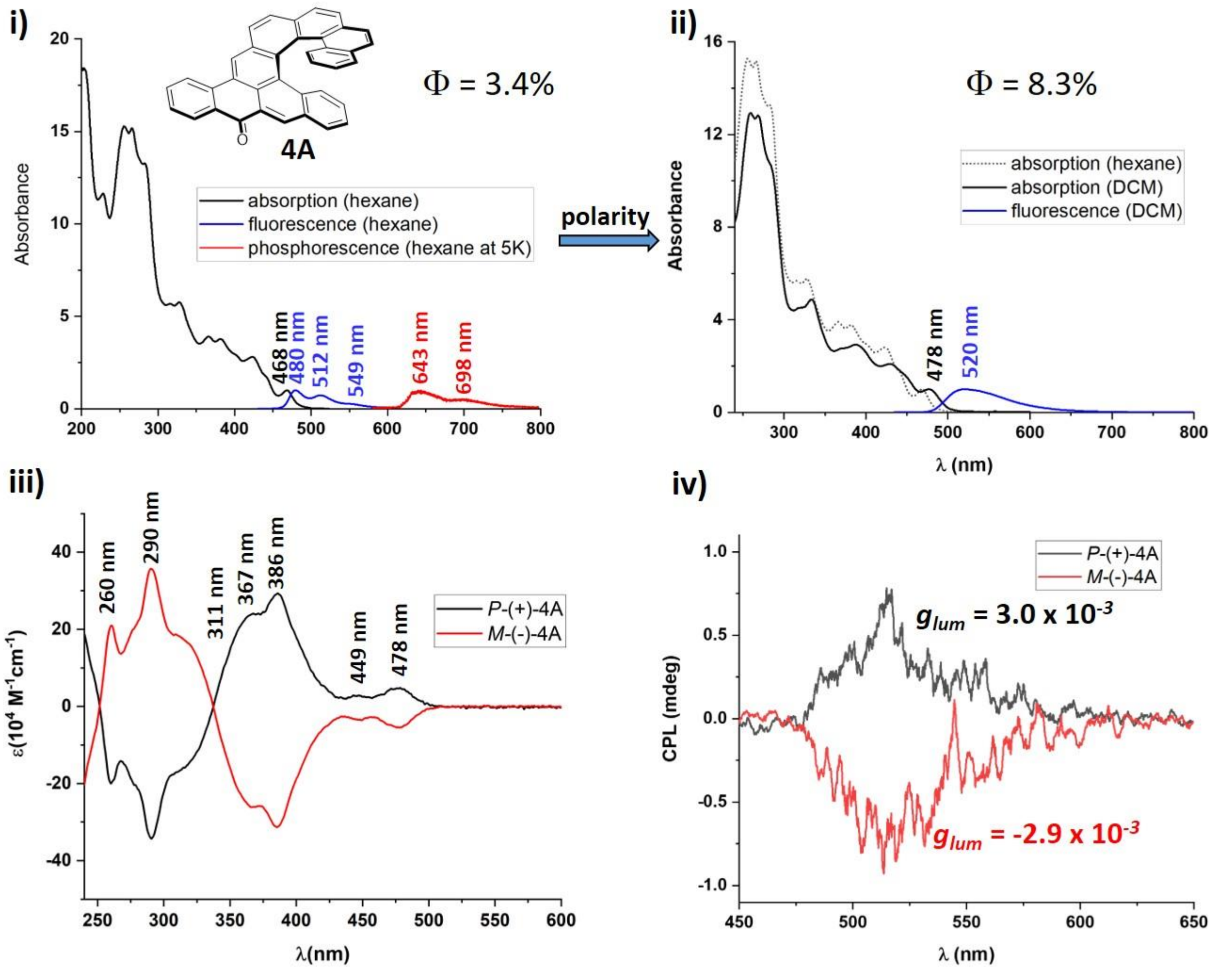

***P*-(+) Peak at [nm]**	**g_{abs}**
478	3.6*10^{-3}
449	1.3*10^{-3}
386	7.5*10^{-3}
367	6.7*10^{-3}
311	-3.0*10^{-3}
290	-2.7*10^{-3}
260	-1.3*10^{-3}

i) UV-vis absorption and emission ($\lambda_{(exc)}$ = 336 nm) in hexane **ii)** UV-vis absorption and emission ($\lambda_{(exc)}$ = 336 nm) in dichloromethane (DCM) **iii)** CD spectra collected in dichloromethane (DCM) **iv)** CPEL spectra and glum values collected in DCM

a)

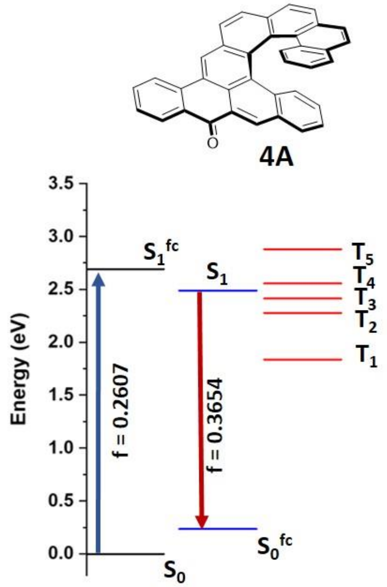

State	E [eV] ACN
S_1	2.49
T_1	1.837
T_2	2.278
T_3	2.412
T_4	2.559
T_5	2.879

b)

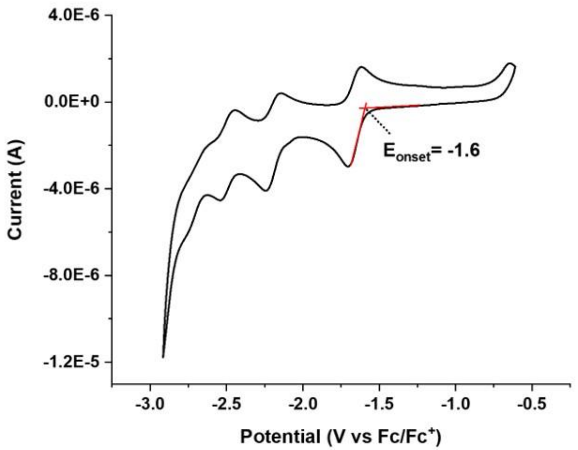

$\lambda_{(max)}$ [nm] DCM	$\lambda_{(em)}$ $\lambda_{(exc)}$ [a] [nm] DCM	g_{lum} [b] (10^{-3}) DCM	$[\alpha]_D^{20}$ DCM	Φ_{PL} [c] (%) hexane/DCM/ACN	t_{fl} [ns] hexane/DCM/ACN	k_r [d] [10^7 s^{-1}] hexane/DCM/ACN	k_{nr} [e] [10^7 s^{-1}] Hexane/DCM/ACN	E_{HOMO} [f] [eV]	E_{LUMO} [g] [eV]	$E_{(0,0)}$ [h] [eV] DCM (hexane)	$E_{(0,0)}$ [i] [eV]	E_{T1-S0} [j] [eV]
478	520(336)	+3.0<br>-2.9	+3493<br>-3526	3.4/8.3/7.6	2.76/3.68/3.85	1.21/2.25/1.97	35.0/24.9/24.0	-5.82	-3.2	2.51<br>(2.62)	2.49	2.00

a) Wavelength of excitation b) Measured in dichloromethane (c ≈ 1 x 10^{-5} M) c) Measured at room temperature (c ≈ 1 x 10^{-6} M) d) radiative rate constant calculated as $k_r = \Phi_{PL}/t_{fl}$ e) nonradiative decay rate constant calculated as $\Phi_{PL} = k_r/(k_r+k_{nr})$ f) Calculated as $E_{HOMO} = E_{LUMO} - E(0,0)$ g) Calculated using the equation $E_{LUMO} = -[E'_{red/onset} + 4.8]$ referenced against Fc/Fc^+. h) Determined from the intersection of the absorption and emission curves and using equation $E_g = hc/\lambda$ i) Calculated by TDDFT M06/6-31G(d,p) method j) determined from the phosphorescence λ_{onset}

a) Energy-level diagram calculated by TDDFT M06/6-31G(d,p) method in ACN. **b)** CV voltammogram vs Fc/Fc^+ redox couple in acetonitrile (ACN) with a supporting electrolyte $[Bu_4N][PF_6]$ (0.1M), at a scan rate 0.1 V s^{-1}

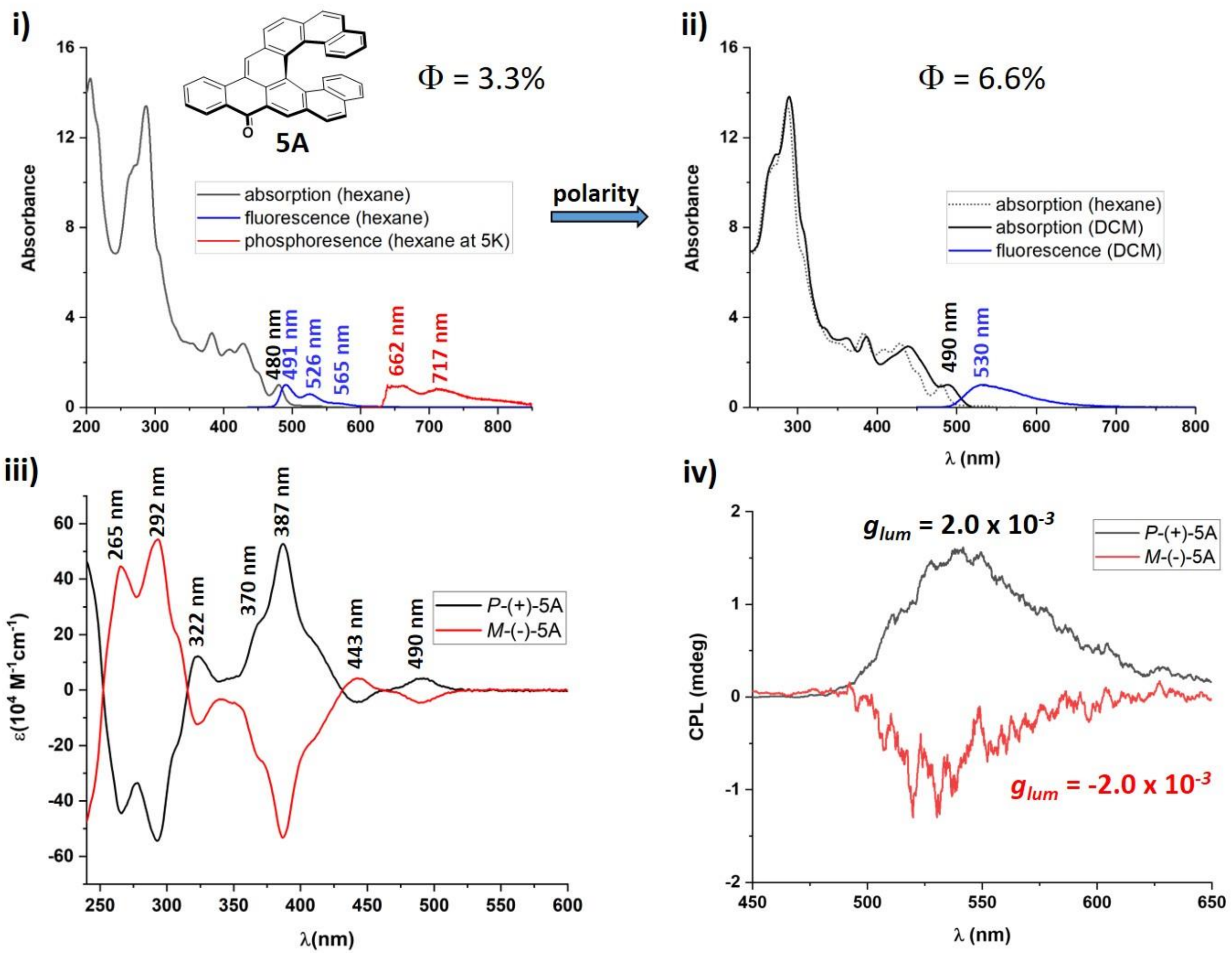

***P*-(+) Peak at [nm]**	**g_{abs}**
490	-2.8*10^{-3}
443	1.0*10^{-3}
387	-10.0*10^{-3}
370	-5.5*10^{-3}
322	-1.6*10^{-3}
292	2.5*10^{-3}
265	2.6*10^{-3}

i) UV-vis absorption and emission ($\lambda_{(exc)}$ = 303nm) in hexane **ii)** UV-vis absorption and emission ($\lambda_{(exc)}$ = 303nm) in dichloromethane (DCM) **iii)** CD spectra collected in dichloromethane (DCM) **iv)** CPEL spectra and glum values collected in DCM

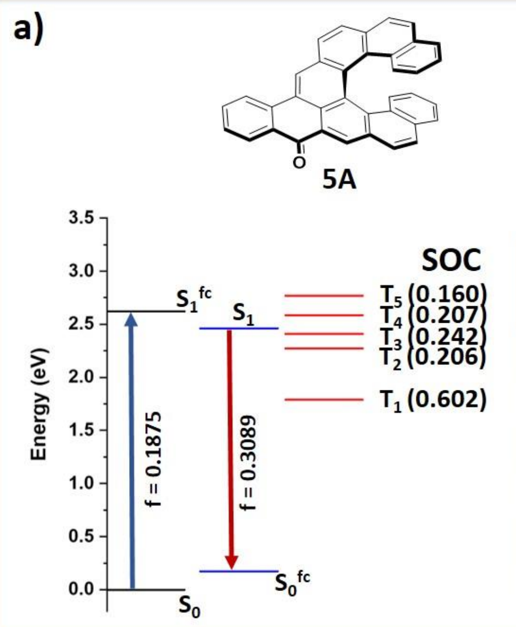

State	E [eV] ACN
S_1	2.46
T_1	1.787
T_2	2.272
T_3	2.409
T_4	2.586
T_5	2.768

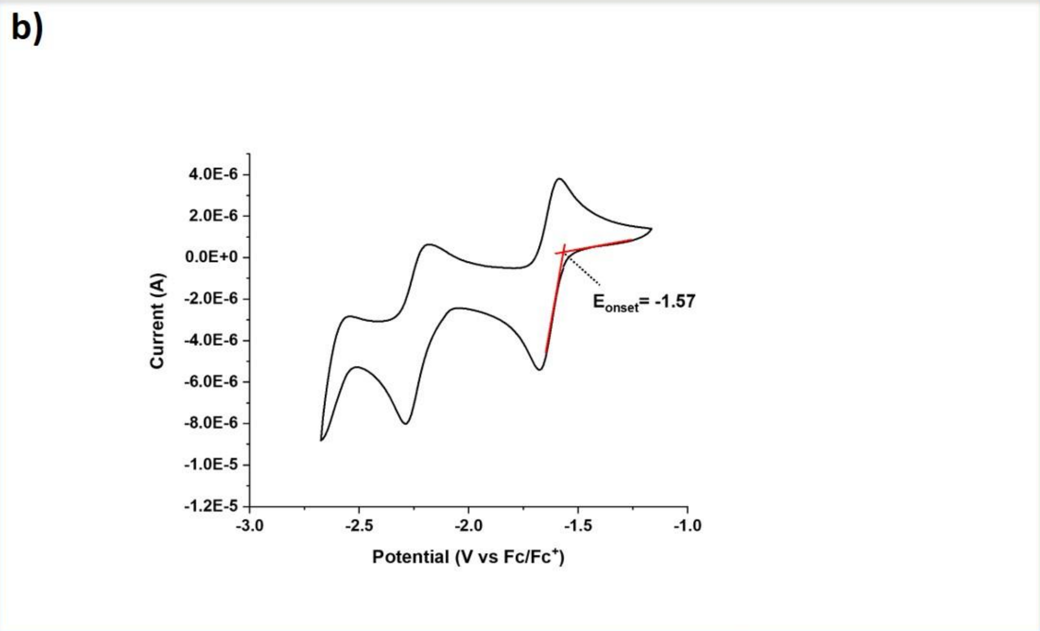

$\lambda_{(max)}$ [nm] DCM	$\lambda_{(em)}$ $\lambda_{(exc)}$[a] [nm] DCM	g_{lum}[b] (10^{-3}) DCM	$[\alpha]_D^{20}$ DCM	Φ_{PL}[c] (%) hexane/DCM/ACN	t_{fl} [ns] hexane/DCM/ACN	k_r[d] [10^7 s^{-1}] hexane/DCM/ACN	k_{nr}[e] [10^7 s^{-1}] Hexane/DCM/ACN	E_{HOMO}[f] [eV]	E_{LUMO}[g] [eV]	$E_{(0,0)}$[h] [eV] DCM (hexane)	$E_{(0,0)}$[i] [eV]	E_{T1-S0}[j] [eV]
490	530(303)	+2.0<br>-2.0	+3620<br>-3672	3.3/6.6/5.8	2.56/3.42/4.09	1.3/1.9/1.4	37.8/27.3.8/23.0	-5.78	-3.23	2.45<br>(2.55)	2.46	1.96

a) Wavelength of excitation b) Measured in dichloromethane (c ≈ 1 x 10^{-5} M) c) Measured at room temperature (c ≈ 1 x 10^{-6} M) d) radiative rate constant calculated as $k_r = \Phi_{PL}/t_{fl}$ e) nonradiative decay rate constant calculated as $\Phi_{PL} = k_r/(k_r+k_{nr})$ f) Calculated as $E_{HOMO} = E_{LUMO} - E(0,0)$ g) Calculated using the equation $E_{LUMO} = -[E'_{red/onset} + 4.8]$ referenced against Fc/Fc^+. h) Determined from the intersection of the absorption and emission curves and using equation $E_g = hc/\lambda$ i) Calculated by TDDFT M06/6-31G(d,p) method j) determined from the phosphorescence λ_{onset}

a) Energy-level diagram calculated by TDDFT M06/6-31G(d,p) method in ACN. **b)** CV voltammogram vs Fc/Fc^+ redox couple in acetonitrile (ACN) with a supporting electrolyte $[Bu_4N][PF_6]$ (0.1M), at a scan rate 0.1 V s^{-1}

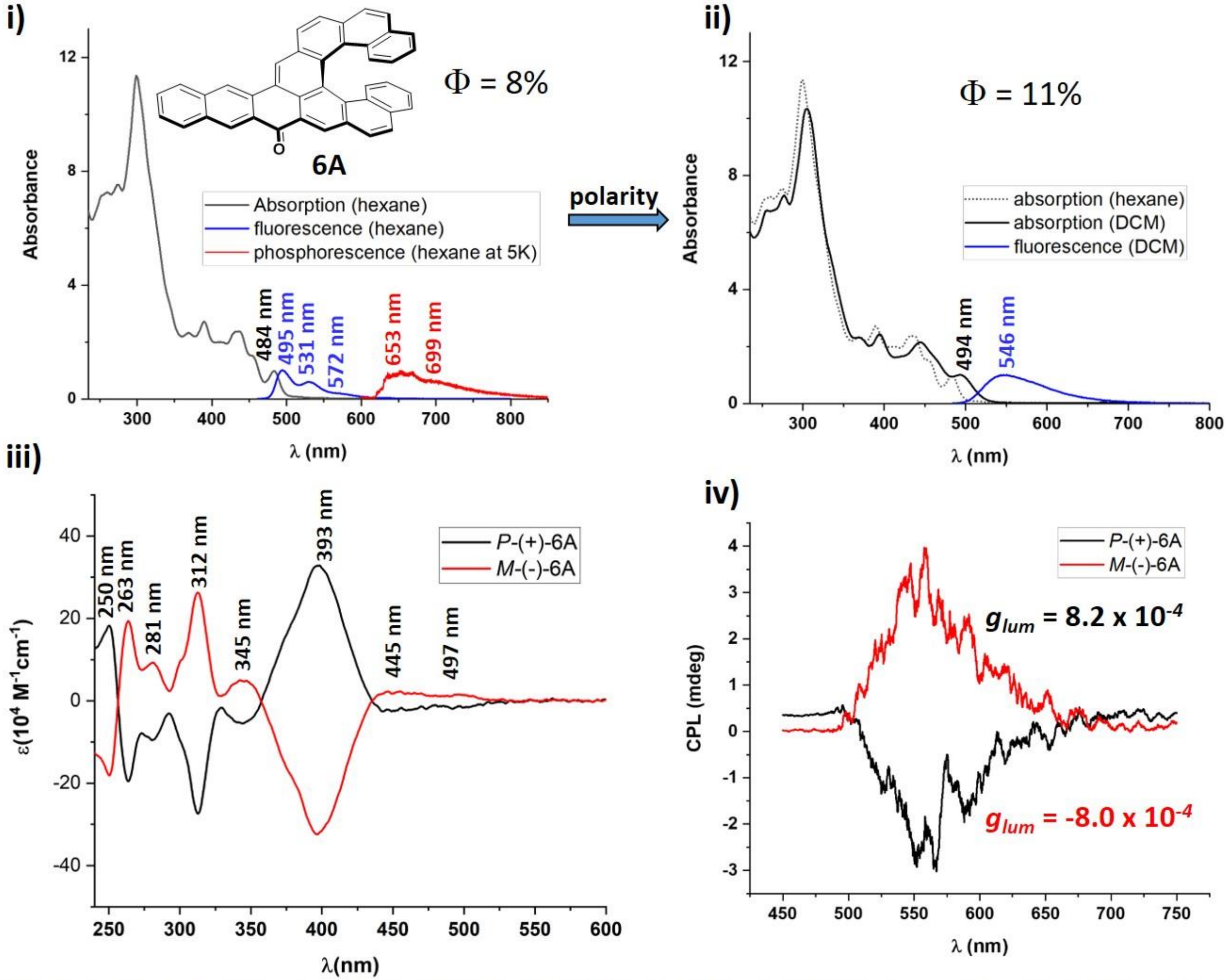

***P*-(+) Peak at [nm]**	**g_{abs}**
497	-0.7*10^{-3}
445	-0.4*10^{-3}
393	6.0*10^{-3}
345	-0.6*10^{-3}
312	-1.3*10^{-3}
281	-0.6*10^{-3}
263	-1.2*10^{-3}
250	1.3*10^{-3}

i) UV-vis absorption and emission ($\lambda_{(exc)}$ = 303nm) in hexane **ii)** UV-vis absorption and emission ($\lambda_{(exc)}$ = 303nm) in dichloromethane (DCM) **iii)** CD spectra collected in dichloromethane (DCM) **iv)** CPEL spectra and glum values collected in DCM

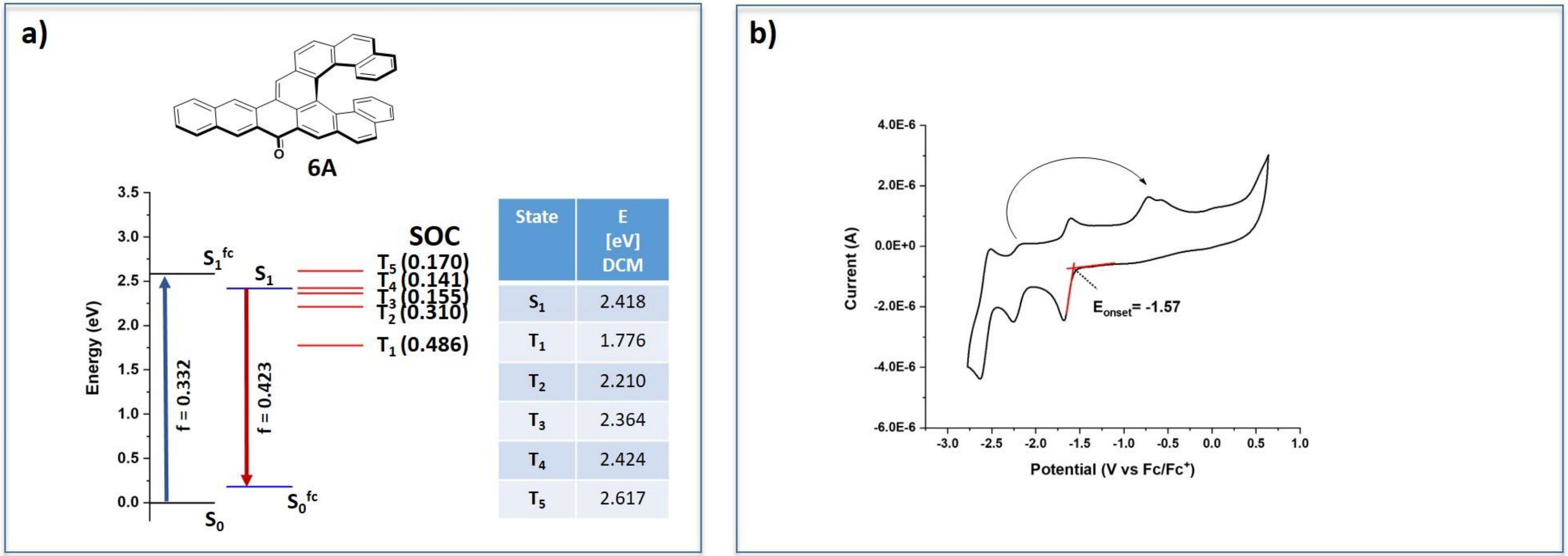

$\lambda_{(max)}$ [nm] DCM	$\lambda_{(em)}$ $\lambda_{(exc)}$[a] [nm] DCM	g_{lum}[b] (10^{-3}) DCM	$[\alpha]_D^{20}$ DCM	Φ_{PL}[c] (%) hexane/DCM/ACN	t_{fl} [ns] hexane/DCM/ACN	k_r[d] [10^7 s^{-1}] hexane/DCM/ACN	k_{nr}[e] [10^7 s^{-1}] Hexane/DCM/ACN	E_{HOMO}[f] [eV]	E_{LUMO}[g] [eV]	$E_{(0,0)}$[h] [eV] DCM (hexane)	$E_{(0,0)}$[i] [eV]	$E_{T1\text{-}S0}$[j] [eV]
494	546(303)	+0.82<br>-0.80	+2014<br>-1945	7.9/11.2/10.8	2.71/2.45/2.95	2.9/4.6/3.7	33.9/36.2/30.2	-5.78	-3.23	2.41<br>(2.53)	2.42	2.00

a) Wavelength of excitation b) Measured in dichloromethane (c ≈ 1 x 10^{-5} M) c) Measured at room temperature (c ≈ 1 x 10^{-6} M) d) radiative rate constant calculated as $k_r = \Phi_{PL}/t_{fl}$ e) nonradiative decay rate constant calculated as $\Phi_{PL} = k_r/(k_r+k_{nr})$ f) Calculated as $E_{HOMO} = E_{LUMO} - E(0,0)$ g) Calculated using the equation $E_{LUMO} = -[E'_{red/onset} + 4.8]$ referenced against Fc/Fc^+. h) Determined from the intersection of the absorption and emission curves and using equation $E_g = hc/\lambda$ i) Calculated by TDDFT M06/6-31G(d,p) method j) determined from the phosphorescence λ_{onset}

a) Energy-level diagram calculated by TDDFT M06/6-31G(d,p) method in ACN. **b)** CV voltammogram vs Fc/Fc^+ redox couple in acetonitrile (ACN) with a supporting electrolyte $[Bu_4N][PF_6]$ (0.1M), at a scan rate 0.1 V s^{-1}, the second irreversible reduction process lead to a product(s) with oxidation peak located at -0.6 V.

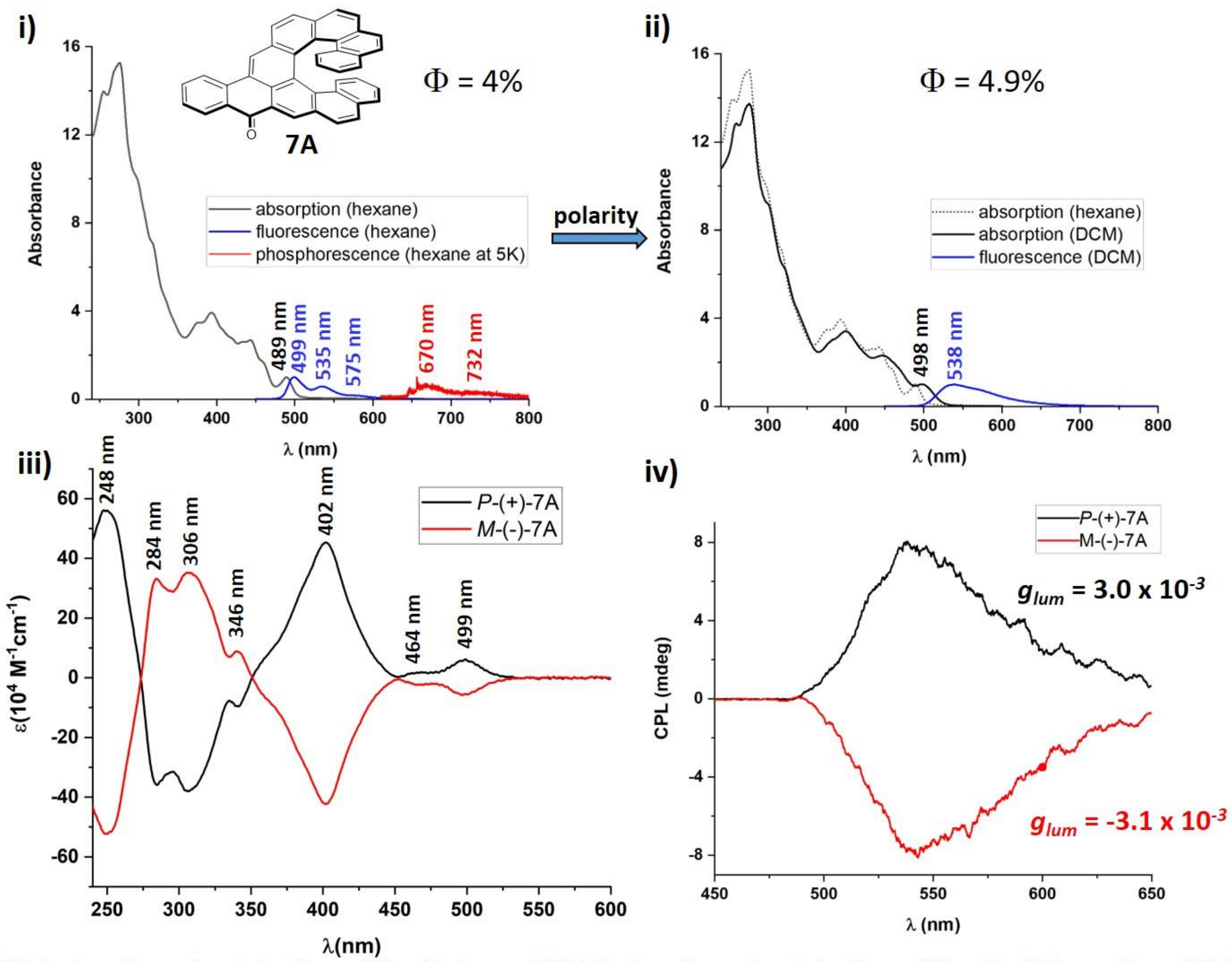

P-(+) Peak at [nm]	g_{abs}
499	7.0*10^{-3}
464	1.0*10^{-3}
402	11.0*10^{-3}
346	-1.5*10^{-3}
306	-3.5*10^{-3}
284	-2.3*10^{-3}
248	4.0*10^{-3}

i) UV-vis absorption and emission ($\lambda_{(exc)}$ = 303nm) in hexane **ii)** UV-vis absorption and emission ($\lambda_{(exc)}$ = 303nm) in dichloromethane (DCM) **iii)** CD spectra collected in dichloromethane (DCM) **iv)** CPEL spectra and glum values collected in DCM

a)

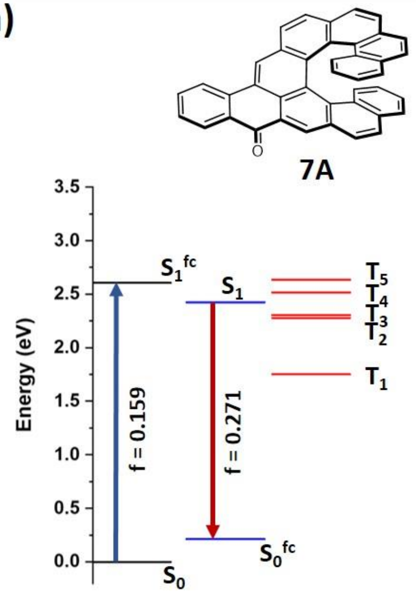

7A

State	E [eV] DCM
S_1	2.424
T_1	1.750
T_2	2.278
T_3	2.303
T_4	2.515
T_5	2.634

b)

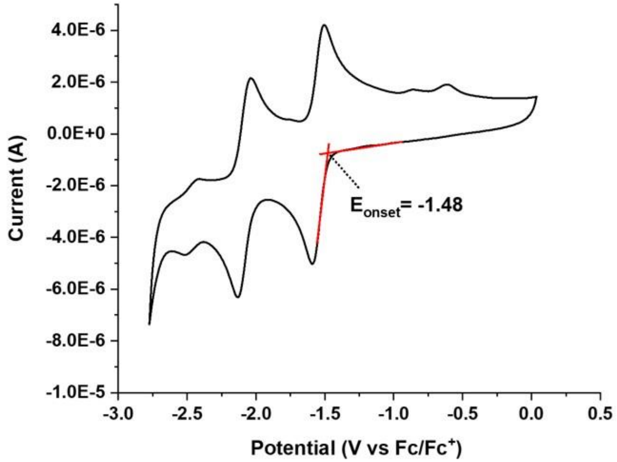

$\lambda_{(max)}$ [nm] DCM	$\lambda_{(em)}$ $\lambda_{(exc)}$[a] [nm] DCM	g_{lum}[b] (10^{-3}) DCM	$[\alpha]_D^{20}$ DCM	Φ_{PL}[c] (%) hexane/DCM/ACN	t_{fl} [ns] hexane/DCM/ACN	k_r[d] [10^7 s^{-1}] hexane/DCM/ACN	k_{nr}[e] [10^7 s^{-1}] Hexane/DCM/ACN	E_{HOMO}[f] [eV]	E_{LUMO}[g] [eV]	$E_{(0,0)}$[h] [eV] DCM (hexane)	$E_{(0,0)}$[i] [eV]	E_{T1-S0}[j] [eV]
498	538(303)	+3.0<br>-3.1	+4789<br>-4633	4.0/4.9/6.8	2.83/3.34/3.53	1.4/1.5/1.9	33.9/28.5/26.4	-5.74	-3.23	2.41<br>(2.51)	2.42	1.93

a) Wavelength of excitation b) Measured in dichloromethane (c ≈ 1 x 10^{-5} M) c) Measured at room temperature (c ≈ 1 x 10^{-6} M) d) radiative rate constant calculated as $k_r = \Phi_{PL}/t_{fl}$ e) nonradiative decay rate constant calculated as $\Phi_{PL} = k_r/(k_r+k_{nr})$ f) Calculated as $E_{HOMO} = E_{LUMO} - E(0,0)$ g) Calculated using the equation $E_{LUMO} = -[E'_{red/onset} + 4.8]$ referenced against Fc/Fc^+. h) Determined from the intersection of the absorption and emission curves and using equation $E_g = hc/\lambda$ i) Calculated by TDDFT M06/6-31G(d,p) method j) determined from the phosphorescence λ_{onset}

a) Energy-level diagram calculated by TDDFT M06/6-31G(d,p) method in ACN. **b)** CV voltammogram vs Fc/Fc^+ redox couple in acetonitrile (ACN) with a supporting electrolyte $[Bu_4N][PF_6]$ (0.1M), at a scan rate 0.1 V s^{-1}

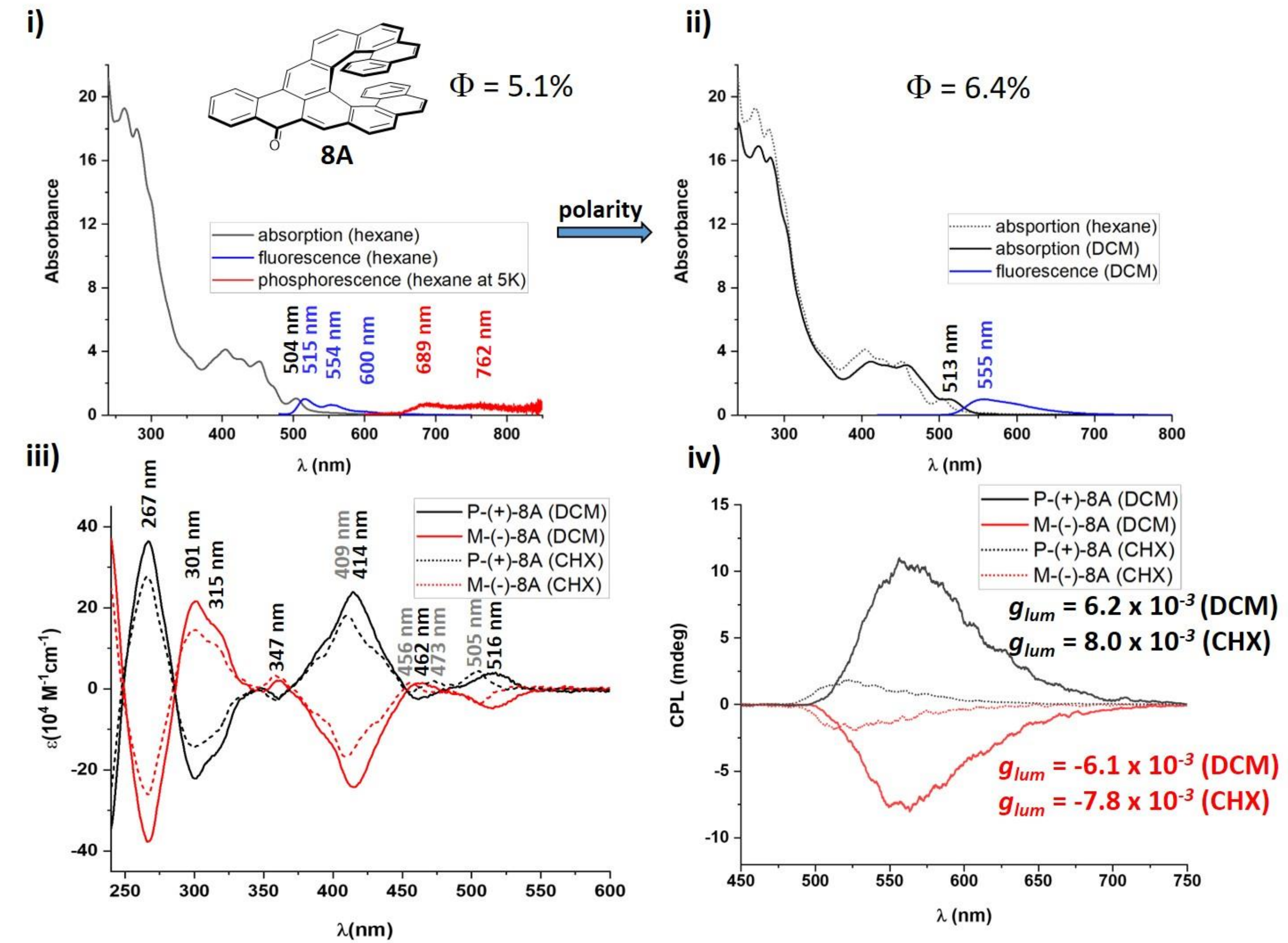

***P*-(+) Peak at [nm] DCM (CHX)**	**g_{abs} DCM (CHX)**
516 (505)	8.6*10^{-3} (9.3*10^{-3})
462 (456)	-0.7*10^{-3} (-0.75*10^{-3})
414 (409)	13.6 *10^{-3} (11.7*10^{-3})
347 (347)	-0.4*10^{-3} (0.2*10^{-3})
315 (315)	3.7*10^{-3} (3.6*10^{-3})
301 (301)	3.2*10^{-3} (3.0*10^{-3})
267 (267)	-4.0*10^{-3} (-3.7*10^{-3})

i) UV-vis absorption and emission ($\lambda_{(exc)}$ = 303nm) in hexane **ii)** UV-vis absorption and emission ($\lambda_{(exc)}$ = 303nm) in dichloromethane (DCM) **iii)** CD spectra collected in dichloromethane (DCM) **iv)** CPEL spectra and glum values collected in DCM and cyclohexane (CHX)

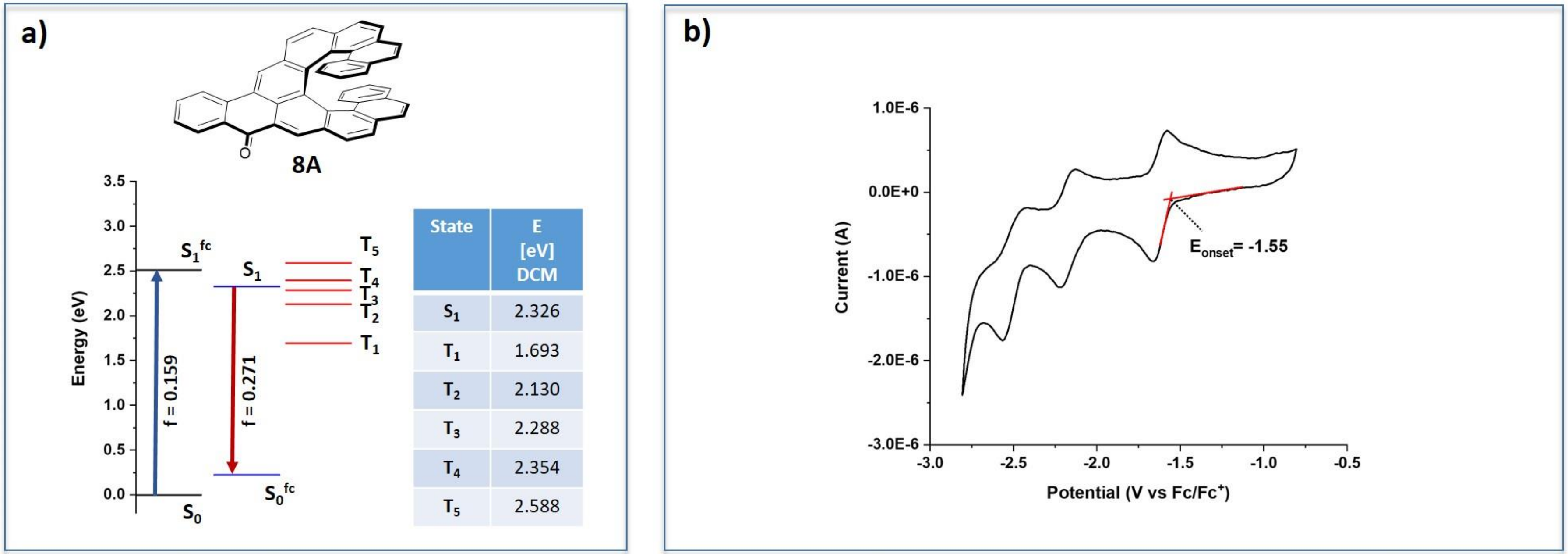

$\lambda_{(max)}$ [nm] DCM	$\lambda_{(em)}$ $\lambda_{(exc)}$ [a] [nm] DCM	g_{lum} [b] (10^{-3}) DCM (hexane)	$[\alpha]_D^{20}$ DCM	Φ_{PL} [c] (%) hexane/DCM/ACN	t_{fl} [ns] hexane/DCM/ACN	k_r [d] [10^7 s^{-1}] hexane/DCM/ACN	k_{nr} [e] [10^7 s^{-1}] Hexane/DCM/ACN	E_{HOMO} [f] [eV]	E_{LUMO} [g] [eV]	$E_{(0,0)}$ [h] [eV] DCM (hexane)	$E_{(0,0)}$ [i] [eV]	E_{T1-S0} [j] [eV]
513	555(303)	+6.2 (8.0)<br>-6.1 (7.8)	+6015<br>-6297	5.1/6.4/4.9	3.31/3.09/3.16	1.54/2.07/1.55	28.7/30.3/30.1	-5.68	-3.25	2.33<br>(2.43)	2.33	1.90

a) Wavelength of excitation b) Measured in dichloromethane (c ≈ 1 x 10^{-5} M) c) Measured at room temperature (c ≈ 1 x 10^{-6} M) d) radiative rate constant calculated as $k_r = \Phi_{PL}/t_{fl}$ e) nonradiative decay rate constant calculated as $\Phi_{PL} = k_r/(k_r+k_{nr})$ f) Calculated as $E_{HOMO} = E_{LUMO} - E(0,0)$ g) Calculated using the equation $E_{LUMO} = -[E'_{red/onset} + 4.8]$ referenced against Fc/Fc^+. h) Determined from the intersection of the absorption and emission curves and using equation $E_g = hc/\lambda$ i) Calculated by TDDFT M06/6-31G(d,p) method j) determined from the phosphorescence λ_{onset}

a) Energy-level diagram calculated by TDDFT M06/6-31G(d,p) method in ACN. **b)** CV voltammogram vs Fc/Fc^+ redox couple in acetonitrile (ACN) with a supporting electrolyte $[Bu_4N][PF_6]$ (0.1M), at a scan rate 0.1 V s^{-1}

Table S1: photophysical data

aceno[6]helicenones			aceno[7]helicenones			aceno[8]helicenone	aceno[9]helicenone
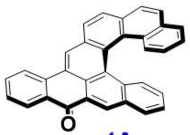 1A	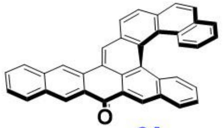 2A	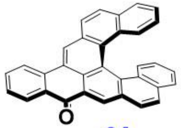 3A	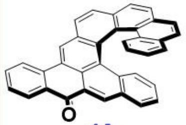 4A	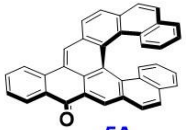 5A	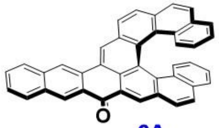 6A	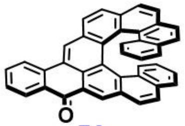 7A	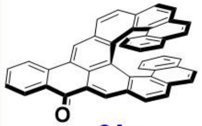 8A

Helicene	$\lambda_{(onset)}$ [nm] hexane	$\lambda_{(em)}$ $\lambda_{(exc)}$ [a] [nm] DCM	g_{lum} [b] (10^{-3}) DCM (hexane)	$[\alpha]_D^{20}$ DCM	Φ_{PL} [c] (%) hexane/DCM/ACN	t_{fl} [ns] hexane/DCM/ACN	k_r [d] [10^7 s^{-1}] hexane/DCM/ACN	k_{nr} [e] [10^7 s^{-1}] Hexane/DCM/ACN	E_{HOMO} [f] [eV]	E_{LUMO} [g] [eV]	$E_{(0,0)}$ [h] [eV] DCM (hexane)	$E_{(0,0)}$ [i] [eV]	$E_{T1\text{-}S0}$ [j] [eV]
1A	469	513(303)	+1.7<br>-1.5	+2147<br>-2167	3.6/12.5/11.4	2.34/4.0/4.25	1.5/3.1/2.7	41.2/21.9/20.8	-5.89	-3.21	2.55<br>(2.68)	2.59	2.08
2A	475	534(303)	+0.58<br>-0.52	+770<br>-748	5.0/8.3/7.6	1.93/2.71/2.5	2.7/3.0/3.0	50.5/33.9/37	-5.88	-3.23	2.49<br>(2.65)	2.51	2.09
3A	467	494(303)	+1.2<br>-1.0	+1094<br>-1031	1.3/8.5/7.8	0.79/2.71/2.89	1.6/3.1/2.7	125.2/33.8/31.9	-5.95	-3.27	2.60<br>(2.68)	2.58	2.00
4A	481	520(336)	+3.0<br>-2.9	+3493<br>-3526	3.4/8.3/7.6	2.76/3.68/3.85	1.21/2.25/1.97	35.0/24.9/24.0	-5.82	-3.2	2.51<br>(2.62)	2.49	2.00
5A	493	530(303)	+2.0<br>-2.0	+3620<br>-3672	3.3/6.6/5.8	2.56/3.42/4.09	1.3/1.9/1.4	37.8/27.3/23.0	-5.78	-3.23	2.45<br>(2.55)	2.46	1.96
6A	497	546(303)	+0.82<br>-0.80	+2014<br>-1945	7.9/11.2/10.8	2.71/2.45/2.95	2.9/4.6/3.7	33.9/36.2/30.2	-5.78	-3.23	2.41<br>(2.53)	2.42	2.00
7A	501	538(303)	+3.0<br>-3.1	+4789<br>-4633	4.0/4.9/6.8	2.83/3.34/3.53	1.4/1.5/1.9	33.9/28.5/26.4	-5.74	-3.23	2.41<br>(2.51)	2.42	1.93
8A	517	555(303)	+6.2 (-7.8)<br>-6.1 (8.0)	+6015<br>-6297	5.1/6.4/4.9	3.31/3.09/3.16	1.54/2.07/1.55	28.7/30.3/30.1	-5.68	-3.25	2.33<br>(2.43)	2.33	1.90

a) Wavelength of excitation b) Measured in dichloromethane (c ≈ 1 x 10^{-5} M) c) Measured at room temperature (c ≈ 1 x 10^{-6} M) d) radiative rate constant calculated as $k_r = \Phi_{PL}/t_{fl}$ e) nonradiative decay rate constant calculated as $\Phi_{PL} = k_r/(k_r+k_{nr})$ f) Calculated as $E_{HOMO} = E_{LUMO} - E(0,0)$ g) Calculated using the equation $E_{LUMO} = -[E'_{red/onset} + 4.8]$ referenced against Fc/Fc^+. h) Determined from the intersection of the absorption and emission curves and using equation $E_g = hc/\lambda$ i) Calculated by TDDFT M06/6-31G(d,p) method j) determined from the phosphorescence λ_{onset}

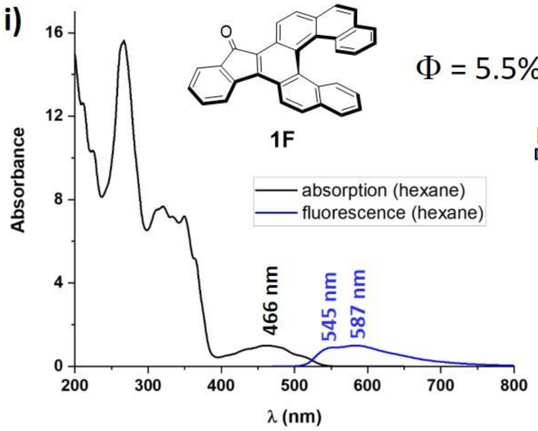

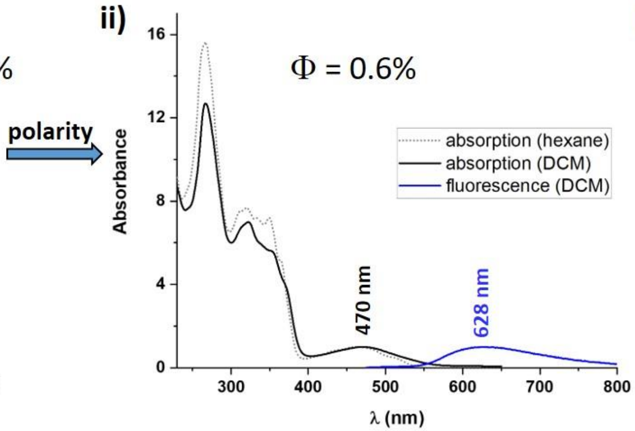

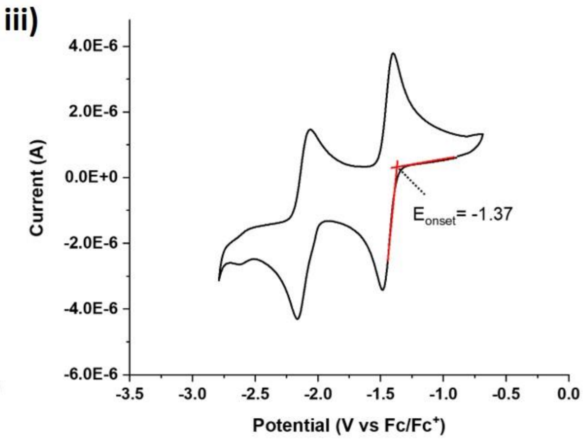

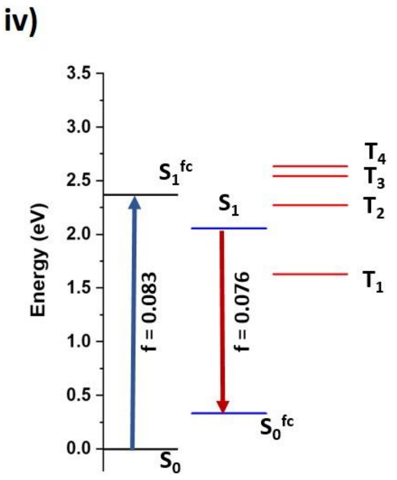

$\lambda_{(onset)}$ [nm] DCM	$\lambda_{(em)}$ $\lambda_{(exc)}$ [a] [nm] DCM	Φ_{PL} [b] (%) hexane/DCM/ACN	t_{fl} [ns] hexane/DCM/ACN	k_r [c] [10^7 s^{-1}] hexane/DCM/ACN	k_{nr} [d] [10^7 s^{-1}] Hexane/DCM/ACN	E_{HOMO} [e] [eV]	E_{LUMO} [f] [eV]	$E_{(0,0)}$ [g] [eV] Hexane (DCM)	$E_{(0,0)}$ [h] [eV]
541	628(303)	5.5/0.6/0.6	6.38/1.14/1.15	0.86/0.53/0.52	14.8/87.2/86.4	-5.8	-3.43	2.37 (2.24)	2.05

State	E [eV] DCM
S_1	2.05
T_1	1.630
T_2	2.271
T_3	2.541
T_4	2.633

a) wavelength of excitation b) Measured at room temperature ($c \approx 1 \times 10^{-6}$ M) c) radiative rate constant calculated as $k_r = \Phi_{PL}/t_{fl}$ d) nonradiative decay rate constant calculated as $\Phi_{PL} = k_r/(k_r+k_{nr})$ e) Calculated as $E_{HOMO} = E_{LUMO} - E(0,0)$ f) Calculated using the equation $E_{LUMO} = -[E'_{red/onset} + 4.8]$ referenced against Fc/Fc^+ g) Determined from the intersection of the absorption and emission curves and using equation $E_g = hc/\lambda$ h) Calculated by TDDFT M06/6-31G(d,p) method

i) UV-vis absorption and emission ($\lambda_{(exc)}$ = 303nm) in hexane **ii)** UV-vis absorption and emission ($\lambda_{(exc)}$ = 303nm) in dichloromethane (DCM) **iii)** CV voltammogram vs Fc/Fc^+ redox couple in dimethylformamide (DMF) with a supporting electrolyte $[Bu_4N][PF_6]$ (0.1M), at a scan rate 0.1 V s^{-1} **iv)** Energy-level diagram calculated by TDDFT M06/6-31G(d,p) method in ACN.

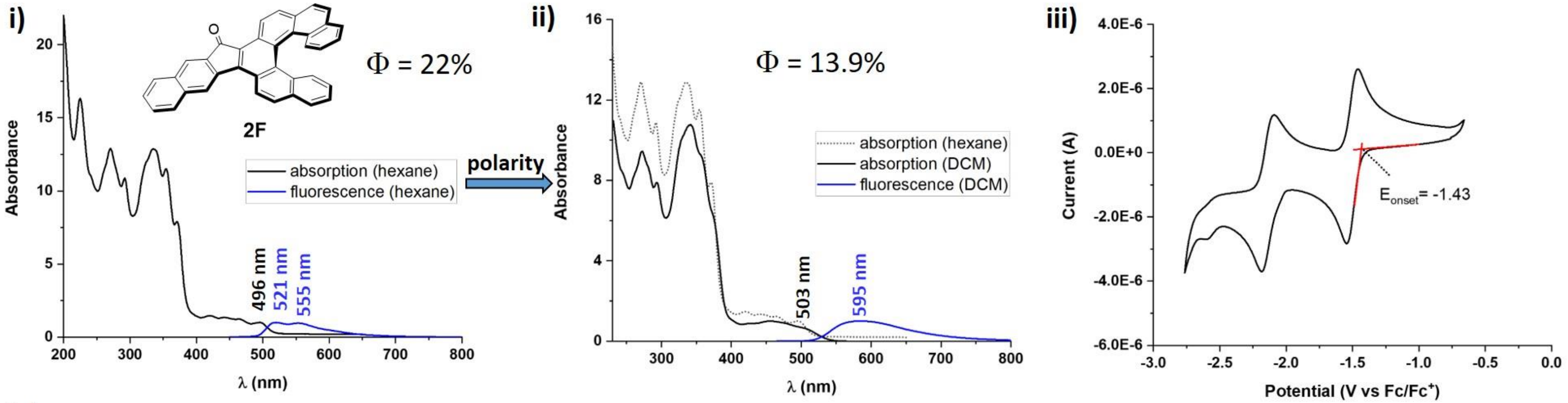

iv)

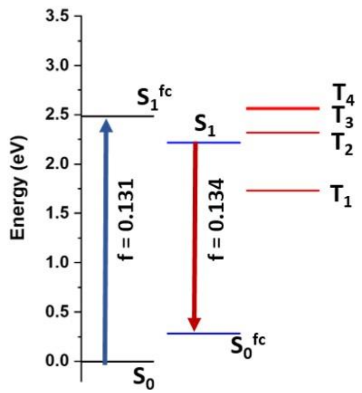

$\lambda_{(onset)}$ [nm] DCM	$\lambda_{(em)}$ $\lambda_{(exc)}$ [a] [nm] DCM	Φ_{PL} [b] (%) hexane/DCM/ACN	t_{fl} [ns] hexane/DCM/ACN	k_r [c] [10^7 s^{-1}] hexane/DCM/ACN	k_{nr} [d] [10^7 s^{-1}] Hexane/DCM/ACN	E_{HOMO} [e] [eV]	E_{LUMO} [f] [eV]	$E_{(0,0)}$ [g] [eV] Hexane (DCM)	$E_{(0,0)}$ [h] [eV]
541	595(336)	22.0/13.9/13.5	10.2/8.96/8.28	2.2/1.5/1.6	7.6/9.6/10.4	-5.87	-3.43	2.44 (2.34)	2.22

State	E [eV] DCM
S_1	2.216
T_1	1.732
T_2	2.319
T_3	2.555
T_4	2.569

a) wavelength of excitation b) Measured at room temperature (c ≈ 1 x 10^{-6} M) c) radiative rate constant calculated as $k_r = \Phi_{PL}/t_{fl}$ d) nonradiative decay rate constant calculated as $\Phi_{PL} = k_r/(k_r+k_{nr})$ e) Calculated as $E_{HOMO} = E_{LUMO} - E(0,0)$ f) Calculated using the equation $E_{LUMO} = -[E'_{red/onset} + 4.8]$ referenced against Fc/Fc^+ g) Determined from the intersection of the absorption and emission curves and using equation $E_g = hc/\lambda$ h) Calculated by TDDFT M06/6-31G(d,p) method

i) UV-vis absorption and emission ($\lambda_{(exc)}$ = 303nm) in hexane **ii)** UV-vis absorption and emission ($\lambda_{(exc)}$ = 303nm) in dichloromethane (DCM) **iii)** CV voltammogram vs Fc/Fc^+ redox couple in dimethylformamide (DMF) with a supporting electrolyte $[Bu_4N][PF_6]$ (0.1M), at a scan rate 0.1 V s^{-1} **iv)** Energy-level diagram calculated by TDDFT M06/6-31G(d,p) method in ACN.

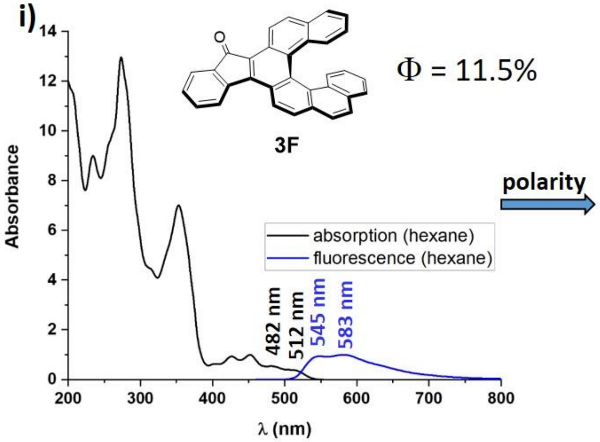

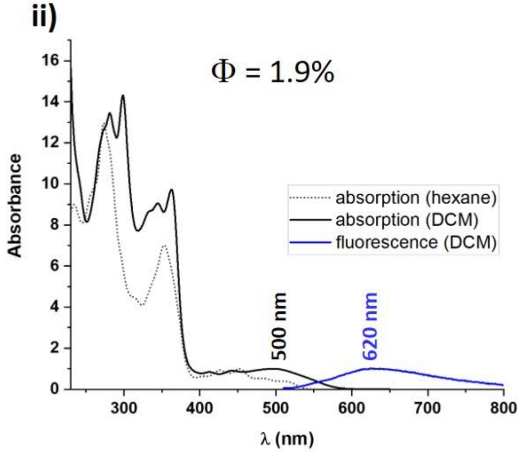

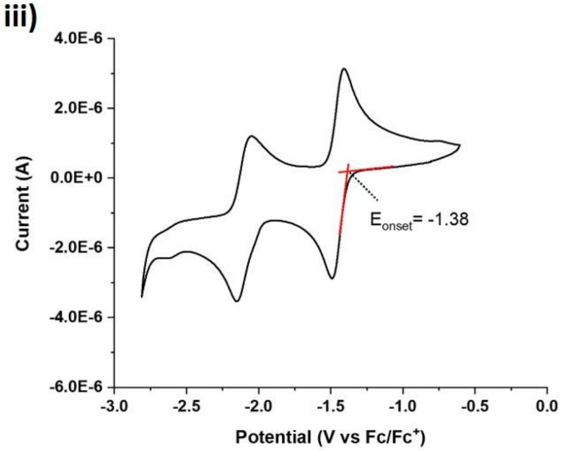

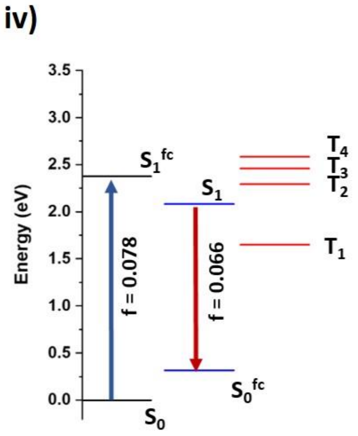

$\lambda_{(onset)}$ [nm] DCM	$\lambda_{(em)}$ $\lambda_{(exc)}$ [a] [nm] DCM	Φ_{PL} [b] (%) hexane/DCM/ACN	t_{fl} [ns] hexane/DCM/ACN	k_r [c] [10^7 s^{-1}] hexane/DCM/ACN	k_{nr} [d] [10^7 s^{-1}] Hexane/DCM/ACN	E_{HOMO} [e] [eV]	E_{LUMO} [f] [eV]	$E_{(0,0)}$ [g] [eV] Hexane (DCM)	$E_{(0,0)}$ [h] [eV]
580	620(303)	11.5/1.9/1.85	9.21/1.9/2.0	1.2/1.1/0.9	9.8/57.4/49.1	-5.79	-3.42	2.37 (2.27)	2.08

State	E [eV] DCM
S_1	2.081
T_1	1.650
T_2	2.296
T_3	2.458
T_4	2.583

a) wavelength of excitation b) Measured at room temperature (c ≈ 1 x 10^{-6} M) c) radiative rate constant calculated as $k_r = \Phi_{PL}/t_{fl}$ d) nonradiative decay rate constant calculated as $\Phi_{PL} = k_r/(k_r+k_{nr})$ e) Calculated as $E_{HOMO} = E_{LUMO} - E(0,0)$ f) Calculated using the equation $E_{LUMO} = -[E'_{red/onset} + 4.8]$ referenced against Fc/Fc+ g) Determined from the intersection of the absorption and emission curves and using equation $E_g = hc/\lambda$ h) Calculated by TDDFT M06/6-31G(d,p) method

i) UV-vis absorption and emission ($\lambda_{(exc)}$ = 303nm) in hexane **ii)** UV-vis absorption and emission ($\lambda_{(exc)}$ = 303nm) in dichloromethane (DCM) **iii)** CV voltammogram vs Fc/Fc+ redox couple in dimethylformamide (DMF) with a supporting electrolyte $[Bu_4N][PF_6]$ (0.1M), at a scan rate 0.1 V s^{-1} **iv)** Energy-level diagram calculated by TDDFT M06/6-31G(d,p) method in ACN.

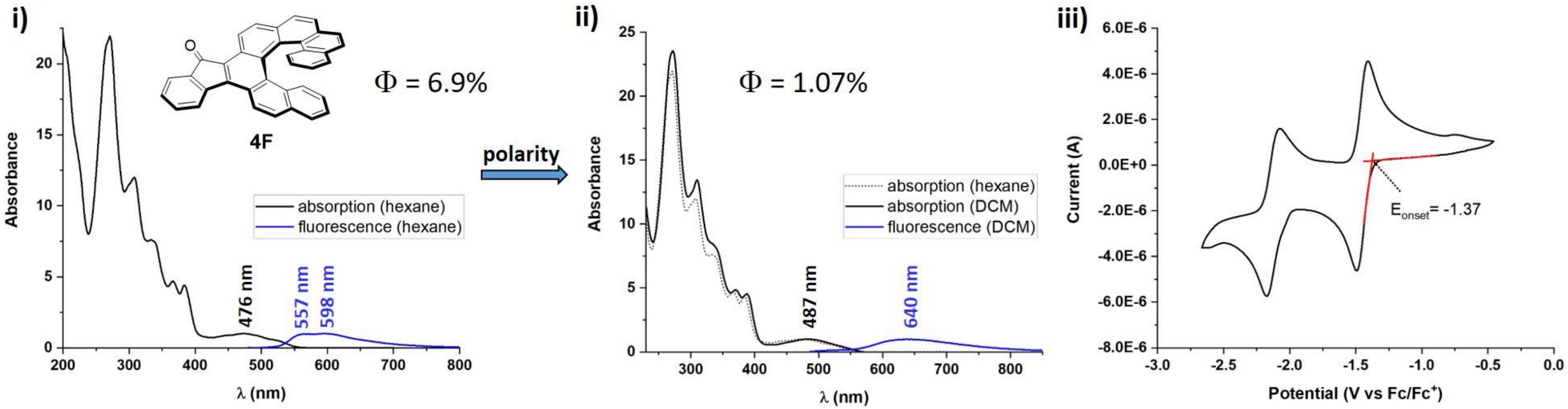

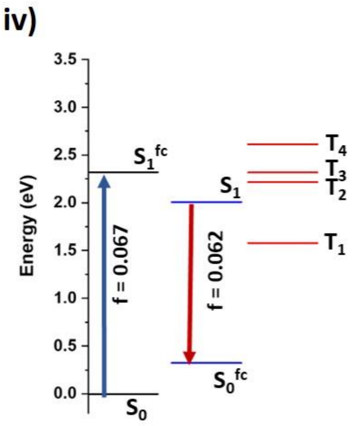

$\lambda_{(onset)}$ [nm] DCM	$\lambda_{(em)}$ $\lambda_{(exc)}$[a] [nm] DCM	Φ_{PL}[b] (%) hexane/DCM/ACN	t_{fl} [ns] hexane/DCM/ACN	k_r[c] [10^7 s^{-1}] hexane/DCM/ACN	k_{nr}[d] [10^7 s^{-1}] Hexane/DCM/ACN	E_{HOMO}[e] [eV]	E_{LUMO}[f] [eV]	$E_{(0,0)}$[g] [eV] Hexane (DCM)	$E_{(0,0)}$[h] [eV]
570	640(303)	6.9/1.07/0.76	6.48/9.1/8.4	1.07/0.12/0.09	14.4/10.9/11.8	-5.68	-3.37	2.31 (2.23)	2.01

State	E [eV] DCM
S_1	2.006
T_1	1.580
T_2	2.215
T_3	2.317
T_4	2.612

a) wavelength of excitation b) Measured at room temperature (c ≈ 1 x 10^{-6} M) c) radiative rate constant calculated as $k_r = \Phi_{PL}/t_{fl}$ d) nonradiative decay rate constant calculated as $\Phi_{PL} = k_r/(k_r+k_{nr})$ e) Calculated as $E_{HOMO} = E_{LUMO} - E(0,0)$ f) Calculated using the equation $E_{LUMO} = -[E'_{red/onset} + 4.8]$ referenced against Fc/Fc$^+$ g) Determined from the intersection of the absorption and emission curves and using equation $E_g = hc/\lambda$ h) Calculated by TDDFT M06/6-31G(d,p) method

i) UV-vis absorption and emission ($\lambda_{(exc)}$ = 303nm) in hexane **ii)** UV-vis absorption and emission ($\lambda_{(exc)}$ = 303nm) in dichloromethane (DCM) **iii)** CV voltammogram vs Fc/Fc$^+$ redox couple in dimethylformamide (DMF) with a supporting electrolyte $[Bu_4N][PF_6]$ (0.1M), at a scan rate 0.1 V s^{-1} **iv)** Energy-level diagram calculated by TDDFT M06/6-31G(d,p) method in ACN.

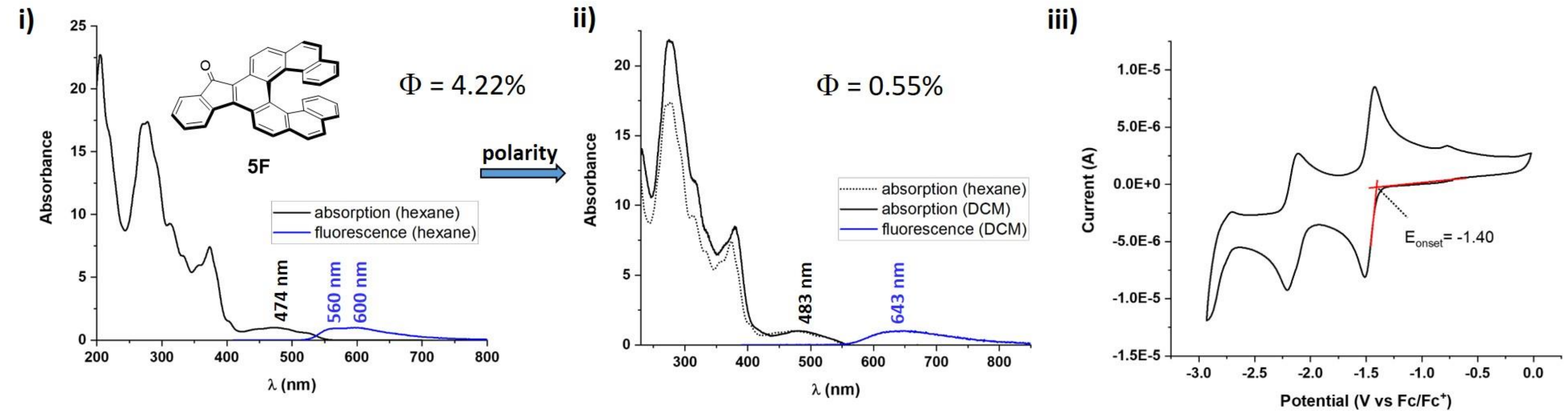

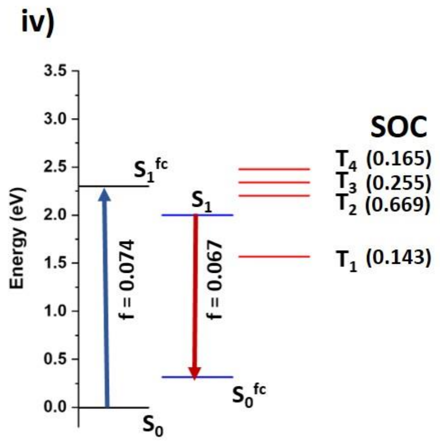

$\lambda_{(onset)}$ [nm] DCM	$\lambda_{(em)}$ $\lambda_{(exc)}$ [a] [nm] DCM	Φ_{PL} [b] (%) hexane/DCM/ACN	t_{fl} [ns] hexane/DCM/ACN	k_r [c] [10^7 s^{-1}] hexane/DCM/ACN	k_{nr} [d] [10^7 s^{-1}] Hexane/DCM/ACN	E_{HOMO} [e] [eV]	E_{LUMO} [f] [eV]	$E_{(0,0)}$ [g] [eV] Hexane (DCM)	$E_{(0,0)}$ [h] [eV]
573	643(336)	4.22/0.55/0.61	6.38/1.15/1.16	0.66/0.47/0.53	15/85.7/86.4	-5.71	-3.4	2.31 (2.23)	2.00

State	E [eV] DCM
S_1	2.001
T_1	1.568
T_2	2.205
T_3	2.341
T_4	2.479

a) wavelength of excitation b) Measured at room temperature (c ≈ 1 x 10^{-6} M) c) radiative rate constant calculated as $k_r = \Phi_{PL}/t_{fl}$ d) nonradiative decay rate constant calculated as $\Phi_{PL} = k_r/(k_r+k_{nr})$ e) Calculated as $E_{HOMO} = E_{LUMO} - E(0,0)$ f) Calculated using the equation $E_{LUMO} = -[E'_{red/onset} + 4.8]$ referenced against Fc/Fc⁺ g) Determined from the intersection of the absorption and emission curves and using equation $E_g = hc/\lambda$ h) Calculated by TDDFT M06/6-31G(d,p) method

i) UV-vis absorption and emission ($\lambda_{(exc)}$ = 303nm) in hexane **ii)** UV-vis absorption and emission ($\lambda_{(exc)}$ = 303nm) in dichloromethane (DCM) **iii)** CV voltammogram vs Fc/Fc⁺ redox couple in dimethylformamide (DMF) with a supporting electrolyte $[Bu_4N][PF_6]$ (0.1M), at a scan rate 0.1 V s^{-1} **iv)** Energy-level diagram calculated by TDDFT M06/6-31G(d,p) method in ACN.

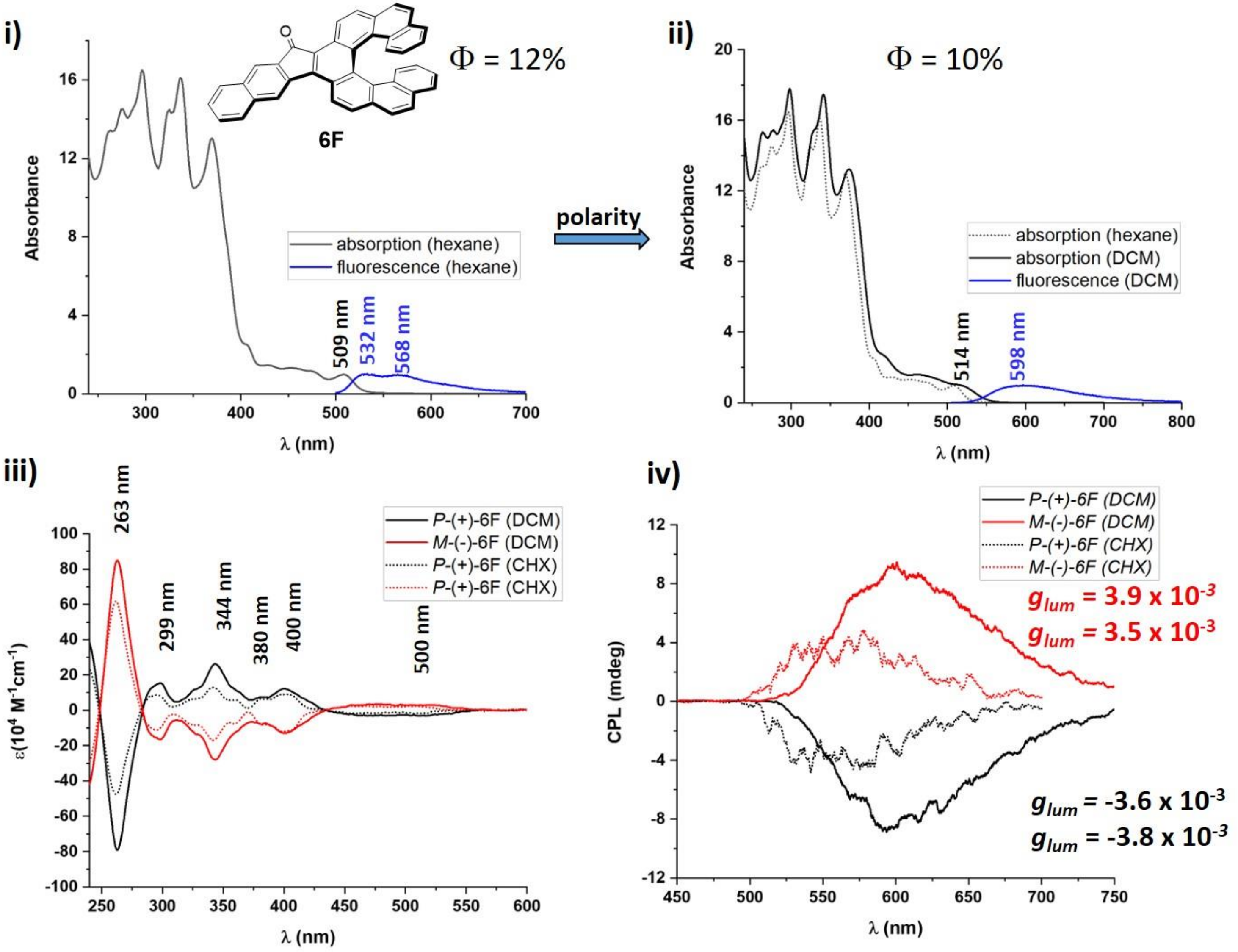

P-(+) Peak at [nm]	G_{abs} DCM (CHX)
500	$-2.5*10^{-3}$ ($-2.8*10^{-3}$)
400	$3.1*10^{-3}$ ($5.8*10^{-3}$)
380	$0.7*10^{-3}$ ($1.1*10^{-3}$)
344	$1.8*10^{-3}$ ($1.6*10^{-3}$)
299	$0.8*10^{-3}$ ($0.8*10^{-3}$)
263	$-5.7*10^{-3}$ ($-5.9*10^{-3}$)

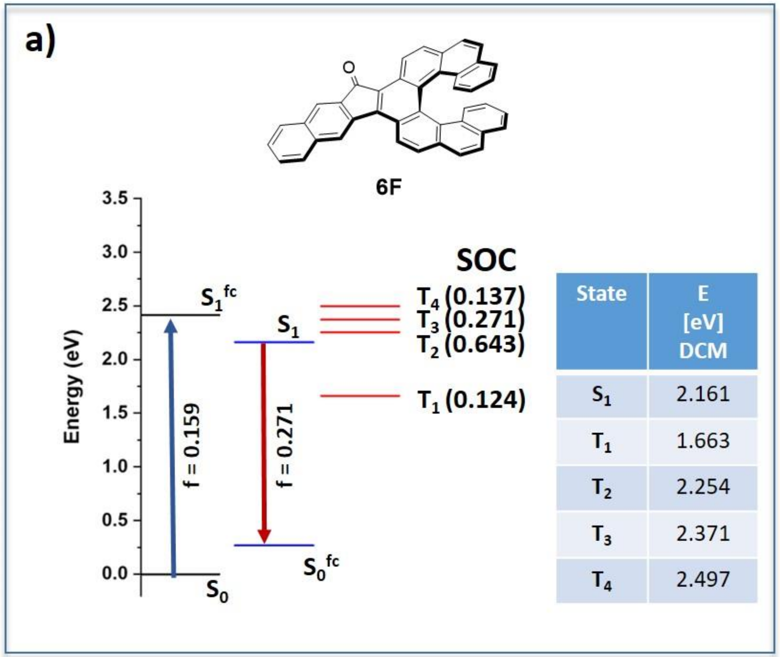

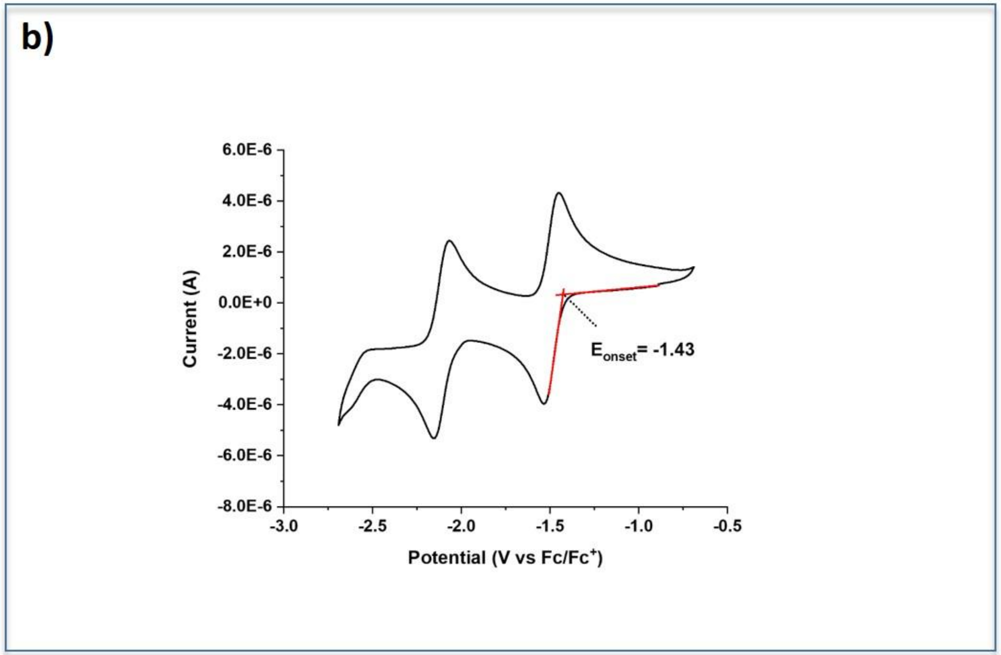

$\lambda_{(onset)}$ [a] [nm] DCM	$\lambda_{(em)}$ $\lambda_{(exc)}$ [b] [nm] DCM	g_{lum} [c] (10^{-3}) DCM	$[\alpha]_D^{20}$ [a]	Φ_{PL} [e] (%) hexane/DCM/ACN	t_{fl} [ns] hexane/DCM/ACN	k_r [f] [10^7 s^{-1}] hexane/DCM/ACN	k_{nr} [g] [10^7 s^{-1}] Hexane/DCM/ACN	E_{HOMO} [h] [eV]	E_{LUMO} [i] [eV]	$E_{(0,0)}$ [j] [eV] Hexane (DCM)	$E_{(0,0)}$ [k] [eV]
555	598(336)	+3.9<br>-3.8	+434<br>-391	12.0/10.0/8.4	7.1/6.9/6.3	1.7/1.45/1.32	12.5/13/14.5	-5.76	-3.37	2.39<br>(2.27)	2.16

a) Measured in dichloromethane. b) wavelength of excitation c) Measured in dichloromethane (c ≈ 1 x 10^{-5} M) e) Measured at room temperature (c ≈ 1 x 10^{-6} M) f) radiative rate constant calculated as $k_r = \Phi_{PL}/t_{fl}$ g) nonradiative decay rate constant calculated as $\Phi_{PL} = k_r/(k_r+k_{nr})$ h) Calculated as $E_{HOMO} = E_{LUMO} - E(0,0)$ i) Calculated using the equation $E_{LUMO} = -[E'_{red/onset} + 4.8]$ referenced against Fc/Fc+. j) Determined from the intersection of the absorption and emission curves and using equation $E_g = hc/\lambda$ k) Calculated by TDDFT M06/6-31G(d,p) method l) determined from the phosphorescence λ_{onset}

a) Energy-level diagram calculated by TDDFT M06/6-31G(d,p) method in ACN. **b)** CV voltammogram vs Fc/Fc+ redox couple in dimethylformamide (DMF) with a supporting electrolyte $[Bu_4N][PF_6]$ (0.1M), at a scan rate 0.1 V s^{-1}

i)

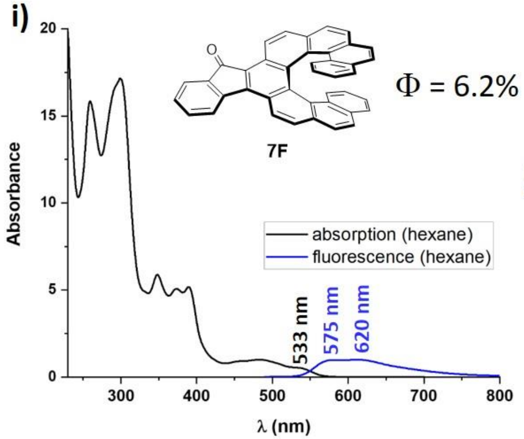

polarity

ii)

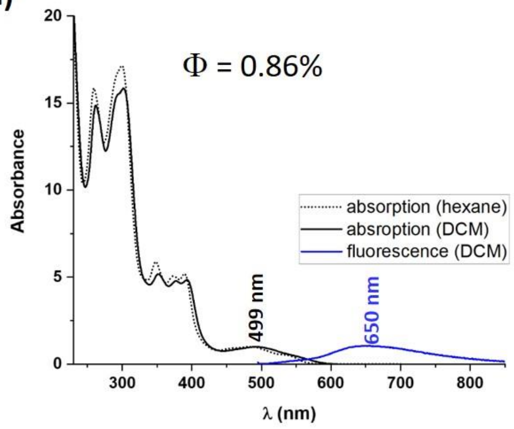

iii)

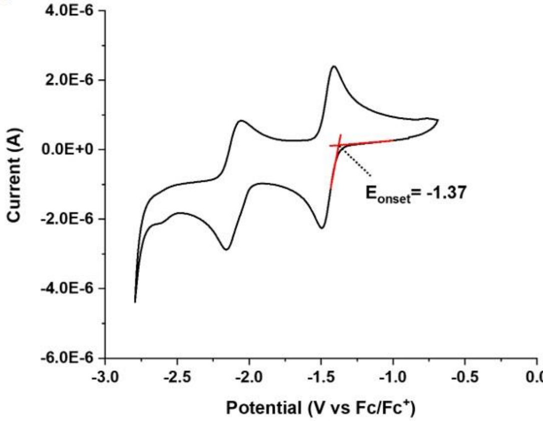

iv)

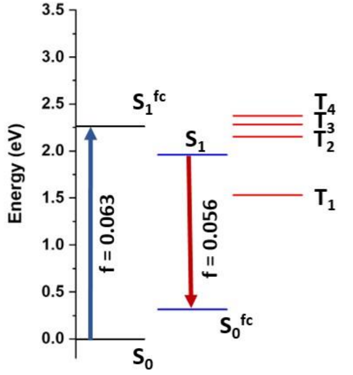

$\lambda_{(onset)}$ [nm] DCM	$\lambda_{(em)}$ $\lambda_{(exc)}$ [a] [nm] DCM	Φ_{PL} [b] (%) hexane/DCM/ACN	t_{fl} [ns] hexane/DCM/ACN	k_r [c] [10^7 s^{-1}] hexane/DCM/ACN	k_{nr} [d] [10^7 s^{-1}] Hexane/DCM/ACN	E_{HOMO} [e] [eV]	E_{LUMO} [f] [eV]	$E_{(0,0)}$ [g] [eV] Hexane (DCM)	$E_{(0,0)}$ [h] [eV]
596	650(303)	6.2/0.86/0.72	6.23/0.95/0.94	1/0.91/0.76	15/105/105	-5.69	-3.43	2.26 (2.19)	1.96

State	E [eV] DCM
S_1	1.96
T_1	1.531
T_2	2.153
T_3	2.280
T_4	2.373

a) wavelength of excitation b) Measured at room temperature (c ≈ 1 x 10^{-6} M) c) radiative rate constant calculated as $k_r = \Phi_{PL}/t_{fl}$ d) nonradiative decay rate constant calculated as $\Phi_{PL} = k_r/(k_r+k_{nr})$ e) Calculated as $E_{HOMO} = E_{LUMO} - E(0,0)$ f) Calculated using the equation $E_{LUMO} = -[E'_{red/onset} + 4.8]$ referenced against Fc/Fc^+ g) Determined from the intersection of the absorption and emission curves and using equation $E_g = hc/\lambda$ h) Calculated by TDDFT M06/6-31G(d,p) method

i) UV-vis absorption and emission ($\lambda_{(exc)}$ = 303nm) in hexane **ii)** UV-vis absorption and emission ($\lambda_{(exc)}$ = 303nm) in dichloromethane (DCM) **iii)** CV voltammogram vs Fc/Fc^+ redox couple in dimethylformamide (DMF) with a supporting electrolyte $[Bu_4N][PF_6]$ (0.1M), at a scan rate 0.1 V s^{-1} **iv)** Energy-level diagram calculated by TDDFT M06/6-31G(d,p) method in ACN.

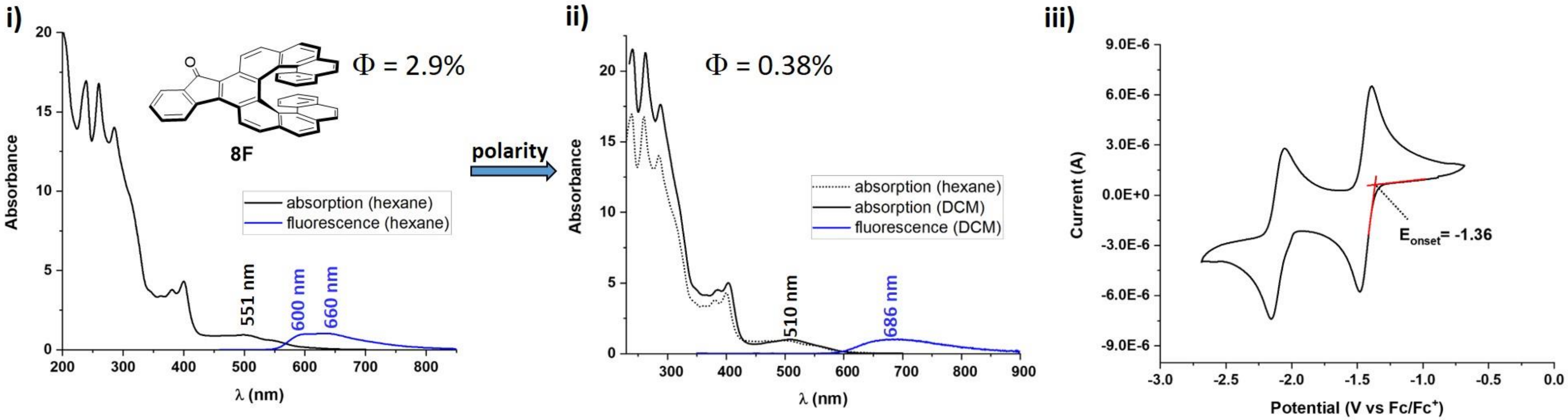

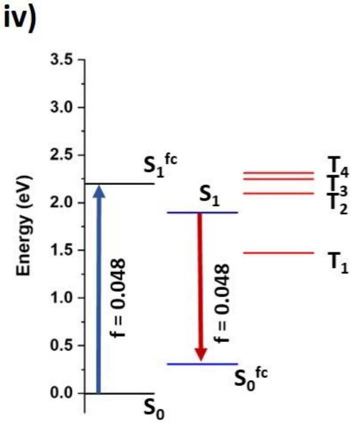

$\lambda_{(onset)}$ [nm] DCM	$\lambda_{(em)}$ $\lambda_{(exc)}$ [a] [nm] DCM	Φ_{PL} [b] (%) hexane/DCM/ACN	t_{fl} [ns] hexane/DCM/ACN	k_r [c] [10^7 s^{-1}] hexane/DCM/ACN	k_{nr} [d] [10^7 s^{-1}] Hexane/DCM/ACN	E_{HOMO} [e] [eV]	E_{LUMO} [f] [eV]	$E_{(0,0)}$ [g] [eV] Hexane (DCM)	$E_{(0,0)}$ [h] [eV]
605	686(303)	2.9/0.38/0.70	5.07/0.74/0.76	0.57/0.51/0.92	19.15/135/130	-5.62	-3.44	2.18 (2.07)	1.90

State	E [eV] DCM
S_1	1.90
T_1	1.472
T_2	2.099
T_3	2.252
T_4	2.312

a) wavelength of excitation b) Measured at room temperature (c ≈ 1 x 10^{-6} M) c) radiative rate constant calculated as k_r = Φ_{PL}/t_{fl} d) nonradiative decay rate constant calculated as $\Phi_{PL} = k_r/(k_r+k_{nr})$ e) Calculated as $E_{HOMO} = E_{LUMO} - E(0,0)$ f) Calculated using the equation $E_{LUMO} = -[E'_{red/onset} + 4.8]$ referenced against Fc/Fc^+ g) Determined from the intersection of the absorption and emission curves and using equation $E_g = hc/\lambda$ h) Calculated by TDDFT M06/6-31G(d,p) method

i) UV-vis absorption and emission ($\lambda_{(exc)}$ = 303nm) in hexane **ii)** UV-vis absorption and emission ($\lambda_{(exc)}$ = 303nm) in dichloromethane (DCM) **iii)** CV voltammogram vs Fc/Fc^+ redox couple in dimethylformamide (DMF) with a supporting electrolyte $[Bu_4N][PF_6]$ (0.1M), at a scan rate 0.1 V s^{-1} **iv)** Energy-level diagram calculated by TDDFT M06/6-31G(d,p) method in ACN.

Table S2: photophysical data

fluoreno[6]helicenes

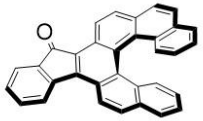

1F

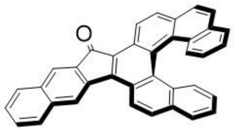

2F

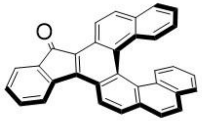

3F

fluoreno[7]helicenes

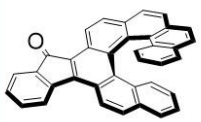

4F

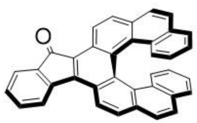

5F

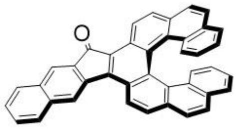

6F

fluoreno[8]helicene

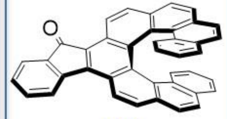

7F

fluoreno[9]helicene

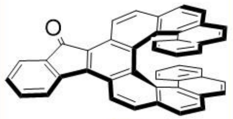

8F

Helicene	$\lambda_{(onset)}$ [nm] hexane	$\lambda_{(em)}$ $\lambda_{(exc)}$ [a] [nm] hexane	Φ_{PL} [b] (%) hexane/DCM/ACN	t_{fl} [ns] hexane/DCM/ACN	k_r [c] [10^7 s^{-1}] hexane/DCM/ACN	k_{nr} [d] [10^7 s^{-1}] Hexane/DCM/ACN	E_{HOMO} [e] [eV]	E_{LUMO} [f] [eV]	$E_{(0,0)}$ [g] [eV] Hexane (DCM)	$E_{(0,0)}$ [h] [eV]
1F	535	545(303)	5.5/0.6/0.6	6.38/1.14/1.15	0.86/0.53/0.52	14.8/87.2/86.4	-5.8	-3.43	2.37 (2.24)	2.05
2F	515	521(336)	22.0/13.9/13.5	10.2/8.96/8.28	2.2/1.5/1.6	7.6/9.6/10.4	-5.87	-3.43	2.44 (2.34)	2.22
3F	534	545(303)	11.5/1.9/1.85	9.21/1.9/2.0	1.2/1.1/0.9	9.8/57.4/49.1	-5.79	-3.42	2.37 (2.27)	2.08
4F	549	557(303)	6.9/1.07/0.76	6.48/9.1/8.4	1.07/0.12/0.09	14.4/10.9/11.8	-5.68	-3.37	2.31 (2.23)	2.01
5F	549	560(336)	4.22/0.55/0.61	6.38/1.15/1.16	0.66/0.47/0.53	15.0/85.7/86.4	-5.71	-3.4	2.31 (2.23)	2.00
6F	528	532(336)	12.0/10.0/8.4	7.1/6.9/6.3	1.7/1.45/1.32	12.5/13.0/14.5	-5.76	-3.37	2.39 (2.27)	2.16
7F	560	575(303)	6.2/0.86/0.72	6.23/0.95/0.94	1.0/0.91/0.76	15.0/104.9/105.4	-5.69	-3.43	2.26 (2.19)	1.96
8F	588	600(303)	2.9/0.38/0.70	5.07/0.74/0.76	0.57/0.51/0.92	19.15/135.0/130.1	-5.62	-3.44	2.18 (2.07)	1.90

a) Wavelength of excitation b) Measured in dichloromethane (c ≈ 1 x 10^{-5} M) c) radiative rate constant calculated as $k_r = \Phi_{PL}/t_{fl}$ d) nonradiative decay rate constant calculated as $\Phi_{PL} = k_r/(k_r+k_{nr})$ e) Calculated as $E_{HOMO} = E_{LUMO} – E(0,0)$ f) Calculated using the equation $E_{LUMO} = -[E'_{red/onset} + 4.8]$ referenced against Fc/Fc^+. g) Determined from the intersection of the absorption and emission curves and using equation $E_g = hc/\lambda$ h) Calculated by TDDFT M06/6-31G(d,p) method

Figure S31. Plots showing the evolution of energy levels.

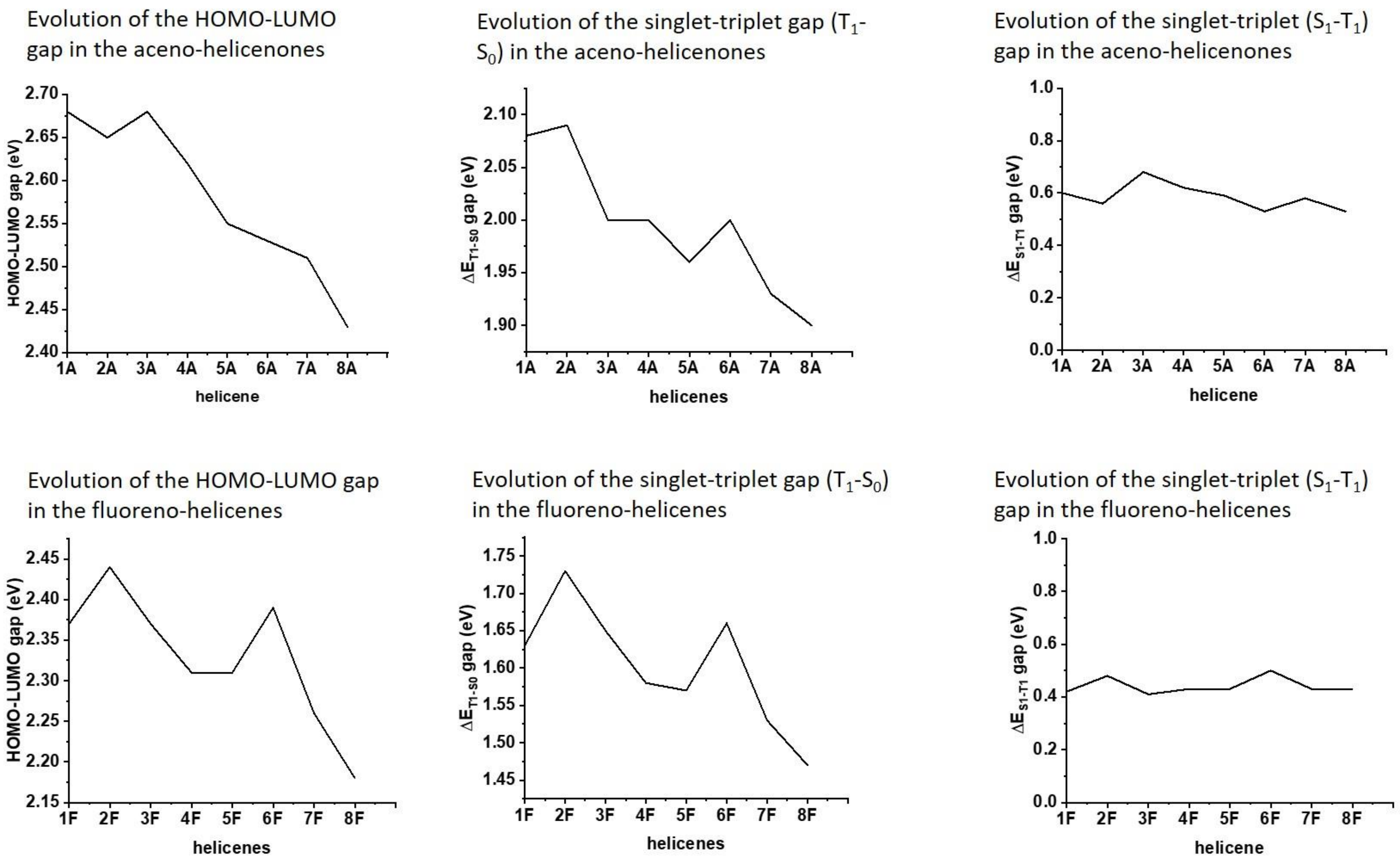

Experimental and Calculated Circular Dichroism Spectra

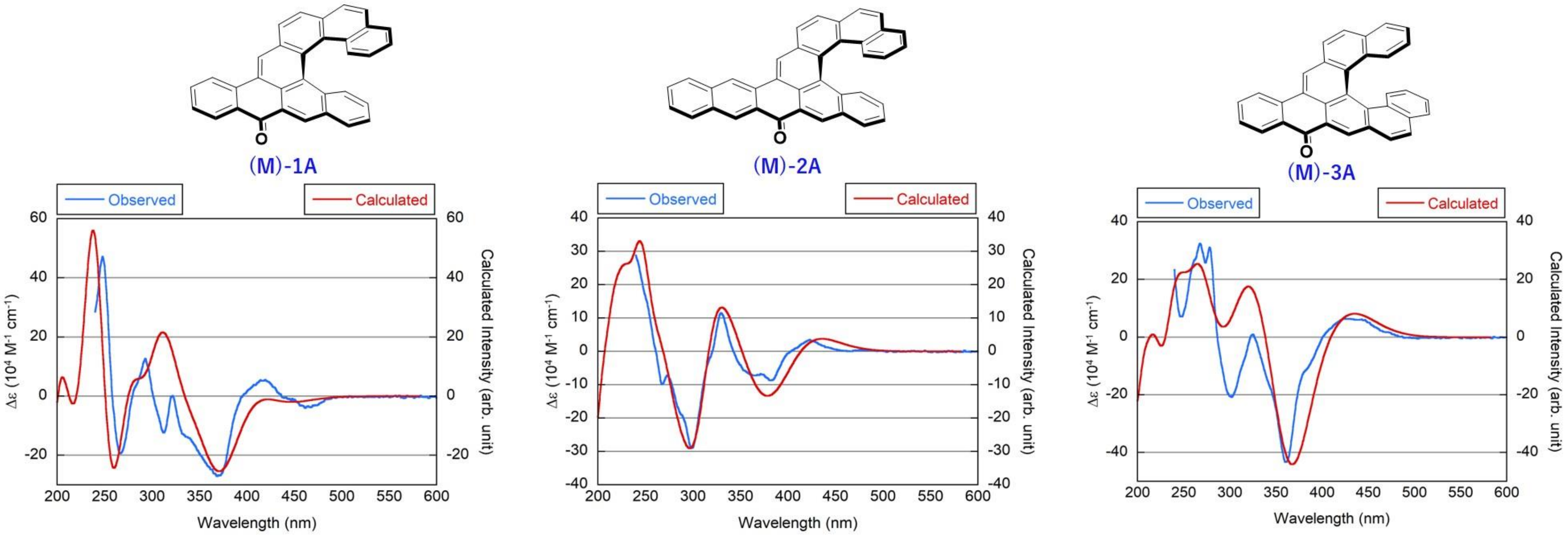

All calculations were performed using Gaussian 16 Rev C.02. The molecular geometries were optimized at the CAM-B3LYP/Def2SVP level. The CD spectra were calculated using the time-dependent density functional theory (TD-DFT) method at the CAM-B3LYP/Def2TZVP level with the solvent effect (dichloromethane; SMD model). The calculated spectra were shifted by −0.35 eV to compensate for the overestimation of the vertical excitation energy by the CAM-B3LYP functional.

Experimental and Calculated Circular Dichroism Spectra

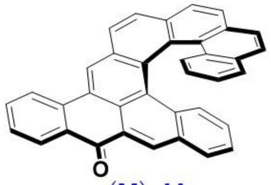

(M)-4A

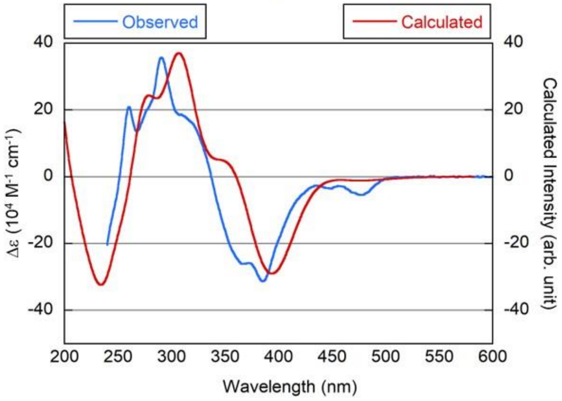

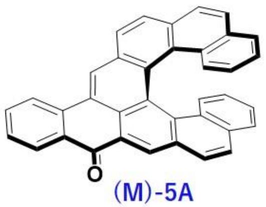

(M)-5A

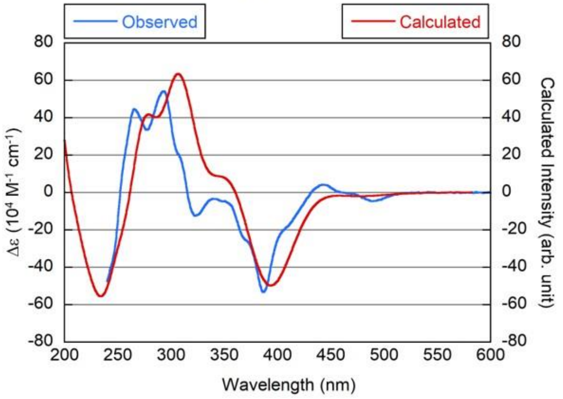

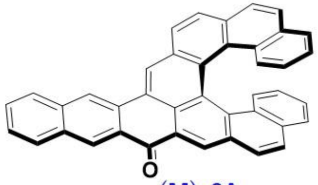

(M)-6A

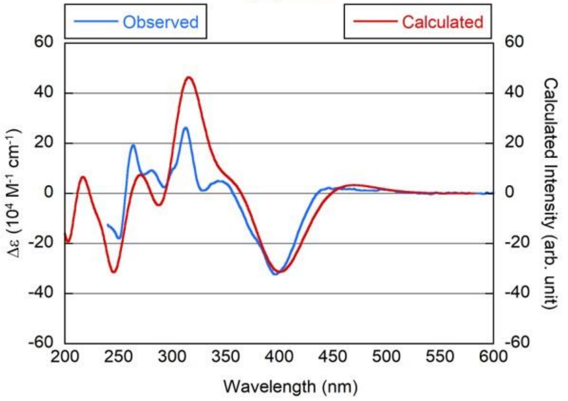

All calculations were performed using Gaussian 16 Rev C.02. The molecular geometries were optimized at the CAM-B3LYP/Def2SVP level. The CD spectra were calculated using the time-dependent density functional theory (TD-DFT) method at the CAM-B3LYP/Def2TZVP level with the solvent effect (dichloromethane; SMD model). The calculated spectra were shifted by −0.35 eV to compensate for the overestimation of the vertical excitation energy by the CAM-B3LYP functional.

Experimental and Calculated Circular Dichroism Spectra

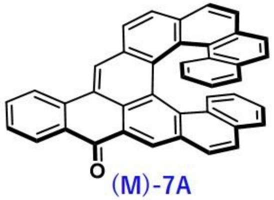

(M)-7A

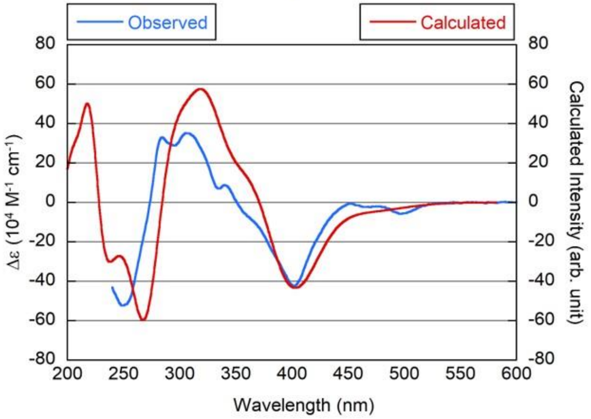

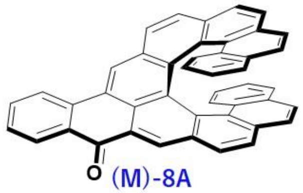

(M)-8A

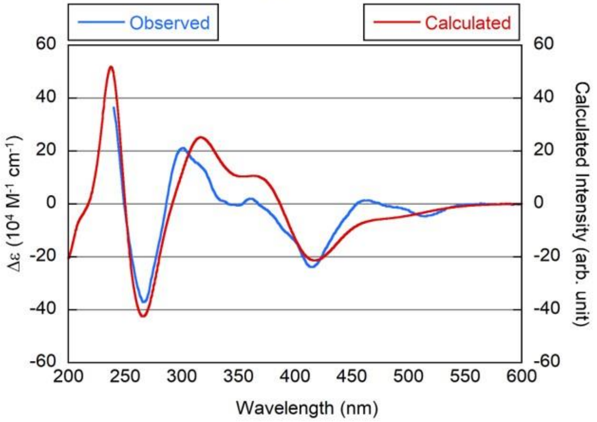

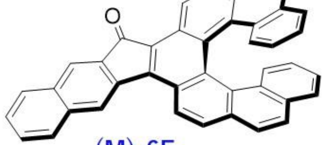

(M)-6F

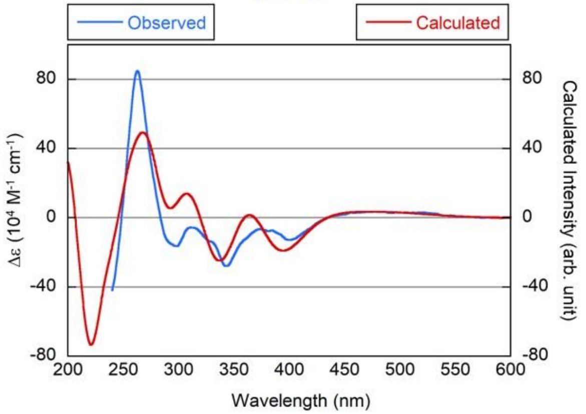

All calculations were performed using Gaussian 16 Rev C.02. The molecular geometries were optimized at the CAM-B3LYP/Def2SVP level. The CD spectra were calculated using the time-dependent density functional theory (TD-DFT) method at the CAM-B3LYP/Def2TZVP level with the solvent effect (dichloromethane; SMD model). The calculated spectra were shifted by −0.35 eV to compensate for the overestimation of the vertical excitation energy by the CAM-B3LYP functional.

Figure S32

Comparison of HOMO and LUMO orbitals with apparent loss of symmetry

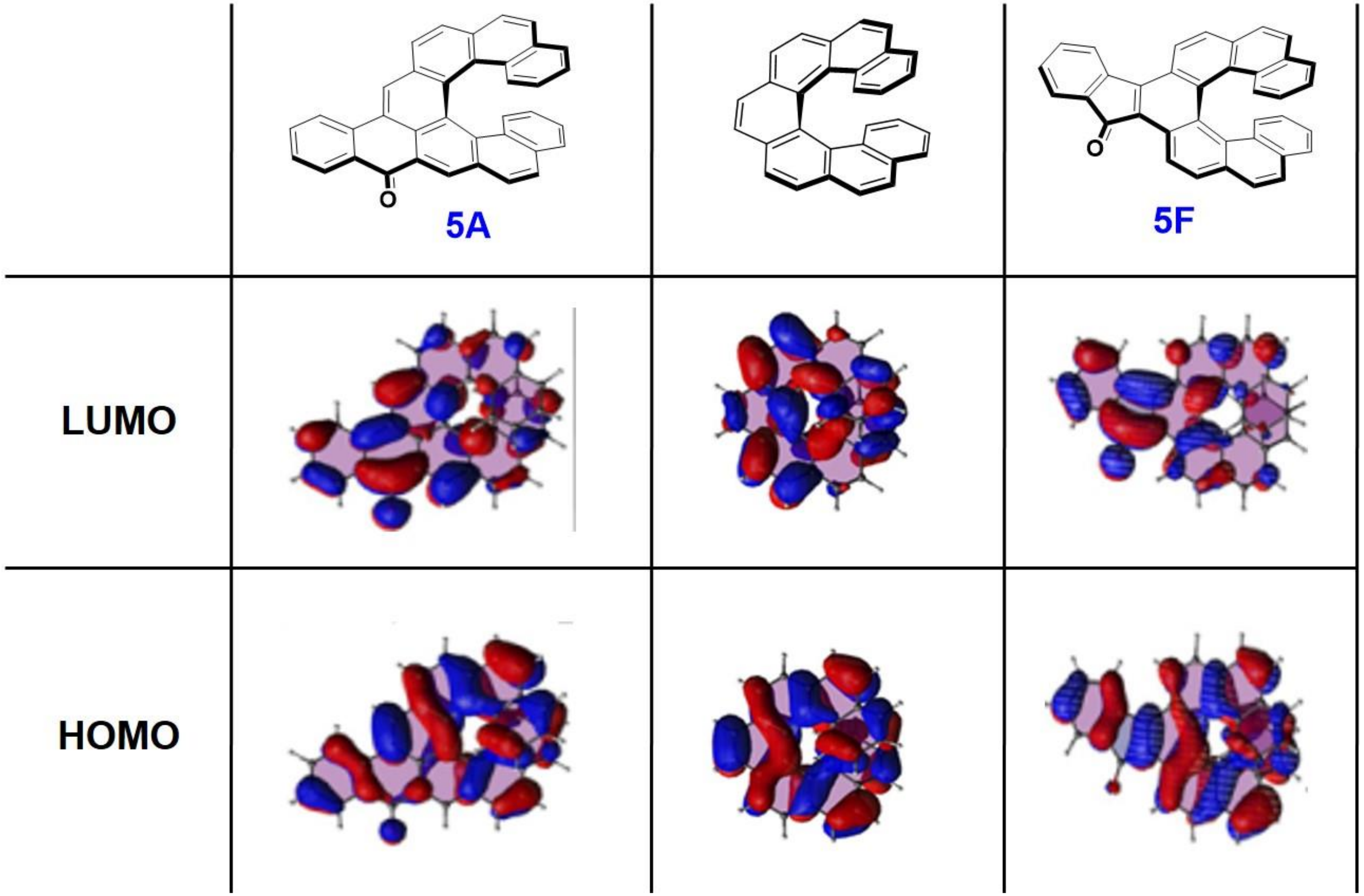

Table S3

Dipole moments of acenohelicenes in the ground and excited state

acenohelicene	Hexane $\mu(S_0)$ / $\mu(S_1)$	DCM $\mu(S_0)$ / $\mu(S_1)$	ACN $\mu(S_0)$ / $\mu(S_1)$
**1A**	4.30 D / 10.37 D	5.20 D / 11.11 D	
**2A**	3.79 D / 9.16 D		4.89 D / 12.05 D
**3A**	4.69 D / 7.87 D		5.87 D / 10.59 D
**4A**	4.12 D / 8.60 D		5.44 D / 12.01 D
**5A**	4.70 D / 10.22 D	5.67 D / 12.64 D	
**6A**	4.15 D / 8.51D	5.05 D / 10.52 D	
**7A**	4.53 D / 9.30 D		
**8A**	4.72 D / 8.89 D	5.81 D / 11.32 D	

Figure S33

Proposed mechanism for two single electron reductions

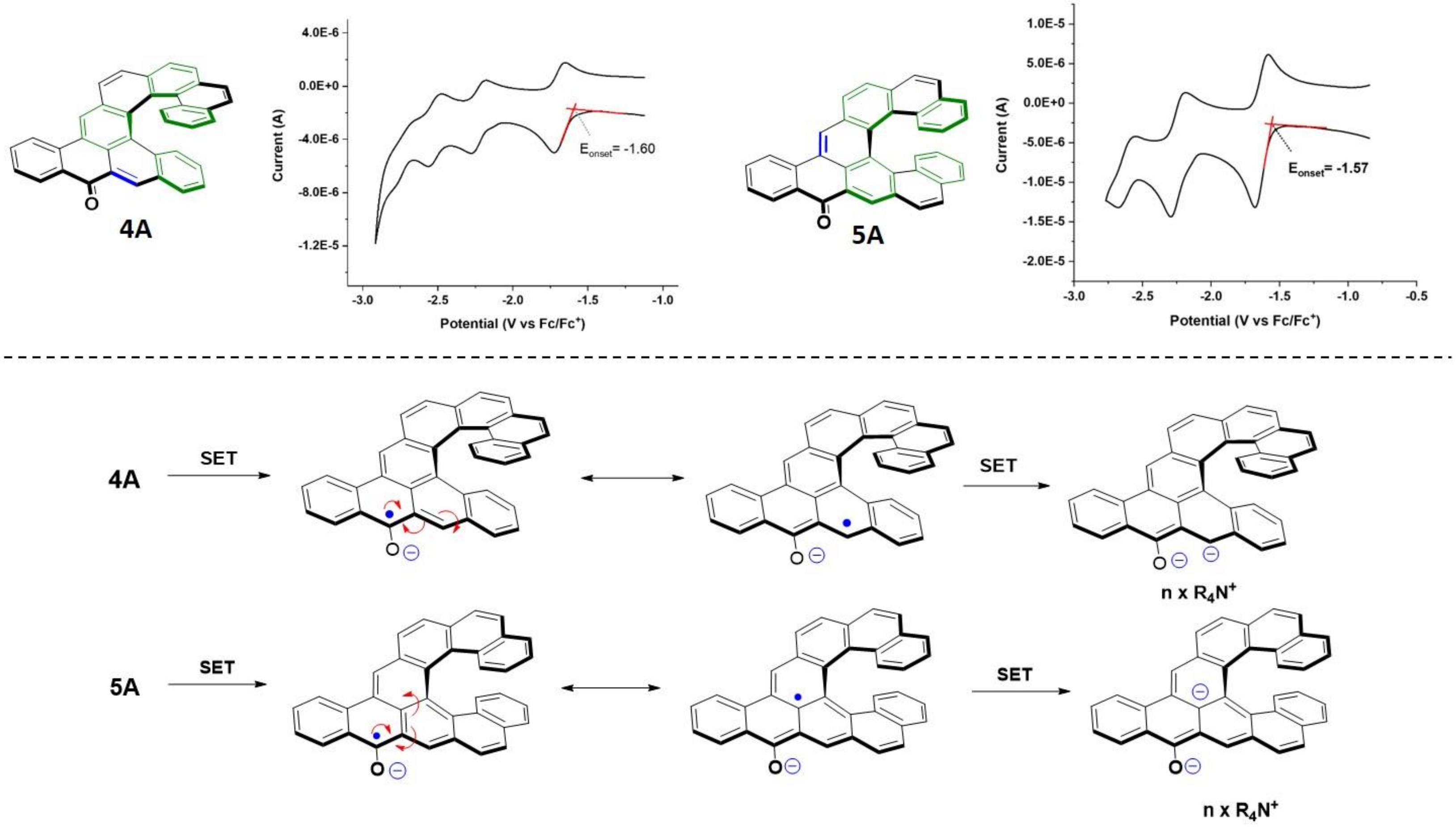

Figure S34

X-ray structure and crystal packing of racemic **5F** (CCDC 2295946), racemic **6F** (CCDC 2295945) and recamic **8F** (CCDC 2295947).
Two conformers of each enantiomer in a racemic mixture were found in the centrosymetric unit cells of 5F anf 6F.
For 8F, the absolute structure cannot be determinded reliably due to the source and the absence of strong scatterer / heavy atom.

5F

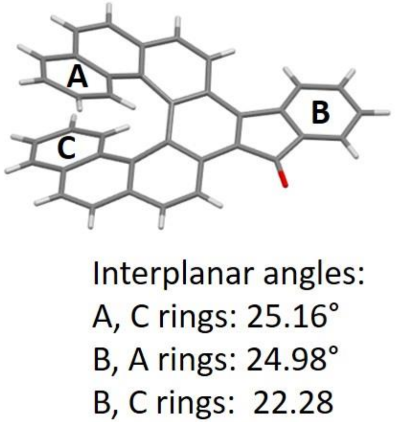

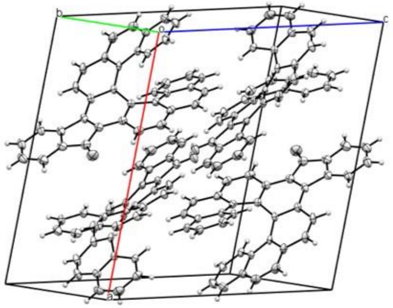

Interplanar angles:
A, C rings: 25.16°
B, A rings: 24.98°
B, C rings: 22.28

6F

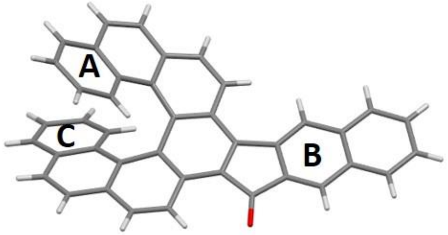

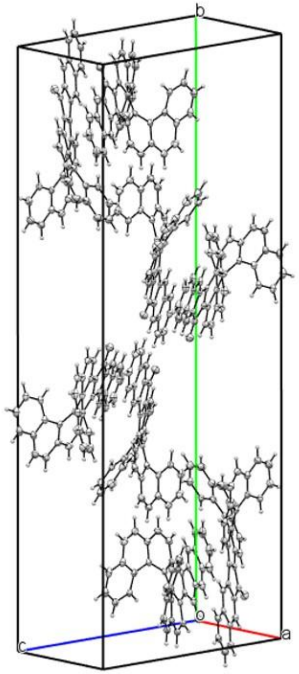

Interplanar angles:
A, C rings: 30.48°
A, B rings: 38.17°
B, C rings: 33.94

8F

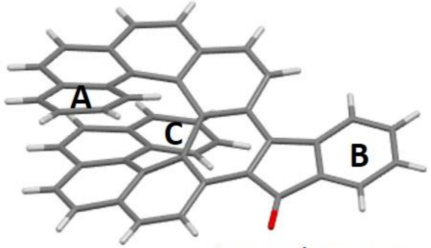

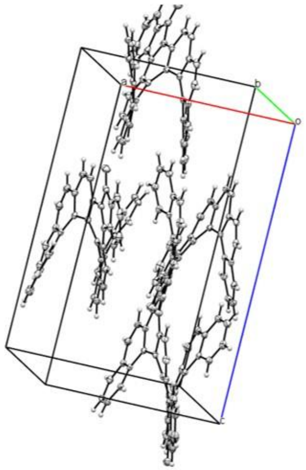

Interplanar angles:
A, C rings: 15.65°
B, A rings: 32.96°
B, C rings: 31.68

Table S4: crystallographic data

Compound	5F	6F	8F
**Formula**	$C_{37}H_{20}O$	$C_{41}H_{22}O$	$C_{45}H_{24}O$
**FW (g·mol^{-1})**	480.53	530.58	580.64
**Crystal color**	purple	orange	red
**Crystal size (mm)**	0.60 x 0.32 x 0.20	0.32 x 0.11 x 0.02	0.35 x 0.18 x 0.12
**Crystal system**	monoclinic	monoclinic	orthorhombic
**Space group**	$P2_1/c$	$P2_1/c$	$P2_12_12_1$
**Temperature**	120 K	120 K	120 K
***a* (Å)**	15.5109(11)	8.0168(4)	10.6528(7)
***b* (Å)**	8.9771(7)	42.038(2)	13.9671(9)
***c* (Å)**	16.8196(13)	15.3836(7)	18.2044(11)
***β* (°)**	101.427(4)	97.965(3)	90
***V* (Å^3)**	2295.6(3)	5134.4(4)	2708.6(3)
Z	4	8	4
**d_{calc}**	1.390	1.373	1.424
**μ (mm^{-1})**	0.082	0.081	0.083
**θ_{min} - θ_{max}**	2.471 ° - 30.464°	1.651 ° - 25.051 °	1.838° - 28.354°
**Refl. coll. / unique**	156291 / 6760	110110 / 9071	201477 / 6779
**Completeness to 2θ**	0.971	0.996	1.000
**R_{int}**	0.0304	0.0822	0.0081
**Refined param./restr.**	343 / 0	757 / 0	416 / 0
**$^aR_1$ ($I > 2\sigma(I)$)**	0.0861	0.0632	0.0442
**$^bwR_2$ (all data)**	0.2198	0.1397	0.1296
**Goodness of fit**	1.164	1.057	1.117

$^aR_1 = \Sigma||F_0| - |F_C||/\Sigma|F_0|$ and $^bwR_2 = [\Sigma w(F_0^2 - F_C^2)^2/\Sigma w(F_o^2)^2]^{1/2}$

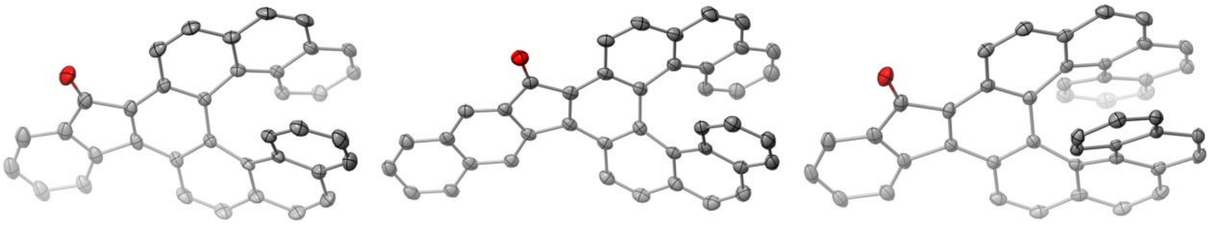

ORTEP-type views of 5F, 6F and 8F as found in the crystals at 120 K. Thermal ellipsoids are depicted at a 50 % probability level. H atoms are omitted for clarity. C: grey, O: red.

Table S5. xyz coordinates [in A] and energies [in A.U.] of molecules optimised in ground (S0) and first electronically excited (S1) states

			1A			
	E(S0) =	-1342.6886	A.U.	E(S1) =	-1342.5935	A.U.
	x	y	z	x	y	z
H	-6.0630	-1.4168	0.7522	5.9915	1.5126	0.7439
H	-7.4844	0.6403	0.8671	7.4389	-0.5168	0.8963
H	-6.4168	2.8630	0.5248	6.4243	-2.7706	0.5635
C	-5.6386	-0.4277	0.6009	5.5810	0.5167	0.5978
C	-4.2680	-0.3410	0.3372	4.2128	0.4208	0.3194
C	-1.5883	2.1824	-0.4271	1.6019	-2.2109	-0.4580
C	-2.2118	0.9934	-0.1371	2.2189	-0.9692	-0.1646
C	-0.1832	2.2891	-0.5228	0.2298	-2.3395	-0.5483
C	-1.4208	-0.2006	-0.1813	1.4204	0.2162	-0.2280
C	0.6315	1.1469	-0.3146	-0.6031	-1.1822	-0.3427
C	-0.0260	-0.1355	-0.4062	0.0296	0.1129	-0.4786
C	0.4114	3.5473	-0.8144	-0.3798	-3.6150	-0.7951
C	1.7649	3.6679	-0.8746	-1.7270	-3.7536	-0.7636
C	2.5995	2.5960	-0.4559	-2.5515	-2.6665	-0.3364
C	2.0320	1.3681	-0.0423	-1.9560	-1.4089	0.0159
H	-2.1598	3.0996	-0.5498	2.2058	-3.1054	-0.5882
C	-3.6536	0.9093	0.1305	3.6266	-0.8602	0.1133
H	-0.2401	4.3939	-1.0215	0.2683	-4.4566	-1.0308
H	2.2323	4.6058	-1.1682	-2.2040	-4.7002	-1.0071
C	4.0142	2.7808	-0.4219	-3.9504	-2.8094	-0.2902
C	2.8802	0.4564	0.7027	-2.7843	-0.4618	0.7466
C	4.2879	0.6571	0.6937	-4.1978	-0.6187	0.7215
C	4.8346	1.8195	0.0737	-4.7603	-1.7910	0.1573
H	4.4179	3.7125	-0.8139	-4.3858	-3.7442	-0.6369
H	5.9154	1.9480	0.0695	-5.8426	-1.9024	0.1487
C	-1.3341	-2.6322	-0.1892	1.2326	2.6488	-0.2167
C	-2.0534	-1.4788	-0.0357	2.0159	1.5111	-0.0757
C	0.0091	-2.6029	-0.6286	-0.0959	2.5680	-0.6649
C	0.6524	-1.3486	-0.8167	-0.6857	1.2864	-0.9023
C	0.6833	-3.8036	-0.9494	-0.8691	3.7288	-0.9157
C	1.9363	-3.7764	-1.5089	-2.1276	3.6393	-1.4606
C	2.5334	-2.5359	-1.7993	-2.6561	2.3810	-1.8166
C	1.9079	-1.3569	-1.4643	-1.9439	1.2355	-1.5438
H	-1.8374	-3.5856	-0.0395	1.6926	3.6182	-0.0381
C	-3.4908	-1.5934	0.2853	3.4288	1.6568	0.2428
H	0.1721	-4.7472	-0.7675	-0.4320	4.6992	-0.6861
H	2.4490	-4.7011	-1.7601	-2.7024	4.5418	-1.6537
H	3.4970	-2.5055	-2.3018	-3.6197	2.3163	-2.3149
H	2.3873	-0.4153	-1.7141	-2.3544	0.2713	-1.8343
C	5.1322	-0.2607	1.3551	-5.0186	0.3561	1.3282

C	2.3782	-0.5936	1.5040	-2.2521	0.5775	1.5249
C	4.6125	-1.3163	2.0658	-4.4662	1.4209	2.0094
C	3.2181	-1.4602	2.1655	-3.0743	1.5098	2.1364
H	6.2078	-0.0974	1.3103	-6.0987	0.2365	1.2680
H	5.2717	-2.0142	2.5757	-5.1061	2.1651	2.4752
H	2.7966	-2.2544	2.7769	-2.6314	2.3100	2.7233
H	1.3045	-0.7148	1.6135	-1.1760	0.6524	1.6567
O	-4.0161	-2.6845	0.4937	3.9517	2.7725	0.4301
C	-6.4193	0.7112	0.6638	6.3774	-0.6107	0.6808
C	-5.8190	1.9566	0.4689	5.8080	-1.8783	0.4902
C	-4.4615	2.0532	0.2116	4.4615	-1.9966	0.2144
H	-4.0274	3.0407	0.0846	4.0428	-2.9913	0.0898
			**2A**			
	E(S0) =	-1496.2203	A.U.	E(S1) =	-1496.1281	A.U.
	x	y	z	x	y	z
H	-3.3364	0.2379	-1.6633	-3.3153	0.1347	-1.7520
H	7.3523	1.5714	0.7029	7.2869	1.6112	0.7567
H	8.9905	-0.2860	0.7346	8.9293	-0.2350	0.8144
H	8.2282	-2.6283	0.4012	8.1989	-2.5847	0.4580
C	7.9347	-0.4880	0.5740	7.8791	-0.4473	0.6311
H	4.9878	2.3426	0.4831	4.9260	2.3805	0.4880
C	7.0302	0.5420	0.5564	6.9673	0.5828	0.5994
C	6.1729	-2.1011	0.1797	6.1423	-2.0679	0.1959
C	4.6883	1.3076	0.3285	4.6301	1.3450	0.3284
C	3.3524	1.0376	0.1186	3.2918	1.0863	0.0961
C	0.9996	-1.8219	-0.6094	1.0100	-1.8472	-0.6730
C	1.4719	-0.5676	-0.3080	1.4720	-0.5470	-0.3572
C	-0.3809	-2.1154	-0.6476	-0.3371	-2.1494	-0.7097
C	0.5277	0.5107	-0.2877	0.5243	0.5291	-0.3502
C	-1.3310	-1.0987	-0.3727	-1.2997	-1.1189	-0.4137
C	-0.8551	0.2617	-0.4562	-0.8507	0.2518	-0.5391
C	-0.8144	-3.4360	-0.9493	-0.7842	-3.4829	-0.9890
C	-2.1406	-3.7375	-0.9539	-2.0992	-3.7940	-0.9075
C	-3.0915	-2.7976	-0.4697	-3.0329	-2.8432	-0.3872
C	-2.6757	-1.5132	-0.0480	-2.5963	-1.5362	-0.0053
H	1.6833	-2.6493	-0.7852	1.7156	-2.6501	-0.8694
C	2.9008	-0.3016	-0.0899	2.8610	-0.2684	-0.1274
H	-0.0656	-4.1821	-1.2079	-0.0438	-4.2232	-1.2842
H	-2.4911	-4.7235	-1.2526	-2.4638	-4.7842	-1.1714
C	-4.4650	-3.1744	-0.3787	-4.3949	-3.1906	-0.2672
C	-3.6041	-0.7407	0.7562	-3.5038	-0.7382	0.8041
C	-4.9708	-1.1307	0.8028	-4.8775	-1.1018	0.8635
C	-5.3840	-2.3443	0.1776	-5.3055	-2.3222	0.2783
H	-4.7565	-4.1443	-0.7773	-4.7133	-4.1637	-0.6351
H	-6.4366	-2.6189	0.2170	-6.3598	-2.5865	0.3302
C	0.1094	2.9089	-0.2254	0.0279	2.9173	-0.2600

C	0.9858	1.8622	-0.1358	0.9590	1.8865	-0.1854
C	-1.2363	2.7055	-0.6075	-1.2972	2.6762	-0.6498
C	-1.7091	1.3796	-0.8059	-1.7341	1.3335	-0.8878
C	-2.0821	3.8091	-0.8643	-2.2194	3.7379	-0.8443
C	-3.3438	3.6224	-1.3722	-3.4788	3.5018	-1.3348
C	-3.7768	2.3187	-1.6761	-3.8665	2.1901	-1.6870
C	-2.9807	1.2298	-1.4026	-3.0084	1.1381	-1.4669
H	0.4840	3.9190	-0.0711	0.3675	3.9340	-0.0760
C	2.4070	2.1705	0.1224	2.3511	2.2078	0.0756
H	-1.6976	4.8097	-0.6757	-1.8926	4.7514	-0.6170
H	-3.9892	4.4730	-1.5742	-4.1688	4.3286	-1.4863
C	-5.9001	-0.3487	1.5229	-5.7892	-0.2852	1.5670
C	-3.2122	0.3526	1.5617	-3.0805	0.3506	1.5864
C	-5.4957	0.7544	2.2365	-5.3553	0.8259	2.2556
C	-4.1302	1.0847	2.2799	-3.9849	1.1227	2.2910
H	-6.9448	-0.6557	1.5201	-6.8403	-0.5678	1.5737
H	-6.2190	1.3467	2.7912	-6.0630	1.4476	2.7970
H	-3.7923	1.9170	2.8925	-3.6262	1.9615	2.8819
H	-2.1607	0.6162	1.6284	-2.0222	0.5886	1.6468
O	2.7852	3.3219	0.3267	2.7363	3.3819	0.2643
C	5.6524	0.2847	0.3469	5.5982	0.3247	0.3630
C	5.2130	-1.0585	0.1528	5.1772	-1.0281	0.1544
C	3.8419	-1.3137	-0.0552	3.8218	-1.2873	-0.0838
H	3.5453	-2.3530	-0.1785	3.5301	-2.3261	-0.2190
H	5.8371	-3.1262	0.0330	5.8125	-3.0932	0.0380
C	7.4999	-1.8213	0.3841	7.4648	-1.7842	0.4284
H	-4.7478	2.1674	-2.1410	-4.8377	2.0135	-2.1417
			**3A**			
	E(S0) =	-1342.6902	A.U.	E(S1) =	-1342.5953	A.U.
	x	y	z	x	y	z
H	6.0611	-1.6429	-0.4062	6.0404	-1.6740	-0.3931
H	7.5827	0.2424	0.2203	7.5629	0.2424	0.1022
H	6.5869	2.4335	0.8572	6.5884	2.4681	0.6584
C	5.6675	-0.6704	-0.1222	5.6440	-0.6935	-0.1422
C	4.2784	-0.5065	-0.1146	4.2557	-0.5492	-0.1035
C	1.6392	2.0948	0.5258	1.6482	2.1098	0.5684
C	2.2423	0.8974	0.2268	2.2578	0.8759	0.2392
C	0.2535	2.2993	0.3416	0.2929	2.3060	0.4049
C	1.4015	-0.2248	-0.0650	1.4155	-0.2598	-0.0241
C	-0.5694	1.2357	-0.0862	-0.5315	1.2159	-0.0685
C	-0.0136	-0.0950	-0.0372	-0.0012	-0.1192	0.0230
C	-0.3011	3.5996	0.5512	-0.3054	3.5867	0.6532
C	-1.6031	3.8544	0.2678	-1.5914	3.8254	0.3133
C	-2.4081	2.8638	-0.3713	-2.3555	2.8375	-0.4006
C	-1.8744	1.5694	-0.6196	-1.7945	1.5509	-0.6570
H	2.2258	2.9558	0.8372	2.2457	2.9474	0.9166

C	3.7022	0.7292	0.2400	3.6869	0.7149	0.2194
H	0.3480	4.3762	0.9515	0.3115	4.3595	1.1066
H	-2.0365	4.8347	0.4566	-2.0535	4.7908	0.5085
C	-3.7099	3.1810	-0.8148	-3.6391	3.1356	-0.8678
C	-2.6292	0.6962	-1.4351	-2.5191	0.6692	-1.4971
C	-3.8852	1.0370	-1.8827	-3.7724	0.9966	-1.9721
C	-4.4496	2.2786	-1.5423	-4.3535	2.2231	-1.6323
H	-4.1066	4.1694	-0.5886	-4.0678	4.1079	-0.6330
H	-5.4492	2.5355	-1.8833	-5.3488	2.4742	-1.9888
C	1.1720	-2.5667	-0.6469	1.1378	-2.6162	-0.5766
C	1.9800	-1.4891	-0.3828	1.9813	-1.5301	-0.3190
C	-0.2154	-2.5108	-0.4111	-0.2327	-2.5302	-0.3523
C	-0.7974	-1.3044	0.0448	-0.8090	-1.3006	0.1089
C	-1.0175	-3.6807	-0.5893	-1.0864	-3.6566	-0.5786
C	-2.3316	-3.6784	-0.2550	-2.4064	-3.6089	-0.2700
C	-2.9019	-2.5515	0.4120	-2.9645	-2.4736	0.4033
C	-2.1184	-1.3859	0.6356	-2.1444	-1.3404	0.6656
H	1.6303	-3.5007	-0.9664	1.5823	-3.5512	-0.9091
C	3.4416	-1.6631	-0.4832	3.4213	-1.7187	-0.4018
H	-0.5442	-4.5664	-1.0083	-0.6452	-4.5530	-1.0106
H	-2.9549	-4.5550	-0.4205	-3.0544	-4.4616	-0.4656
C	-4.2208	-2.6128	0.9094	-4.2950	-2.4970	0.8578
C	-2.6533	-0.3899	1.4834	-2.6762	-0.3341	1.5006
C	-3.9313	-0.4844	1.9850	-3.9775	-0.3869	1.9568
C	-4.7396	-1.5893	1.6674	-4.8099	-1.4604	1.6101
H	-4.8111	-3.5035	0.7011	-4.9081	-3.3649	0.6195
H	-5.7552	-1.6501	2.0500	-5.8397	-1.4901	1.9564
H	-4.3119	0.2965	2.6385	-4.3536	0.4080	2.5964
H	-2.0404	0.4644	1.7557	-2.0458	0.5001	1.7995
O	3.9376	-2.7281	-0.8461	3.9247	-2.8196	-0.6929
C	6.5041	0.3737	0.2246	6.4844	0.3741	0.1322
C	5.9436	1.6016	0.5817	5.9376	1.6241	0.4468
C	4.5696	1.7760	0.5867	4.5671	1.7892	0.4862
H	4.1738	2.7481	0.8656	4.1720	2.7733	0.7197
H	-4.4379	0.3440	-2.5122	-4.3036	0.3010	-2.6159
H	-2.2067	-0.2609	-1.7271	-2.0714	-0.2788	-1.7815
			**4A**			
	E(S0) =	-1496.2184	A.U.	E(S1) =	-1496.1271	A.U.
	x	y	z	x	y	z
H	1.3935	-3.7936	2.9478	1.1322	-3.7599	2.9594
C	1.6591	-2.8502	2.4774	1.4409	-2.8260	2.4970
C	0.7458	-1.7814	2.4734	0.5487	-1.7413	2.4387
H	-6.0748	-1.8172	0.2874	-6.0119	-1.8124	0.3090
H	-7.6828	0.0373	0.7723	-7.5937	0.0413	0.8561
H	-6.8379	2.3784	0.8381	-6.7450	2.3855	0.9283
C	-5.7481	-0.7812	0.3196	-5.6765	-0.7789	0.3436

C	-4.3957	-0.5168	0.0788	-4.3260	-0.5308	0.0676
C	-1.9702	2.3473	-0.1589	-1.9240	2.3886	-0.2464
C	-2.4820	1.0725	-0.1332	-2.4433	1.0694	-0.1775
C	-0.5803	2.5957	-0.2295	-0.5683	2.6391	-0.3305
C	-1.5903	-0.0129	-0.4216	-1.5672	-0.0191	-0.4672
C	0.3272	1.5107	-0.2809	0.3522	1.5280	-0.3427
C	-0.2154	0.2251	-0.6420	-0.1929	0.2270	-0.7071
C	-0.0925	3.9331	-0.2422	-0.0527	3.9778	-0.3305
C	1.2448	4.1713	-0.3179	1.2824	4.1987	-0.2848
C	2.1773	3.1047	-0.1806	2.1935	3.1095	-0.0988
C	1.7151	1.7792	0.0229	1.6867	1.7716	0.0501
H	-2.6203	3.2149	-0.0755	-2.5938	3.2435	-0.2006
C	-3.9060	0.8039	0.1075	-3.8327	0.8042	0.0939
H	-0.8119	4.7495	-0.2441	-0.7601	4.8024	-0.3912
H	1.6294	5.1834	-0.4275	1.6912	5.2045	-0.3537
C	3.5732	3.3640	-0.2478	3.5764	3.3108	-0.1500
C	2.6643	0.8006	0.4969	2.6143	0.7550	0.5347
C	4.0367	1.0515	0.2792	4.0021	0.9690	0.3276
C	4.4695	2.3465	-0.1187	4.4598	2.2486	-0.0353
H	3.9029	4.3810	-0.4507	3.9505	4.3154	-0.3365
H	5.5356	2.5191	-0.2536	5.5289	2.4082	-0.1587
C	-1.2631	-2.3878	-0.8376	-1.2178	-2.3896	-0.9247
C	-2.0932	-1.3525	-0.4999	-2.0661	-1.3618	-0.5543
C	0.0627	-2.1520	-1.2676	0.0962	-2.1320	-1.3518
C	0.5704	-0.8241	-1.2548	0.5947	-0.7904	-1.3289
C	0.8498	-3.2086	-1.7816	0.9345	-3.1684	-1.8260
C	2.0771	-2.9588	-2.3438	2.1823	-2.8949	-2.3342
C	2.5314	-1.6305	-2.4438	2.6267	-1.5596	-2.4250
C	1.7970	-0.5924	-1.9168	1.8502	-0.5349	-1.9369
H	-1.6652	-3.3992	-0.8458	-1.6070	-3.4056	-0.9349
C	-3.5062	-1.6591	-0.2029	-3.4620	-1.6693	-0.2517
H	0.4442	-4.2182	-1.7495	0.5584	-4.1898	-1.7986
H	2.6757	-3.7719	-2.7456	2.8109	-3.7016	-2.7027
H	3.4700	-1.4176	-2.9499	3.5807	-1.3369	-2.8963
H	2.1622	0.4239	-2.0294	2.1883	0.4911	-2.0538
C	4.9928	0.0074	0.4636	4.9256	-0.1009	0.5400
C	2.3062	-0.4117	1.2031	2.2078	-0.4141	1.2481
C	4.6111	-1.2229	0.8950	4.5005	-1.3030	1.0076
C	3.2694	-1.4506	1.3231	3.1452	-1.4814	1.4161
H	6.0340	0.2180	0.2257	5.9749	0.0721	0.3093
H	5.3331	-2.0319	0.9915	5.1946	-2.1311	1.1365
C	2.9072	-2.6739	1.9280	2.7239	-2.6846	2.0103
C	1.0651	-0.5939	1.8542	0.9225	-0.5652	1.8322
O	-3.9249	-2.8153	-0.1966	-3.8972	-2.8349	-0.2785
C	-6.6327	0.2463	0.5877	-6.5478	0.2501	0.6456
C	-6.1571	1.5587	0.6225	-6.0709	1.5688	0.6827

C	-4.8190	1.8309	0.3908	-4.7444	1.8365	0.4144
H	-4.4851	2.8632	0.4410	-4.4020	2.8659	0.4699
H	0.3534	0.2261	1.8854	0.2354	0.2756	1.8310
H	3.6523	-3.4665	1.9778	3.4410	-3.4987	2.0988
H	-0.2168	-1.8875	2.9682	-0.4406	-1.8283	2.8809

			5A			
	E(S0) =	-1496.2166	A.U.	E(S1) =	-1496.1261	A.U.
	x	y	z	x	y	z
H	-6.3756	-1.8098	0.4393	-6.3358	-1.8262	0.4414
H	-7.9873	0.0964	0.2582	-7.9393	0.0881	0.3353
H	-7.0999	2.3936	-0.1082	-7.0614	2.3961	-0.0064
C	-6.0308	-0.7925	0.2739	-5.9881	-0.8080	0.2869
C	-4.6527	-0.5827	0.1631	-4.6098	-0.6147	0.1481
C	-2.1399	2.1618	-0.3882	-2.1222	2.1809	-0.4424
C	-2.6879	0.9239	-0.1569	-2.6755	0.9029	-0.1882
C	-0.7456	2.3891	-0.3208	-0.7592	2.4046	-0.3792
C	-1.7990	-0.1988	-0.0841	-1.7982	-0.2214	-0.1289
C	0.1324	1.3161	-0.0347	0.1201	1.3101	-0.0683
C	-0.3981	-0.0185	-0.2076	-0.3943	-0.0299	-0.2730
C	-0.2244	3.6967	-0.5293	-0.2095	3.7138	-0.5839
C	1.1152	3.9205	-0.4458	1.1178	3.9364	-0.4248
C	1.9876	2.9042	0.0347	1.9632	2.9073	0.0969
C	1.4743	1.6350	0.3833	1.4143	1.6236	0.4180
H	-2.7701	3.0287	-0.5733	-2.7678	3.0283	-0.6581
C	-4.1376	0.7113	-0.0502	-4.0991	0.6952	-0.0573
H	-0.9142	4.4955	-0.7946	-0.8812	4.5107	-0.8967
H	1.5359	4.8962	-0.6816	1.5581	4.9072	-0.6407
C	3.3835	3.1703	0.1636	3.3447	3.1290	0.2486
C	2.3268	0.7506	1.1506	2.2353	0.7220	1.2061
C	3.7210	1.0199	1.2122	3.6342	0.9614	1.2982
C	4.2312	2.2326	0.6615	4.1729	2.1597	0.7669
H	3.7542	4.1374	-0.1715	3.7540	4.0833	-0.0761
H	5.3023	2.4191	0.7167	5.2448	2.3319	0.8409
C	-1.4633	-2.5869	0.1573	-1.4201	-2.6132	0.0788
C	-2.3184	-1.5151	0.0999	-2.3112	-1.5446	0.0404
C	-0.0975	-2.4488	-0.1612	-0.0715	-2.4469	-0.2456
C	0.4112	-1.1747	-0.5032	0.4316	-1.1542	-0.5925
C	0.7607	-3.5917	-0.1552	0.8298	-3.5524	-0.1950
C	2.0608	-3.4844	-0.5273	2.1356	-3.4131	-0.5493
C	2.5526	-2.2633	-1.0811	2.6170	-2.1900	-1.1113
C	1.7048	-1.1243	-1.1505	1.7438	-1.0671	-1.1989
H	-1.8712	-3.5702	0.3835	-1.8059	-3.6013	0.3203
C	-3.7627	-1.7538	0.2795	-3.7310	-1.7900	0.2219
H	0.3454	-4.5420	0.1743	0.4463	-4.5102	0.1525
H	2.7297	-4.3418	-0.4833	2.8224	-4.2550	-0.4794
C	3.8520	-2.1992	-1.6270	3.9233	-2.1084	-1.6296

C	2.1457	-0.0235	-1.9187	2.1974	0.0465	-1.9392
C	3.4035	0.0025	-2.4773	3.4732	0.0947	-2.4642
C	4.2831	-1.0788	-2.2982	4.3608	-0.9757	-2.2832
H	4.4961	-3.0720	-1.5336	4.5758	-2.9741	-1.5243
H	5.2833	-1.0442	-2.7226	5.3704	-0.9250	-2.6826
H	3.7123	0.8624	-3.0666	3.7860	0.9709	-3.0277
H	1.4731	0.8119	-2.0901	1.5240	0.8811	-2.1164
C	4.5787	0.1202	1.8814	4.4567	0.0440	1.9857
C	1.8370	-0.3340	1.9119	1.7006	-0.3447	1.9455
C	4.0776	-0.9747	2.5453	3.9120	-1.0455	2.6341
C	2.6875	-1.1773	2.5908	2.5226	-1.2189	2.6398
H	5.6468	0.3320	1.8873	5.5288	0.2288	2.0163
H	4.7462	-1.6563	3.0649	4.5521	-1.7433	3.1665
H	2.2771	-2.0010	3.1702	2.0788	-2.0385	3.1989
H	0.7655	-0.4971	1.9818	0.6233	-0.4784	1.9935
O	-4.2040	-2.8776	0.5095	-4.1878	-2.9312	0.4198
C	-6.9169	0.2636	0.1740	-6.8705	0.2555	0.2282
C	-6.4173	1.5507	-0.0331	-6.3776	1.5520	0.0337
C	-5.0539	1.7699	-0.1393	-5.0200	1.7645	-0.1031
H	-4.7033	2.7872	-0.2878	-4.6663	2.7830	-0.2362
**6A**						
	E(S0) =	-1649.7464	A.U.	E(S1) =	-1649.6576	A.U.
	x	y	z	x	y	z
H	-7.7759	-1.8096	0.4157	-7.7336	-1.8045	0.4623
H	-9.5031	-0.0430	0.2415	-9.4463	-0.0224	0.3802
H	-8.8354	2.3232	-0.1245	-8.7800	2.3485	0.0417
C	-8.4492	0.2097	0.1584	-8.3945	0.2274	0.2668
H	-5.3633	-2.4618	0.4087	-5.3289	-2.4843	0.3681
C	-7.4954	-0.7702	0.2550	-7.4446	-0.7659	0.3129
C	-6.7450	1.8994	-0.1577	-6.6928	1.9083	-0.0701
C	-5.1067	-1.4169	0.2438	-5.0659	-1.4391	0.2141
C	-3.7737	-1.0828	0.1339	-3.7286	-1.1282	0.0715
C	-1.5042	1.8877	-0.4038	-1.5008	1.8872	-0.5290
C	-1.9454	0.6064	-0.1806	-1.9351	0.5667	-0.2652
C	-0.1365	2.2356	-0.3231	-0.1659	2.2353	-0.4502
C	-0.9628	-0.4358	-0.1070	-0.9583	-0.4751	-0.1933
C	0.8299	1.2420	-0.0357	0.8040	1.2283	-0.1076
C	0.4182	-0.1319	-0.2208	0.4219	-0.1556	-0.3192
C	0.2690	3.5855	-0.5212	0.2591	3.5881	-0.6603
C	1.5824	3.9261	-0.4241	1.5548	3.9338	-0.4723
C	2.5368	2.9877	0.0593	2.4784	2.9935	0.0856
C	2.1360	1.6754	0.3948	2.0534	1.6679	0.4061
H	-2.2064	2.6971	-0.5902	-2.2180	2.6683	-0.7674
C	-3.3736	0.2723	-0.0795	-3.3318	0.2371	-0.1359
H	-0.4866	4.3213	-0.7893	-0.4795	4.3150	-0.9919
H	1.9174	4.9365	-0.6504	1.9084	4.9401	-0.6851

C	3.9012	3.3782	0.2048	3.8256	3.3629	0.2784
C	3.0583	0.8656	1.1636	2.9436	0.8549	1.2153
C	4.4216	1.2602	1.2429	4.3058	1.2423	1.3487
C	4.8255	2.5187	0.7070	4.7317	2.4910	0.8281
H	4.1856	4.3773	-0.1205	4.1400	4.3538	-0.0429
H	5.8745	2.8015	0.7755	5.7774	2.7735	0.9331
C	-0.4143	-2.7871	0.1230	-0.3662	-2.8227	0.0358
C	-1.3615	-1.7958	0.0694	-1.3529	-1.8398	-0.0241
C	0.9352	-2.5259	-0.1897	0.9652	-2.5345	-0.2645
C	1.3287	-1.2093	-0.5204	1.3550	-1.2017	-0.6097
C	1.8926	-3.5867	-0.1864	1.9643	-3.5546	-0.1914
C	3.1804	-3.3595	-0.5481	3.2575	-3.2966	-0.5143
C	3.5637	-2.0950	-1.0897	3.6389	-2.0322	-1.0674
C	2.6161	-1.0370	-1.1583	2.6670	-0.9982	-1.1865
H	-0.7333	-3.8043	0.3421	-0.6640	-3.8400	0.2798
C	-2.7781	-2.1672	0.2474	-2.7436	-2.2164	0.1399
H	1.5628	-4.5731	0.1340	1.6607	-4.5431	0.1493
H	3.9239	-4.1532	-0.5045	4.0181	-4.0708	-0.4252
C	4.8567	-1.9086	-1.6223	4.9460	-1.8277	-1.5457
C	2.9621	0.1053	-1.9146	3.0330	0.1491	-1.9236
C	4.2172	0.2499	-2.4606	4.3138	0.3183	-2.4101
C	5.1903	-0.7479	-2.2802	5.2942	-0.6599	-2.1929
H	5.5768	-2.7198	-1.5288	5.6748	-2.6266	-1.4154
H	6.1872	-0.6186	-2.6938	6.3066	-0.5141	-2.5608
H	4.4520	1.1395	-3.0400	4.5579	1.2178	-2.9711
H	2.2184	0.8780	-2.0863	2.2876	0.9139	-2.1267
C	5.3509	0.4357	1.9135	5.2030	0.4177	2.0607
C	2.6624	-0.2676	1.9091	2.5078	-0.2679	1.9402
C	4.9457	-0.7078	2.5602	4.7614	-0.7231	2.6942
C	3.5797	-1.0375	2.5880	3.3972	-1.0455	2.6596
H	6.3951	0.7440	1.9331	6.2471	0.7191	2.1228
H	5.6688	-1.3313	3.0800	5.4568	-1.3493	3.2464
H	3.2407	-1.9017	3.1544	3.0294	-1.9097	3.2071
H	1.6096	-0.5281	1.9665	1.4516	-0.5224	1.9524
O	-3.1129	-3.3272	0.4748	-3.0970	-3.3974	0.3232
C	-6.1198	-0.4472	0.1474	-6.0731	-0.4567	0.1677
C	-5.7342	0.9100	-0.0633	-5.6886	0.9069	-0.0295
C	-4.3653	1.2315	-0.1666	-4.3285	1.2163	-0.1739
H	-4.1144	2.2798	-0.3132	-4.0686	2.2644	-0.3024
H	-6.4507	2.9352	-0.3178	-6.3924	2.9442	-0.2185
C	-8.0683	1.5564	-0.0500	-8.0163	1.5760	0.0742

7A

	E(S0) =	-1803.2723	A.U.	E(S1) =	-1803.1868	A.U.
	x	y	z	x	y	z
H	0.0495	-2.0237	1.6863	0.0751	-2.0569	1.7127
H	0.9739	-4.2619	1.9695	0.9871	-4.3213	1.8819

H	4.9437	-2.6315	2.1329	4.9792	-2.7342	1.9622
H	-0.0495	5.0961	-2.0613	-0.3735	5.1032	-1.9275
C	0.3564	4.0934	-1.9528	0.0875	4.1220	-1.8484
C	-0.4674	2.9722	-2.1511	-0.6848	2.9630	-2.0407
H	-6.7252	1.3142	0.9963	-6.6951	1.2228	1.0093
H	-8.2827	-0.4518	0.1478	-8.2179	-0.5187	0.0614
H	-7.3276	-2.4956	-0.9055	-7.2308	-2.5205	-1.0497
C	-6.3500	0.4050	0.5338	-6.3016	0.3338	0.5225
C	-4.9636	0.2537	0.4295	-4.9089	0.2045	0.4517
C	-2.3638	-2.1507	-0.8455	-2.2716	-2.2133	-0.8225
C	-2.9536	-1.0464	-0.2795	-2.8972	-1.0719	-0.2604
C	-0.9720	-2.2107	-1.1002	-0.9076	-2.2592	-1.0673
C	-2.0969	-0.0334	0.2687	-2.0780	-0.0562	0.3038
C	-0.1488	-1.1198	-0.7441	-0.1020	-1.1218	-0.7226
C	-0.6919	-0.1711	0.1975	-0.6628	-0.1897	0.2452
C	-0.3943	-3.3657	-1.7006	-0.2825	-3.3851	-1.6964
C	0.9506	-3.4284	-1.9000	1.0428	-3.3598	-1.9839
C	1.7667	-2.2772	-1.7080	1.8086	-2.1638	-1.8024
C	1.1787	-1.0536	-1.3025	1.1636	-0.9724	-1.3332
H	-2.9603	-2.9990	-1.1736	-2.8670	-3.0740	-1.1179
C	-4.4102	-0.8996	-0.1626	-4.3354	-0.9365	-0.1713
H	-1.0401	-4.2094	-1.9362	-0.8880	-4.2657	-1.9029
H	1.4220	-4.3411	-2.2595	1.5480	-4.2360	-2.3856
C	3.1714	-2.3508	-1.9085	3.1990	-2.1590	-1.9813
C	1.9528	0.1537	-1.4565	1.8922	0.2807	-1.4507
C	3.3571	0.0300	-1.5380	3.3060	0.2213	-1.5294
C	3.9504	-1.2485	-1.7267	3.9424	-1.0164	-1.7502
H	3.6098	-3.3140	-2.1633	3.6923	-3.0904	-2.2531
H	5.0350	-1.3114	-1.7972	5.0285	-1.0424	-1.8165
C	-1.8173	2.1080	1.3659	-1.7950	2.0394	1.5047
C	-2.6512	1.1286	0.8860	-2.6424	1.0794	0.9685
C	-0.4248	1.9045	1.4585	-0.4136	1.8248	1.5840
C	0.1245	0.6641	1.0456	0.1414	0.5888	1.1180
C	0.4262	2.9290	1.9610	0.4734	2.8402	2.0278
C	1.7651	2.7137	2.0613	1.8270	2.6330	2.0474
C	2.3006	1.4090	1.8671	2.3711	1.3402	1.8188
C	1.4577	0.3405	1.4875	1.5062	0.2719	1.4766
H	-2.2540	3.0346	1.7340	-2.2284	2.9613	1.8876
C	-4.1085	1.3381	0.9521	-4.0852	1.2716	1.0353
H	-0.0151	3.8898	2.2177	0.0563	3.8074	2.3025
H	2.4407	3.5106	2.3667	2.5055	3.4388	2.3217
C	3.6904	1.1778	2.0902	3.7661	1.1016	1.9851
C	1.9622	-1.0088	1.6532	2.0025	-1.0703	1.6270
C	3.3551	-1.2034	1.8637	3.4037	-1.2791	1.7800
C	4.2116	-0.0742	2.0219	4.2755	-0.1574	1.9048
H	4.3241	2.0367	2.3046	4.4138	1.9568	2.1703

H	5.2776	-0.2452	2.1631	5.3445	-0.3368	2.0041
C	3.8696	-2.5108	1.9990	3.9033	-2.5940	1.8694
C	1.1277	-2.1469	1.7332	1.1523	-2.2004	1.7013
C	4.1836	1.1914	-1.4667	4.0754	1.4217	-1.4386
C	1.3824	1.4758	-1.6273	1.2585	1.5602	-1.6051
C	3.6414	2.4341	-1.3974	3.4697	2.6352	-1.3728
C	2.2328	2.6124	-1.5364	2.0537	2.7433	-1.5083
H	5.2633	1.0509	-1.4558	5.1604	1.3346	-1.4199
H	4.2724	3.3173	-1.3126	4.0540	3.5490	-1.2808
C	1.6883	3.9084	-1.6659	1.4373	4.0066	-1.6024
C	0.0351	1.7001	-1.9935	-0.1135	1.7174	-1.9249
O	-4.5902	2.3660	1.4241	-4.5930	2.2740	1.5667
C	-7.2061	-0.5728	0.0631	-7.1386	-0.6312	-0.0033
C	-6.6684	-1.7185	-0.5264	-6.5832	-1.7572	-0.6252
C	-5.2973	-1.8766	-0.6391	-5.2125	-1.9028	-0.7079
H	-4.9174	-2.7775	-1.1123	-4.8136	-2.7795	-1.2105
H	-0.6154	0.8504	-2.1808	-0.7196	0.8371	-2.1166
H	2.3573	4.7618	-1.5643	2.0594	4.8943	-1.4999
C	3.0353	-3.6036	2.0066	3.0534	-3.6776	1.8721
H	3.4409	-4.6052	2.1252	3.4523	-4.6855	1.9543
H	-1.5077	3.1058	-2.4386	-1.7394	3.0487	-2.2911
C	1.6472	-3.4103	1.9049	1.6639	-3.4729	1.8170

8A

	E(S0) =	-1803.2723	A.U.	E(S1) =	-1803.1868	A.U.
	x	y	z	x	y	z
H	0.0495	-2.0237	1.6863	0.0751	-2.0569	1.7127
H	0.9739	-4.2619	1.9695	0.9871	-4.3213	1.8819
H	4.9437	-2.6315	2.1329	4.9792	-2.7342	1.9622
H	-0.0495	5.0961	-2.0613	-0.3735	5.1032	-1.9275
C	0.3564	4.0934	-1.9528	0.0875	4.1220	-1.8484
C	-0.4674	2.9722	-2.1511	-0.6848	2.9630	-2.0407
H	-6.7252	1.3142	0.9963	-6.6951	1.2228	1.0093
H	-8.2827	-0.4518	0.1478	-8.2179	-0.5187	0.0614
H	-7.3276	-2.4956	-0.9055	-7.2308	-2.5205	-1.0497
C	-6.3500	0.4050	0.5338	-6.3016	0.3338	0.5225
C	-4.9636	0.2537	0.4295	-4.9089	0.2045	0.4517
C	-2.3638	-2.1507	-0.8455	-2.2716	-2.2133	-0.8225
C	-2.9536	-1.0464	-0.2795	-2.8972	-1.0719	-0.2604
C	-0.9720	-2.2107	-1.1002	-0.9076	-2.2592	-1.0673
C	-2.0969	-0.0334	0.2687	-2.0780	-0.0562	0.3038
C	-0.1488	-1.1198	-0.7441	-0.1020	-1.1218	-0.7226
C	-0.6919	-0.1711	0.1975	-0.6628	-0.1897	0.2452
C	-0.3943	-3.3657	-1.7006	-0.2825	-3.3851	-1.6964
C	0.9506	-3.4284	-1.9000	1.0428	-3.3598	-1.9839
C	1.7667	-2.2772	-1.7080	1.8086	-2.1638	-1.8024
C	1.1787	-1.0536	-1.3025	1.1636	-0.9724	-1.3332

H	-2.9603	-2.9990	-1.1736	-2.8670	-3.0740	-1.1179
C	-4.4102	-0.8996	-0.1626	-4.3354	-0.9365	-0.1713
H	-1.0401	-4.2094	-1.9362	-0.8880	-4.2657	-1.9029
H	1.4220	-4.3411	-2.2595	1.5480	-4.2360	-2.3856
C	3.1714	-2.3508	-1.9085	3.1990	-2.1590	-1.9813
C	1.9528	0.1537	-1.4565	1.8922	0.2807	-1.4507
C	3.3571	0.0300	-1.5380	3.3060	0.2213	-1.5294
C	3.9504	-1.2485	-1.7267	3.9424	-1.0164	-1.7502
H	3.6098	-3.3140	-2.1633	3.6923	-3.0904	-2.2531
H	5.0350	-1.3114	-1.7972	5.0285	-1.0424	-1.8165
C	-1.8173	2.1080	1.3659	-1.7950	2.0394	1.5047
C	-2.6512	1.1286	0.8860	-2.6424	1.0794	0.9685
C	-0.4248	1.9045	1.4585	-0.4136	1.8248	1.5840
C	0.1245	0.6641	1.0456	0.1414	0.5888	1.1180
C	0.4262	2.9290	1.9610	0.4734	2.8402	2.0278
C	1.7651	2.7137	2.0613	1.8270	2.6330	2.0474
C	2.3006	1.4090	1.8671	2.3711	1.3402	1.8188
C	1.4577	0.3405	1.4875	1.5062	0.2719	1.4766
H	-2.2540	3.0346	1.7340	-2.2284	2.9613	1.8876
C	-4.1085	1.3381	0.9521	-4.0852	1.2716	1.0353
H	-0.0151	3.8898	2.2177	0.0563	3.8074	2.3025
H	2.4407	3.5106	2.3667	2.5055	3.4388	2.3217
C	3.6904	1.1778	2.0902	3.7661	1.1016	1.9851
C	1.9622	-1.0088	1.6532	2.0025	-1.0703	1.6270
C	3.3551	-1.2034	1.8637	3.4037	-1.2791	1.7800
C	4.2116	-0.0742	2.0219	4.2755	-0.1574	1.9048
H	4.3241	2.0367	2.3046	4.4138	1.9568	2.1703
H	5.2776	-0.2452	2.1631	5.3445	-0.3368	2.0041
C	3.8696	-2.5108	1.9990	3.9033	-2.5940	1.8694
C	1.1277	-2.1469	1.7332	1.1523	-2.2004	1.7013
C	4.1836	1.1914	-1.4667	4.0754	1.4217	-1.4386
C	1.3824	1.4758	-1.6273	1.2585	1.5602	-1.6051
C	3.6414	2.4341	-1.3974	3.4697	2.6352	-1.3728
C	2.2328	2.6124	-1.5364	2.0537	2.7433	-1.5083
H	5.2633	1.0509	-1.4558	5.1604	1.3346	-1.4199
H	4.2724	3.3173	-1.3126	4.0540	3.5490	-1.2808
C	1.6883	3.9084	-1.6659	1.4373	4.0066	-1.6024
C	0.0351	1.7001	-1.9935	-0.1135	1.7174	-1.9249
O	-4.5902	2.3660	1.4241	-4.5930	2.2740	1.5667
C	-7.2061	-0.5728	0.0631	-7.1386	-0.6312	-0.0033
C	-6.6684	-1.7185	-0.5264	-6.5832	-1.7572	-0.6252
C	-5.2973	-1.8766	-0.6391	-5.2125	-1.9028	-0.7079
H	-4.9174	-2.7775	-1.1123	-4.8136	-2.7795	-1.2105
H	-0.6154	0.8504	-2.1808	-0.7196	0.8371	-2.1166
H	2.3573	4.7618	-1.5643	2.0594	4.8943	-1.4999
C	3.0353	-3.6036	2.0066	3.0534	-3.6776	1.8721

H	3.4409	-4.6052	2.1252	3.4523	-4.6855	1.9543
H	-1.5077	3.1058	-2.4386	-1.7394	3.0487	-2.2911
C	1.6472	-3.4103	1.9049	1.6639	-3.4729	1.8170

1F

	E(S0) =	-1342.6669	A.U.	E(S1) =	-1342.5915	A.U.
	x	y	z	x	y	z
H	-4.0261	-2.5580	1.0569	3.9027	2.5747	1.1922
H	-6.4429	-2.3335	1.3067	6.3135	2.3641	1.4947
H	-7.6063	-0.2175	0.7985	7.5129	0.2799	0.9133
O	-3.7078	2.8729	-0.6127	3.7362	-2.8452	-0.7349
C	-4.4865	-1.6124	0.7907	4.3931	1.6532	0.8942
C	-4.4348	0.6785	0.0776	4.3756	-0.6537	0.0667
C	-2.1277	1.0437	-0.2650	2.1472	-1.0220	-0.3637
C	-2.3114	-0.2901	0.0584	2.2930	0.3539	0.0541
C	-0.8422	1.6144	-0.4200	0.8799	-1.5951	-0.5275
C	-1.2130	-1.1856	-0.0220	1.1754	1.2321	-0.0345
C	0.2895	0.7726	-0.2341	-0.2658	-0.7734	-0.2807
C	0.0744	-0.6542	-0.3147	-0.0897	0.6801	-0.3536
C	-0.6663	2.9896	-0.7361	0.7226	-2.9800	-0.8287
C	0.5893	3.5056	-0.8377	-0.5223	-3.5319	-0.8392
C	1.7232	2.7458	-0.4412	-1.6418	-2.7870	-0.3883
C	1.5648	1.4112	-0.0055	-1.4842	-1.4282	0.0334
C	-3.4501	1.7178	-0.3171	3.4550	-1.6747	-0.3996
C	-3.7562	-0.5260	0.3315	3.6677	0.5654	0.3613
H	-1.5497	3.5923	-0.9215	1.6197	-3.5465	-1.0607
H	0.7423	4.5372	-1.1498	-0.6754	-4.5665	-1.1389
C	3.0160	3.3498	-0.4521	-2.9300	-3.3809	-0.3783
C	2.6691	0.8061	0.7143	-2.5856	-0.8201	0.7695
C	3.9510	1.4184	0.6544	-3.8659	-1.4327	0.7214
C	4.1031	2.6849	0.0164	-4.0155	-2.7053	0.0963
H	3.1076	4.3556	-0.8582	-3.0339	-4.3886	-0.7751
H	5.0947	3.1319	-0.0247	-5.0051	-3.1571	0.0686
C	-0.3350	-3.4511	-0.0671	0.2515	3.4755	-0.1492
C	-1.3732	-2.5985	0.1217	1.3052	2.6473	0.0871
C	0.9140	-2.9728	-0.5576	-0.9770	2.9634	-0.6429
C	1.0966	-1.5794	-0.7644	-1.1258	1.5550	-0.8214
C	1.9386	-3.8773	-0.9084	-2.0308	3.8329	-0.9960
C	3.0916	-3.4370	-1.5135	-3.1846	3.3499	-1.5642
C	3.2309	-2.0735	-1.8247	-3.2990	1.9727	-1.8380
C	2.2565	-1.1723	-1.4628	-2.2975	1.1032	-1.4832
H	-0.4636	-4.5222	0.0763	0.3560	4.5517	-0.0252
H	-2.3476	-2.9967	0.3759	2.2665	3.0720	0.3517
H	1.7827	-4.9369	-0.7128	-1.8997	4.8999	-0.8241
H	3.8739	-4.1414	-1.7842	-3.9903	4.0276	-1.8343
H	4.1089	-1.7272	-2.3640	-4.1824	1.5948	-2.3467
H	2.3798	-0.1272	-1.7301	-2.4011	0.0500	-1.7275

C	5.0542	0.7988	1.2808	-4.9635	-0.8169	1.3564
C	2.5318	-0.3383	1.5320	-2.4351	0.3163	1.5849
C	4.8994	-0.3591	2.0050	-4.8000	0.3430	2.0810
C	3.6156	-0.9108	2.1576	-3.5178	0.8926	2.2197
H	6.0300	1.2747	1.1968	-5.9398	-1.2925	1.2823
H	5.7551	-0.8254	2.4868	-5.6507	0.8099	2.5702
H	3.4731	-1.7892	2.7823	-3.3690	1.7739	2.8383
H	1.5476	-0.7722	1.6822	-1.4487	0.7497	1.7235
C	-5.8730	-1.4793	0.9493	5.7537	1.5298	1.0800
C	-6.5300	-0.2887	0.6675	6.4388	0.3350	0.7537
C	-5.7974	0.8188	0.2306	5.7529	-0.7565	0.2559
H	-6.2724	1.7745	0.0198	6.2568	-1.6906	0.0180
			**2F**			
	E(S0) =	-1496.2014	A.U.	E(S1) =	-1496.1199	A.U.
H	-8.1289	-2.3879	1.2205	8.0375	2.4186	1.3448
H	-5.7031	-2.8257	1.0594	5.6113	2.8432	1.1652
H	-9.0311	-0.1256	0.7224	8.9599	0.1722	0.7909
C	-7.9623	-0.3084	0.6488	7.8899	0.3457	0.7084
C	-7.4506	-1.5893	0.9307	7.3644	1.6277	1.0246
H	-3.4586	-2.0635	0.6281	3.3896	2.0690	0.6812
C	-6.0992	-1.8352	0.8417	6.0224	1.8638	0.9260
C	-7.1109	0.7088	0.2831	7.0609	-0.6673	0.3049
O	-2.4898	3.3626	-0.8124	2.4922	-3.3818	-0.9193
C	-3.7990	-1.0650	0.3679	3.7507	1.0834	0.3984
C	-3.4873	1.2381	-0.2572	3.4394	-1.2451	-0.2975
C	-1.1439	1.3593	-0.4456	1.1531	-1.3701	-0.5436
C	-1.4821	0.0386	-0.1916	1.4762	-0.0012	-0.2192
C	0.2027	1.7985	-0.4906	-0.1776	-1.8046	-0.5866
C	-0.4740	-0.9630	-0.2493	0.4585	0.9923	-0.2815
C	1.2261	0.8387	-0.2648	-1.2062	-0.8465	-0.3067
C	0.8758	-0.5558	-0.4297	-0.8811	0.5725	-0.4777
C	0.5382	3.1591	-0.7274	-0.5080	-3.1761	-0.7989
C	1.8437	3.5470	-0.7139	-1.8012	-3.5843	-0.6858
C	2.8617	2.6589	-0.2747	-2.7947	-2.6967	-0.1961
C	2.5370	1.3309	0.0837	-2.4583	-1.3446	0.1339
C	-2.3773	2.1750	-0.5495	2.3727	-2.1728	-0.6349
C	-2.9490	-0.0608	-0.0096	2.8865	0.0692	-0.0201
H	-0.2644	3.8563	-0.9458	0.3015	-3.8492	-1.0649
H	2.1223	4.5690	-0.9647	-2.0894	-4.6083	-0.9143
C	4.2070	3.1233	-0.1669	-4.1332	-3.1406	-0.0600
C	3.5178	0.5838	0.8480	-3.4282	-0.5801	0.9092
C	4.8561	1.0596	0.9063	-4.7672	-1.0477	0.9890
C	5.1829	2.3287	0.3424	-5.1006	-2.3243	0.4521
H	4.4305	4.1296	-0.5166	-4.3765	-4.1485	-0.3896
H	6.2154	2.6701	0.3912	-6.1322	-2.6641	0.5211

C	0.1691	-3.3028	-0.3671	-0.2241	3.3169	-0.4628
C	-0.7882	-2.3554	-0.1991	0.7487	2.3886	-0.2521
C	1.4922	-2.9348	-0.7430	-1.5356	2.9234	-0.8374
C	1.8299	-1.5597	-0.8584	-1.8496	1.5333	-0.9203
C	2.4428	-3.9242	-1.0739	-2.5113	3.8878	-1.1668
C	3.6757	-3.5788	-1.5732	-3.7525	3.5112	-1.6206
C	3.9756	-2.2235	-1.7962	-4.0374	2.1433	-1.8007
C	3.0738	-1.2435	-1.4518	-3.1123	1.1847	-1.4674
H	-0.0780	-4.3603	-0.2965	0.0064	4.3795	-0.4104
H	-1.8153	-2.6601	-0.0407	1.7675	2.7169	-0.0822
H	2.1655	-4.9696	-0.9496	-2.2501	4.9403	-1.0699
H	4.4001	-4.3475	-1.8296	-4.4976	4.2611	-1.8729
H	4.9217	-1.9457	-2.2540	-4.9939	1.8420	-2.2208
H	3.3222	-0.2055	-1.6509	-3.3497	0.1392	-1.6410
C	5.8409	0.3022	1.5767	-5.7404	-0.2825	1.6642
C	3.2036	-0.5716	1.5981	-3.0944	0.5717	1.6436
C	5.5153	-0.8619	2.2312	-5.3977	0.8869	2.3061
C	4.1742	-1.2810	2.2679	-4.0571	1.2964	2.3193
H	6.8638	0.6755	1.5838	-6.7647	-0.6498	1.6879
H	6.2810	-1.4355	2.7473	-6.1527	1.4701	2.8263
H	3.8971	-2.1645	2.8378	-3.7670	2.1865	2.8716
H	2.1716	-0.9051	1.6579	-2.0598	0.9005	1.6836
C	-5.2009	-0.8135	0.4656	5.1251	0.8400	0.5041
C	-5.7216	0.4846	0.1836	5.6624	-0.4644	0.1892
C	-4.8156	1.5200	-0.1749	4.7887	-1.4939	-0.2025
H	-5.1797	2.5281	-0.3704	5.1748	-2.4891	-0.4194
H	-7.4952	1.7038	0.0657	7.4620	-1.6506	0.0661

3F

	E(S0) =	-1342.6670	A.U.	E(S1) =	-1342.5906	A.U.
	x	y	z	x	y	z
H	4.0025	-2.8332	-0.3991	3.9970	-2.8326	-0.5273
H	6.4255	-2.7745	-0.1193	6.4140	-2.7474	-0.2618
H	7.6146	-0.6849	0.4365	7.5761	-0.6567	0.3708
O	3.7521	2.7315	0.7624	3.7243	2.7468	0.8622
C	4.4734	-1.8807	-0.1812	4.4618	-1.8865	-0.2709
C	4.4486	0.4714	0.2943	4.3960	0.4893	0.3170
C	2.1474	0.9867	0.1696	2.1530	0.9840	0.2199
C	2.3086	-0.3550	-0.1344	2.3133	-0.4044	-0.1517
C	0.8931	1.6349	0.0725	0.9033	1.6126	0.1425
C	1.1733	-1.1641	-0.3917	1.1686	-1.2113	-0.4000
C	-0.2424	0.8569	-0.2606	-0.2221	0.8291	-0.2441
C	-0.1228	-0.5819	-0.2333	-0.1194	-0.6274	-0.2045
C	0.7657	3.0411	0.2925	0.7592	3.0207	0.3608
C	-0.4287	3.6607	0.1144	-0.4227	3.6349	0.1036
C	-1.5440	2.9550	-0.4267	-1.4991	2.9186	-0.5013
C	-1.4364	1.5624	-0.6887	-1.3698	1.5178	-0.7516

C	3.4776	1.5833	0.4545	3.4551	1.5770	0.5141
C	3.7541	-0.7022	-0.0446	3.7050	-0.7108	-0.0763
H	1.6470	3.5871	0.6140	1.6300	3.5561	0.7272
H	-0.5373	4.7252	0.3151	-0.5528	4.6986	0.2942
C	-2.7275	3.6451	-0.7646	-2.6799	3.5823	-0.8789
C	-2.4828	0.9565	-1.4198	-2.3914	0.8838	-1.5062
C	-3.6186	1.6541	-1.7638	-3.5234	1.5626	-1.8872
C	-3.7623	3.0046	-1.4044	-3.6878	2.9164	-1.5430
H	-2.7919	4.7061	-0.5283	-2.7747	4.6424	-0.6510
H	-4.6688	3.5458	-1.6631	-4.5928	3.4445	-1.8318
C	0.1613	-3.2750	-1.0046	0.1126	-3.3325	-0.9111
C	1.2759	-2.5196	-0.8146	1.2433	-2.5706	-0.7956
C	-1.1098	-2.7975	-0.5871	-1.1365	-2.8347	-0.4856
C	-1.2370	-1.4890	-0.0716	-1.2315	-1.4955	-0.0046
C	-2.2481	-3.6527	-0.6706	-2.3028	-3.6485	-0.5622
C	-3.4553	-3.2559	-0.1919	-3.5048	-3.1938	-0.1202
C	-3.5787	-2.0279	0.5221	-3.6023	-1.9402	0.5578
C	-2.4538	-1.1674	0.6493	-2.4515	-1.1187	0.6938
H	0.2339	-4.2831	-1.4074	0.1693	-4.3501	-1.2920
H	2.2493	-2.9272	-1.0593	2.2005	-2.9938	-1.0766
H	-2.1240	-4.6278	-1.1378	-2.2073	-4.6401	-1.0003
H	-4.3321	-3.8941	-0.2856	-4.4005	-3.8052	-0.2116
C	-4.7955	-1.6910	1.1537	-4.8165	-1.5438	1.1539
C	-2.5701	-0.0691	1.5304	-2.5367	-0.0067	1.5557
C	-3.7584	0.2262	2.1583	-3.7288	0.3527	2.1484
C	-4.8958	-0.5714	1.9445	-4.8891	-0.4030	1.9218
H	-5.6485	-2.3540	1.0188	-5.6929	-2.1744	1.0141
H	-5.8363	-0.3227	2.4294	-5.8299	-0.1090	2.3798
H	-3.8122	1.0777	2.8321	-3.7632	1.2183	2.8049
H	-1.7019	0.5533	1.7260	-1.6452	0.5785	1.7658
H	-4.4033	1.1563	-2.3284	-4.2880	1.0543	-2.4686
H	-2.3897	-0.0808	-1.7277	-2.2688	-0.1541	-1.8017
C	5.8640	-1.8501	-0.0076	5.8308	-1.8427	-0.1108
C	6.5357	-0.6762	0.3084	6.4951	-0.6482	0.2556
C	5.8149	0.5101	0.4721	5.7803	0.5133	0.4770
H	6.3021	1.4461	0.7367	6.2673	1.4395	0.7735

4F

	E(S0) =	-1496.1949	A.U.	E(S1) =	-1496.1212	A.U.
	x	y	z	x	y	z
H	0.4817	-2.3498	2.9471	0.2879	-2.3865	2.8588
H	2.6204	-3.6366	2.9407	2.4121	-3.6901	2.9031
H	4.6618	-2.5946	1.9937	4.5020	-2.6541	2.0678
C	1.3641	-1.9372	2.4635	1.1912	-1.9686	2.4214
C	2.5722	-2.6555	2.4749	2.3926	-2.6972	2.4612
H	-4.0222	-2.8777	0.2937	-3.8712	-2.9027	0.4271
H	-6.4327	-2.9474	0.6408	-6.2729	-2.9976	0.8216

H	-7.7960	-0.8908	0.6884	-7.6733	-0.9582	0.7851
O	-4.2497	2.8013	-0.0285	-4.2594	2.8055	-0.1586
C	-4.5693	-1.9413	0.2826	-4.4482	-1.9850	0.3728
C	-4.7468	0.4471	0.1457	-4.6661	0.4376	0.1204
C	-2.5008	1.1033	-0.1640	-2.4988	1.1112	-0.2658
C	-2.5477	-0.2812	-0.1468	-2.5019	-0.3306	-0.1734
C	-1.2808	1.8192	-0.2245	-1.3002	1.8323	-0.3327
C	-1.3740	-1.0263	-0.4384	-1.3167	-1.0563	-0.4771
C	-0.0744	1.0729	-0.2747	-0.0778	1.0888	-0.3155
C	-0.1609	-0.3164	-0.6553	-0.1241	-0.3258	-0.7038
C	-1.2414	3.2425	-0.2328	-1.2747	3.2591	-0.3189
C	-0.0429	3.8830	-0.3038	-0.0811	3.9103	-0.2746
C	1.1793	3.1682	-0.1629	1.1275	3.1915	-0.0690
C	1.1589	1.7654	0.0367	1.0989	1.7677	0.0957
C	-3.8774	1.6395	-0.0202	-3.8600	1.6240	-0.1143
C	-3.9540	-0.7125	0.0921	-3.8425	-0.7397	0.1035
H	-2.1820	3.7839	-0.2343	-2.2269	3.7774	-0.3789
H	0.0038	4.9654	-0.4099	-0.0282	4.9938	-0.3604
C	2.4194	3.8596	-0.2201	2.3711	3.8573	-0.0930
C	2.3674	1.1380	0.5162	2.3040	1.1190	0.5847
C	3.5899	1.8153	0.3109	3.5352	1.7888	0.4105
C	3.5920	3.1810	-0.0837	3.5426	3.1594	0.0490
H	2.4079	4.9292	-0.4205	2.3827	4.9294	-0.2775
H	4.5489	3.6840	-0.2101	4.5013	3.6649	-0.0491
C	-0.2564	-3.1376	-0.8870	-0.1699	-3.1245	-1.0338
C	-1.3765	-2.4515	-0.5449	-1.2963	-2.4743	-0.6327
C	0.9150	-2.4463	-1.3100	0.9837	-2.4000	-1.4349
C	0.9348	-1.0270	-1.2811	0.9758	-0.9745	-1.3488
C	2.0190	-3.1548	-1.8293	2.1135	-3.0649	-1.9534
C	3.0849	-2.4885	-2.3865	3.1879	-2.3582	-2.4412
C	3.0532	-1.0860	-2.4755	3.1400	-0.9513	-2.4654
C	2.0035	-0.3759	-1.9388	2.0636	-0.2782	-1.9396
H	-0.2618	-4.2253	-0.9232	-0.1575	-4.2099	-1.1140
H	-2.2897	-2.9963	-0.3416	-2.1972	-3.0442	-0.4384
H	1.9917	-4.2430	-1.8095	2.1062	-4.1533	-1.9796
H	3.9277	-3.0420	-2.7926	4.0526	-2.8804	-2.8426
H	3.8581	-0.5559	-2.9789	3.9562	-0.3900	-2.9140
H	1.9901	0.7051	-2.0413	2.0337	0.8054	-2.0052
C	4.8285	1.1320	0.5028	4.7629	1.0951	0.6278
C	2.4123	-0.1304	1.2137	2.3115	-0.1435	1.2685
C	4.8583	-0.1577	0.9272	4.7662	-0.1967	1.0494
C	3.6566	-0.8061	1.3399	3.5476	-0.8362	1.4218
H	5.7490	1.6673	0.2762	5.6939	1.6224	0.4293
H	5.8007	-0.6928	1.0298	5.6992	-0.7431	1.1733
C	3.7016	-2.0839	1.9382	3.5530	-2.1267	1.9881
C	1.2902	-0.7066	1.8510	1.1528	-0.7211	1.8421

C	-5.9546	-1.9820	0.4941	-5.8068	-2.0385	0.6101
C	-6.7238	-0.8263	0.5241	-6.6063	-0.8729	0.5948
C	-6.1090	0.4173	0.3530	-6.0374	0.3644	0.3570
H	-6.6739	1.3465	0.3847	-6.6283	1.2777	0.3582
H	0.3524	-0.1594	1.8778	0.2264	-0.1542	1.8582

5F

	E(S0) =	-1496.1949	A.U.	E(S1) =	-1496.1214	A.U.
	x	y	z	x	y	z
H	4.4565	-2.7827	-0.5161	4.3776	-2.7601	-0.7984
H	6.8890	-2.6601	-0.4891	6.8068	-2.6318	-0.8368
H	8.0768	-0.5325	-0.0991	8.0046	-0.5388	-0.2834
O	4.1861	2.8094	0.5202	4.1795	2.7921	0.7003
C	4.9191	-1.8165	-0.3472	4.8557	-1.8239	-0.5285
C	4.8845	0.5436	0.0798	4.8233	0.5343	0.1186
C	2.5732	1.0142	0.1491	2.5752	0.9962	0.2745
C	2.7304	-0.3412	-0.0884	2.7136	-0.4058	-0.0476
C	1.3044	1.6438	0.1391	1.3214	1.6208	0.2652
C	1.5881	-1.1838	-0.1405	1.5623	-1.2408	-0.0593
C	0.1603	0.8363	-0.0915	0.1777	0.8161	-0.0310
C	0.3057	-0.5948	0.0744	0.2924	-0.6337	0.1664
C	1.1570	3.0426	0.3577	1.1799	3.0290	0.4462
C	-0.0834	3.6031	0.3375	-0.0429	3.6062	0.2937
C	-1.2162	2.8564	-0.0897	-1.1419	2.8537	-0.2013
C	-1.0717	1.4986	-0.4466	-0.9876	1.4679	-0.5089
C	3.9099	1.6441	0.2866	3.8940	1.6112	0.4119
C	4.1864	-0.6557	-0.1451	4.1103	-0.6792	-0.1758
H	2.0454	3.6260	0.5776	2.0668	3.5907	0.7236
H	-0.2210	4.6548	0.5829	-0.1935	4.6637	0.5014
C	-2.4953	3.4841	-0.1606	-2.4145	3.4641	-0.3318
C	-2.1603	0.8735	-1.1674	-2.0589	0.8144	-1.2464
C	-3.4355	1.5018	-1.1700	-3.3306	1.4441	-1.3118
C	-3.5828	2.8055	-0.6107	-3.4890	2.7637	-0.7992
H	-2.5802	4.5146	0.1799	-2.5217	4.5013	-0.0210
H	-4.5679	3.2685	-0.6211	-4.4717	3.2283	-0.8546
C	0.5494	-3.3570	-0.3836	0.4924	-3.4119	-0.1433
C	1.6686	-2.5837	-0.3894	1.6242	-2.6513	-0.2152
C	-0.6915	-2.8299	0.0652	-0.7386	-2.8426	0.2557
C	-0.7834	-1.4708	0.4337	-0.8044	-1.4535	0.5561
C	-1.8381	-3.6743	0.1392	-1.9161	-3.6398	0.3096
C	-3.0226	-3.2008	0.6063	-3.1082	-3.1047	0.6886
C	-3.1043	-1.8946	1.1726	-3.1743	-1.7730	1.1989
C	-1.9617	-1.0490	1.1632	-2.0050	-0.9664	1.2170
H	0.6007	-4.4096	-0.6551	0.5313	-4.4805	-0.3456
H	2.6232	-3.0274	-0.6428	2.5745	-3.1294	-0.4198
H	-1.7437	-4.7007	-0.2105	-1.8431	-4.6830	0.0079
H	-3.9101	-3.8310	0.6216	-4.0162	-3.7049	0.6839

C	-4.2915	-1.4619	1.8025	-4.3715	-1.2812	1.7591
C	-2.0176	0.1316	1.9377	-2.0524	0.2381	1.9479
C	-3.1754	0.5181	2.5739	-3.2264	0.6893	2.5144
C	-4.3388	-0.2645	2.4760	-4.4076	-0.0583	2.3906
H	-5.1625	-2.1144	1.7678	-5.2621	-1.9055	1.7083
H	-5.2558	0.0568	2.9633	-5.3340	0.3082	2.8254
H	-3.1841	1.4322	3.1629	-3.2295	1.6239	3.0698
H	-1.1257	0.7414	2.0490	-1.1439	0.8187	2.0850
C	-4.5299	0.8609	-1.7907	-4.4052	0.7925	-1.9524
C	-2.0091	-0.3002	-1.9399	-1.8836	-0.3804	-1.9693
C	-4.3664	-0.3266	-2.4637	-4.2225	-0.4195	-2.5797
C	-3.0823	-0.8886	-2.5696	-2.9419	-0.9903	-2.6139
H	-5.5036	1.3471	-1.7514	-5.3765	1.2838	-1.9648
H	-5.2143	-0.8072	-2.9454	-5.0541	-0.9128	-3.0757
H	-2.9308	-1.7896	-3.1594	-2.7742	-1.9163	-3.1584
H	-1.0222	-0.7384	-2.0587	-0.8962	-0.8300	-2.0325
C	6.3193	-1.7482	-0.3283	6.2334	-1.7507	-0.5603
C	6.9905	-0.5527	-0.1083	6.9181	-0.5538	-0.2481
C	6.2603	0.6201	0.1034	6.2157	0.5883	0.0869
H	6.7479	1.5757	0.2840	6.7189	1.5244	0.3187
			**6F**			
	E(S0) =	-1649.7293	A.U.	E(S1) =	-1649.6499	A.U.
	x	y	z	x	y	z
H	-8.6160	-2.6590	0.4426	-8.5647	-2.6735	0.5334
H	-6.1731	-3.0165	0.5070	-6.1223	-3.0292	0.5898
H	-9.5415	-0.3810	0.0758	-9.4910	-0.3990	0.1197
C	-8.4650	-0.5280	0.1030	-8.4135	-0.5416	0.1426
C	-7.9402	-1.8178	0.3107	-7.8858	-1.8389	0.3786
H	-3.9301	-2.1653	0.3691	-3.8930	-2.1748	0.4164
C	-6.5790	-2.0190	0.3471	-6.5338	-2.0373	0.4102
C	-7.6170	0.5423	-0.0653	-7.5764	0.5241	-0.0557
O	-3.0115	3.3879	-0.5339	-2.9978	3.4127	-0.6115
C	-4.2708	-1.1458	0.2153	-4.2414	-1.1611	0.2404
C	-3.9775	1.2117	-0.1587	-3.9287	1.2214	-0.1973
C	-1.6323	1.4053	-0.1798	-1.6332	1.4176	-0.2449
C	-1.9453	0.0695	0.0272	-1.9400	0.0301	0.0103
C	-0.2984	1.8861	-0.1380	-0.3145	1.8890	-0.2091
C	-0.9028	-0.8983	0.0767	-0.8947	-0.9304	0.0497
C	0.7416	0.9501	0.0910	0.7270	0.9443	0.0591
C	0.4383	-0.4530	-0.1113	0.4411	-0.4768	-0.1676
C	0.0100	3.2632	-0.3193	-0.0061	3.2750	-0.3526
C	1.3059	3.6789	-0.2647	1.2766	3.6977	-0.1917
C	2.3378	2.7989	0.1618	2.2800	2.8055	0.2762
C	2.0332	1.4579	0.4820	1.9609	1.4405	0.5515
C	-2.8792	2.1915	-0.3237	-2.8634	2.1942	-0.3850
C	-3.4200	-0.0854	0.0546	-3.3666	-0.0930	0.0460

H	-0.8025	3.9483	-0.5388	-0.8198	3.9454	-0.6122
H	1.5662	4.7134	-0.4819	1.5524	4.7346	-0.3731
C	3.6789	3.2741	0.2674	3.6162	3.2537	0.4073
C	3.0311	0.6951	1.2026	2.9502	0.6460	1.2661
C	4.3689	1.1741	1.2410	4.2901	1.1161	1.3329
C	4.6734	2.4655	0.7172	4.6030	2.4169	0.8480
H	3.8869	4.2956	-0.0462	3.8465	4.2774	0.1192
H	5.7045	2.8126	0.7544	5.6347	2.7589	0.9040
C	-0.1163	-3.1786	0.2792	-0.0764	-3.2092	0.1596
C	-1.1426	-2.2845	0.2922	-1.1112	-2.3223	0.2415
C	1.1805	-2.7839	-0.1443	1.2043	-2.7925	-0.2716
C	1.4276	-1.4355	-0.4795	1.4308	-1.4182	-0.5660
C	2.2291	-3.7476	-0.2226	2.2756	-3.7242	-0.3479
C	3.4658	-3.3970	-0.6617	3.5189	-3.3350	-0.7422
C	3.6996	-2.0956	-1.1967	3.7395	-2.0149	-1.2378
C	2.6565	-1.1302	-1.1834	2.6765	-1.0715	-1.2322
H	-0.2884	-4.2242	0.5265	-0.2331	-4.2619	0.3871
H	-2.1433	-2.6244	0.5276	-2.0974	-2.6817	0.5096
H	2.0173	-4.7646	0.1021	2.0814	-4.7536	-0.0520
H	4.2793	-4.1203	-0.6788	4.3471	-4.0411	-0.7571
C	4.9366	-1.7822	-1.8003	4.9829	-1.6615	-1.8019
C	2.8529	0.0535	-1.9296	2.8644	0.1306	-1.9441
C	4.0559	0.3244	-2.5412	4.0807	0.4471	-2.5129
C	5.1249	-0.5830	-2.4457	5.1626	-0.4412	-2.4134
H	5.7303	-2.5269	-1.7696	5.7920	-2.3894	-1.7691
H	6.0792	-0.3540	-2.9133	6.1237	-0.1823	-2.8504
H	4.1739	1.2445	-3.1086	4.1937	1.3846	-3.0519
H	2.0344	0.7593	-2.0392	2.0320	0.8200	-2.0608
C	5.3723	0.3989	1.8619	5.2831	0.3262	1.9499
C	2.7339	-0.4720	1.9418	2.6370	-0.5318	1.9686
C	5.0632	-0.7782	2.5015	4.9592	-0.8665	2.5563
C	3.7222	-1.1934	2.5727	3.6188	-1.2774	2.5926
H	6.3952	0.7722	1.8501	6.3068	0.6961	1.9614
H	5.8424	-1.3632	2.9836	5.7281	-1.4672	3.0347
H	3.4591	-2.0858	3.1358	3.3436	-2.1863	3.1219
H	1.7018	-0.7988	2.0330	1.6032	-0.8601	2.0330
C	-5.6833	-0.9411	0.1788	-5.6283	-0.9565	0.2096
C	-6.2178	0.3647	-0.0318	-6.1680	0.3604	-0.0306
C	-5.3159	1.4497	-0.2046	-5.2860	1.4361	-0.2344
H	-5.6913	2.4584	-0.3732	-5.6718	2.4373	-0.4221
H	-8.0116	1.5439	-0.2273	-7.9779	1.5195	-0.2376
			**7F**			
	E(S0) =	-1649.7225	A.U.	E(S1) =	-1649.6506	A.U.
	x	y	z	x	y	z
H	0.1461	-2.7511	-3.0171	0.4270	-2.8983	-2.7942
H	-1.9650	-4.0821	-3.0784	-1.6475	-4.2724	-2.9513

H	-4.0954	-3.0122	-2.3943	-3.8417	-3.2369	-2.4480
C	-0.7811	-2.3190	-2.6477	-0.5302	-2.4715	-2.5049
C	-1.9723	-3.0640	-2.6969	-1.7024	-3.2426	-2.6074
H	4.4264	-2.8352	0.4792	4.3551	-2.8495	0.2000
H	6.8631	-2.8781	0.4238	6.7862	-2.8829	0.0766
H	8.1889	-0.8193	0.1192	8.1059	-0.7947	-0.0630
O	4.5254	2.8125	-0.2289	4.4823	2.8916	-0.0859
C	4.9534	-1.8999	0.3257	4.8899	-1.9058	0.1654
C	5.0756	0.4755	0.0084	4.9975	0.5320	0.0540
C	2.7994	1.0998	-0.0134	2.7789	1.1483	0.1042
C	2.8689	-0.2738	0.1492	2.8387	-0.2880	0.2377
C	1.5672	1.7814	-0.1741	1.5572	1.8102	-0.0729
C	1.6798	-1.0233	0.3562	1.6545	-1.0192	0.5263
C	0.3746	1.0173	-0.1293	0.3703	1.0133	-0.0843
C	0.4448	-0.3139	0.4319	0.4205	-0.3064	0.5595
C	1.5019	3.1926	-0.3594	1.4803	3.2128	-0.3264
C	0.2904	3.8012	-0.4747	0.2725	3.7793	-0.5906
C	-0.9036	3.0393	-0.6211	-0.8825	2.9717	-0.7872
C	-0.8403	1.6242	-0.6295	-0.7844	1.5457	-0.7163
C	4.1741	1.6511	-0.0996	4.1305	1.6990	0.0153
C	4.3004	-0.6863	0.1672	4.2173	-0.6673	0.1677
H	2.4288	3.7573	-0.3482	2.3998	3.7866	-0.2619
H	0.2155	4.8865	-0.5159	0.1682	4.8581	-0.6895
C	-2.1563	3.6960	-0.7495	-2.1495	3.5691	-0.9544
C	-1.9974	0.9021	-1.0975	-1.9180	0.7646	-1.1758
C	-3.2444	1.5656	-1.0776	-3.1840	1.3897	-1.2040
C	-3.3026	2.9708	-0.8738	-3.2775	2.7966	-1.0551
H	-2.1802	4.7830	-0.6987	-2.2186	4.6549	-0.9418
H	-4.2759	3.4580	-0.8890	-4.2618	3.2587	-1.1014
C	0.5010	-3.1078	0.7009	0.4891	-3.0412	1.1740
C	1.6661	-2.4422	0.4760	1.6509	-2.4032	0.8458
C	-0.6834	-2.3976	1.0348	-0.7067	-2.3128	1.3683
C	-0.6800	-0.9867	1.0337	-0.7028	-0.8991	1.1979
C	-1.8637	-3.1133	1.3904	-1.9136	-2.9852	1.7058
C	-2.9735	-2.4589	1.8201	-3.0659	-2.2934	1.9230
C	-2.9397	-1.0506	2.0401	-3.0532	-0.8680	1.9816
C	-1.7758	-0.3089	1.6965	-1.8522	-0.1620	1.7014
H	0.4748	-4.1957	0.7069	0.4768	-4.1166	1.3407
H	2.5802	-3.0008	0.3170	2.5761	-2.9659	0.8117
H	-1.8508	-4.1984	1.3054	-1.8973	-4.0723	1.7565
H	-3.8829	-3.0030	2.0697	-3.9974	-2.8142	2.1374
C	-4.0282	-0.4018	2.6636	-4.1998	-0.1581	2.3967
C	-1.7098	1.0359	2.1290	-1.8086	1.2089	2.0286
C	-2.7723	1.6413	2.7594	-2.9320	1.8797	2.4638
C	-3.9596	0.9284	3.0020	-4.1510	1.1998	2.6153
H	-4.8006	1.4171	3.4875	-5.0381	1.7356	2.9434

H	-2.6858	2.6769	3.0797	-2.8659	2.9387	2.7011
H	-0.7938	1.6009	1.9817	-0.8682	1.7487	1.9510
C	-4.4506	0.8311	-1.2878	-4.3607	0.6169	-1.4339
C	-1.9604	-0.4357	-1.6529	-1.8166	-0.5795	-1.6764
C	-4.4217	-0.4960	-1.5734	-4.2792	-0.7118	-1.7056
C	-3.1780	-1.1499	-1.8214	-3.0091	-1.3330	-1.8835
H	-5.3960	1.3628	-1.1945	-5.3242	1.1206	-1.3828
H	-5.3425	-1.0635	-1.6972	-5.1763	-1.3094	-1.8555
C	-3.1517	-2.4758	-2.3063	-2.9201	-2.6715	-2.3205
C	-0.7793	-1.0393	-2.1422	-0.5876	-1.1734	-2.0556
C	6.3549	-1.9247	0.3021	6.2684	-1.9270	0.0828
C	7.1037	-0.7675	0.1327	7.0220	-0.7355	-0.0022
C	6.4536	0.4615	-0.0123	6.3883	0.4932	-0.0226
H	7.0046	1.3913	-0.1359	6.9448	1.4246	-0.1029
H	0.1485	-0.4747	-2.1390	0.3225	-0.5808	-2.0249
H	-4.9164	-0.9884	2.8930	-5.1182	-0.7158	2.5729

8F

	E(S0) =	-1803.2506	A.U.	E(S1) =	-1803.1809	A.U.
	x	y	z	x	y	z
H	4.0432	-3.7964	1.8370	4.1465	-3.8240	1.5993
H	-0.2416	-4.0515	1.9849	-0.1285	-4.1140	1.9101
C	0.6648	-3.4607	1.8752	0.7683	-3.5121	1.7843
H	1.9849	-5.1778	1.9255	2.1004	-5.2189	1.7278
H	-0.4064	-1.6246	1.8142	-0.3144	-1.6803	1.8396
H	-0.5760	3.8858	-2.1340	-0.8856	3.8586	-1.9544
H	1.4521	5.3186	-1.8748	1.0736	5.3825	-1.7180
H	3.6661	4.2438	-1.5669	3.3519	4.4260	-1.5298
C	0.3905	3.4305	-1.9303	0.1088	3.4457	-1.8037
C	1.5334	4.2371	-1.7984	1.2152	4.3052	-1.6837
H	-4.7678	2.4310	1.4152	-4.7224	2.5673	1.0783
H	-7.2018	2.3072	1.3894	-7.1502	2.4282	1.0153
H	-8.3924	0.4118	0.3504	-8.3132	0.3970	0.2129
O	-4.5029	-2.5458	-1.3355	-4.4266	-2.7503	-1.0789
C	-5.2336	1.5801	0.9304	-5.1826	1.6463	0.7356
C	-5.2026	-0.5128	-0.2423	-5.1095	-0.6158	-0.1707
C	-2.8906	-0.9129	-0.5022	-2.8479	-1.0278	-0.3606
C	-3.0487	0.2941	0.1581	-3.0090	0.2807	0.2252
C	-1.6203	-1.3973	-0.9062	-1.5900	-1.5040	-0.7551
C	-1.9063	1.0040	0.6185	-1.8738	0.9893	0.6973
C	-0.4816	-0.6163	-0.5908	-0.4627	-0.6590	-0.5167
C	-0.6260	0.4145	0.4125	-0.5924	0.3995	0.4960
C	-1.4660	-2.6442	-1.5771	-1.4224	-2.7423	-1.4449
C	-0.2171	-3.0975	-1.8722	-0.1841	-3.1088	-1.8723
C	0.9236	-2.2606	-1.7175	0.9108	-2.2039	-1.7909
C	0.7664	-0.9346	-1.2464	0.7141	-0.8843	-1.2726
C	-4.2267	-1.4935	-0.7829	-4.1586	-1.6304	-0.6024

C	-4.5039	0.5627	0.3330	-4.4184	0.5420	0.3123
H	-2.3537	-3.2351	-1.7792	-2.2987	-3.3711	-1.5689
H	-0.0708	-4.0941	-2.2855	-0.0088	-4.0896	-2.3105
C	2.2187	-2.7543	-2.0291	2.2135	-2.6215	-2.1311
C	1.8622	-0.0170	-1.4462	1.7753	0.0863	-1.4722
C	3.1512	-0.5623	-1.6395	3.0827	-0.3989	-1.7005
C	3.3102	-1.9523	-1.8914	3.2813	-1.7709	-1.9902
H	2.3180	-3.7948	-2.3330	2.3619	-3.6502	-2.4533
H	4.3154	-2.3402	-2.0470	4.2950	-2.1209	-2.1769
C	-0.8530	2.9195	1.6540	-0.8189	2.8368	1.8546
C	-1.9832	2.2774	1.2538	-1.9501	2.2214	1.4070
C	0.4026	2.2525	1.6479	0.4385	2.1875	1.7856
C	0.4833	0.9147	1.1922	0.5133	0.8624	1.2610
C	1.5743	2.9267	2.0835	1.6256	2.8580	2.1627
C	2.7753	2.2853	2.0753	2.8395	2.2392	2.0468
C	2.8434	0.8867	1.8299	2.9167	0.8523	1.7537
C	1.6720	0.1632	1.5148	1.7387	0.1191	1.4888
H	-0.8979	3.9364	2.0391	-0.8671	3.8276	2.3027
H	-2.9415	2.7738	1.3438	-2.9115	2.6974	1.5578
H	1.4942	3.9719	2.3756	1.5539	3.8924	2.4935
H	3.6937	2.8125	2.3279	3.7620	2.7772	2.2580
C	4.0962	0.2121	1.9382	4.1795	0.1887	1.7796
C	1.7238	-1.2811	1.6291	1.8051	-1.3190	1.5706
C	2.9900	-1.9223	1.7203	3.0812	-1.9535	1.5846
C	4.1772	-1.1375	1.8169	4.2642	-1.1596	1.6370
H	4.9880	0.8138	2.1061	5.0742	0.7952	1.9102
H	5.1369	-1.6491	1.8680	5.2294	-1.6632	1.6297
C	3.0599	-3.3301	1.7954	3.1606	-3.3617	1.6143
C	0.5733	-2.0913	1.7664	0.6630	-2.1411	1.7258
C	4.2991	0.2856	-1.6222	4.1908	0.5004	-1.6954
C	1.7193	1.4220	-1.5594	1.5568	1.5053	-1.5594
C	4.1749	1.6320	-1.5047	4.0013	1.8380	-1.5566
C	2.8839	2.2384	-1.5332	2.6826	2.3802	-1.5404
H	5.2801	-0.1813	-1.6958	5.1908	0.0828	-1.7985
H	5.0522	2.2755	-1.4647	4.8462	2.5236	-1.5243
C	2.7596	3.6420	-1.6166	2.4799	3.7752	-1.5706
C	0.4844	2.0619	-1.8131	0.2766	2.0816	-1.7462
C	-6.6337	1.5064	0.9225	-6.5626	1.5727	0.6915
C	-7.3063	0.4404	0.3402	-7.2263	0.4167	0.2307
C	-6.5780	-0.5959	-0.2517	-6.5005	-0.6775	-0.2049
H	-7.0673	-1.4531	-0.7093	-6.9859	-1.5771	-0.5771
H	-0.4105	1.4610	-1.9462	-0.5845	1.4340	-1.8855
C	1.9188	-4.0948	1.8560	2.0255	-4.1349	1.6951

Computional Investigation of intramolecular Electrophilic Aromatic Cyclization

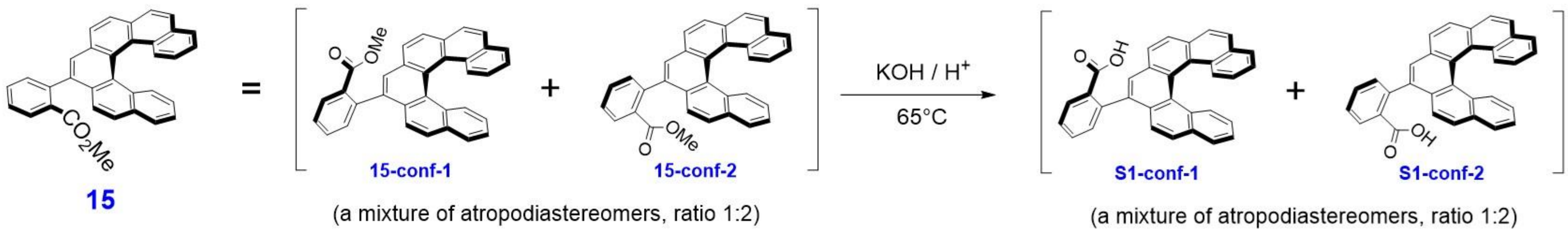

(a mixture of atropodiastereomers, ratio 1:2)

(a mixture of atropodiastereomers, ratio 1:2)

For clarity, only atropodiastereomers with *M*-helixity are shown.

Mechanism of Intramolecular Electrophilic Aromatic Cyclization:

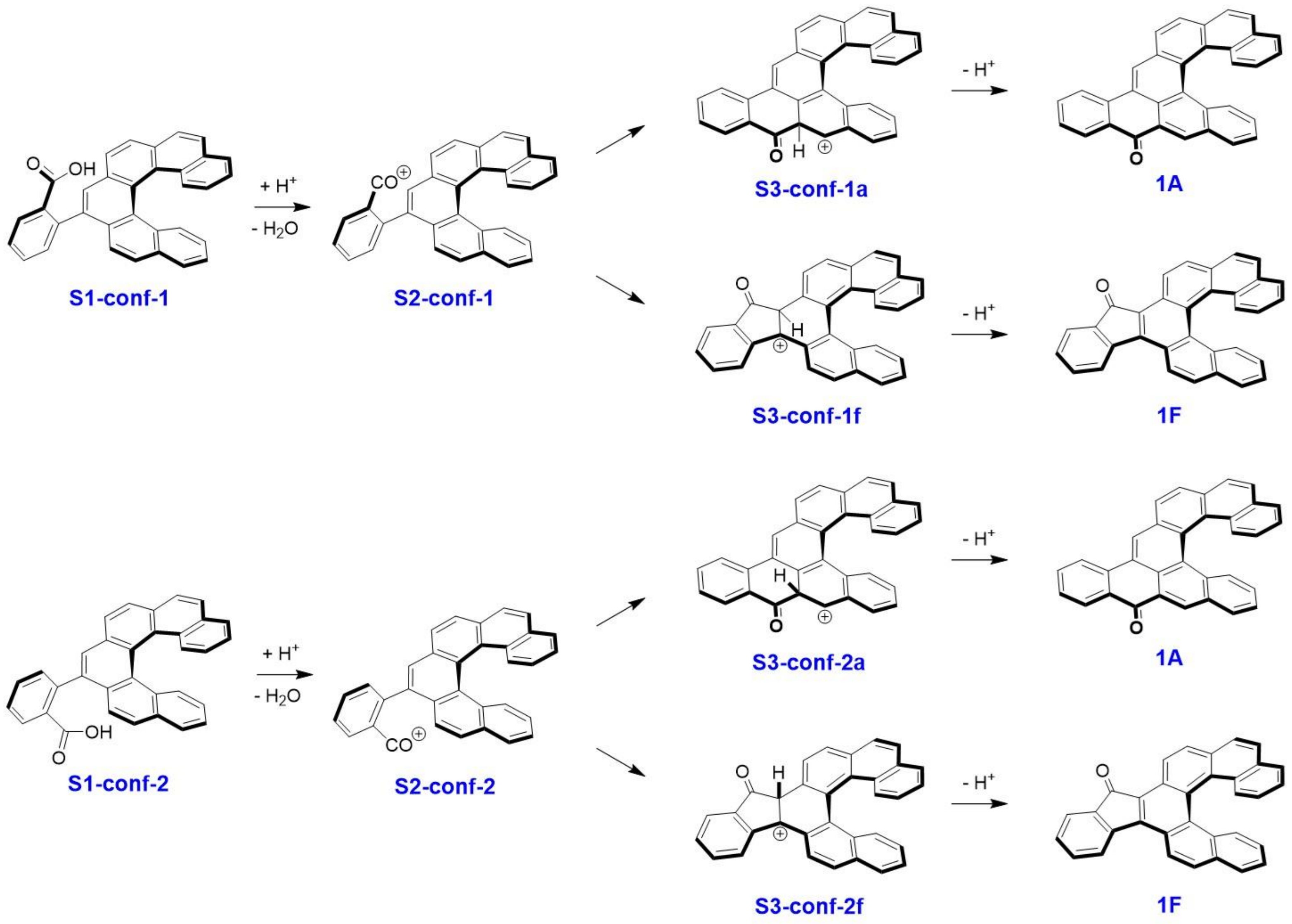

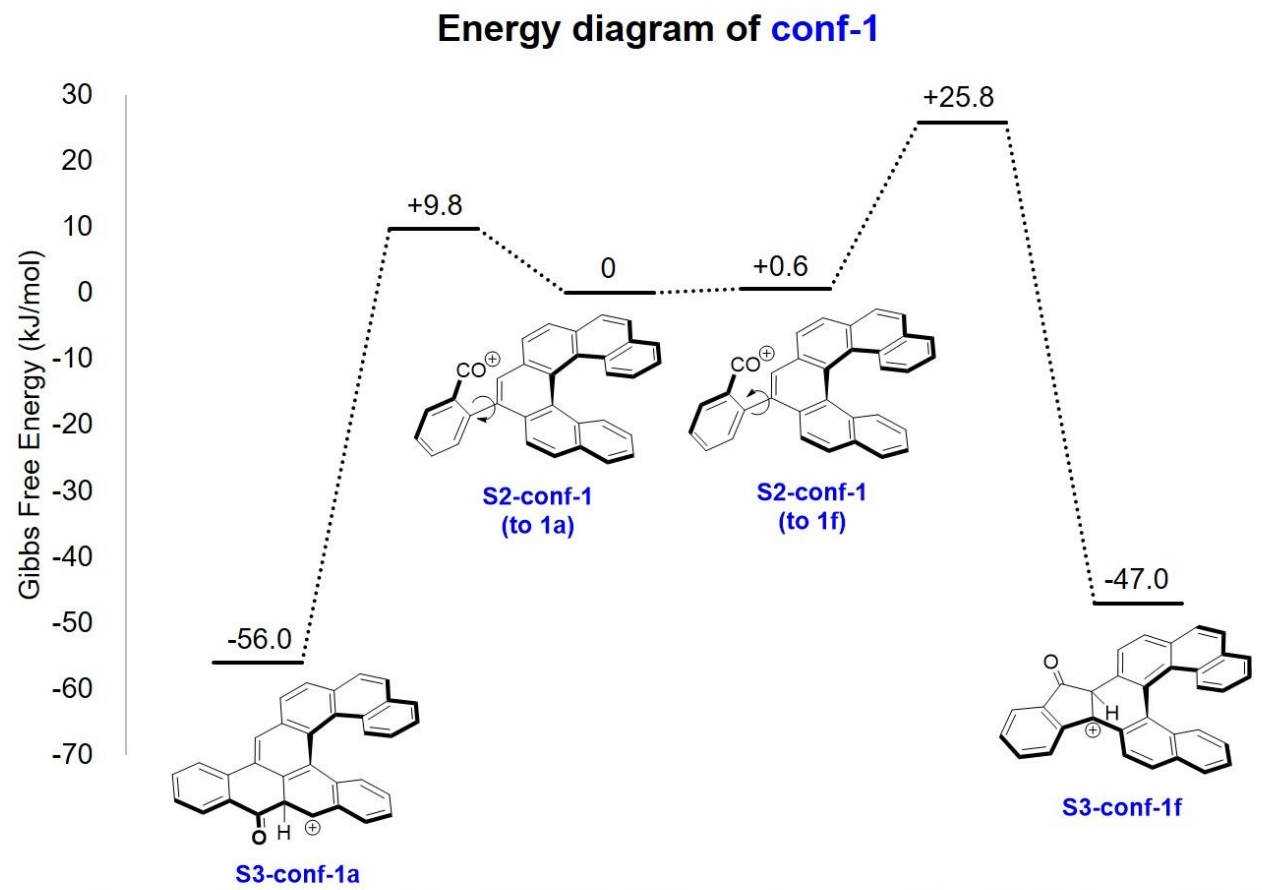

All calculations were performed using GRRM 23 program with Gaussian 16 Rev C.02. The equilibrium (EQ) and transition structures (TS) were optimized at the level of ωB97X-D/Def2SVP. The values of the Gibbs free energy of EQs and TSs were calculated at 353.15 K.

S2-conf-1 has two conformers that are close in energy, and these structures are in equilibrium. One conformer has a reaction pathway leading to the transition state that gives **1a**, and the other has a reaction pathway leading to the transition state that gives **1f**.

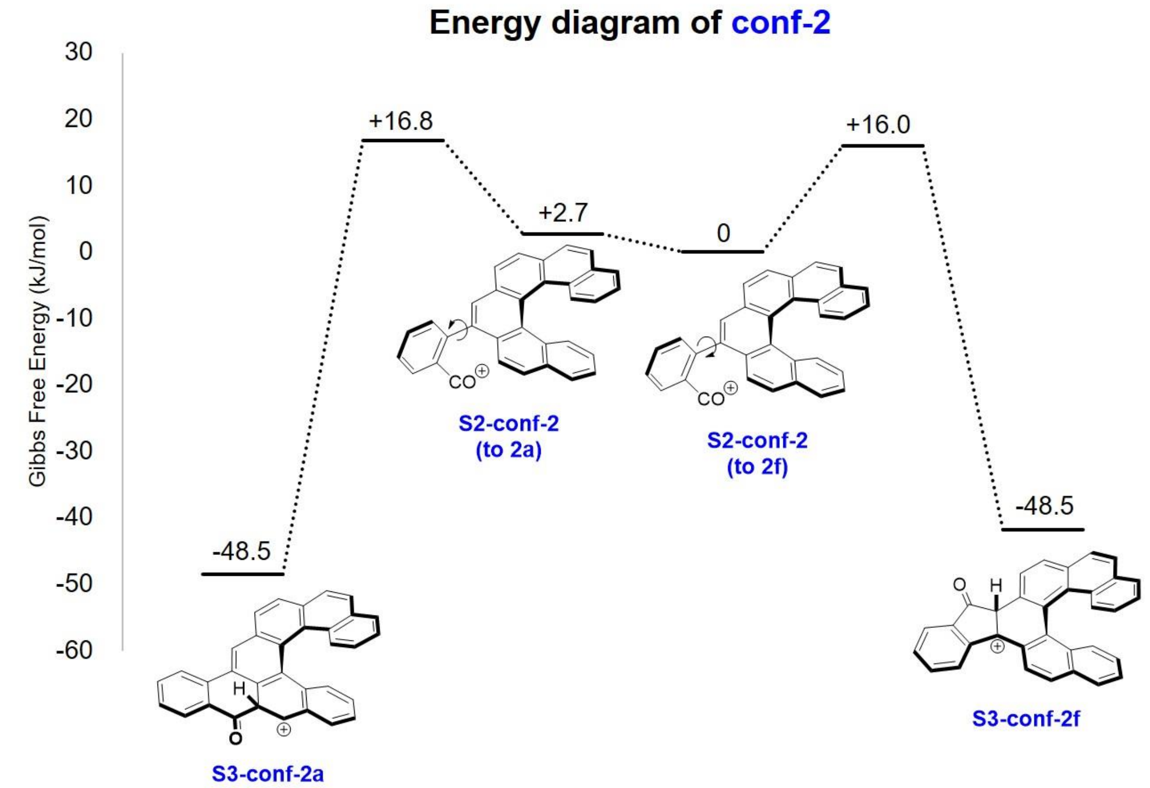

All calculations were performed using GRRM 23 program with Gaussian 16 Rev C.02. The equilibrium (EQ) and transition structures (TS) were optimized at the level of ωB97X-D/Def2SVP. The values of the Gibbs free energy of EQs and TSs were calculated at 353.15 K.

S2-conf-2 also has two conformers that are close in energy, and these structures are in equilibrium. One conformer has a reaction pathway leading to the transition state that gives **2a**, and the other has a reaction pathway leading to the transition state that gives **2f**.

Table S6. Optimized geometry (Cartesian coordinates, Å) of S2-conf-1 (to_1a)

C	-0.42401600	-0.79563600	-0.77707100
C	-0.04963500	0.59472400	-0.57172100
C	0.55864600	-1.64467300	-1.34956000
C	-1.71568300	-1.37278600	-0.48060900
C	-2.08981600	-2.54153700	-1.18265000
C	0.18329900	-2.88703100	-1.93997400
C	-1.12331800	-3.26898100	-1.94013100
C	-2.62483900	-0.86876800	0.54025600
C	-3.43619800	-3.02586700	-1.10520100
C	1.92650700	-1.27876000	-1.26289100
H	0.94986300	-3.50332800	-2.41438200
H	-1.43714300	-4.18051300	-2.45282600
C	1.31530800	0.90989700	-0.38860200
C	-1.00259400	1.69305500	-0.64847000
C	-0.60355900	2.99088700	-0.23036100
C	1.69763900	2.25306200	-0.04990900
C	0.76672200	3.23344500	0.09301500
C	-2.30500300	1.55162700	-1.18926800
C	-1.53808300	4.05500100	-0.23034400
C	2.30869200	-0.10335500	-0.66138300
H	1.07078200	4.24586400	0.36822800
C	-2.23219100	0.04126400	1.55187700
C	-3.95734300	-1.35950900	0.57760000
C	-4.87179800	-0.84294000	1.52674600
C	-3.13087100	0.51123000	2.48437000
C	-4.47409900	0.09042800	2.45677000
C	-4.34907900	-2.42021700	-0.30301300
H	-5.89800800	-1.21744600	1.52366900
H	-1.19536000	0.36836800	1.61197500
H	-2.79575200	1.20912500	3.25412600
H	-5.18541300	0.47809100	3.18831000
C	-3.18913200	2.60665100	-1.20976800
H	-4.18349000	2.46613600	-1.63731000
C	-2.81866000	3.86436800	-0.69419500
H	-5.38144100	-2.77543200	-0.27038400
H	-3.71395500	-3.89012100	-1.71204000
H	2.67685000	-2.01026700	-1.57397500
C	3.72085500	0.07188400	-0.29662000
H	-1.21513200	5.03891700	0.11744400
H	-2.61433100	0.59917600	-1.61491100
C	4.09034200	0.42003900	1.03922600
C	4.77834100	-0.13923100	-1.18675400
C	3.09467900	0.51964100	1.99897500
C	5.43981900	0.59079000	1.45769200
C	6.09985500	0.01776400	-0.78251600
H	4.54830500	-0.39728100	-2.22169300
H	6.89831400	-0.13830000	-1.51132600
C	6.43765700	0.39007500	0.53112200
H	5.67095300	0.86563400	2.48827100
H	7.48223400	0.51713000	0.81705900
H	2.75362700	2.51110200	0.05382600
O	2.34031900	0.57538800	2.83537600
H	-3.53265600	4.68989600	-0.69845500

Table S7. Optimized geometry (Cartesian coordinates, Å) of S3-conf-1a

C	-0.46766100	-0.95136400	-0.76060900
C	-0.03122200	0.38328600	-0.41838200
C	0.51377200	-1.79920000	-1.33565100
C	-1.80545300	-1.47136600	-0.59387700
C	-2.19097100	-2.55646400	-1.41122200
C	0.11437800	-2.98412500	-2.02598700
C	-1.20662400	-3.29032100	-2.14172800
C	-2.75689300	-0.98979600	0.39635200
C	-3.56858700	-2.94540100	-1.47889300
C	1.88582500	-1.48182900	-1.19814400
H	0.87893800	-3.61017000	-2.49011800
H	-1.52788400	-4.14797300	-2.73622800
C	1.33821700	0.63798100	-0.25250300
C	-0.93295100	1.52571700	-0.36440700
C	-0.54183300	2.72074800	0.35286900
C	1.80179100	1.97902100	0.20410100
C	0.77713400	2.89656100	0.68811900
C	-2.18784300	1.55160200	-0.99886800
C	-1.49399900	3.77143600	0.59289200
C	2.32434500	-0.32844100	-0.57819900
H	1.11647200	3.77859800	1.24067200
C	-2.38161500	-0.22216200	1.52743200
C	-4.12117200	-1.36483500	0.27618800
C	-5.07368600	-0.84611500	1.18731400
C	-3.31963300	0.24543600	2.42158400
C	-4.68693400	-0.03716000	2.23189700
C	-4.50660800	-2.32452400	-0.71696400
H	-6.12254300	-1.12624900	1.06564600
H	-1.32671500	-0.02642700	1.71981600
H	-2.99896100	0.81890400	3.29363900
H	-5.42813400	0.34567100	2.93579900
C	-3.05603900	2.60863400	-0.80439900
H	-4.02590600	2.58994700	-1.30664300
C	-2.73739900	3.70761500	0.03355400
H	-5.56001500	-2.60076600	-0.79983300
H	-3.85064400	-3.74506400	-2.16678800
H	2.60224900	-2.22251800	-1.55595400
C	3.74895100	-0.08149600	-0.24308000
H	-1.17991500	4.63592500	1.18160400
H	-2.49034900	0.72197300	-1.63526100
C	4.10489800	0.98724600	0.60946200
C	4.77300900	-0.93276700	-0.67809000
C	3.10744400	1.98805400	1.03283500
C	5.42579700	1.15813400	1.04331900
C	6.08760700	-0.74358800	-0.26451100
H	4.55528300	-1.76451700	-1.34817300
H	6.86364100	-1.42212000	-0.62398500
C	6.42029900	0.29553400	0.60702100
H	5.64906900	1.99136600	1.71205100
H	7.45221000	0.43331700	0.93340300
H	2.15702000	2.51343500	-0.71542500
O	3.28585200	2.84007300	1.86385900
H	-3.46415100	4.50596200	0.18748100

Table S8. Optimized geometry (Cartesian coordinates, Å) of the transition state between S2-conf-1 (to_1a) and S3-conf-1a

C	-0.42846	-0.83721	-0.88151
C	-0.02698	0.53947	-0.63316
C	0.52755	-1.67296	-1.51265
C	-1.71619	-1.41157	-0.56752
C	-2.13097	-2.53950	-1.31175
C	0.11793	-2.88459	-2.13858
C	-1.19634	-3.24609	-2.12385
C	-2.58168	-0.94916	0.50852
C	-3.48551	-3.00114	-1.21847
C	1.91159	-1.34567	-1.42076
H	0.86259	-3.49450	-2.65413
H	-1.53620	-4.12983	-2.66773
C	1.34842	0.83405	-0.55781
C	-0.95887	1.65457	-0.57464
C	-0.51205	2.92239	-0.08764
C	1.79166	2.16818	-0.22269
C	0.87186	3.15570	0.06922
C	-2.29640	1.57274	-1.03048
C	-1.43712	3.99004	0.08330
C	2.32633	-0.18034	-0.83506
H	1.21360	4.16266	0.32112
C	-2.13323	-0.11417	1.56097
C	-3.92401	-1.41020	0.55816
C	-4.79643	-0.92448	1.56170
C	-2.99107	0.32385	2.54623
C	-4.34655	-0.05704	2.53156
C	-4.36386	-2.41624	-0.36434
H	-5.83181	-1.27286	1.57000
H	-1.08317	0.17098	1.61391
H	-2.61368	0.95936	3.34976
H	-5.02577	0.30394	3.30608
C	-3.16375	2.63267	-0.89050
H	-4.18827	2.53564	-1.25456
C	-2.74821	3.84200	-0.29060
H	-5.40229	-2.75182	-0.31981
H	-3.79710	-3.83056	-1.85669
H	2.63358	-2.11163	-1.71335
C	3.70283	-0.00226	-0.34068
H	-1.07499	4.93977	0.48306
H	-2.64895	0.66230	-1.51000
C	3.90577	0.67829	0.88696
C	4.82857	-0.57416	-0.93491
C	2.83152	1.33718	1.56693
C	5.16580	0.75432	1.51519
C	6.08240	-0.48148	-0.33247
H	4.72451	-1.09414	-1.88833
H	6.94522	-0.93042	-0.82879
C	6.25708	0.17126	0.89187
H	5.27668	1.27665	2.46724
H	7.24359	0.23009	1.35325
H	2.80552	2.48855	-0.47466
O	2.36902	1.70312	2.54582
H	-3.45597	4.66332	-0.16783

Table S9. Optimized geometry (Cartesian coordinates, Å) of S2-conf-1 (to 1f)

C	-0.45026300	-0.82993400	-0.55866200
C	-0.06309400	0.56567000	-0.44145500
C	0.55591000	-1.75065200	-0.95150900
C	-1.78109100	-1.35291900	-0.33432200
C	-2.12256100	-2.57173800	-0.96359400
C	0.20074000	-3.03439800	-1.46219600
C	-1.11041300	-3.38812700	-1.55243400
C	-2.76508800	-0.74333000	0.54960400
C	-3.48558600	-3.01337200	-0.98064300
C	1.91817000	-1.38218900	-0.83350100
H	0.98897500	-3.70514900	-1.81098700
H	-1.40148500	-4.33594500	-2.00980500
C	1.28884400	0.87883200	-0.17486800
C	-0.98595800	1.67360200	-0.63827700
C	-0.61270600	2.97281900	-0.20293400
C	1.63076100	2.19607600	0.28240900
C	0.70105700	3.18619200	0.32153600
C	-2.22521600	1.53222800	-1.30967000
C	-1.52246600	4.04904900	-0.33325800
C	2.28895100	-0.13763400	-0.36167800
H	0.96365400	4.17521100	0.70329900
C	-2.43724400	0.22839100	1.52643400
C	-4.11180600	-1.19128900	0.49157100
C	-5.09503800	-0.57151800	1.29984000
C	-3.40454400	0.79858400	2.32438700
C	-4.75443200	0.42087800	2.19053900
C	-4.45201200	-2.31340100	-0.33235900
H	-6.12950000	-0.91400900	1.22267700
H	-1.39872400	0.52296400	1.67041700
H	-3.11900200	1.54175000	3.07130400
H	-5.51869000	0.88800400	2.81436100
C	-3.08200300	2.60013900	-1.45656500
H	-4.02664700	2.46356900	-1.98594500
C	-2.74558900	3.86522200	-0.93655100
H	-5.49447600	-2.63663800	-0.37512700
H	-3.73149500	-3.92187700	-1.53426900
H	2.67755500	-2.10010800	-1.15920200
C	3.72763400	0.11754000	-0.18445400
H	-1.22714800	5.03548300	0.03143300
H	-2.50258400	0.57009100	-1.73721200
C	4.48250300	-0.79207200	0.60791300
C	4.44349900	1.15391200	-0.79289500
C	3.76964400	-1.74143400	1.33240900
C	5.89541200	-0.72085600	0.75430200
C	5.82404200	1.23620100	-0.64977700
H	3.91027500	1.87139700	-1.41822500
H	6.36195800	2.03831700	-1.15989800
C	6.55329800	0.30439200	0.11331400
H	6.43243500	-1.44911300	1.36411400
H	7.63666900	0.39493900	0.20024500
H	2.64382900	2.39825200	0.63300600
O	3.28527100	-2.50136900	2.01300100
H	-3.43956500	4.70126700	-1.04062700

Table S10. Optimized geometry (Cartesian coordinates, Å) of S3-conf-1f

C	-0.35692800	-0.75964000	-0.36456900
C	-0.05663900	0.65939400	-0.43784700
C	0.68026200	-1.63931800	-0.73264200
C	-1.65438100	-1.30245400	-0.04361800
C	-1.94567500	-2.60914600	-0.50017800
C	0.39532900	-2.97150300	-1.07259000
C	-0.90655400	-3.41636400	-1.02210200
C	-2.64708900	-0.62103200	0.77023500
C	-3.28630100	-3.11811600	-0.41378600
C	2.06575400	-1.10672200	-0.85378200
H	1.20204400	-3.63295400	-1.38711600
H	-1.14398300	-4.43405400	-1.34030000
C	1.27134500	1.12735200	-0.10931100
C	-1.02907900	1.64214900	-0.82845900
C	-0.80142100	3.00932000	-0.49522600
C	1.43436300	2.50507200	0.30424300
C	0.42728100	3.39299300	0.15386500
C	-2.20212700	1.30024000	-1.54909200
C	-1.78528200	3.96515900	-0.80411600
C	2.31931100	0.23739200	-0.26354500
H	0.55043300	4.42988400	0.47190500
C	-2.34559000	0.48777400	1.59803600
C	-3.97281800	-1.13007200	0.79658100
C	-4.97163300	-0.43833800	1.52183300
C	-3.32810100	1.13261900	2.31630600
C	-4.66281100	0.68696000	2.25304500
C	-4.27207000	-2.37715000	0.15066200
H	-5.99167900	-0.82879400	1.51454100
H	-1.31380000	0.82331100	1.70581400
H	-3.06644500	1.97819200	2.95511000
H	-5.43961700	1.20666300	2.81681700
C	-3.13494800	2.26032800	-1.87019100
H	-4.02555200	1.98582100	-2.43719700
C	-2.93806300	3.59551600	-1.47241700
H	-5.29777400	-2.75055000	0.18299400
H	-3.49508700	-4.10817300	-0.82392400
H	2.29914400	-0.99691400	-1.93552000
C	3.73850300	0.32675800	0.07792600
H	-1.62104200	5.00758400	-0.52426100
H	-2.35449100	0.27105800	-1.87257900
C	4.27335600	-0.97826600	0.10544900
C	4.57682600	1.41107400	0.37114500
C	3.25529200	-1.96243100	-0.33582800
C	5.59208900	-1.23627700	0.45370000
C	5.90215000	1.15611300	0.71581600
H	4.23640300	2.44265800	0.30164200
H	6.56413200	1.99433800	0.94074700
C	6.40568100	-0.14987500	0.77192000
H	5.96509500	-2.26190500	0.47171500
H	7.44685900	-0.31316800	1.05623800
H	2.38044600	2.82238600	0.73521300
O	3.33666800	-3.15565500	-0.35297800
H	-3.69057700	4.34892300	-1.71351700

Table S11. Optimized geometry (Cartesian coordinates, Å) of the transition state between S2-conf-1 to S3-conf-1f

C	-0.38074	-0.77421	-0.57688
C	-0.04727	0.64100	-0.53374
C	0.63980	-1.65915	-1.00787
C	-1.67497	-1.33939	-0.26924
C	-1.99534	-2.59370	-0.83888
C	0.31677	-2.96601	-1.46315
C	-0.98265	-3.38157	-1.45809
C	-2.63902	-0.73511	0.63924
C	-3.33784	-3.09218	-0.76816
C	1.99516	-1.22313	-0.97698
H	1.11098	-3.61229	-1.84289
H	-1.25452	-4.35613	-1.86881
C	1.30221	1.02329	-0.31653
C	-1.02049	1.69859	-0.73164
C	-0.68763	3.02625	-0.34803
C	1.60111	2.36559	0.10039
C	0.63058	3.31371	0.13345
C	-2.27663	1.47853	-1.34940
C	-1.64807	4.05556	-0.47942
C	2.32761	0.05819	-0.52711
H	0.85826	4.32544	0.47538
C	-2.30452	0.28722	1.56029
C	-3.96671	-1.23882	0.66458
C	-4.93474	-0.62618	1.49588
C	-3.25546	0.85152	2.38174
C	-4.59320	0.41502	2.32897
C	-4.29892	-2.40697	-0.09762
H	-5.95646	-1.01244	1.48436
H	-1.27269	0.62588	1.64428
H	-2.96576	1.63403	3.08557
H	-5.34539	0.87593	2.97176
C	-3.18377	2.50299	-1.49894
H	-4.14022	2.30984	-1.98766
C	-2.88241	3.79754	-1.03253
H	-5.32738	-2.77388	-0.07403
H	-3.57195	-4.03102	-1.27411
H	2.74954	-1.83666	-1.48066
C	3.75299	0.23659	-0.20266
H	-1.38634	5.06559	-0.15665
H	-2.52522	0.49066	-1.73392
C	4.26049	-0.85928	0.52460
C	4.63116	1.26956	-0.52944
C	3.26098	-1.82872	0.83424
C	5.60839	-0.98310	0.90698
C	5.96904	1.16285	-0.15182
H	4.28762	2.12685	-1.10926
H	6.66208	1.96059	-0.42726
C	6.45751	0.05432	0.55872
H	5.96295	-1.85098	1.46490
H	7.50994	0.01212	0.84284
H	2.61510	2.61792	0.41005
O	2.80913	-2.69272	1.42781
H	-3.61534	4.59954	-1.13778

Table S12. Optimized geometry (Cartesian coordinates, Å) of S2-conf-2 (to 2a)

C	-0.56144800	-1.09306800	-0.16038000
C	0.01807800	0.23187100	-0.04812700
C	0.33049100	-2.18075800	-0.35505100
C	-1.97852500	-1.39129400	-0.10632100
C	-2.42231900	-2.58647200	-0.71385800
C	-0.15711900	-3.43447000	-0.83138600
C	-1.48268400	-3.59153800	-1.09581100
C	-2.96248700	-0.57973100	0.59565600
C	-3.82330300	-2.81520200	-0.90955900
C	1.71433800	-2.01097100	-0.09890100
H	0.55235700	-4.24362500	-1.01669300
H	-1.85692000	-4.51937200	-1.53343900
C	1.35963500	0.35188700	0.37466500
C	-0.68160200	1.45927200	-0.40708600
C	-0.17248100	2.70786600	0.03892800
C	1.82888800	1.62321200	0.86669200
C	1.06699000	2.74304600	0.75389600
C	-1.82070100	1.47300300	-1.24738400
C	-0.87236900	3.90178100	-0.25557800
C	2.21751000	-0.80367800	0.32033100
H	1.41111900	3.69124800	1.17303100
C	-2.61896400	0.39032800	1.56915700
C	-4.34315600	-0.81679000	0.35995000
C	-5.31403300	0.00260200	0.98510200
C	-3.58091100	1.15611900	2.18982400
C	-4.94251800	0.98707700	1.87210400
C	-4.74406200	-1.92913600	-0.44948600
H	-6.36982700	-0.17942400	0.77156900
H	-1.57678700	0.52296300	1.85628100
H	-3.28457200	1.89039700	2.94132800
H	-5.69918100	1.60790400	2.35536000
C	-2.46798000	2.65052400	-1.55158400
H	-3.34036400	2.63094500	-2.20721000
C	-2.01200800	3.87524900	-1.02727200
H	-5.80909100	-2.09108000	-0.62980500
H	-4.13398200	-3.71496900	-1.44466400
H	2.38176300	-2.86325200	-0.25071700
C	3.67268500	-0.68049800	0.52587200
H	-0.48096100	4.84926000	0.12192500
H	-2.18654100	0.54175000	-1.67689200
C	4.43898400	0.26303400	-0.22202700
C	4.39132700	-1.49900000	1.39817500
C	3.80134400	1.02111900	-1.19851600
C	5.84394800	0.41453900	-0.08024700
C	5.77104200	-1.36259900	1.53871200
H	3.84721000	-2.23478000	1.99261300
H	6.29879700	-2.01098600	2.24165900
C	6.49918200	-0.40603700	0.81356200
H	6.38668900	1.15482600	-0.67066400
H	7.57715500	-0.31315400	0.94972700
H	2.78535900	1.67405100	1.39216300
O	3.37602800	1.63060900	-2.04535500
H	-2.54420300	4.80033000	-1.25598400

Table S13. Optimized geometry (Cartesian coordinates, Å) of S3-conf-2a

C	-0.62182200	-1.19859700	-0.15537100
C	-0.00349100	0.10907700	-0.15955800
C	0.25149400	-2.30101800	-0.34752200
C	-2.03463900	-1.46036700	-0.00498800
C	-2.52725100	-2.69224300	-0.49096100
C	-0.28104400	-3.57770500	-0.70345800
C	-1.62337700	-3.73575900	-0.85749700
C	-2.96946100	-0.56520100	0.66351600
C	-3.93970300	-2.91049400	-0.58961200
C	1.64747500	-2.13124900	-0.20382900
H	0.40638600	-4.40631200	-0.88410900
H	-2.03462100	-4.68734900	-1.20022600
C	1.37006200	0.21590200	0.12751400
C	-0.67532600	1.30367800	-0.64703500
C	-0.09913800	2.61522500	-0.42734700
C	1.96156000	1.54811100	0.43385900
C	1.16540500	2.71894900	0.09004600
C	-1.84898100	1.25843600	-1.42658600
C	-0.79452400	3.80400100	-0.84676000
C	2.21694300	-0.91961100	0.13896500
H	1.63700800	3.69566900	0.23514200
C	-2.56558500	0.48815900	1.52178100
C	-4.36379200	-0.79304700	0.51455500
C	-5.28984000	0.10394800	1.10091900
C	-3.48377100	1.33119500	2.10885200
C	-4.86155200	1.16172900	1.87029800
C	-4.82190700	-1.96885000	-0.16535700
H	-6.35746100	-0.07621300	0.95597100
H	-1.50960600	0.61632800	1.75853300
H	-3.14213600	2.12193300	2.77980200
H	-5.58453900	1.83967400	2.32742400
C	-2.46776700	2.41693500	-1.84987300
H	-3.37400400	2.33464600	-2.45426500
C	-1.96869000	3.70689600	-1.53215400
H	-5.89701200	-2.12649300	-0.27550400
H	-4.29242900	-3.84730300	-1.02560200
H	2.27924900	-3.00635200	-0.36496200
C	3.67570200	-0.76418600	0.35357900
H	-0.34132000	4.77393900	-0.63188000
H	-2.27649000	0.29886300	-1.70820900
C	4.28231000	0.50753400	0.26823200
C	4.50510700	-1.86877700	0.58539400
C	3.47291800	1.73254100	0.13075200
C	5.67314300	0.65045900	0.35512700
C	5.88417400	-1.71854900	0.69245700
H	4.07557400	-2.86523800	0.69553200
H	6.50538400	-2.59680800	0.87859900
C	6.47678300	-0.46051300	0.56570800
H	6.09894700	1.65186100	0.27043900
H	7.55928500	-0.35002800	0.64560800
H	1.99300800	1.59402800	1.55381800
O	3.91189600	2.83227200	-0.08205800
H	-2.50117300	4.59775100	-1.86677300

Table S14. Optimized geometry (Cartesian coordinates, Å) of the transition state between S2-conf-2 to S3-conf-2a

C	-0.57839	-1.18340	-0.04984
C	0.03383	0.12939	0.04685
C	0.29731	-2.29282	-0.18310
C	-2.00239	-1.43792	-0.04752
C	-2.45559	-2.64886	-0.61723
C	-0.20773	-3.55236	-0.61734
C	-1.53013	-3.69184	-0.91424
C	-2.99268	-0.56286	0.56496
C	-3.85369	-2.84648	-0.86641
C	1.69210	-2.13688	0.05457
H	0.48637	-4.38467	-0.75005
H	-1.91122	-4.63009	-1.32276
C	1.36124	0.21588	0.49780
C	-0.58519	1.37001	-0.40081
C	-0.03900	2.62476	0.01052
C	1.88081	1.49335	0.95514
C	1.14142	2.64356	0.79186
C	-1.67992	1.39525	-1.29501
C	-0.67319	3.83687	-0.37227
C	2.21512	-0.93611	0.45217
H	1.48656	3.58557	1.22436
C	-2.66645	0.44116	1.50953
C	-4.36709	-0.76789	0.27025
C	-5.33902	0.11309	0.80299
C	-3.63127	1.26798	2.04206
C	-4.97946	1.12856	1.65997
C	-4.76551	-1.90985	-0.49962
H	-6.38852	-0.04596	0.54467
H	-1.63770	0.54765	1.85154
H	-3.34949	2.02482	2.77668
H	-5.73817	1.79571	2.07325
C	-2.25172	2.58661	-1.68579
H	-3.09174	2.57281	-2.38276
C	-1.76947	3.81996	-1.19903
H	-5.82553	-2.04893	-0.72322
H	-4.16772	-3.76365	-1.36893
H	2.34731	-2.99421	-0.11697
C	3.67555	-0.73073	0.52845
H	-0.25773	4.78269	-0.01768
H	-2.07193	0.46144	-1.69416
C	4.23813	0.41037	-0.09685
C	4.56462	-1.65303	1.07658
C	3.39619	1.40228	-0.69531
C	5.62695	0.62158	-0.18738
C	5.94304	-1.44359	1.00799
H	4.17070	-2.54238	1.57142
H	6.61611	-2.17723	1.45638
C	6.47790	-0.31636	0.37849
H	6.02164	1.50818	-0.68702
H	7.55760	-0.16942	0.33015
H	2.71411	1.49930	1.66131
O	3.19310	2.16839	-1.51638
H	-2.24938	4.75222	-1.50096

Table S15. Optimized geometry (Cartesian coordinates, Å) of S2-conf-2 (to 2f)

C	-0.57728400	-0.93563000	-0.35950000
C	-0.01507400	0.38851300	-0.14025500
C	0.32074800	-1.96087600	-0.75287800
C	-1.97399600	-1.28555800	-0.23096300
C	-2.43492400	-2.42050800	-0.93753100
C	-0.16731700	-3.16834300	-1.33230500
C	-1.50619700	-3.34029900	-1.50955700
C	-2.92047800	-0.58662200	0.62826200
C	-3.84076300	-2.67585800	-1.04706500
C	1.70853000	-1.79314900	-0.51250500
H	0.54479600	-3.92903500	-1.65963900
H	-1.89171800	-4.22430800	-2.02157100
C	1.34375500	0.50139100	0.22935400
C	-0.76187800	1.61612000	-0.36728400
C	-0.23173100	2.84795300	0.10172900
C	1.86674100	1.76848700	0.65052600
C	1.08662900	2.88139500	0.65273300
C	-1.97474600	1.65871800	-1.09992200
C	-0.97774700	4.04209600	-0.04987900
C	2.20176600	-0.64428600	0.07082600
H	1.48642000	3.83460600	1.00548600
C	-2.52648700	0.29513500	1.66382000
C	-4.30826800	-0.84947900	0.47837200
C	-5.24802600	-0.13838400	1.26245800
C	-3.45660300	0.95596800	2.43599100
C	-4.83381000	0.76453000	2.21521200
C	-4.74332800	-1.88182400	-0.41569700
H	-6.31191500	-0.33823700	1.11559800
H	-1.46877600	0.44384600	1.87550000
H	-3.12091100	1.62506600	3.23055800
H	-5.56590600	1.30296700	2.81983800
C	-2.66868400	2.83593600	-1.26441000
H	-3.59746800	2.83748000	-1.83768300
C	-2.18639800	4.03656800	-0.70629400
H	-5.81479300	-2.06210400	-0.52764000
H	-4.17055700	-3.51991500	-1.65628100
H	2.35841300	-2.65926700	-0.67332900
C	3.61941100	-0.62546100	0.46006000
H	-0.56366600	4.97419100	0.34130200
H	-2.36253400	0.75091700	-1.55843900
C	4.61161600	-0.94675600	-0.50886700
C	4.07781200	-0.36119000	1.75498300
C	4.19953000	-1.04202900	-1.83175300
C	5.99578300	-1.07155200	-0.20116200
C	5.42855800	-0.47230600	2.06269800
H	3.35303000	-0.10728500	2.53018000
H	5.75499500	-0.28920200	3.08879700
C	6.38720300	-0.83463400	1.09638300
H	6.71892900	-1.33304800	-0.97531200
H	7.43876600	-0.92035200	1.37250100
H	2.90756900	1.84620300	0.96771800
O	3.96801600	-1.07103400	-2.93665100
H	-2.75349400	4.96162300	-0.82493800

Table S16. Optimized geometry (Cartesian coordinates, Å) of S3-conf-2f

C	-0.46067400	-0.85252300	-0.25575000
C	0.04529300	0.46152100	0.11127900
C	0.49187200	-1.80022000	-0.68076300
C	-1.85854300	-1.20045900	-0.33060500
C	-2.22417000	-2.25194400	-1.20366300
C	0.10548000	-2.91265200	-1.44599800
C	-1.22100900	-3.07193200	-1.77538800
C	-2.89654100	-0.58491800	0.48016400
C	-3.61038000	-2.49811200	-1.48961300
C	1.88994200	-1.65592500	-0.20514100
H	0.86395700	-3.60400700	-1.81315200
H	-1.52297900	-3.88339200	-2.44134400
C	1.36077300	0.61795000	0.68911400
C	-0.69207800	1.66642500	-0.17948300
C	-0.31523300	2.88427900	0.45861200
C	1.72875100	1.88278000	1.28664500
C	0.89163500	2.94082400	1.24136800
C	-1.76856700	1.69839500	-1.10414000
C	-1.09513100	4.03913400	0.26539200
C	2.27694200	-0.40267600	0.48412400
H	1.15915000	3.88198600	1.72520100
C	-2.62258500	0.19827400	1.62751100
C	-4.25581800	-0.82737000	0.14816000
C	-5.27926600	-0.18243100	0.88192600
C	-3.63504700	0.79979300	2.34202700
C	-4.97729700	0.63390400	1.94911300
C	-4.58189100	-1.77125600	-0.88370700
H	-6.31962800	-0.36287800	0.60242700
H	-1.59665900	0.30864100	1.97968300
H	-3.39655000	1.39043500	3.22846200
H	-5.77570800	1.11916800	2.51340800
C	-2.49579700	2.84954600	-1.30547200
H	-3.31299300	2.85559400	-2.02810400
C	-2.18038700	4.01880800	-0.58975700
H	-5.63329600	-1.93941800	-1.12623800
H	-3.86260000	-3.27701900	-2.21191800
H	1.99943200	-2.44727900	0.57083200
C	3.74254700	-0.38463100	0.56775100
H	-0.81593300	4.96040500	0.78039800
H	-2.01085600	0.80928100	-1.68330700
C	4.24585500	-1.23988700	-0.43524000
C	4.63928500	0.30284700	1.39293500
C	3.12781800	-1.95146000	-1.10454800
C	5.60623000	-1.39322700	-0.66617300
C	6.00596100	0.14032700	1.17192300
H	4.30307400	0.92349600	2.22269700
H	6.71510400	0.66364800	1.81595200
C	6.48850900	-0.68207500	0.14644300
H	5.95947900	-2.05456700	-1.45939500
H	7.56521400	-0.77334700	-0.00795100
H	2.69221800	1.97643600	1.77969800
O	3.14925100	-2.65007200	-2.07327500
H	-2.77379800	4.92319600	-0.73775500

Table S17. Optimized geometry (Cartesian coordinates, Å) of the transition state between S2-conf-2 to S3-conf-2f

C	-0.50621	-0.94019	-0.24118
C	0.03793	0.38295	0.04650
C	0.41902	-1.96003	-0.56571
C	-1.90873	-1.28174	-0.24838
C	-2.30552	-2.40719	-1.00889
C	-0.01090	-3.15919	-1.19229
C	-1.33121	-3.32315	-1.49745
C	-2.92859	-0.58501	0.52359
C	-3.69698	-2.65133	-1.25639
C	1.78784	-1.79210	-0.19006
H	0.72529	-3.92094	-1.45774
H	-1.66791	-4.20015	-2.05407
C	1.35878	0.50165	0.54830
C	-0.67885	1.60890	-0.25118
C	-0.20090	2.83841	0.28103
C	1.83827	1.76675	1.02713
C	1.05569	2.87392	0.96476
C	-1.81236	1.65297	-1.10277
C	-0.93313	4.02963	0.06588
C	2.22646	-0.62633	0.44236
H	1.41073	3.82549	1.36569
C	-2.62924	0.28104	1.60277
C	-4.29650	-0.83482	0.23555
C	-5.30210	-0.12330	0.93202
C	-3.62468	0.94310	2.28761
C	-4.97495	0.76638	1.93047
C	-4.65000	-1.85584	-0.70772
H	-6.34834	-0.31223	0.68115
H	-1.59627	0.41497	1.92130
H	-3.36342	1.59883	3.12029
H	-5.75833	1.30438	2.46734
C	-2.48977	2.82933	-1.32680
H	-3.35505	2.83514	-1.99175
C	-2.06950	4.02482	-0.71040
H	-5.70683	-2.02664	-0.92423
H	-3.97191	-3.48755	-1.90240
H	2.39321	-2.70275	-0.13001
C	3.68746	-0.56453	0.63209
H	-0.56609	4.95995	0.50477
H	-2.14745	0.74786	-1.60573
C	4.36308	-0.96714	-0.53596
C	4.44485	-0.19820	1.74220
C	3.45623	-1.26038	-1.60276
C	5.76181	-1.05907	-0.63063
C	5.83493	-0.28092	1.66184
H	3.95977	0.11121	2.66897
H	6.43307	-0.01663	2.53642
C	6.48994	-0.70456	0.49431
H	6.25014	-1.38030	-1.55175
H	7.57978	-0.74668	0.46926
H	2.84144	1.83779	1.44676
O	3.18173	-1.38420	-2.70564
H	-2.62501	4.94953	-0.87716
